# Saturated oxygen and nitrogen heterocycles *via* oxidative coupling of alkyltrifluoroborates with alkenols, alkenoic acids and protected alkenylamines†
†Electronic supplementary information (ESI) available: Experimental procedures, NMR spectra of all new intermediates and products. See DOI: 10.1039/c9sc02835h


**DOI:** 10.1039/c9sc02835h

**Published:** 2019-08-19

**Authors:** Jonathan M. Shikora, Chanchamnan Um, Zainab M. Khoder, Sherry R. Chemler

**Affiliations:** a Department of Chemistry , State University of New York at Buffalo , Natural Science Complex , Buffalo , New York 14260 , USA . Email: schemler@buffalo.edu

## Abstract

Saturated heterocycles are important components of many bioactive compounds. The method disclosed herein enables a general route to a range of 5-, 6- and 7-membered oxygen and nitrogen heterocycles by coupling potassium alkyltrifluoroborates with heteroatom-tethered alkenes, predominantly styrenes, under copper-catalyzed conditions, in the presence of MnO_2_. The method was applied to the synthesis of the core of the anti-depressant drug citalopram. The reaction scope and observed reactivity is consistent with a polar/radical mechanism involving intermolecular addition of the alkyl radical to the alkene followed by [Cu(iii)]-facilitated C–O (or C–N) bond forming reductive elimination.

## Abstract 2

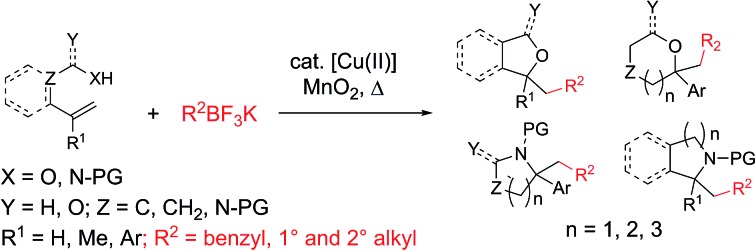
The oxidative coupling of alkyltrifluoroborates with heteroatom-tethered vinylarenes leads to a broad range of saturated oxygen and nitrogen heterocycles.

## Introduction

**Scheme 1:**
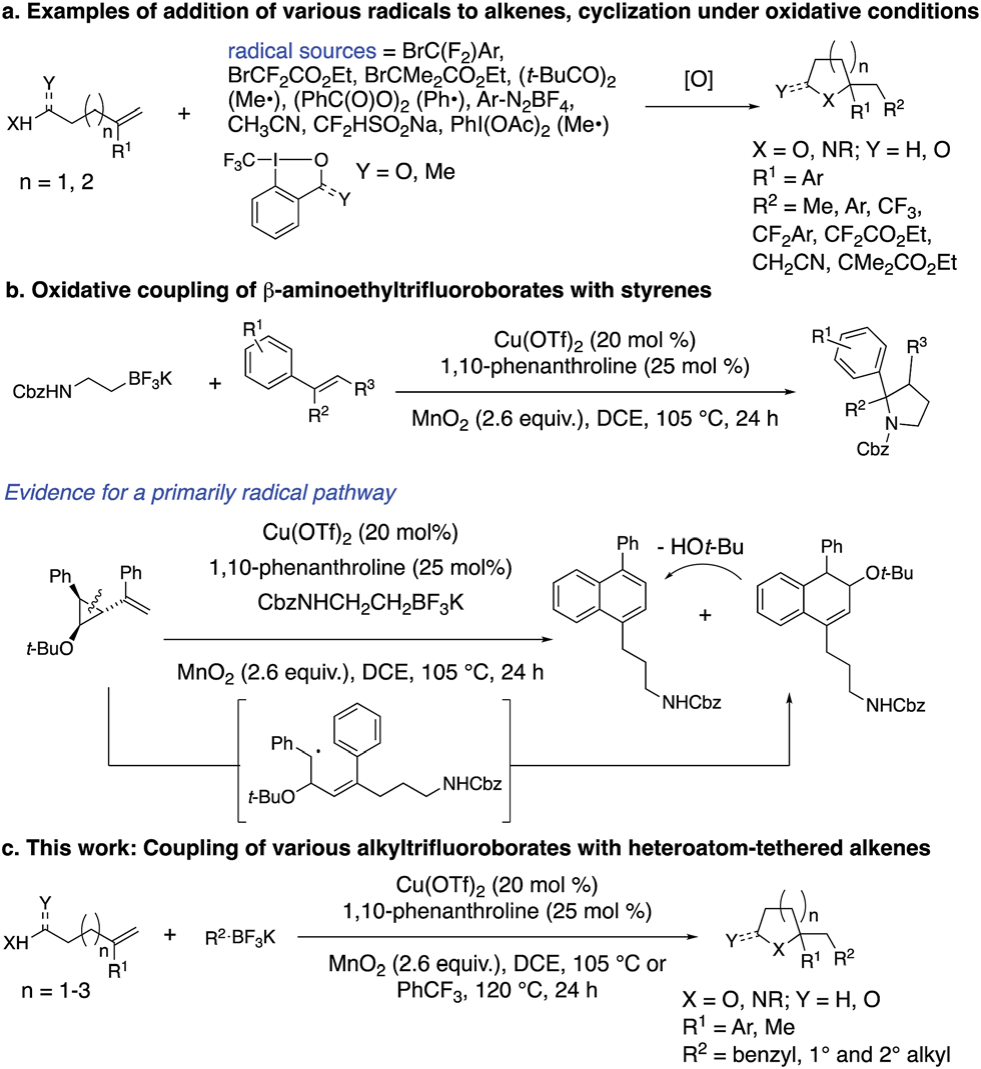
Radical additions to alkenes and oxidative cyclizations thereof.

The flexible, convergent synthesis of saturated oxygen and nitrogen heterocycles can be accomplished by the addition of a carbon radical to readily available alkenoic acids, alkenols and alkenyl amines under oxidative conditions (Scheme 1a).^1^–^10^

The carbon radical source is an important variable in these alkene addition processes. Alkylhalides have been applied, in particular when their metal-catalyzed reduction forms stabilized or semi-stabilized carbon radicals, such as those conjugated with carbonyl groups^11^ or that are perhalogenated.^10^ Halosulfonate salts that form radicals by oxidative desulfonation have also been used.^4^,^5^ Hypervalent iodine reagents that decompose in the presence of metal catalysts to generate alkyl radicals have been used in such alkene coupling reactions.^2^,^3^,^6^–^8^ Nitriles, whose alpha positions can be deprotonated and oxidized, have been applied as the stabilized alkyl radical component.^9^ Less stable alkyl radicals, generated by decomposition of peroxides^1^ and from aryldiazonium salts, have also been used.^3^ Benzyl and other simple alkyl radicals are generally lacking in these radical addition/cyclization methodologies.

We and others have been exploring the use of alkyltrifluoroborates as radical precursors in oxidative coupling reactions.^12^ Oxidation of the alkyltrifluoroborates by metal catalysts under either thermal or photochemical activation enables the generation of a range of alkyl radicals that can undergo direct addition or metal-catalyzed coupling with appropriate coupling partners including alkenes. Primary, secondary and benzylic radicals are among the alkyl radicals formed under such oxidative conditions from alkyltrifluoroborates.

We recently reported on the synthesis of 2-arylpyrrolidines *via* copper-catalyzed oxidative coupling of styrenes with potassium *N*-carbamolyl-β-aminoethyl trifluoroborates (Scheme 1b).^13^ In these reactions, the [Cu] salt oxidizes the alkyltrifluoroborate to a primary carbon radical. Addition of the radical to the styrene, followed by cyclization *via* addition of the pendant carbamate under the oxidative conditions generates the 2-arylpyrrolidine. A radical clock experiment supported a mechanism involving primarily carbon radical intermediates (as opposed to carbocation).^13^ Herein we present a new approach to the synthesis of a broad range of saturated heterocycles by addition of various potassium alkyltrifluoroborates to heteroatom-functionalized alkenes, primarily styrenes.

## Results and discussion

The oxidative coupling/cyclization of 2-(1-phenylvinyl)benzoic acid **1a** with potassium benzyltrifluoroborate (limiting reagent) was investigated as illustrated in Table 1. Reaction variables such as substrate and copper loading, solvent, temperature, ligand and oxidant were explored. Coupling **1a** (1 equiv.) with BnBF_3_K in the presence of 20 mol% Cu(OTf)_2_, 25 mol% 1,10-phenanthroline (**3a**) and MnO_2_ (2.6 equiv.) in 1,2-dichloroethane (DCE) at 105 °C gave 78% of lactone **2a** (Table 1, entries 1). An increase of **1a** loading to 1.5 equiv. resulted in a higher isolated yield of **2a** (84%, Table 1, entry 2). A decrease in Cu(OTf)_2_ loading to 5 and 10 mol% both gave 71% isolated yield of lactone **2a** (Table 1, entries 3 and 4). In the absence of [Cu], lactone **2a** is still formed, albeit in diminished yield (51%, Table 1, entry 5 and see below Scheme 4 and associated discussion). The reactions in toluene and dioxane were less productive while the reaction in PhCF_3_ gave a comparable yield to DCE (Table 1, entries 6–8). The use of other ligands, **3b–e** and **4a**, or no ligand, gave reactions with equivalent or lower yields (Table 1, entries 9–14). When Ag_2_CO_3_ or K_2_S_2_O_8_ were used instead of MnO_2_, lower yields were obtained (Table 1, entries 15 and 16). The reaction scale with respect to limiting BnBF_3_K could be run on 1 mmol scale (73% isolated **2a**, Table 1, entry 17).

**Table 1:** Reaction optimization^*a*^

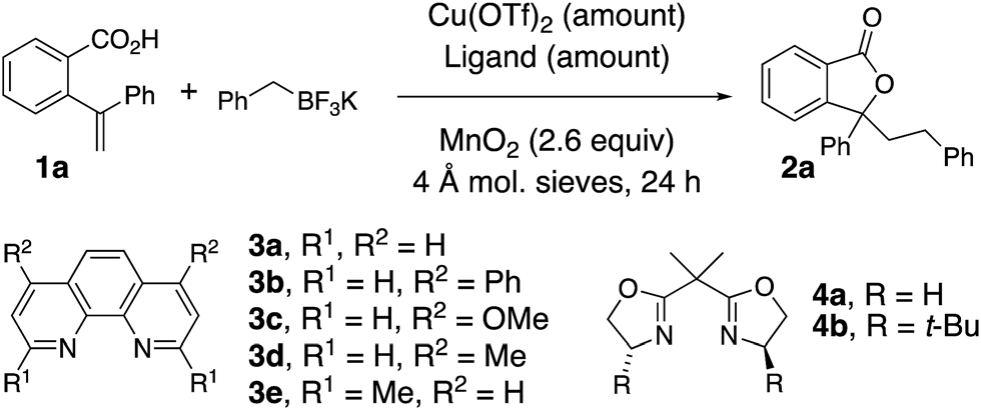					
Entry	Mol% Cu(OTf)_2_	Ligand (mol%)	Solvent	Temp (°C)	Yield (%)
1^*b*^	20	**3a** (25)	DCE	105	78
2	20	**3a** (25)	DCE	105	84
3	5	**3a** (6)	DCE	105	71
4	10	**3a** (12)	DCE	105	71
5	—	—	DCE	105	51
6	20	**3a** (25)	PhCH_3_	120	48
7	20	**3a** (25)	PhCF_3_	120	82
8	20	**3a** (25)	Dioxane	120	71
9	20	**3b** (25)	DCE	105	62
10	20	**3c** (25)	DCE	105	84
11	20	**3d** (25)	DCE	105	77
12	20	**3e** (25)	DCE	105	66
13	20	**4a** (25)	DCE	105	71
14	20	—	DCE	105	54
15^*c*^	20	**3a** (25)	DCE	105	67
16^*d*^	20	**3a** (25)	DCE	105	35
17^*e*^	20	**3a** (25)	DCE	105	73

^*a*^All reactions were run in a sealed tube under argon with 0.125 mmol BnBF_3_K and 1.5 equiv. of acid **1a** in DCE (0.125 mM) unless otherwise noted.

^*b*^Reaction run with 1 equiv. of acid **1a**.

^*c*^Reaction run with Ag_2_CO_3_ (1.3 equiv.) instead of MnO_2_.

^*d*^Reaction run with K_2_S_2_O_8_ (2.6 equiv.) instead of MnO_2_.

^*e*^Reaction run with 1 mmol BnBF_3_K.

Using the optimal reaction conditions (Table 1, entry 2), the alkyltrifluoroborate scope was next explored in the coupling/cyclization reaction with alkenoic acids **1** (Table 2). Alkyltrifluoroborates such as benzylic, primary, methyl, ethyl, neopentyl, allylic and secondary, and alkyls functionalized with a nitrile, an acetal and a carbamate all underwent the coupling reaction with varying levels of efficiency. A *tert*-butyl ester-functionalized alkyltrifluoroborate was also a viable coupling partner [see Table 4 (**8g**) and Table 5 (**12j**)]. Neither CF_3_BF_3_K nor *t*-BuOCH_2_BF_3_K gave the desired coupling product with **1a**.

**Table 2:** Trifluoroborate scope^*a*^

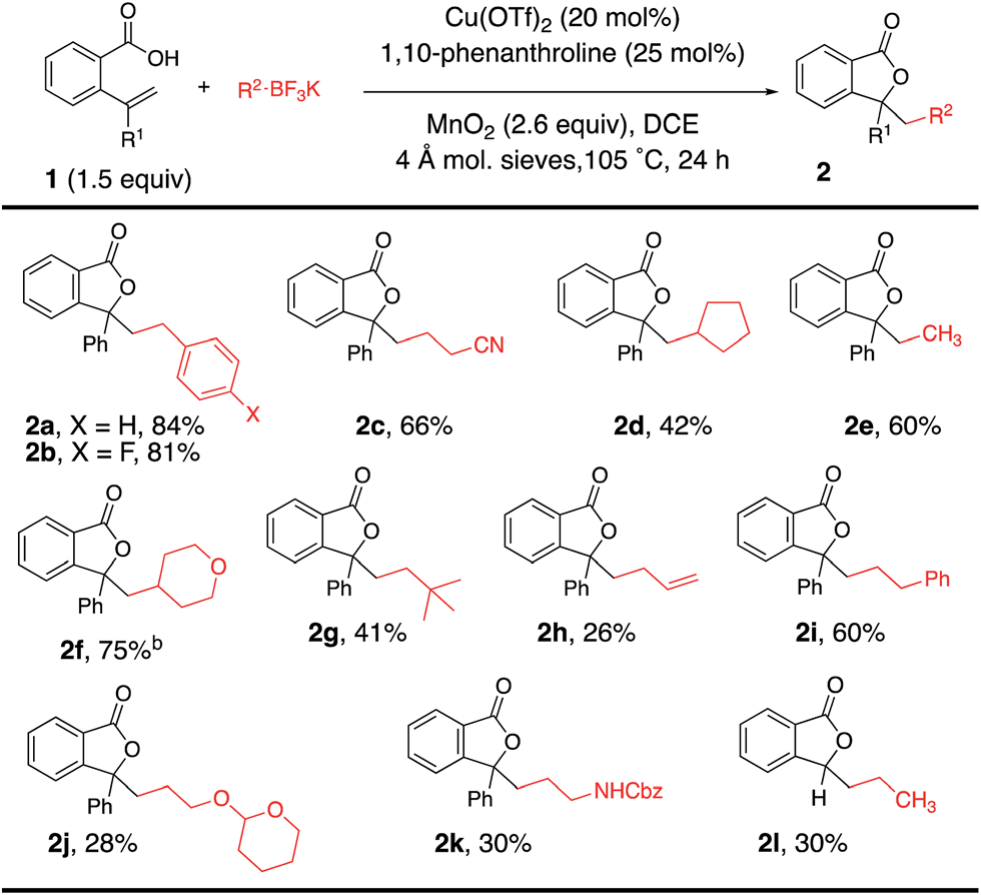

^*a*^Reaction conditions from Table 1, entry 2 were used unless otherwise noted.

^*b*^10 mol% Cu(OTf)_2_ and 12 mol% 1,10-phenanthroline was used.

Alkenoic acid substituent effects were further explored (Tables 2 and 3). Diaryl alkenes, such as **1a**, reacted most efficiently to give phthalide **2a** in 84% (Table 2). Replacing the phenyl with methyl (Table 3, **2m**, 64%) or H (Table 3, **2n**, 39%) gave lower phthalide yields. Substitution on the arene conjugated to the alkene was tolerated, and lactones **5a–f** were formed in good yields. A 1,1-dialkyl substituted alkene did undergo addition and cyclization but the desired product was generated in much lower yield (**5g**, 12%). The remainder of the mass in the lower yielding reactions included unreacted alkenoic acid, (PhCH_2_)_2_ (benzyl radical dimerization)^14^ and benzyl alcohol. Both 6-membered (**6a** and **6b**) and 7-membered ring lactones (**7a** and **7b**) could also be formed. The 2,2′-disubstituted biaryl-backboned alkenoic acid provided **7b** more efficiently than the largely aliphatic-backboned heptenoic acid gave **7a**.

**Table 3:** Alkenoic acid scope^*a*^

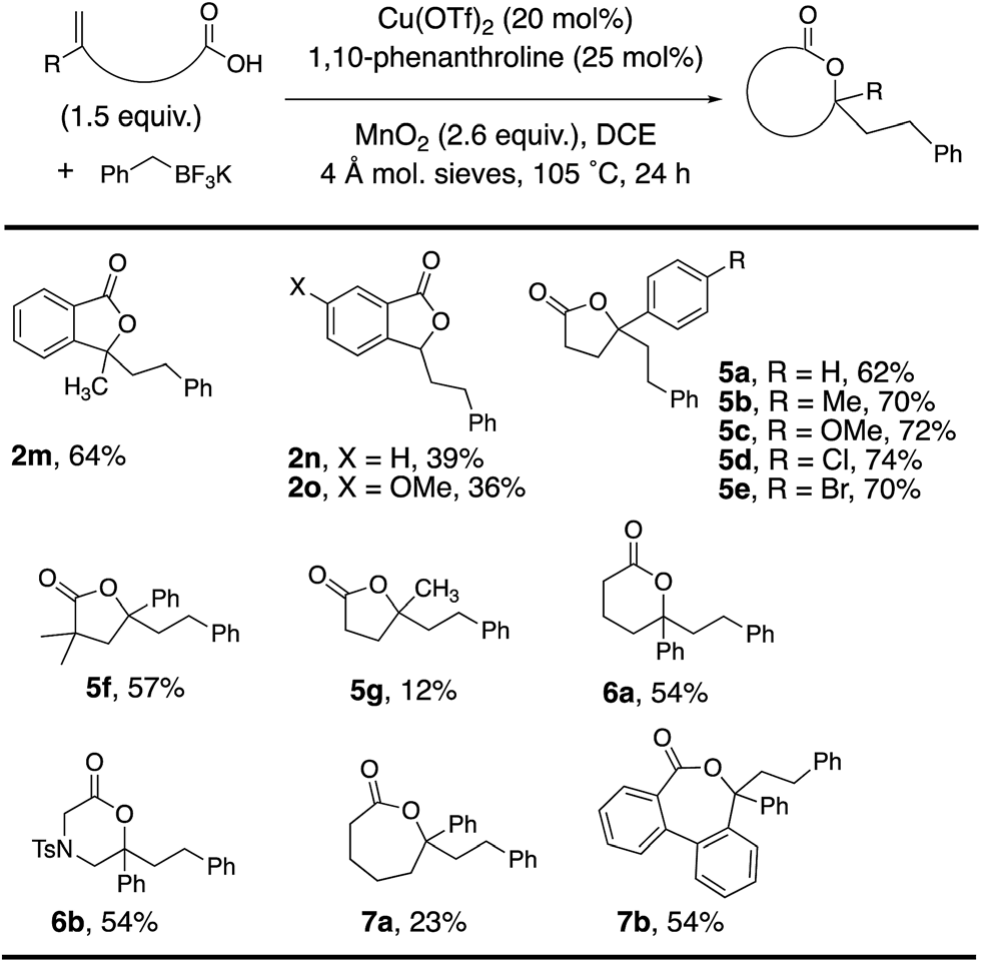

^*a*^Reaction conditions from Table 1, entry 2 were used unless otherwise noted.

The scope was next expanded to alkenol coupling/cyclization for the synthesis of saturated oxygen heterocycles (Table 4). The synthesis of tetrahydrofurans (**8a–j**), phthalans (**9a–c**), isochromans (**10a**,** 10b**), a pyran (**10c**), morpholines (**10d**, **10e**) and oxepanes (**11a**, **11b**) is enabled by this oxidative coupling reaction. Phthalan **9c**, product of coupling of potassium *N*-Cbz-β-aminoethyltrifluoroborate with the respective 4-cyanophenyl-functionalized alkenol, is a reasonable intermediate for the synthesis of citalopram,^9^,^15^ a drug used for the treatment of depression.

**Table 4:** Alkenol scope^*a*^

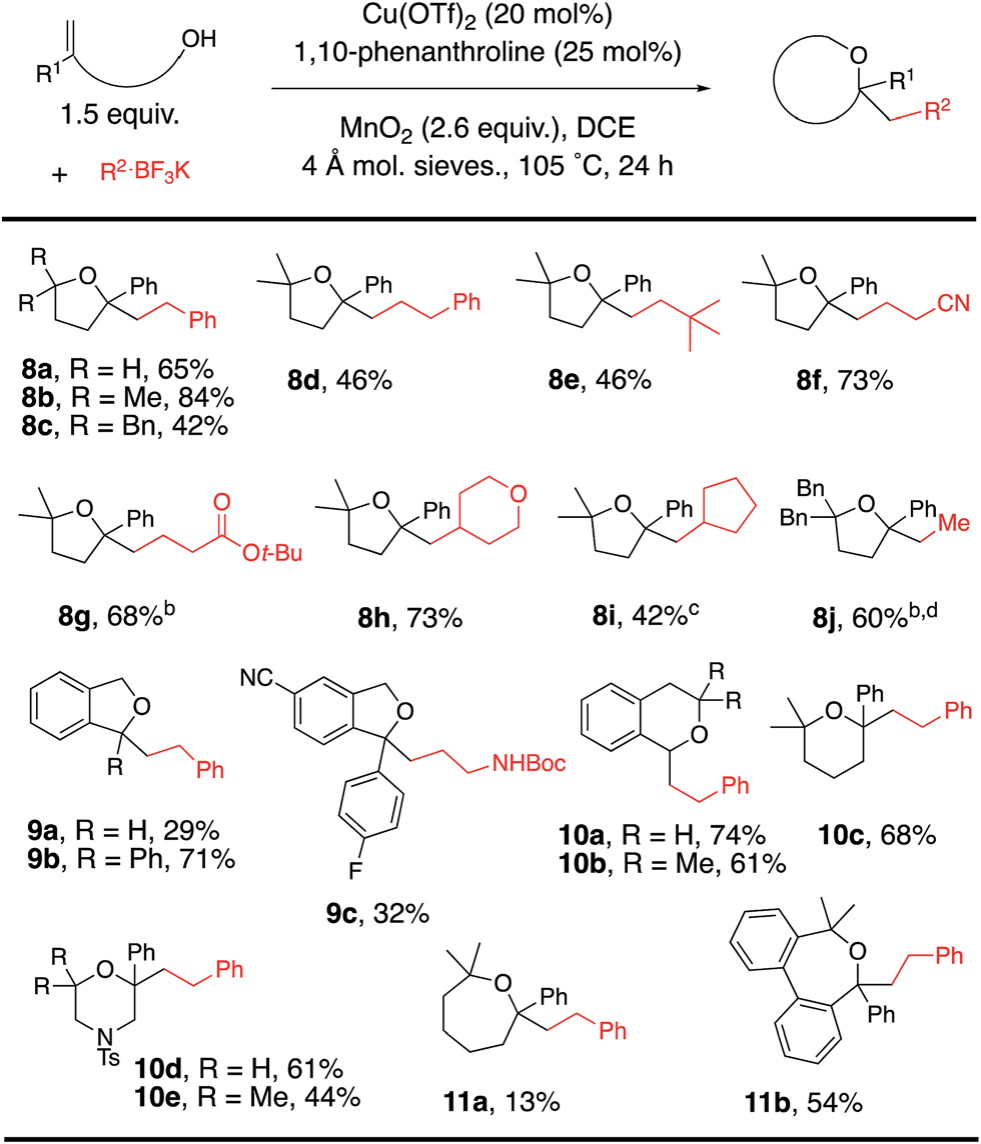

^*a*^Reaction conditions from Table 1, entry 2 were used unless otherwise noted.

^*b*^Reaction run in PhCF_3_ at 120 °C.

^*c*^Reaction run with 10 mol% Cu(OTf)_2_, 12 mol% 1,10-phenanthroline.

^*d*^Reaction run for 48 h.

The reaction was expanded to protected alkenyl amine coupling/cyclization for the synthesis of saturated nitrogen heterocycles (Table 5). In this reaction higher alkenylamine loading (3 equiv., 57% of **12c**) gave notably higher isolated yield (32% of **12c** was obtained when 2 equiv. of alkenyl amide was used). Variously *N*-substituted alkenylamines produced pyrrolidines (**12a–f**, **12h–l**), a cyclic urea (**12g**), an isoindoline (**13**), a tetrahydroisoquinoline (**14**) and a tetrahydrobenzoazepine (**15**).

**Table 5:** Alkenyl amide scope^*a*^

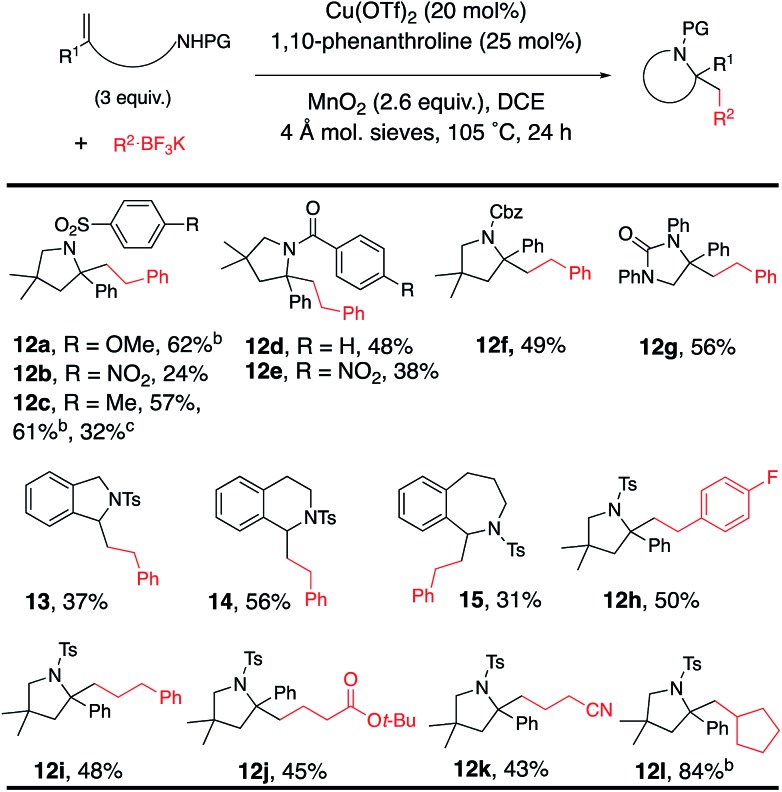

^*a*^Reaction conditions from Table 1, entry 2 were used except 3 equiv. of alkenylamide was used.

^*b*^Reaction run in PhCF_3_ at 120 °C.

^*c*^Reaction run with 2 equiv. alkenylamide. PG = protecting group.

### Enantioselective conditions

**Scheme 2:**
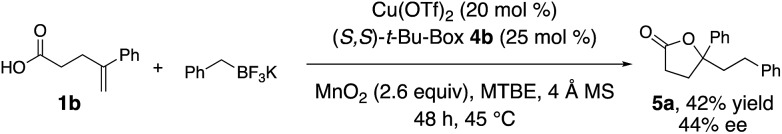
Ligand-induced enantioselectivity.

We explored the potential for a chiral ligand to control absolute stereochemistry in our oxidative coupling. A brief substrate and ligand screen revealed that 4-phenylpent-4-enoic acid (**1b**) can undergo enantioselective coupling/cyclization under minimally modified (lower temperature, *t*-BuOMe as solvent) reaction conditions using the (*S*,*S*)-*t*-Bu-Box **4b** (Scheme 2, 42% yield, 44% ee).^2^,^3^ In the absence of [Cu], 15% of lactone **5a** was formed as a racemate, indicating some background reaction can occur. In the absence of MnO_2_, using 50 mol% copper loading and 55 mol% of **4b**, **5a** was obtained in 22% yield and 44% ee, indicating the potential MnO_2_ promoted background reaction is unlikely to be affecting the reaction's enantioselectivity.

### Proposed mechanism

**Scheme 3:**
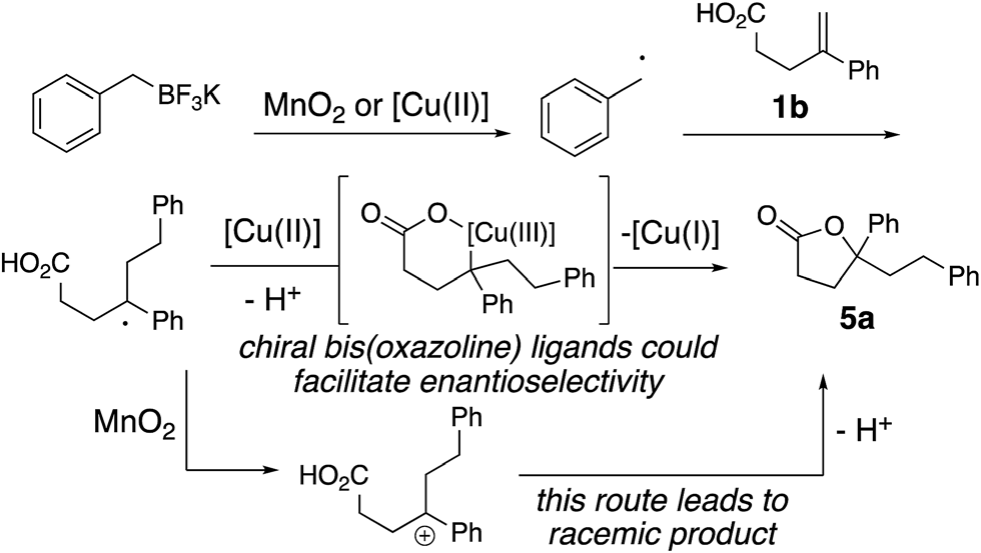
Proposed mechanism.

The proposed mechanism is illustrated in Scheme 3. Either copper(ii) or MnO_2_, or a mixture of both, oxidizes the alkyltrifluoroborate to the corresponding radical.^14^ Addition of the resulting alkyl radical to **1b** provides a benzylic radical intermediate. The enantioselective result in Scheme 2 supports the involvement of a chiral ligand-bound copper complex in the C–O bond formation. Thus, formation of an alkyl copper(iii) intermediate *via* addition of the alkyl radical to the [Cu(ii)] complex and subsequent C–O bond formation *via* reductive elimination generates lactone **5a** (Scheme 3).^3^,^16^ The [Cu(ii)] is regenerated by oxidation of [Cu(i)] to [Cu(ii)] by MnO_2_ to continue the catalytic cycle (not shown). The background reaction observed under MnO_2_-only conditions likely occurs *via* the alternative benzylic carbocation intermediate, which would be expected to give racemic **5a** (Scheme 3).

### MnO_2_-promoted reactions

**Scheme 4:**
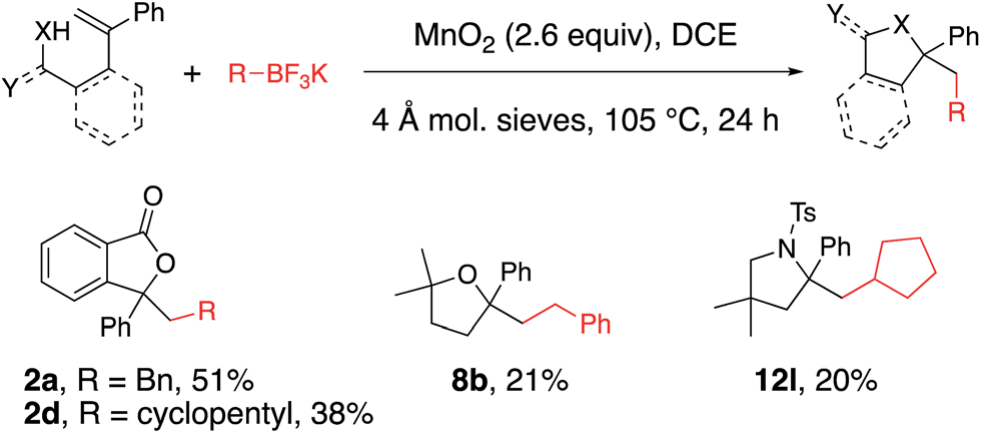
MnO_2_-only oxidative cyclization.

The possibility of an oxidative coupling involving only MnO_2_ as oxidant was further investigated (Scheme 4). Manganese salts have previously demonstrated the ability to oxidize alkyltrifluoroborates and boronic acids to radicals, that then undergo oxidative coupling with alkenoic acids^17^ or heteroarenes (Minisci reaction).^18^ Under reaction conditions analogous to our alkyltrifluoroborate couplings, but in the absence of any [Cu] salt, we found that alkenoic acids, alkenyl alcohols and alkenyl amines do undergo the oxidative cyclizations, albeit with generally lower efficiency. The secondary cyclopentyl trifluoroborate reacted similarly with alkenoic acid **1a** in the presence of MnO_2_, both with and without [Cu] catalyst (42% *vs.* 38% of **2d**, compare Table 2 to Scheme 4), while benzyltrifluoroborate gave notably higher yield with the catalytic [Cu] conditions (84% *vs.* 51% of **2a**, compare Table 1, entry 3 to Scheme 4). In the absence of [Cu] catalyst, alkenol and alkenyl amide substrates gave considerably lower yields of their corresponding heterocycles **8b** (84% with [Cu] *vs.* 21% without, Table 4 and Scheme 4) and **12l** (84% with [Cu] *vs.* 20% without, Table 5 and Scheme 4). In the MnO_2_ promoted reactions it is likely the C–O and C–N bond formations involve addition of the heteroatom to a carbocationic intermediate (Scheme 3).^17^

## Conclusions

The copper-catalyzed addition of alkyltrifluoroborates to heteroatom-tethered vinyl arenes under oxidative conditions has been demonstrated to be a general route to saturated oxygen and nitrogen heterocycles. The possibility of asymmetric catalysis has been demonstrated. The observed reactivity is largely consistent with alkyl radical addition to the vinyl arene and [Cu(iii)]-facilitated C–O and C–N bond formation. A good range of alkyltrifluoroborates serve as alkyl radical source; these reagents are complementary to radical precursors that generally involve generation of more stabilized radicals.

## Conflicts of interest

There are no conflicts to declare.

## Supplementary Material

Supplementary information

Supplementary information
