# Supplementary material for: Saturated oxygen and nitrogen heterocycles via oxidative coupling of alkyltrifluoroborates with alkenols, alkenoic acids and protected alkenylamines
Source: Chem Sci. 2019 Aug 19;10(40):9265–9. doi: 10.1039/c9sc02835h (PMC7003886; doi:10.1039/c9sc02835h)

Electronic Supplementary Material (ESI) for Chemical Science.
This journal is © The Royal Society of Chemistry 2019

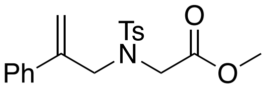

***S*-1m**

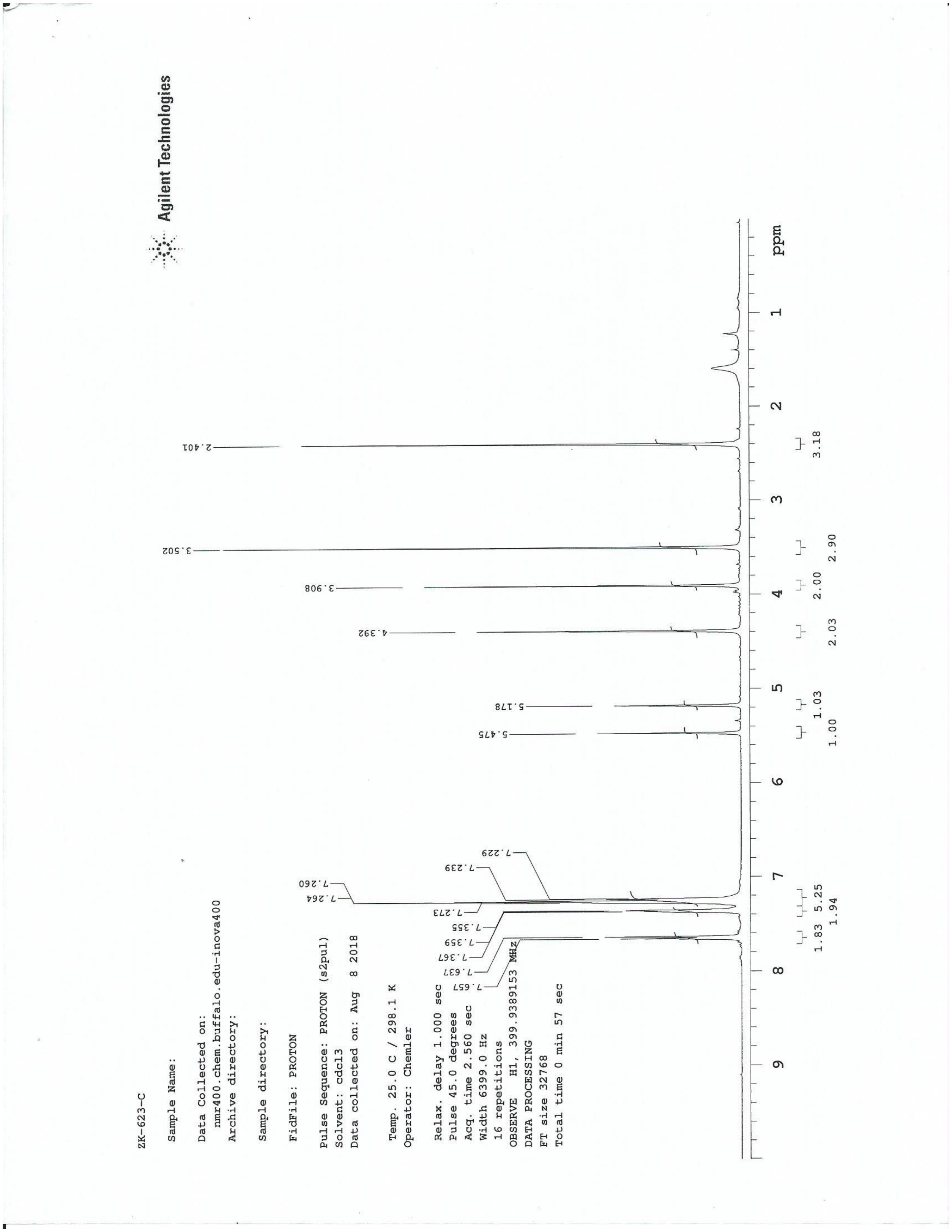

ZK-623-C

Sample Name:

Data Collected on:
nmr400.chem.buffalo.edu-inova400
Archive directory:

Sample directory:

FidFile: CARBON

Pulse Sequence: CARBON (s2pul)
Solvent: cdcl3
Data collected on: Aug 8 2018

Temp. 25.0 C / 298.1 K
Operator: Chemler

Relax. delay 2.000 sec
Pulse 45.0 degrees
Acq. time 1.303 sec
Width 25141.4 Hz
536 repetitions
OBSERVE C13, 100.5647184 MHz
DECOUPLE H1, 399.9409068 MHz
Power 33 dB
continuously on
WALTZ-16 modulated
DATA PROCESSING
Line broadening 0.5 Hz
FT size 65536
Total time 92 hr

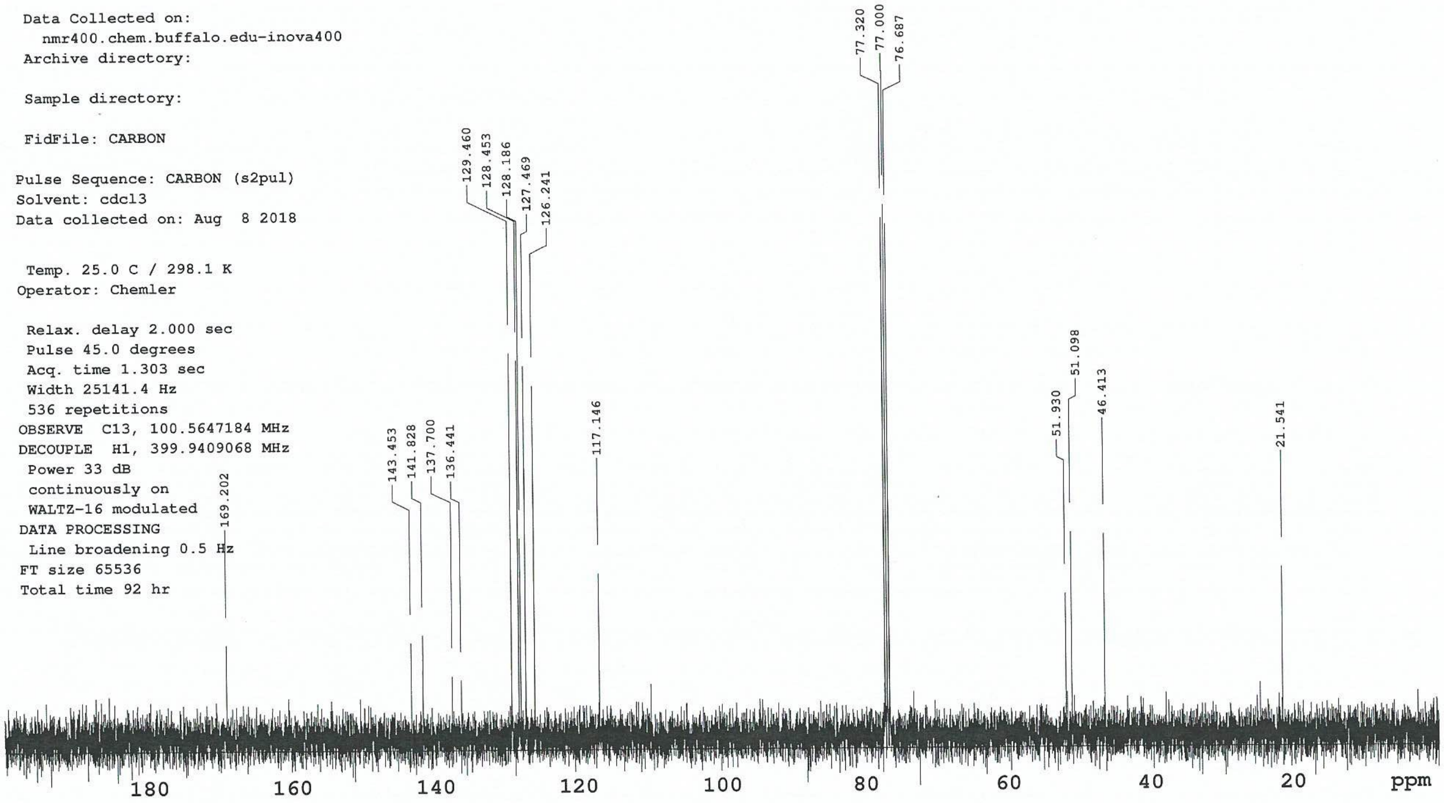

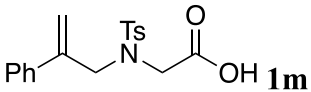
Ts
N
O
OH
Ph
1m

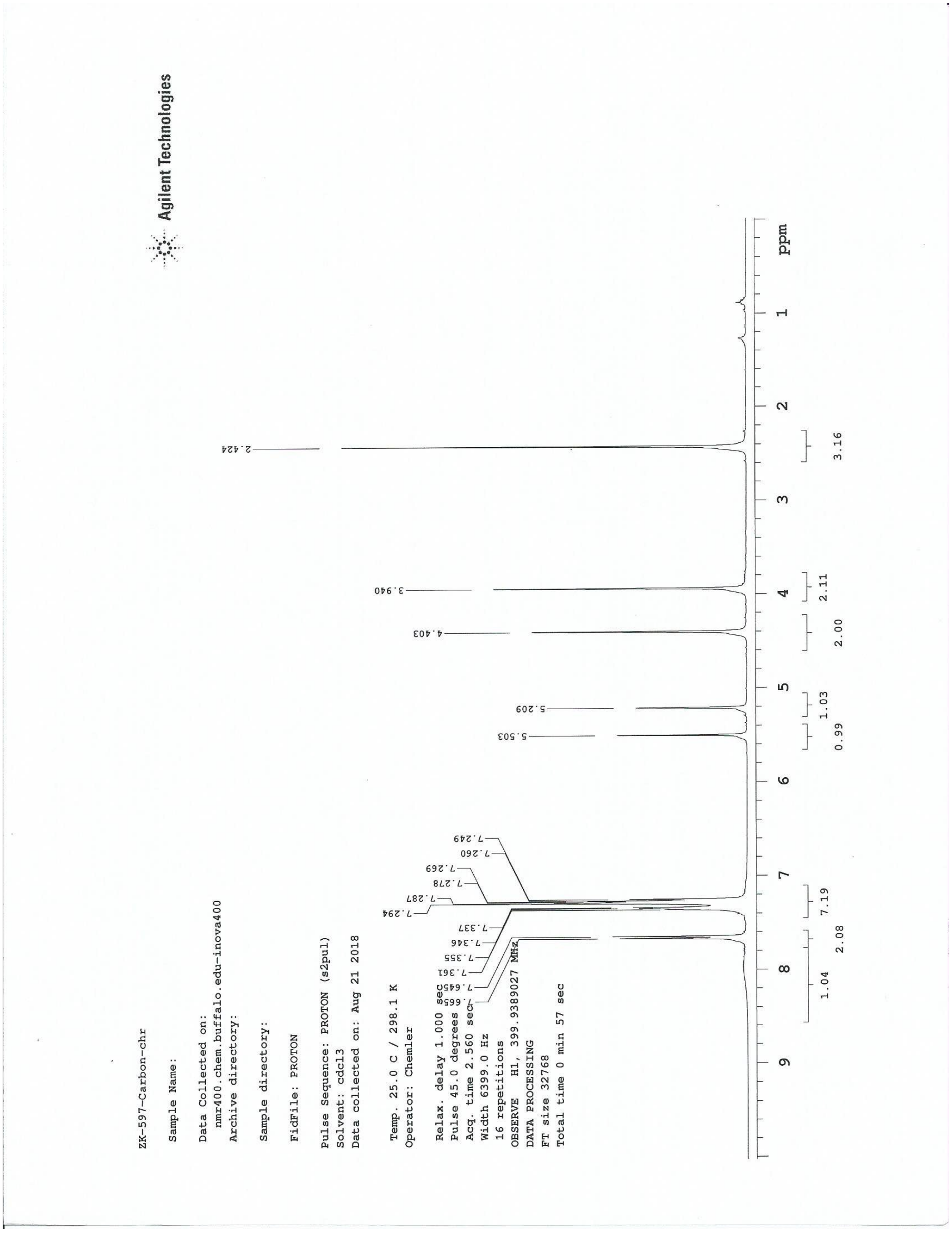
Agilent Technologies
ZK-597-Carbon-chr
Sample Name:
Data Collected on:
nmr400.chem.buffalo.edu-inova400
Archive directory:
Sample directory:
FidFile: PROTON
Pulse Sequence: PROTON (s2pul)
Solvent: cdcl3
Data collected on: Aug 21 2018
Temp. 25.0 C / 298.1 K
Operator: Chemler
Relax. delay 1.000 sec
Pulse 45.0 degrees
Acq. time 2.560 sec
Width 6399.0 Hz
16 repetitions
OBSERVE H1, 399.9389027 MHz
DATA PROCESSING
FT size 32768
Total time 0 min 57 sec
2.424
3.940
4.403
5.209
5.503
7.249
7.260
7.269
7.278
7.287
7.294
7.337
7.346
7.355
7.361
7.645
7.665
3.16
2.11
2.00
1.03
0.99
7.19
2.08
1.04
ppm
1
2
3
4
5
6
7
8
9

ZK-597-Carbon-chr

Sample Name:

Data Collected on:
nmr400.chem.buffalo.edu-inova400
Archive directory:

Sample directory:

FidFile: CARBON

Pulse Sequence: CARBON (s2pul)
Solvent: cdcl3
Data collected on: Aug 21 2018

Temp. 25.0 C / 298.1 K
Operator: Chemler

Relax. delay 1.000 sec
Pulse 45.0 degrees
Acq. time 1.303 sec
Width 25141.4 Hz
2464 repetitions
OBSERVE C13, 100.5647200 MHz
DECOUPLE H1, 399.9409068 MHz
Power 33 dB
continuously on
WALTZ-16 modulated
DATA PROCESSING
Line broadening 0.5 Hz
FT size 65536
Total time 6422 hr, 50 min

Agilent Technologies

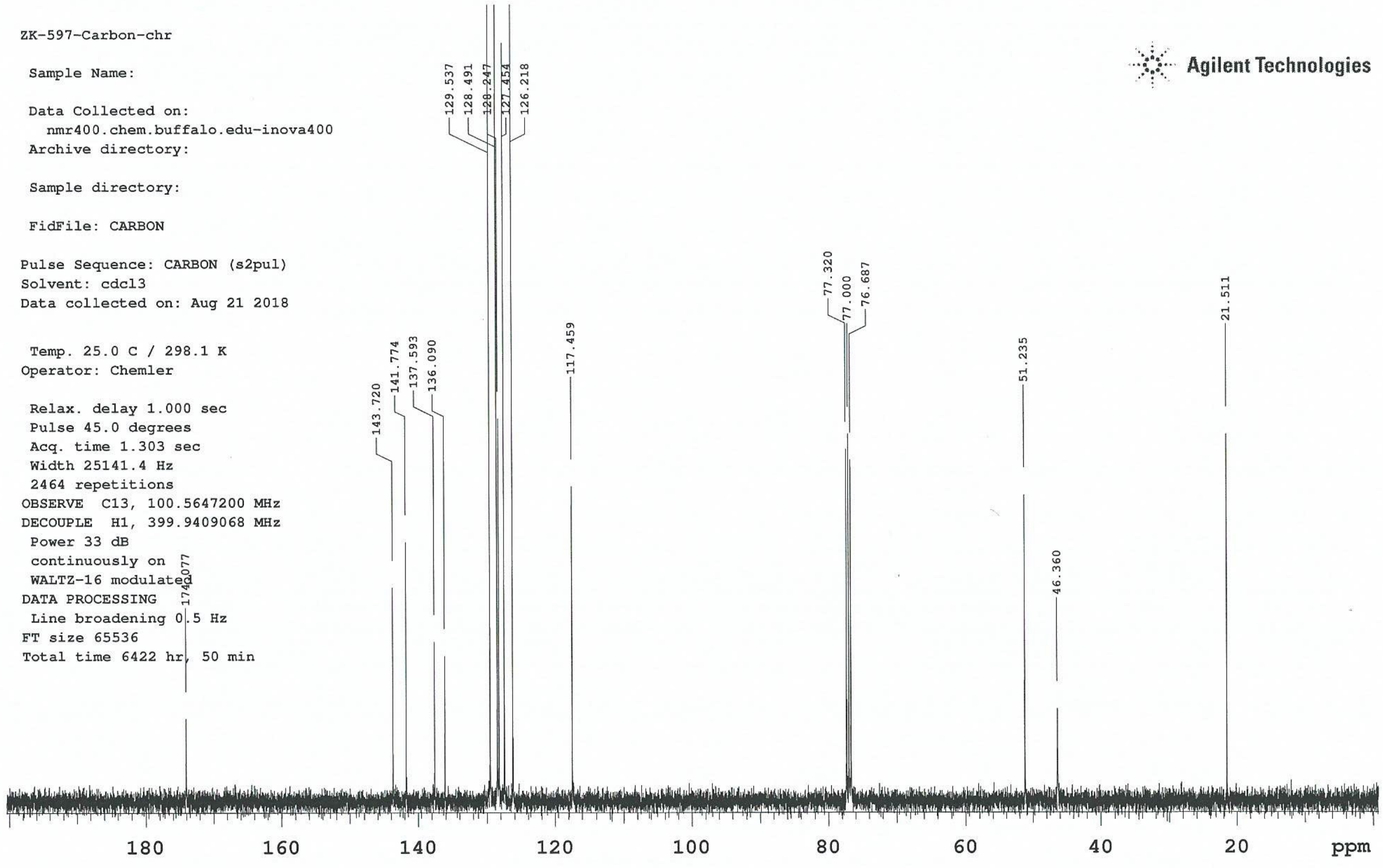

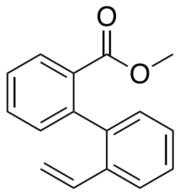

S-1o

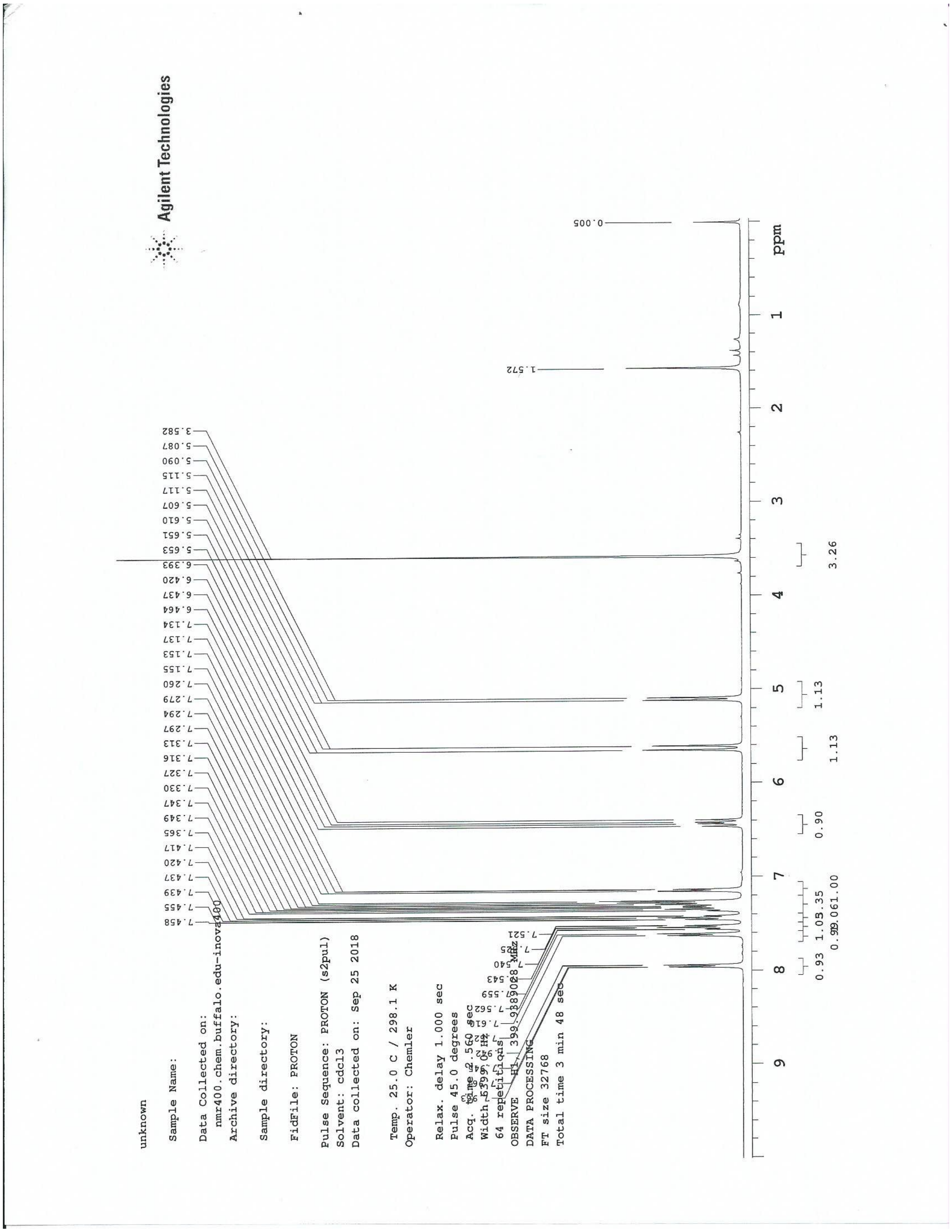
Agilent Technologies
unknown
Sample Name:
Data Collected on:
nmr400.chem.buffalo.edu-inova400
Archive directory:
Sample directory:
FidFile: PROTON
Pulse Sequence: PROTON (s2pul)
Solvent: cdcl3
Data collected on: Sep 25 2018
Temp. 25.0 C / 298.1 K
Operator: Chemler
Relax. delay 1.000 sec
Pulse 45.0 degrees
Acq. time 2.560 sec
Width 6397.4 Hz
64 repetitions
OBSERVE H1, 399.9380288 MHz
DATA PROCESSING
FT size 32768
Total time 3 min 48 sec
3.582
5.087
5.090
5.115
5.117
5.607
5.610
5.651
5.653
6.393
6.420
6.437
6.464
7.134
7.137
7.153
7.155
7.260
7.279
7.294
7.297
7.313
7.316
7.327
7.330
7.347
7.349
7.365
7.417
7.420
7.437
7.439
7.455
7.458
7.521
7.540
7.543
7.559
7.562
7.610
0.005
1.572
3.26
1.13
1.13
0.90
1.00
1.05
1.35
0.93
ppm
1
2
3
4
5
6
7
8
9

unknown

Sample Name:

Data Collected on:
nmr400.chem.buffalo.edu-inova400
Archive directory:

Sample directory:

FidFile: CARBON

Pulse Sequence: CARBON (s2pul)
Solvent: cdcl3
Data collected on: Sep 25 2018

Temp. 25.0 C / 298.1 K
Operator: Chemler

Relax. delay 1.000 sec
Pulse 45.0 degrees
Acq. time 1.303 sec
Width 25141.4 Hz
936 repetitions
OBSERVE C13, 100.5647184 MHz
DECOUPLE H1, 399.9409068 MHz
Power 33 dB
continuously on
WALTZ-16 modulated
DATA PROCESSING
Line broadening 0.5 Hz
FT size 65536
Total time 64 hr, 13 min

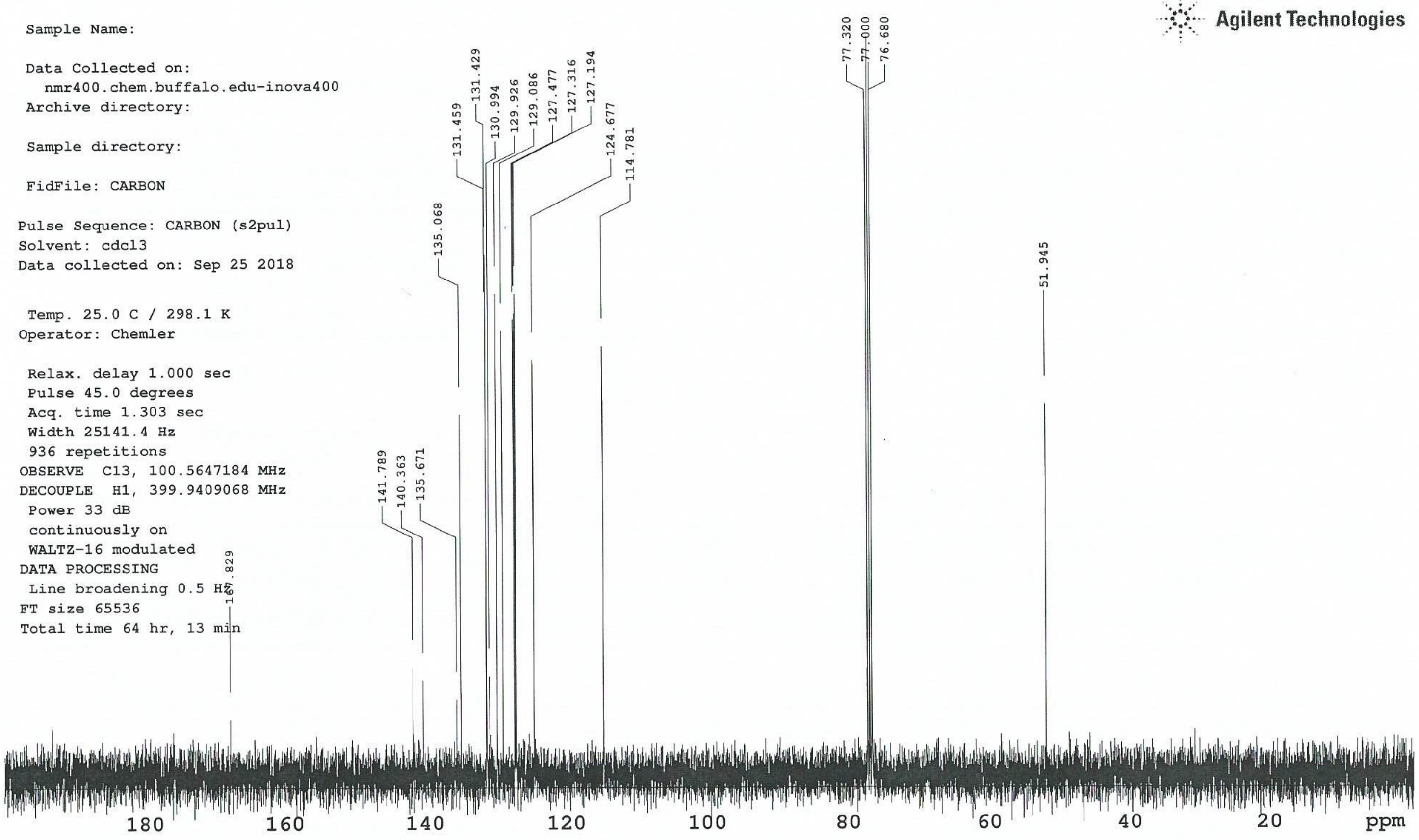

Agilent Technologies

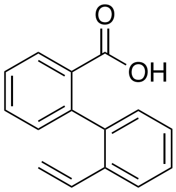

1o

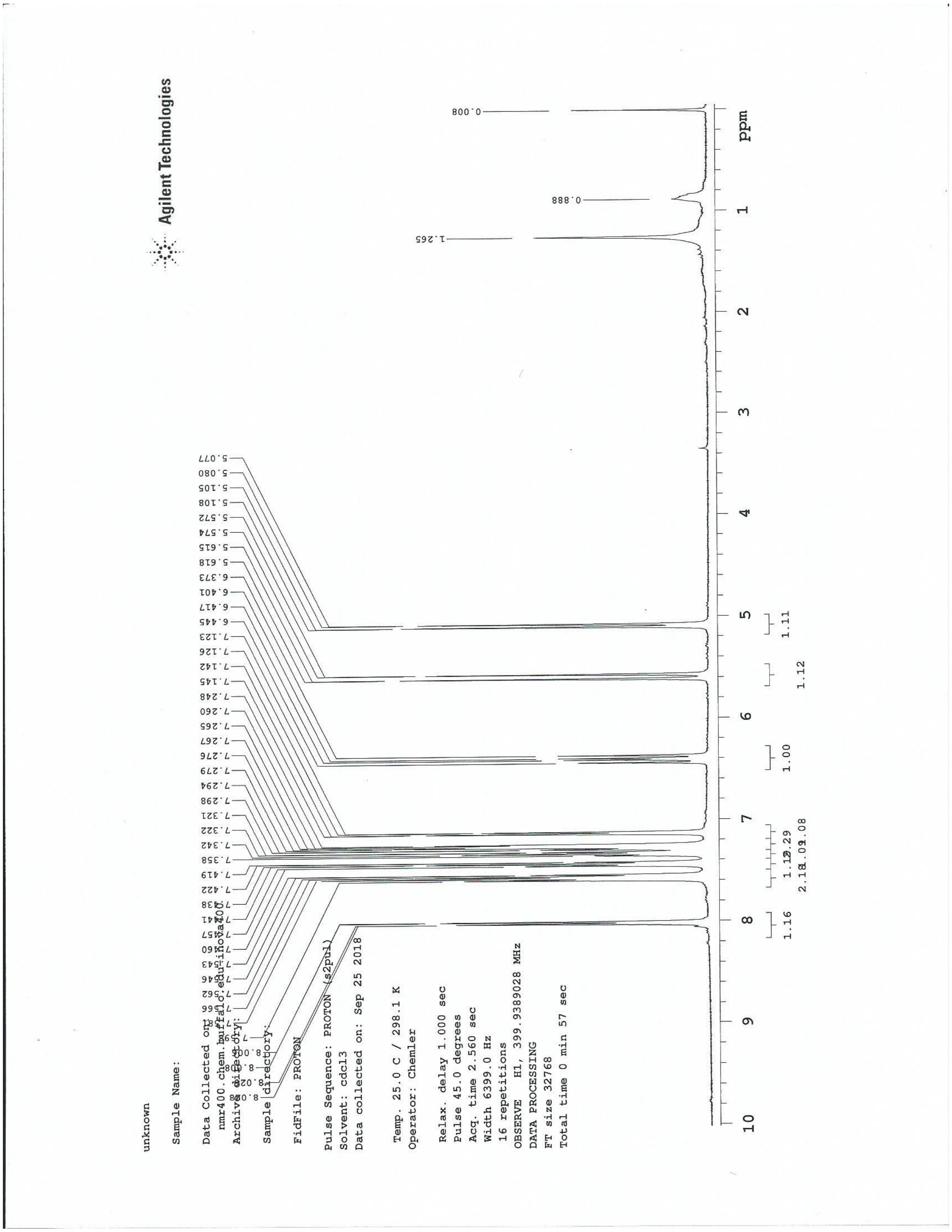

Agilent Technologies
unknown
Sample Name:
Data Collected on: nmr400.chem.buffalo.edu-inova400
Archive directory:
Sample directory:
FidFile: PROTON
Pulse Sequence: PROTON (s2pul)
Solvent: cdcl3
Data collected on: Sep 25 2018
Temp. 25.0 C / 298.1 K
Operator: Chemler
Relax. delay 1.000 sec
Pulse 45.0 degrees
Acq. time 2.560 sec
Width 6399.0 Hz
16 repetitions
OBSERVE H1, 399.9389028 MHz
DATA PROCESSING
FT size 32768
Total time 0 min 57 sec
7.566
7.562
7.546
7.543
7.460
7.457
7.441
7.438
7.422
7.419
7.358
7.342
7.322
7.321
7.298
7.294
7.279
7.276
7.267
7.265
7.260
7.248
7.145
7.142
7.126
7.123
6.445
6.417
6.401
6.373
5.618
5.615
5.574
5.572
5.108
5.105
5.080
5.077
1.265
0.888
0.008
1.16
1.00
1.12
1.11
ppm
10
9
8
7
6
5
4
3
2
1

JE_2_33_B

Sample Name:

Data Collected on:
nmr400.chem.buffalo.edu-inova400
Archive directory:

Sample directory:

FidFile: CARBON

Pulse Sequence: CARBON (s2pul)
Solvent: c6d6
Data collected on: Sep 25 2018

Temp. 25.0 C / 298.1 K
Operator: Chemler

Relax. delay 1.000 sec
Pulse 45.0 degrees
Acq. time 1.303 sec
Width 25141.4 Hz
1696 repetitions
OBSERVE C13, 100.5647183 MHz
DECOUPLE H1, 399.9409428 MHz
Power 33 dB
continuously on
WALTZ-16 modulated
DATA PROCESSING
Line broadening 0.5 Hz
FT size 65536
Total time 64 hr, 13 min

Agilent Technologies

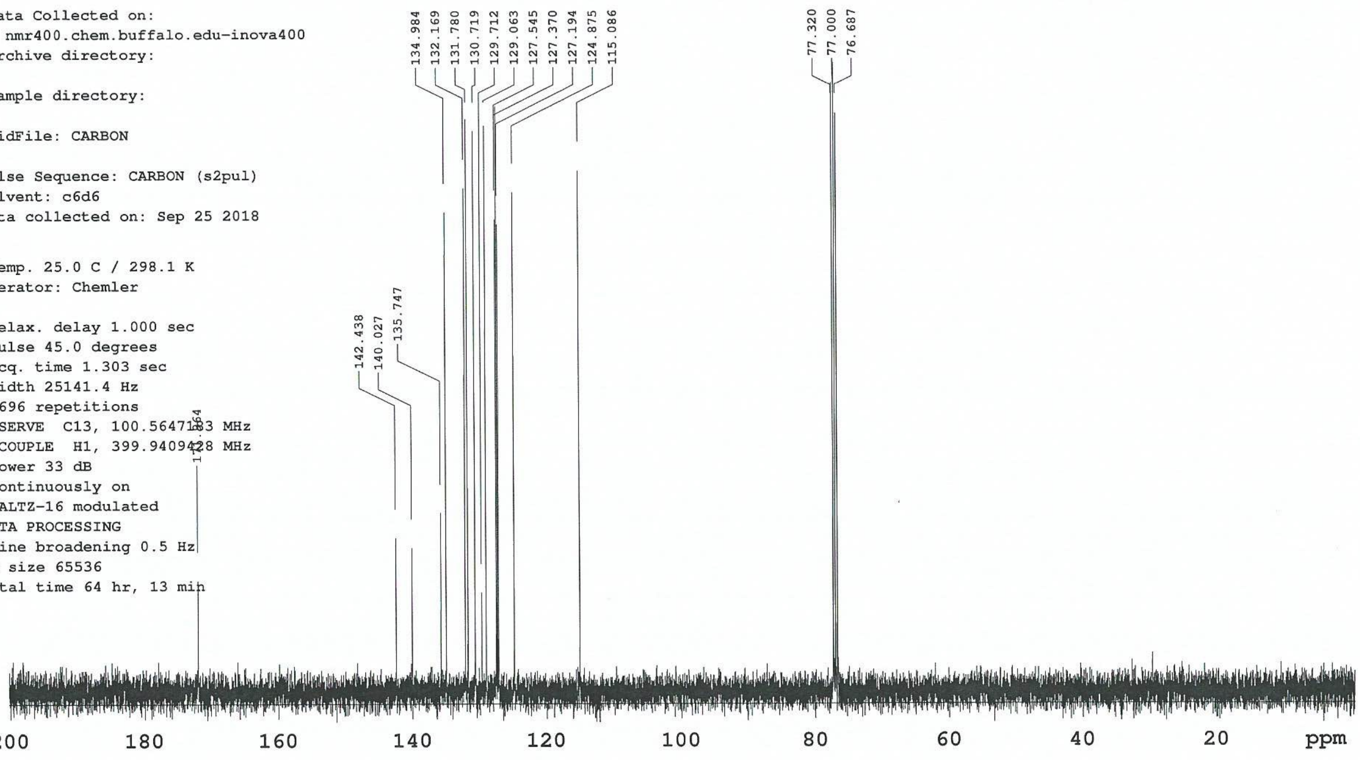

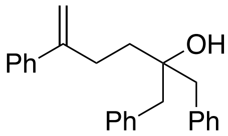
Ph
OH
Ph
Ph

***S*-8c**

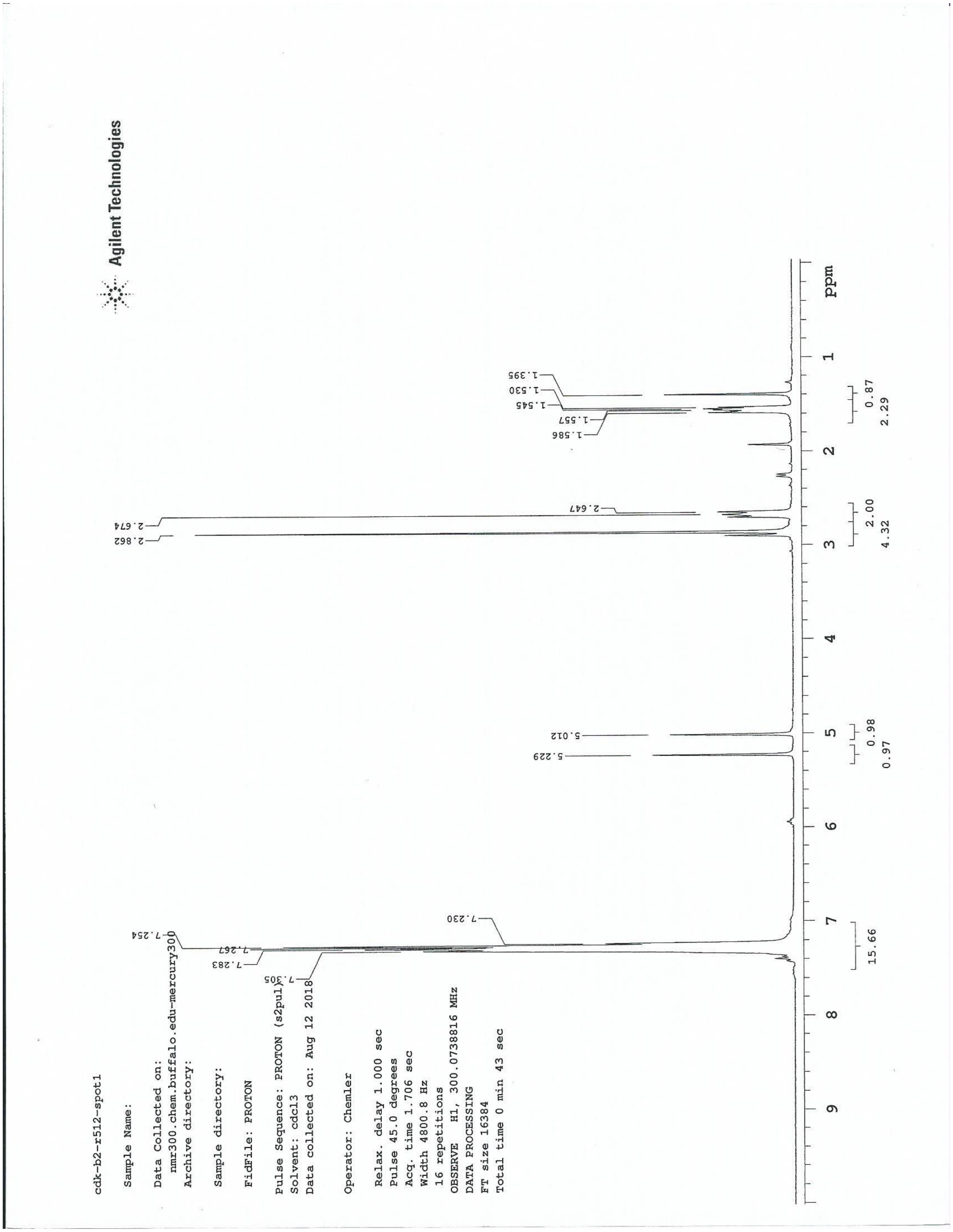
Agilent Technologies
cdk-b2-r512-spot1
Sample Name:
Data Collected on:
nmr300.chem.buffalo.edu-mercury300
Archive directory:
Sample directory:
FidFile: PROTON
Pulse Sequence: PROTON (s2pul)
Solvent: cdcl3
Data collected on: Aug 12 2018
Operator: Chemler
Relax. delay 1.000 sec
Pulse 45.0 degrees
Acq. time 1.706 sec
Width 4800.8 Hz
16 repetitions
OBSERVE H1, 300.0738816 MHz
DATA PROCESSING
FT size 16384
Total time 0 min 43 sec
7.305
7.283
7.267
7.254
7.230
5.229
5.012
2.862
2.674
2.647
1.586
1.557
1.545
1.530
1.395
15.66
0.97
0.98
4.32
2.00
2.29
0.87
9
8
7
6
5
4
3
2
1
ppm

cdk-b2-r512-spot1

Sample Name:

Data Collected on:
nmr300.chem.buffalo.edu-mercury300
Archive directory:

Sample directory:

FidFile: CARBON

Pulse Sequence: CARBON (s2pul)
Solvent: cdcl3
Data collected on: Aug 12 2018

Operator: Chemler

Relax. delay 2.000 sec
Pulse 45.0 degrees
Acq. time 0.868 sec
Width 18867.9 Hz
208 repetitions
OBSERVE C13, 75.4536389 MHz
DECOUPLE H1, 300.0754430 MHz
Power 37 dB
continuously on
WALTZ-16 modulated
DATA PROCESSING
Line broadening 0.5 Hz
FT size 32768
Total time 79 hr, 55 min

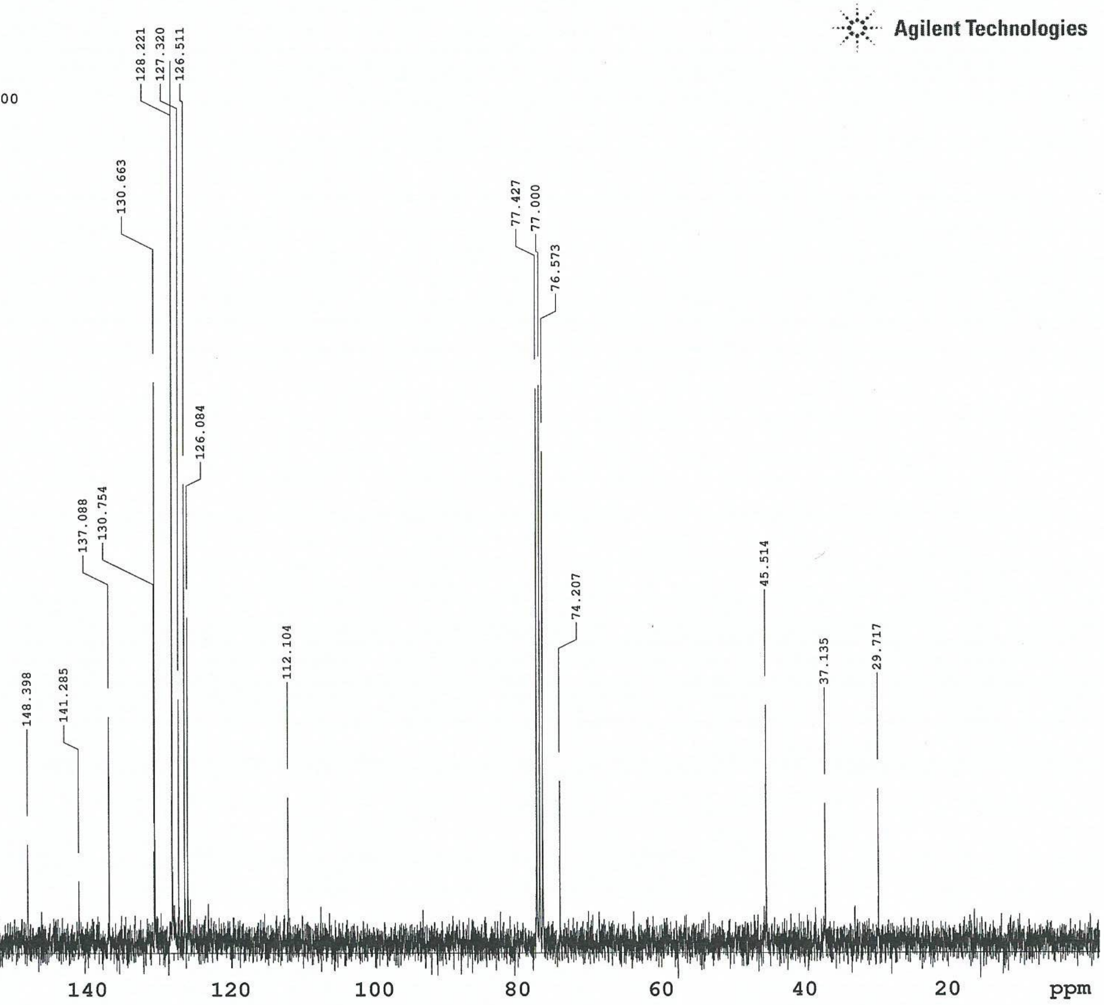

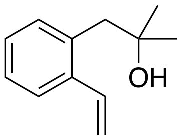

***S*-10b**

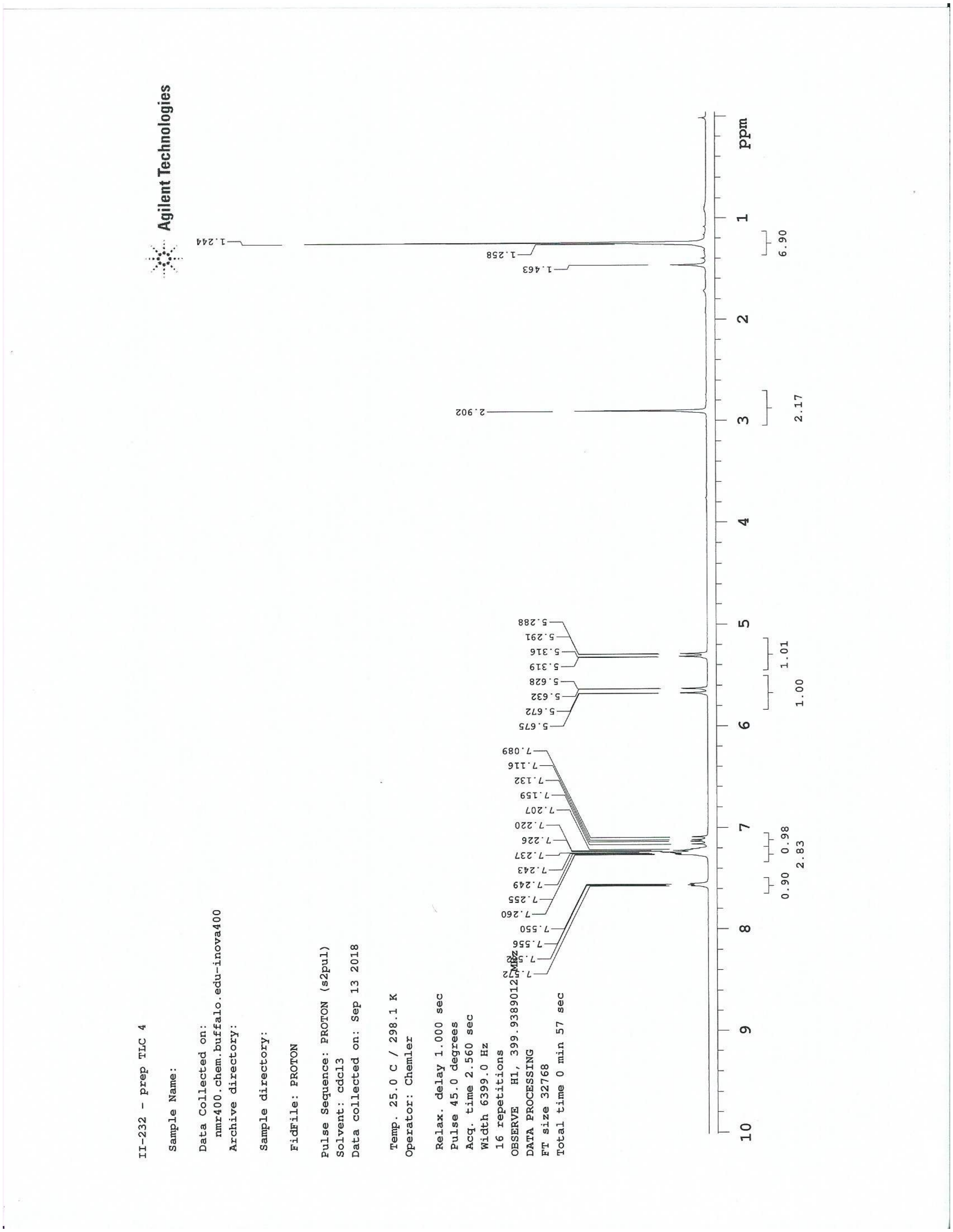
Agilent Technologies
II-232 - prep TLC 4
Sample Name:
Data Collected on:
nmr400.chem.buffalo.edu-inova400
Archive directory:
Sample directory:
FidFile: PROTON
Pulse Sequence: PROTON (s2pul)
Solvent: cdcl3
Data collected on: Sep 13 2018
Temp. 25.0 C / 298.1 K
Operator: Chemler
Relax. delay 1.000 sec
Pulse 45.0 degrees
Acq. time 2.560 sec
Width 6399.0 Hz
16 repetitions
OBSERVE H1, 399.9389012 MHz
DATA PROCESSING
FT size 32768
Total time 0 min 57 sec
1.244
1.258
1.463
2.902
5.288
5.291
5.316
5.319
5.628
5.632
5.672
5.675
7.089
7.116
7.132
7.159
7.207
7.220
7.226
7.237
7.243
7.249
7.255
7.260
7.550
7.556
7.572
6.90
2.17
1.01
1.00
0.98
2.83
0.90
ppm
1
2
3
4
5
6
7
8
9
10

II-232 - prep TLC 4

Sample Name:

Data Collected on:
nmr400.chem.buffalo.edu-inova400
Archive directory:

Sample directory:

FidFile: CARBON

Pulse Sequence: CARBON (s2pul)
Solvent: cdcl3
Data collected on: Sep 13 2018

Temp. 25.0 C / 298.1 K
Operator: Chemler

Relax. delay 1.000 sec
Pulse 45.0 degrees
Acq. time 1.303 sec
Width 25141.4 Hz
128 repetitions
OBSERVE C13, 100.5647223 MHz
DECOUPLE H1, 399.9409068 MHz
Power 33 dB
continuously on
WALTZ-16 modulated
DATA PROCESSING
Line broadening 0.5 Hz
FT size 65536
Total time 6422 hr, 50 min

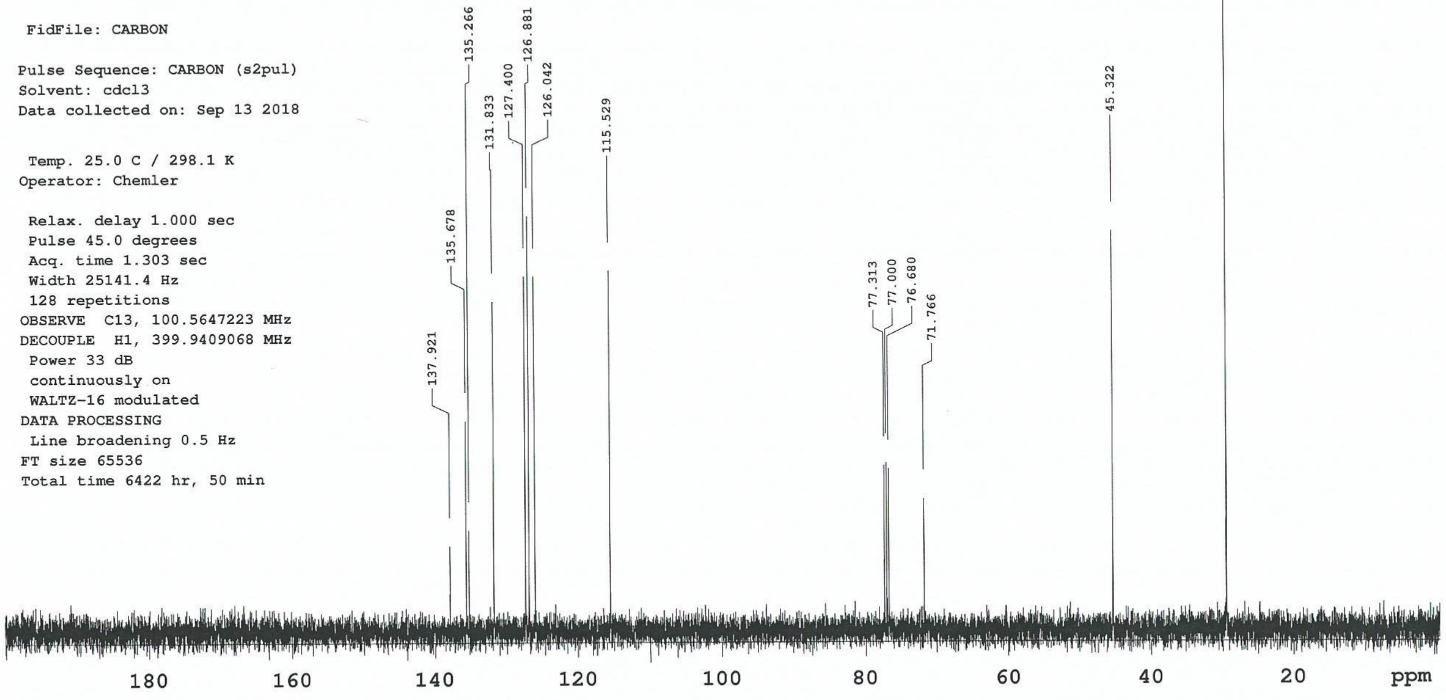

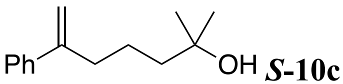
Ph
OH

***S*-10c**

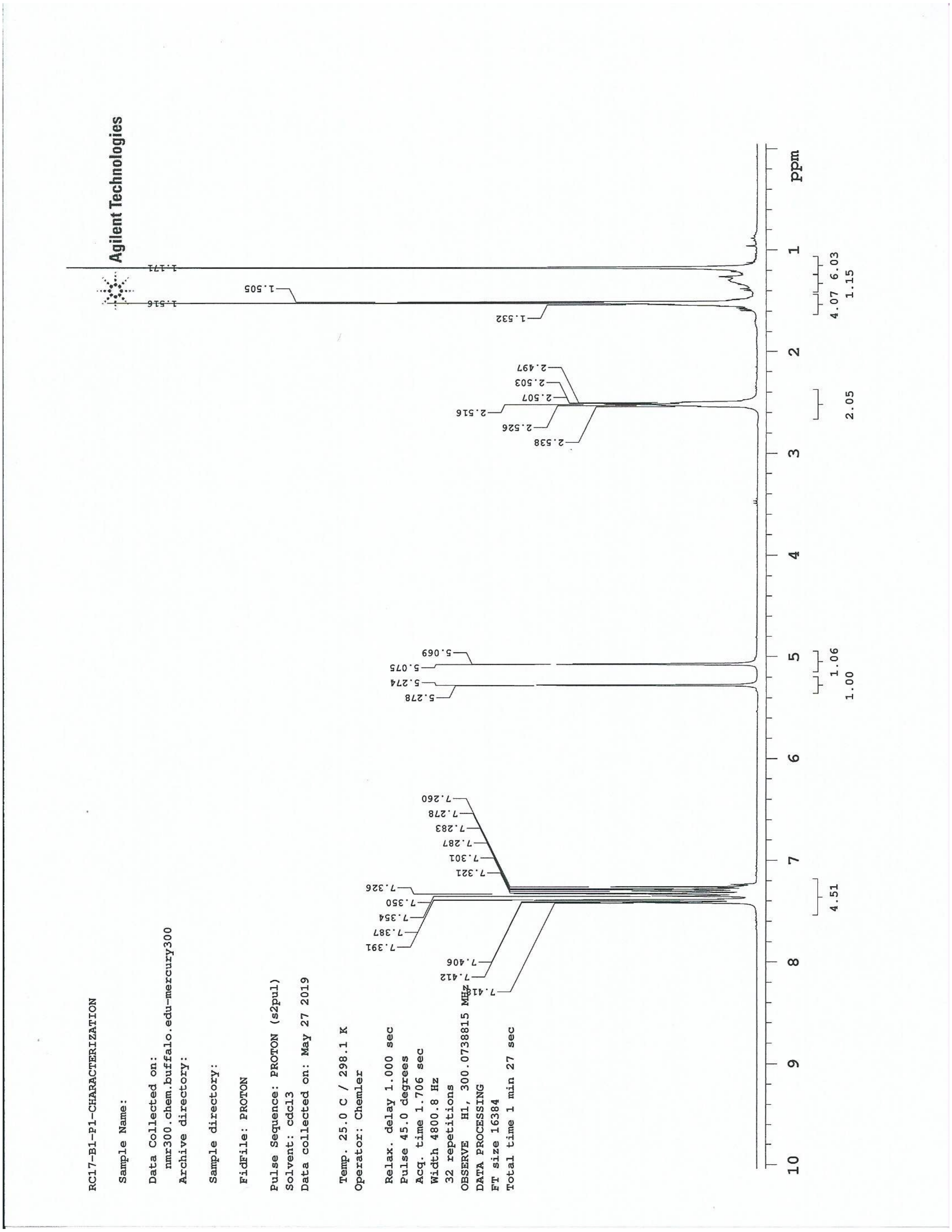
Agilent Technologies
RC17-B1-P1-CHARACTERIZATION
Sample Name:
Data Collected on:
nmr300.chem.buffalo.edu-mercury300
Archive directory:
Sample directory:
FidFile: PROTON
Pulse Sequence: PROTON (s2pul)
Solvent: cdcl3
Data collected on: May 27 2019
Temp. 25.0 C / 298.1 K
Operator: Chemler
Relax. delay 1.000 sec
Pulse 45.0 degrees
Acq. time 1.706 sec
Width 4800.8 Hz
32 repetitions
OBSERVE H1, 300.0738815 MHz
DATA PROCESSING
FT size 16384
Total time 1 min 27 sec
1.171
1.505
1.516
1.532
2.497
2.503
2.507
2.516
2.526
2.538
5.069
5.075
5.274
5.278
7.260
7.278
7.283
7.287
7.301
7.321
7.326
7.350
7.354
7.387
7.391
7.406
7.412
7.418
4.07
6.03
1.15
2.05
1.06
1.00
4.51
ppm

RC17-B1-P1-CHARACTERIZATION

Sample Name:

Data Collected on:
nmr300.chem.buffalo.edu-mercury300
Archive directory:

Sample directory:

FidFile: CARBON

Pulse Sequence: CARBON (s2pul)
Solvent: cdcl3
Data collected on: May 27 2019

Temp. 25.0 C / 298.1 K
Operator: Chemler

Relax. delay 1.000 sec
Pulse 45.0 degrees
Acq. time 0.868 sec
Width 18867.9 Hz
504 repetitions
OBSERVE C13, 75.4536389 MHz
DECOUPLE H1, 300.0754430 MHz
Power 37 dB
continuously on
WALTZ-16 modulated
DATA PROCESSING
Line broadening 0.5 Hz
FT size 32768
Total time 52 hr, 8 min

Agilent Technologies

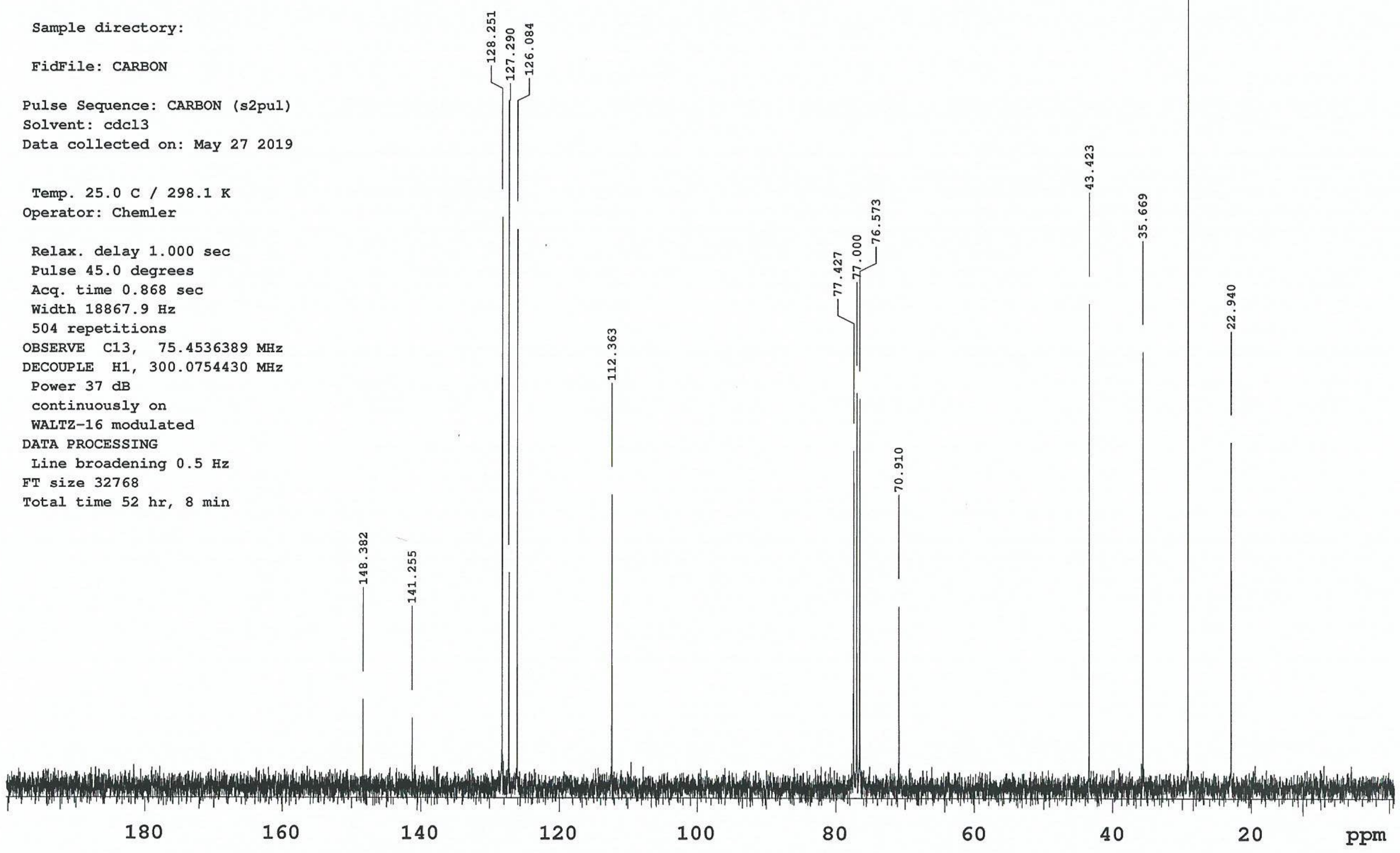

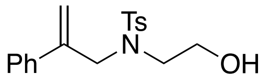
Ts
N
Ph
OH

***S*-10d**

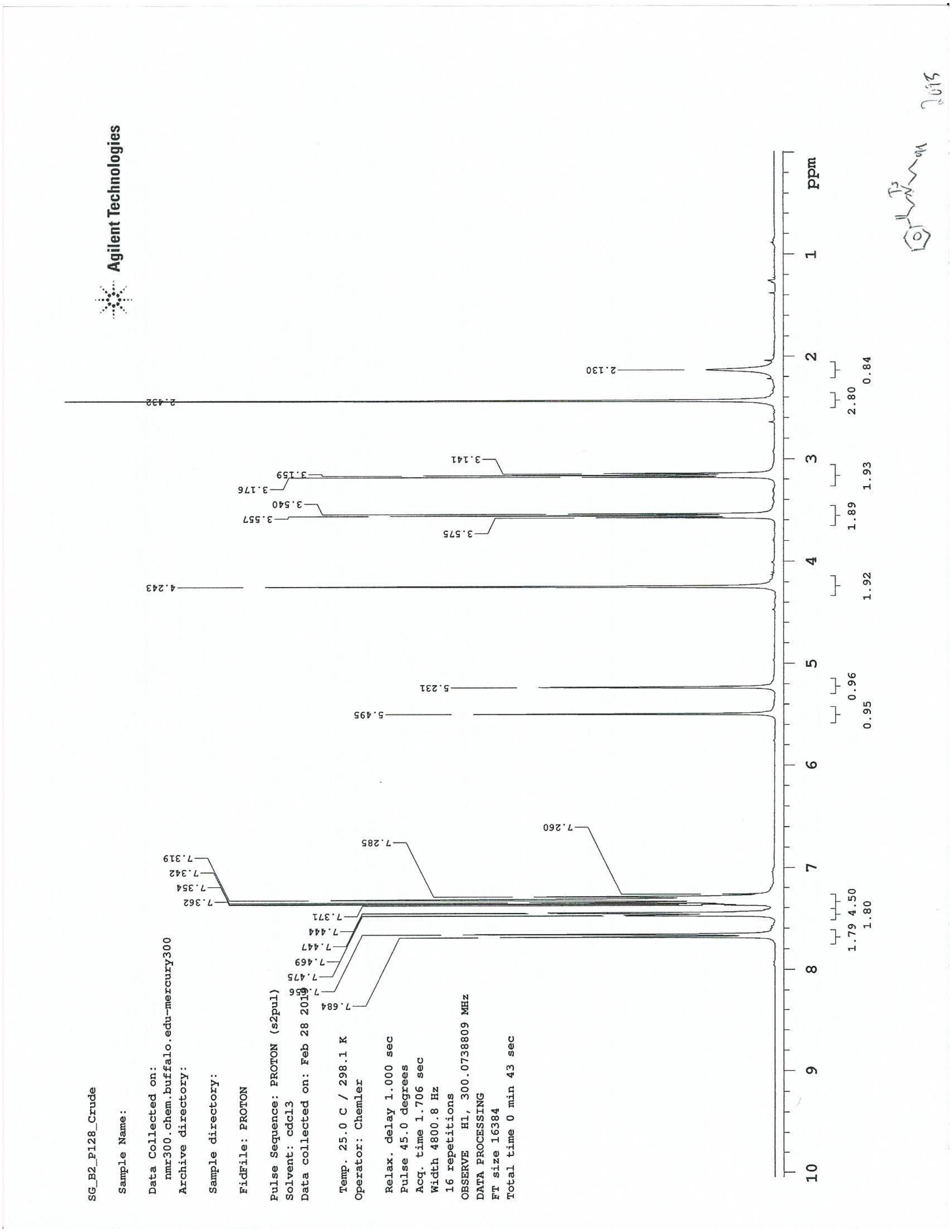
Agilent Technologies
SG_B2_P128_Crude
Sample Name:
Data Collected on:
nmr300.chem.buffalo.edu-mercury300
Archive directory:
Sample directory:
FidFile: PROTON
Pulse Sequence: PROTON (s2pul)
Solvent: cdcl3
Data collected on: Feb 28 2019
Temp. 25.0 C / 298.1 K
Operator: Chemler
Relax. delay 1.000 sec
Pulse 45.0 degrees
Acq. time 1.706 sec
Width 4800.8 Hz
16 repetitions
OBSERVE H1, 300.0738809 MHz
DATA PROCESSING
FT size 16384
Total time 0 min 43 sec
7.684
7.656
7.475
7.469
7.447
7.444
7.371
7.362
7.354
7.342
7.319
7.285
7.260
5.495
5.231
4.243
3.575
3.557
3.540
3.176
3.159
3.141
2.432
2.130
1.79
1.80
4.50
0.95
0.96
1.92
1.89
1.93
2.80
0.84
ppm
10
9
8
7
6
5
4
3
2
1

SG_B2_P128_Crude

Sample Name:

Data Collected on:
nmr300.chem.buffalo.edu-mercury300
Archive directory:

Sample directory:

FidFile: CARBON

Pulse Sequence: CARBON (s2pul)
Solvent: cdcl3
Data collected on: Feb 28 2019

Temp. 25.0 C / 298.1 K
Operator: Chemler

Relax. delay 1.000 sec
Pulse 45.0 degrees
Acq. time 0.868 sec
Width 18867.9 Hz
144 repetitions
OBSERVE C13, 75.4536435 MHz
DECOUPLE H1, 300.0754430 MHz
Power 37 dB
continuously on
WALTZ-16 modulated
DATA PROCESSING
Line broadening 0.5 Hz
FT size 32768
Total time 521 hr, 26 min

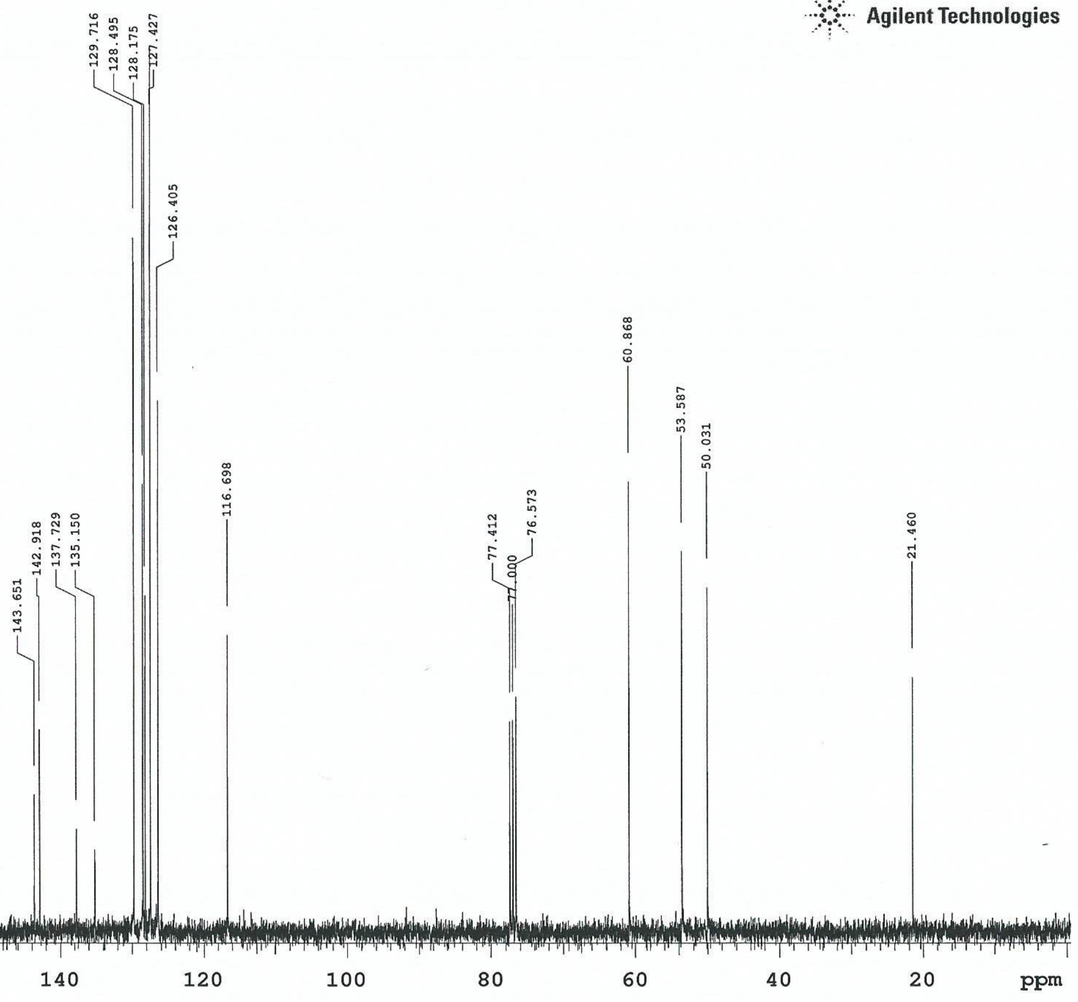

2045

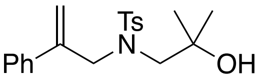

Ph
Ts
N
OH

***S*-10e**

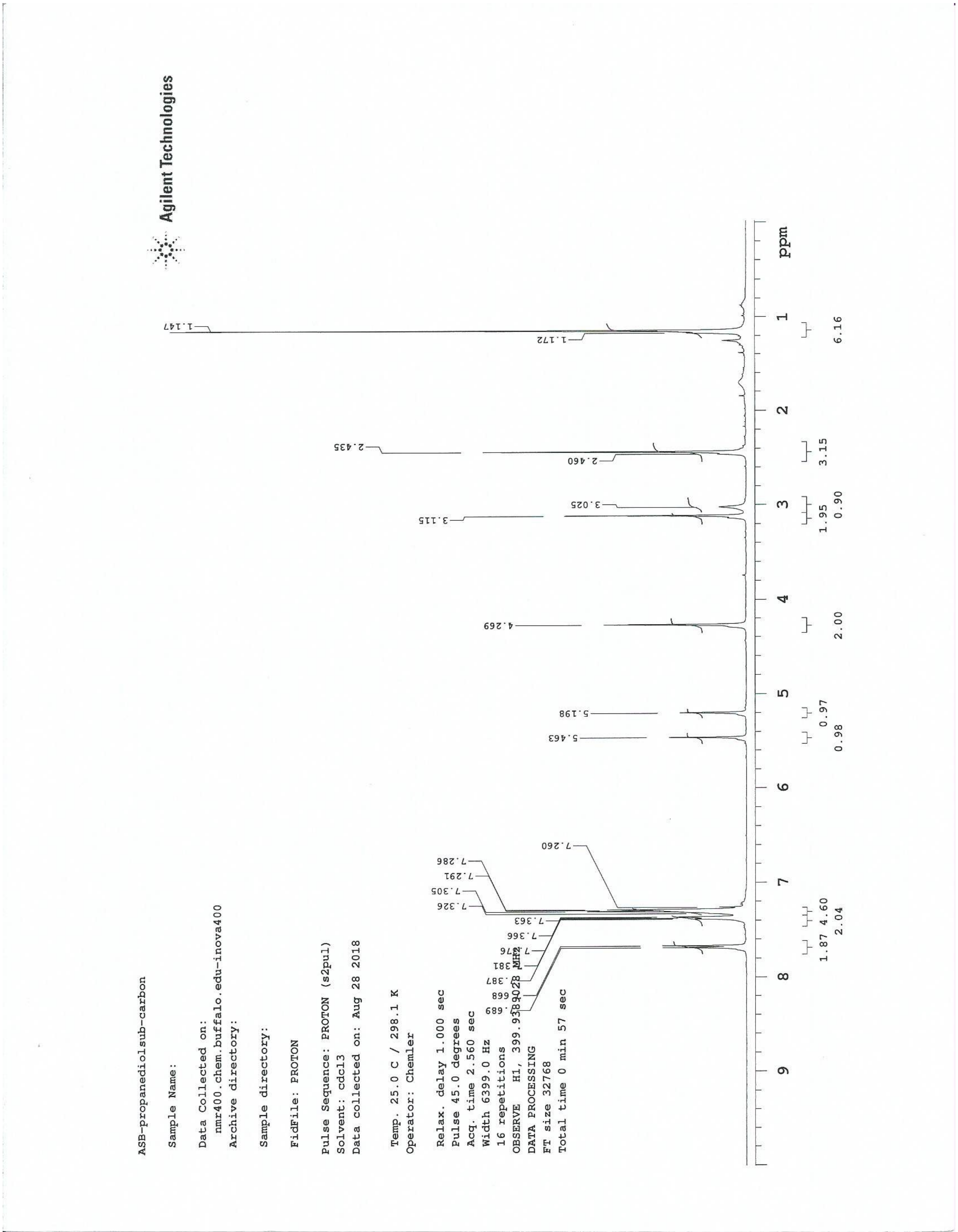

Agilent Technologies
ASB-propanediolsub-carbon
Sample Name:
Data Collected on:
nmr400.chem.buffalo.edu-inova400
Archive directory:
Sample directory:
FidFile: PROTON
Pulse Sequence: PROTON (s2pul)
Solvent: cdcl3
Data collected on: Aug 28 2018
Temp. 25.0 C / 298.1 K
Operator: Chemler
Relax. delay 1.000 sec
Pulse 45.0 degrees
Acq. time 2.560 sec
Width 6399.0 Hz
16 repetitions
OBSERVE H1, 399.9389028 MHz
DATA PROCESSING
FT size 32768
Total time 0 min 57 sec
1.147
1.172
2.435
2.460
3.025
3.115
4.269
5.198
5.463
7.260
7.286
7.291
7.305
7.326
7.363
7.366
7.376
7.381
7.387
7.668
7.689
6.16
3.15
1.95
0.90
2.00
0.97
0.98
1.87
4.60
2.04
ppm

ASB-propanediolsub-carbon

Sample Name:

Data Collected on:
nmr400.chem.buffalo.edu-inova400
Archive directory:

Sample directory:

FidFile: CARBON

Pulse Sequence: CARBON (s2pul)
Solvent: cdcl3
Data collected on: Aug 28 2018

Temp. 25.0 C / 298.1 K
Operator: Chemler

Relax. delay 1.000 sec
Pulse 45.0 degrees
Acq. time 1.303 sec
Width 25141.4 Hz
240 repetitions
OBSERVE C13, 100.5647207 MHz
DECOUPLE H1, 399.9409068 MHz
Power 33 dB
continuously on
WALTZ-16 modulated
DATA PROCESSING
Line broadening 0.5 Hz
FT size 65536
Total time 64 hr, 13 min

Agilent Technologies

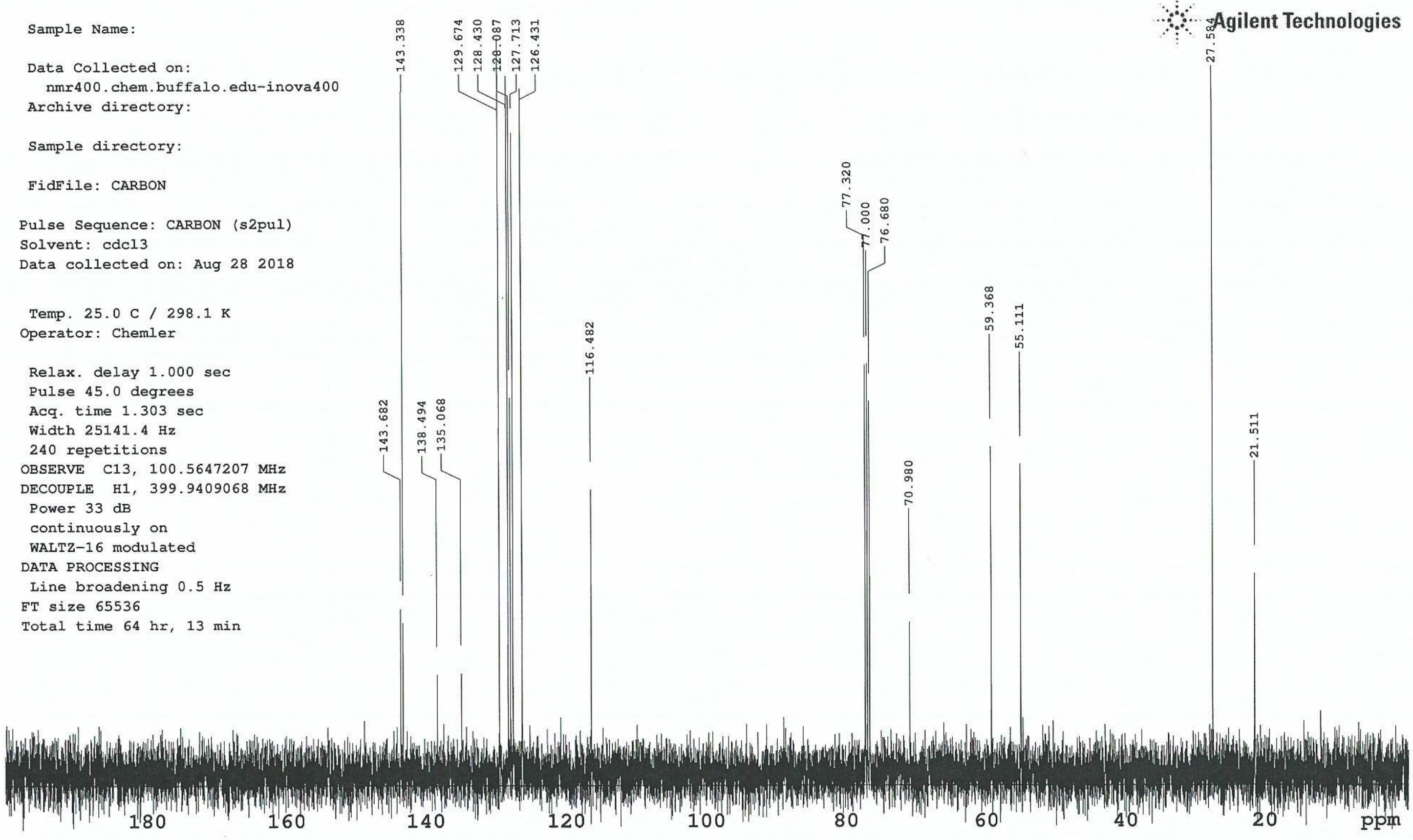

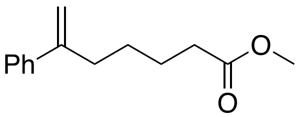
Ph
O
O

***S*-2**

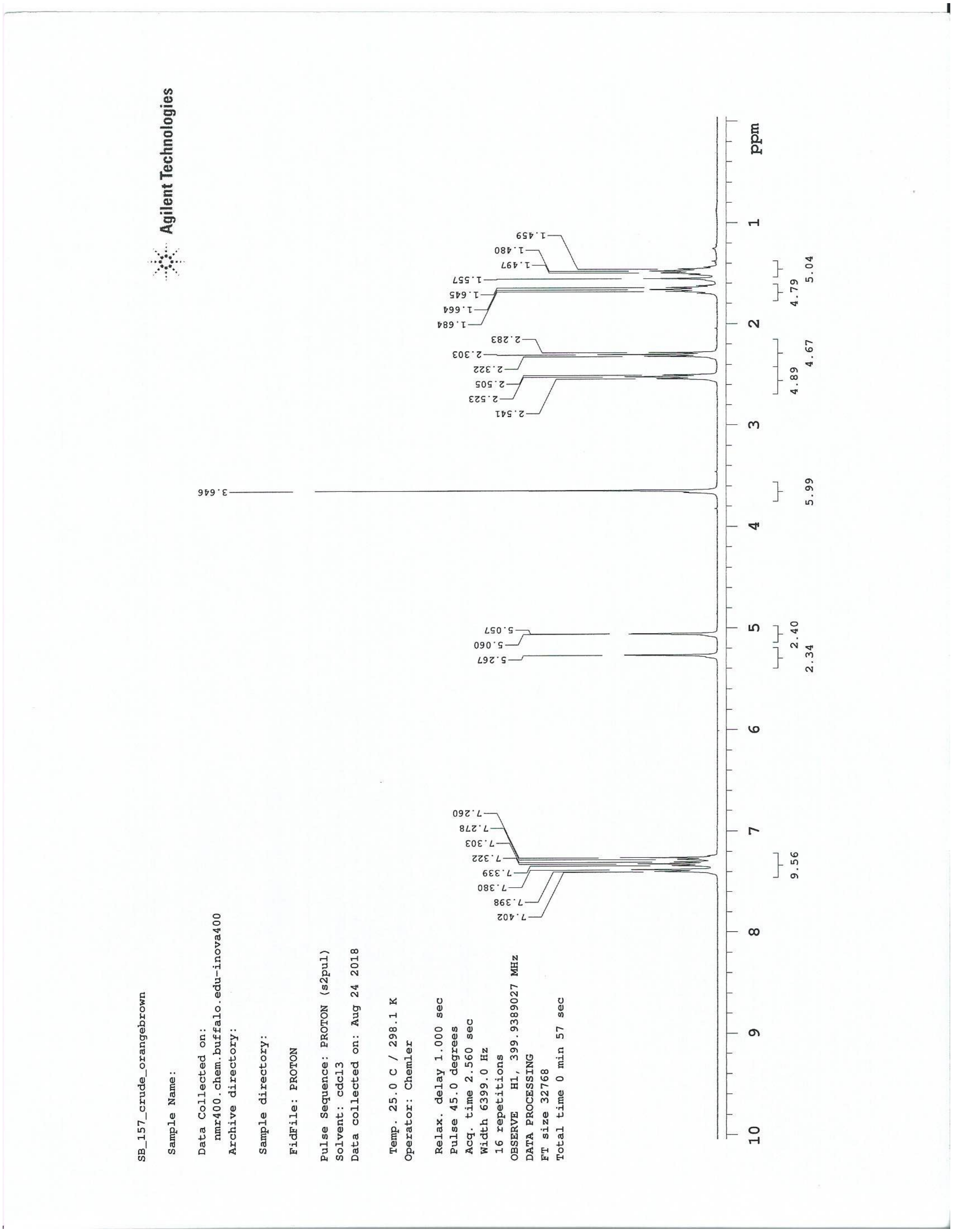
Agilent Technologies
SB_157_crude_orangebrown
Sample Name:
Data Collected on:
nmr400.chem.buffalo.edu-inova400
Archive directory:
Sample directory:
FidFile: PROTON
Pulse Sequence: PROTON (s2pul)
Solvent: cdcl3
Data collected on: Aug 24 2018
Temp. 25.0 C / 298.1 K
Operator: Chemler
Relax. delay 1.000 sec
Pulse 45.0 degrees
Acq. time 2.560 sec
Width 6399.0 Hz
16 repetitions
OBSERVE H1, 399.9389027 MHz
DATA PROCESSING
FT size 32768
Total time 0 min 57 sec
7.402
7.398
7.380
7.339
7.322
7.303
7.278
7.260
5.267
5.060
5.057
3.646
2.541
2.523
2.505
2.322
2.303
2.283
1.684
1.664
1.645
1.557
1.497
1.480
1.459
9.56
2.34
2.40
5.99
4.89
4.67
4.79
5.04
10
9
8
7
6
5
4
3
2
1
ppm

SB_157_crude_orangebrown

Sample Name:

Data Collected on:
nmr400.chem.buffalo.edu-inova400
Archive directory:

Sample directory:

FidFile: CARBON

Pulse Sequence: CARBON (s2pul)
Solvent: cdcl3
Data collected on: Aug 24 2018

Temp. 25.0 C / 298.1 K
Operator: Chemler

Relax. delay 1.000 sec
Pulse 45.0 degrees
Acq. time 1.303 sec
Width 25141.4 Hz
1576 repetitions
OBSERVE C13, 100.5647177 MHz
DECOUPLE H1, 399.9409068 MHz
Power 33 dB
continuously on
WALTZ-16 modulated
DATA PROCESSING
Line broadening 0.5 Hz
FT size 65536
Total time 642 hr, 17 min

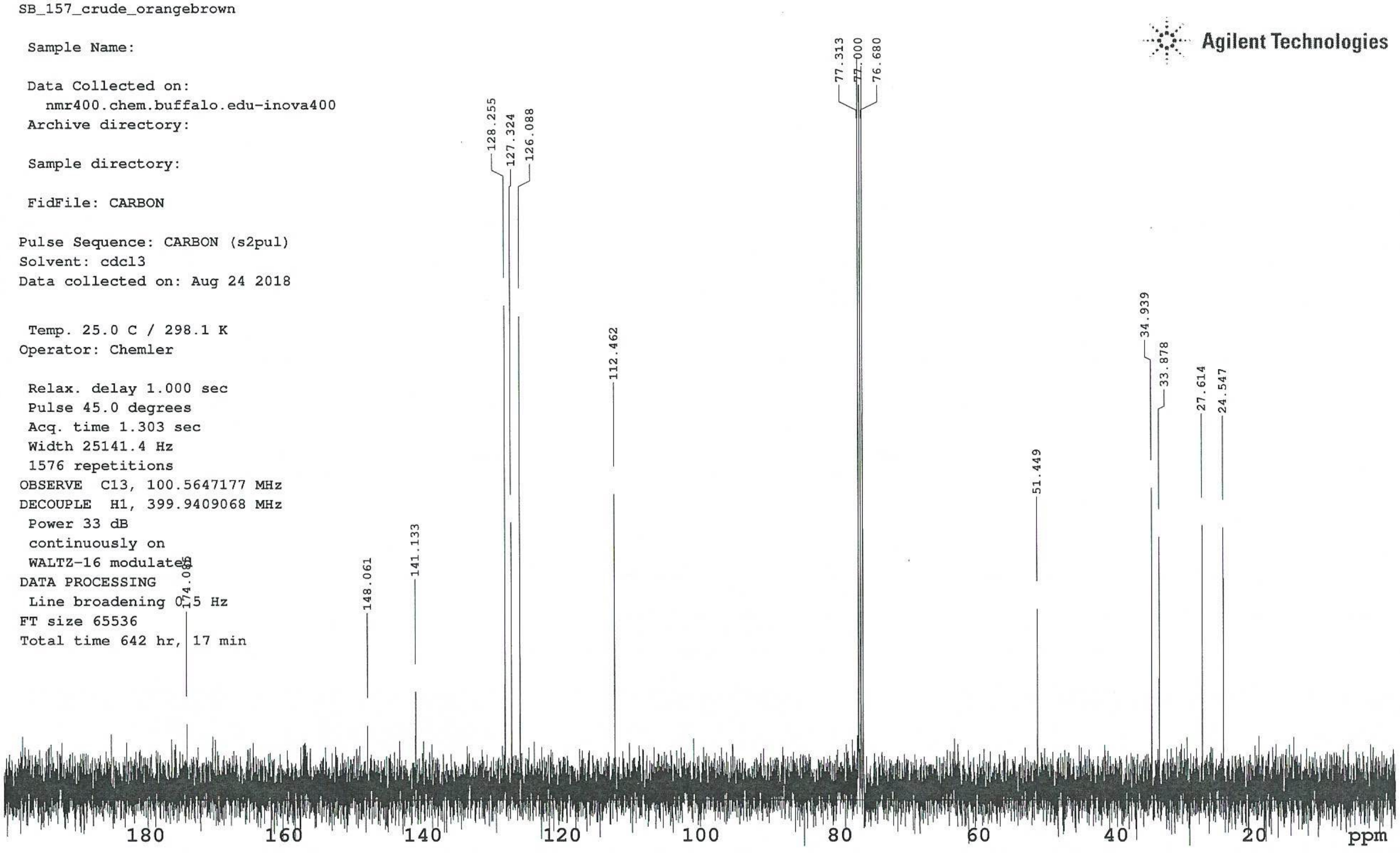

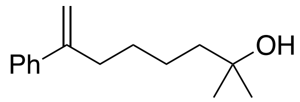
Ph
OH

***S*-11a**

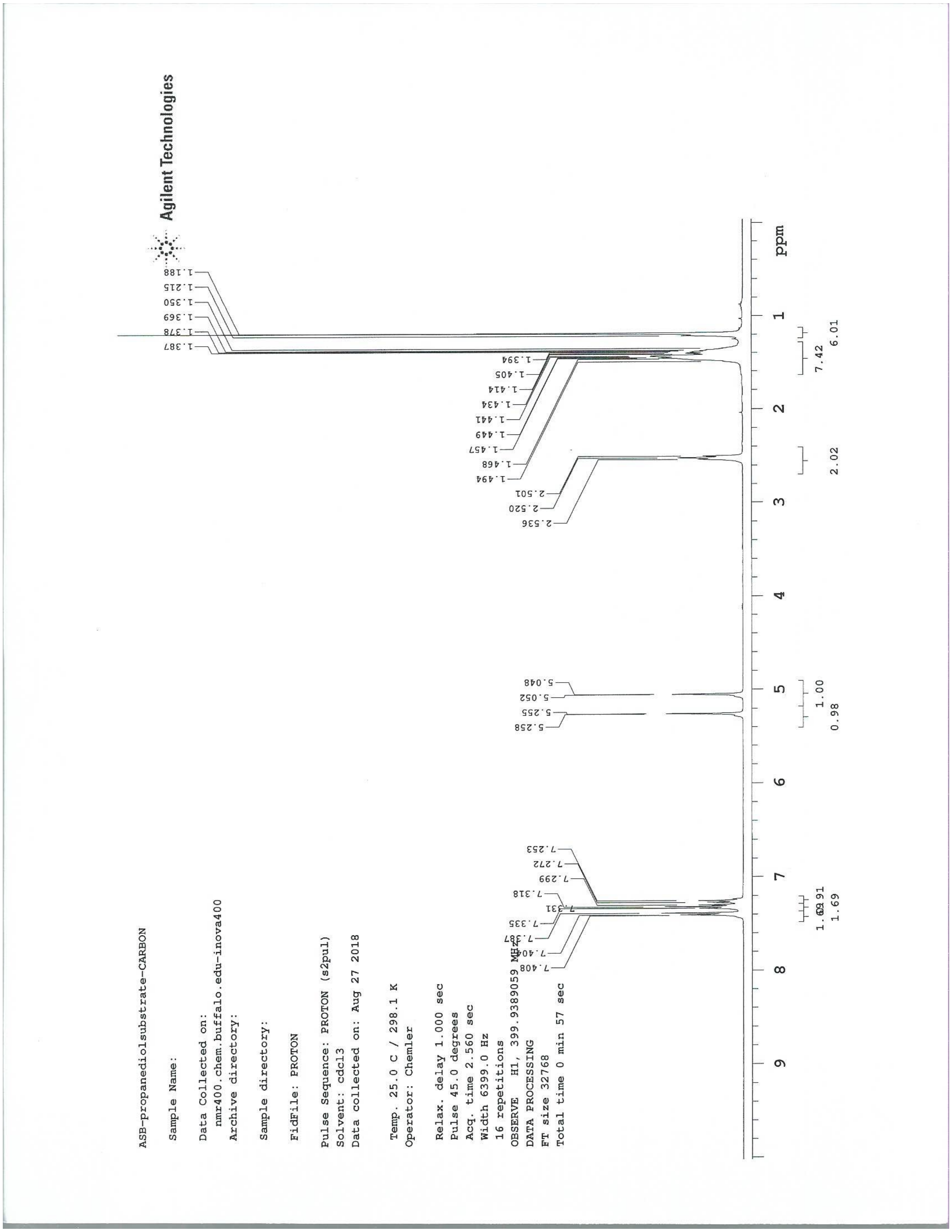
Agilent Technologies
ASB-propanediolsubstrate-CARBON
Sample Name:
Data Collected on:
nmr400.chem.buffalo.edu-inova400
Archive directory:
Sample directory:
FidFile: PROTON
Pulse Sequence: PROTON (s2pul)
Solvent: cdcl3
Data collected on: Aug 27 2018
Temp. 25.0 C / 298.1 K
Operator: Chemler
Relax. delay 1.000 sec
Pulse 45.0 degrees
Acq. time 2.560 sec
Width 6399.0 Hz
16 repetitions
OBSERVE H1, 399.9389059 MHz
DATA PROCESSING
FT size 32768
Total time 0 min 57 sec
ppm
7.42
6.01
2.02
1.00
0.98
1.69

ASB-propanediolsubstrate-CARBON

Sample Name:

Data Collected on:
nmr400.chem.buffalo.edu-inova400
Archive directory:

Sample directory:

FidFile: CARBON

Pulse Sequence: CARBON (s2pul)
Solvent: cdcl3
Data collected on: Aug 27 2018

Temp. 25.0 C / 298.1 K
Operator: Chemler

Relax. delay 1.000 sec
Pulse 45.0 degrees
Acq. time 1.303 sec
Width 25141.4 Hz
500 repetitions
OBSERVE C13, 100.5647192 MHz
DECOUPLE H1, 399.9409068 MHz
Power 33 dB
continuously on
WALTZ-16 modulated
DATA PROCESSING
Line broadening 0.5 Hz
FT size 65536
Total time 642 hr, 17 min

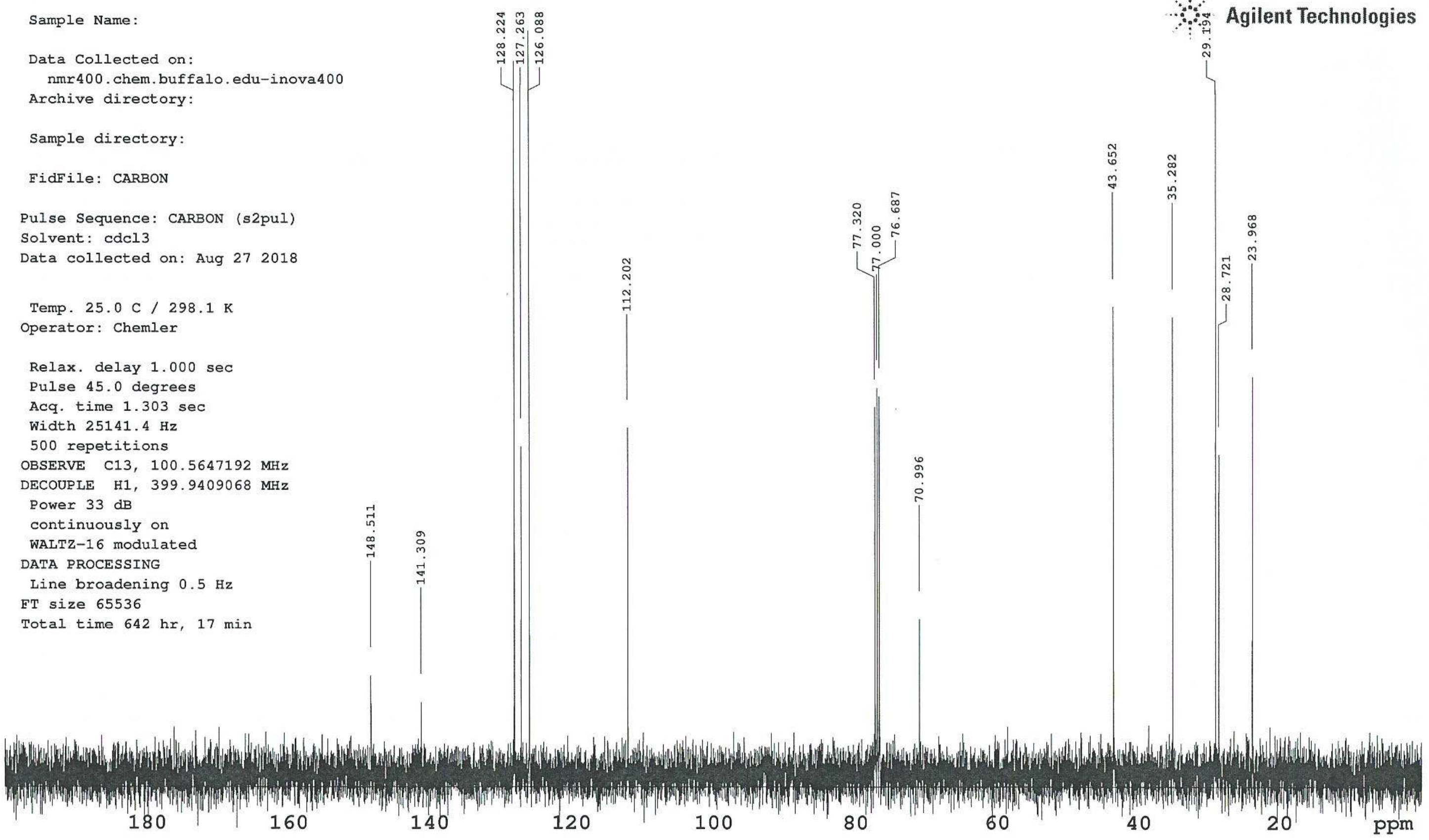

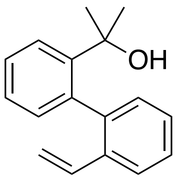
OH

***S*-11b**

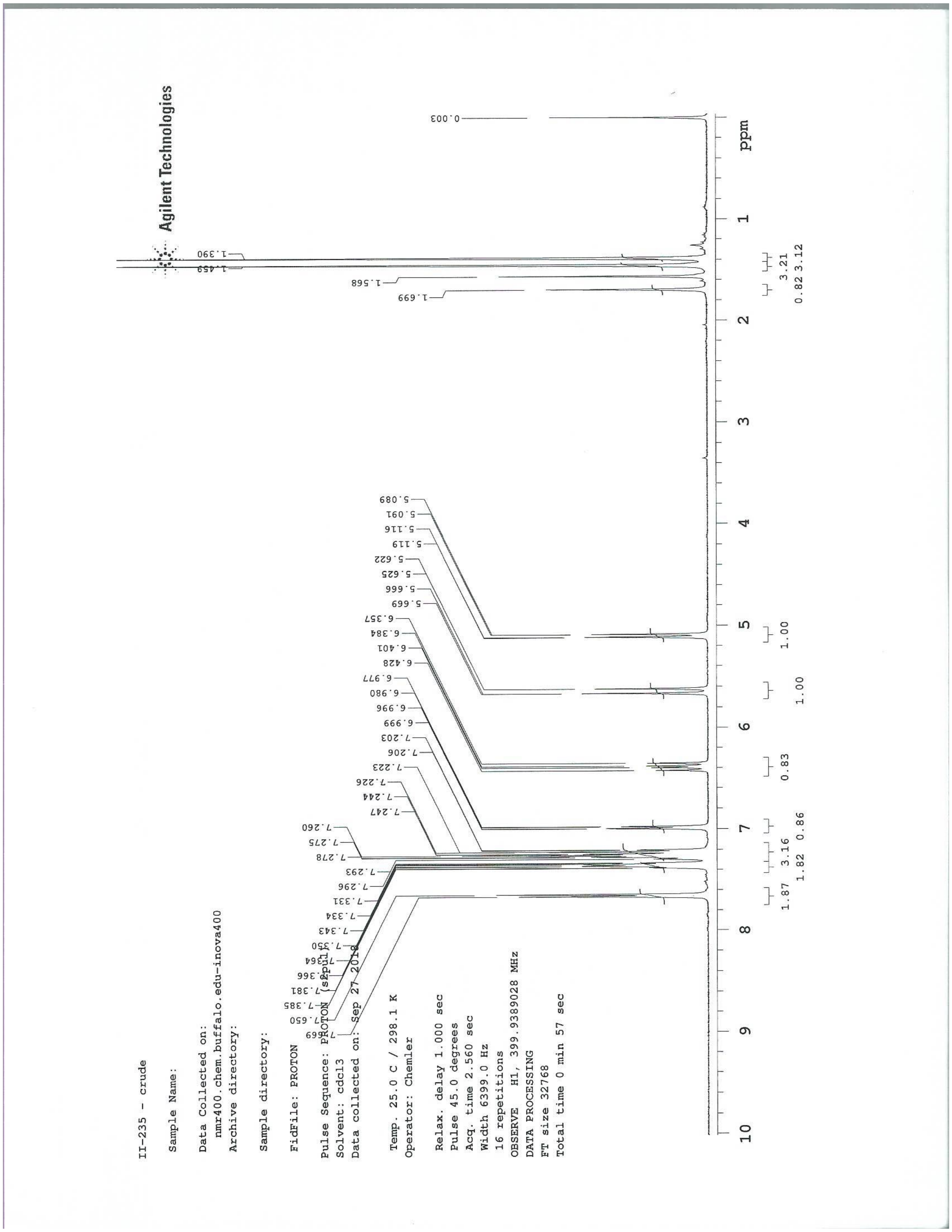
Agilent Technologies
II-235 - crude
Sample Name:
Data Collected on:
nmr400.chem.buffalo.edu-inova400
Archive directory:
Sample directory:
FidFile: PROTON
Pulse Sequence: PROTON (s2pul)
Solvent: cdcl3
Data collected on: Sep 27 2018
Temp. 25.0 C / 298.1 K
Operator: Chemler
Relax. delay 1.000 sec
Pulse 45.0 degrees
Acq. time 2.560 sec
Width 6399.0 Hz
16 repetitions
OBSERVE H1, 399.9389028 MHz
DATA PROCESSING
FT size 32768
Total time 0 min 57 sec
ppm

II-235 - crude

Sample Name:

Data Collected on:
nmr400.chem.buffalo.edu-inova400
Archive directory:

Sample directory:

FidFile: CARBON

Pulse Sequence: CARBON (s2pul)
Solvent: cdcl3
Data collected on: Sep 27 2018

Temp. 25.0 C / 298.1 K
Operator: Chemler

Relax. delay 1.000 sec
Pulse 45.0 degrees
Acq. time 1.303 sec
Width 25141.4 Hz
1792 repetitions
OBSERVE C13, 100.5647177 MHz
DECOUPLE H1, 399.9409068 MHz
Power 33 dB
continuously on
WALTZ-16 modulated
DATA PROCESSING
Line broadening 0.5 Hz
FT size 65536
Total time 642 hr, 17 min

Agilent Technologies

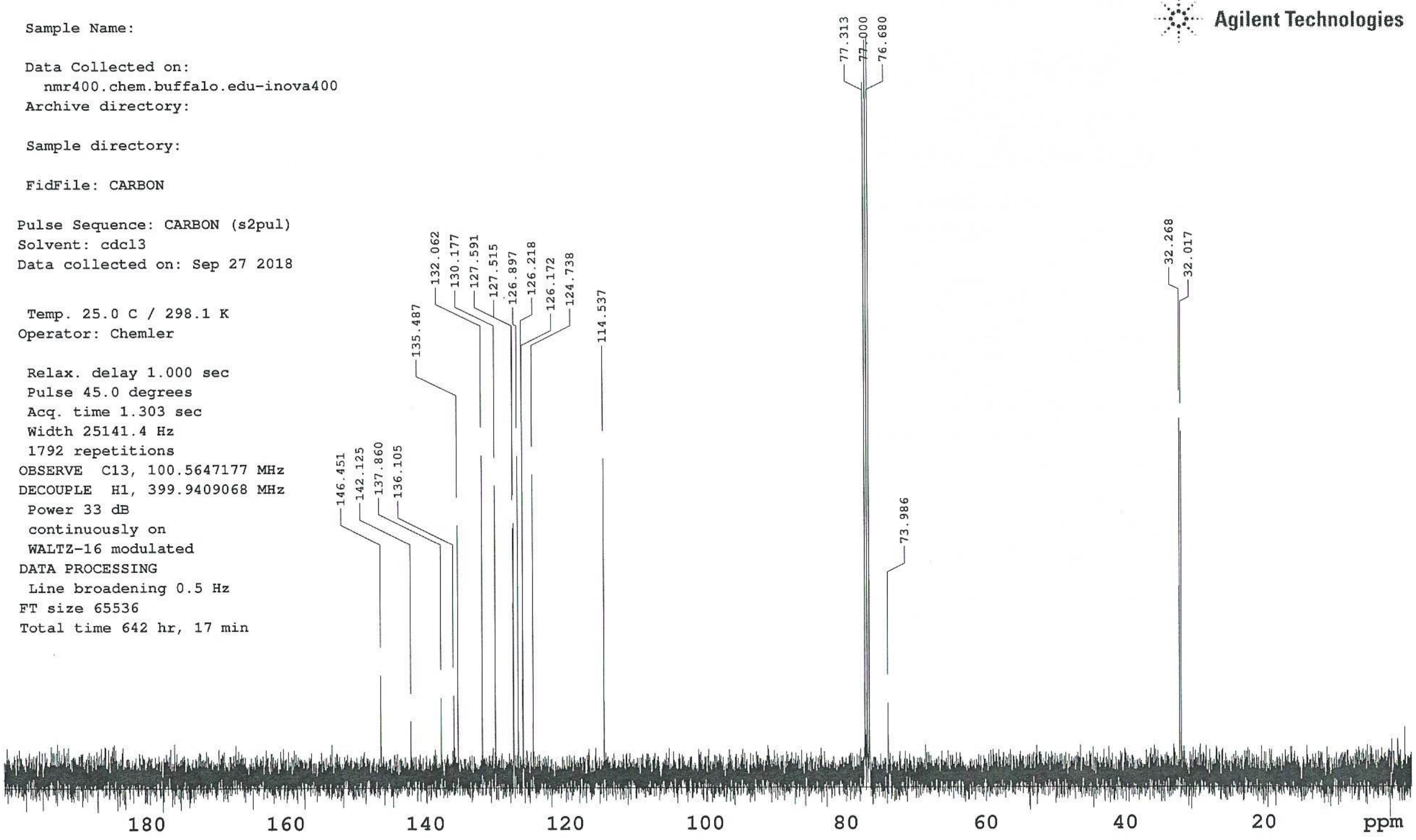

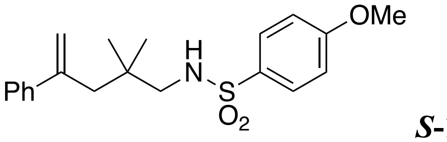

OMe
H
N
S
O2
Ph

***S*-12a**

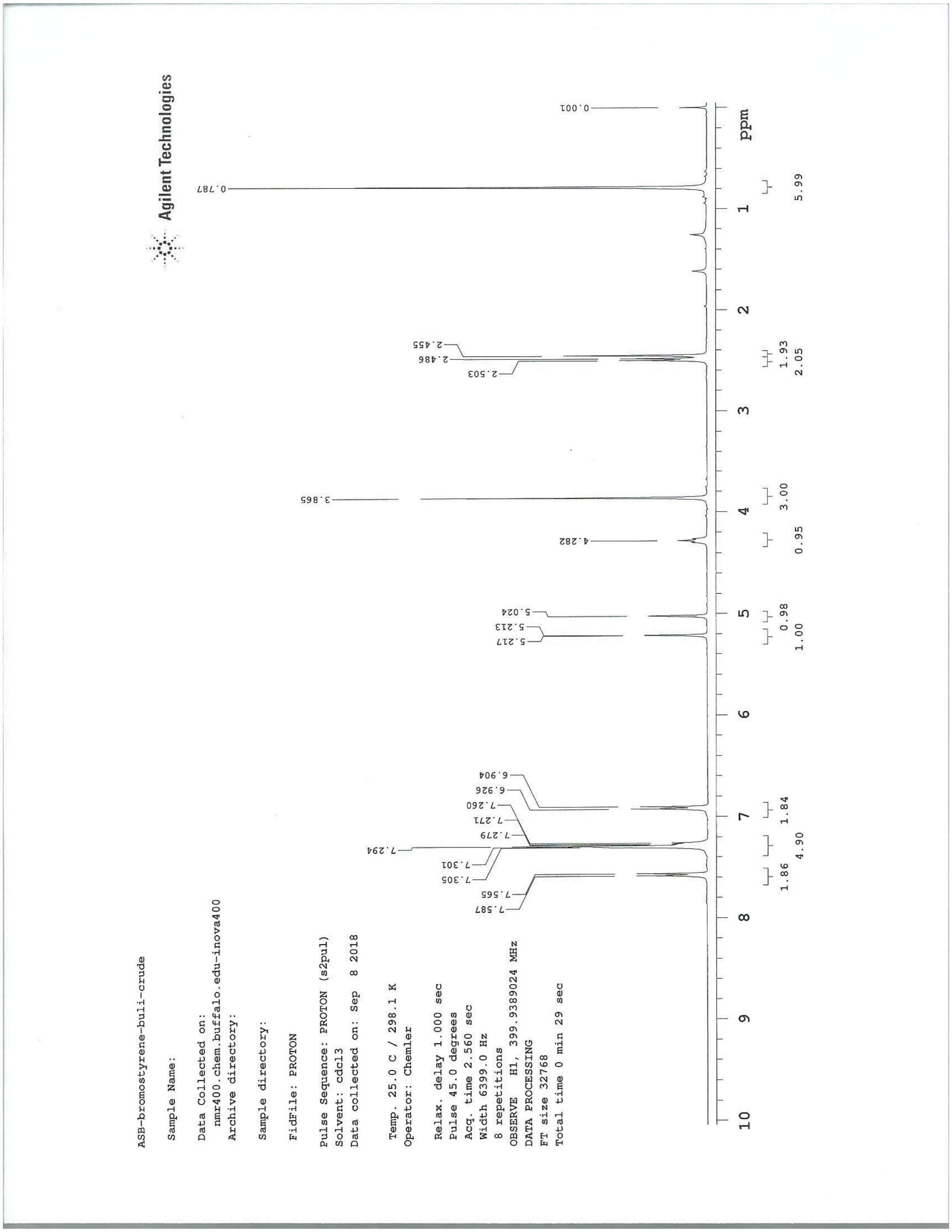

Agilent Technologies
ASB-bromostyrene-buli-crude
Sample Name:
Data Collected on:
nmr400.chem.buffalo.edu-inova400
Archive directory:
Sample directory:
FidFile: PROTON
Pulse Sequence: PROTON (s2pul)
Solvent: cdcl3
Data collected on: Sep 8 2018
Temp. 25.0 C / 298.1 K
Operator: Chemler
Relax. delay 1.000 sec
Pulse 45.0 degrees
Acq. time 2.560 sec
Width 6399.0 Hz
8 repetitions
OBSERVE H1, 399.9389024 MHz
DATA PROCESSING
FT size 32768
Total time 0 min 29 sec
ppm

ASB-bromostyrene-buli-crude

Sample Name:

Data Collected on:
nmr400.chem.buffalo.edu-inova400
Archive directory:

Sample directory:

FidFile: CARBON

Pulse Sequence: CARBON (s2pul)
Solvent: cdcl3
Data collected on: Sep 8 2018

Temp. 25.0 C / 298.1 K
Operator: Chemler

Relax. delay 1.000 sec
Pulse 45.0 degrees
Acq. time 1.303 sec
Width 25141.4 Hz
360 repetitions
OBSERVE C13, 100.5647200 MHz
DECOUPLE H1, 399.9409068 MHz
Power 33 dB
continuously on
WALTZ-16 modulated
DATA PROCESSING
Line broadening 0.5 Hz
FT size 65536
Total time 642 hr, 17 min

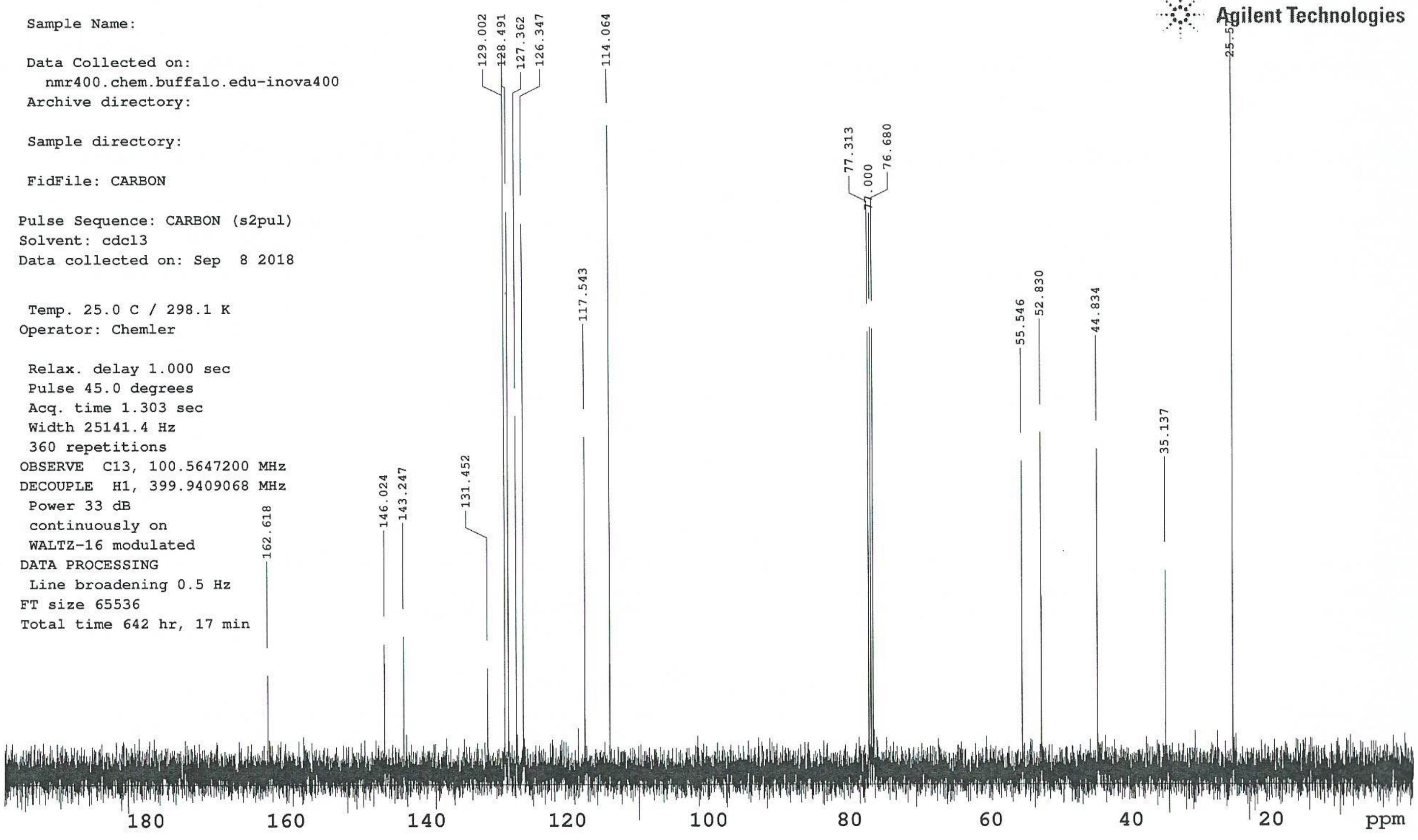

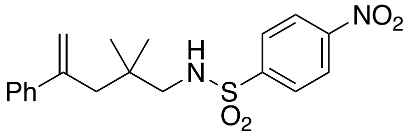
Ph
H
N
S
O2
NO2

***S*-12b**

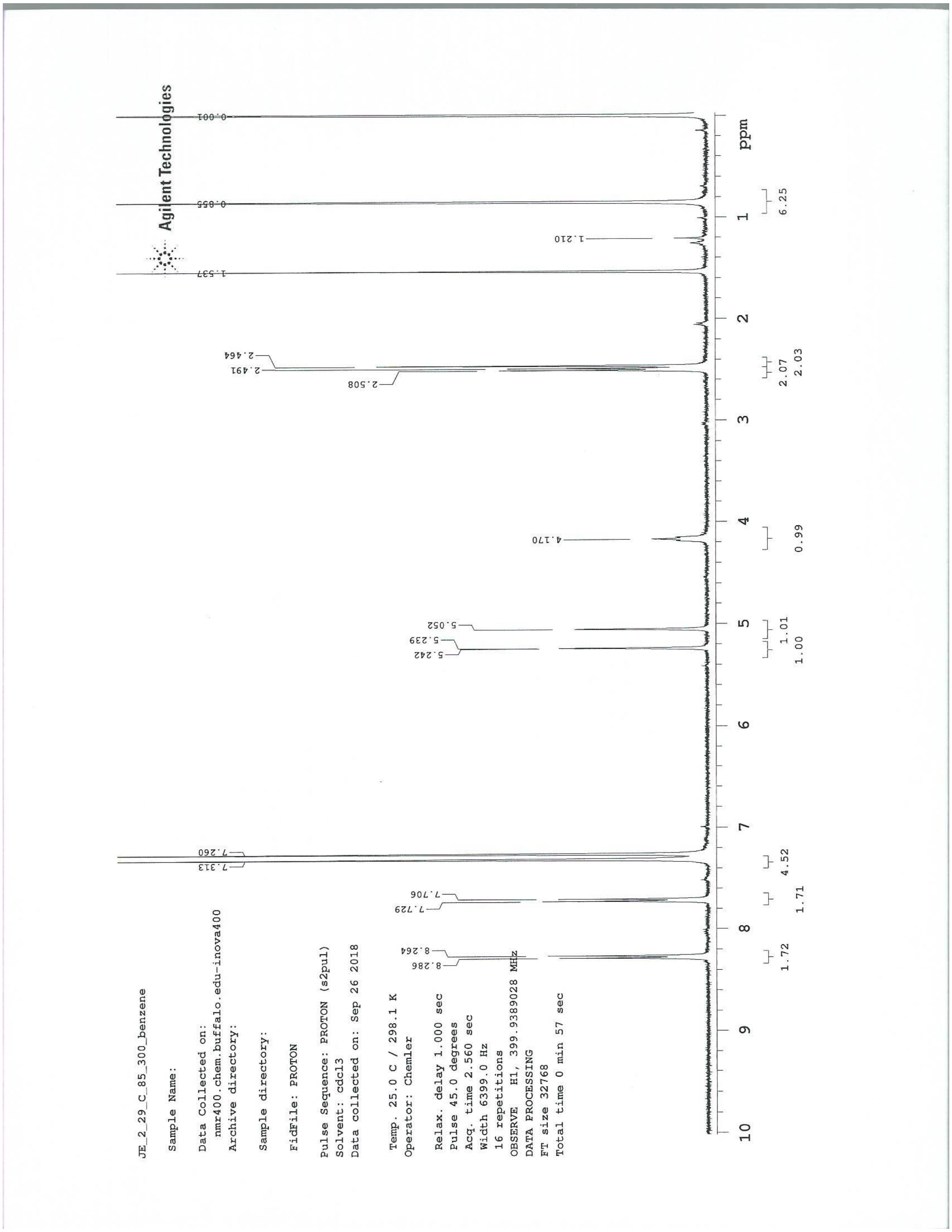
Agilent Technologies
JE_2_29_C_85_300_benzene
Sample Name:
Data Collected on:
nmr400.chem.buffalo.edu-inova400
Archive directory:
Sample directory:
FidFile: PROTON
Pulse Sequence: PROTON (s2pul)
Solvent: cdcl3
Data collected on: Sep 26 2018
Temp. 25.0 C / 298.1 K
Operator: Chemler
Relax. delay 1.000 sec
Pulse 45.0 degrees
Acq. time 2.560 sec
Width 6399.0 Hz
16 repetitions
OBSERVE H1, 399.9389028 MHz
DATA PROCESSING
FT size 32768
Total time 0 min 57 sec
0.001
0.855
1.210
1.537
2.464
2.491
2.508
4.170
5.052
5.239
5.242
7.260
7.313
7.706
7.729
8.264
8.286
6.25
2.07
2.03
0.99
1.01
1.00
4.52
1.71
1.72
ppm

JE_2_33_B

Sample Name:

Data Collected on:
nmr400.chem.buffalo.edu-inova400
Archive directory:

Sample directory:

FidFile: CARBON

Pulse Sequence: CARBON (s2pul)
Solvent: c6d6
Data collected on: Sep 25 2018

Temp. 25.0 C / 298.1 K
Operator: Chemler

Relax. delay 1.000 sec
Pulse 45.0 degrees
Acq. time 1.303 sec
Width 25141.4 Hz
1048 repetitions
OBSERVE C13, 100.5647199 MHz
DECOUPLE H1, 399.9409428 MHz
Power 33 dB
continuously on
WALTZ-16 modulated
DATA PROCESSING
Line broadening 0.5 Hz
FT size 65536
Total time 64 hr, 13 min

Agilent Technologies

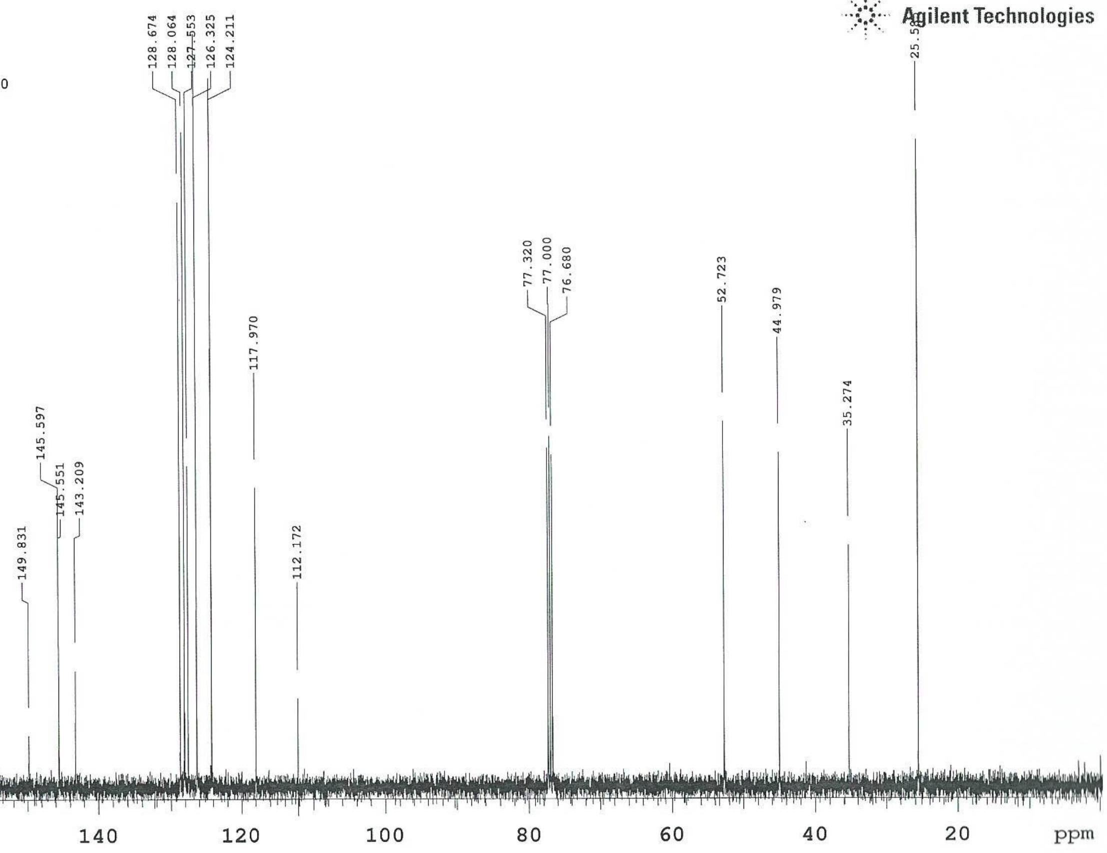

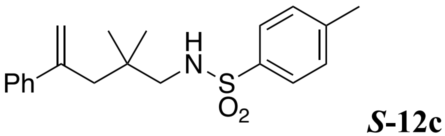

***S*-12c**

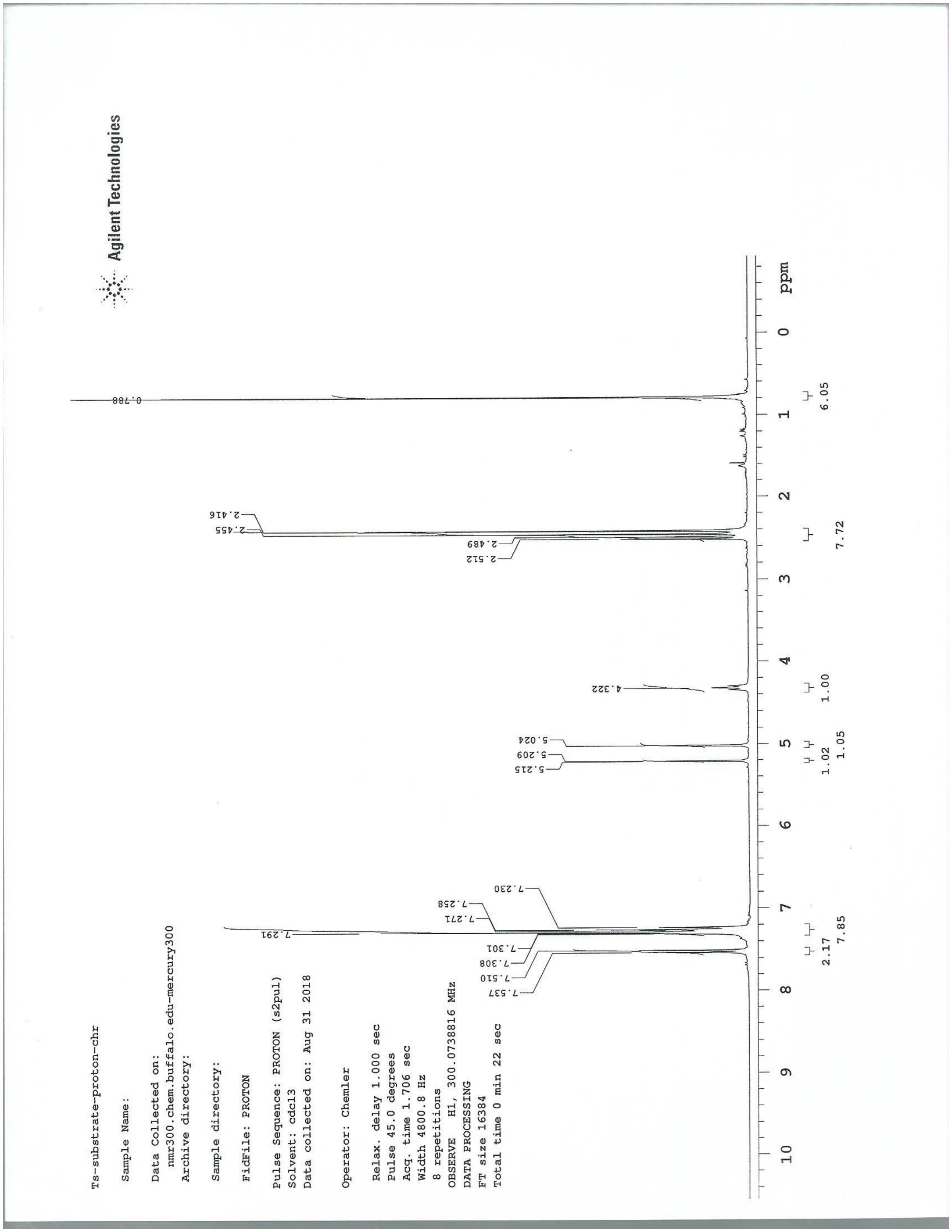
Agilent Technologies
Ts-substrate-proton-chr
Sample Name:
Data Collected on:
nmr300.chem.buffalo.edu-mercury300
Archive directory:
Sample directory:
FidFile: PROTON
Pulse Sequence: PROTON (s2pul)
Solvent: cdcl3
Data collected on: Aug 31 2018
Operator: Chemler
Relax. delay 1.000 sec
Pulse 45.0 degrees
Acq. time 1.706 sec
Width 4800.8 Hz
8 repetitions
OBSERVE H1, 300.0738816 MHz
DATA PROCESSING
FT size 16384
Total time 0 min 22 sec
0.788
2.416
2.455
2.489
2.512
4.322
5.024
5.209
5.215
7.230
7.258
7.271
7.291
7.301
7.308
7.510
7.537
6.05
7.72
1.00
1.02
1.05
2.17
7.85
ppm

Ts-substrate-proton-chr

Sample Name:

Data Collected on:
nmr300.chem.buffalo.edu-mercury300
Archive directory:

Sample directory:

FidFile: CARBON

Pulse Sequence: CARBON (s2pul)
Solvent: cdcl3
Data collected on: Aug 31 2018

Operator: Chemler

Relax. delay 1.000 sec
Pulse 45.0 degrees
Acq. time 0.868 sec
Width 18867.9 Hz
2240 repetitions
OBSERVE C13, 75.4536400 MHz
DECOUPLE H1, 300.0754430 MHz
Power 37 dB
continuously on
WALTZ-16 modulated
DATA PROCESSING
Line broadening 0.5 Hz
FT size 32768
Total time 643 hr, 45 min

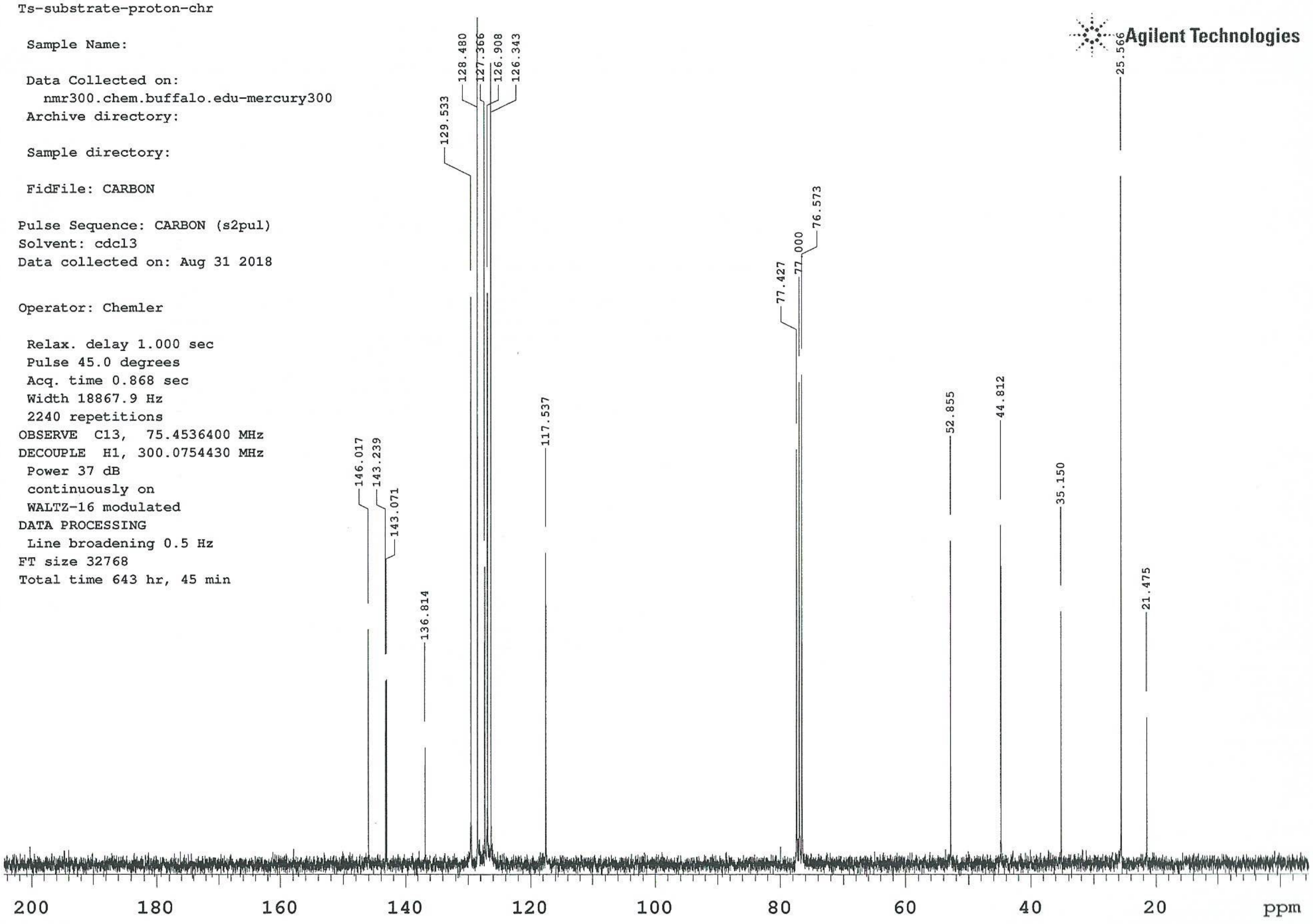

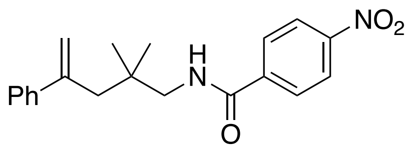

NO2
H
N
Ph
O

***S*-12e**

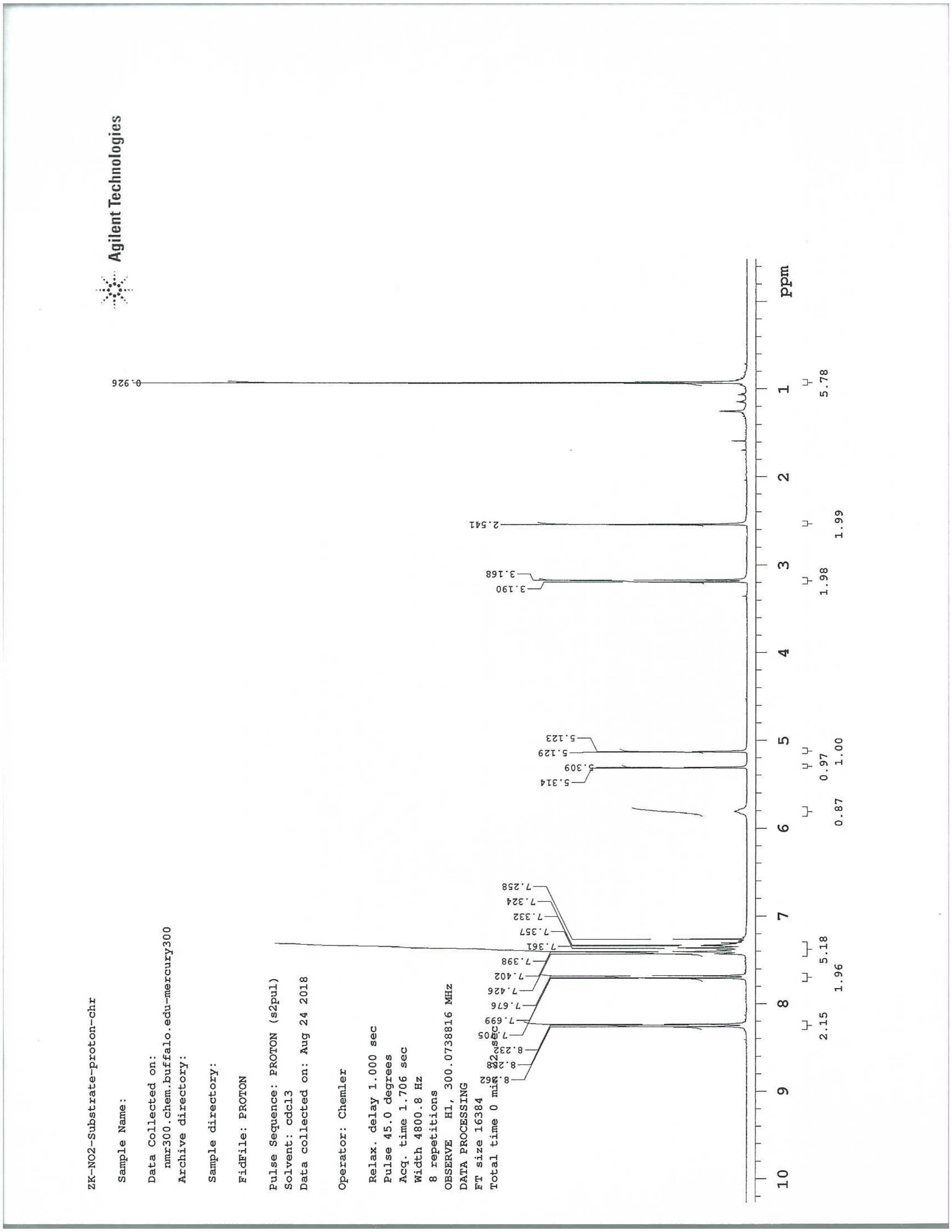

Agilent Technologies
ZK-NO2-Substrate-proton-chr
Sample Name:
Data Collected on:
nmr300.chem.buffalo.edu-mercury300
Archive directory:
Sample directory:
FidFile: PROTON
Pulse Sequence: PROTON (s2pul)
Solvent: cdcl3
Data collected on: Aug 24 2018
Operator: Chemler
Relax. delay 1.000 sec
Pulse 45.0 degrees
Acq. time 1.706 sec
Width 4800.8 Hz
8 repetitions
OBSERVE H1, 300.0738816 MHz
DATA PROCESSING
FT size 16384
0.926
2.541
3.168
3.190
5.123
5.129
5.309
5.314
7.258
7.324
7.332
7.357
7.361
7.398
7.402
7.426
7.676
7.699
8.232
8.288
5.78
1.99
1.98
0.97
1.00
0.87
5.18
1.96
2.15
ppm

ZK-NO2-Substrate-proton-chr

Sample Name:

Data Collected on:
nmr300.chem.buffalo.edu-mercury300
Archive directory:

Sample directory:

FidFile: ZK-NO2-Substrate-carbon-chr

Pulse Sequence: CARBON (s2pul)
Solvent: cdcl3
Data collected on: Aug 24 2018

Operator: Chemler

Relax. delay 1.000 sec
Pulse 45.0 degrees
Acq. time 0.868 sec
Width 18867.9 Hz
1088 repetitions
OBSERVE C13, 75.4536389 MHz
DECOUPLE H1, 300.0754430 MHz
Power 37 dB
continuously on
WALTZ-16 modulated
DATA PROCESSING
Line broadening 0.5 Hz
FT size 32768
Total time 643 hr, 45 min

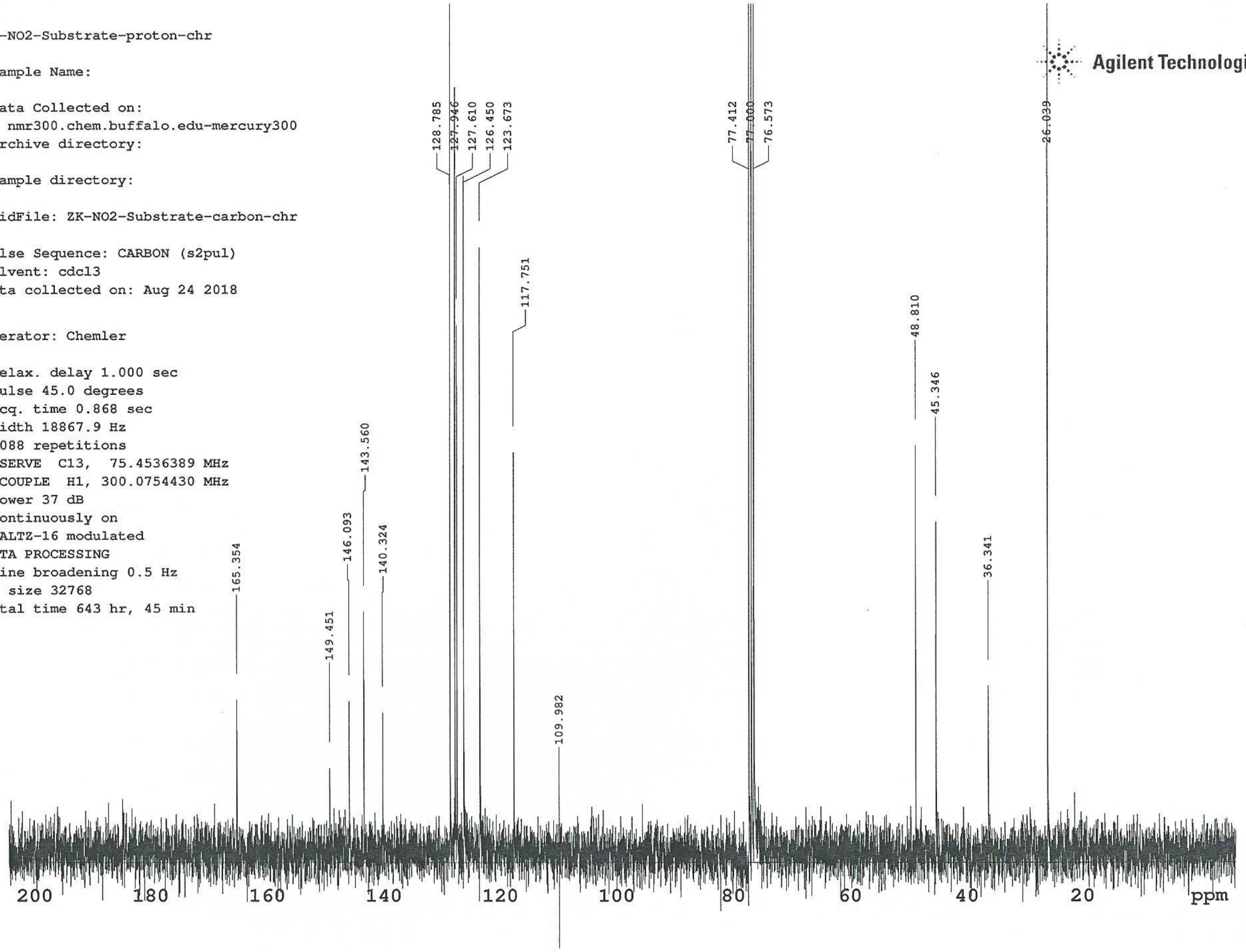

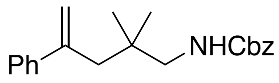
Ph
NHCbz

***S*-12f**

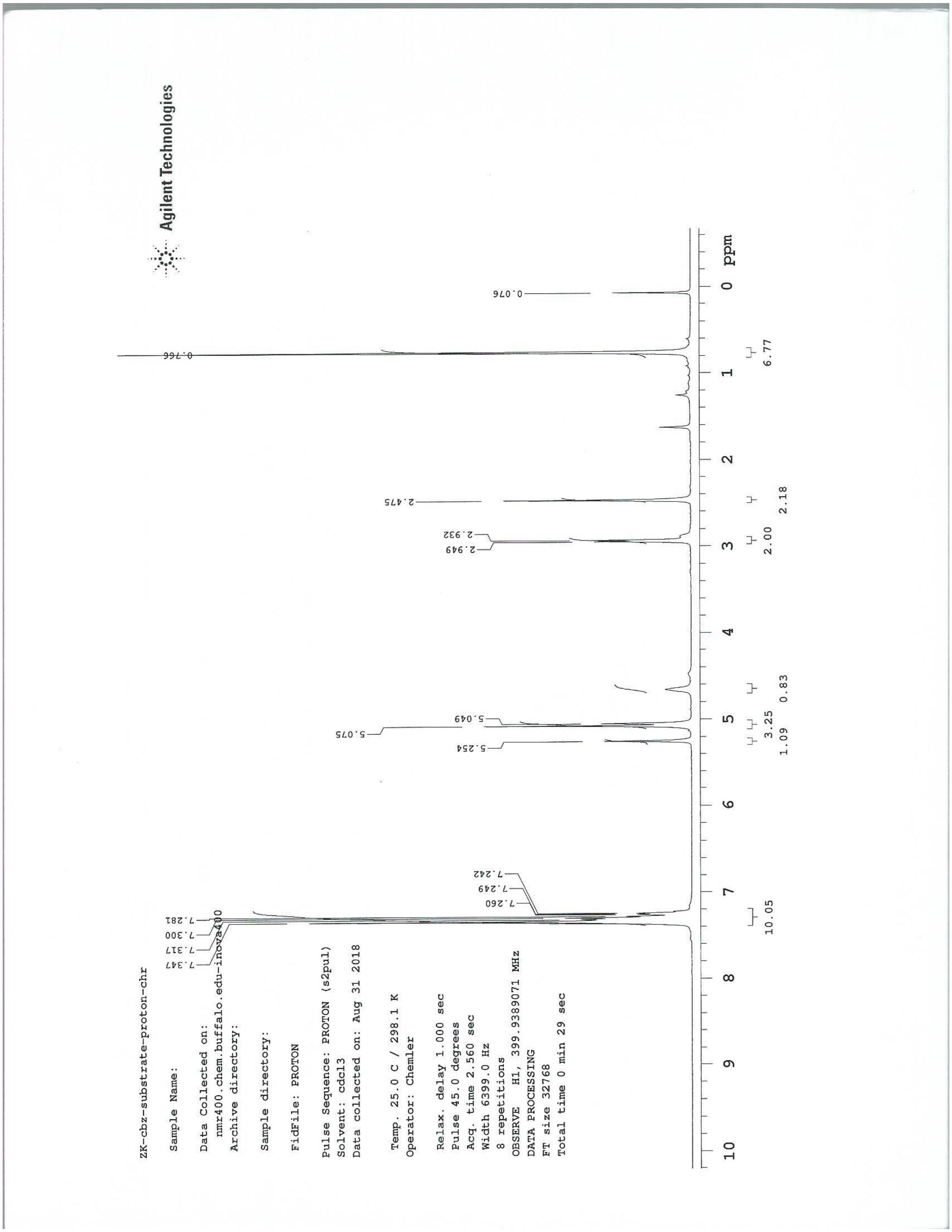
Agilent Technologies
ZK-cbz-substrate-proton-chr
Sample Name:
Data Collected on:
nmr400.chem.buffalo.edu-inova400
Archive directory:
Sample directory:
FidFile: PROTON
Pulse Sequence: PROTON (s2pul)
Solvent: cdcl3
Data collected on: Aug 31 2018
Temp. 25.0 C / 298.1 K
Operator: Chemler
Relax. delay 1.000 sec
Pulse 45.0 degrees
Acq. time 2.560 sec
Width 6399.0 Hz
8 repetitions
OBSERVE H1, 399.9389071 MHz
DATA PROCESSING
FT size 32768
Total time 0 min 29 sec
7.347
7.317
7.300
7.281
7.260
7.249
7.242
5.254
5.075
5.049
2.949
2.932
2.475
0.766
0.076
10.05
1.09
3.25
0.83
2.00
2.18
6.77
10
9
8
7
6
5
4
3
2
1
0
ppm

ZK-cbz-substrate-carbon-chr

Sample Name:

Data Collected on:
nmr400.chem.buffalo.edu-inova400
Archive directory:

Sample directory:

FidFile: CARBON

Pulse Sequence: CARBON (s2pul)
Solvent: cdcl3
Data collected on: Aug 31 2018

Temp. 25.0 C / 298.1 K
Operator: Chemler

Relax. delay 1.000 sec
Pulse 45.0 degrees
Acq. time 1.303 sec
Width 25141.4 Hz
2112 repetitions
OBSERVE C13, 100.5647207 MHz
DECOUPLE H1, 399.9409068 MHz
Power 33 dB
continuously on
WALTZ-16 modulated
DATA PROCESSING
Line broadening 0.5 Hz
FT size 65536
Total time 792 hr, 56 min

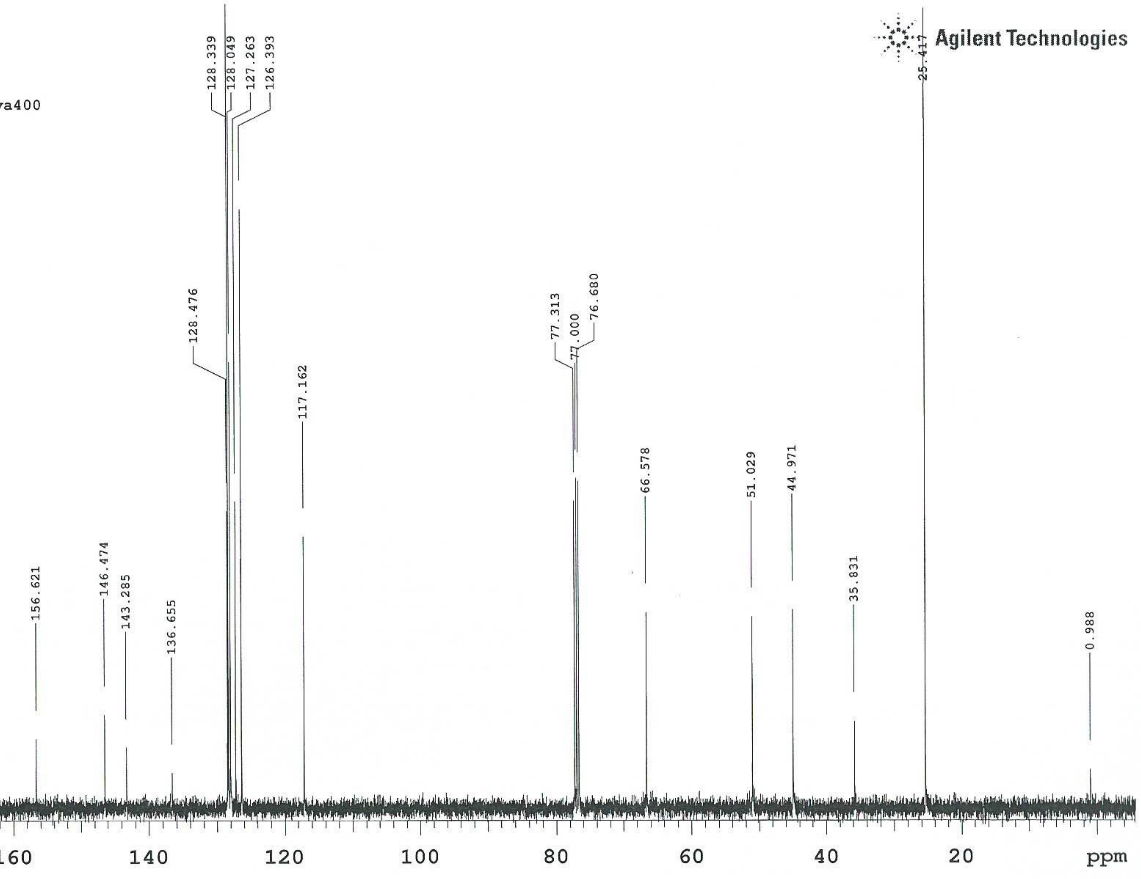

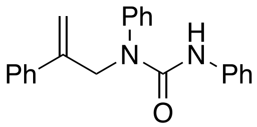
Ph
Ph
N
H
N
Ph
O

***S*-12g**

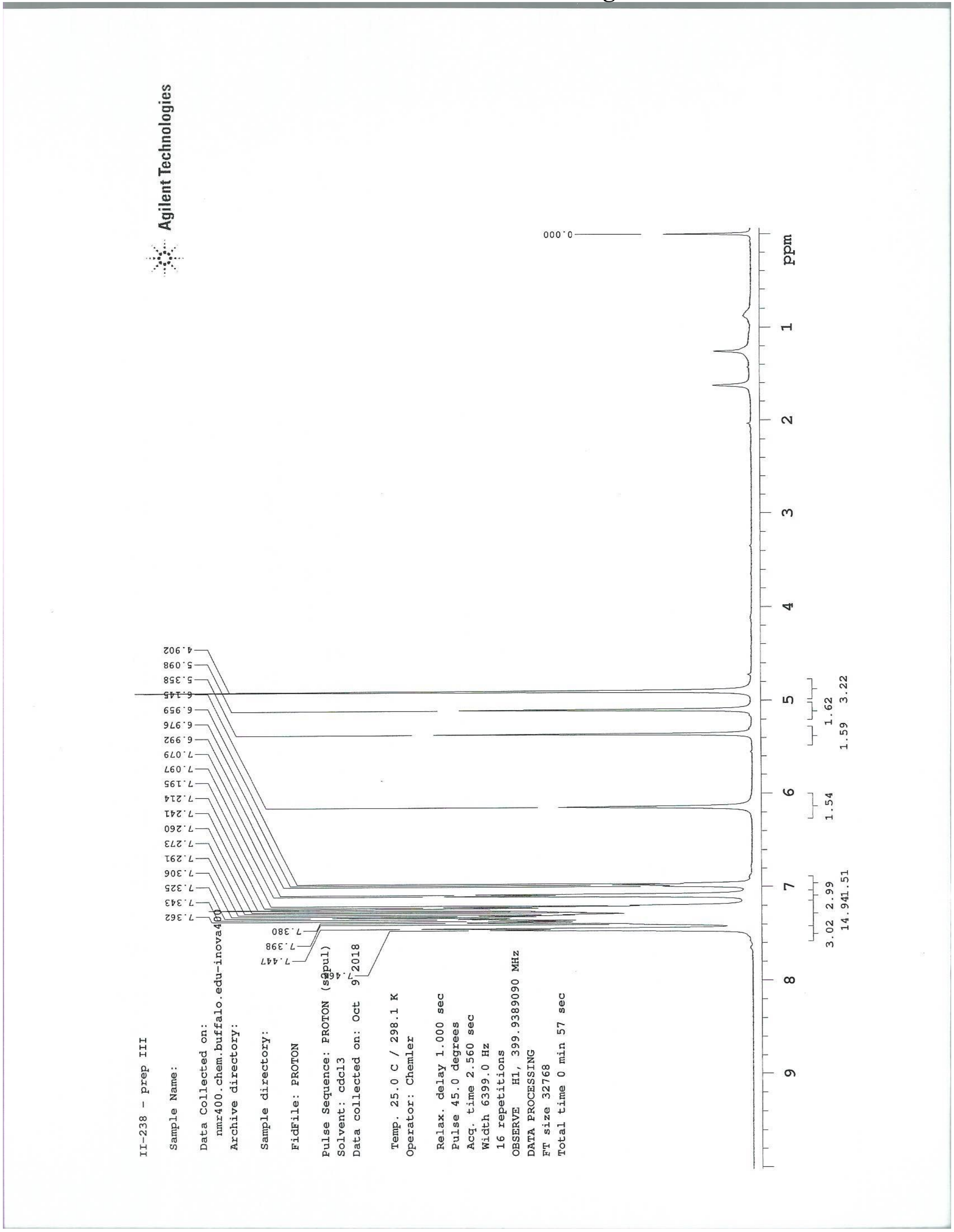
Agilent Technologies
II-238 - prep III
Sample Name:
Data Collected on:
nmr400.chem.buffalo.edu-inova400
Archive directory:
Sample directory:
FidFile: PROTON
Pulse Sequence: PROTON (s2pul)
Solvent: cdcl3
Data collected on: Oct 9 2018
Temp. 25.0 C / 298.1 K
Operator: Chemler
Relax. delay 1.000 sec
Pulse 45.0 degrees
Acq. time 2.560 sec
Width 6399.0 Hz
16 repetitions
OBSERVE H1, 399.9389090 MHz
DATA PROCESSING
FT size 32768
Total time 0 min 57 sec
0.000
4.902
5.098
5.358
6.145
6.959
6.976
6.992
7.079
7.097
7.195
7.214
7.241
7.260
7.273
7.291
7.306
7.325
7.343
7.362
7.380
7.398
7.447
7.465
3.02 2.99
14.941.51
1.54
1.59 1.62 3.22
ppm

SB_183_crude

Sample Name:

Data Collected on:
nmr400.chem.buffalo.edu-inova400
Archive directory:

Sample directory:

FidFile: CARBON

Pulse Sequence: CARBON (s2pul)
Solvent: cdcl3
Data collected on: Oct 6 2018

Temp. 25.0 C / 298.1 K
Operator: Chemler

Relax. delay 1.000 sec
Pulse 45.0 degrees
Acq. time 1.303 sec
Width 25141.4 Hz
368 repetitions
OBSERVE C13, 100.5647246 MHz
DECOUPLE H1, 399.9409068 MHz
Power 33 dB
continuously on
WALTZ-16 modulated
DATA PROCESSING
Line broadening 0.5 Hz
FT size 65536
Total time 6422 hr, 50 min

Agilent Technologies

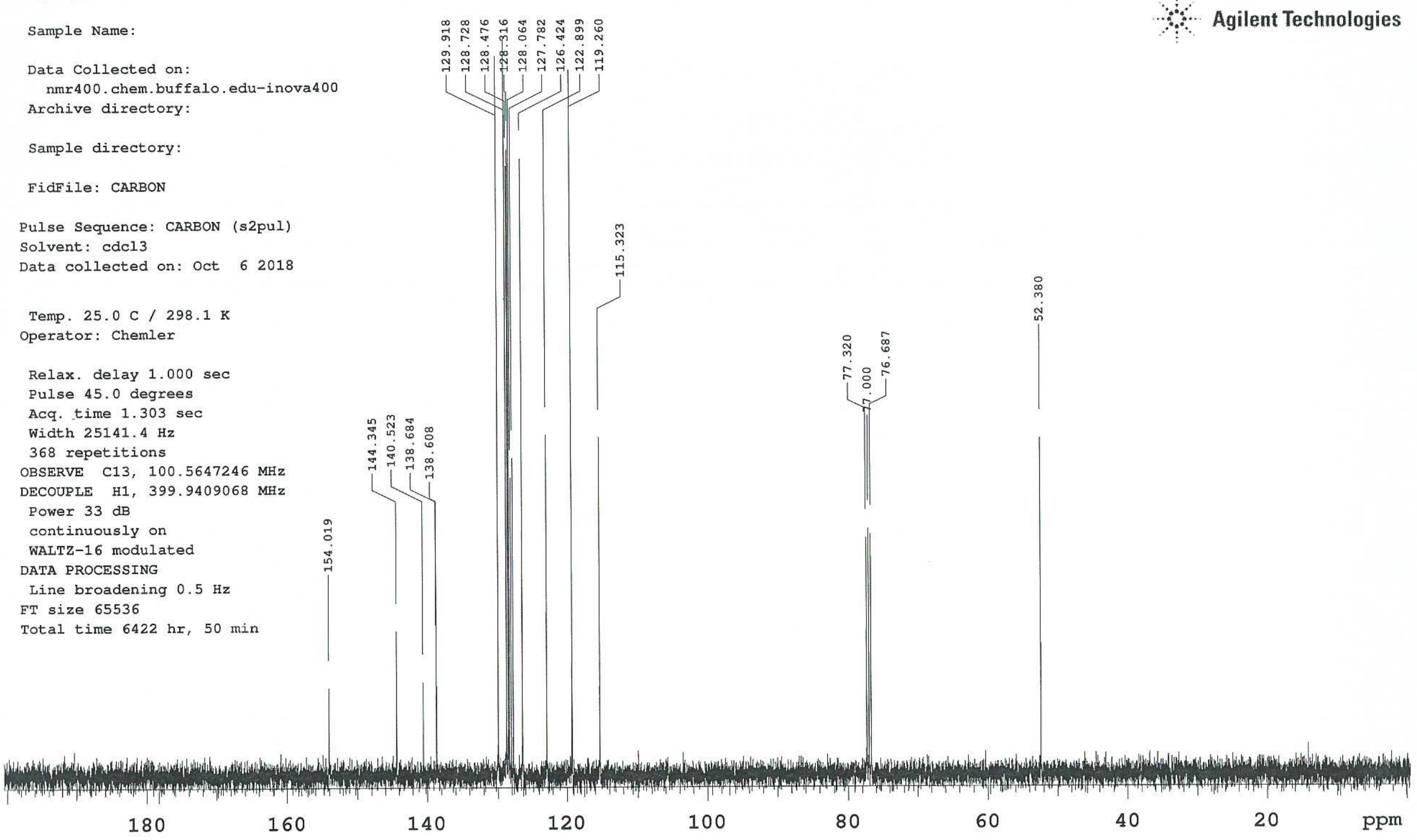

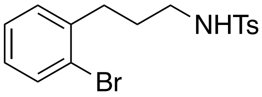

NHTs
Br

***S*-3**

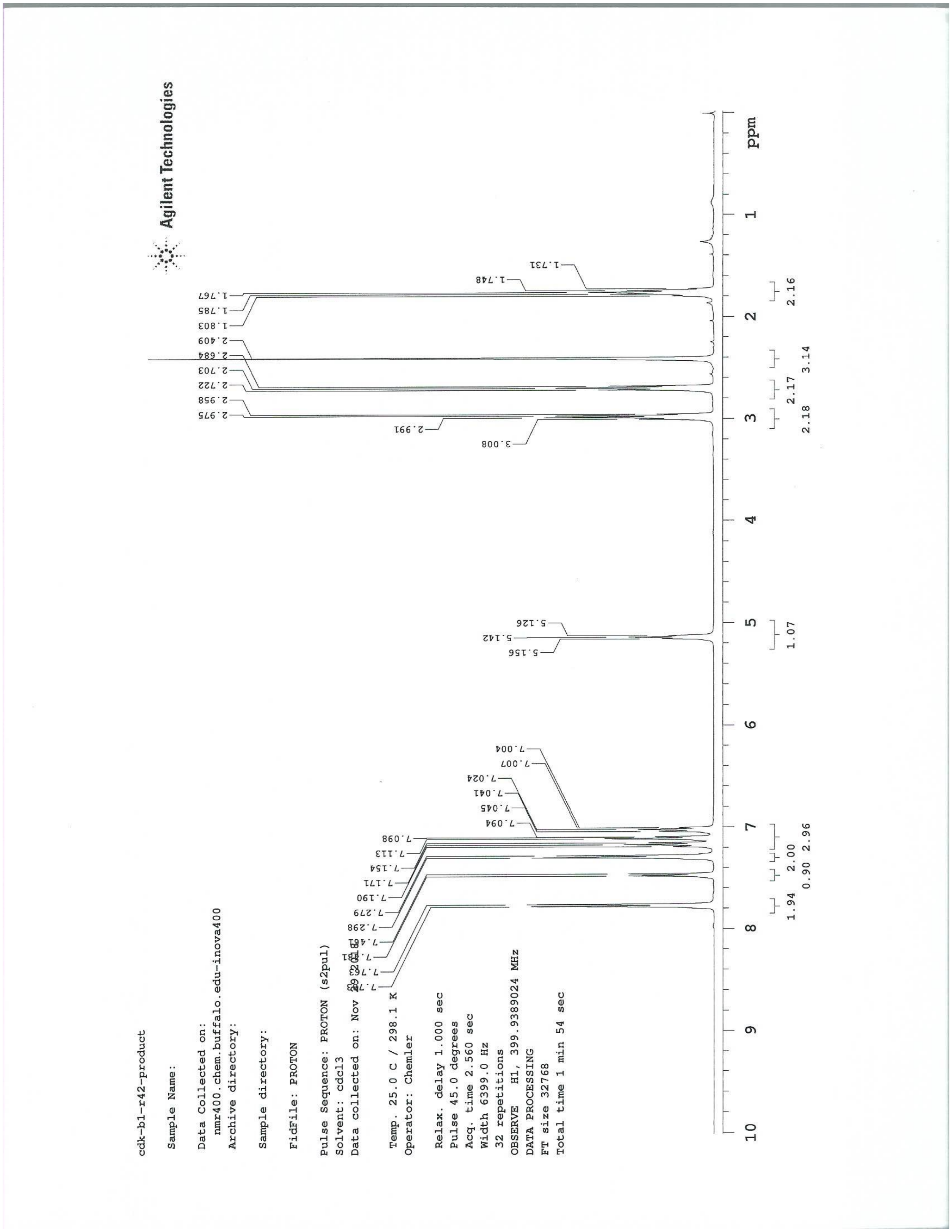

Agilent Technologies
cdk-bl-r42-product
Sample Name:
Data Collected on:
nmr400.chem.buffalo.edu-inova400
Archive directory:
Sample directory:
FidFile: PROTON
Pulse Sequence: PROTON (s2pul)
Solvent: cdcl3
Data collected on: Nov
Temp. 25.0 C / 298.1 K
Operator: Chemler
Relax. delay 1.000 sec
Pulse 45.0 degrees
Acq. time 2.560 sec
Width 6399.0 Hz
32 repetitions
OBSERVE H1, 399.9389024 MHz
DATA PROCESSING
FT size 32768
Total time 1 min 54 sec
1.731
1.748
1.767
1.785
1.803
2.409
2.684
2.703
2.722
2.958
2.975
2.991
3.008
5.126
5.142
5.156
7.004
7.007
7.024
7.041
7.045
7.094
7.098
7.113
7.154
7.171
7.190
7.279
7.298
2.16
3.14
2.17
2.18
1.07
2.96
2.00
0.90
1.94
ppm
1
2
3
4
5
6
7
8
9
10

cdk-b1-r42-product

Sample Name:

Data Collected on:
nmr400.chem.buffalo.edu-inova400
Archive directory:

Sample directory:

FidFile: CARBON

Pulse Sequence: CARBON (s2pul)
Solvent: cdcl3
Data collected on: Nov 29 2018

Temp. 25.0 C / 298.1 K
Operator: Chemler

Relax. delay 2.000 sec
Pulse 45.0 degrees
Acq. time 1.303 sec
Width 25141.4 Hz
176 repetitions
OBSERVE C13, 100.5647299 MHz
DECOUPLE H1, 399.9409068 MHz
Power 33 dB
continuously on
WALTZ-16 modulated
DATA PROCESSING
Line broadening 0.5 Hz
FT size 65536
Total time 92 hr

Agilent Technologies

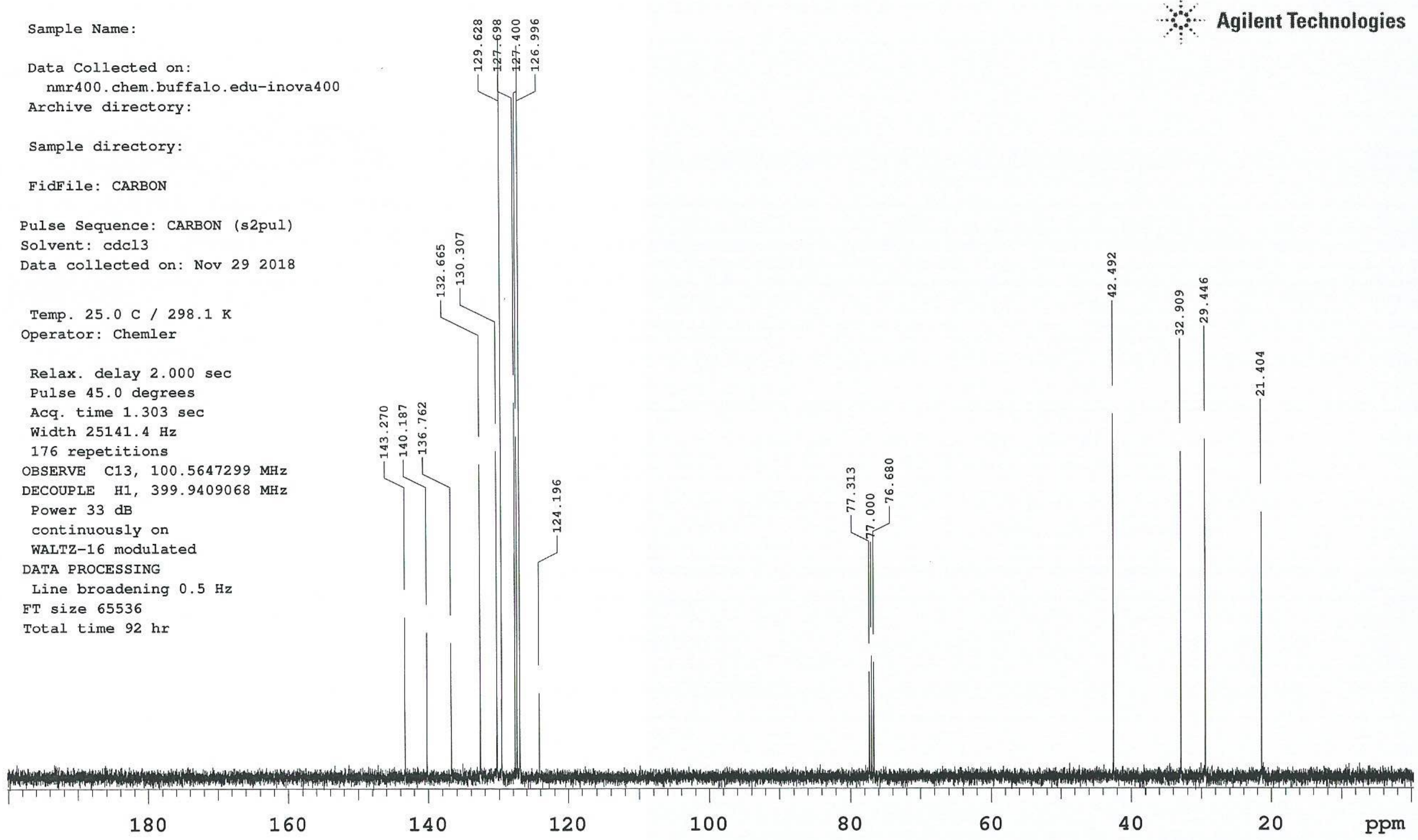

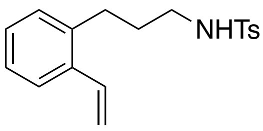
NHTs

***S*-15**

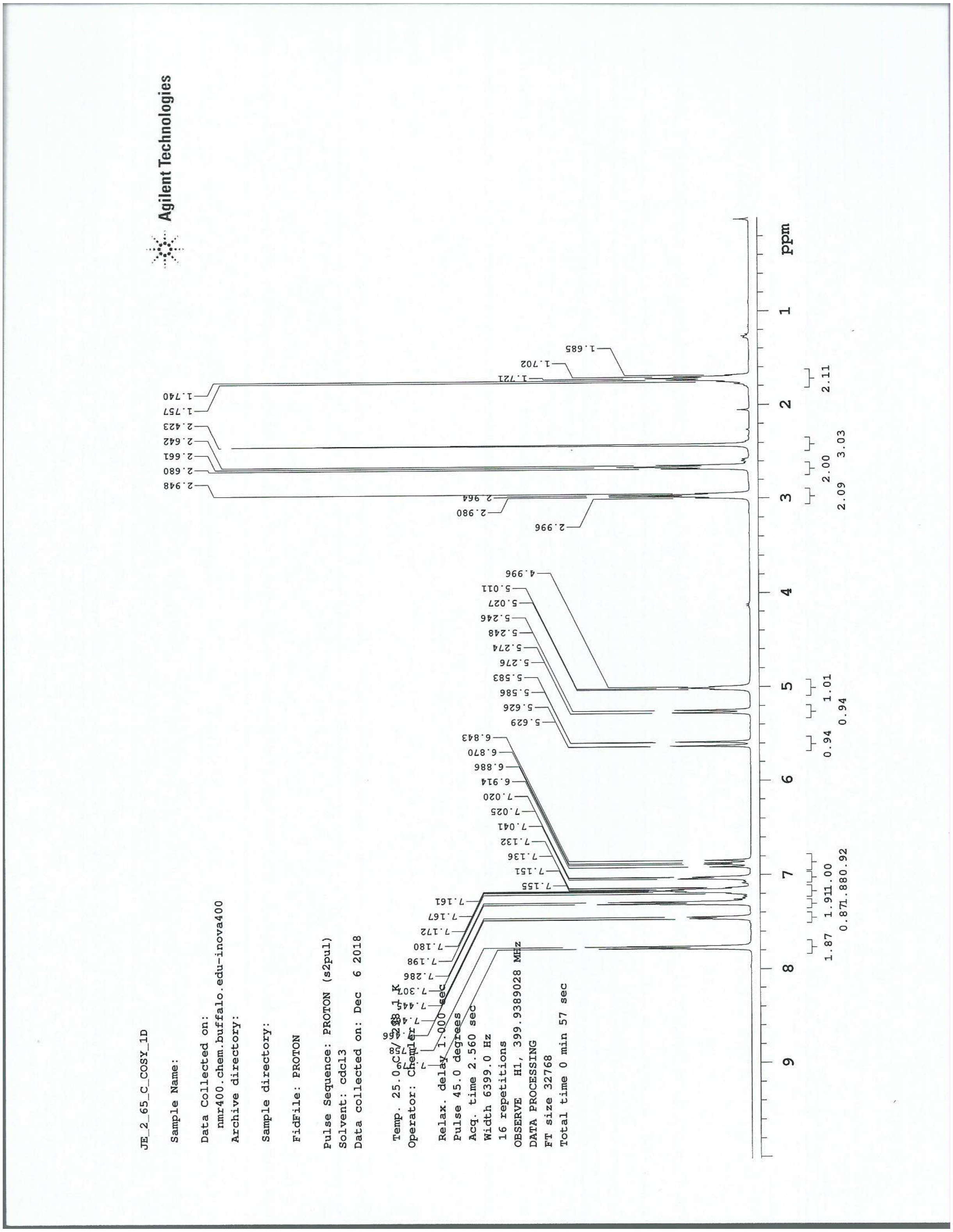
Agilent Technologies
JE_2_65_C_COSY_1D
Sample Name:
Data Collected on:
nmr400.chem.buffalo.edu-inova400
Archive directory:
Sample directory:
FidFile: PROTON
Pulse Sequence: PROTON (s2pul)
Solvent: cdcl3
Data collected on: Dec 6 2018
Relax. delay 1.000 sec
Pulse 45.0 degrees
Acq. time 2.560 sec
Width 6399.0 Hz
16 repetitions
OBSERVE H1, 399.9389028 MHz
DATA PROCESSING
FT size 32768
Total time 0 min 57 sec
ppm

JE_2_67_G

Sample Name:

Data Collected on:
nmr400.chem.buffalo.edu-inova400
Archive directory:

Sample directory:

FidFile: CARBON

Pulse Sequence: CARBON (s2pul)
Solvent: cdcl3
Data collected on: Dec 4 2018

Temp. 25.0 C / 298.1 K
Operator: Chemler

Relax. delay 2.000 sec
Pulse 45.0 degrees
Acq. time 1.303 sec
Width 25141.4 Hz
528 repetitions
OBSERVE C13, 100.5647223 MHz
DECOUPLE H1, 399.9409068 MHz
Power 33 dB
continuously on
WALTZ-16 modulated
DATA PROCESSING
Line broadening 0.5 Hz
FT size 65536
Total time 9 hr, 12 min

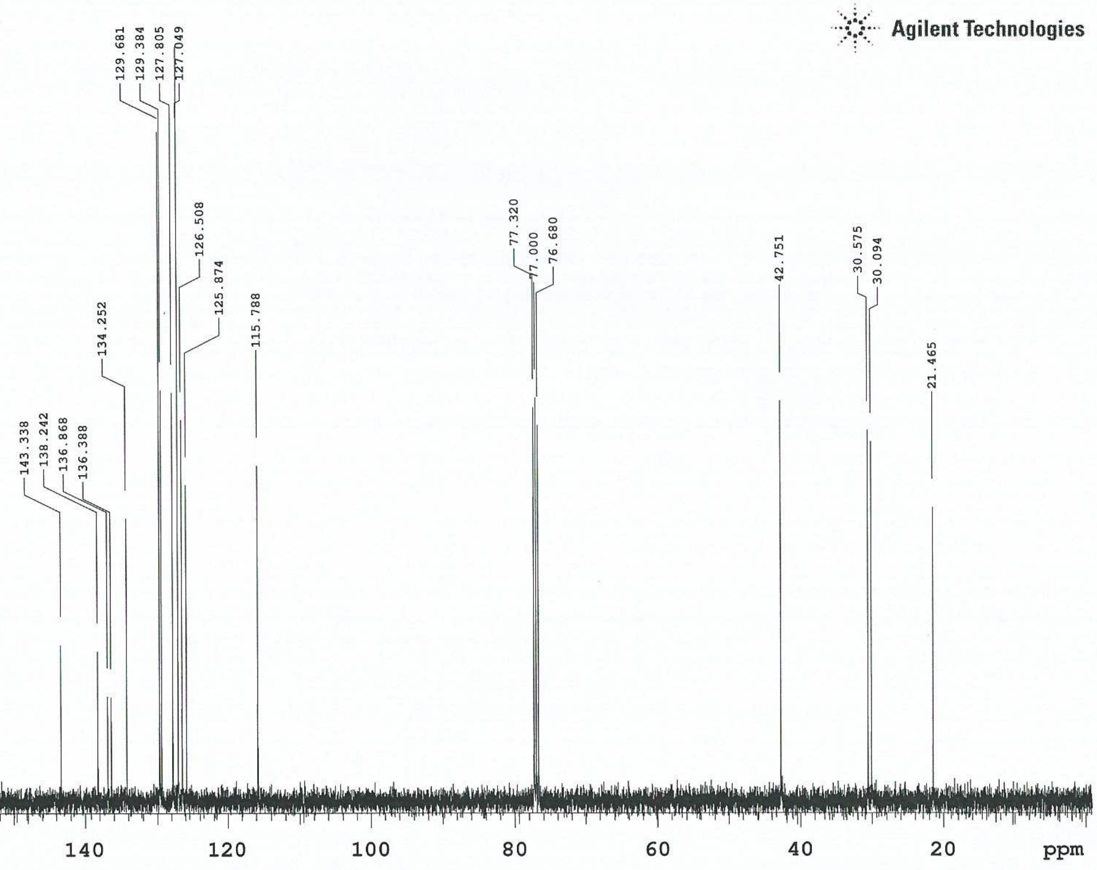

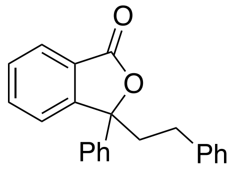
O
O
Ph
Ph

2a

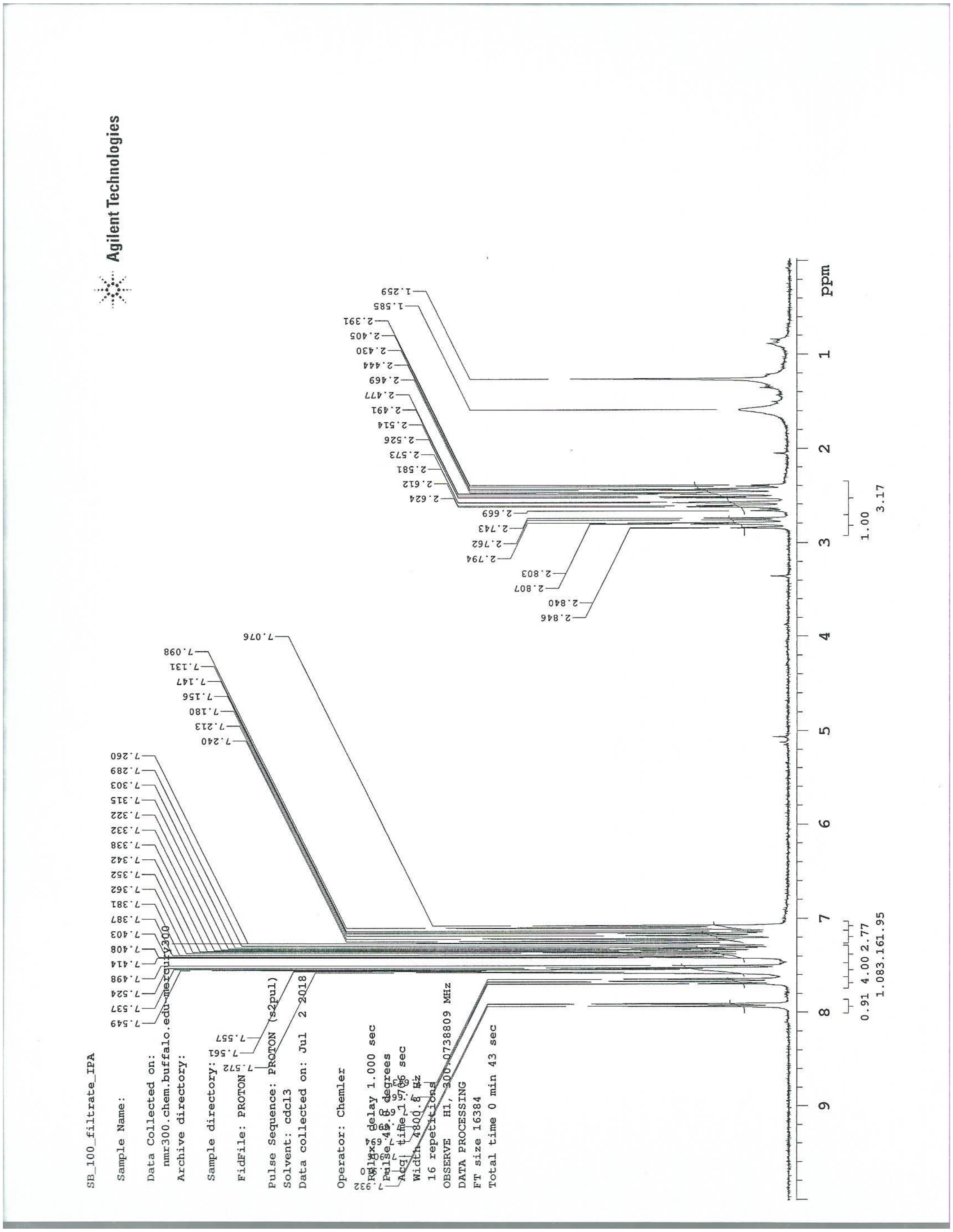
Agilent Technologies
SB_100_filtrate_IPA
Sample Name:
Data Collected on:
nmr300.chem.buffalo.edu-mercury300
Archive directory:
Sample directory:
FidFile: PROTON
Pulse Sequence: PROTON (s2pul)
Solvent: cdcl3
Data collected on: Jul 2 2018
Operator: Chemler
Relax. delay 1.000 sec
Pulse 45.0 degrees
Acq. time 1.706 sec
Width 4800.8 Hz
16 repetitions
OBSERVE H1, 300.0738809 MHz
DATA PROCESSING
FT size 16384
Total time 0 min 43 sec
ppm

SB_100_filtrate_IPA

Sample Name:

Data Collected on:
nmr300.chem.buffalo.edu-mercury300
Archive directory:

Sample directory:

FidFile: CARBON

Pulse Sequence: CARBON (s2pul)
Solvent: cdcl3
Data collected on: Jul 2 2018

Operator: Chemler

Relax. delay 1.000 sec
Pulse 45.0 degrees
Acq. time 0.868 sec
Width 18867.9 Hz
2080 repetitions
OBSERVE C13, 75.4536377 MHz
DECOUPLE H1, 300.0754430 MHz
Power 37 dB
continuously on
WALTZ-16 modulated
DATA PROCESSING
Line broadening 0.5 Hz
FT size 32768
Total time 521 hr, 26 min

Agilent Technologies

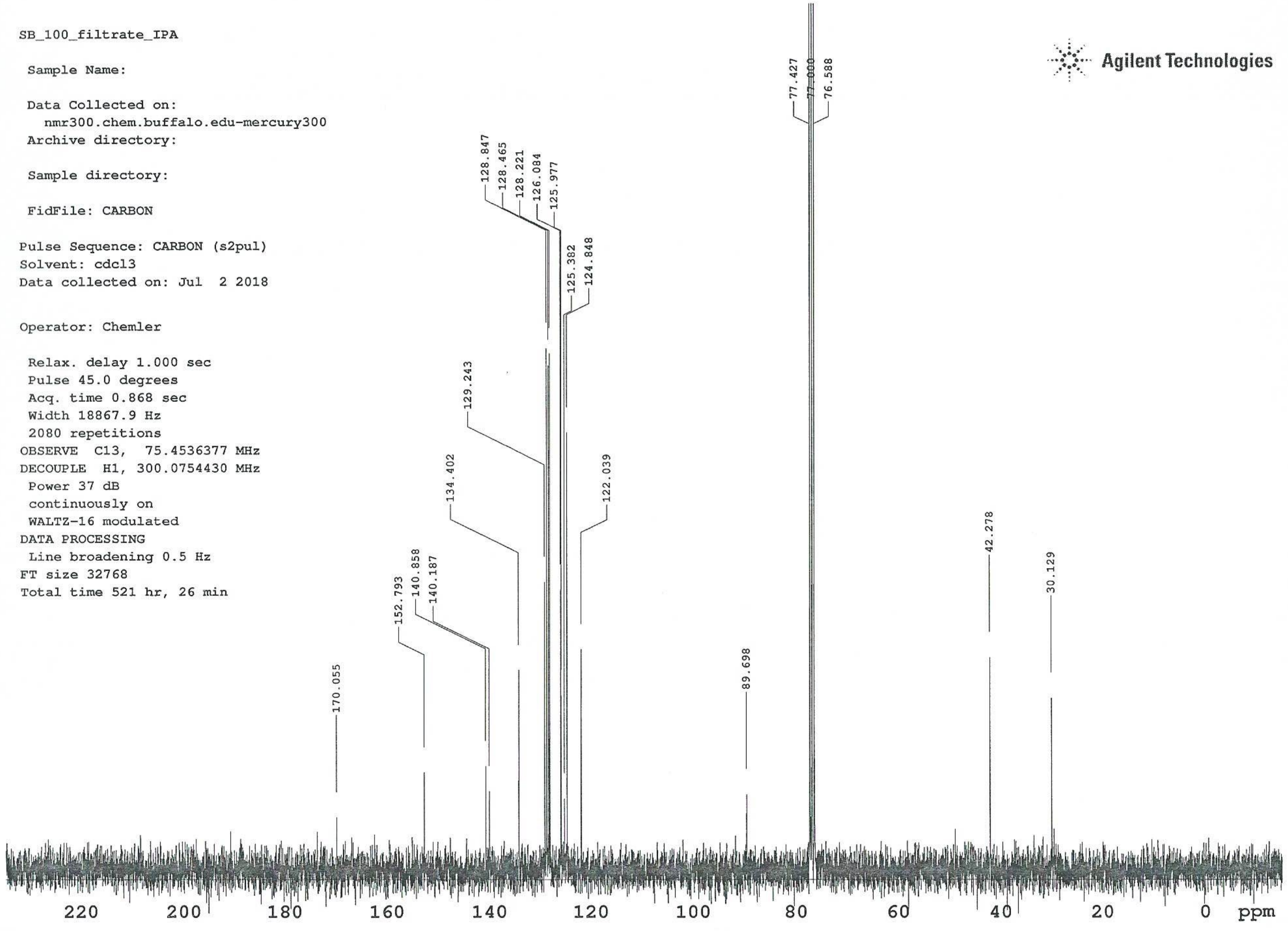

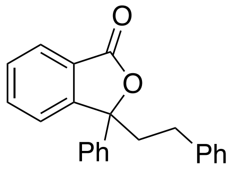
O
O
Ph
Ph

2b

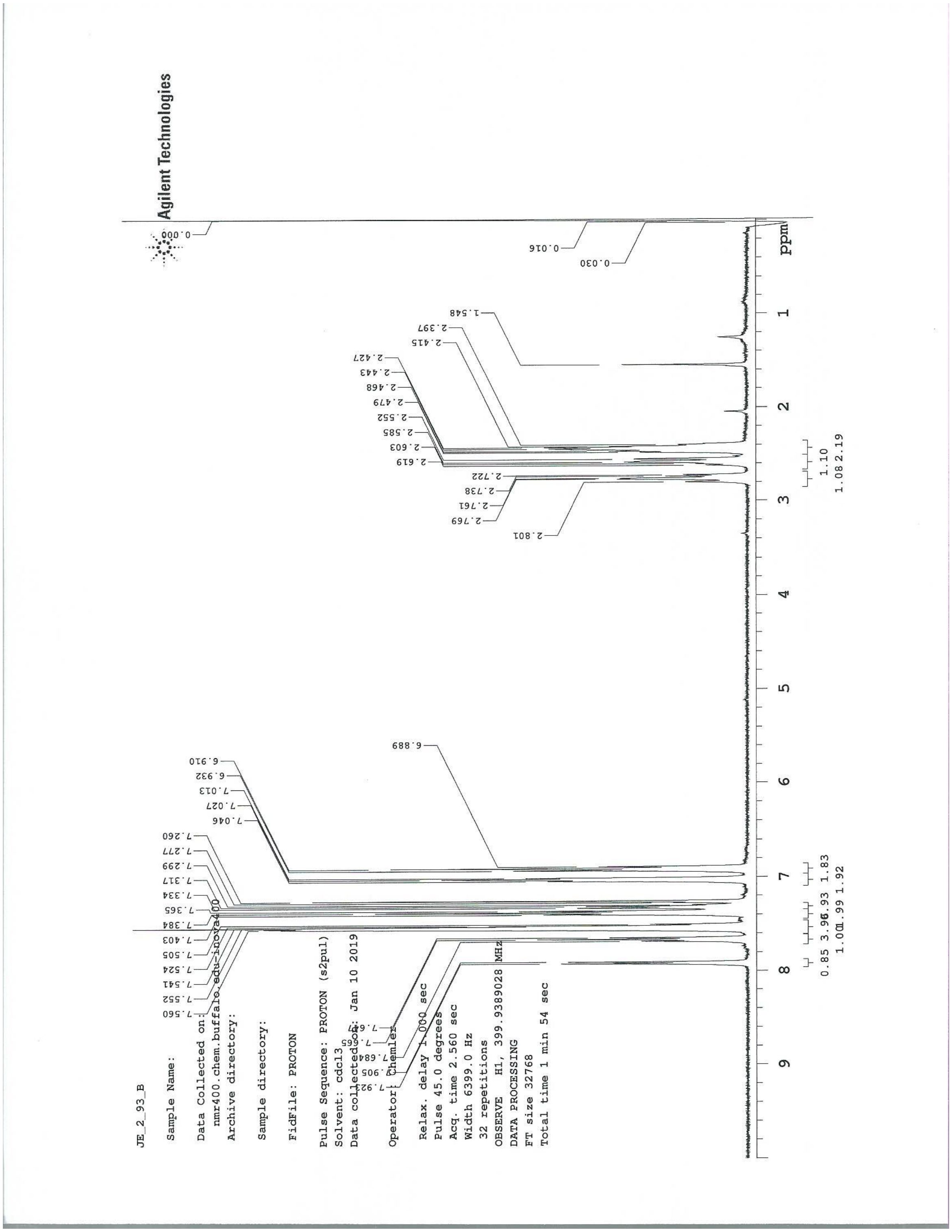
Agilent Technologies
JE_2_93_B
Sample Name:
Data Collected on:
nmr400.chem.buffalo.edu-inova400
Archive directory:
Sample directory:
FidFile: PROTON
Pulse Sequence: PROTON (s2pul)
Solvent: cdcl3
Data collected on: Jan 10 2019
Operator: Chemler
Relax. delay 1.000 sec
Pulse 45.0 degrees
Acq. time 2.560 sec
Width 6399.0 Hz
32 repetitions
OBSERVE H1, 399.9389028 MHz
DATA PROCESSING
FT size 32768
Total time 1 min 54 sec
ppm

ib2_255_col_12-15

Sample Name:

Data Collected on:
nmr400.chem.buffalo.edu-inova400
Archive directory:

Sample directory:

FidFile: CARBON

Pulse Sequence: CARBON (s2pul)
Solvent: cdcl3
Data collected on: Jan 12 2019

Operator: Chemler

Relax. delay 2.000 sec
Pulse 45.0 degrees
Acq. time 1.303 sec
Width 25141.4 Hz
15600 repetitions
OBSERVE C13, 100.5647177 MHz
DECOUPLE H1, 399.9409068 MHz
Power 33 dB
continuously on
WALTZ-16 modulated
DATA PROCESSING
Line broadening 0.5 Hz
FT size 65536
Total time 9200 hr, 37 min

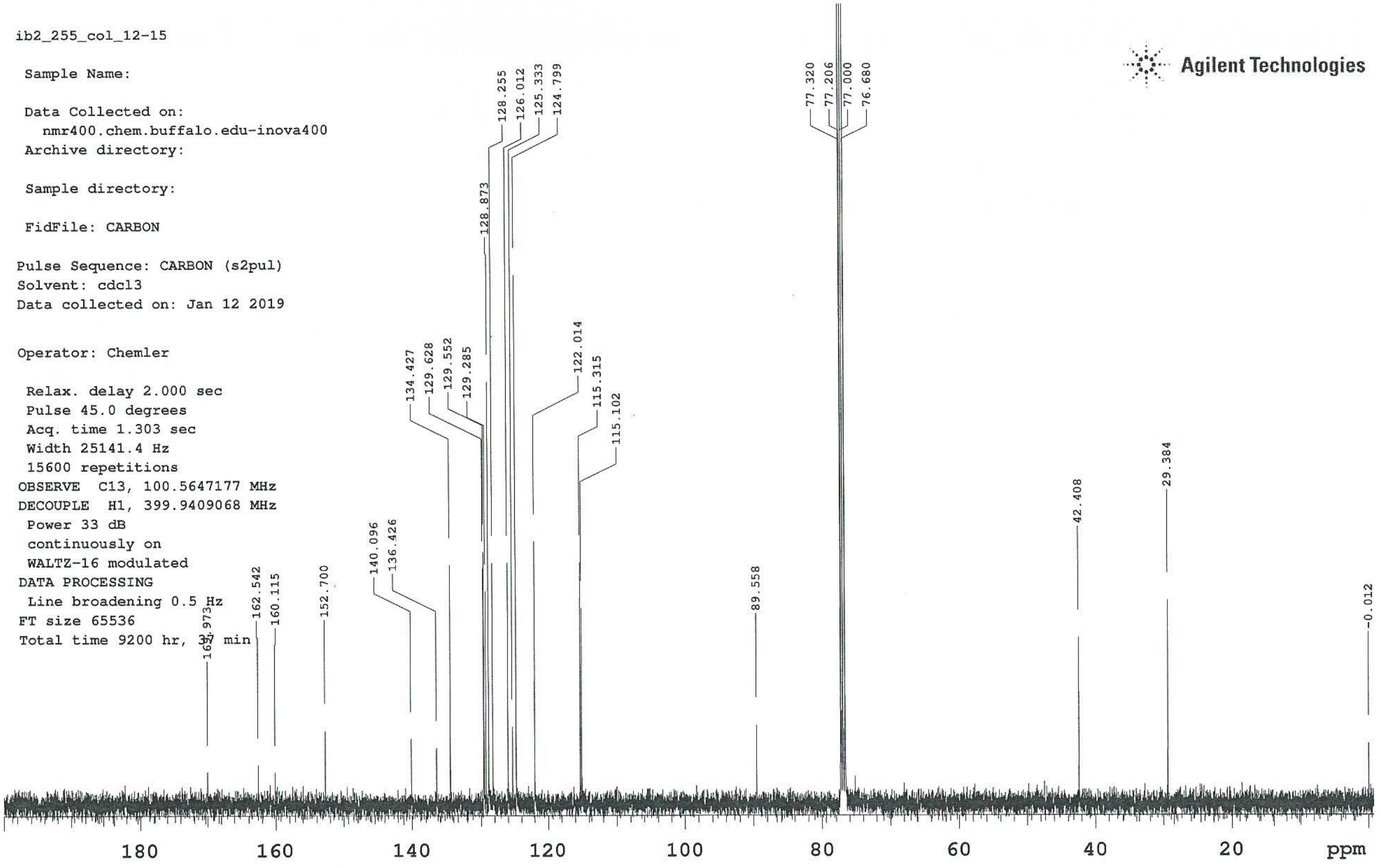

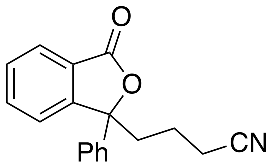

2c

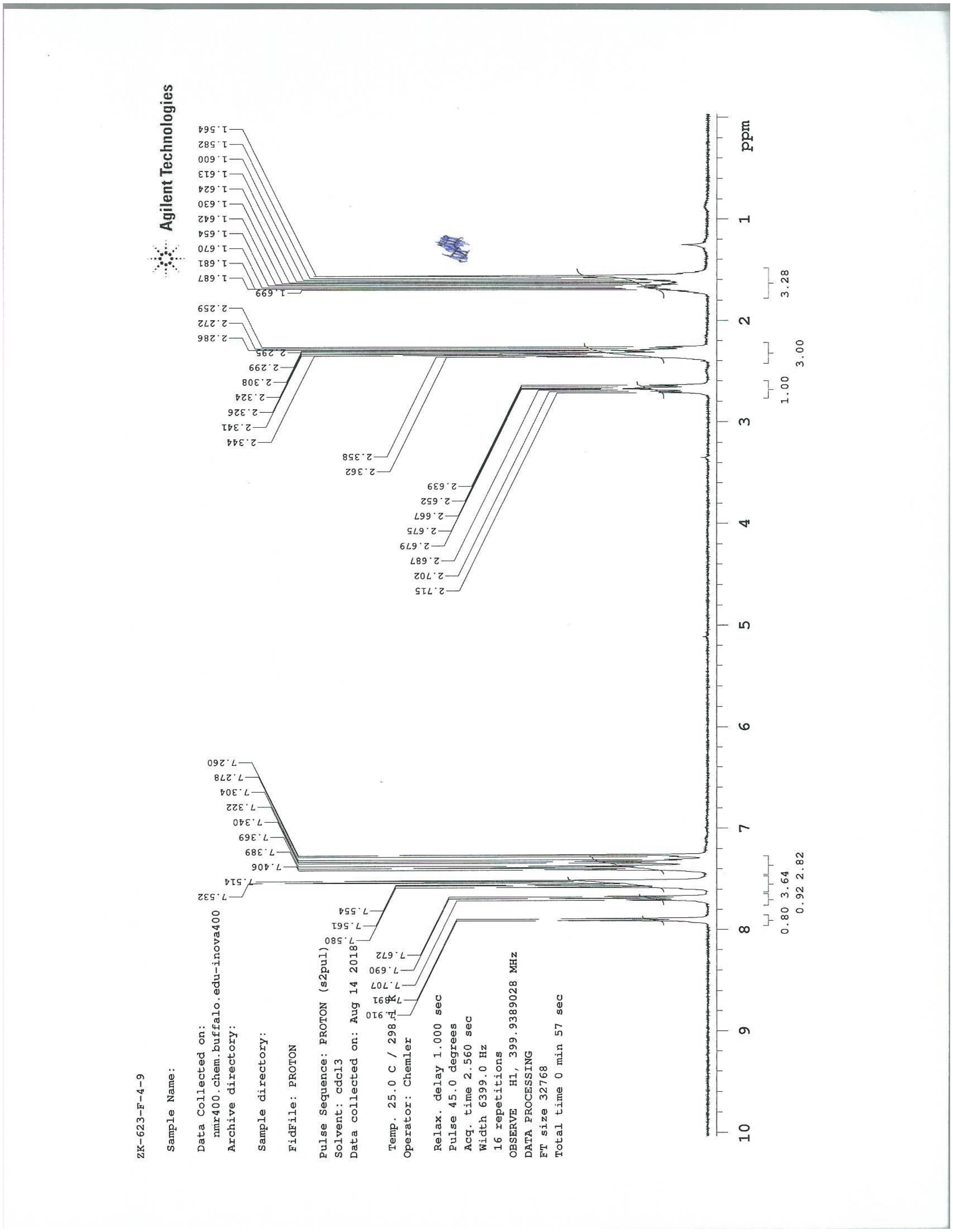
Agilent Technologies
1.564
1.582
1.600
1.613
1.624
1.630
1.642
1.654
1.670
1.681
1.687
1.699
2.259
2.272
2.286
2.295
2.299
2.308
2.324
2.326
2.341
2.344
2.358
2.362
2.639
2.652
2.667
2.675
2.679
2.687
2.702
2.715
7.260
7.278
7.304
7.322
7.340
7.369
7.389
7.406
7.514
7.532
7.554
7.561
7.580
7.672
7.690
7.707
7.891
7.910
ppm
3.28
3.00
1.00
0.80 3.64
0.92 2.82
ZK-623-F-4-9
Sample Name:
Data Collected on:
nmr400.chem.buffalo.edu-inova400
Archive directory:
Sample directory:
FidFile: PROTON
Pulse Sequence: PROTON (s2pul)
Solvent: cdcl3
Data collected on: Aug 14 2018
Temp. 25.0 C / 298.1 K
Operator: Chemler
Relax. delay 1.000 sec
Pulse 45.0 degrees
Acq. time 2.560 sec
Width 6399.0 Hz
16 repetitions
OBSERVE H1, 399.9389028 MHz
DATA PROCESSING
FT size 32768
Total time 0 min 57 sec

ASB-bromostyrene-buli-crude

Sample Name:

Data Collected on:
nmr400.chem.buffalo.edu-inova400
Archive directory:

Sample directory:

FidFile: CARBON

Pulse Sequence: CARBON (s2pul)
Solvent: cdcl3
Data collected on: Sep 8 2018

Temp. 25.0 C / 298.1 K
Operator: Chemler

Relax. delay 1.000 sec
Pulse 45.0 degrees
Acq. time 1.303 sec
Width 25141.4 Hz
25616 repetitions
OBSERVE C13, 100.5647169 MHz
DECOUPLE H1, 399.9409068 MHz
Power 33 dB
continuously on
WALTZ-16 modulated
DATA PROCESSING
Line broadening 0.5 Hz
FT size 65536
Total time 64 hr, 13 min

Agilent Technologies

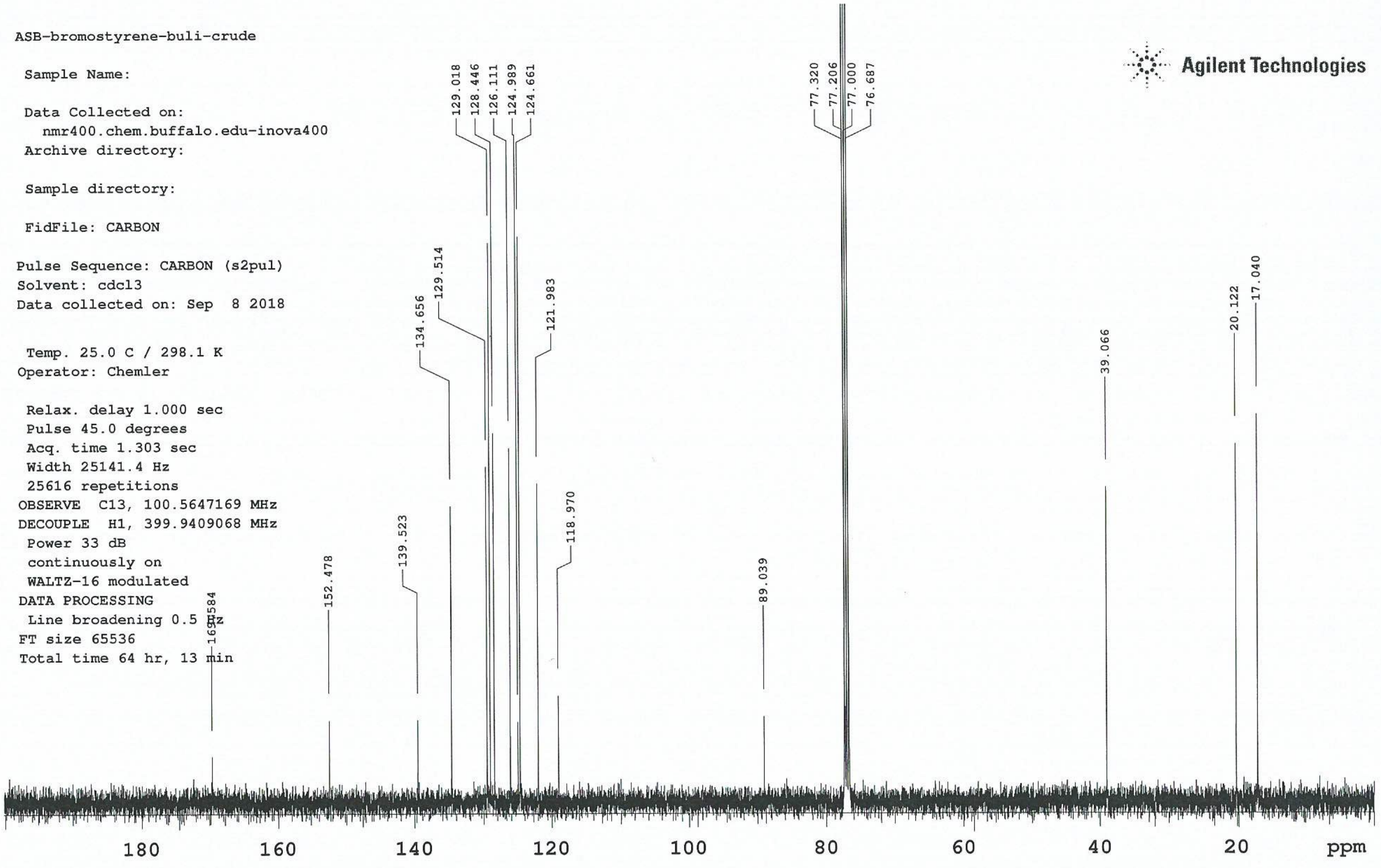

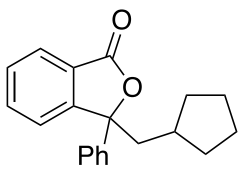

2d

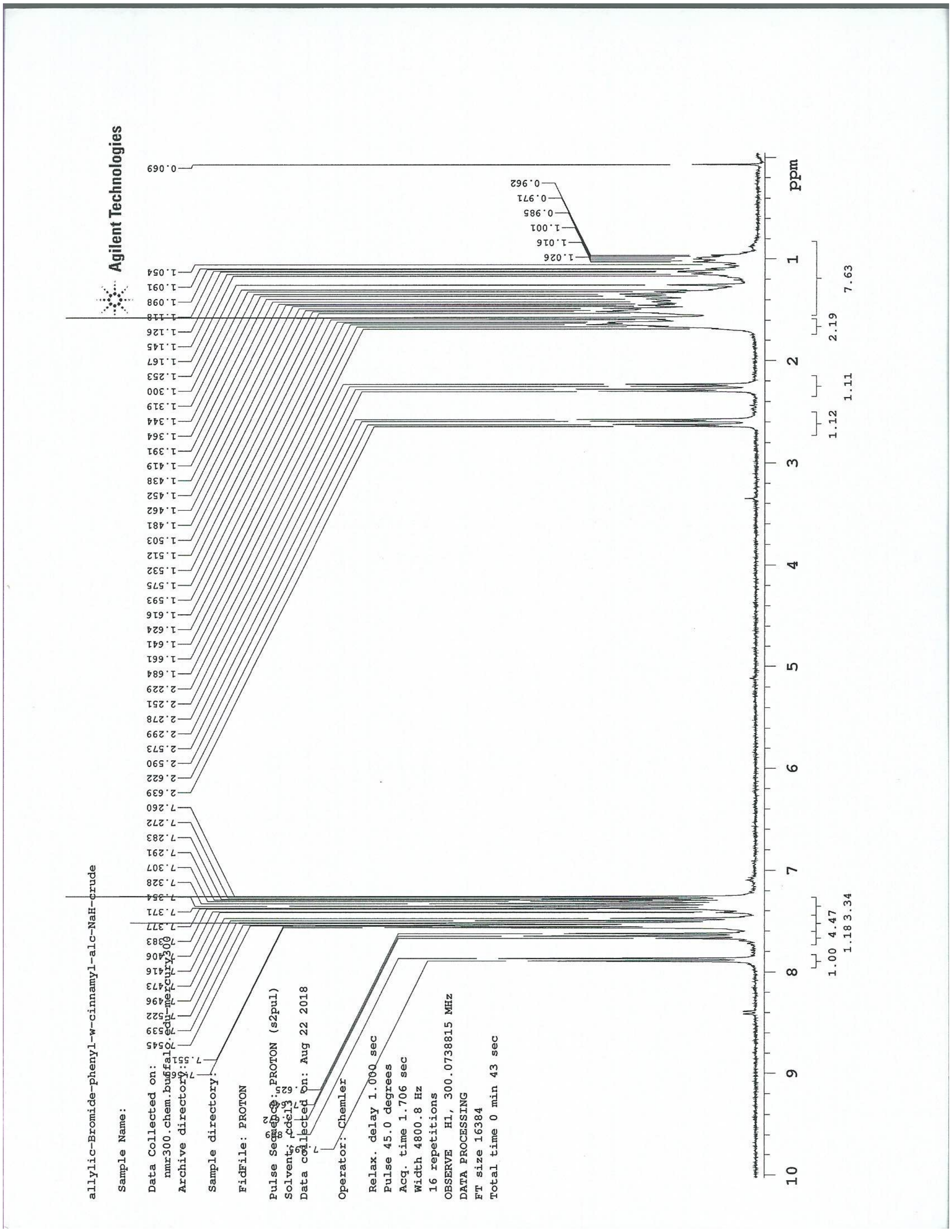
Agilent Technologies
allylic-Bromide-phenyl-w-cinnamyl-alc-NaH-crude
Sample Name:
Data Collected on:
nmr300.chem.buffalo.edu-mercury300
Archive directory:
Sample directory:
FidFile: PROTON
Pulse Sequence: PROTON (s2pul)
Solvent: cdcl3
Data collected on: Aug 22 2018
Operator: Chemler
Relax. delay 1.000 sec
Pulse 45.0 degrees
Acq. time 1.706 sec
Width 4800.8 Hz
16 repetitions
OBSERVE H1, 300.0738815 MHz
DATA PROCESSING
FT size 16384
Total time 0 min 43 sec
ppm

SB_100_filtrate_IPA

Sample Name:

Data Collected on:
nmr300.chem.buffalo.edu-mercury300
Archive directory:

Sample directory:

FidFile: CARBON

Pulse Sequence: CARBON (s2pul)
Solvent: cdcl3
Data collected on: Jul 3 2018

Operator: Chemler

Relax. delay 2.000 sec
Pulse 45.0 degrees
Acq. time 0.868 sec
Width 18867.9 Hz
2376 repetitions
OBSERVE C13, 75.4536377 MHz
DECOUPLE H1, 300.0754430 MHz
Power 37 dB
continuously on
WALTZ-16 modulated
DATA PROCESSING
Line broadening 0.5 Hz
FT size 32768
Total time 79 hr, 55 min

Agilent Technologies

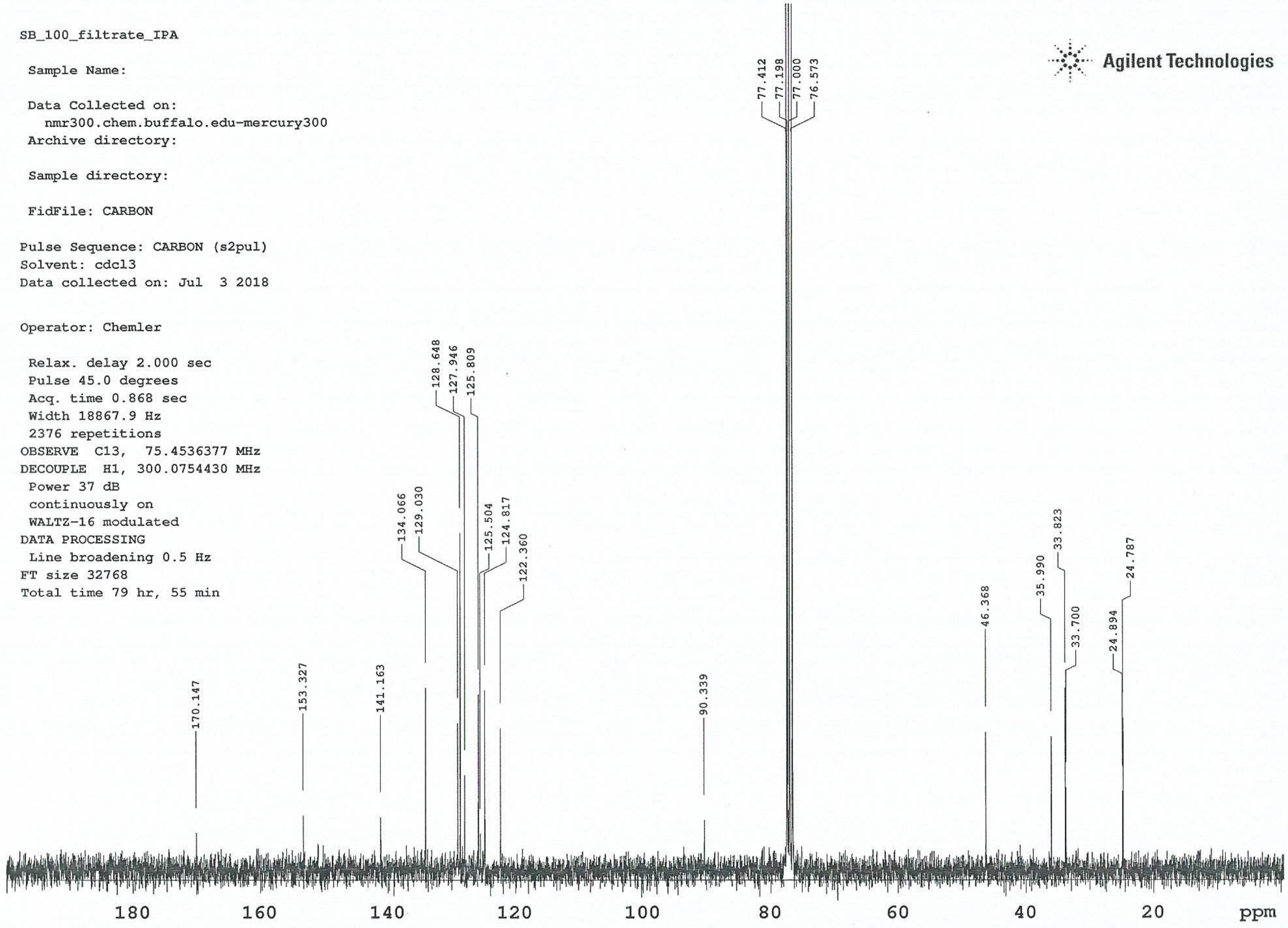

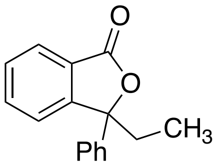
O
O
CH3
Ph

2e

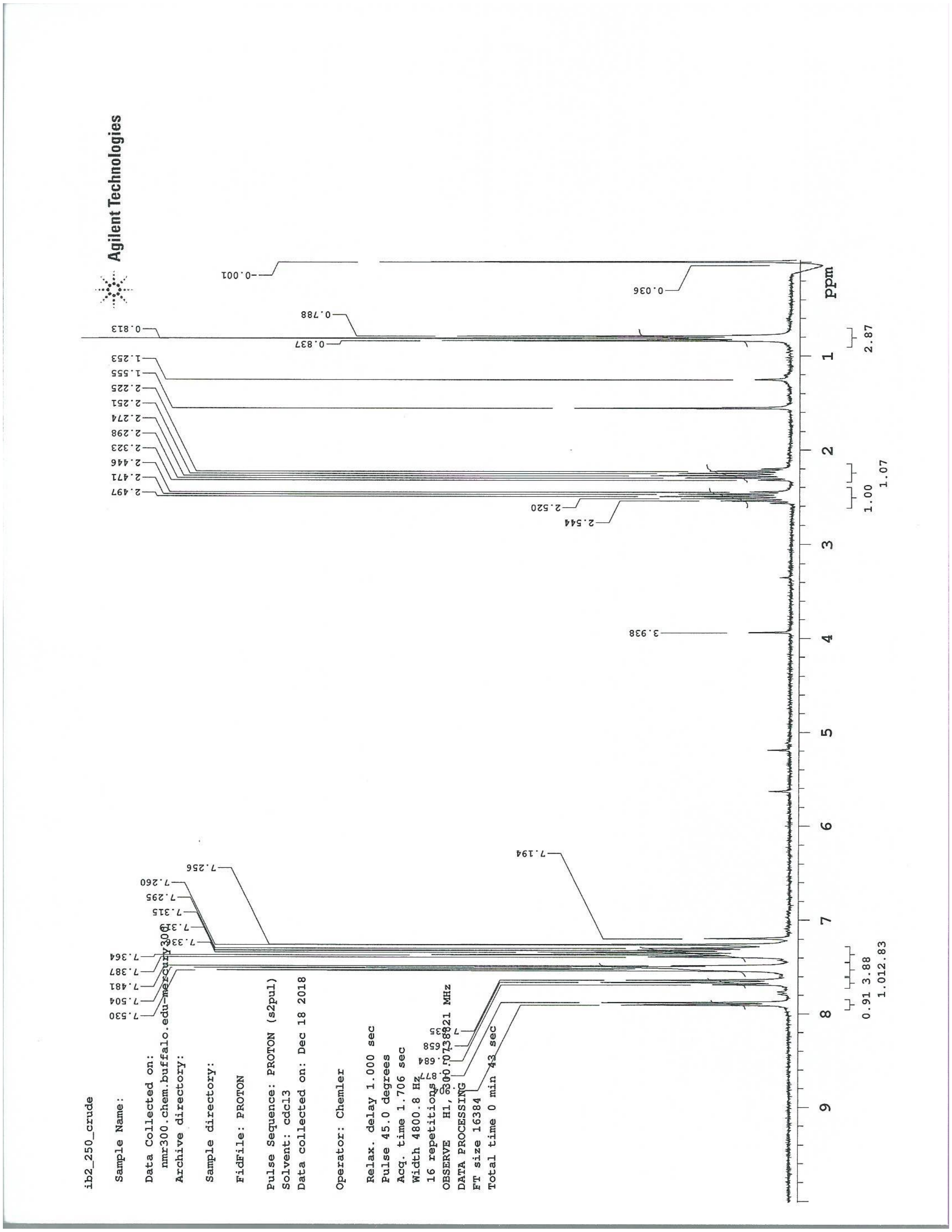
Agilent Technologies
ib2_250_crude
Sample Name:
Data Collected on:
nmr300.chem.buffalo.edu-mercury300
Archive directory:
Sample directory:
FidFile: PROTON
Pulse Sequence: PROTON (s2pul)
Solvent: cdcl3
Data collected on: Dec 18 2018
Operator: Chemler
Relax. delay 1.000 sec
Pulse 45.0 degrees
Acq. time 1.706 sec
Width 4800.8 Hz
16 repetitions
OBSERVE H1, 300.0738821 MHz
DATA PROCESSING
FT size 16384
Total time 0 min 43 sec
ppm

cdk-b2-r572-crude

Sample Name:

Data Collected on:
nmr300.chem.buffalo.edu-mercury300
Archive directory:

Sample directory:

FidFile: CARBON

Pulse Sequence: CARBON (s2pul)
Solvent: cdcl3
Data collected on: Dec 21 2018

Operator: Chemler

Relax. delay 2.000 sec
Pulse 45.0 degrees
Acq. time 0.868 sec
Width 18867.9 Hz
2680 repetitions
OBSERVE C13, 75.4536377 MHz
DECOUPLE H1, 300.0754430 MHz
Power 37 dB
continuously on
WALTZ-16 modulated
DATA PROCESSING
Line broadening 0.5 Hz
FT size 32768
Total time 799 hr, 13 min

Agilent Technologies

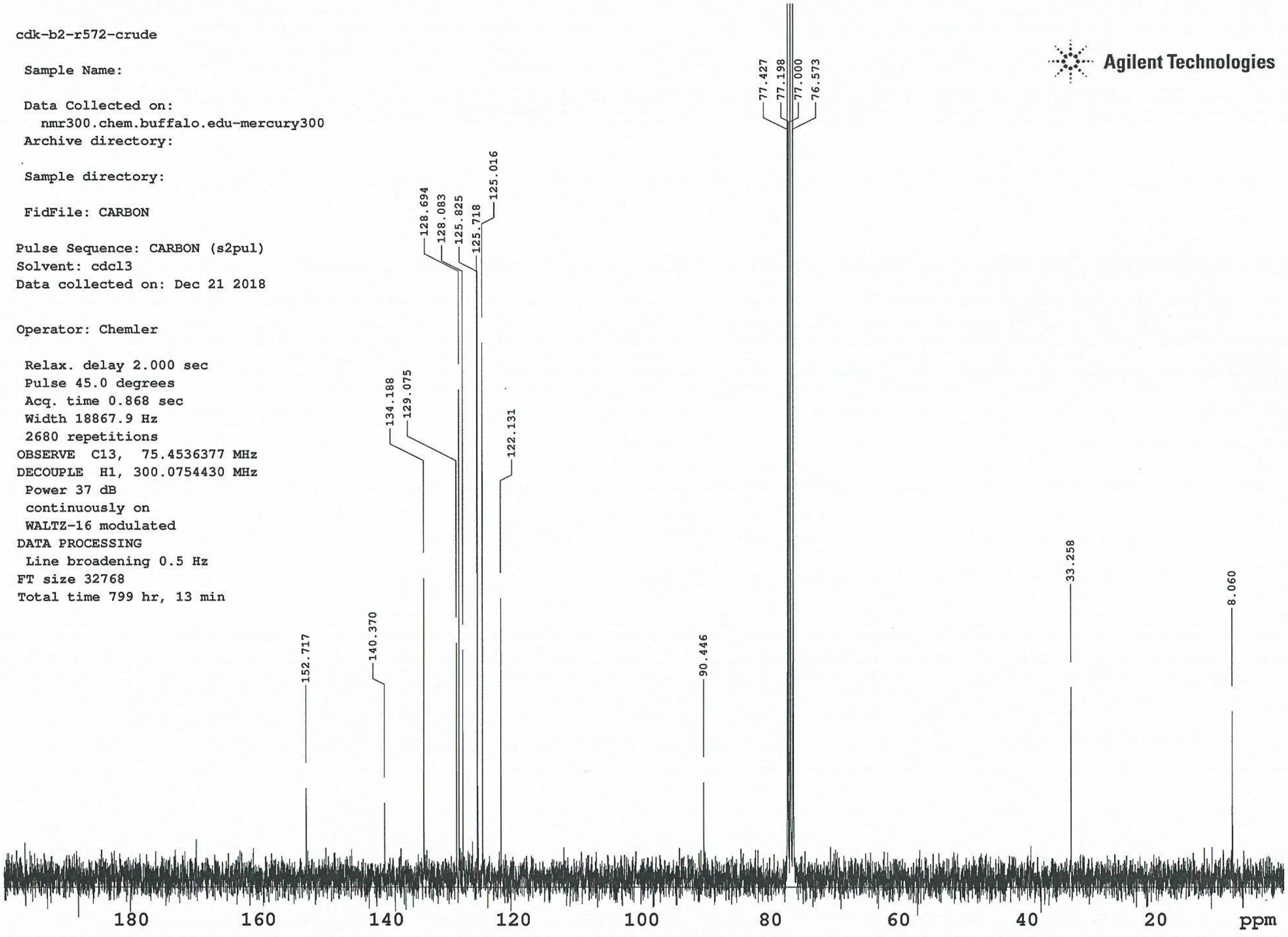

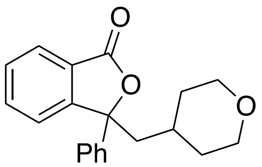

2f

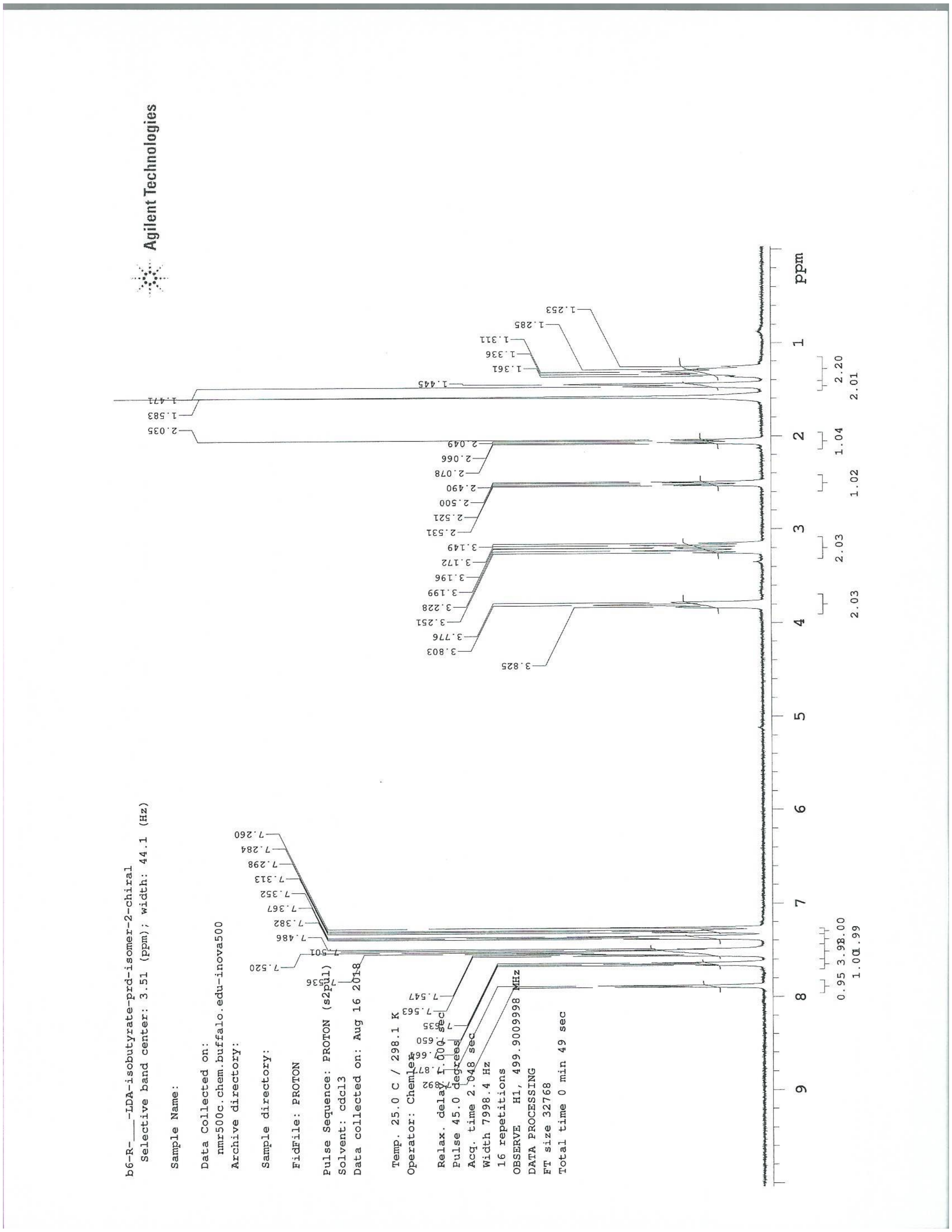

Agilent Technologies
b6-R-___-LDA-isobutyrate-prd-isomer-2-chiral
Selective band center: 3.51 (ppm); width: 44.1 (Hz)
Sample Name:
Data Collected on:
nmr500c.chem.buffalo.edu-inova500
Archive directory:
Sample directory:
FidFile: PROTON
Pulse Sequence: PROTON (s2pul)
Solvent: cdcl3
Data collected on: Aug 16 2018
Temp. 25.0 C / 298.1 K
Operator: Chemlex
Relax. delay 1.000 sec
Pulse 45.0 degrees
Acq. time 2.048 sec
Width 7998.4 Hz
16 repetitions
OBSERVE H1, 499.9009998 MHz
DATA PROCESSING
FT size 32768
Total time 0 min 49 sec
ppm

b6-R-___-LDA-isobutyrate-prd-isomer-2-chiral
Selective band center: 3.51 (ppm); width: 44.1 (Hz)

Sample Name:

Data Collected on:
nmr500c.chem.buffalo.edu-inova500
Archive directory:

Sample directory:

FidFile: CARBON

Pulse Sequence: CARBON (s2pul)
Solvent: cdcl3
Data collected on: Aug 16 2018

Temp. 25.0 C / 298.1 K
Operator: Chemler

Relax. delay 2.000 sec
Pulse 45.0 degrees
Acq. time 1.043 sec
Width 31421.8 Hz
1712 repetitions
OBSERVE C13, 125.7002064 MHz
DECOUPLE H1, 499.9034960 MHz
Power 40 dB
continuously on
WALTZ-16 modulated
DATA PROCESSING
Line broadening 0.5 Hz
FT size 65536
Total time 847 hr, 42 min

Agilent Technologies

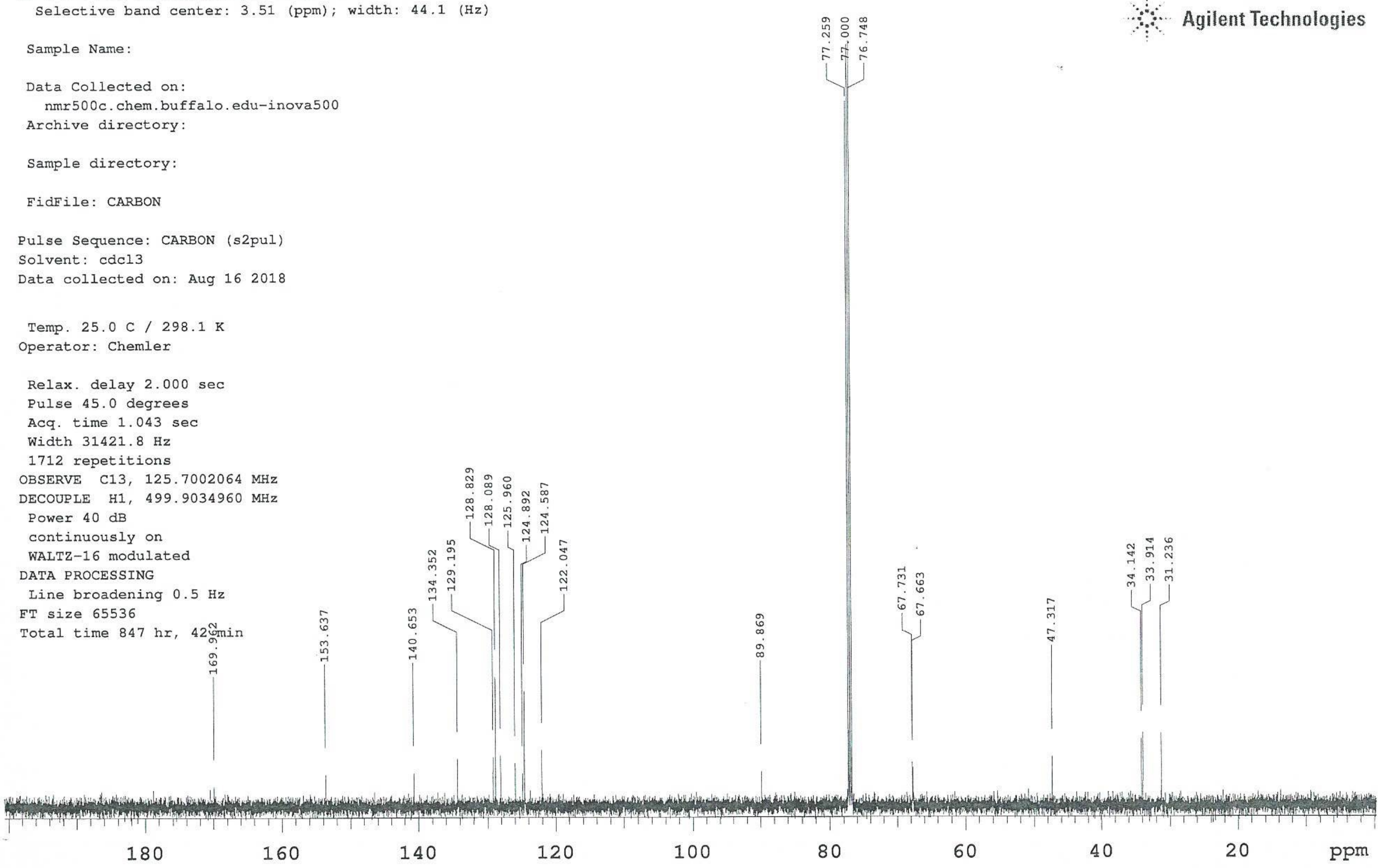

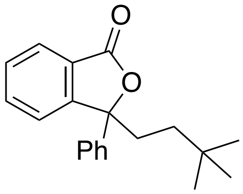
O
O
Ph

2g

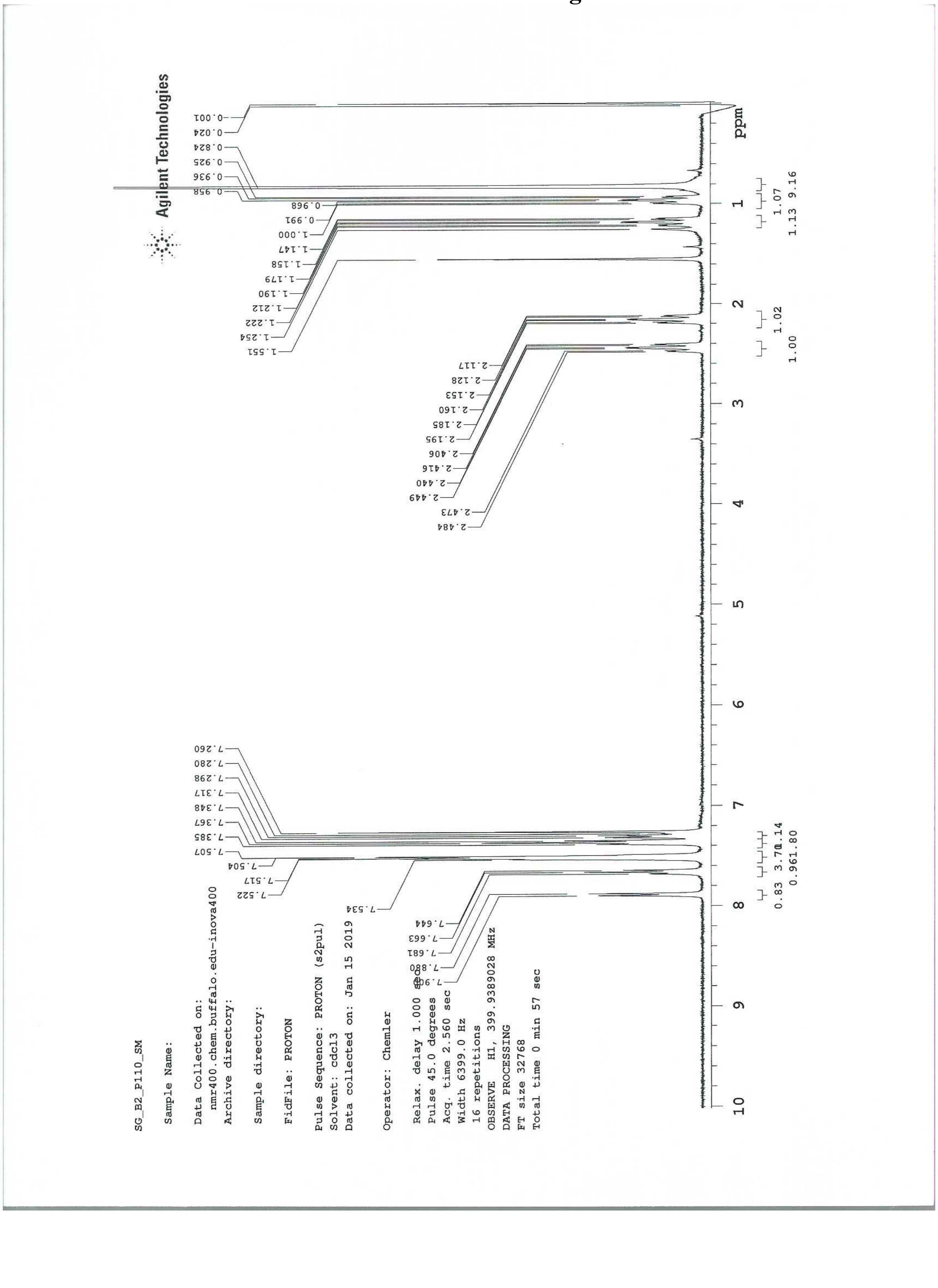
Agilent Technologies
SG_B2_P110_SM
Sample Name:
Data Collected on:
nmr400.chem.buffalo.edu-inova400
Archive directory:
Sample directory:
FidFile: PROTON
Pulse Sequence: PROTON (s2pul)
Solvent: cdcl3
Data collected on: Jan 15 2019
Operator: Chemler
Relax. delay 1.000 sec
Pulse 45.0 degrees
Acq. time 2.560 sec
Width 6399.0 Hz
16 repetitions
OBSERVE H1, 399.9389028 MHz
DATA PROCESSING
FT size 32768
Total time 0 min 57 sec
0.001
0.024
0.824
0.925
0.936
0.958
0.968
0.991
1.000
1.147
1.158
1.179
1.190
1.212
1.222
1.254
1.551
2.117
2.128
2.153
2.160
2.185
2.195
2.406
2.416
2.440
2.449
2.473
2.484
7.260
7.280
7.298
7.317
7.348
7.367
7.385
7.507
7.504
7.517
7.522
7.534
7.644
7.663
7.681
7.880
7.900

II-262 - crude

Sample Name:

Data Collected on:
nmr400.chem.buffalo.edu-inova400
Archive directory:

Sample directory:

FidFile: CARBON

Pulse Sequence: CARBON (s2pul)
Solvent: cdcl3
Data collected on: Jan 17 2019

Operator: Chemler

Relax. delay 2.000 sec
Pulse 45.0 degrees
Acq. time 1.303 sec
Width 25141.4 Hz
12576 repetitions
OBSERVE C13, 100.5647177 MHz
DECOUPLE H1, 399.9409068 MHz
Power 33 dB
continuously on
WALTZ-16 modulated
DATA PROCESSING
Line broadening 0.5 Hz
FT size 65536
Total time 9200 hr, 37 min

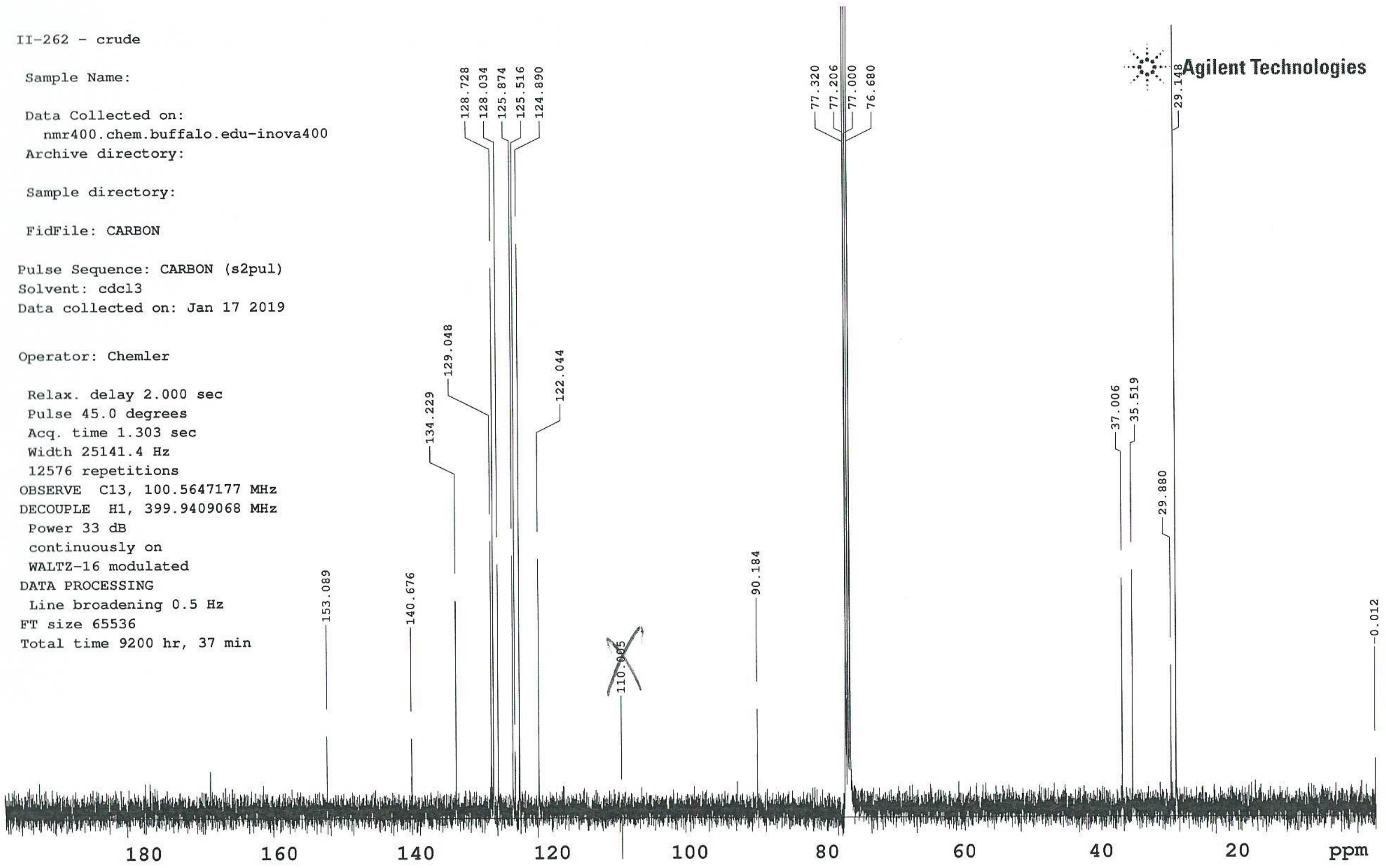

Agilent Technologies

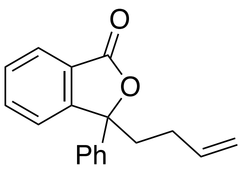

2h

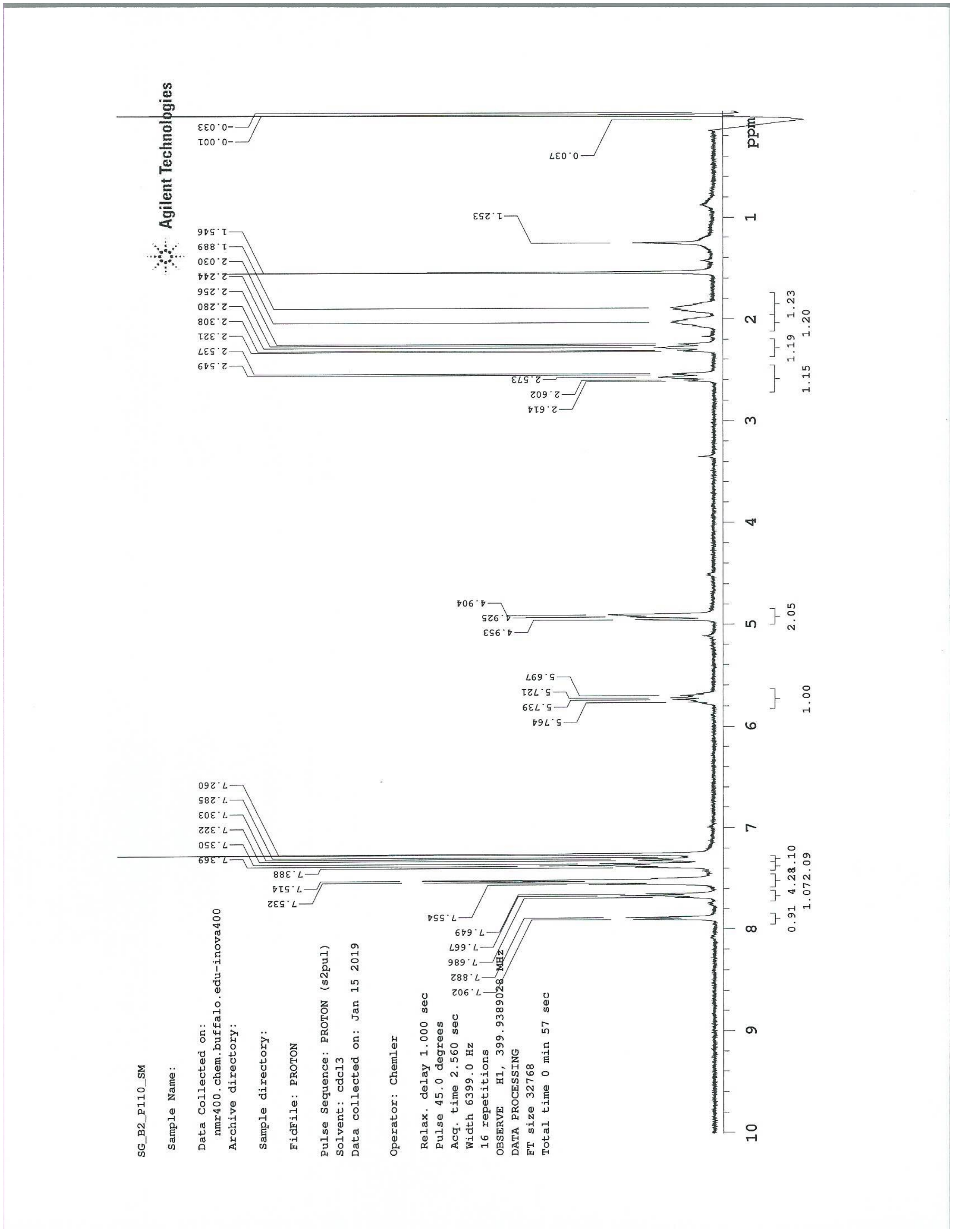

Agilent Technologies
SG_B2_P110_SM
Sample Name:
Data Collected on:
nmr400.chem.buffalo.edu-inova400
Archive directory:
Sample directory:
FidFile: PROTON
Pulse Sequence: PROTON (s2pul)
Solvent: cdcl3
Data collected on: Jan 15 2019
Operator: Chemler
Relax. delay 1.000 sec
Pulse 45.0 degrees
Acq. time 2.560 sec
Width 6399.0 Hz
16 repetitions
OBSERVE H1, 399.9389028 MHz
DATA PROCESSING
FT size 32768
Total time 0 min 57 sec
ppm

ASB-B___-benzothiazolesubstrate-achiral-crude

Sample Name:

Data Collected on:
nmr400.chem.buffalo.edu-inova400
Archive directory:

Sample directory:

FidFile: CARBON

Pulse Sequence: CARBON (s2pul)
Solvent: cdcl3
Data collected on: Jan 21 2019

Operator: Chemler

Relax. delay 2.000 sec
Pulse 45.0 degrees
Acq. time 1.303 sec
Width 25141.4 Hz
15592 repetitions
OBSERVE C13, 100.5647177 MHz
DECOUPLE H1, 399.9409068 MHz
Power 33 dB
continuously on
WALTZ-16 modulated
DATA PROCESSING
Line broadening 0.5 Hz
FT size 65536
Total time 9200 hr, 37 min

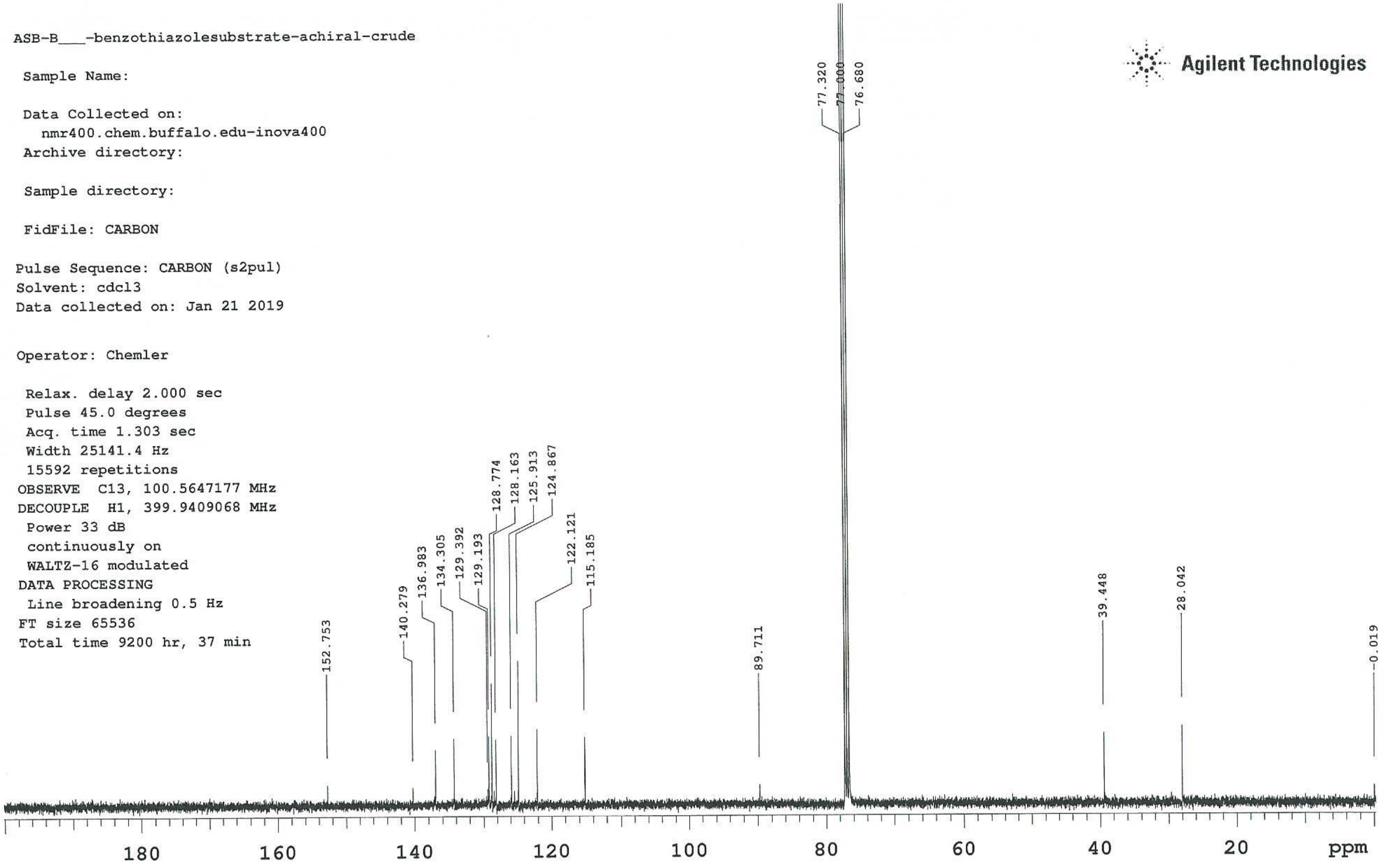

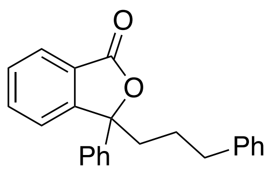
O
O
Ph
Ph

2i

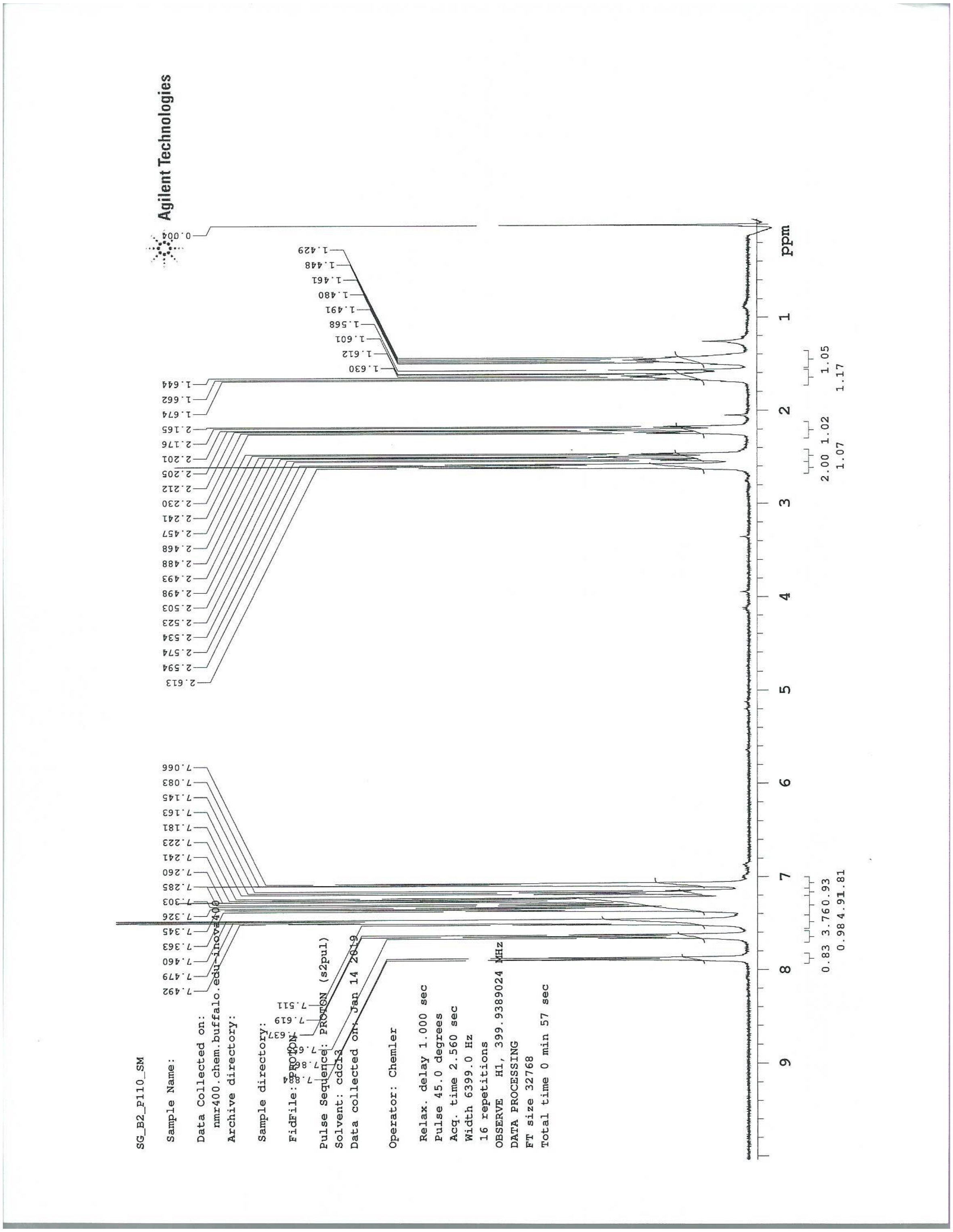
Agilent Technologies
SG_B2_P110_SM
Sample Name:
Data Collected on:
nmr400.chem.buffalo.edu-inova400
Archive directory:
Sample directory:
FidFile: PROTON
Pulse Sequence: PROTON (s2pul)
Solvent: cdcl3
Data collected on: Jan 14 2019
Operator: Chemler
Relax. delay 1.000 sec
Pulse 45.0 degrees
Acq. time 2.560 sec
Width 6399.0 Hz
16 repetitions
OBSERVE H1, 399.9389024 MHz
DATA PROCESSING
FT size 32768
Total time 0 min 57 sec
ppm

SG_B2_P110_SM

Sample Name:

Data Collected on:
nmr400.chem.buffalo.edu-inova400
Archive directory:

Sample directory:

FidFile: CARBON

Pulse Sequence: CARBON (s2pul)
Solvent: cdcl3
Data collected on: Jan 15 2019

Operator: Chemler

Relax. delay 2.000 sec
Pulse 45.0 degrees
Acq. time 1.303 sec
Width 25141.4 Hz
9768 repetitions
OBSERVE C13, 100.5647192 MHz
DECOUPLE H1, 399.9409068 MHz
Power 33 dB
continuously on
WALTZ-16 modulated
DATA PROCESSING
Line broadening 0.5 Hz
FT size 65536
Total time 9200 hr, 37 min

Agilent Technologies

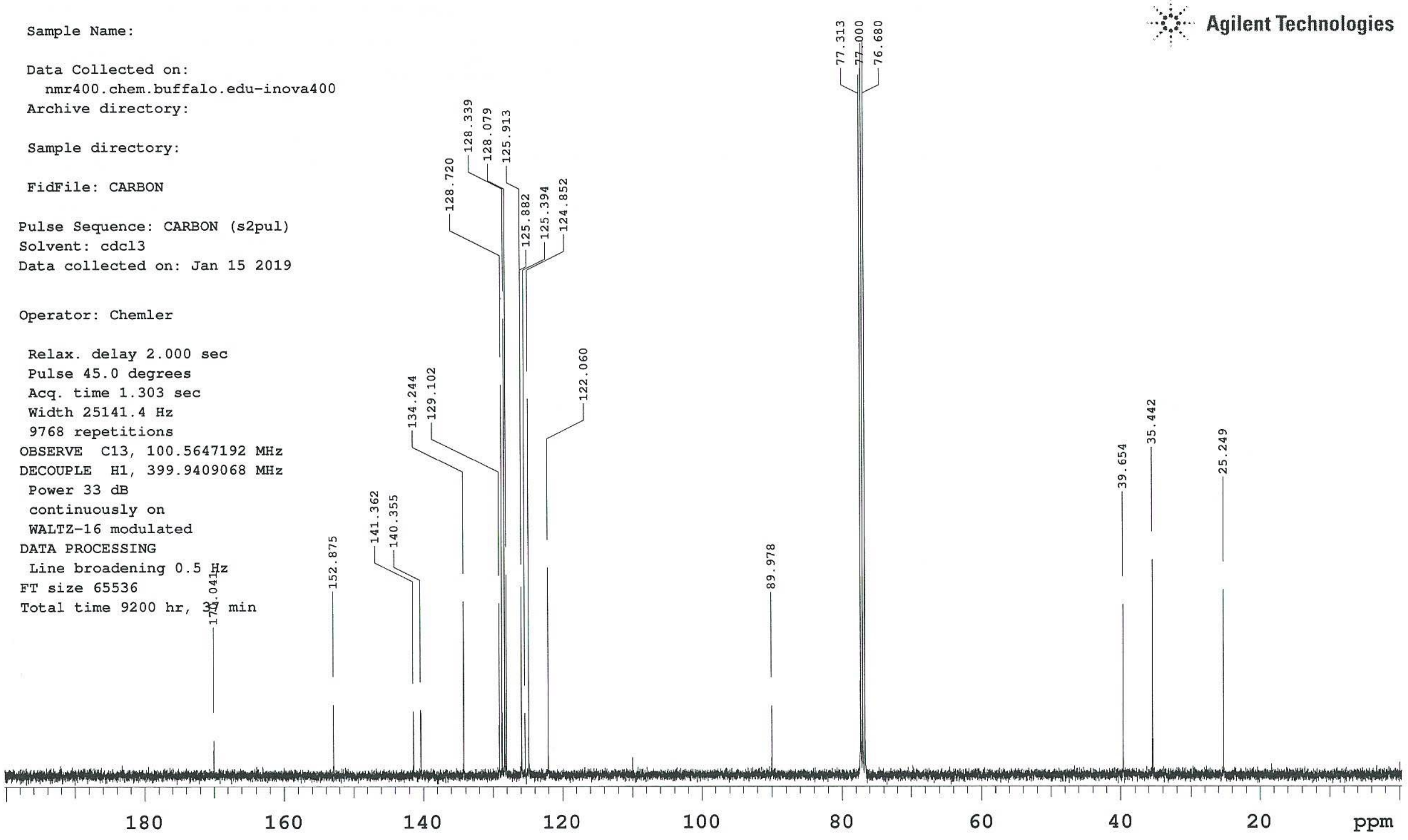

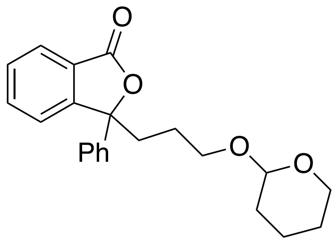

2j

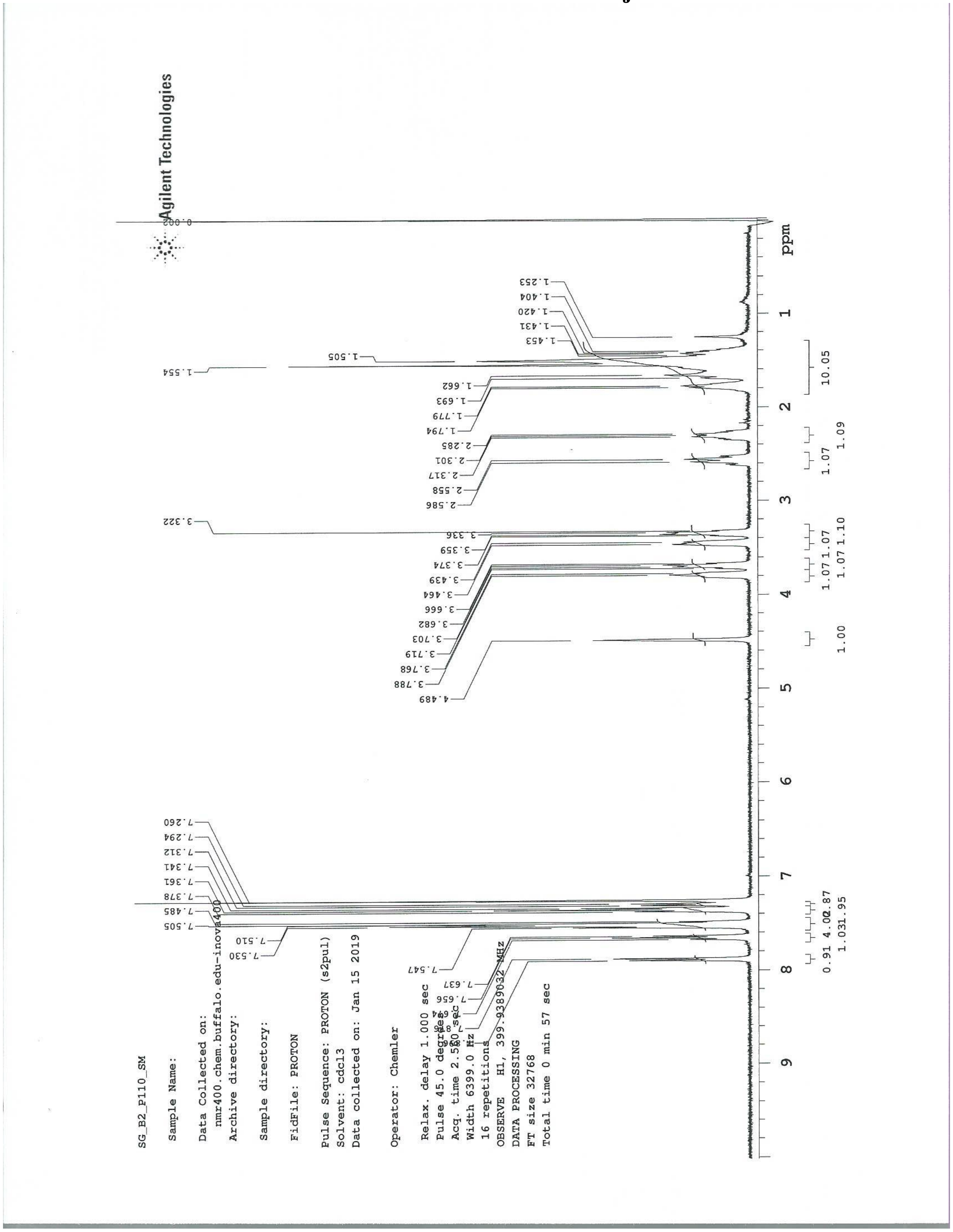
SG_B2_P110_SM
Sample Name:
Data Collected on:
nmr400.chem.buffalo.edu-inova400
Archive directory:
Sample directory:
FidFile: PROTON
Pulse Sequence: PROTON (s2pul)
Solvent: cdcl3
Data collected on: Jan 15 2019
Operator: Chemler
Relax. delay 1.000 sec
Pulse 45.0 degrees
Acq. time 2.560 sec
Width 6399.0 Hz
16 repetitions
OBSERVE H1, 399.9389032 MHz
DATA PROCESSING
FT size 32768
Total time 0 min 57 sec
Agilent Technologies
ppm

II-259 - f.42-48

Sample Name:

Data Collected on:
nmr400.chem.buffalo.edu-inova400
Archive directory:

Sample directory:

FidFile: CARBON

Pulse Sequence: CARBON (s2pul)
Solvent: cdcl3
Data collected on: Jan 18 2019

Operator: Chemler

Relax. delay 2.000 sec
Pulse 45.0 degrees
Acq. time 1.303 sec
Width 25141.4 Hz
14128 repetitions
OBSERVE C13, 100.5647184 MHz
DECOUPLE H1, 399.9409068 MHz
Power 33 dB
continuously on
WALTZ-16 modulated
DATA PROCESSING
Line broadening 0.5 Hz
FT size 65536
Total time 92006 hr, 14 min

Agilent Technologies

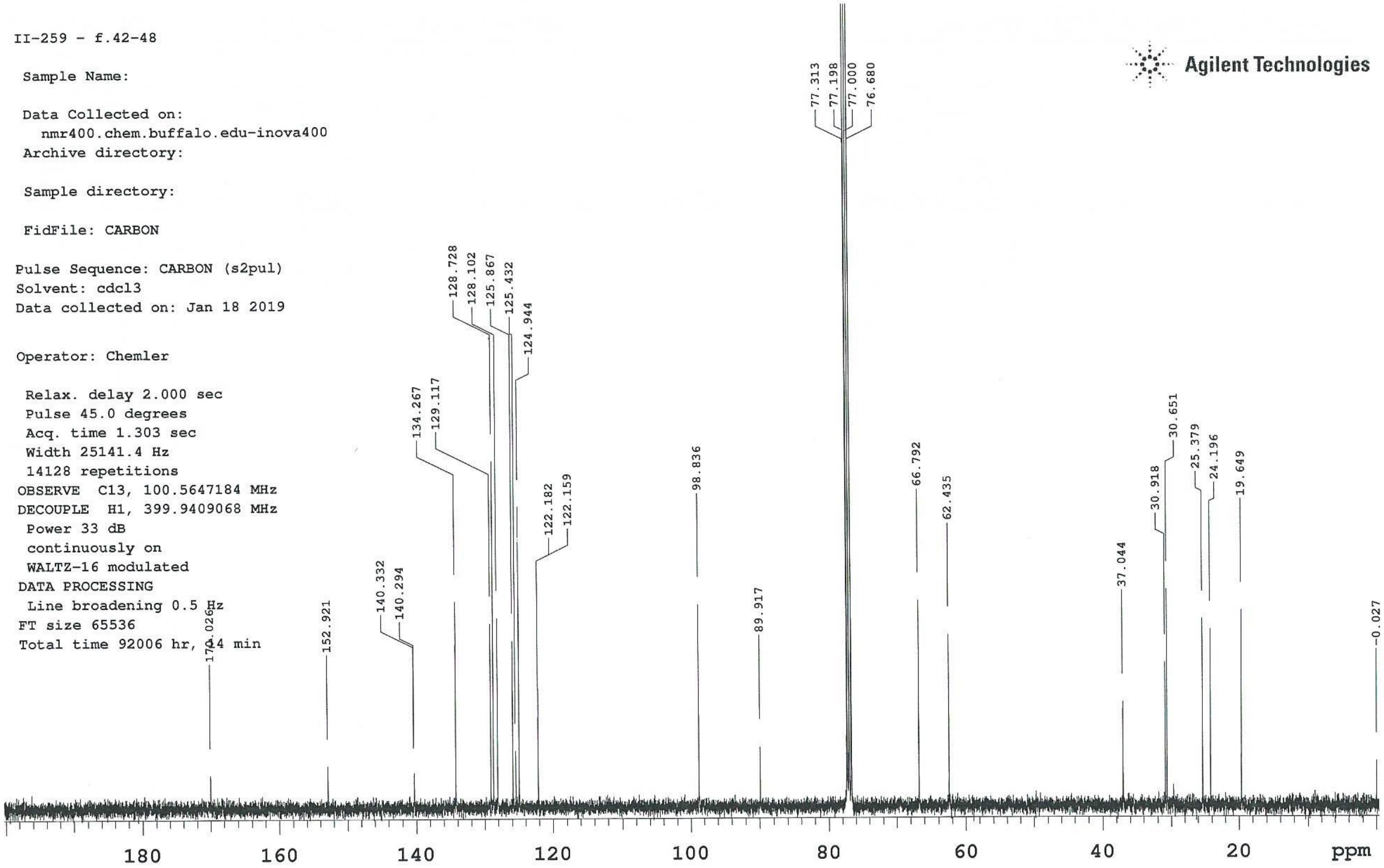

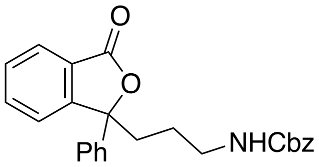
O
O
Ph
NHCbz

2k

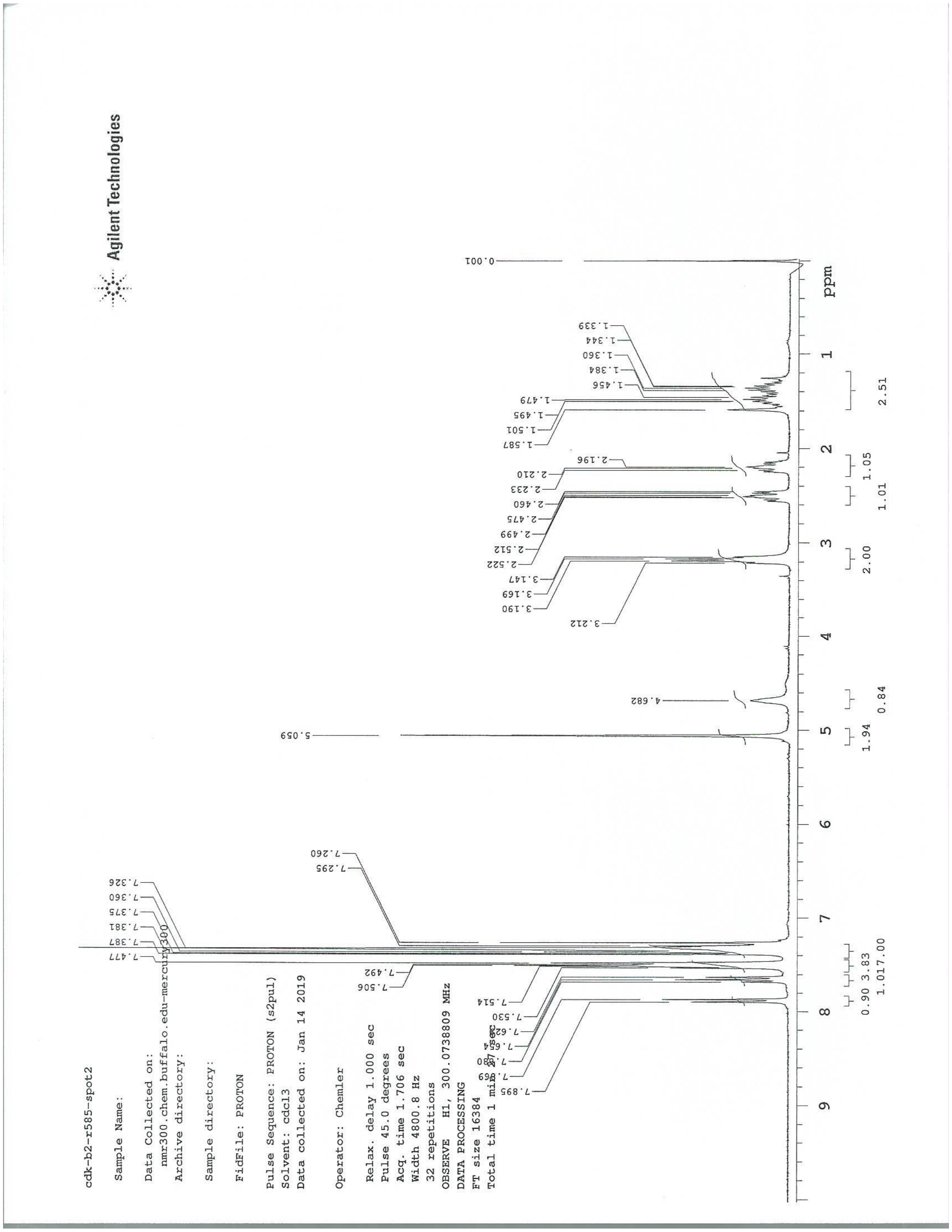
Agilent Technologies
cdk-b2-r585-spot2
Sample Name:
Data Collected on:
nmr300.chem.buffalo.edu-mercury300
Archive directory:
Sample directory:
FidFile: PROTON
Pulse Sequence: PROTON (s2pul)
Solvent: cdcl3
Data collected on: Jan 14 2019
Operator: Chemler
Relax. delay 1.000 sec
Pulse 45.0 degrees
Acq. time 1.706 sec
Width 4800.8 Hz
32 repetitions
OBSERVE H1, 300.0738809 MHz
DATA PROCESSING
FT size 16384
ppm

ASB-B___-TBAFdeprotected-Benzothiazolediol

Sample Name:

Data Collected on:
nmr300.chem.buffalo.edu-mercury300
Archive directory:

Sample directory:

FidFile: CARBON

Pulse Sequence: CARBON (s2pul)
Solvent: cdcl3
Data collected on: Jan 15 2019

Operator: Chemler

Relax. delay 2.000 sec
Pulse 45.0 degrees
Acq. time 0.868 sec
Width 18867.9 Hz
11152 repetitions
OBSERVE C13, 75.4536377 MHz
DECOUPLE H1, 300.0754430 MHz
Power 37 dB
continuously on
WALTZ-16 modulated
DATA PROCESSING
Line broadening 0.5 Hz
FT size 32768
Total time 799227 hr, 21 min

Agilent Technologies

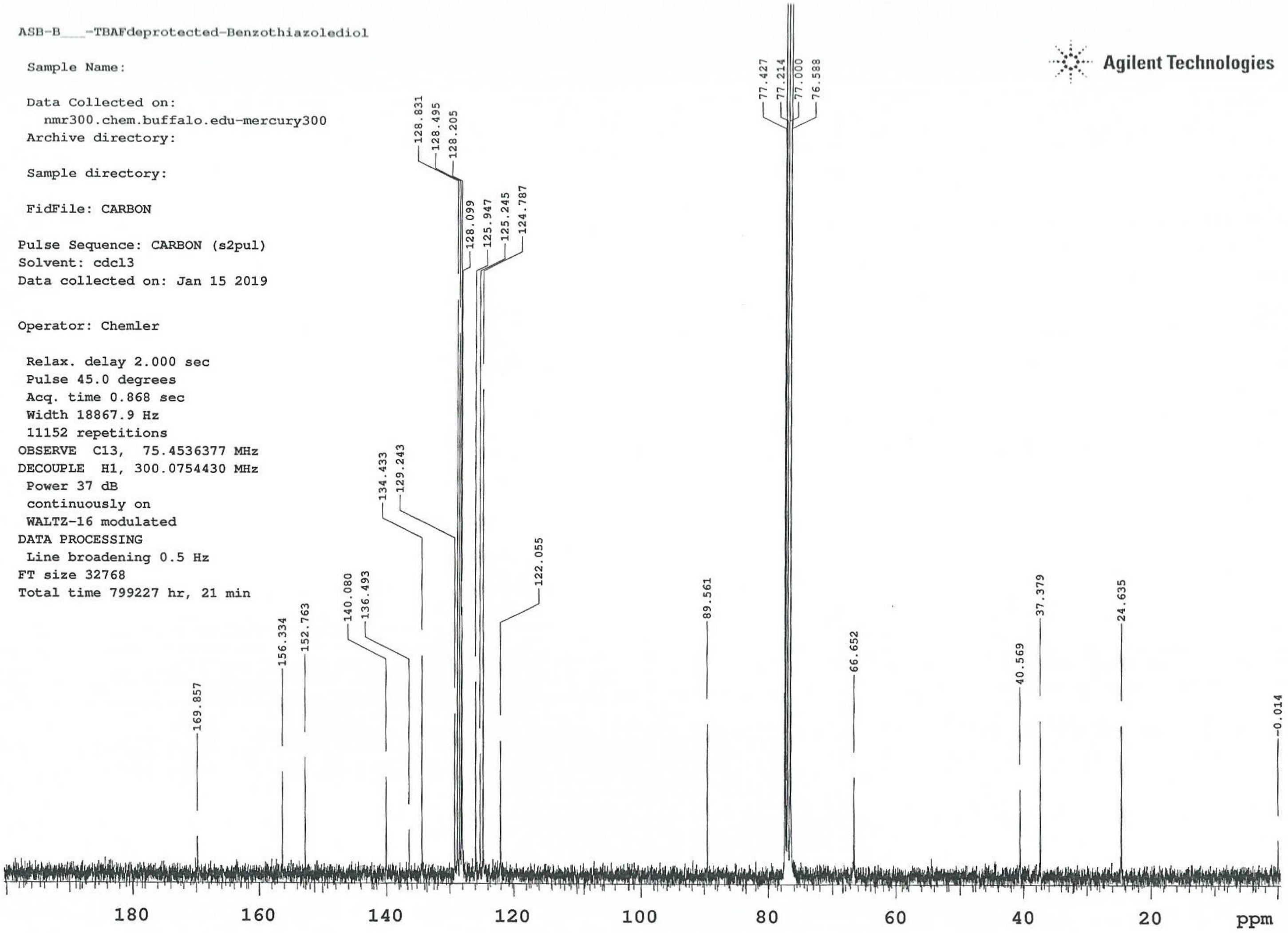

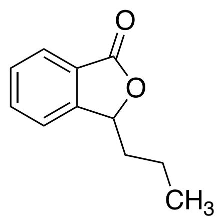
O
O
CH3

2l

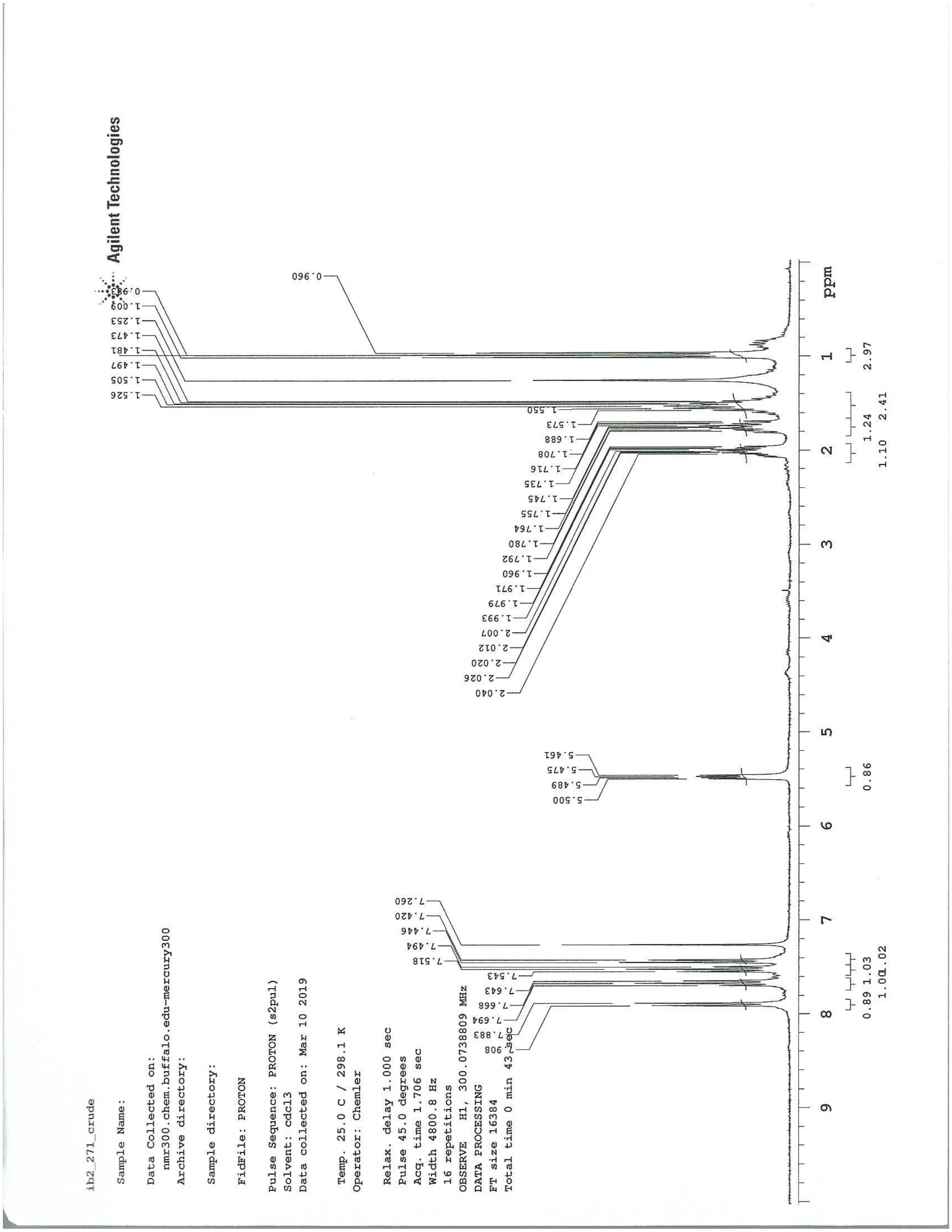
Agilent Technologies
ib2_271_crude
Sample Name:
Data Collected on:
nmr300.chem.buffalo.edu-mercury300
Archive directory:
Sample directory:
FidFile: PROTON
Pulse Sequence: PROTON (s2pul)
Solvent: cdcl3
Data collected on: Mar 10 2019
Temp. 25.0 C / 298.1 K
Operator: Chemler
Relax. delay 1.000 sec
Pulse 45.0 degrees
Acq. time 1.706 sec
Width 4800.8 Hz
16 repetitions
OBSERVE H1, 300.0738809 MHz
DATA PROCESSING
FT size 16384
Total time 0 min 43 sec
ppm

ib2_271_crude

Sample Name:

Data Collected on:
nmr300.chem.buffalo.edu-mercury300
Archive directory:

Sample directory:

FidFile: CARBON

Pulse Sequence: CARBON (s2pul)
Solvent: cdcl3
Data collected on: Mar 9 2019

Temp. 25.0 C / 298.1 K
Operator: Chemler

Relax. delay 2.000 sec
Pulse 45.0 degrees
Acq. time 0.868 sec
Width 18867.9 Hz
12944 repetitions
OBSERVE C13, 75.4536377 MHz
DECOUPLE H1, 300.0754430 MHz
Power 37 dB
continuously on
WALTZ-16 modulated
DATA PROCESSING
Line broadening 0.5 Hz
FT size 32768
Total time 79922 hr, 45 min

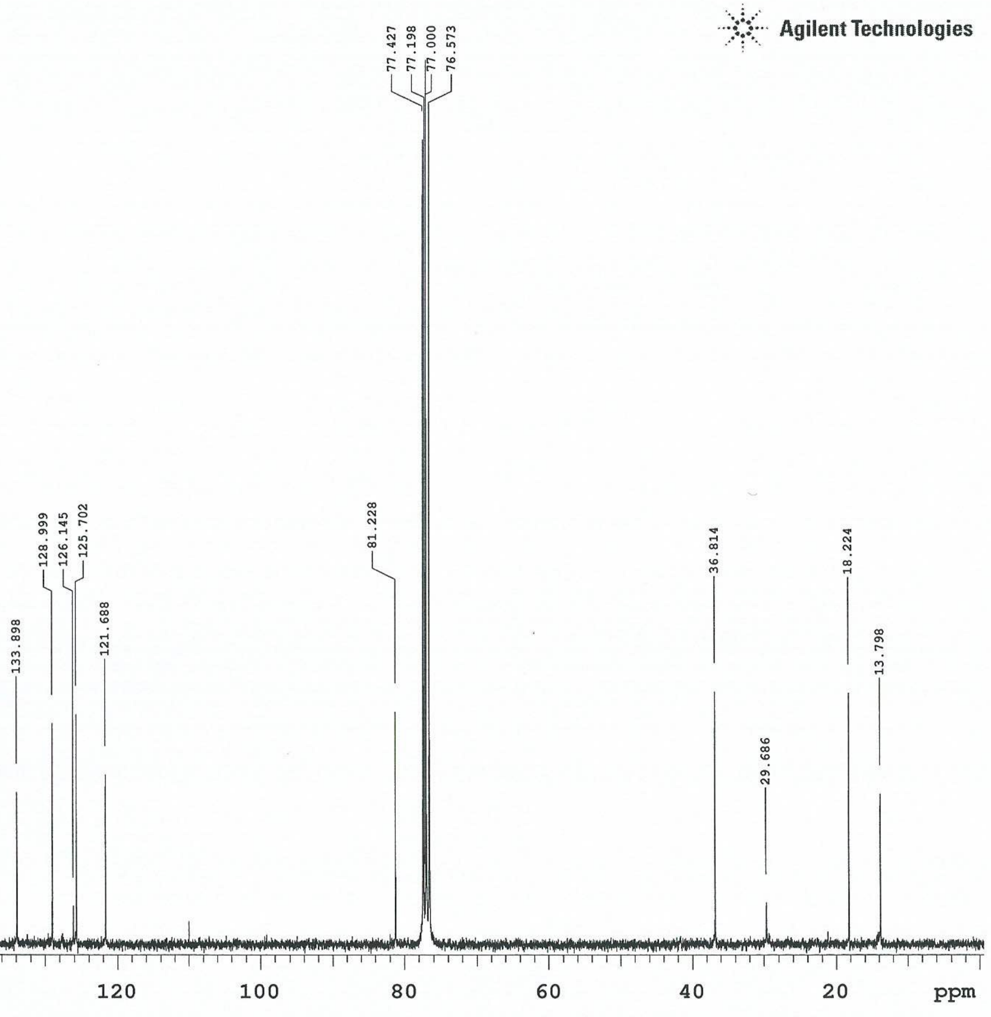

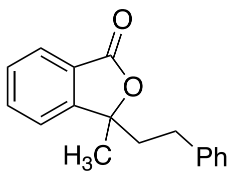
O
O
H_3C
Ph

2m

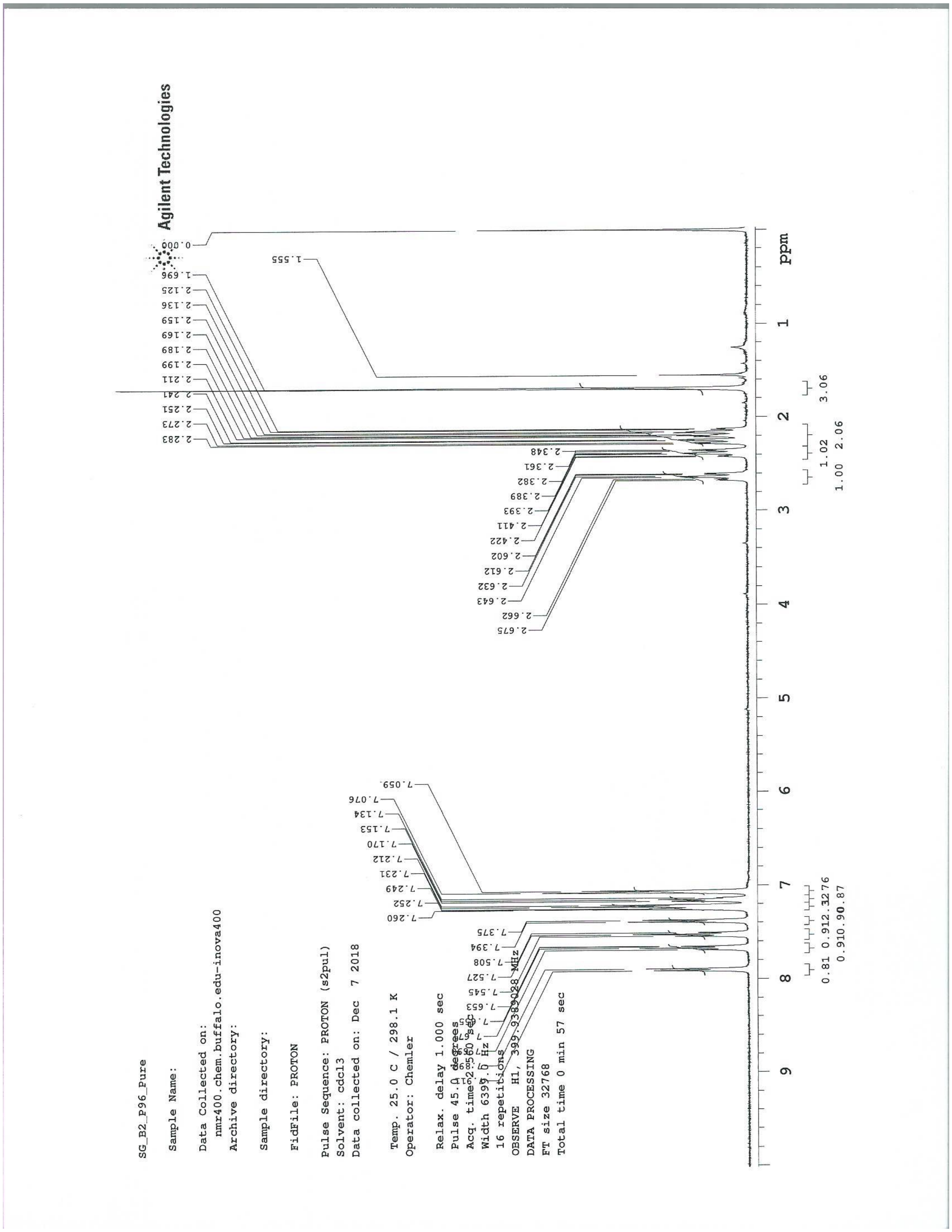
Agilent Technologies
SG_B2_P96_Pure
Sample Name:
Data Collected on:
nmr400.chem.buffalo.edu-inova400
Archive directory:
Sample directory:
FidFile: PROTON
Pulse Sequence: PROTON (s2pul)
Solvent: cdcl3
Data collected on: Dec 7 2018
Temp. 25.0 C / 298.1 K
Operator: Chemler
Relax. delay 1.000 sec
Width 6399.0 Hz
16 repetitions
DATA PROCESSING
FT size 32768
Total time 0 min 57 sec
ppm

SG_B2_P96_Pure

Sample Name:

Data Collected on:
nmr400.chem.buffalo.edu-inova400
Archive directory:

Sample directory:

FidFile: CARBON

Pulse Sequence: CARBON (s2pul)
Solvent: cdcl3
Data collected on: Dec 7 2018

Temp. 25.0 C / 298.1 K
Operator: Chemler

Relax. delay 2.000 sec
Pulse 45.0 degrees
Acq. time 1.303 sec
Width 25141.4 Hz
12592 repetitions
OBSERVE C13, 100.5647169 MHz
DECOUPLE H1, 399.9409068 MHz
Power 33 dB
continuously on
WALTZ-16 modulated
DATA PROCESSING
Line broadening 0.5 Hz
FT size 65536
Total time 920 hr, 3 min

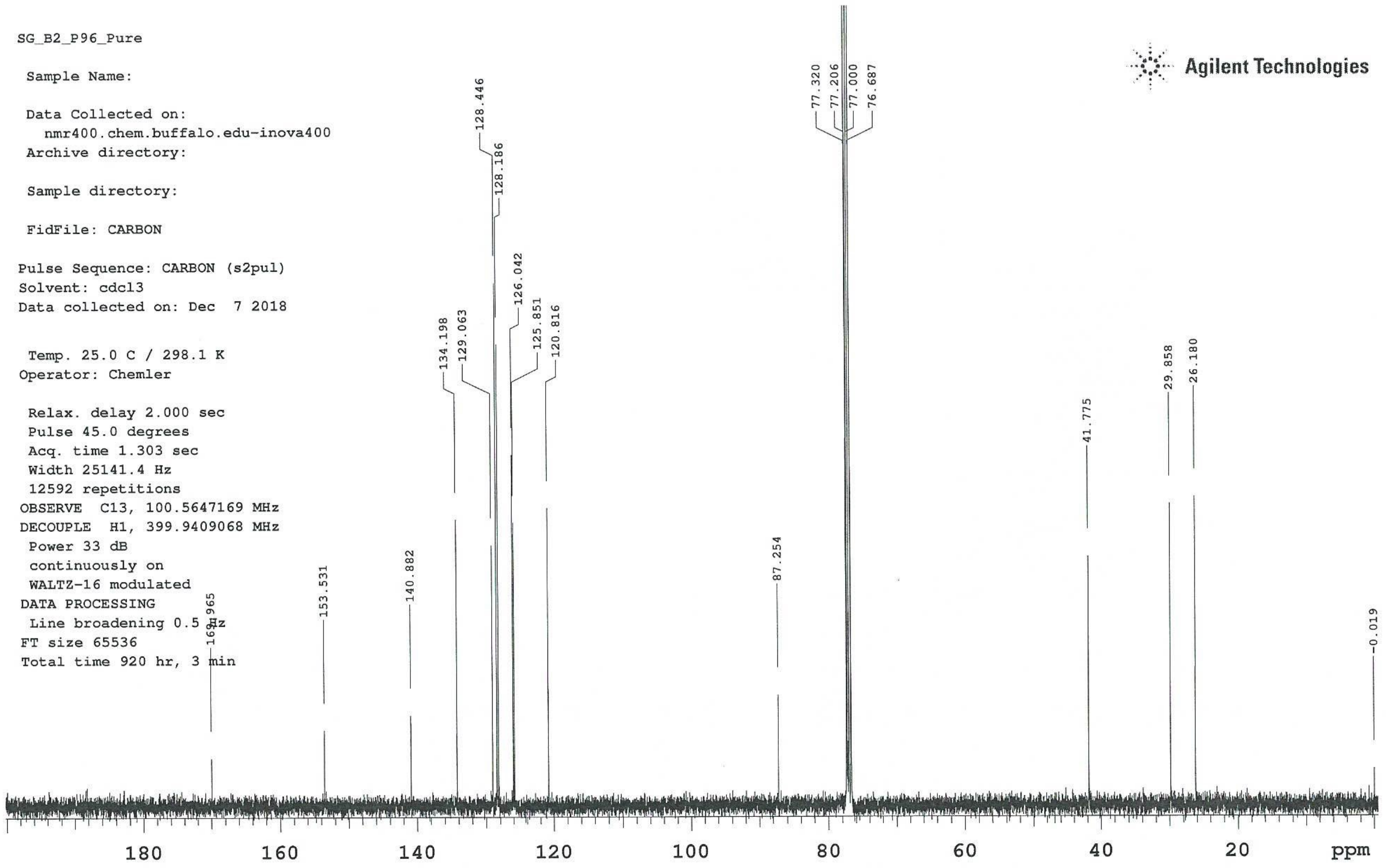

Agilent Technologies

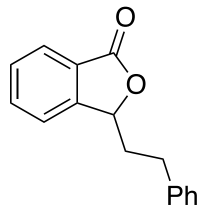

2n

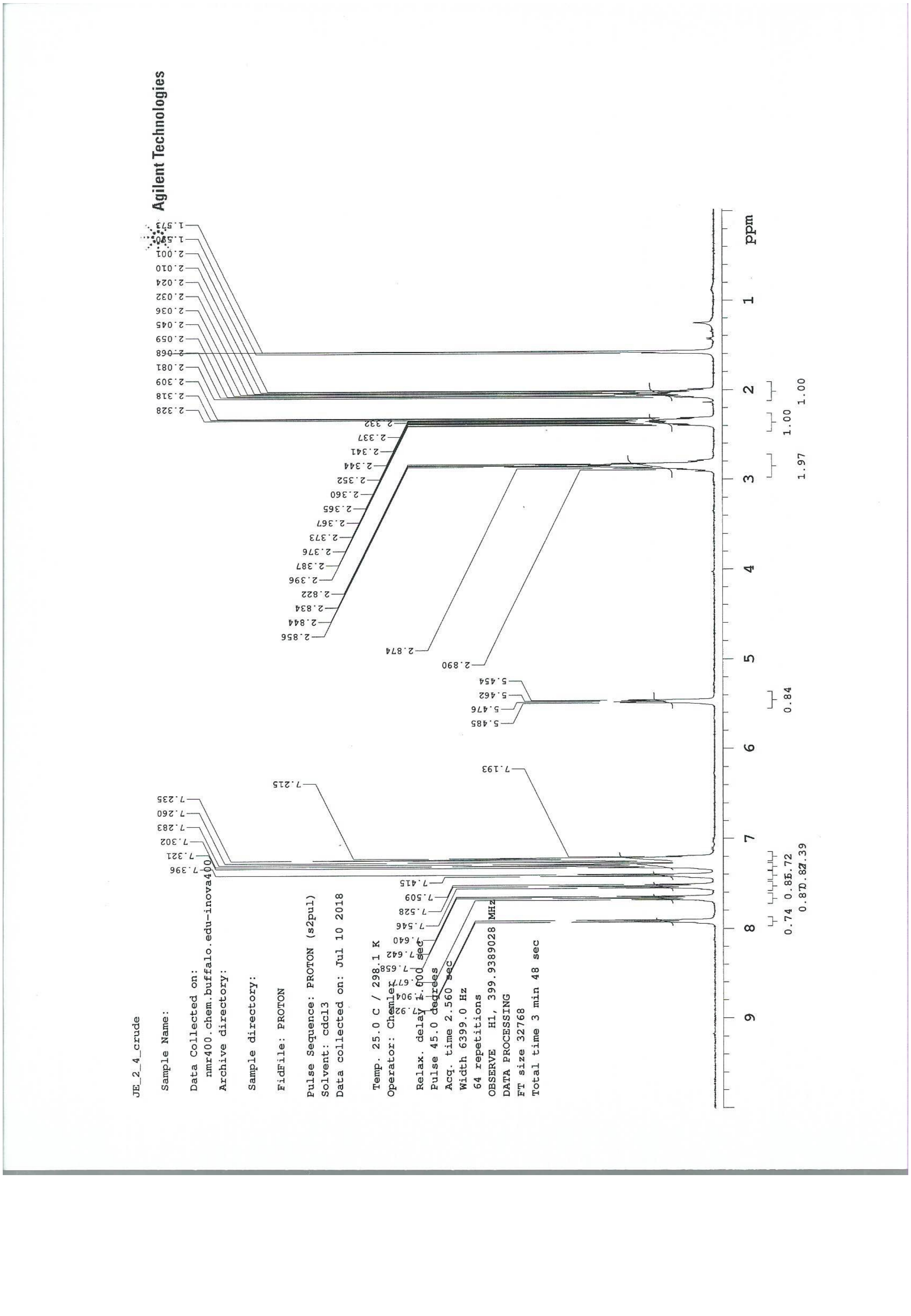
Agilent Technologies
JE_2_4_crude
Sample Name:
Data Collected on:
nmr400.chem.buffalo.edu-inova400
Archive directory:
Sample directory:
FidFile: PROTON
Pulse Sequence: PROTON (s2pul)
Solvent: cdcl3
Data collected on: Jul 10 2018
Temp. 25.0 C / 298.1 K
Operator: Chemler
Relax. delay 1.000 sec
Pulse 45.0 degrees
Acq. time 2.560 sec
Width 6399.0 Hz
64 repetitions
OBSERVE H1, 399.9389028 MHz
DATA PROCESSING
FT size 32768
Total time 3 min 48 sec
ppm

ASB-B___-spot2-eliiminationgrouptransfer

Sample Name:

Data Collected on:
nmr400.chem.buffalo.edu-inova400
Archive directory:

Sample directory:

FidFile: CARBON

Pulse Sequence: CARBON (s2pul)
Solvent: cdcl3
Data collected on: Aug 12 2018

Temp. 25.0 C / 298.1 K
Operator: Chemler

Relax. delay 2.000 sec
Pulse 45.0 degrees
Acq. time 1.303 sec
Width 25141.4 Hz
4024 repetitions
OBSERVE C13, 100.5647169 MHz
DECOUPLE H1, 399.9409068 MHz
Power 33 dB
continuously on
WALTZ-16 modulated
DATA PROCESSING
Line broadening 0.5 Hz
FT size 65536
Total time 920 hr, 3 min

Agilent Technologies

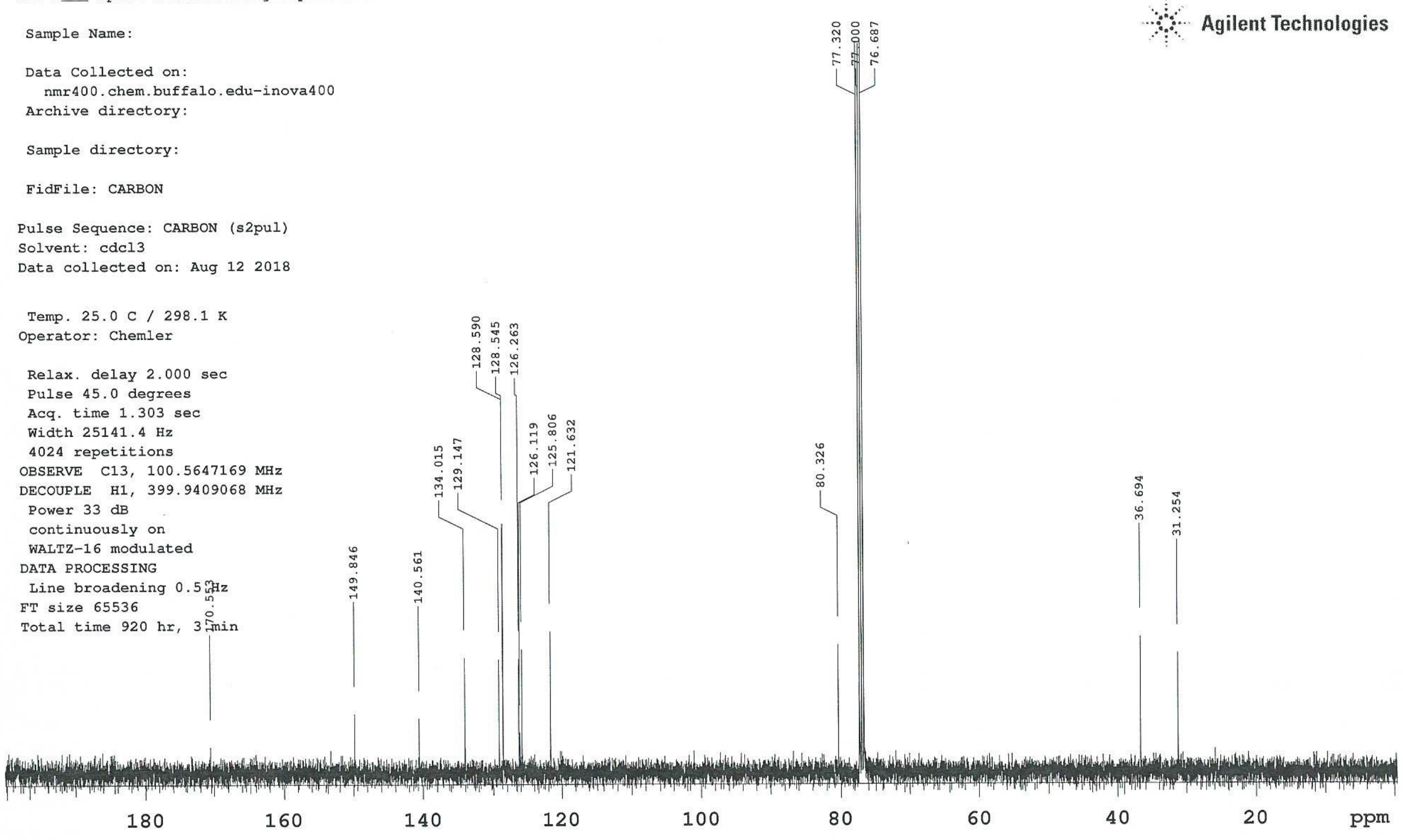

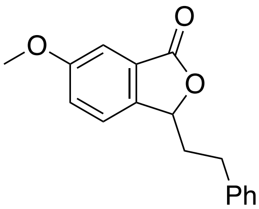

2o

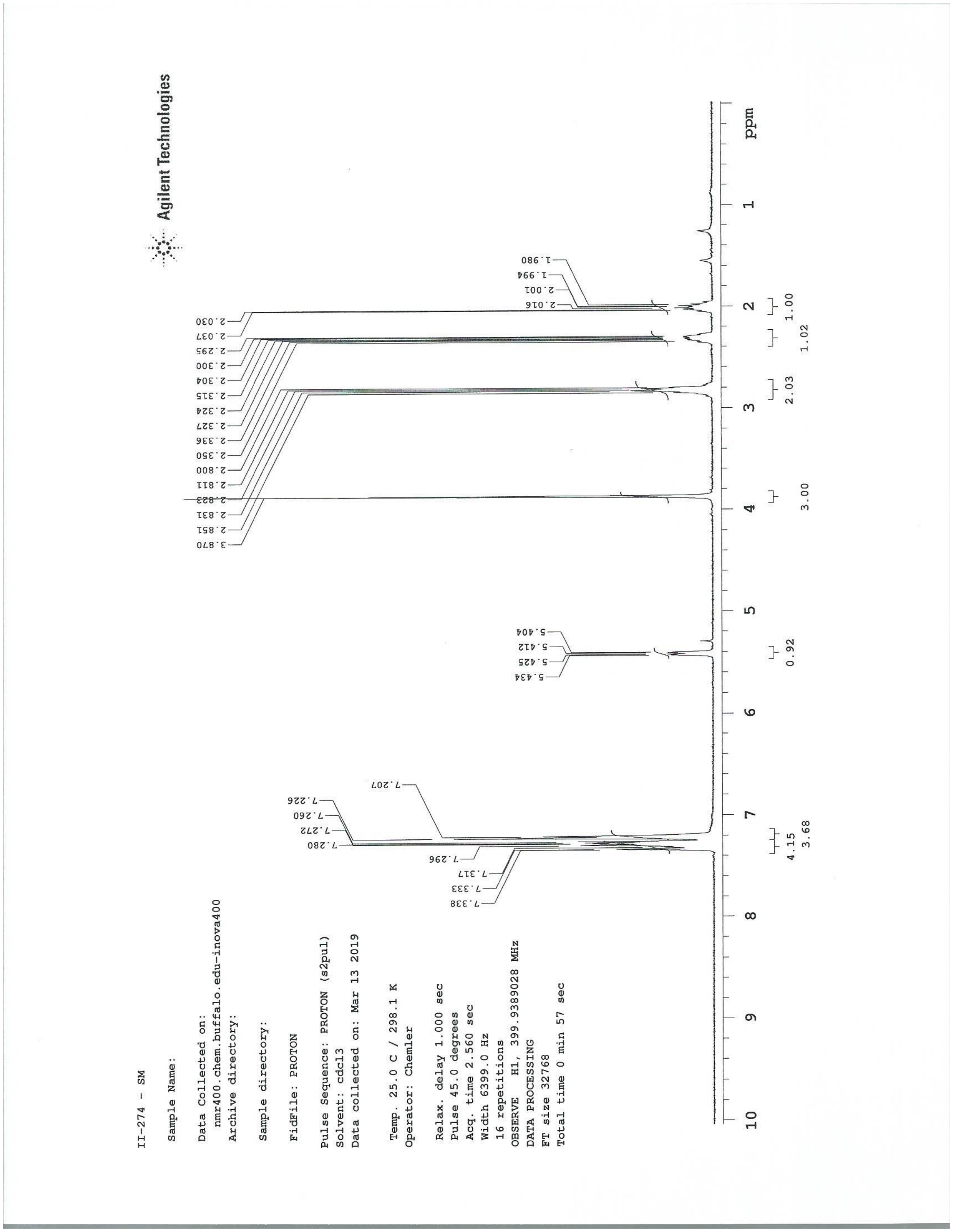
II-274 - SM
Sample Name:
Data Collected on:
nmr400.chem.buffalo.edu-inova400
Archive directory:
Sample directory:
FidFile: PROTON
Pulse Sequence: PROTON (s2pul)
Solvent: cdcl3
Data collected on: Mar 13 2019
Temp. 25.0 C / 298.1 K
Operator: Chemler
Relax. delay 1.000 sec
Pulse 45.0 degrees
Acq. time 2.560 sec
Width 6399.0 Hz
16 repetitions
OBSERVE H1, 399.9389028 MHz
DATA PROCESSING
FT size 32768
Total time 0 min 57 sec
Agilent Technologies
7.338
7.333
7.317
7.296
7.280
7.272
7.260
7.226
7.207
5.434
5.425
5.412
5.404
3.870
2.851
2.831
2.823
2.811
2.800
2.350
2.336
2.327
2.324
2.315
2.304
2.300
2.295
2.037
2.030
2.016
2.001
1.994
1.980
4.15
3.68
0.92
3.00
2.03
1.02
1.00
ppm
10
9
8
7
6
5
4
3
2
1

cdk-

Sample Name:

Data Collected on:
nmr400.chem.buffalo.edu-inova400
Archive directory:

Sample directory:

FidFile: CARBON

Pulse Sequence: CARBON (s2pul)
Solvent: cdcl3
Data collected on: Mar 15 2019

Temp. 25.0 C / 298.1 K
Operator: Chemler

Relax. delay 2.000 sec
Pulse 45.0 degrees
Acq. time 1.303 sec
Width 25141.4 Hz
11336 repetitions
OBSERVE C13, 100.5647177 MHz
DECOUPLE H1, 399.9409068 MHz
Power 33 dB
continuously on
WALTZ-16 modulated
DATA PROCESSING
Line broadening 0.5 Hz
FT size 65536
Total time 92006 hr, 14 min

Agilent Technologies

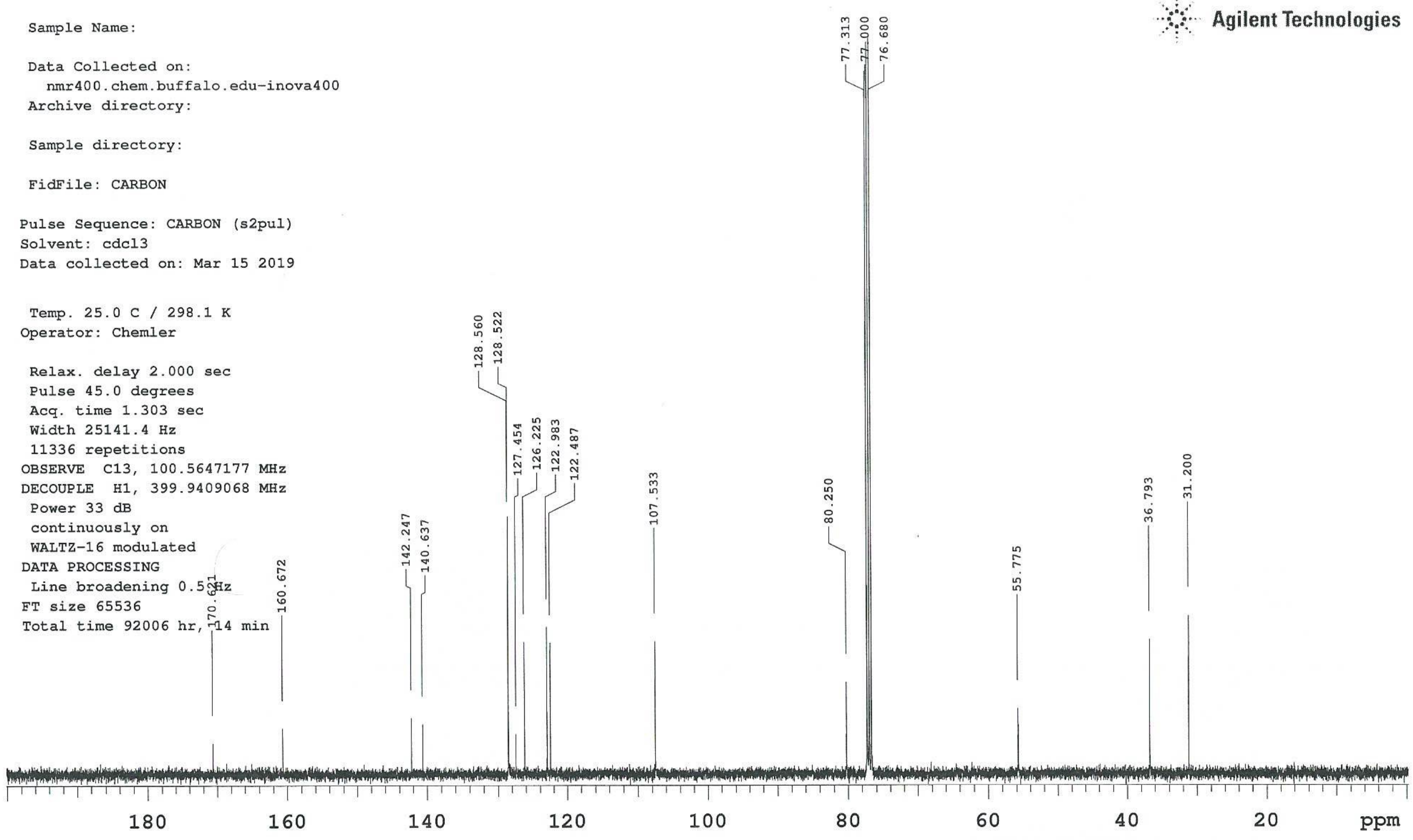

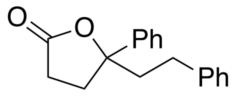
O
O
Ph
Ph

5a

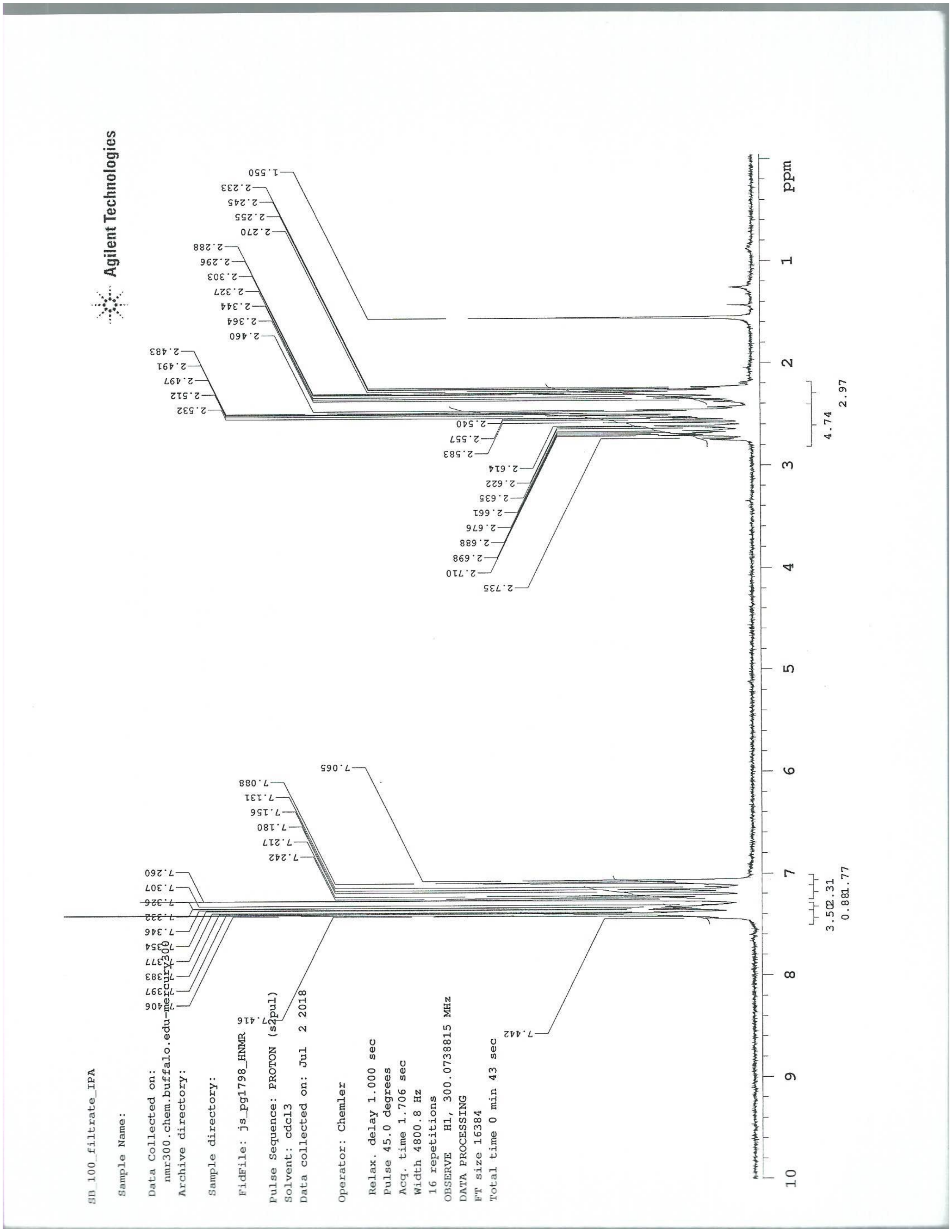
Agilent Technologies
SB_100_filtrate_IPA
Sample Name:
Data Collected on:
nmr300.chem.buffalo.edu-mercury300
Archive directory:
Sample directory:
FidFile: js_pg1798_HNMR
Pulse Sequence: PROTON (s2pul)
Solvent: cdcl3
Data collected on: Jul 2 2018
Operator: Chemler
Relax. delay 1.000 sec
Pulse 45.0 degrees
Acq. time 1.706 sec
Width 4800.8 Hz
16 repetitions
OBSERVE H1, 300.0738815 MHz
DATA PROCESSING
FT size 16384
Total time 0 min 43 sec
ppm

SG_B2_P53_F30

Sample Name:

Data Collected on:
nmr300.chem.buffalo.edu-mercury300
Archive directory:

Sample directory:

FidFile: CARBON

Pulse Sequence: CARBON (s2pul)
Solvent: cdcl3
Data collected on: Jul 11 2018

Operator: Chemler

Relax. delay 2.000 sec
Pulse 45.0 degrees
Acq. time 0.868 sec
Width 18867.9 Hz
1168 repetitions
OBSERVE C13, 75.4536377 MHz
DECOUPLE H1, 300.0754430 MHz
Power 37 dB
continuously on
WALTZ-16 modulated
DATA PROCESSING
Line broadening 0.5 Hz
FT size 32768
Total time 799 hr, 13 min

Agilent Technologies

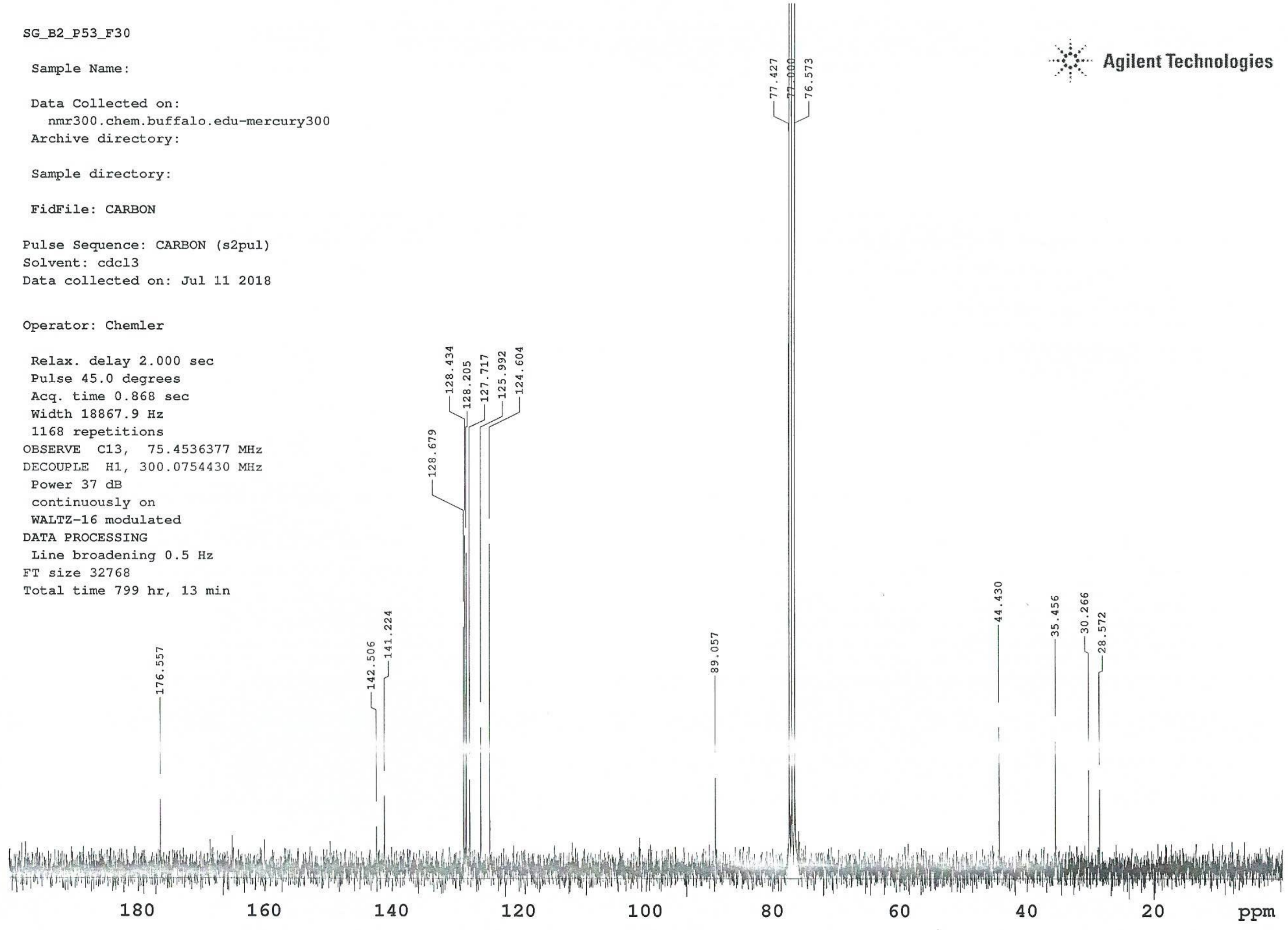

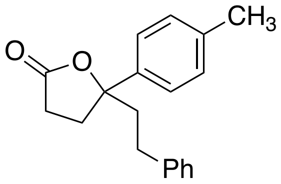
O
O
CH3
Ph

5b

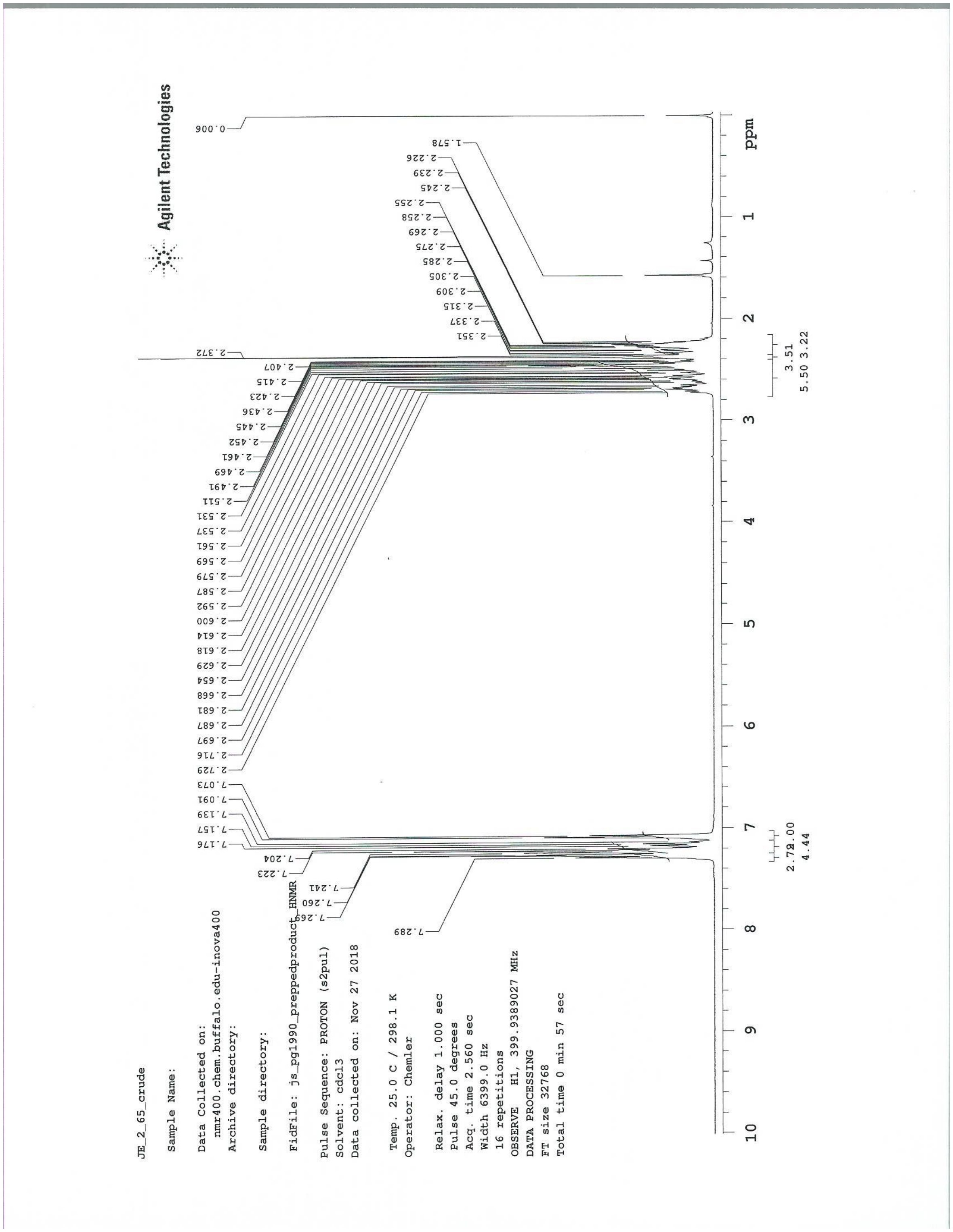
Agilent Technologies
JE_2_65_crude
Sample Name:
Data Collected on:
nmr400.chem.buffalo.edu-inova400
Archive directory:
Sample directory:
FidFile: js_pg1990_preppedproduct_HNMR
Pulse Sequence: PROTON (s2pul)
Solvent: cdcl3
Data collected on: Nov 27 2018
Temp. 25.0 C / 298.1 K
Operator: Chemler
Relax. delay 1.000 sec
Pulse 45.0 degrees
Acq. time 2.560 sec
Width 6399.0 Hz
16 repetitions
OBSERVE H1, 399.9389027 MHz
DATA PROCESSING
FT size 32768
Total time 0 min 57 sec
0.006
1.578
2.226
2.239
2.245
2.255
2.258
2.269
2.275
2.285
2.305
2.309
2.315
2.337
2.351
2.372
2.407
2.415
2.423
2.436
2.445
2.452
2.461
2.469
2.491
2.511
2.531
2.537
2.561
2.569
2.579
2.587
2.592
2.600
2.614
2.618
2.629
2.654
2.668
2.681
2.687
2.697
2.716
2.729
7.073
7.091
7.139
7.157
7.176
7.204
7.223
7.241
7.260
7.269
7.289
3.51
5.50 3.22
2.72
2.00
4.44
ppm
1
2
3
4
5
6
7
8
9
10

JE_2_65_crude

Sample Name:

Data Collected on:
nmr400.chem.buffalo.edu-inova400
Archive directory:

Sample directory:

FidFile: CARBON

Pulse Sequence: CARBON (s2pul)
Solvent: cdcl3
Data collected on: Nov 27 2018

Temp. 25.0 C / 298.1 K
Operator: Chemler

Relax. delay 2.000 sec
Pulse 45.0 degrees
Acq. time 1.303 sec
Width 25141.4 Hz
424 repetitions
OBSERVE C13, 100.5647192 MHz
DECOUPLE H1, 399.9409068 MHz
Power 33 dB
continuously on
WALTZ-16 modulated
DATA PROCESSING
Line broadening 0.5 Hz
FT size 65536
Total time 920 hr, 3 min

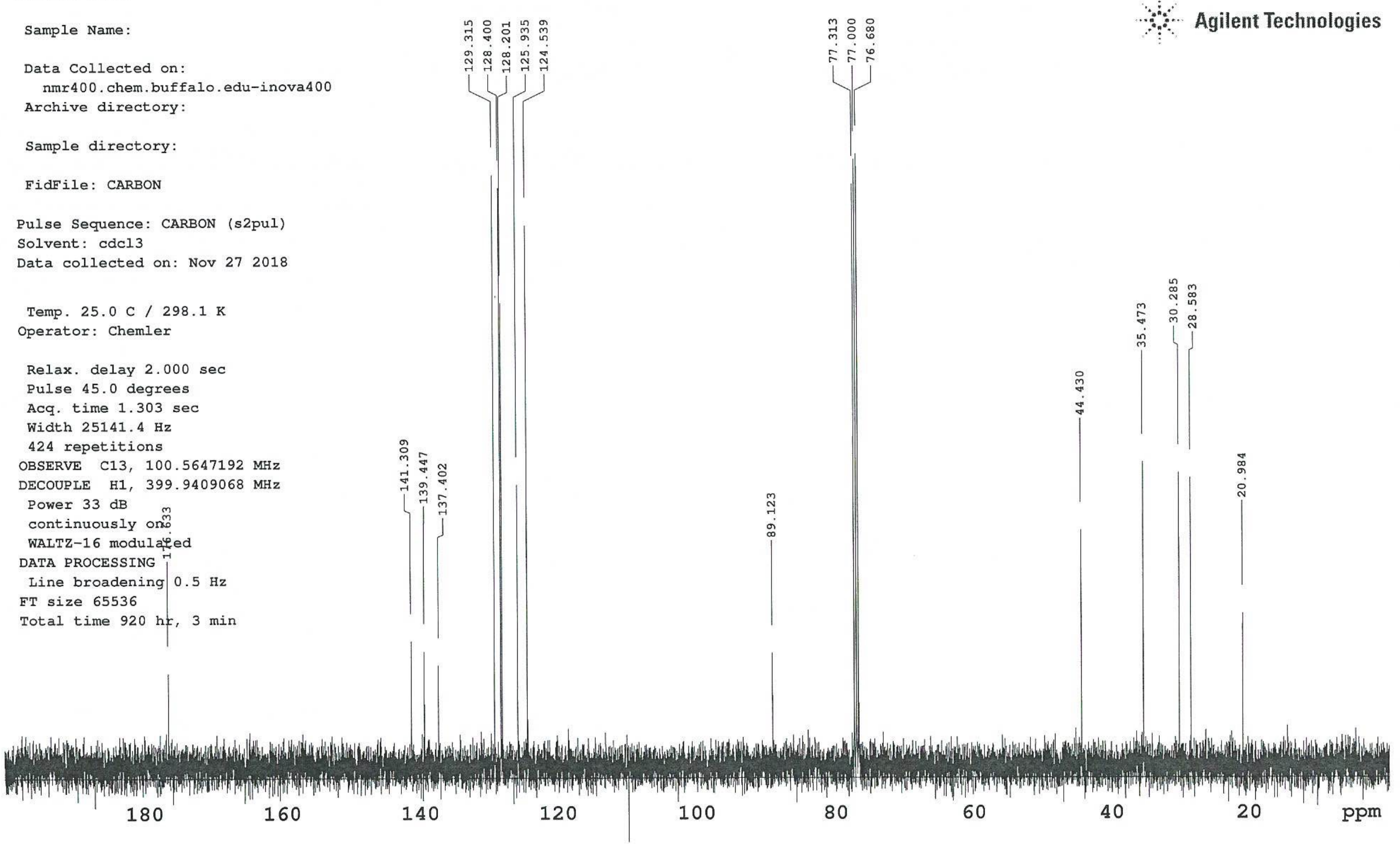

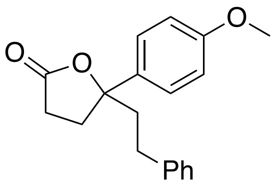
O
O
O
Ph

5c

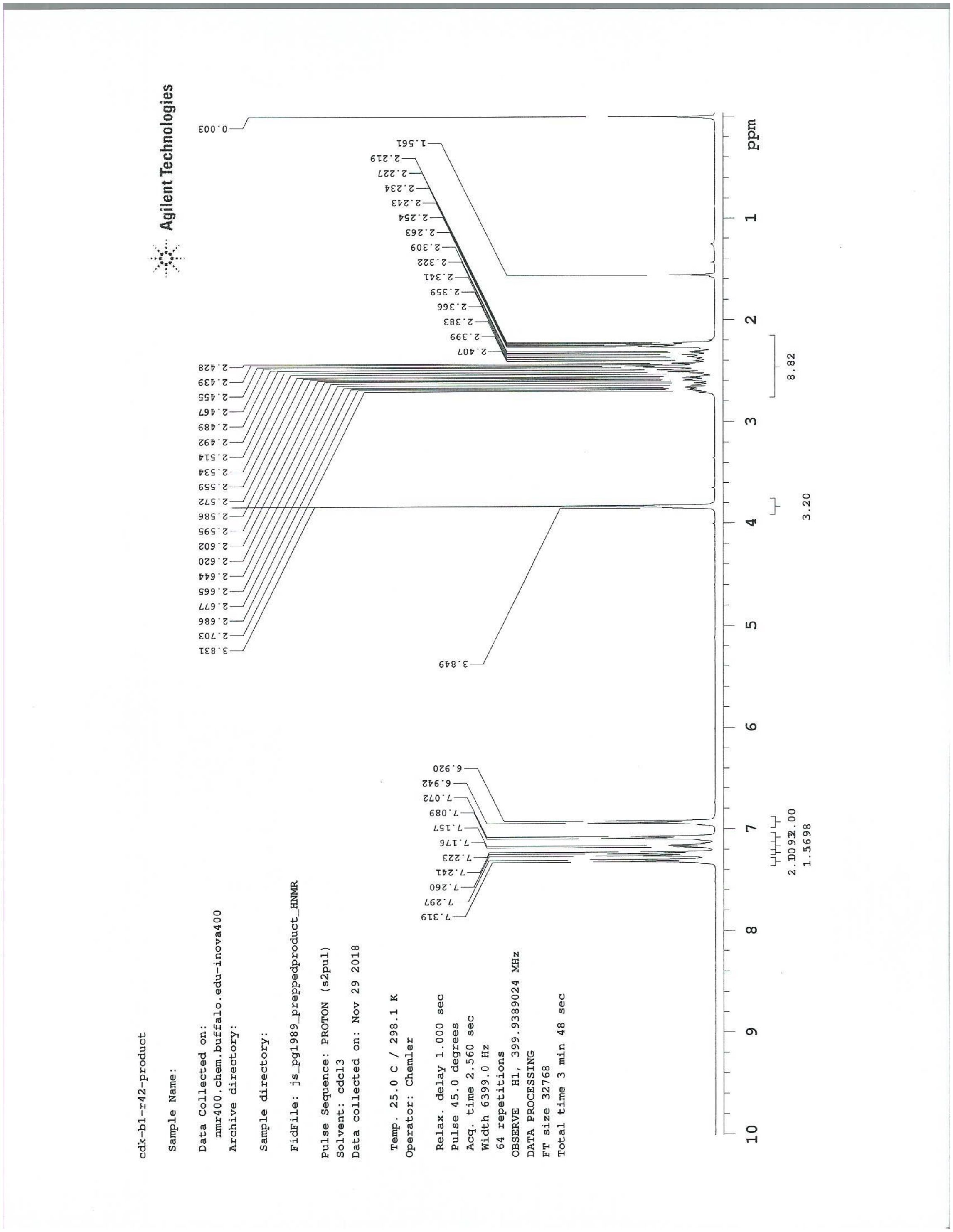
Agilent Technologies
cdk-b1-r42-product
Sample Name:
Data Collected on:
nmr400.chem.buffalo.edu-inova400
Archive directory:
Sample directory:
FidFile: js_pg1989_preppedproduct_HNMR
Pulse Sequence: PROTON (s2pul)
Solvent: cdcl3
Data collected on: Nov 29 2018
Temp. 25.0 C / 298.1 K
Operator: Chemler
Relax. delay 1.000 sec
Pulse 45.0 degrees
Acq. time 2.560 sec
Width 6399.0 Hz
64 repetitions
OBSERVE H1, 399.9389024 MHz
DATA PROCESSING
FT size 32768
Total time 3 min 48 sec
0.003
1.561
2.219
2.227
2.234
2.243
2.254
2.263
2.309
2.322
2.341
2.359
2.366
2.383
2.399
2.407
2.428
2.439
2.455
2.467
2.489
2.492
2.514
2.534
2.559
2.572
2.586
2.595
2.602
2.620
2.644
2.665
2.677
2.686
2.703
3.831
3.849
6.920
6.942
7.072
7.089
7.157
7.176
7.223
7.241
7.260
7.297
7.319
8.82
3.20
2.00
1.5698
ppm
10 9 8 7 6 5 4 3 2 1

Agilent Technologies

cdk-b1-r42-product

Sample Name:

Data Collected on:
nmr400.chem.buffalo.edu-inova400
Archive directory:

Sample directory:

FidFile: CARBON

Pulse Sequence: CARBON (s2pul)
Solvent: cdcl3
Data collected on: Nov 29 2018

Temp. 25.0 C / 298.1 K
Operator: Chemler

Relax. delay 2.000 sec
Pulse 45.0 degrees
Acq. time 1.303 sec
Width 25141.4 Hz
736 repetitions
OBSERVE C13, 100.5647177 MHz
DECOUPLE H1, 399.9409068 MHz
Power 33 dB
continuously on
WALTZ-16 modulated
DATA PROCESSING
Line broadening 0.5 Hz
FT size 65536
Total time 920 hr, 3 min

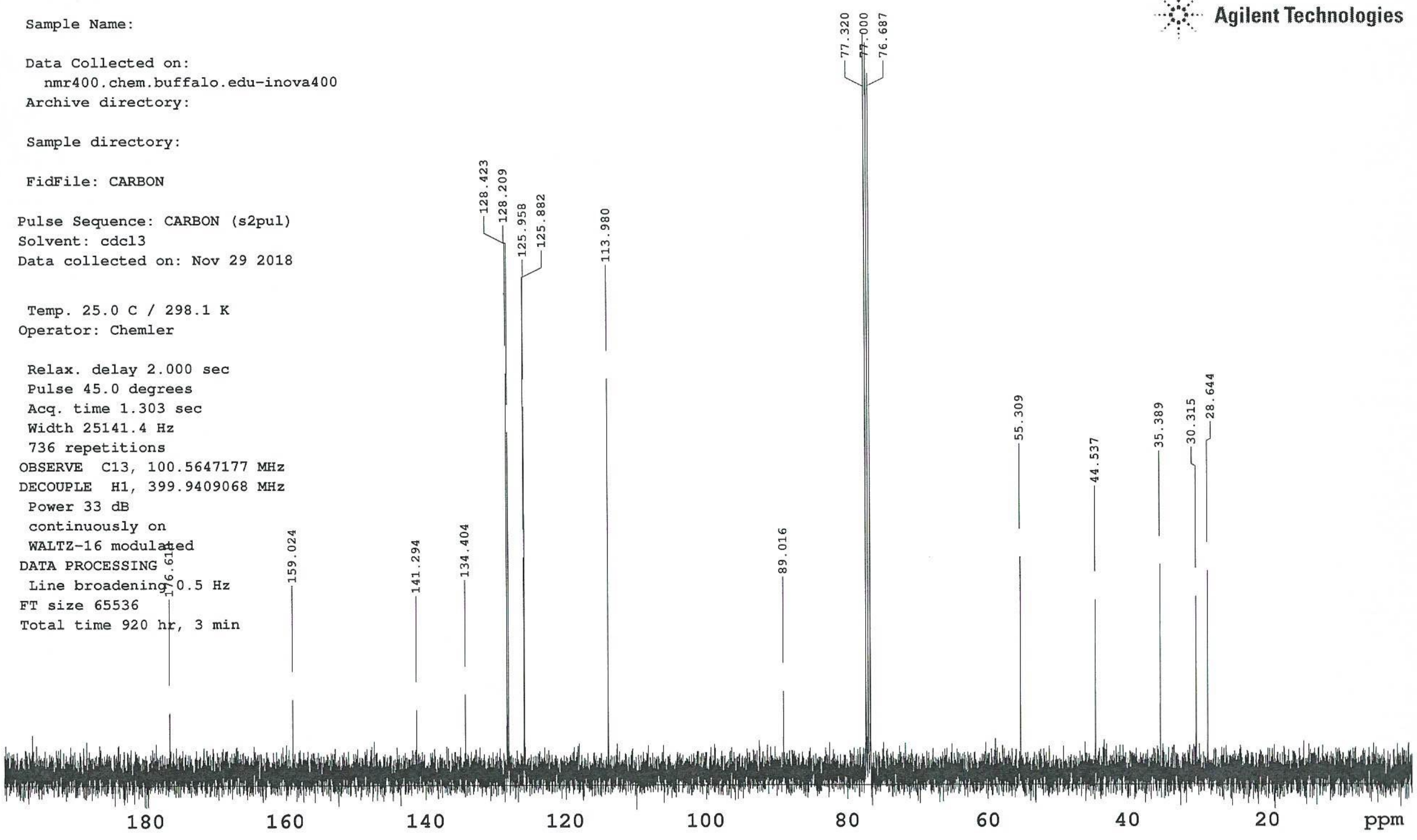

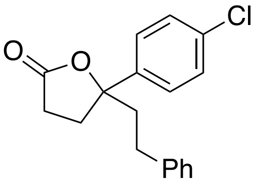
O
O
Cl
Ph

5d

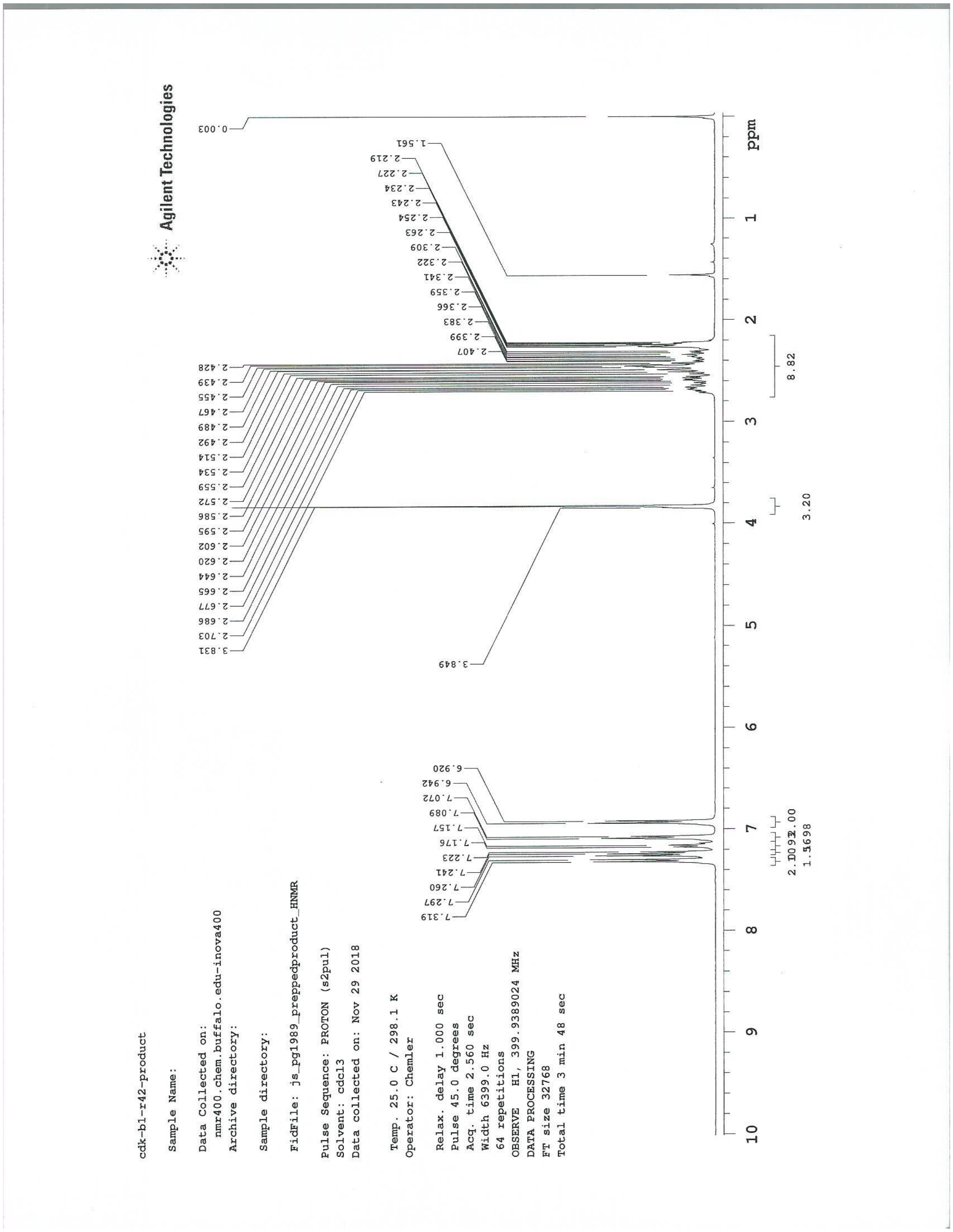
Agilent Technologies
cdk-b1-r42-product
Sample Name:
Data Collected on:
nmr400.chem.buffalo.edu-inova400
Archive directory:
Sample directory:
FidFile: js_pg1989_preppedproduct_HNMR
Pulse Sequence: PROTON (s2pul)
Solvent: cdcl3
Data collected on: Nov 29 2018
Temp. 25.0 C / 298.1 K
Operator: Chemler
Relax. delay 1.000 sec
Pulse 45.0 degrees
Acq. time 2.560 sec
Width 6399.0 Hz
64 repetitions
OBSERVE H1, 399.9389024 MHz
DATA PROCESSING
FT size 32768
Total time 3 min 48 sec
ppm

cdk-b2-r559-crude

Sample Name:

Data Collected on:
nmr300.chem.buffalo.edu-mercury300
Archive directory:

Sample directory:

FidFile: CARBON

Pulse Sequence: CARBON (s2pul)
Solvent: cdcl3
Data collected on: Dec 8 2018

Operator: Chemler

Relax. delay 2.000 sec
Pulse 45.0 degrees
Acq. time 0.868 sec
Width 18867.9 Hz
12032 repetitions
OBSERVE C13, 75.4536389 MHz
DECOUPLE H1, 300.0754430 MHz
Power 37 dB
continuously on
WALTZ-16 modulated
DATA PROCESSING
Line broadening 0.5 Hz
FT size 32768
Total time 79 hr, 55 min

Agilent Technologies

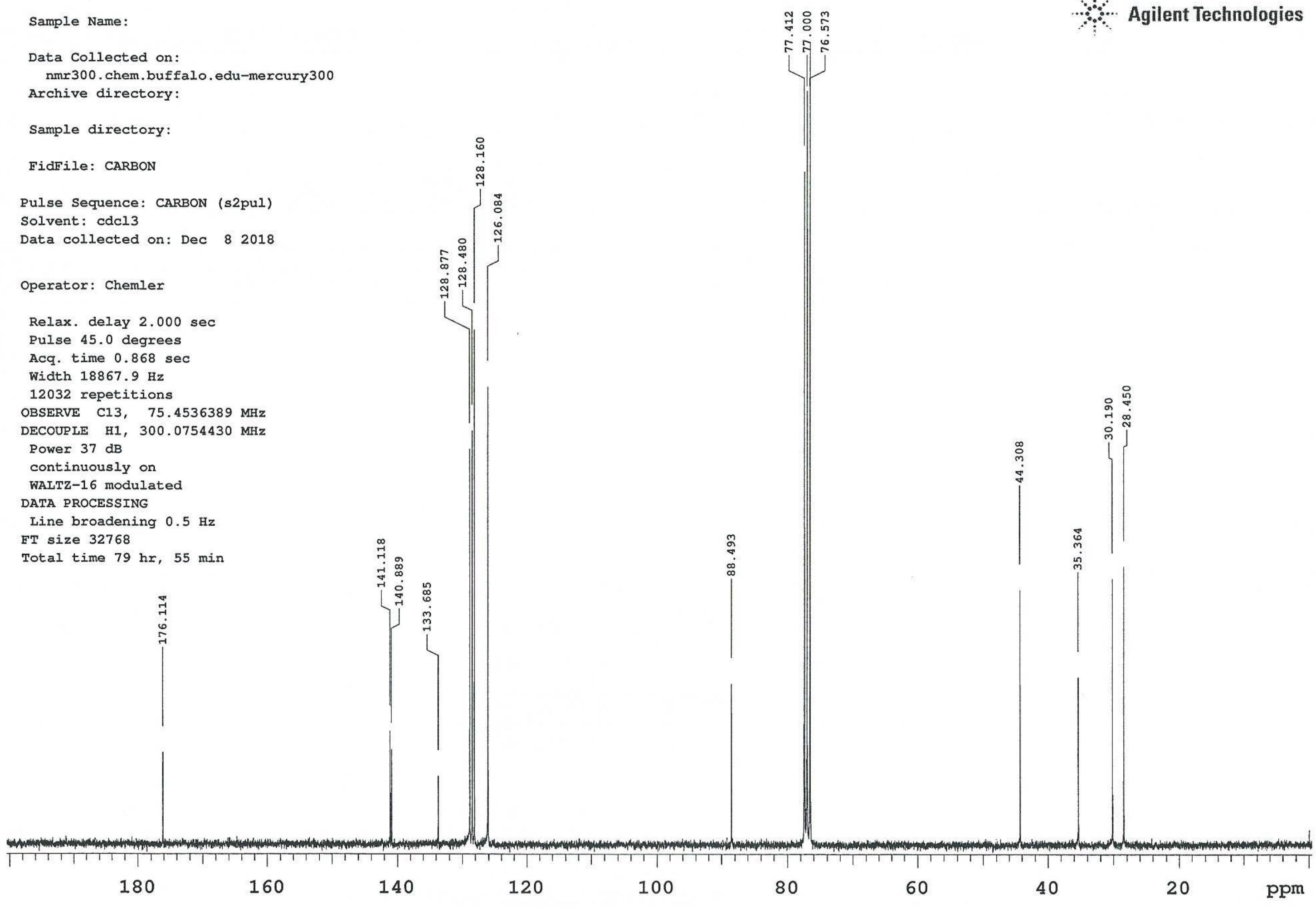

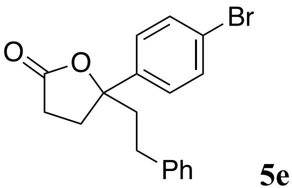
O
O
Br
Ph

5e

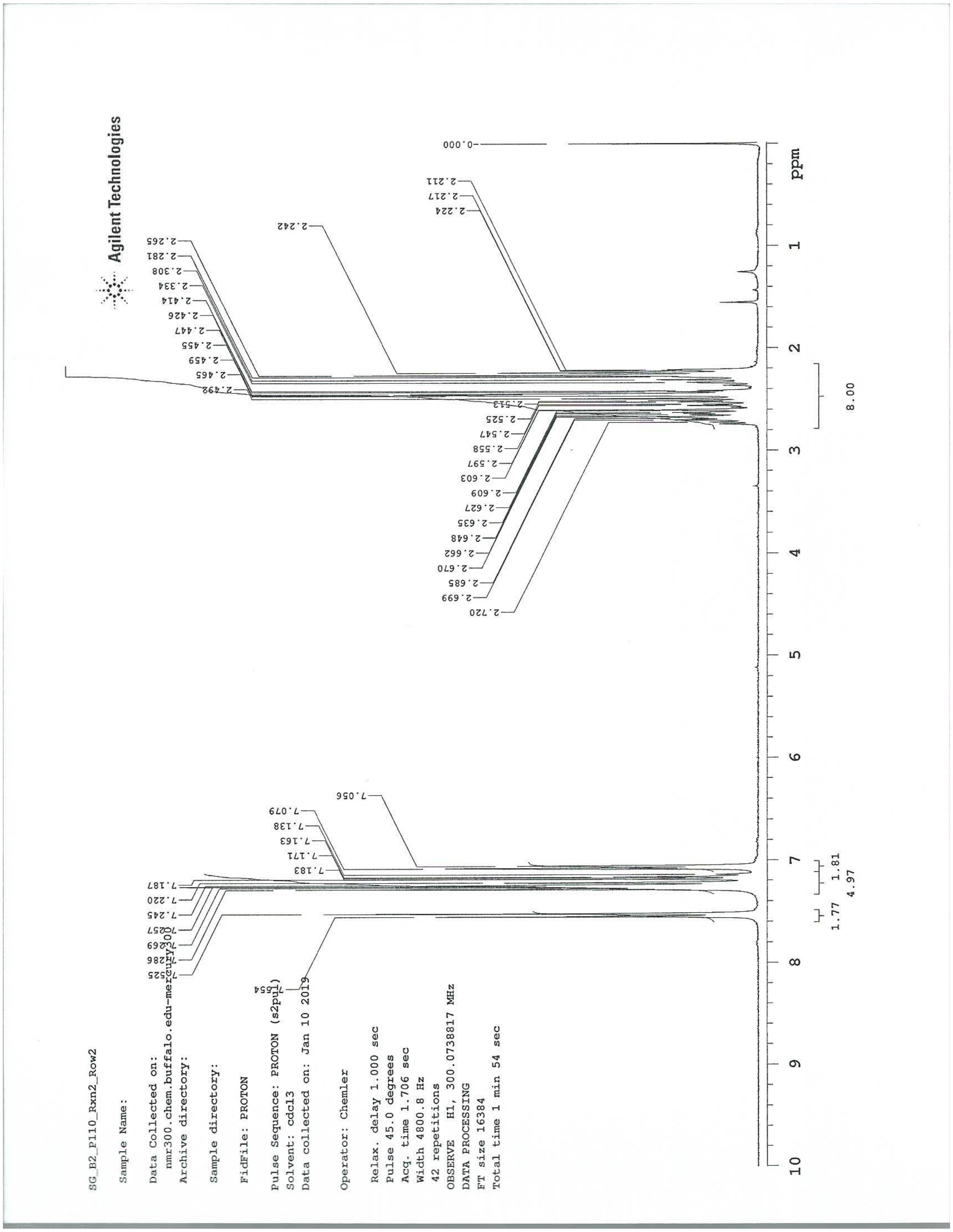
Agilent Technologies
SG_B2_P110_Rxn2_Row2
Sample Name:
Data Collected on:
nmr300.chem.buffalo.edu-mercury300
Archive directory:
Sample directory:
FidFile: PROTON
Pulse Sequence: PROTON (s2pul)
Solvent: cdcl3
Data collected on: Jan 10 2019
Operator: Chemler
Relax. delay 1.000 sec
Pulse 45.0 degrees
Acq. time 1.706 sec
Width 4800.8 Hz
42 repetitions
OBSERVE H1, 300.0738817 MHz
DATA PROCESSING
FT size 16384
Total time 1 min 54 sec
ppm
8.00
1.77
4.97
1.81

SG_B2_P110_Rxn2_Row2

Sample Name:

Data Collected on:
nmr300.chem.buffalo.edu-mercury300
Archive directory:

Sample directory:

FidFile: CARBON

Pulse Sequence: CARBON (s2pul)
Solvent: cdcl3
Data collected on: Jan 10 2019

Operator: Chemler

Relax. delay 2.000 sec
Pulse 45.0 degrees
Acq. time 0.868 sec
Width 18867.9 Hz
1080 repetitions
OBSERVE C13, 75.4536377 MHz
DECOUPLE H1, 300.0754430 MHz
Power 37 dB
continuously on
WALTZ-16 modulated
DATA PROCESSING
Line broadening 0.5 Hz
FT size 32768
Total time 79 hr, 55 min

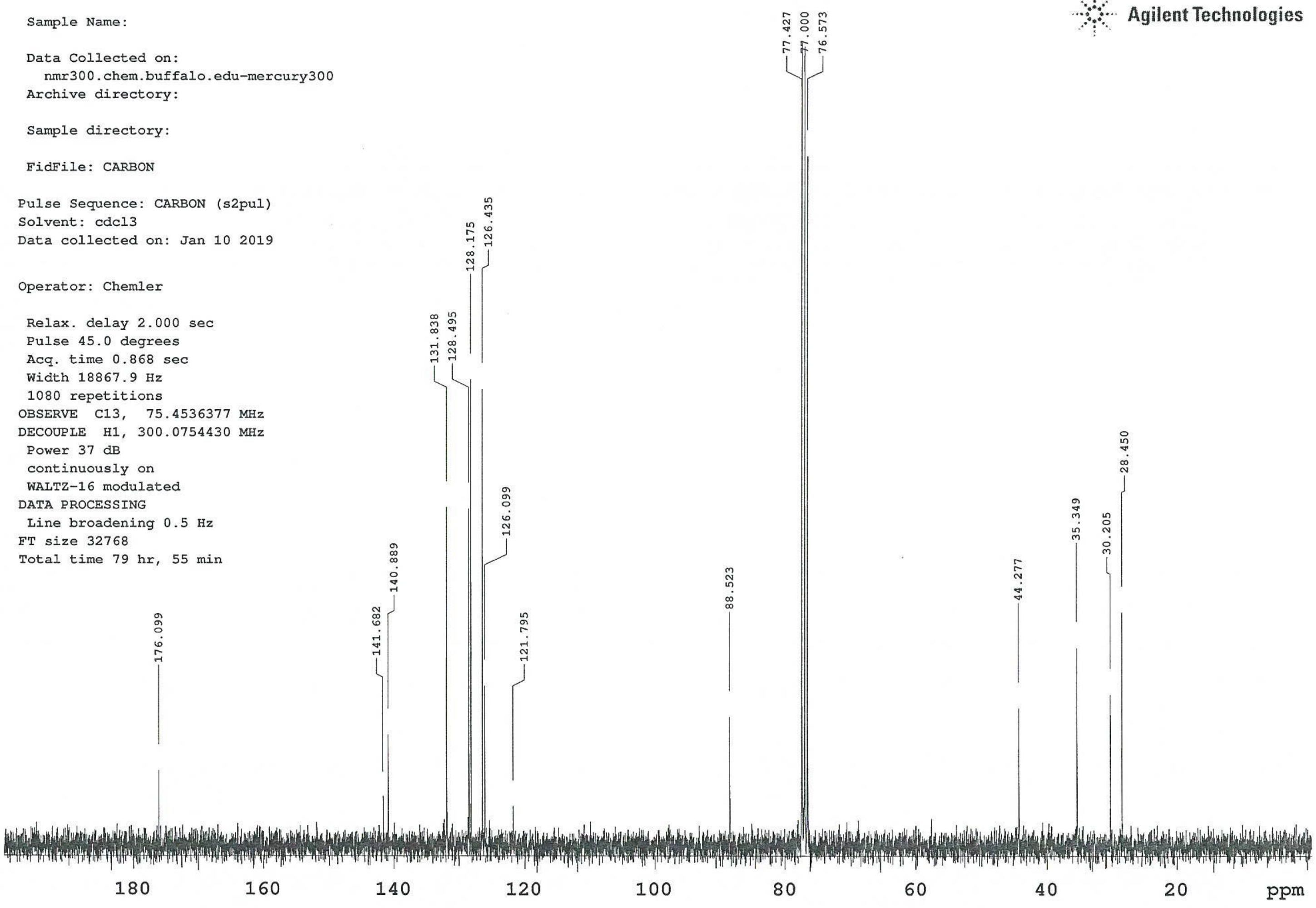

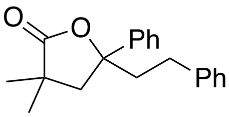

5f

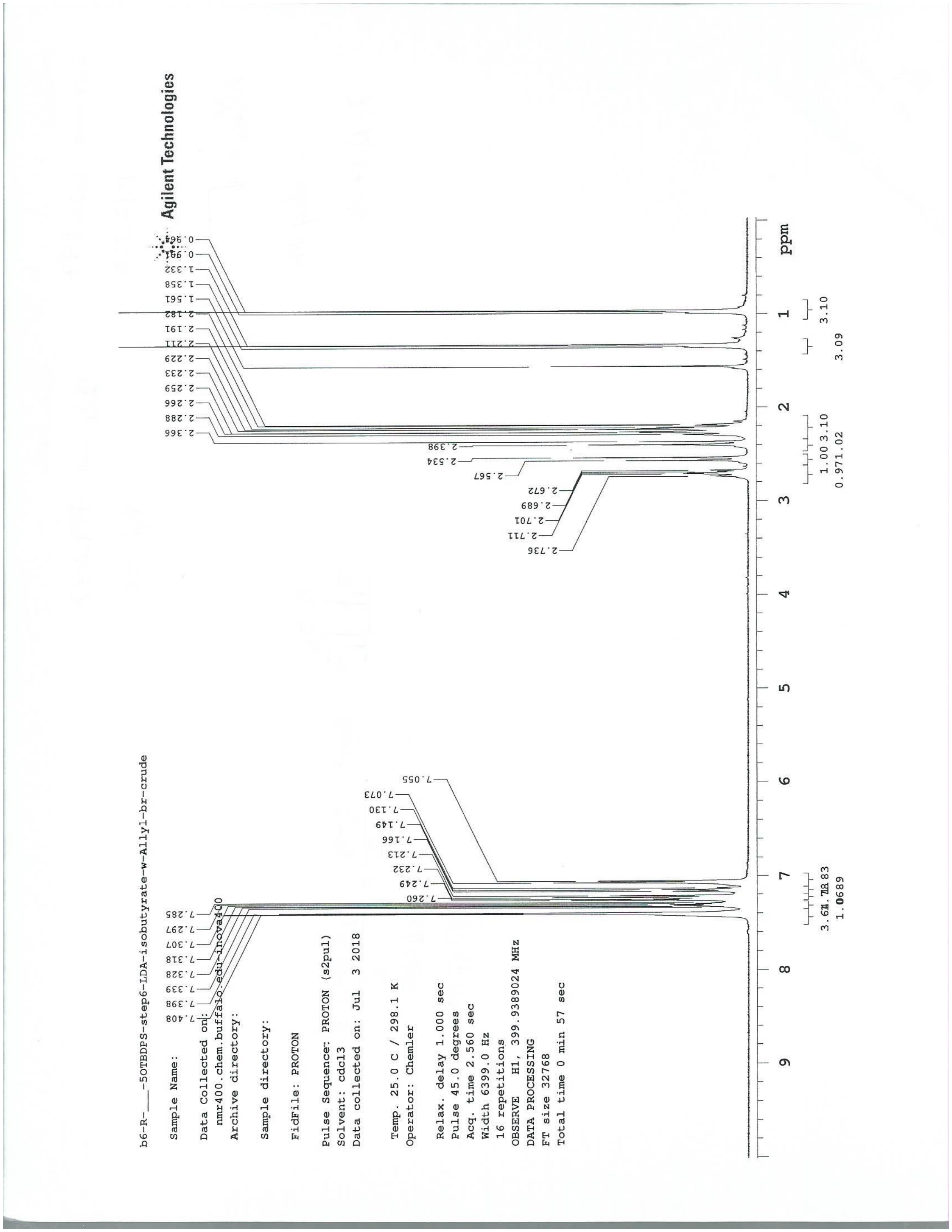
Agilent Technologies
b6-R-___-5OTBDPS-step6-LDA-isobutyrate-w-Allyl-br-crude
Sample Name:
Data Collected on:
nmr400.chem.buffalo.edu-inova400
Archive directory:
Sample directory:
FidFile: PROTON
Pulse Sequence: PROTON (s2pul)
Solvent: cdcl3
Data collected on: Jul 3 2018
Temp. 25.0 C / 298.1 K
Operator: Chemler
Relax. delay 1.000 sec
Pulse 45.0 degrees
Acq. time 2.560 sec
Width 6399.0 Hz
16 repetitions
OBSERVE H1, 399.9389024 MHz
DATA PROCESSING
FT size 32768
Total time 0 min 57 sec
7.408
7.398
7.339
7.328
7.318
7.307
7.297
7.285
7.260
7.249
7.232
7.213
7.166
7.149
7.130
7.073
7.055
2.736
2.711
2.701
2.689
2.672
2.567
2.534
2.398
2.366
2.288
2.266
2.259
2.233
2.229
2.211
2.191
2.182
1.561
1.358
1.332
0.961
0.964
ppm
3.10
3.09
3.10
1.00
0.97
1.02

b6-R-___-5OTBDPS-step6-LDA-isobutyrate-w-Allyl-br-crude

Sample Name:

Data Collected on:
nmr400.chem.buffalo.edu-inova400
Archive directory:

Sample directory:

FidFile: CARBON

Pulse Sequence: CARBON (s2pul)
Solvent: cdcl3
Data collected on: Jul 3 2018

Temp. 25.0 C / 298.1 K
Operator: Chemler

Relax. delay 2.000 sec
Pulse 45.0 degrees
Acq. time 1.303 sec
Width 25141.4 Hz
656 repetitions
OBSERVE C13, 100.5647177 MHz
DECOUPLE H1, 399.9409068 MHz
Power 33 dB
continuously on
WALTZ-16 modulated
DATA PROCESSING
Line broadening 0.5 Hz
FT size 65536
Total time 92 hr

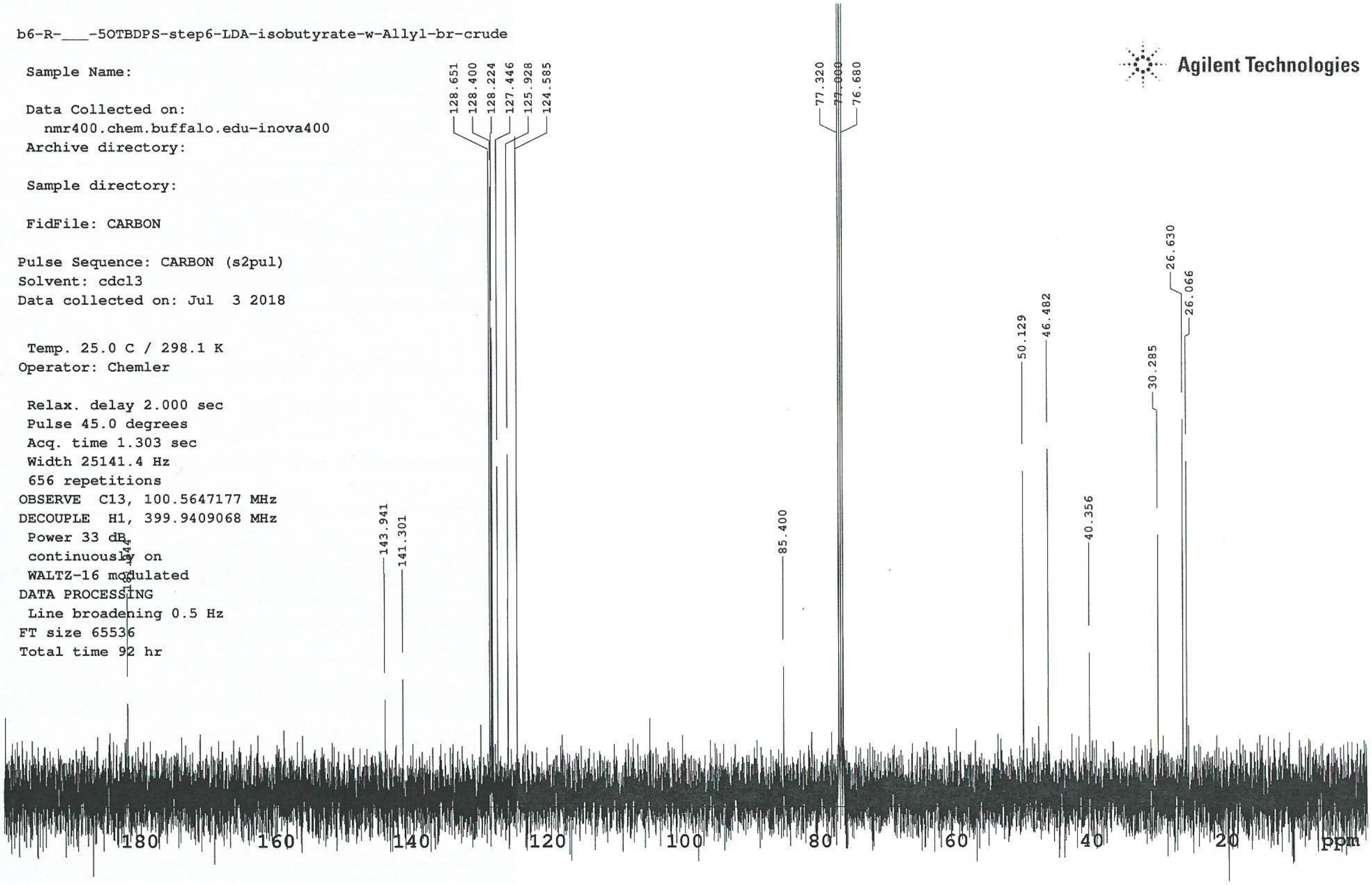

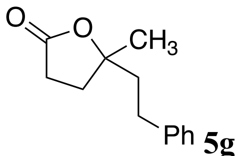
O
O
CH3
Ph

5g

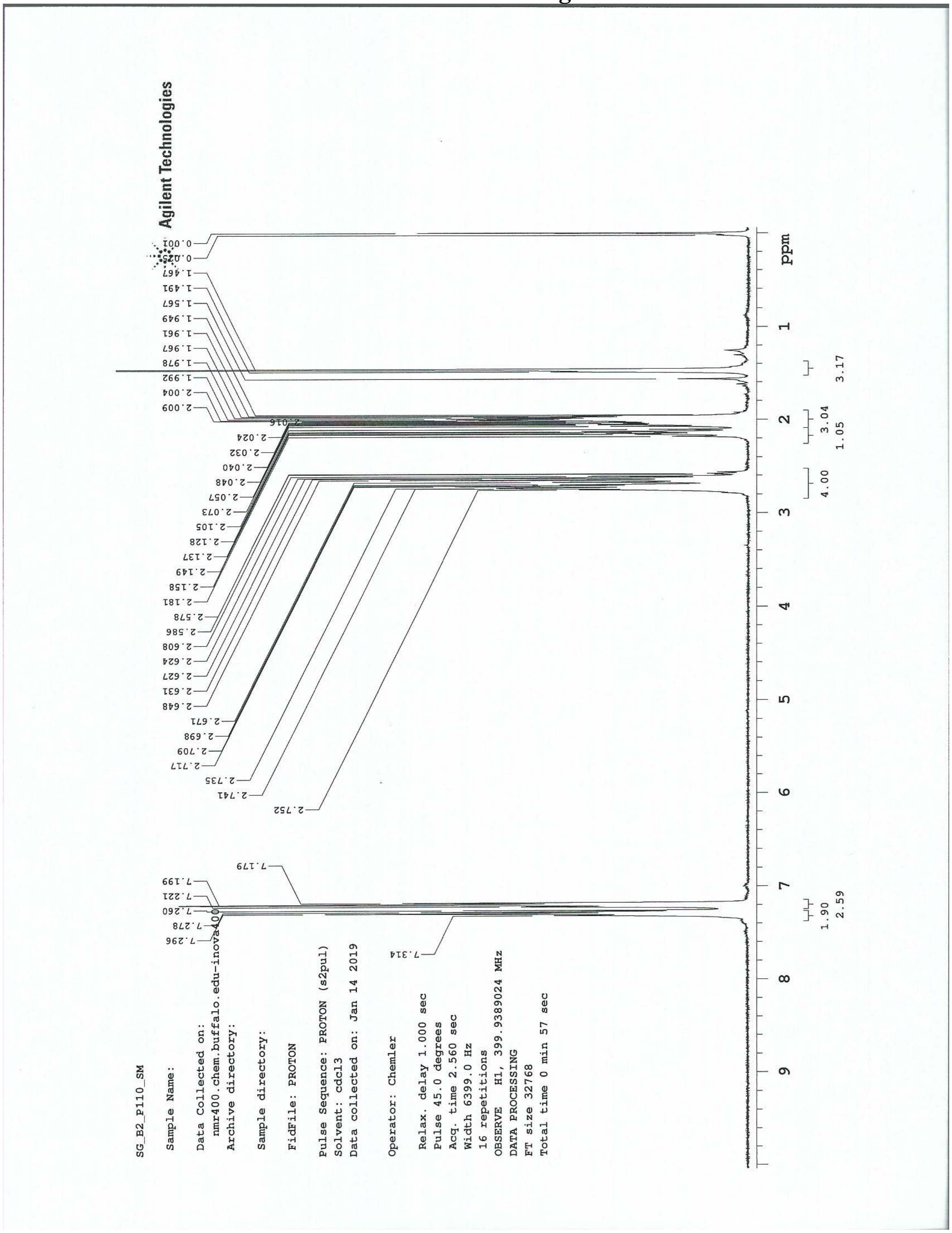
Agilent Technologies
SG_B2_P110_SM
Sample Name:
Data Collected on:
nmr400.chem.buffalo.edu-inova400
Archive directory:
Sample directory:
FidFile: PROTON
Pulse Sequence: PROTON (s2pul)
Solvent: cdcl3
Data collected on: Jan 14 2019
Operator: Chemler
Relax. delay 1.000 sec
Pulse 45.0 degrees
Acq. time 2.560 sec
Width 6399.0 Hz
16 repetitions
OBSERVE H1, 399.9389024 MHz
DATA PROCESSING
FT size 32768
Total time 0 min 57 sec
7.314
7.296
7.278
7.260
7.221
7.199
7.179
2.752
2.741
2.735
2.717
2.709
2.698
2.671
2.648
2.631
2.627
2.624
2.608
2.586
2.578
2.181
2.158
2.149
2.137
2.128
2.105
2.073
2.057
2.048
2.040
2.032
2.024
2.016
2.009
2.004
1.992
1.978
1.967
1.961
1.949
1.567
1.491
1.467
0.001
1.90
2.59
4.00
1.05
3.04
3.17
9
8
7
6
5
4
3
2
1
ppm

JE_2_93_crude

Sample Name:

Data Collected on:
nmr400.chem.buffalo.edu-inova400
Archive directory:

Sample directory:

FidFile: CARBON

Pulse Sequence: CARBON (s2pul)
Solvent: cdcl3
Data collected on: Jan 9 2019

Temp. 25.0 C / 298.1 K
Operator: Chemler

Relax. delay 2.000 sec
Pulse 45.0 degrees
Acq. time 1.303 sec
Width 25141.4 Hz
2208 repetitions
OBSERVE C13, 100.5647177 MHz
DECOUPLE H1, 399.9409068 MHz
Power 33 dB
continuously on
WALTZ-16 modulated
DATA PROCESSING
Line broadening 0.5 Hz
FT size 65536
Total time 92 hr

Agilent Technologies

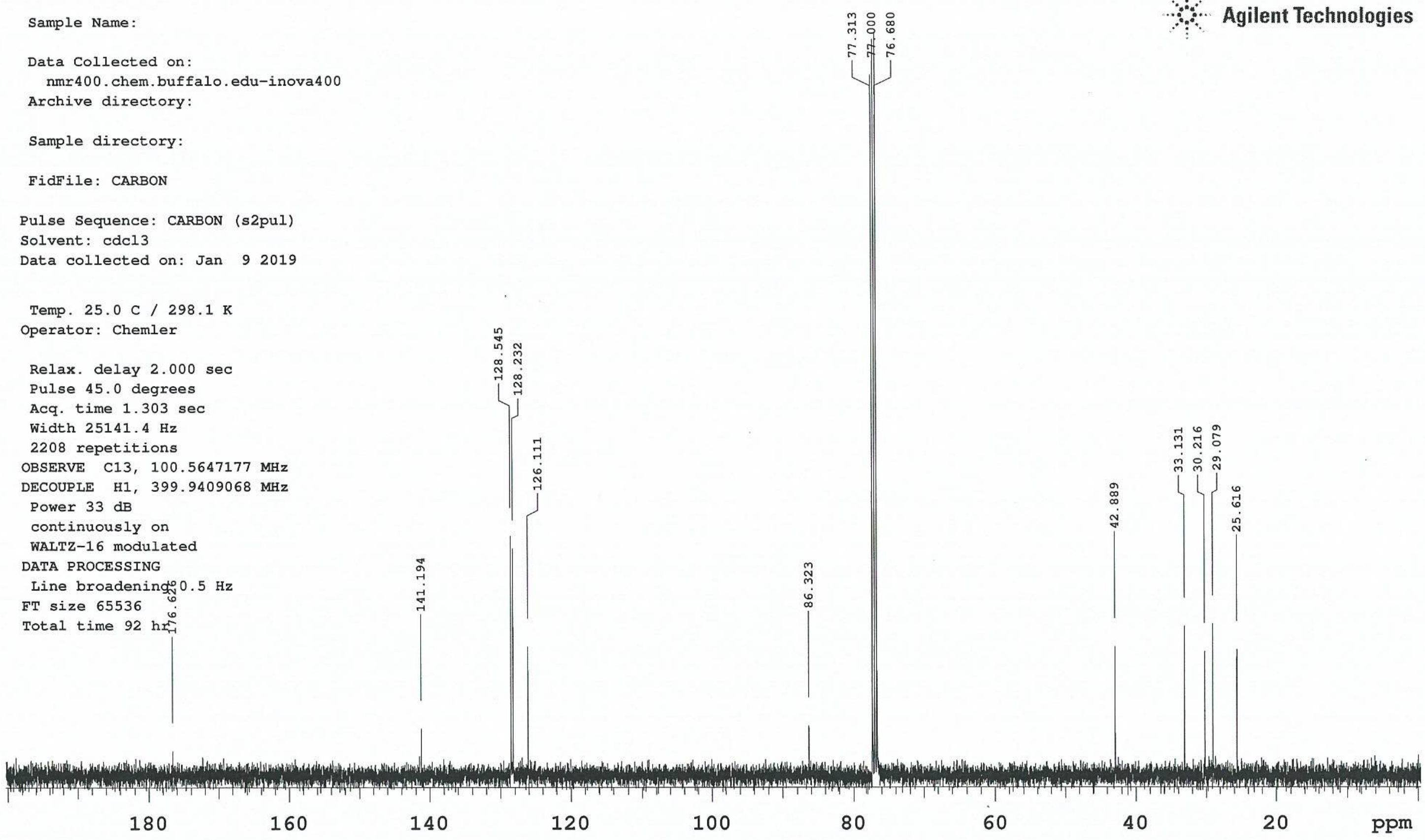

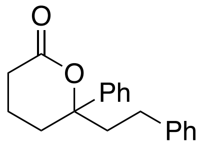

6a

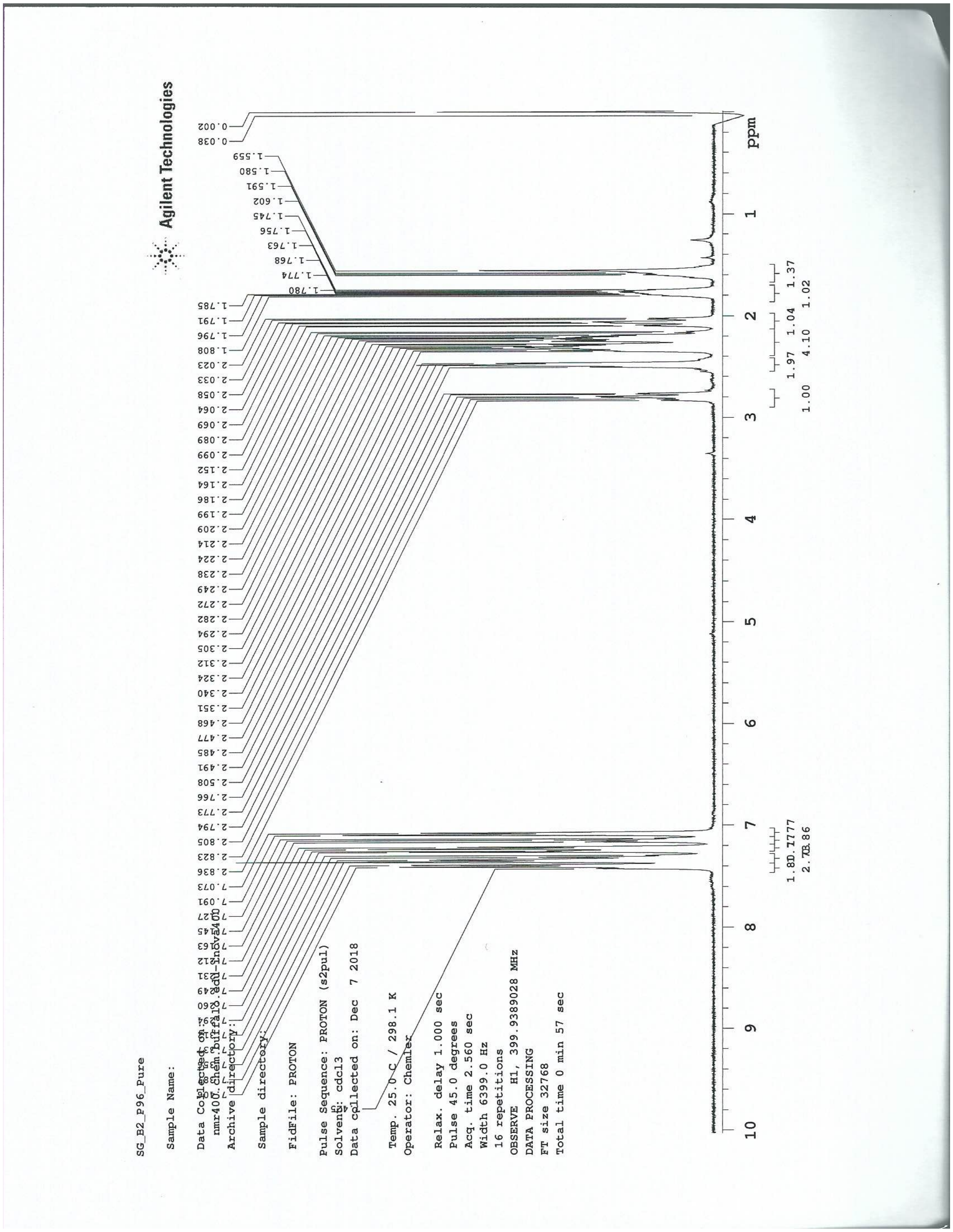
Agilent Technologies
SG_B2_P96_Pure
Sample Name:
Data Collected on:
nmr400.chem.buffalo.edu-inova400
Archive directory:
Sample directory:
FidFile: PROTON
Pulse Sequence: PROTON (s2pul)
Solvent: cdcl3
Data collected on: Dec 7 2018
Temp. 25.0 C / 298.1 K
Operator: Chemler
Relax. delay 1.000 sec
Pulse 45.0 degrees
Acq. time 2.560 sec
Width 6399.0 Hz
16 repetitions
OBSERVE H1, 399.9389028 MHz
DATA PROCESSING
FT size 32768
Total time 0 min 57 sec
ppm

SG_B2_P96_Pure

Sample Name:

Data Collected on:
nmr400.chem.buffalo.edu-inova400
Archive directory:

Sample directory:

FidFile: CARBON

Pulse Sequence: CARBON (s2pul)
Solvent: cdcl3
Data collected on: Dec 7 2018

Temp. 25.0 C / 298.1 K
Operator: Chemler

Relax. delay 2.000 sec
Pulse 45.0 degrees
Acq. time 1.303 sec
Width 25141.4 Hz
2416 repetitions
OBSERVE C13, 100.5647177 MHz
DECOUPLE H1, 399.9409068 MHz
Power 33 dB
continuously on
WALTZ-16 modulated
DATA PROCESSING
Line broadening 0.5 Hz
FT size 65536
Total time 920 hr, 3 min

Agilent Technologies

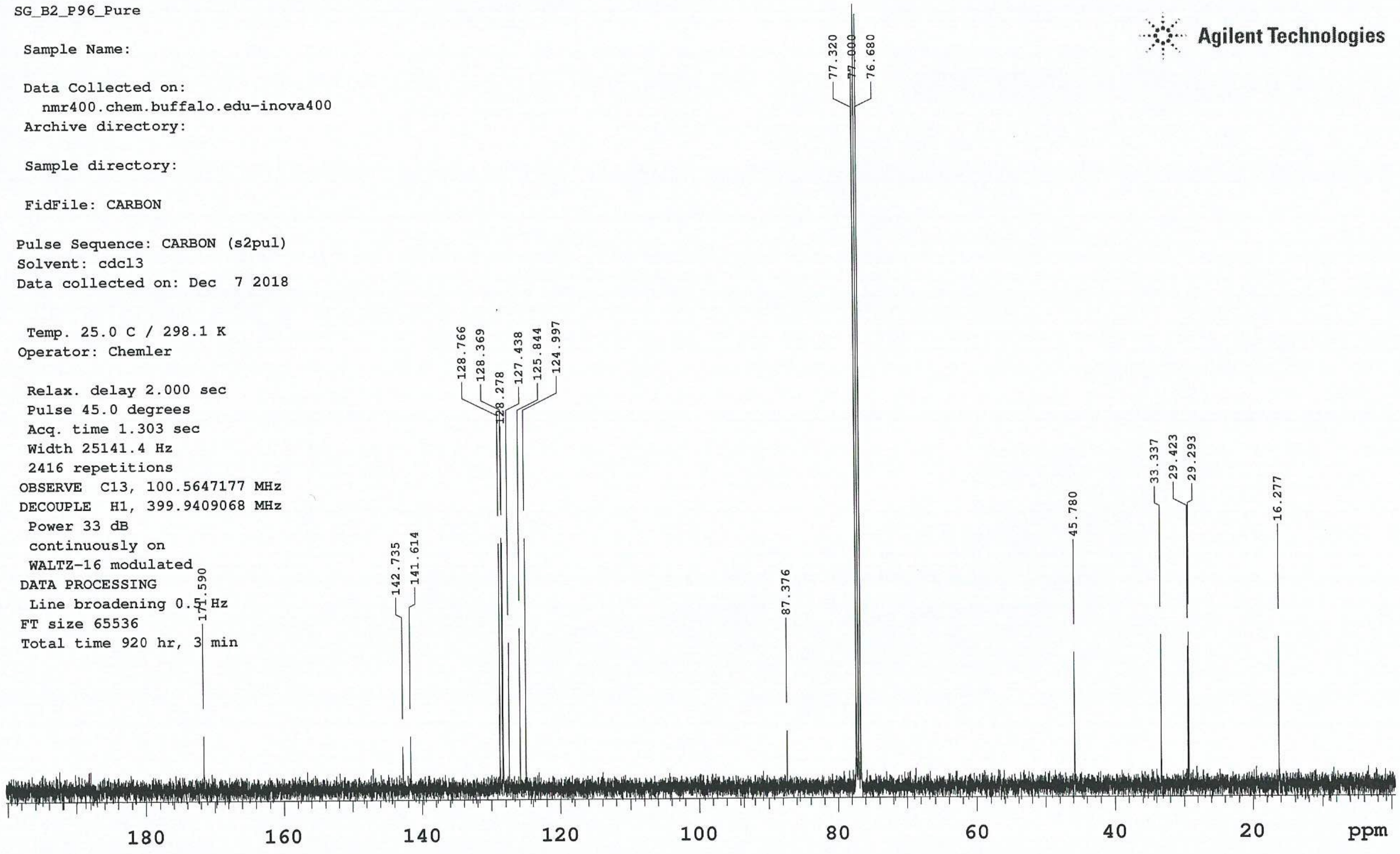

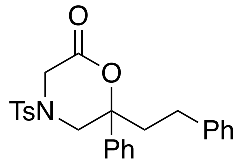
O
O
TsN
Ph
Ph

6b

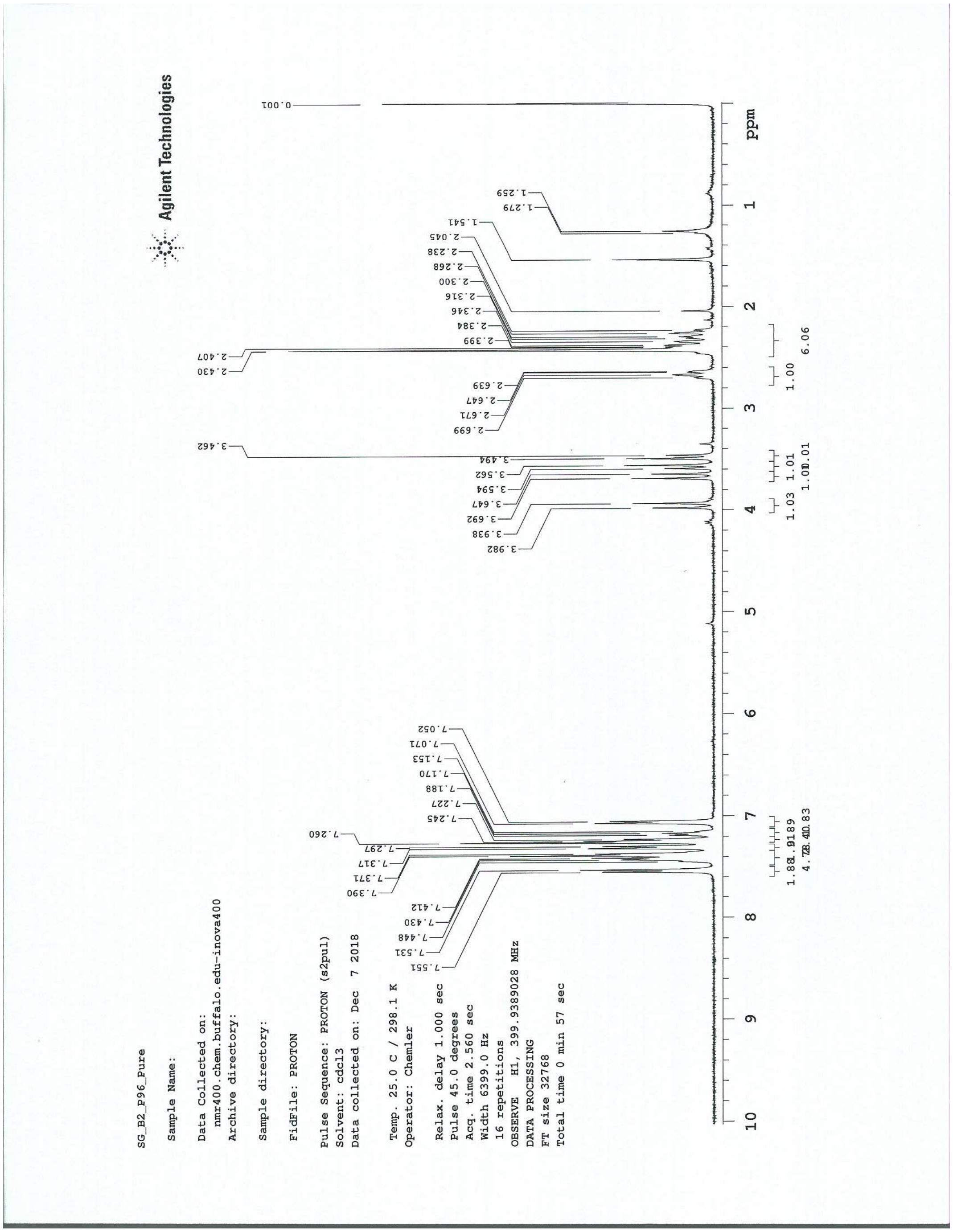
Agilent Technologies
SG_B2_P96_Pure
Sample Name:
Data Collected on:
nmr400.chem.buffalo.edu-inova400
Archive directory:
Sample directory:
FidFile: PROTON
Pulse Sequence: PROTON (s2pul)
Solvent: cdcl3
Data collected on: Dec 7 2018
Temp. 25.0 C / 298.1 K
Operator: Chemler
Relax. delay 1.000 sec
Pulse 45.0 degrees
Acq. time 2.560 sec
Width 6399.0 Hz
16 repetitions
OBSERVE H1, 399.9389028 MHz
DATA PROCESSING
FT size 32768
Total time 0 min 57 sec
ppm

cdk-b1-r42-product

Sample Name:

Data Collected on:
nmr400.chem.buffalo.edu-inova400
Archive directory:

Sample directory:

FidFile: CARBON

Pulse Sequence: CARBON (s2pul)
Solvent: cdcl3
Data collected on: Nov 29 2018

Temp. 25.0 C / 298.1 K
Operator: Chemler

Relax. delay 2.000 sec
Pulse 45.0 degrees
Acq. time 1.303 sec
Width 25141.4 Hz
1448 repetitions
OBSERVE C13, 100.5647177 MHz
DECOUPLE H1, 399.9409068 MHz
Power 33 dB
continuously on
WALTZ-16 modulated
DATA PROCESSING
Line broadening 0.5 Hz
FT size 65536
Total time 92 hr

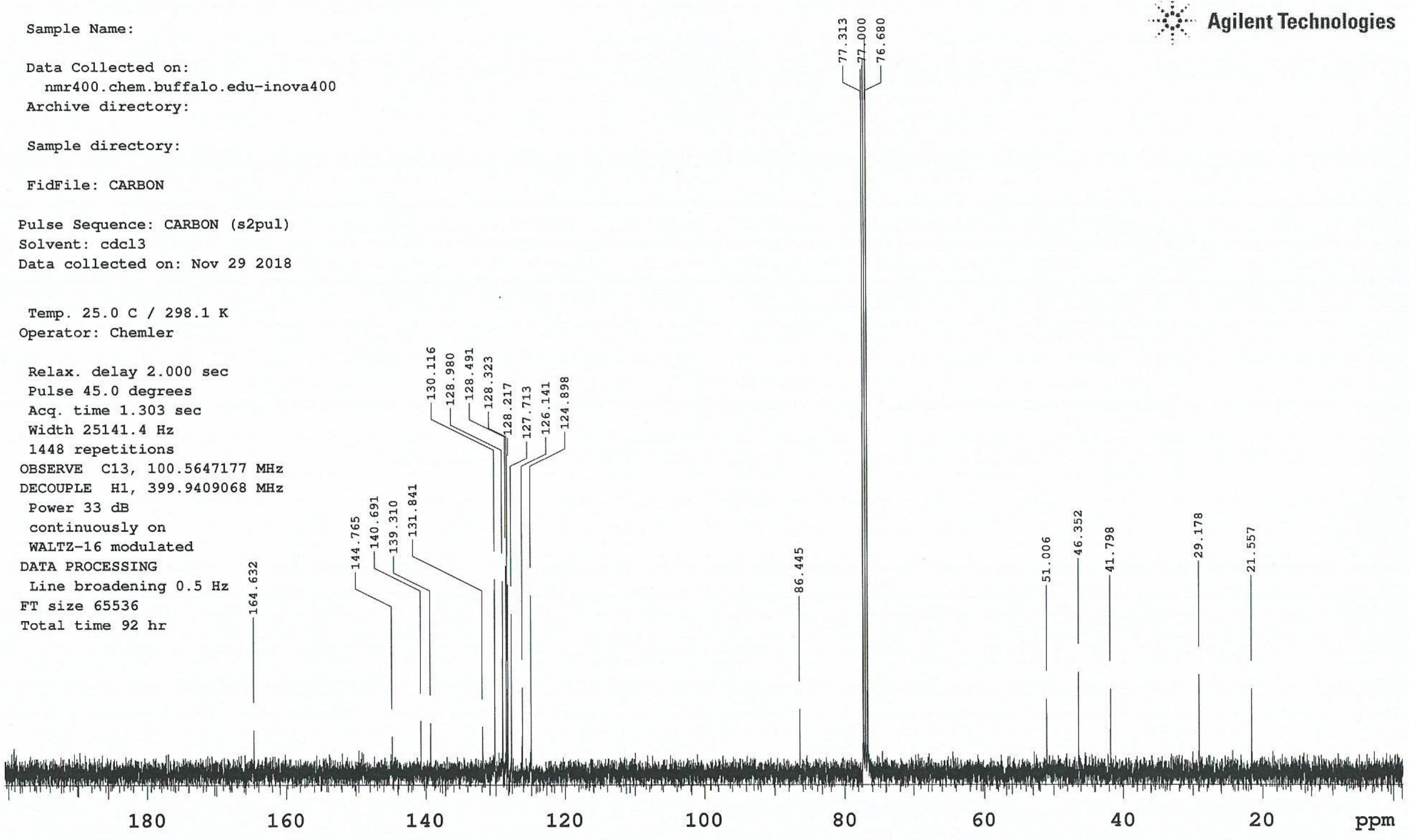

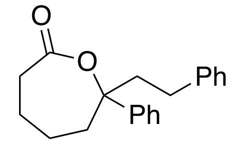

7a

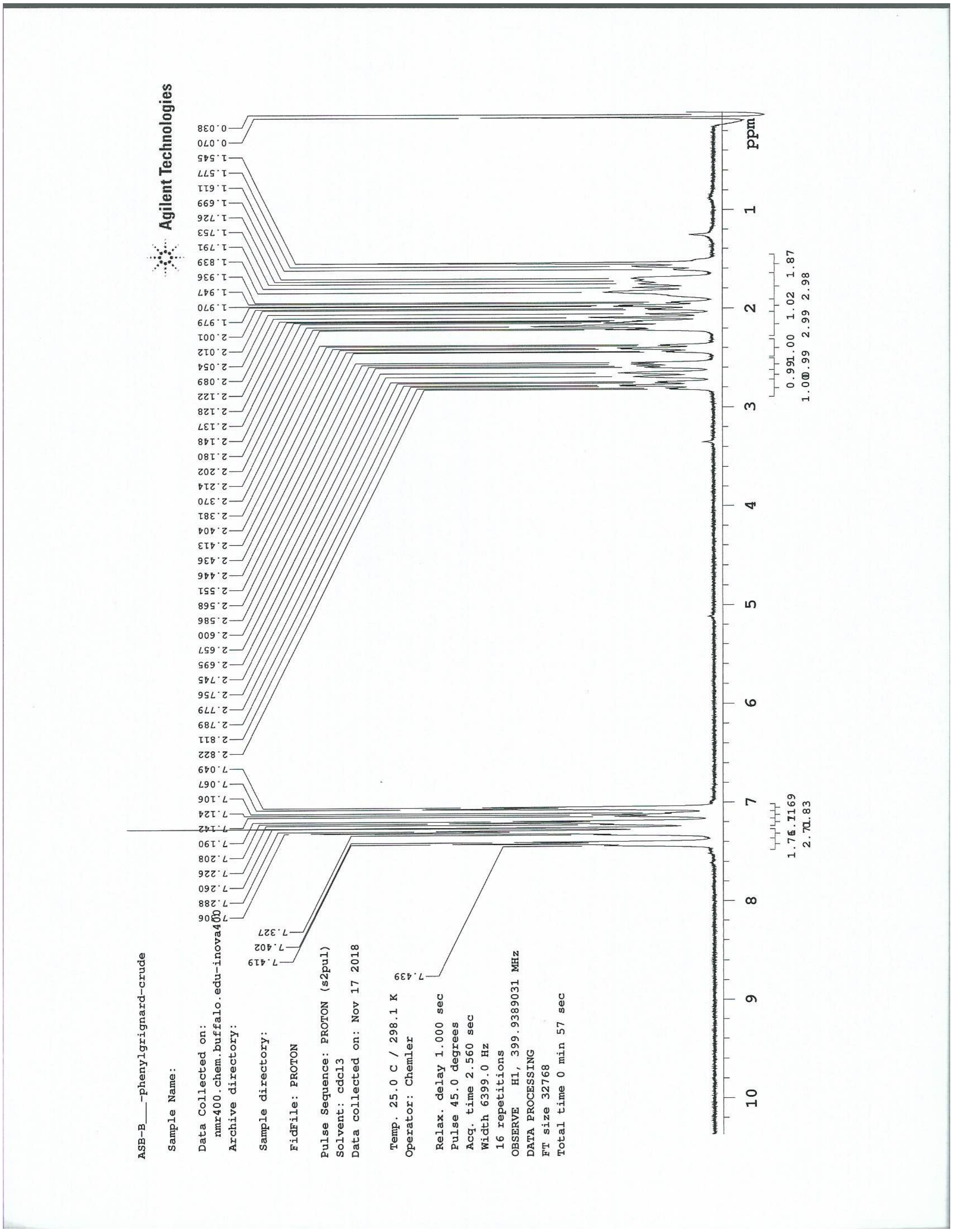

Agilent Technologies
ASB-B___-phenylgrignard-crude
Sample Name:
Data Collected on:
nmr400.chem.buffalo.edu-inova400
Archive directory:
Sample directory:
FidFile: PROTON
Pulse Sequence: PROTON (s2pul)
Solvent: cdcl3
Data collected on: Nov 17 2018
Temp. 25.0 C / 298.1 K
Operator: Chemler
Relax. delay 1.000 sec
Pulse 45.0 degrees
Acq. time 2.560 sec
Width 6399.0 Hz
16 repetitions
OBSERVE H1, 399.9389031 MHz
DATA PROCESSING
FT size 32768
Total time 0 min 57 sec
ppm

ASB-B___-phenylgrignard-crude

Sample Name:

Data Collected on:
nmr400.chem.buffalo.edu-inova400
Archive directory:

Sample directory:

FidFile: CARBON

Pulse Sequence: CARBON (s2pul)
Solvent: cdcl3
Data collected on: Nov 17 2018

Temp. 25.0 C / 298.1 K
Operator: Chemler

Relax. delay 2.000 sec
Pulse 45.0 degrees
Acq. time 1.303 sec
Width 25141.4 Hz
16856 repetitions
OBSERVE C13, 100.5647169 MHz
DECOUPLE H1, 399.9409068 MHz
Power 33 dB
continuously on
WALTZ-16 modulated
DATA PROCESSING
Line broadening 0.5 Hz
FT size 65536
Total time 9200 [illegible]r, 37 min

Agilent Technologies

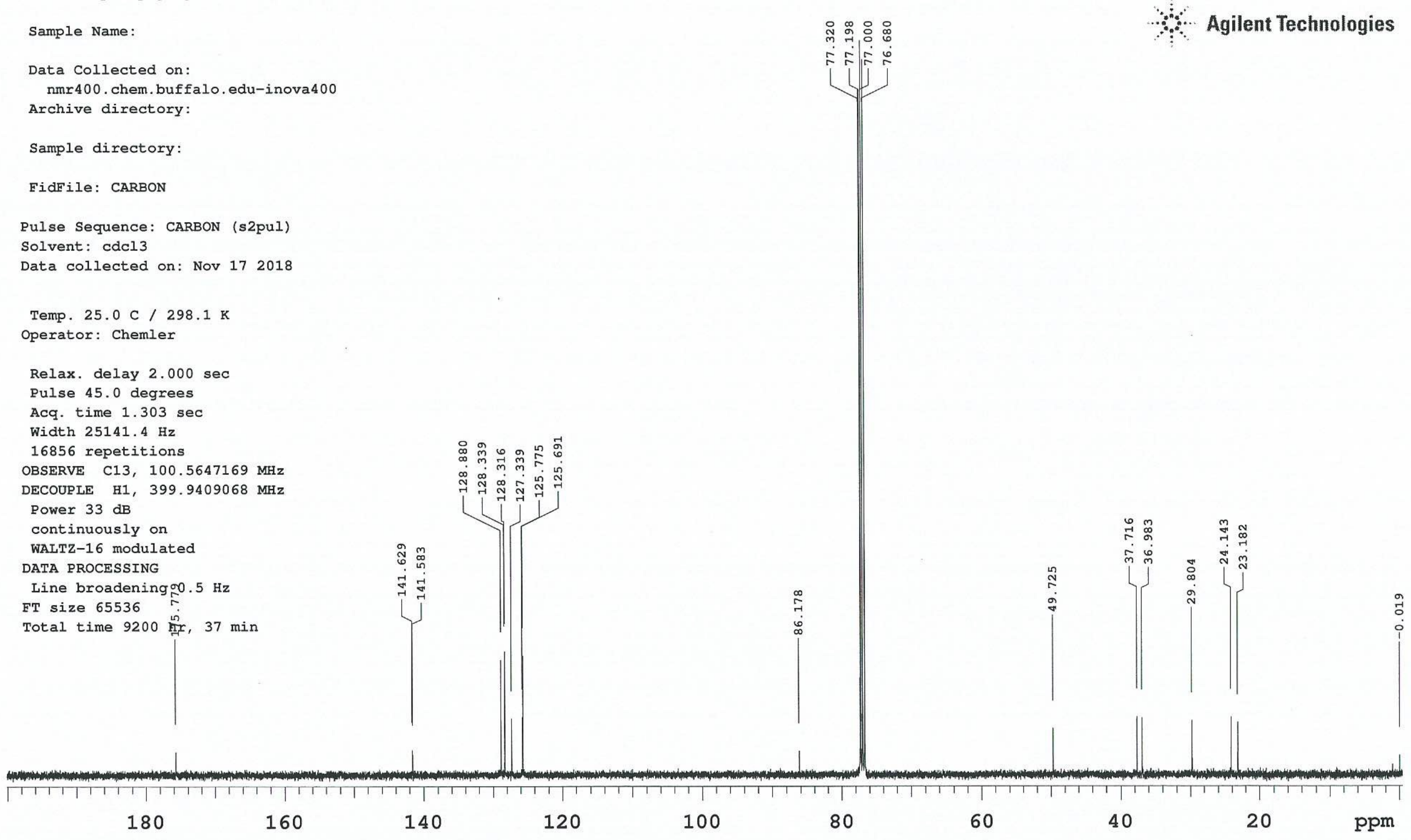

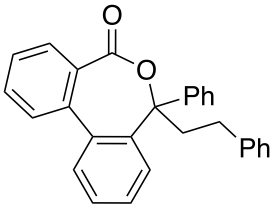
O
O
Ph
Ph

7b

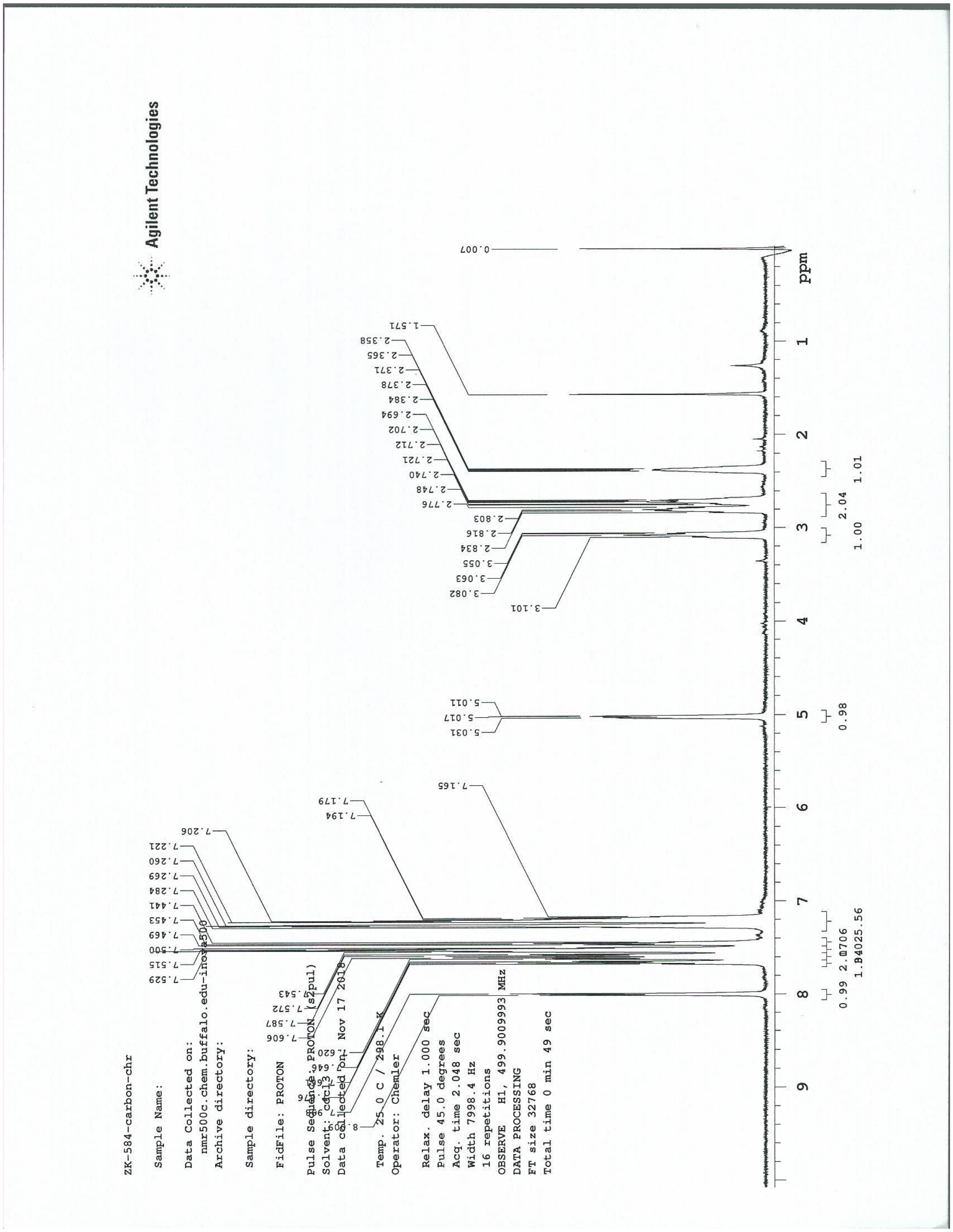
Agilent Technologies
ZK-584-carbon-chr
Sample Name:
Data Collected on:
nmr500c.chem.buffalo.edu-inova500
Archive directory:
Sample directory:
FidFile: PROTON
Pulse Sequence: PROTON (s2pul)
Solvent: cdcl3
Data collected on: Nov 17 2018
Temp. 25.0 C / 298.1 K
Operator: Chemler
Relax. delay 1.000 sec
Pulse 45.0 degrees
Acq. time 2.048 sec
Width 7998.4 Hz
16 repetitions
OBSERVE H1, 499.9009993 MHz
DATA PROCESSING
FT size 32768
Total time 0 min 49 sec
7.529
7.515
7.500
7.469
7.453
7.441
7.284
7.269
7.260
7.221
7.206
7.194
7.179
7.165
5.031
5.017
5.011
3.101
3.082
3.063
3.055
2.834
2.816
2.803
2.776
2.748
2.740
2.721
2.712
2.702
2.694
2.384
2.378
2.371
2.365
2.358
1.571
0.007
ppm
9
8
7
6
5
4
3
2
1
0.99
2.0706
1.84025.56
0.98
1.00
2.04
1.01

ZK-584-carbon-chr

Sample Name:

Data Collected on:
nmr500c.chem.buffalo.edu-inova500
Archive directory:

Sample directory:

FidFile: CARBON

Pulse Sequence: CARBON (s2pul)
Solvent: cdcl3
Data collected on: Nov 17 2018

Temp. 25.0 C / 298.1 K
Operator: Chemler

Relax. delay 2.000 sec
Pulse 45.0 degrees
Acq. time 1.043 sec
Width 31421.8 Hz
10000 repetitions
OBSERVE C13, 125.7002083 MHz
DECOUPLE H1, 499.9034960 MHz
Power 40 dB
continuously on
WALTZ-16 modulated
DATA PROCESSING
Line broadening 0.5 Hz
FT size 65536
Total time 8 hr, 28 min

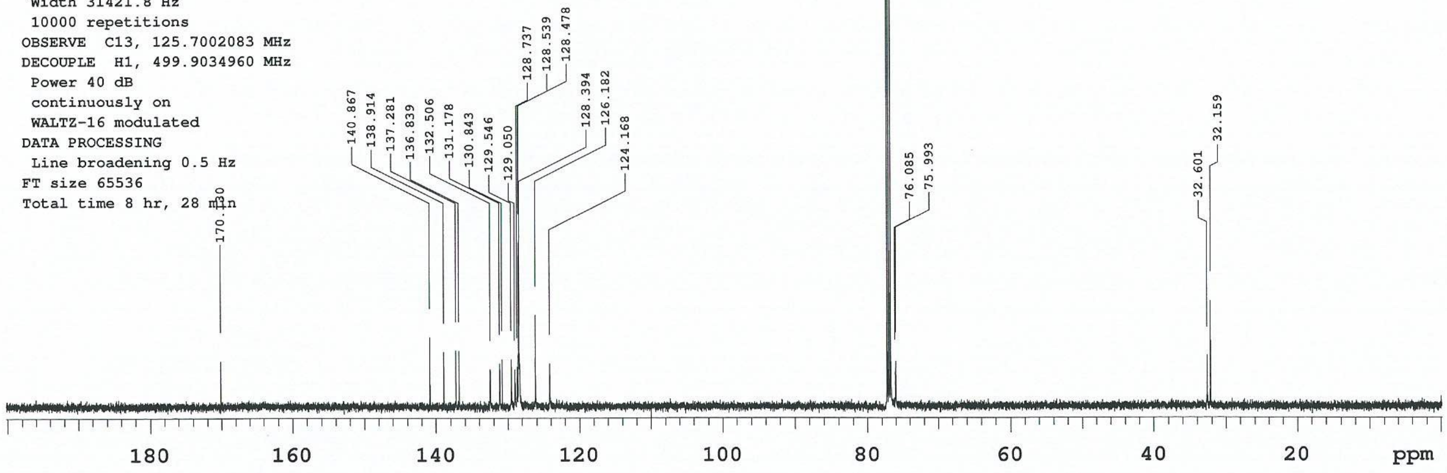

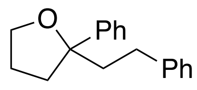

Ph
Ph

8a

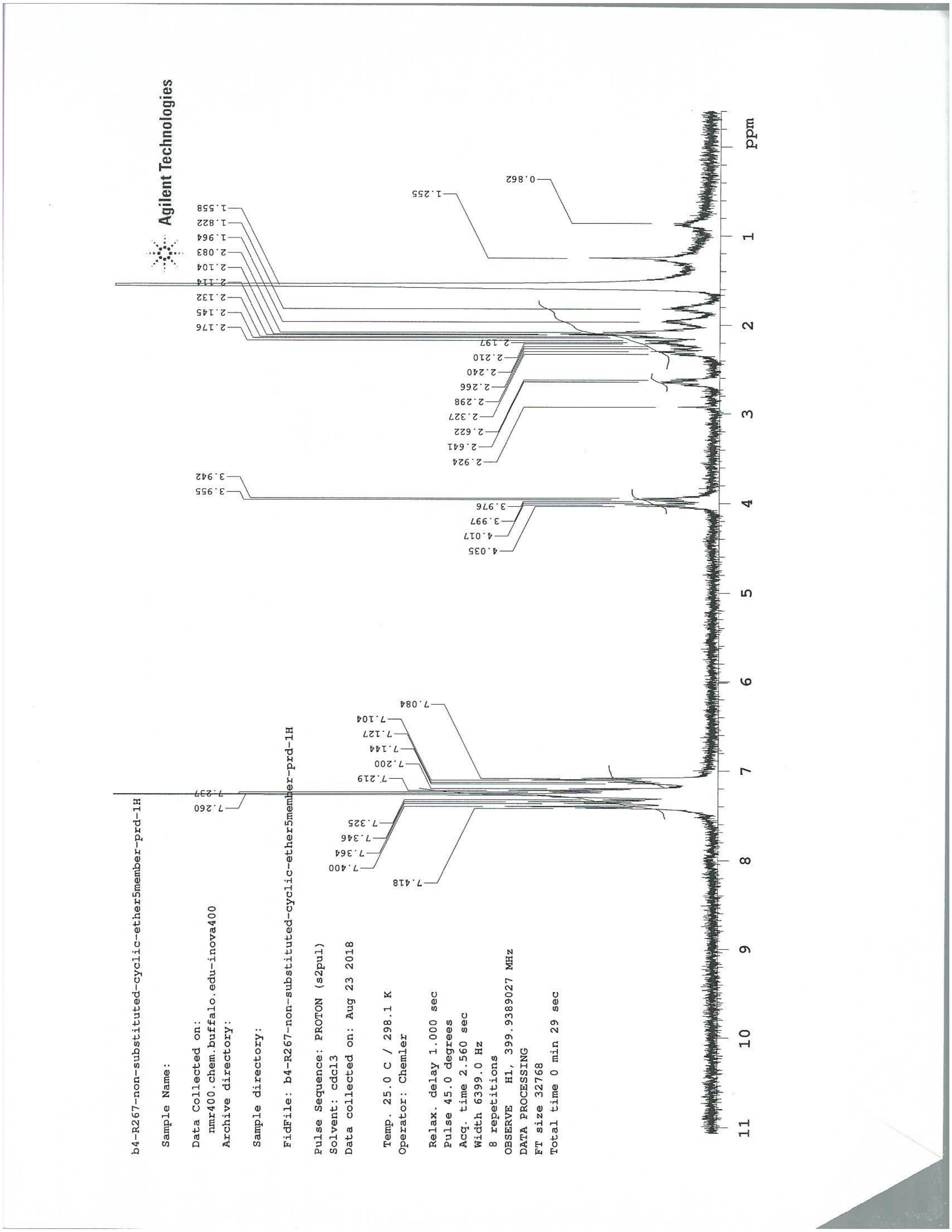

Agilent Technologies
b4-R267-non-substituted-cyclic-ether5member-prd-1H
Sample Name:
Data Collected on:
nmr400.chem.buffalo.edu-inova400
Archive directory:
Sample directory:
FidFile: b4-R267-non-substituted-cyclic-ether5member-prd-1H
Pulse Sequence: PROTON (s2pul)
Solvent: cdcl3
Data collected on: Aug 23 2018
Temp. 25.0 C / 298.1 K
Operator: Chemler
Relax. delay 1.000 sec
Pulse 45.0 degrees
Acq. time 2.560 sec
Width 6399.0 Hz
8 repetitions
OBSERVE H1, 399.9389027 MHz
DATA PROCESSING
FT size 32768
Total time 0 min 29 sec
7.418
7.400
7.364
7.346
7.325
7.260
7.237
7.219
7.200
7.144
7.127
7.104
7.084
4.035
4.017
3.997
3.976
3.955
3.942
2.924
2.641
2.622
2.327
2.298
2.266
2.240
2.210
2.197
2.176
2.145
2.132
2.114
2.104
2.083
1.964
1.822
1.558
1.255
0.862
ppm
11
10
9
8
7
6
5
4
3
2
1

ZK-624-HPLC-26min

Sample Name:

Data Collected on:
nmr500c.chem.buffalo.edu-inova500
Archive directory:

Sample directory:

FidFile: CARBON

Pulse Sequence: CARBON (s2pul)
Solvent: cdcl3
Data collected on: Aug 23 2018

Temp. 25.0 C / 298.1 K
Operator: Chemler

Relax. delay 1.000 sec
Pulse 45.0 degrees
Acq. time 1.043 sec
Width 31421.8 Hz
2044 repetitions
OBSERVE C13, 125.7002073 MHz
DECOUPLE H1, 499.9034960 MHz
Power 40 dB
continuously on
WALTZ-16 modulated
DATA PROCESSING
Line broadening 0.5 Hz
FT size 65536
Total time 703 hr, 37 min

Agilent Technologies

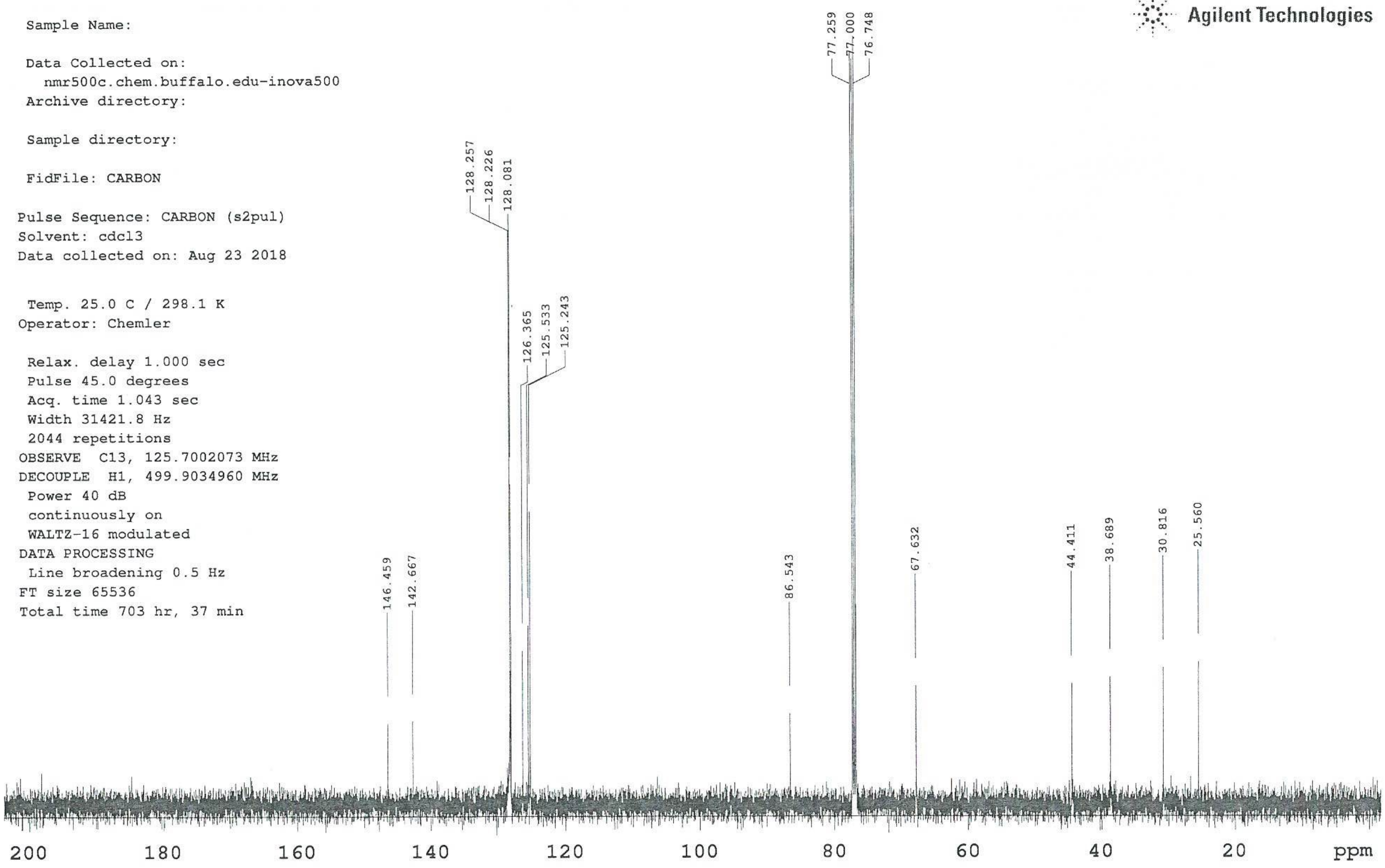

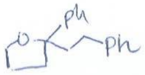

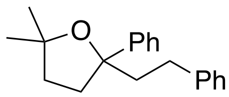

O
Ph
Ph

8b

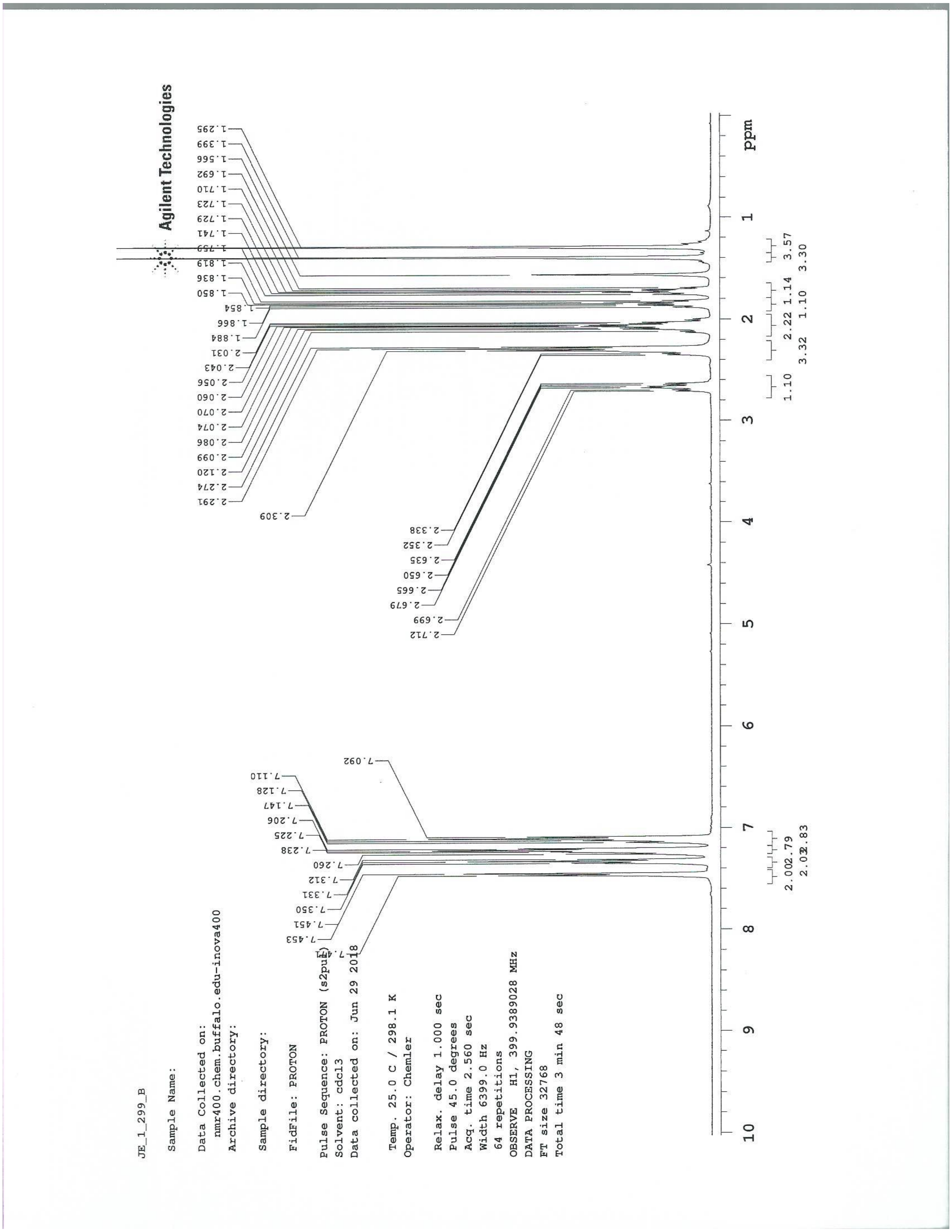

Agilent Technologies
JE_1_299_B
Sample Name:
Data Collected on:
nmr400.chem.buffalo.edu-inova400
Archive directory:
Sample directory:
FidFile: PROTON
Pulse Sequence: PROTON (s2pul)
Solvent: cdcl3
Data collected on: Jun 29 2018
Temp. 25.0 C / 298.1 K
Operator: Chemler
Relax. delay 1.000 sec
Pulse 45.0 degrees
Acq. time 2.560 sec
Width 6399.0 Hz
64 repetitions
OBSERVE H1, 399.9389028 MHz
DATA PROCESSING
FT size 32768
Total time 3 min 48 sec
1.295
1.399
1.566
1.692
1.710
1.723
1.729
1.741
1.759
1.819
1.836
1.850
1.854
1.866
1.884
2.031
2.043
2.056
2.060
2.070
2.074
2.086
2.099
2.120
2.274
2.291
2.309
2.338
2.352
2.635
2.650
2.665
2.679
2.699
2.712
7.092
7.110
7.128
7.147
7.206
7.225
7.238
7.260
7.312
7.331
7.350
7.451
7.453
7.471
3.57
3.30
1.14
1.10
2.22
3.32
1.10
2.00
2.79
2.03
2.83
ppm
1
2
3
4
5
6
7
8
9
10

tube-2-5OTBDPS-JC-after-TBAF-crude

Sample Name:

Data Collected on:
nmr400.chem.buffalo.edu-inova400
Archive directory:

Sample directory:

FidFile: CARBON

Pulse Sequence: CARBON (s2pul)
Solvent: cdcl3
Data collected on: Jul 1 2018

Temp. 25.0 C / 298.1 K
Operator: Chemler

Relax. delay 1.000 sec
Pulse 45.0 degrees
Acq. time 1.303 sec
Width 25141.4 Hz
2224 repetitions
OBSERVE C13, 100.5647177 MHz
DECOUPLE H1, 399.9409068 MHz
Power 33 dB
continuously on
WALTZ-16 modulated
DATA PROCESSING
Line broadening 0.5 Hz
FT size 65536
Total time 64 hr, 13 min

Agilent Technologies

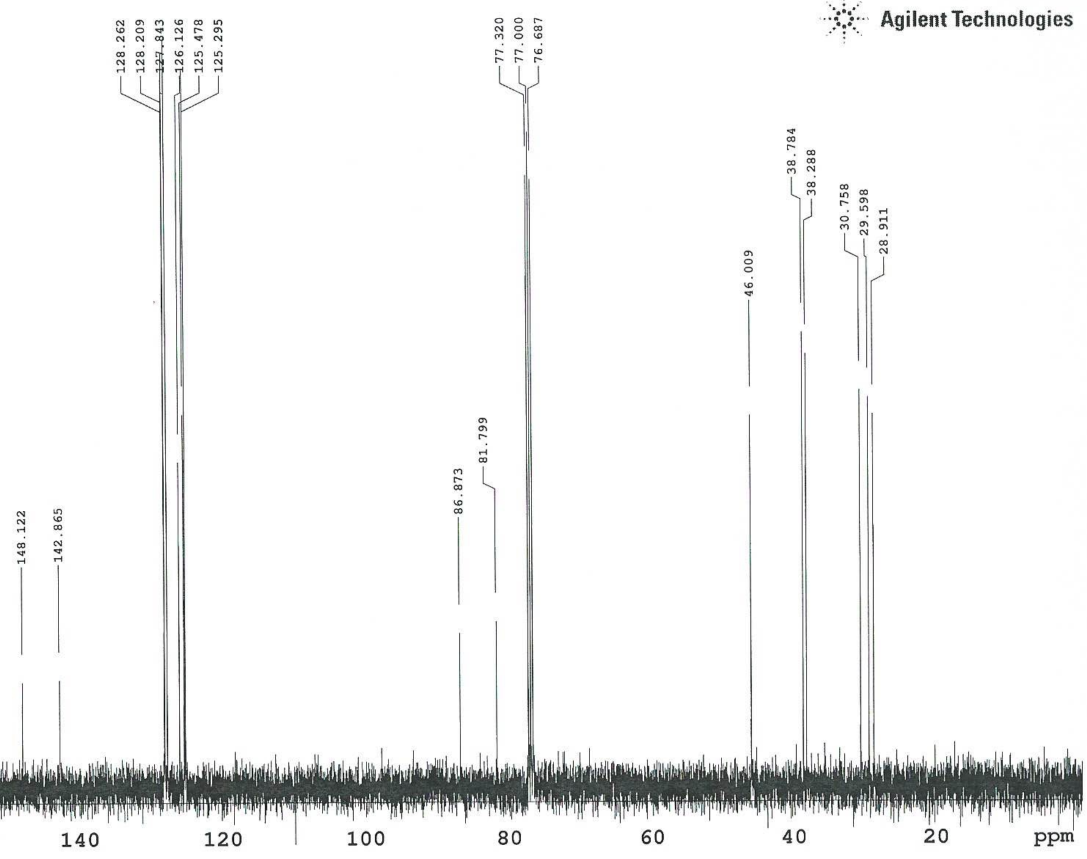

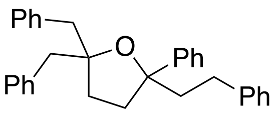
Ph
O
Ph
Ph
Ph

8c

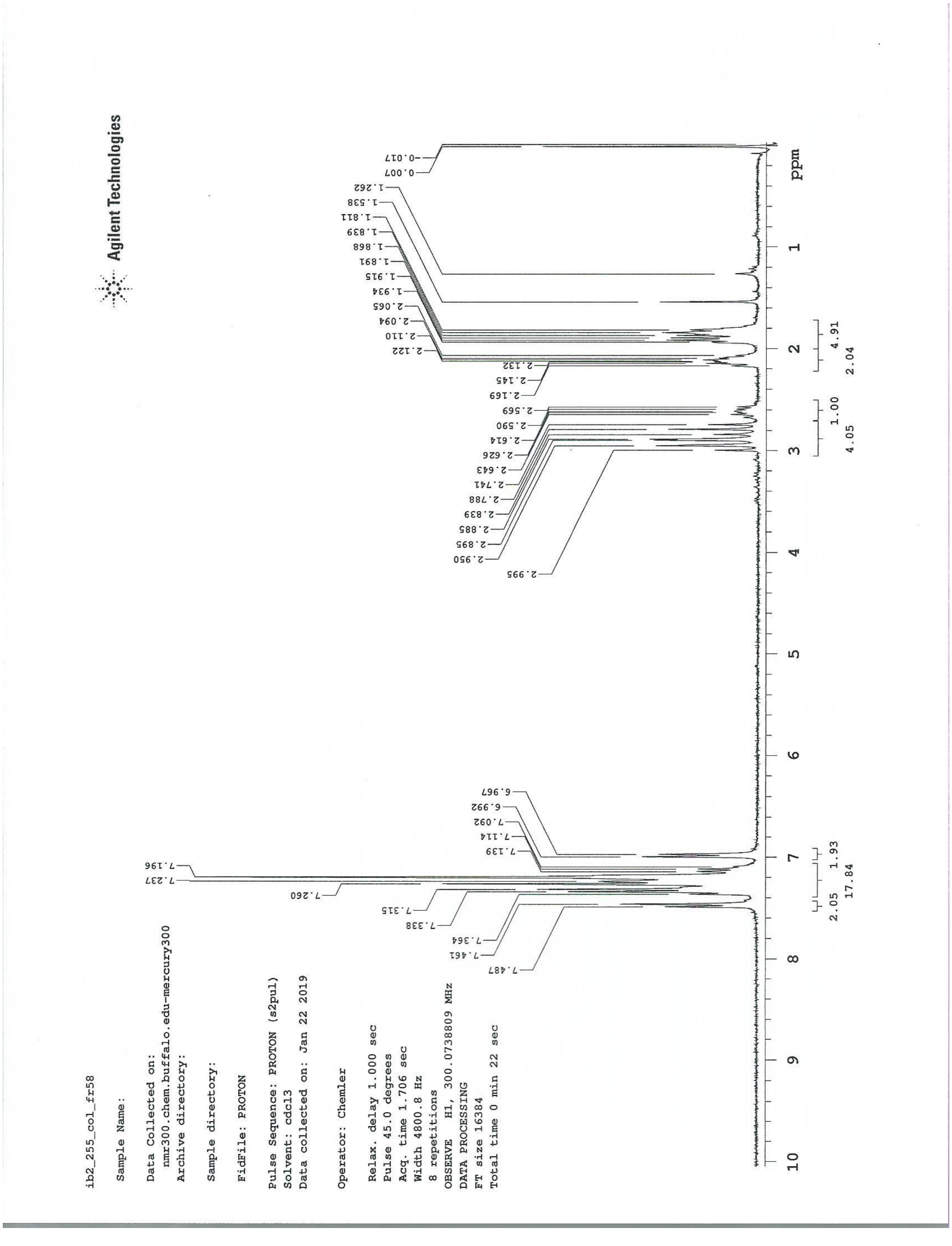
Agilent Technologies
ib2_255_col_fr58
Sample Name:
Data Collected on:
nmr300.chem.buffalo.edu-mercury300
Archive directory:
Sample directory:
FidFile: PROTON
Pulse Sequence: PROTON (s2pul)
Solvent: cdcl3
Data collected on: Jan 22 2019
Operator: Chemler
Relax. delay 1.000 sec
Pulse 45.0 degrees
Acq. time 1.706 sec
Width 4800.8 Hz
8 repetitions
OBSERVE H1, 300.0738809 MHz
DATA PROCESSING
FT size 16384
Total time 0 min 22 sec
7.487
7.461
7.364
7.338
7.315
7.260
7.237
7.196
7.139
7.114
7.092
6.992
6.967
2.995
2.950
2.895
2.885
2.839
2.788
2.741
2.643
2.626
2.614
2.590
2.569
2.169
2.145
2.132
2.122
2.110
2.094
2.065
1.934
1.915
1.891
1.868
1.839
1.811
1.538
1.262
0.007
-0.017
2.05
17.84
1.93
4.05
1.00
2.04
4.91
ppm
10
9
8
7
6
5
4
3
2
1

ib2_255_col_fr58

Sample Name:

Data Collected on:
nmr300.chem.buffalo.edu-mercury300
Archive directory:

Sample directory:

FidFile: CARBON

Pulse Sequence: CARBON (s2pul)
Solvent: cdcl3
Data collected on: Jan 22 2019

Operator: Chemler

Relax. delay 2.000 sec
Pulse 45.0 degrees
Acq. time 0.868 sec
Width 18867.9 Hz
3240 repetitions
OBSERVE C13, 75.4536377 MHz
DECOUPLE H1, 300.0754430 MHz
Power 37 dB
continuously on
WALTZ-16 modulated
DATA PROCESSING
Line broadening 0.5 Hz
FT size 32768
Total time 7992 hr, 16 min

Agilent Technologies

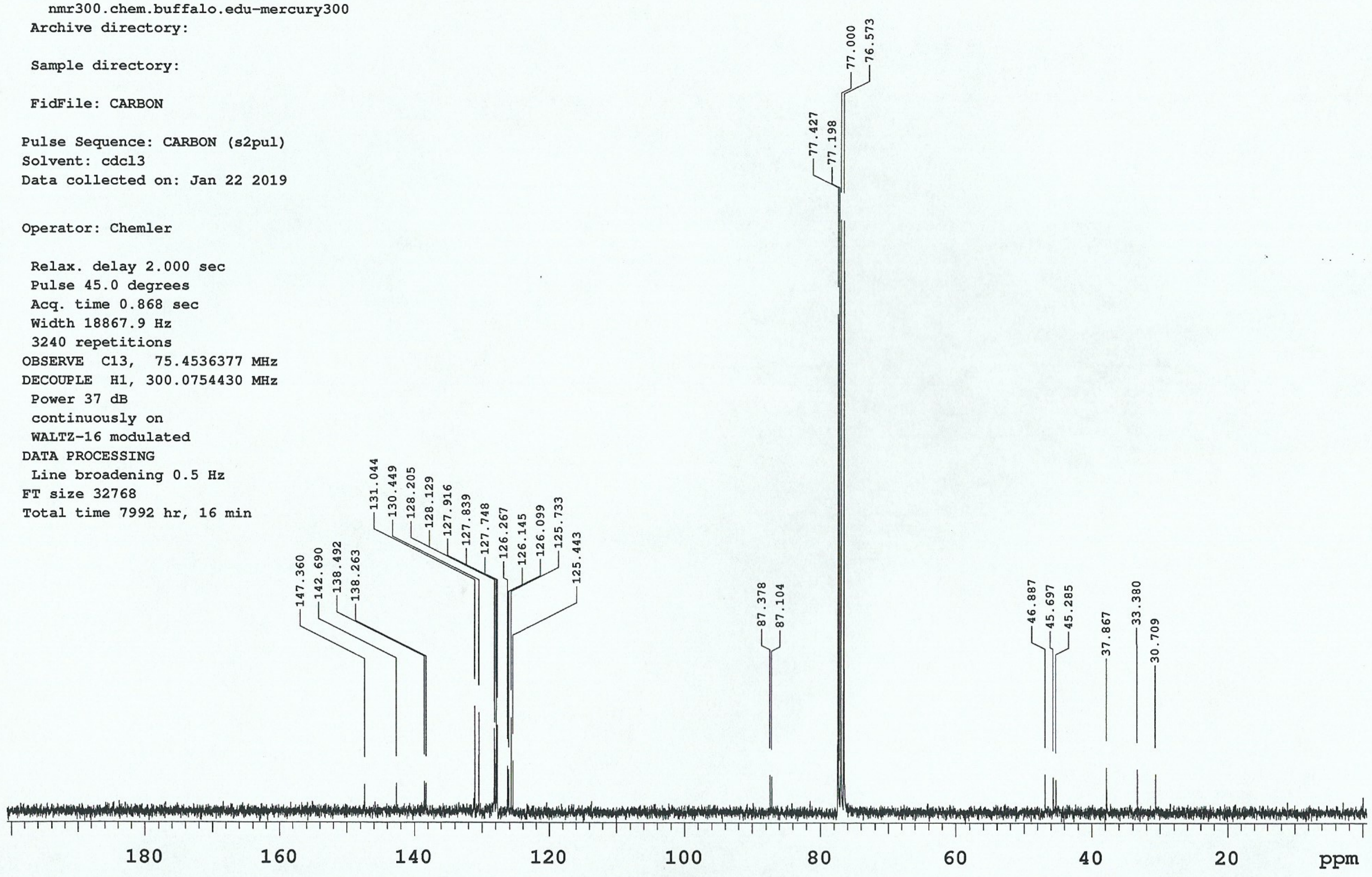

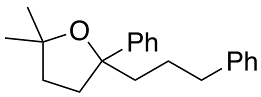

8d

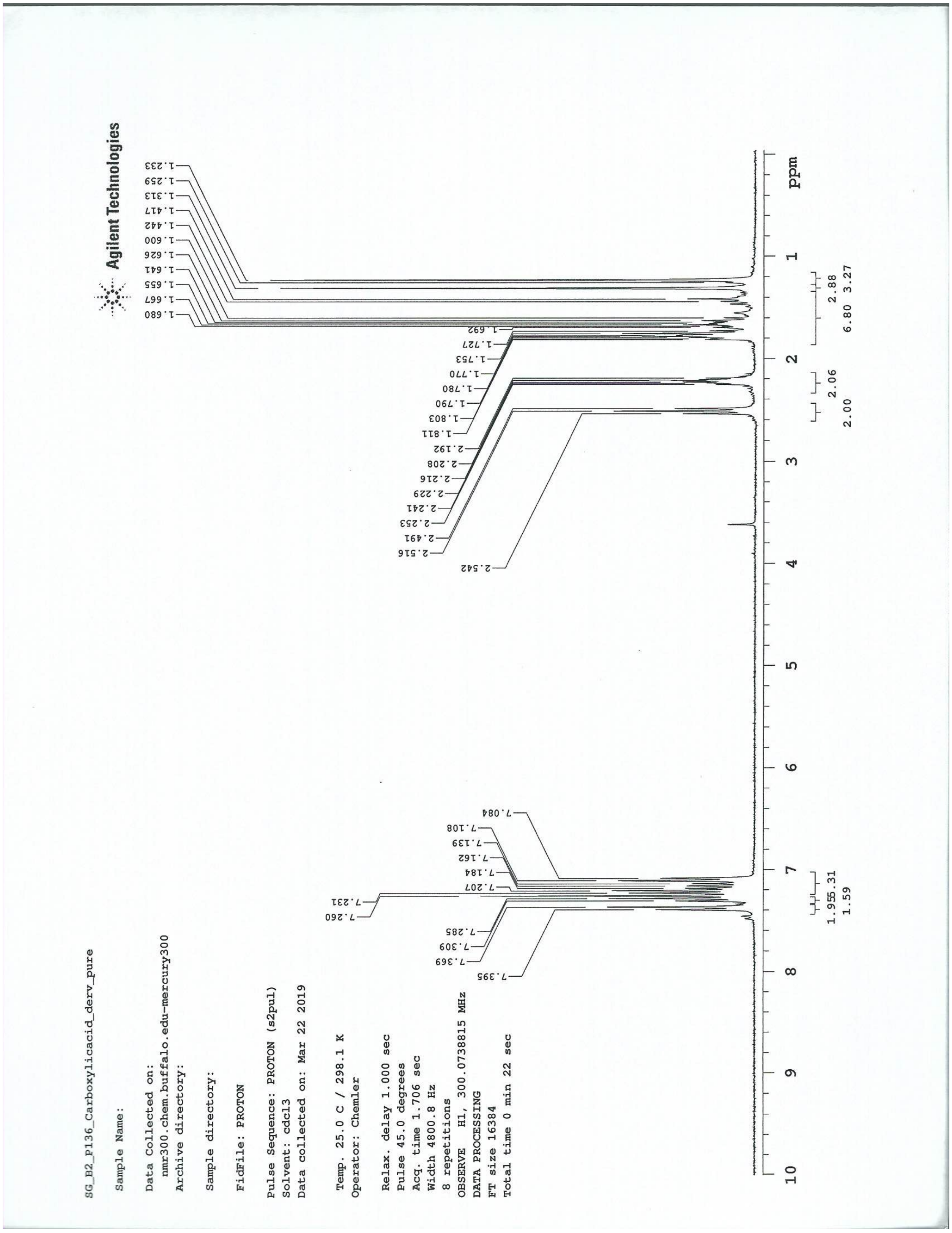

Agilent Technologies
SG_B2_P136_Carboxylicacid_derv_pure
Sample Name:
Data Collected on:
nmr300.chem.buffalo.edu-mercury300
Archive directory:
Sample directory:
FidFile: PROTON
Pulse Sequence: PROTON (s2pul)
Solvent: cdcl3
Data collected on: Mar 22 2019
Temp. 25.0 C / 298.1 K
Operator: Chemler
Relax. delay 1.000 sec
Pulse 45.0 degrees
Acq. time 1.706 sec
Width 4800.8 Hz
8 repetitions
OBSERVE H1, 300.0738815 MHz
DATA PROCESSING
FT size 16384
Total time 0 min 22 sec
1.233
1.259
1.313
1.417
1.442
1.600
1.626
1.641
1.655
1.667
1.680
1.692
1.727
1.753
1.770
1.780
1.790
1.803
1.811
2.192
2.208
2.216
2.229
2.241
2.253
2.491
2.516
2.542
7.084
7.108
7.139
7.162
7.184
7.207
7.231
7.260
7.285
7.309
7.369
7.395
1.95
5.31
1.59
2.00
2.06
6.80
2.88
3.27
ppm
10
9
8
7
6
5
4
3
2
1

SG_B2_p136_Rxn1_cyano

Sample Name:

Data Collected on:
nmr300.chem.buffalo.edu-mercury300
Archive directory:

Sample directory:

FidFile: CARBON

Pulse Sequence: CARBON (s2pul)
Solvent: cdcl3
Data collected on: Mar 23 2019

Temp. 25.0 C / 298.1 K
Operator: Chemler

Relax. delay 2.000 sec
Pulse 45.0 degrees
Acq. time 0.868 sec
Width 18867.9 Hz
520 repetitions
OBSERVE C13, 75.4536377 MHz
DECOUPLE H1, 300.0754430 MHz
Power 37 dB
continuously on
WALTZ-16 modulated
DATA PROCESSING
Line broadening 0.5 Hz
FT size 32768
Total time 79922 hr, 45 min

Agilent Technologies

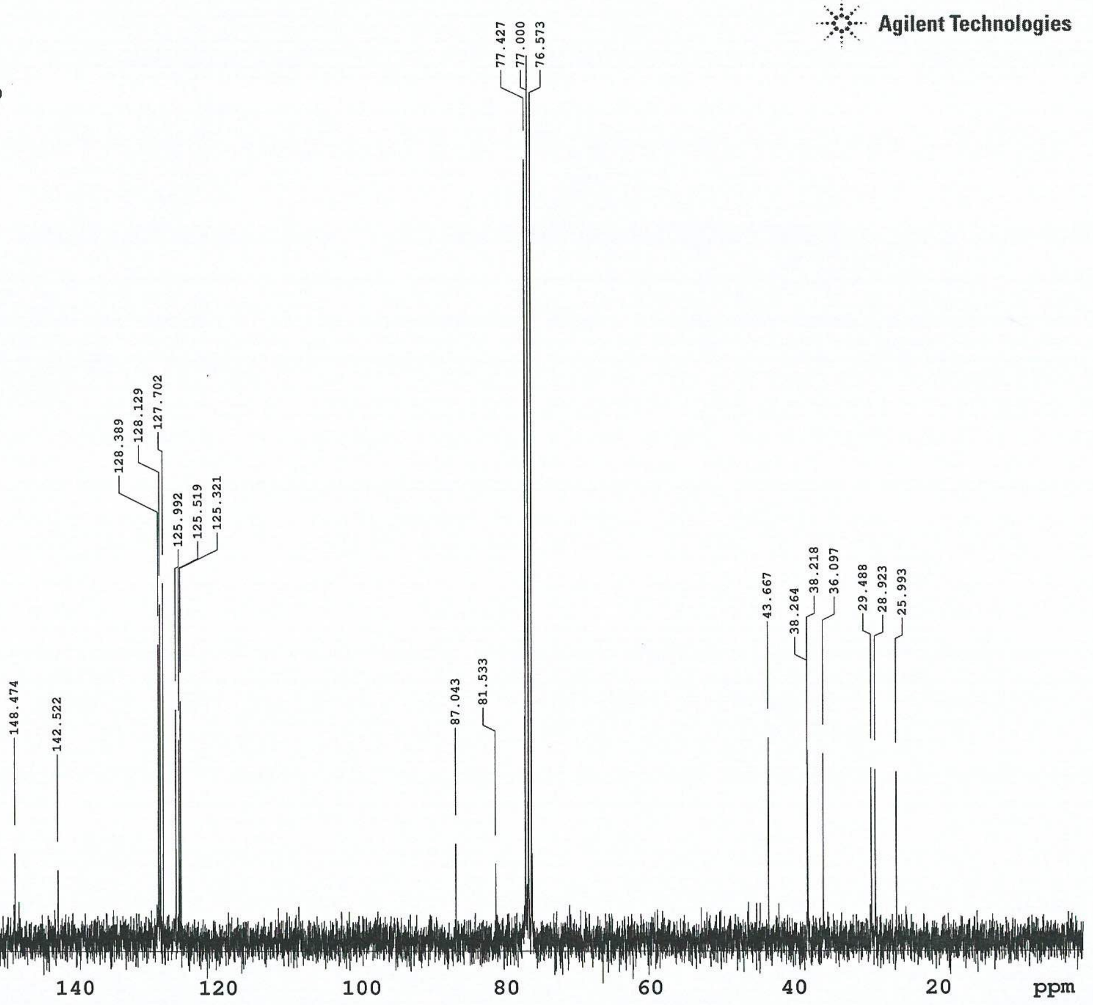

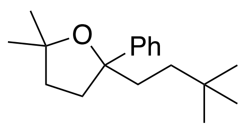
O
Ph

8e

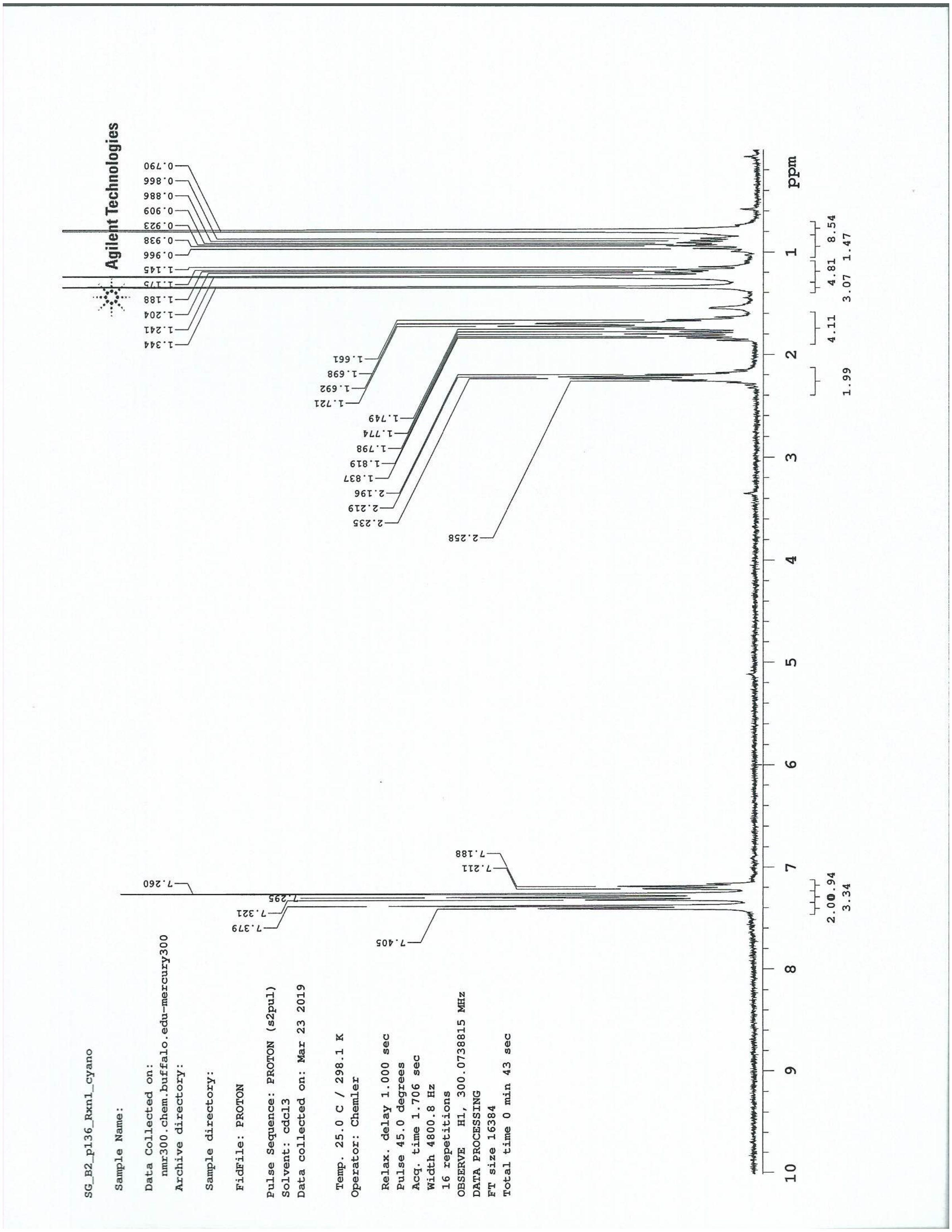
SG_B2_p136_Rxn1_cyano
Sample Name:
Data Collected on:
nmr300.chem.buffalo.edu-mercury300
Archive directory:
Sample directory:
FidFile: PROTON
Pulse Sequence: PROTON (s2pul)
Solvent: cdcl3
Data collected on: Mar 23 2019
Temp. 25.0 C / 298.1 K
Operator: Chemler
Relax. delay 1.000 sec
Pulse 45.0 degrees
Acq. time 1.706 sec
Width 4800.8 Hz
16 repetitions
OBSERVE H1, 300.0738815 MHz
DATA PROCESSING
FT size 16384
Total time 0 min 43 sec
Agilent Technologies
0.790
0.866
0.886
0.909
0.923
0.938
0.966
1.145
1.175
1.188
1.204
1.241
1.344
1.661
1.698
1.692
1.721
1.749
1.774
1.798
1.819
1.837
2.196
2.219
2.235
2.258
7.188
7.211
7.260
7.295
7.321
7.379
7.405
8.54
4.81
1.47
3.07
4.11
1.99
2.00
0.94
3.34
ppm
10
9
8
7
6
5
4
3
2
1

SG_B2_p136_Rxn1_cyano

Sample Name:

Data Collected on:
nmr300.chem.buffalo.edu-mercury300
Archive directory:

Sample directory:

FidFile: CARBON

Pulse Sequence: CARBON (s2pul)
Solvent: cdcl3
Data collected on: Mar 23 2019

Temp. 25.0 C / 298.1 K
Operator: Chemler

Relax. delay 2.000 sec
Pulse 45.0 degrees
Acq. time 0.868 sec
Width 18867.9 Hz
2696 repetitions
OBSERVE C13, 75.4536377 MHz
DECOUPLE H1, 300.0754430 MHz
Power 37 dB
continuously on
WALTZ-16 modulated
DATA PROCESSING
Line broadening 0.5 Hz
FT size 32768
Total time 799227 hr, 35 min

Agilent Technologies

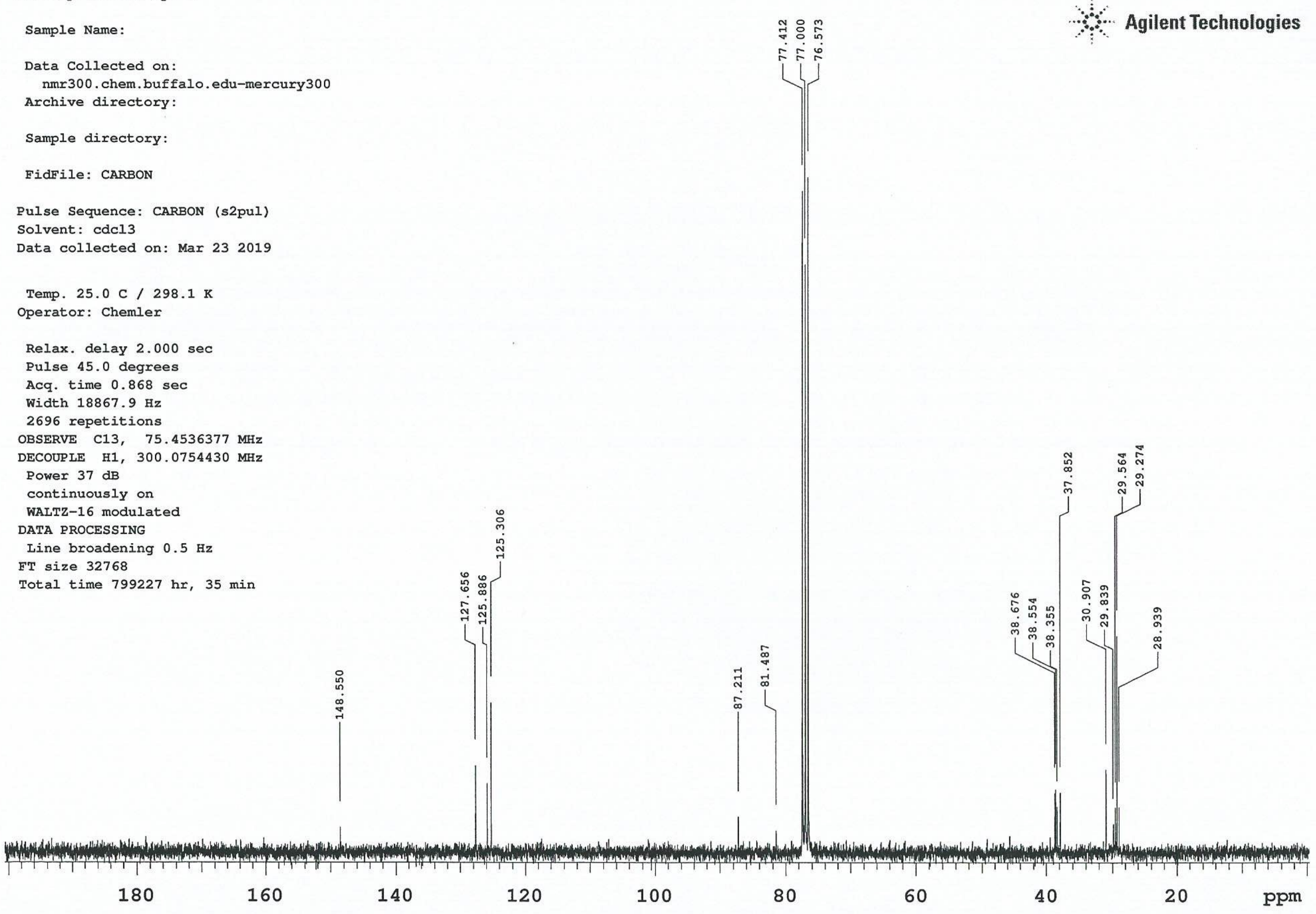

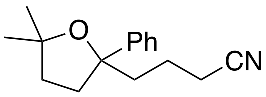
O
Ph
CN

8f

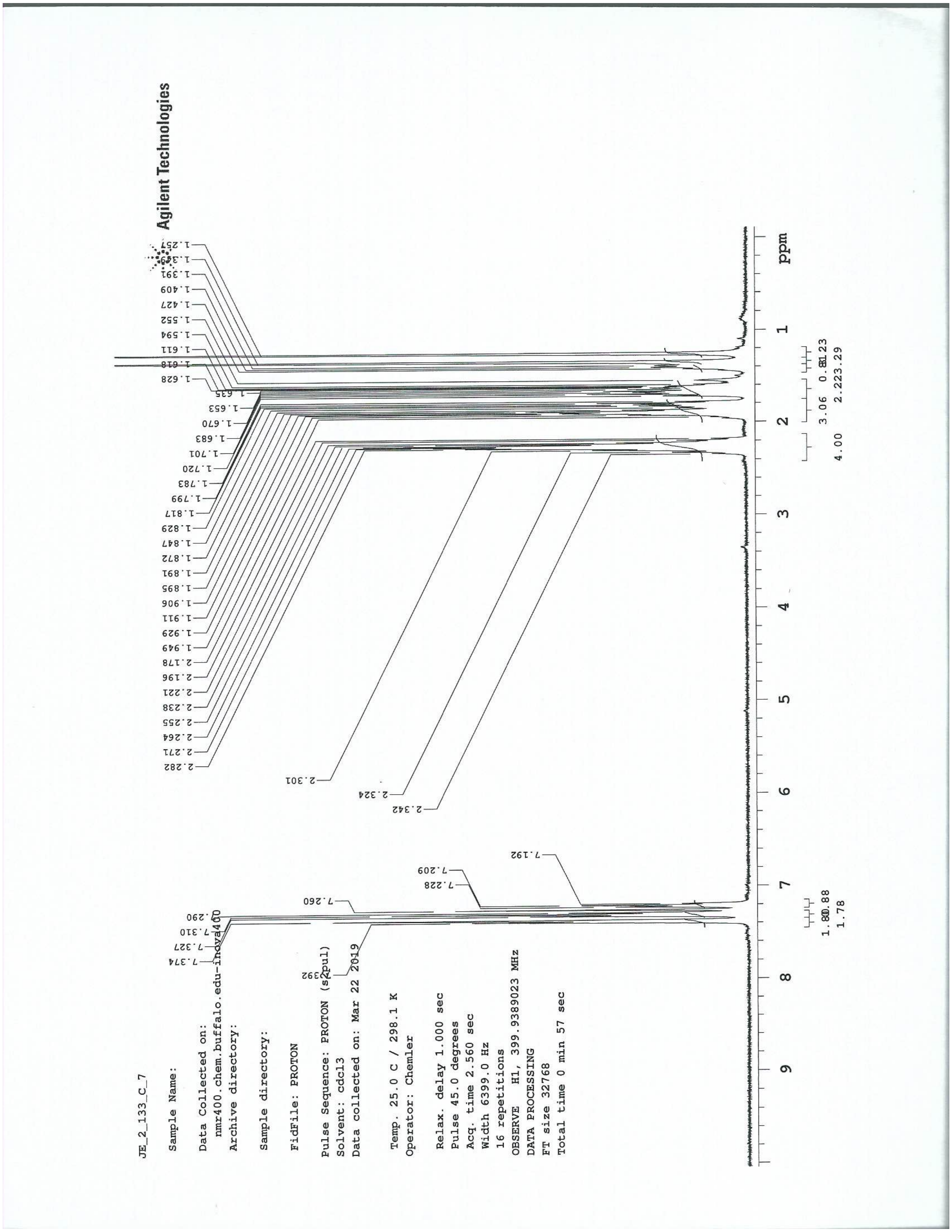
JE_2_133_C_7
Sample Name:
Data Collected on:
nmr400.chem.buffalo.edu-inova400
Archive directory:
Sample directory:
FidFile: PROTON
Pulse Sequence: PROTON (s2pul)
Solvent: cdcl3
Data collected on: Mar 22 2019
Temp. 25.0 C / 298.1 K
Operator: Chemler
Relax. delay 1.000 sec
Pulse 45.0 degrees
Acq. time 2.560 sec
Width 6399.0 Hz
16 repetitions
OBSERVE H1, 399.9389023 MHz
DATA PROCESSING
FT size 32768
Total time 0 min 57 sec
Agilent Technologies
ppm

Parameter	Value
Spectrometer	inova
Solvent	cdcl3
Temperature	25.0
Pulse Sequence	s2pul
Experiment	1D
Probe	HCN
Receiver Gain	30
Relaxation Delay	3.0000
Pulse Width	16.1000
Presaturation Frequency	
Acquisition Time	1.3033
Acquisition Date	2016-09-22T10:13:46
Spectrometer Frequency	100.58
Spectral Width	25141.4
Lowest Frequency	-1507.6
Nucleus	13C
Acquired Size	32768
Spectral Size	65536

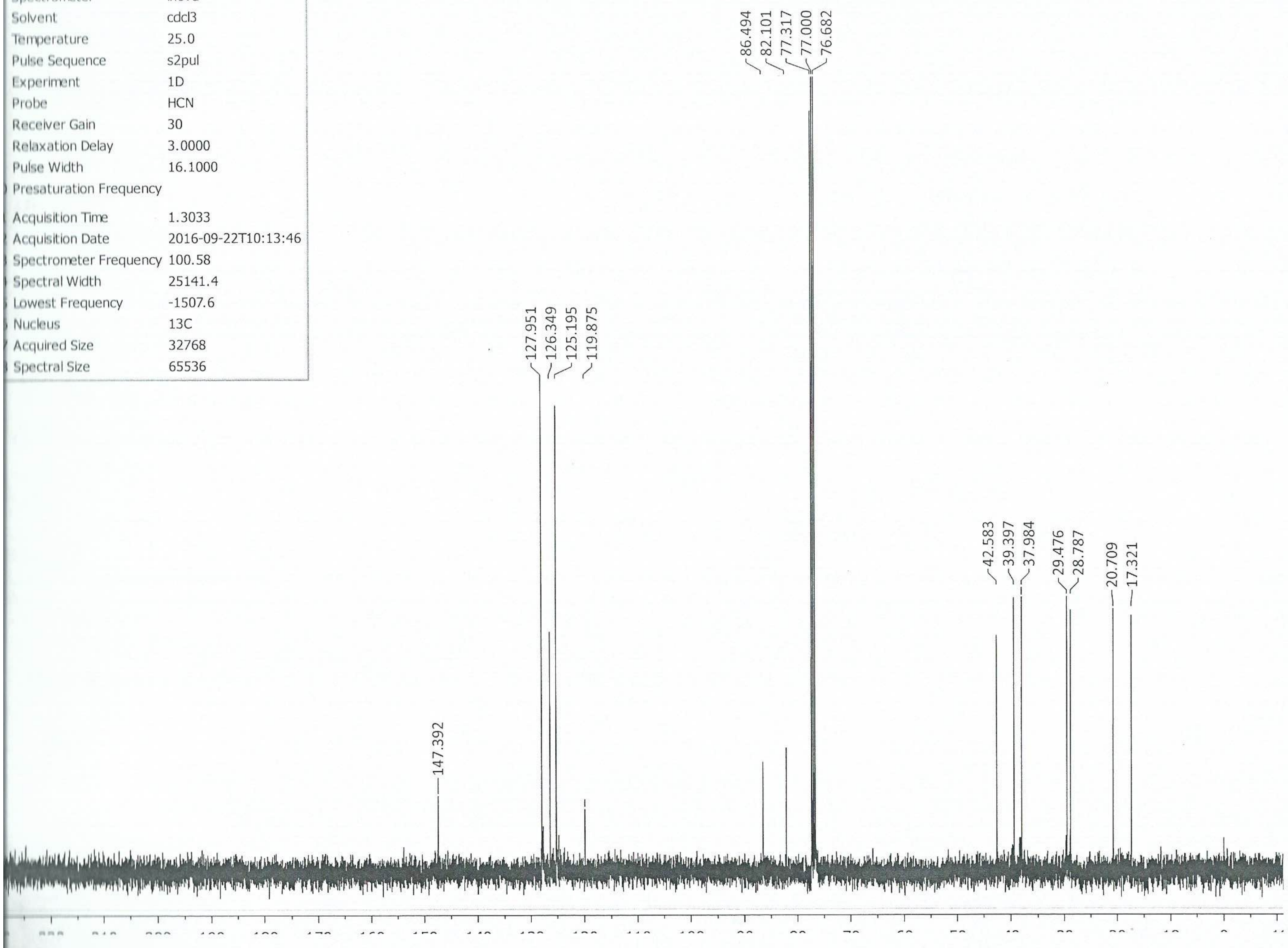

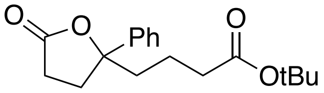
O
O
Ph
O
OtBu

8g

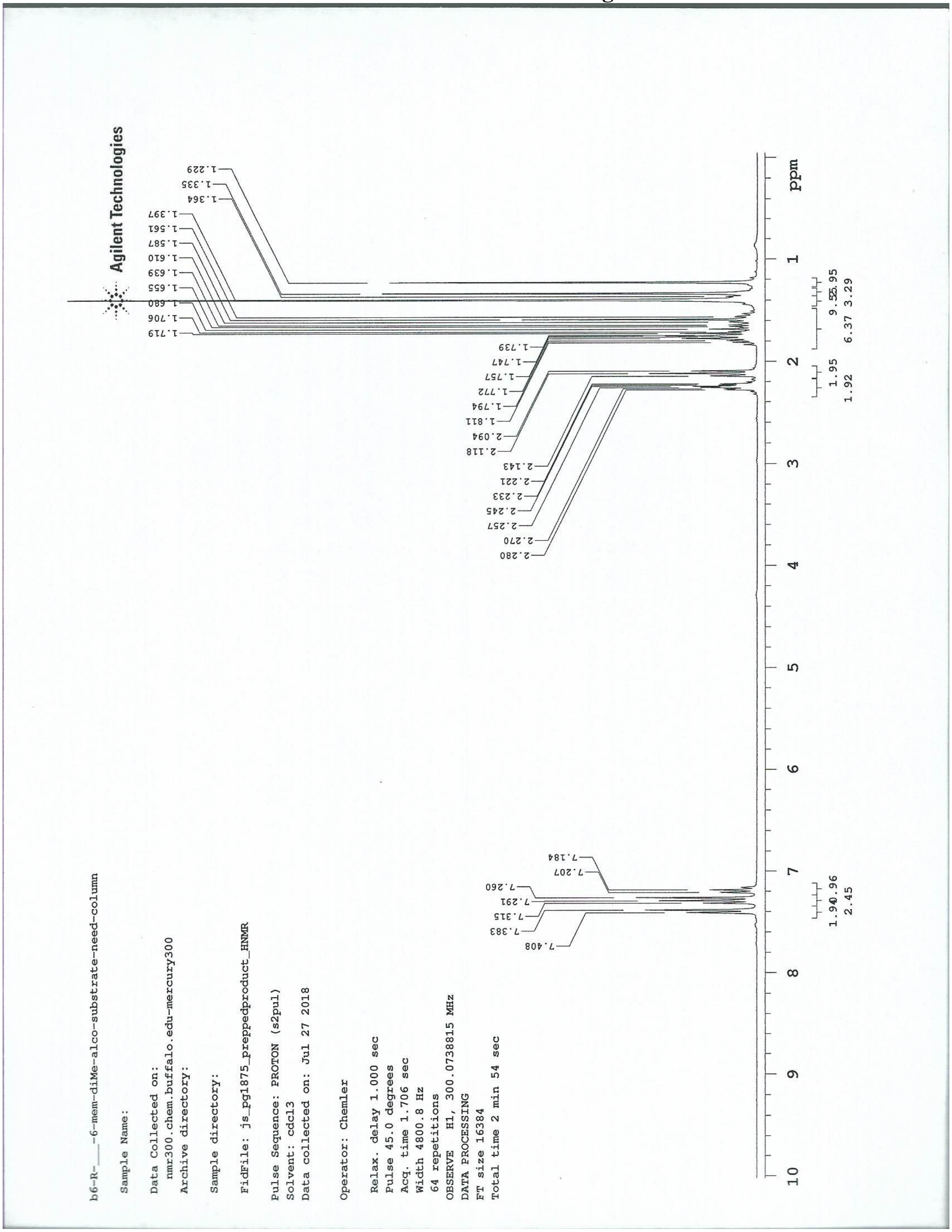
Agilent Technologies
b6-R-___-6-mem-diMe-alco-substrate-need-column
Sample Name:
Data Collected on:
nmr300.chem.buffalo.edu-mercury300
Archive directory:
Sample directory:
FidFile: js_pg1875_preppedproduct_HNMR
Pulse Sequence: PROTON (s2pul)
Solvent: cdcl3
Data collected on: Jul 27 2018
Operator: Chemler
Relax. delay 1.000 sec
Pulse 45.0 degrees
Acq. time 1.706 sec
Width 4800.8 Hz
64 repetitions
OBSERVE H1, 300.0738815 MHz
DATA PROCESSING
FT size 16384
Total time 2 min 54 sec
ppm

b6-R-___-6-mem-diMe-alco-substrate-need-column

Sample Name:

Data Collected on:
nmr300.chem.buffalo.edu-mercury300
Archive directory:

Sample directory:

FidFile: js_pg1875_preppedproduct_CNMR

Pulse Sequence: CARBON (s2pul)
Solvent: cdcl3
Data collected on: Jul 27 2018

Operator: Chemler

Relax. delay 2.000 sec
Pulse 45.0 degrees
Acq. time 0.868 sec
Width 18867.9 Hz
1912 repetitions
OBSERVE C13, 75.4536377 MHz
DECOUPLE H1, 300.0754430 MHz
Power 37 dB
continuously on
WALTZ-16 modulated
DATA PROCESSING
Line broadening 0.5 Hz
FT size 32768
Total time 7992 hr, 16 min

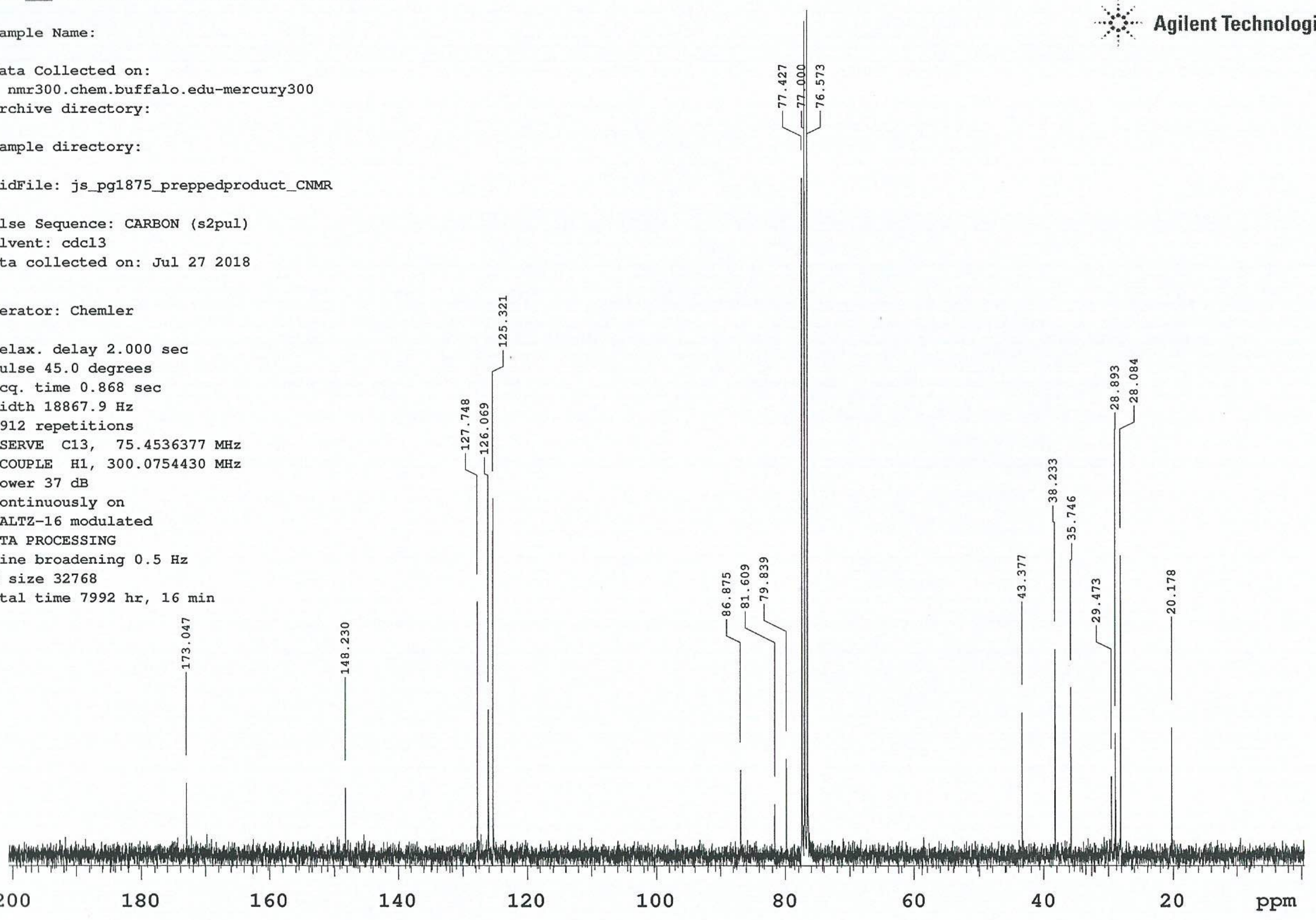

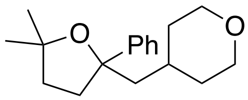

8h

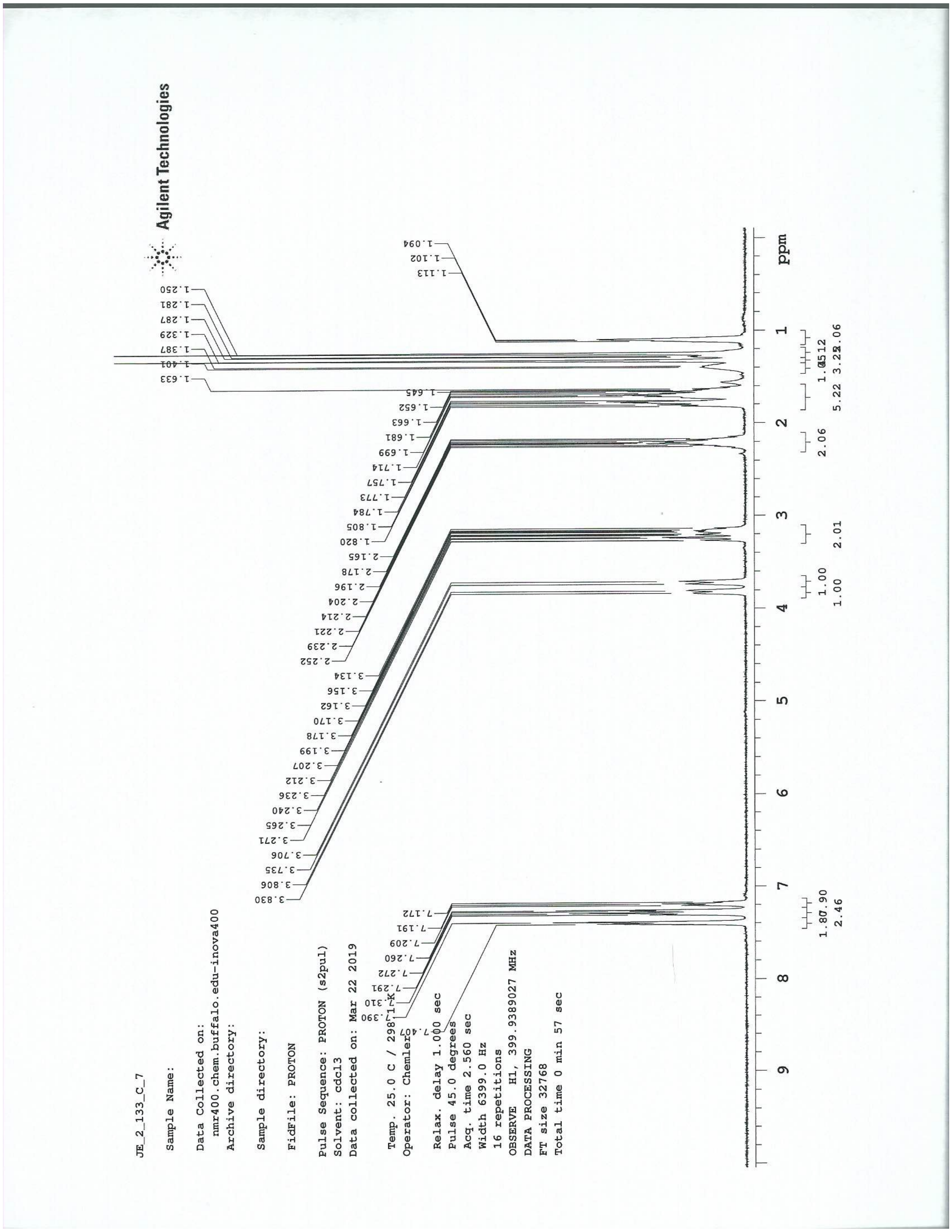

Agilent Technologies
JE_2_133_C_7
Sample Name:
Data Collected on:
nmr400.chem.buffalo.edu-inova400
Archive directory:
Sample directory:
FidFile: PROTON
Pulse Sequence: PROTON (s2pul)
Solvent: cdcl3
Data collected on: Mar 22 2019
Temp. 25.0 C / 298.1 K
Operator: Chemler
Relax. delay 1.000 sec
Pulse 45.0 degrees
Acq. time 2.560 sec
Width 6399.0 Hz
16 repetitions
OBSERVE H1, 399.9389027 MHz
DATA PROCESSING
FT size 32768
Total time 0 min 57 sec
7.407
7.390
7.310
7.291
7.272
7.260
7.209
7.191
7.172
3.830
3.806
3.735
3.706
3.271
3.265
3.240
3.236
3.212
3.207
3.199
3.178
3.170
3.162
3.156
3.134
2.252
2.239
2.221
2.214
2.204
2.196
2.178
2.165
1.820
1.805
1.784
1.773
1.757
1.714
1.699
1.681
1.663
1.652
1.645
1.633
1.401
1.387
1.329
1.287
1.281
1.250
1.113
1.102
1.094
ppm
2.46
1.00
1.00
2.01
2.06
5.22

JE_2_133_C_7

Sample Name:

Data Collected on:
nmr400.chem.buffalo.edu-inova400
Archive directory:

Sample directory:

FidFile: CARBON

Pulse Sequence: CARBON (s2pul)
Solvent: cdcl3
Data collected on: Mar 22 2019

Temp. 25.0 C / 298.1 K
Operator: Chemler

Relax. delay 2.000 sec
Pulse 45.0 degrees
Acq. time 1.303 sec
Width 25141.4 Hz
1848 repetitions
OBSERVE C13, 100.5647169 MHz
DECOUPLE H1, 399.9409068 MHz
Power 33 dB
continuously on
WALTZ-16 modulated
DATA PROCESSING
Line broadening 0.5 Hz
FT size 65536
Total time 9200 hr, 37 min

Agilent Technologies

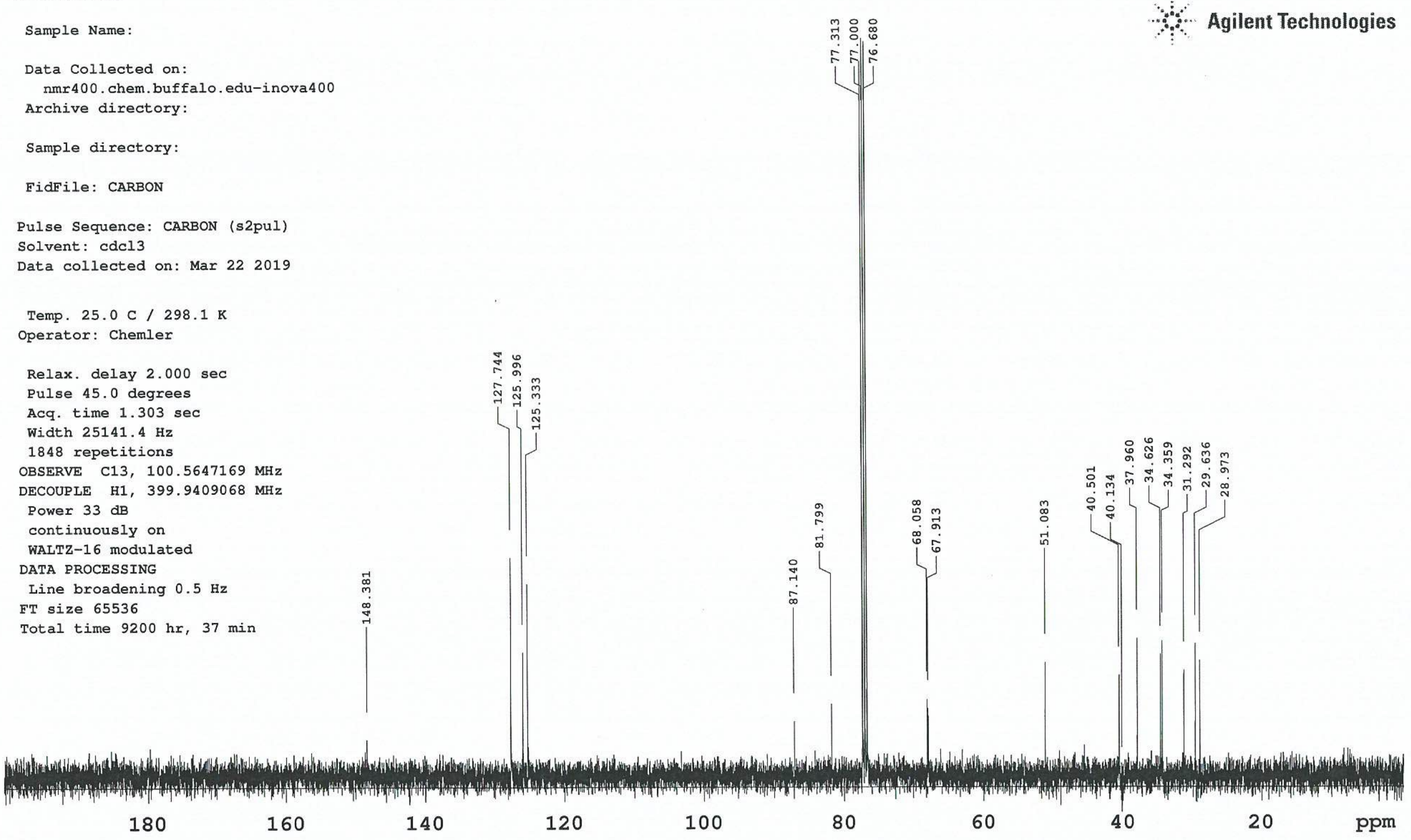

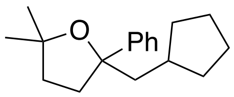

O
Ph

8i

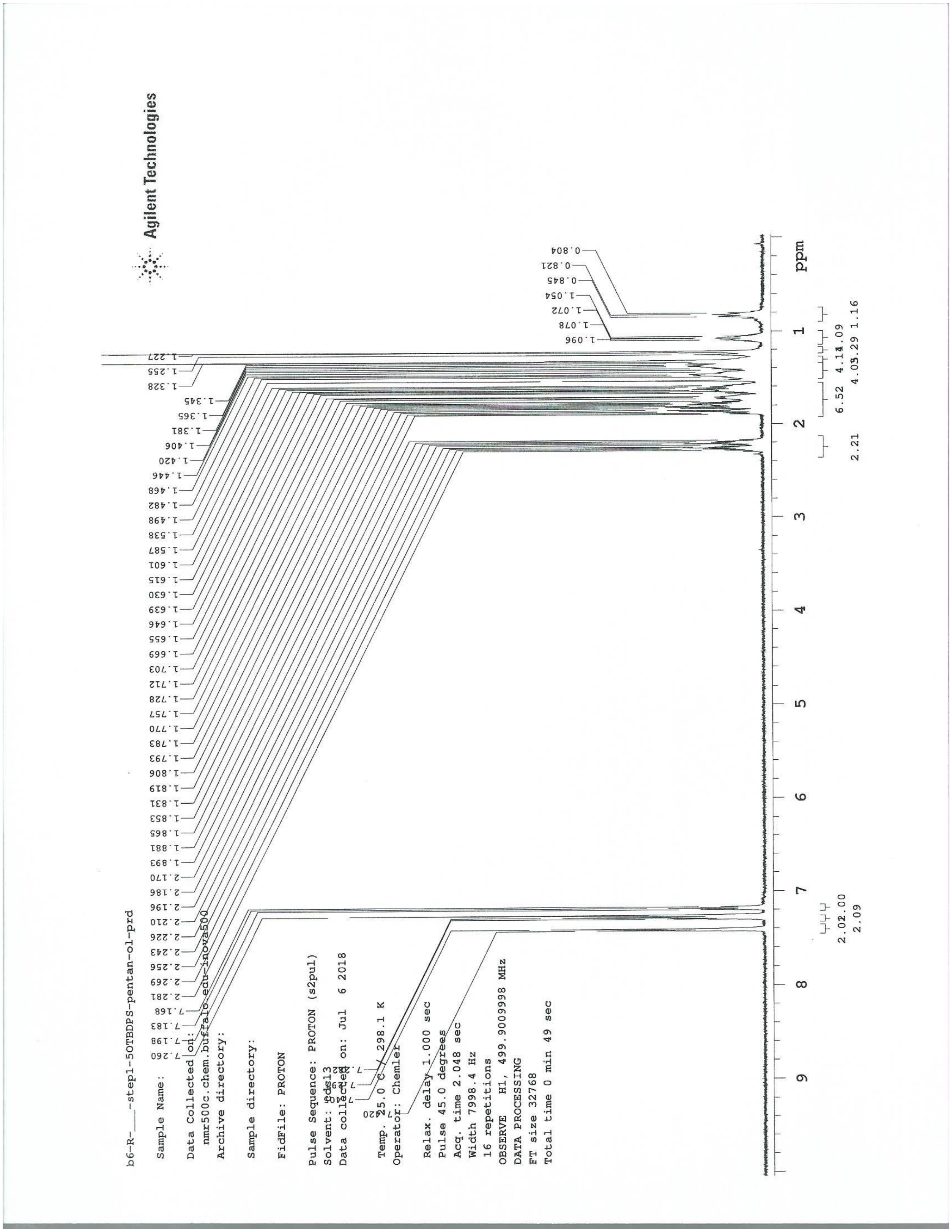

Agilent Technologies
b6-R-___-step1-5OTBDPS-pentan-ol-prd
Sample Name:
Data Collected on:
nmr500c.chem.buffalo.edu-inova500
Archive directory:
Sample directory:
FidFile: PROTON
Pulse Sequence: PROTON (s2pul)
Solvent: cdcl3
Data collected on: Jul 6 2018
Temp. 25.0 C / 298.1 K
Operator: Chemler
Relax. delay 1.000 sec
Pulse 45.0 degrees
Acq. time 2.048 sec
Width 7998.4 Hz
16 repetitions
OBSERVE H1, 499.9009998 MHz
DATA PROCESSING
FT size 32768
Total time 0 min 49 sec
7.260
7.198
7.183
7.168
2.281
2.269
2.256
2.243
2.226
2.210
2.196
2.186
2.170
1.893
1.881
1.865
1.853
1.831
1.819
1.806
1.793
1.783
1.770
1.757
1.728
1.712
1.703
1.669
1.655
1.646
1.639
1.630
1.615
1.601
1.587
1.538
1.498
1.482
1.468
1.446
1.420
1.406
1.381
1.365
1.345
1.328
1.255
1.227
1.096
1.078
1.072
1.054
0.845
0.821
0.804
2.02
2.00
2.09
2.21
6.52
4.11
1.09
4.03
3.29
1.16
9
8
7
6
5
4
3
2
1
ppm

SG_B2_P53_F30

Sample Name:

Data Collected on:
nmr300.chem.buffalo.edu-mercury300
Archive directory:

Sample directory:

FidFile: CARBON

Pulse Sequence: CARBON (s2pul)
Solvent: cdcl3
Data collected on: Jul 11 2018

Operator: Chemler

Relax. delay 2.000 sec
Pulse 45.0 degrees
Acq. time 0.868 sec
Width 18867.9 Hz
1304 repetitions
OBSERVE C13, 75.4536377 MHz
DECOUPLE H1, 300.0754430 MHz
Power 37 dB
continuously on
WALTZ-16 modulated
DATA PROCESSING
Line broadening 0.5 Hz
FT size 32768
Total time 79 hr, 55 min

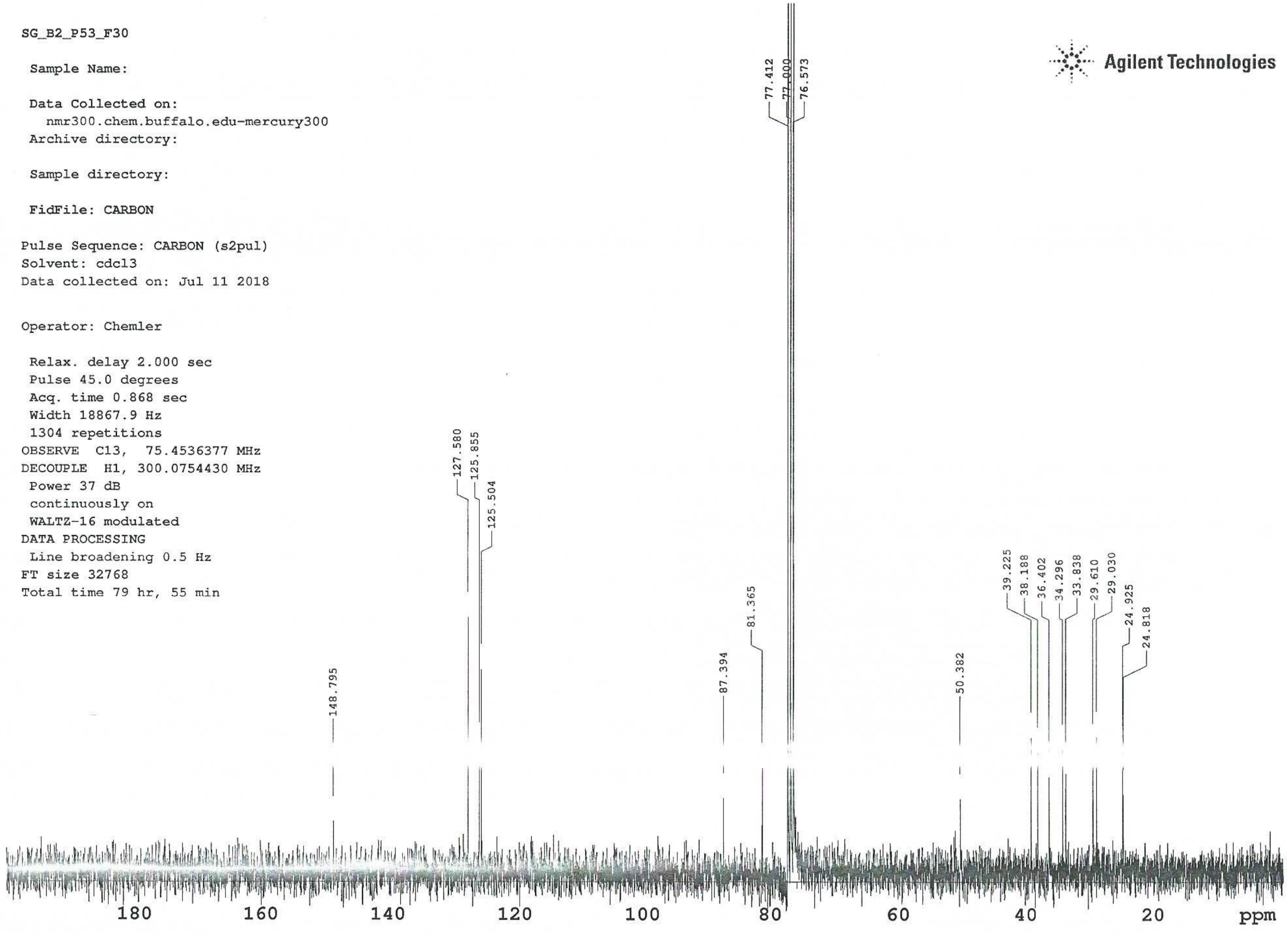

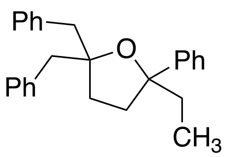
Ph
O
Ph
Ph
CH3

8j

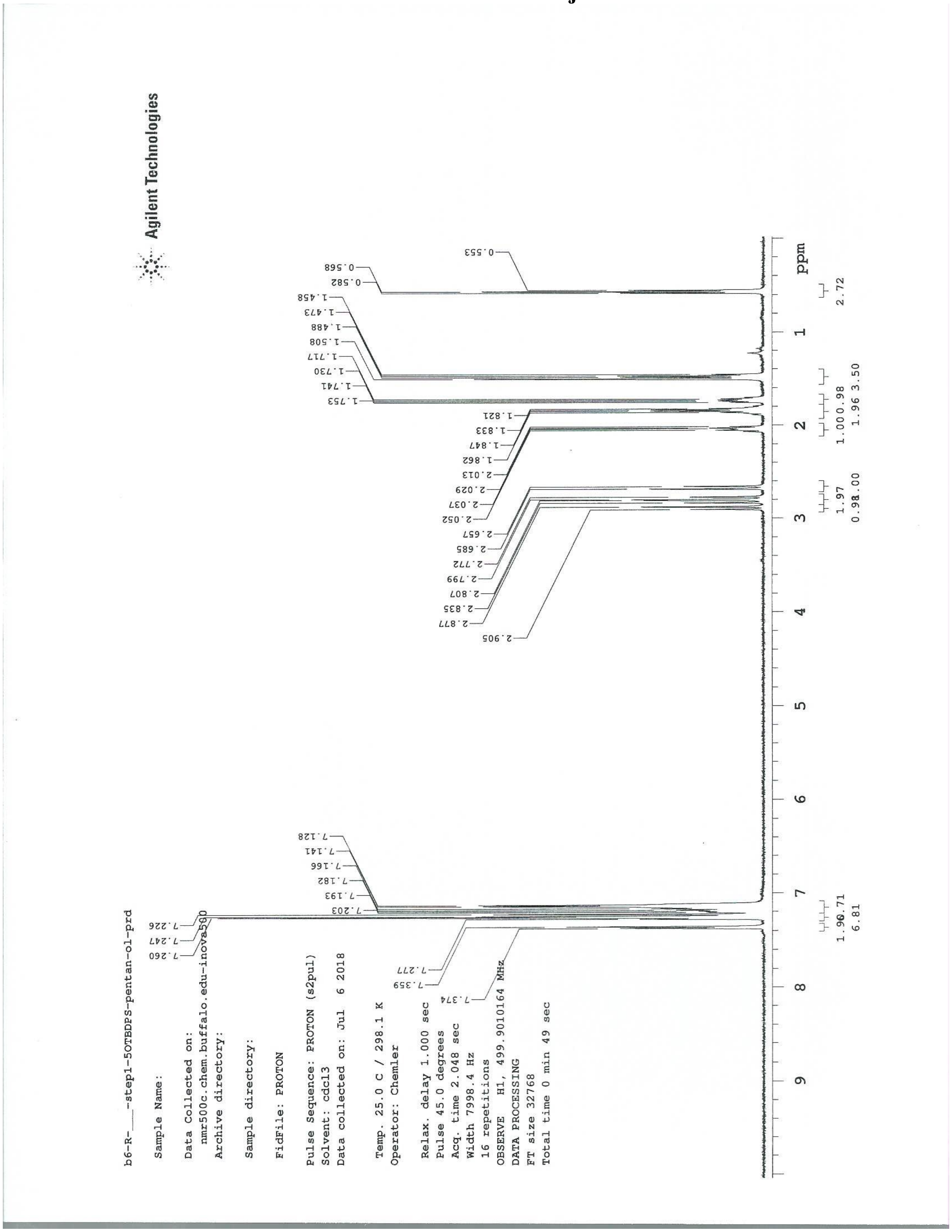
Agilent Technologies
b6-R-___-step1-5OTBDPS-pentan-ol-prd
Sample Name:
Data Collected on:
nmr500c.chem.buffalo.edu-inova500
Archive directory:
Sample directory:
FidFile: PROTON
Pulse Sequence: PROTON (s2pul)
Solvent: cdcl3
Data collected on: Jul 6 2018
Temp. 25.0 C / 298.1 K
Operator: Chemler
Relax. delay 1.000 sec
Pulse 45.0 degrees
Acq. time 2.048 sec
Width 7998.4 Hz
16 repetitions
OBSERVE H1, 499.9010164 MHz
DATA PROCESSING
FT size 32768
Total time 0 min 49 sec
ppm

b6-R-___-LDA-isobutyrate-prd-isomer-2-chiral
Selective band center: 3.51 (ppm); width: 44.1 (Hz)

Sample Name:

Data Collected on:
nmr500c.chem.buffalo.edu-inova500
Archive directory:

Sample directory:

FidFile: CARBON

Pulse Sequence: CARBON (s2pul)
Solvent: cdcl3
Data collected on: Aug 2 2018

Temp. 25.0 C / 298.1 K
Operator: Chemler

Relax. delay 2.000 sec
Pulse 45.0 degrees
Acq. time 1.043 sec
Width 31421.8 Hz
1128 repetitions
OBSERVE C13, 125.7002064 MHz
DECOUPLE H1, 499.9034960 MHz
Power 40 dB
continuously on
WALTZ-16 modulated
DATA PROCESSING
Line broadening 0.5 Hz
FT size 65536
Total time 847 hr, 42 min

Agilent Technologies

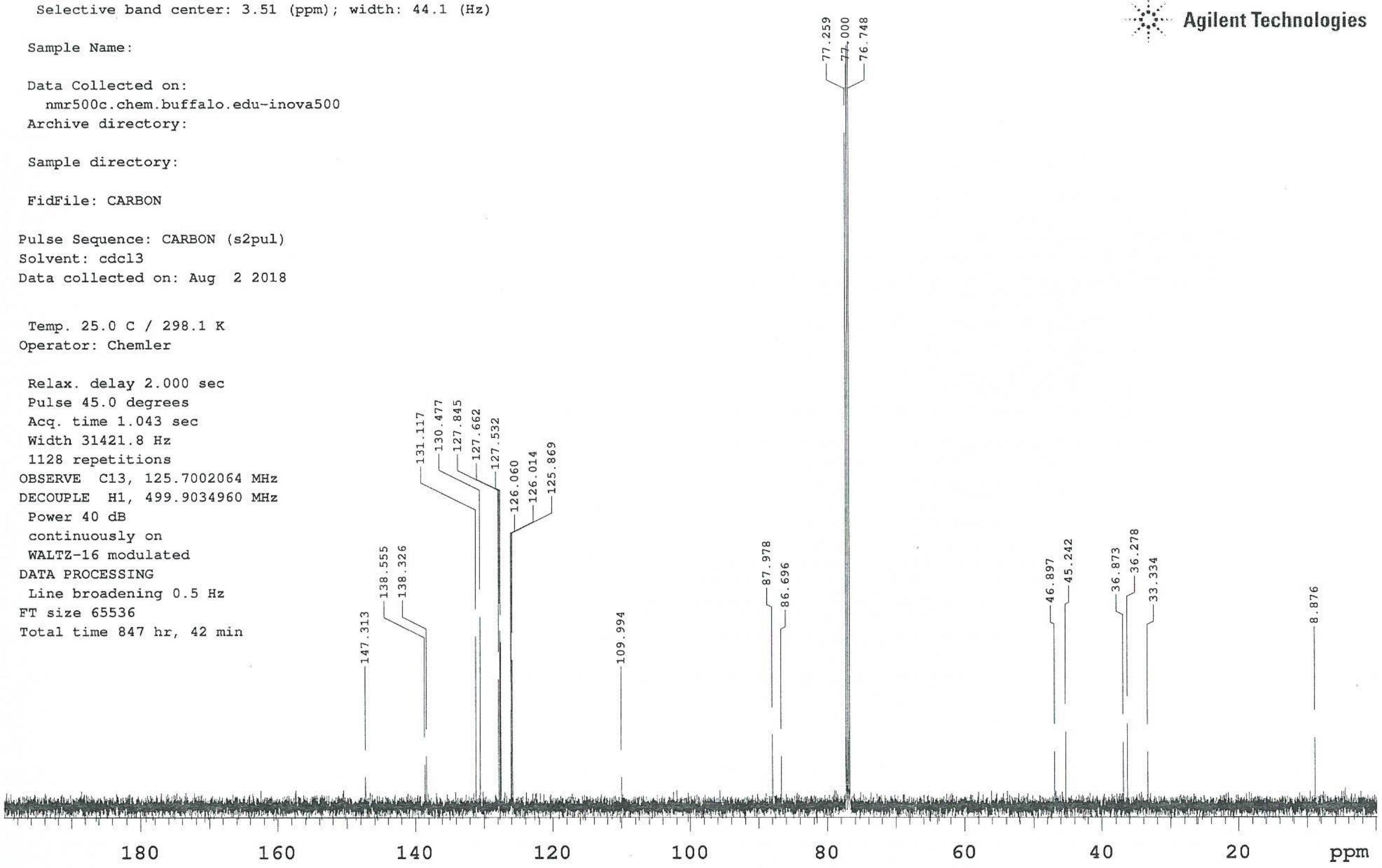

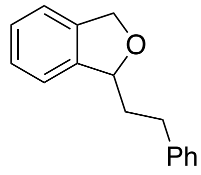

9a

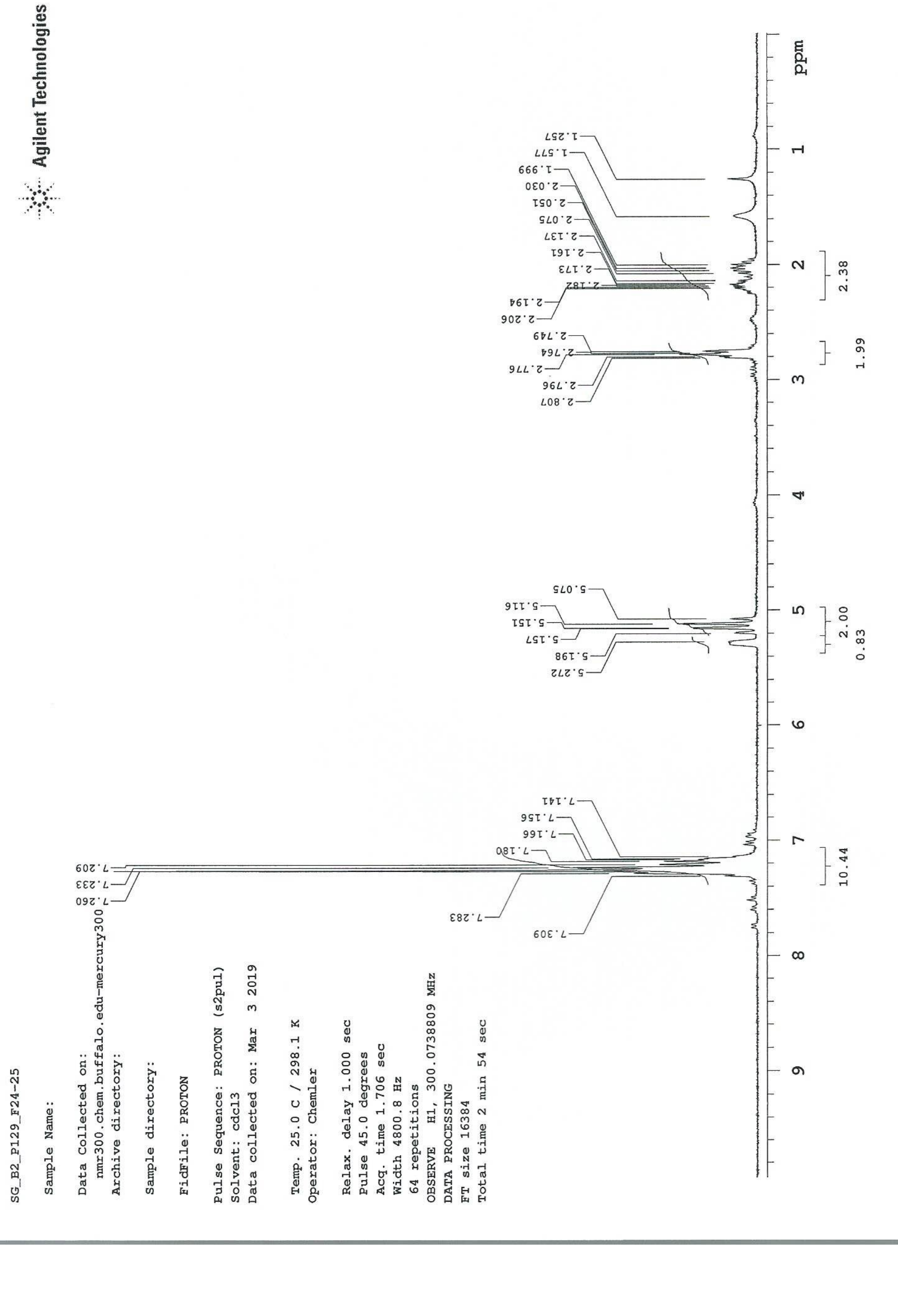

Agilent Technologies
SG_B2_P129_F24-25
Sample Name:
Data Collected on:
nmr300.chem.buffalo.edu-mercury300
Archive directory:
Sample directory:
FidFile: PROTON
Pulse Sequence: PROTON (s2pul)
Solvent: cdcl3
Data collected on: Mar 3 2019
Temp. 25.0 C / 298.1 K
Operator: Chemler
Relax. delay 1.000 sec
Pulse 45.0 degrees
Acq. time 1.706 sec
Width 4800.8 Hz
64 repetitions
OBSERVE H1, 300.0738809 MHz
DATA PROCESSING
FT size 16384
Total time 2 min 54 sec
7.309
7.283
7.260
7.233
7.209
7.180
7.166
7.156
7.141
5.272
5.198
5.157
5.151
5.116
5.075
2.807
2.796
2.776
2.764
2.749
2.206
2.194
2.182
2.173
2.161
2.137
2.075
2.051
2.030
1.999
1.577
1.257
10.44
0.83
2.00
1.99
2.38
9
8
7
6
5
4
3
2
1
ppm

SG_B2_P129_F24-25

Sample Name:

Data Collected on:
nmr300.chem.buffalo.edu-mercury300
Archive directory:

Sample directory:

FidFile: CARBON

Pulse Sequence: CARBON (s2pul)
Solvent: cdcl3
Data collected on: Mar 3 2019

Temp. 25.0 C / 298.1 K
Operator: Chemler

Relax. delay 1.000 sec
Pulse 45.0 degrees
Acq. time 0.868 sec
Width 18867.9 Hz
25968 repetitions
OBSERVE C13, 75.4536366 MHz
DECOUPLE H1, 300.0754430 MHz
Power 37 dB
continuously on
WALTZ-16 modulated
DATA PROCESSING
Line broadening 0.5 Hz
FT size 32768
Total time 5214 hr, 29 min

Agilent Technologies

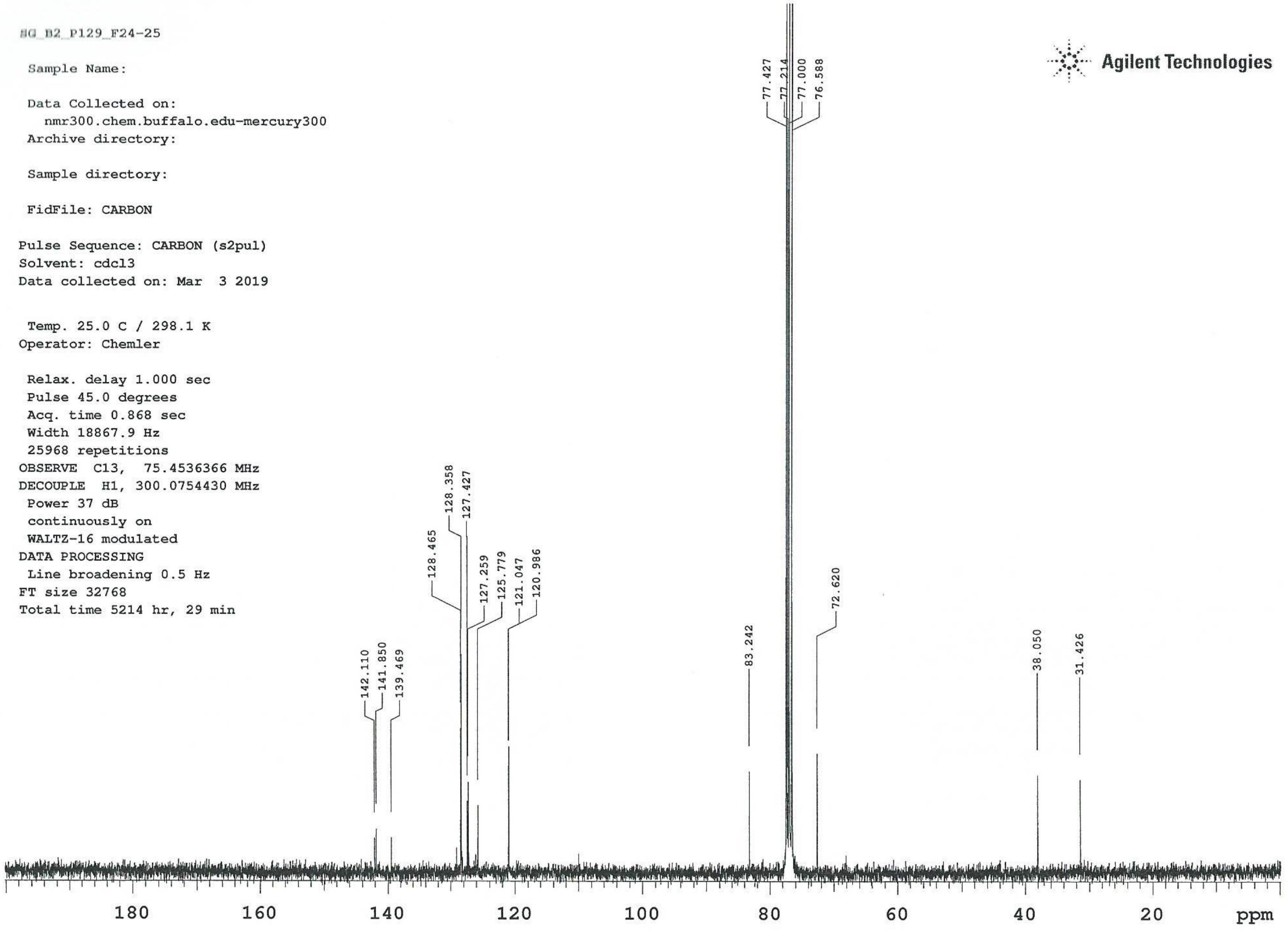

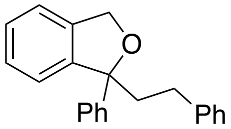

9b

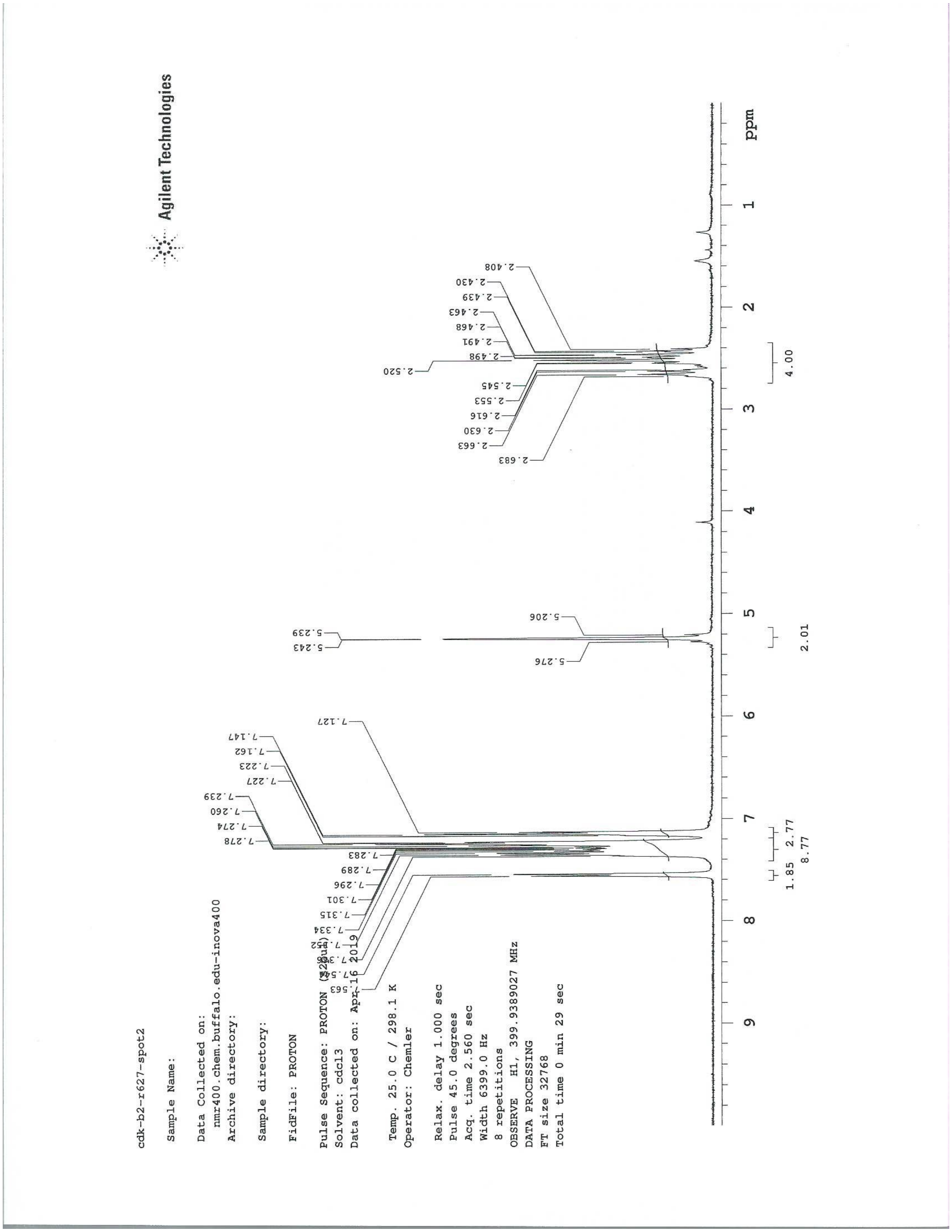

Agilent Technologies
cdk-b2-r627-spot2
Sample Name:
Data Collected on:
nmr400.chem.buffalo.edu-inova400
Archive directory:
Sample directory:
FidFile: PROTON
Pulse Sequence: PROTON (s2pul)
Solvent: cdcl3
Data collected on: Apr 16 2019
Temp. 25.0 C / 298.1 K
Operator: Chemler
Relax. delay 1.000 sec
Pulse 45.0 degrees
Acq. time 2.560 sec
Width 6399.0 Hz
8 repetitions
OBSERVE H1, 399.9389027 MHz
DATA PROCESSING
FT size 32768
Total time 0 min 29 sec
ppm
4.00
2.01
1.85 2.77
8.77

cdk-b2-r627-spot2

Sample Name:

Data Collected on:
nmr400.chem.buffalo.edu-inova400
Archive directory:

Sample directory:

FidFile: ERRBEON

Pulse Sequence: ERRBEON (s2pul)
Solvent: cdcl3
Data collected on: Apr 16 2019

Temp. 25.0 C / 298.1 K
Operator: Chemler

Relax. delay 1.000 sec
Pulse 45.0 degrees
Acq. time 2.568 sec
Width 25999104HHz
8888peepbtitsons
OBSERVE C13, 399.9589027 MHz
DECOUPROCESSING399.9409068 MHz
FPowere33268
Totatiimmeslymon 29 sec
WALTZ-16 modulated
DATA PROCESSING
Line broadening 0.5 Hz
FT size 65536
Total time 64228 hr, 28 min

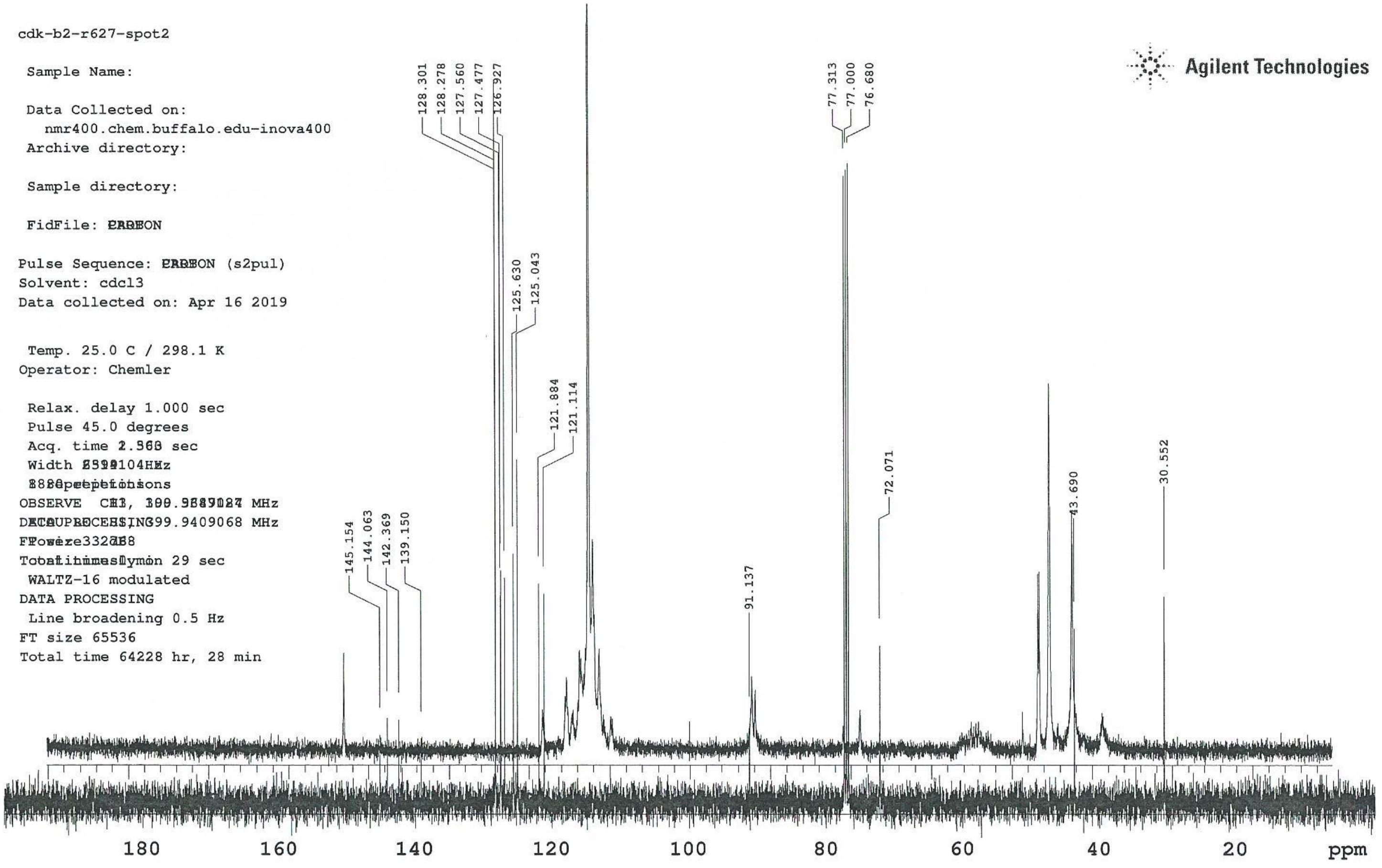

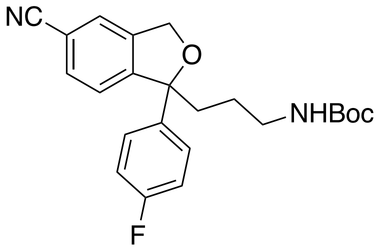
NC
O
NHBoc
F

9c

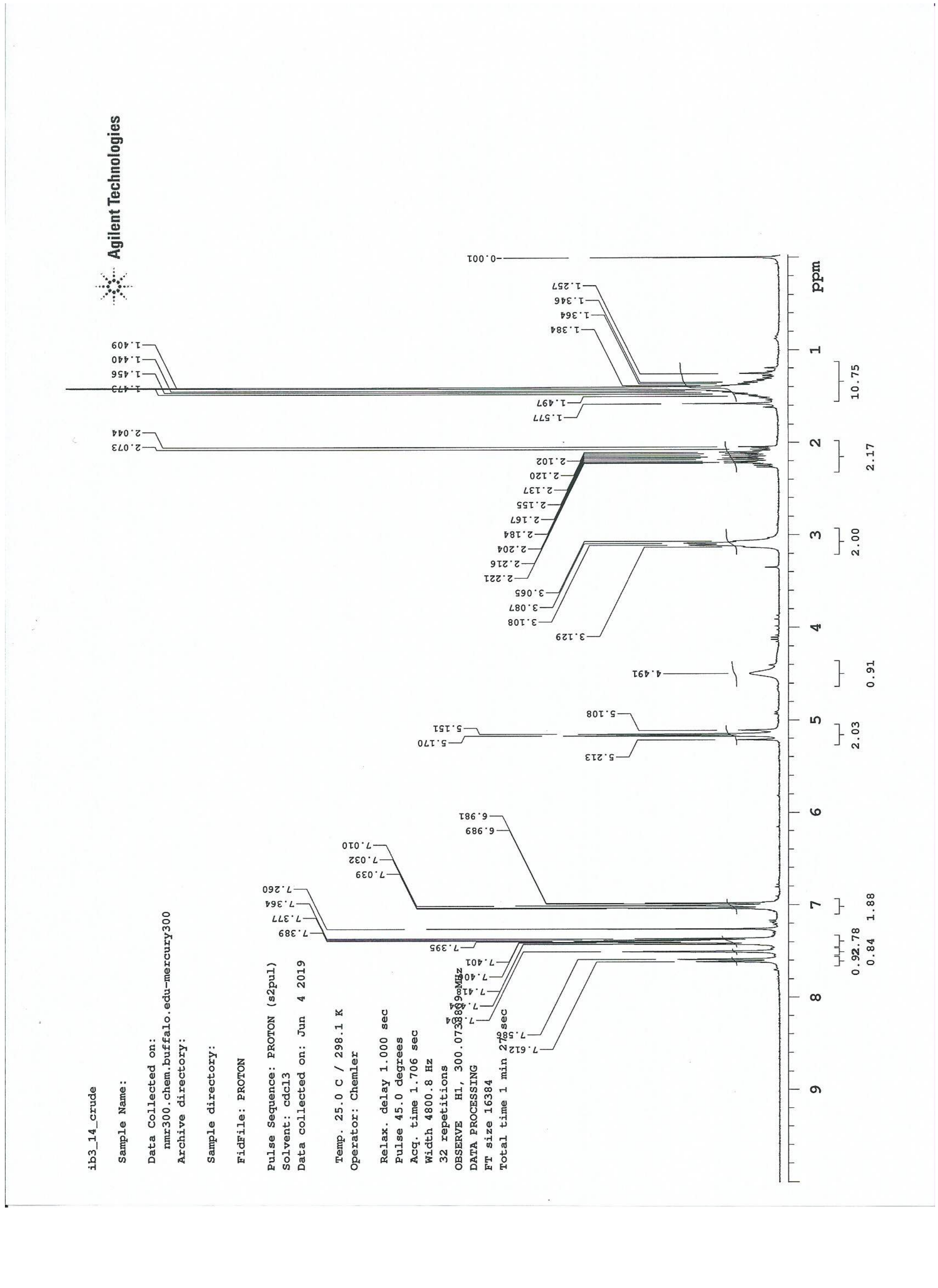
Agilent Technologies
ib3_14_crude
Sample Name:
Data Collected on:
nmr300.chem.buffalo.edu-mercury300
Archive directory:
Sample directory:
FidFile: PROTON
Pulse Sequence: PROTON (s2pul)
Solvent: cdcl3
Data collected on: Jun 4 2019
Temp. 25.0 C / 298.1 K
Operator: Chemler
Relax. delay 1.000 sec
Pulse 45.0 degrees
Acq. time 1.706 sec
Width 4800.8 Hz
32 repetitions
OBSERVE H1, 300.0738819 MHz
DATA PROCESSING
FT size 16384
Total time 1 min 28 sec
ppm

RC19-B1-F32-F35

Sample Name:

Data Collected on:
nmr300.chem.buffalo.edu-mercury300
Archive directory:

Sample directory:

FidFile: CARBON

Pulse Sequence: CARBON (s2pul)
Solvent: cdcl3
Data collected on: Jun 6 2019

Temp. 25.0 C / 298.1 K
Operator: Chemler

Relax. delay 2.000 sec
Pulse 45.0 degrees
Acq. time 0.868 sec
Width 18867.9 Hz
13944 repetitions
OBSERVE C13, 75.4536377 MHz
DECOUPLE H1, 300.0754430 MHz
Power 37 dB
continuously on
WALTZ-16 modulated
DATA PROCESSING
Line broadening 0.5 Hz
FT size 32768
Total time 7992 hr, 16 min

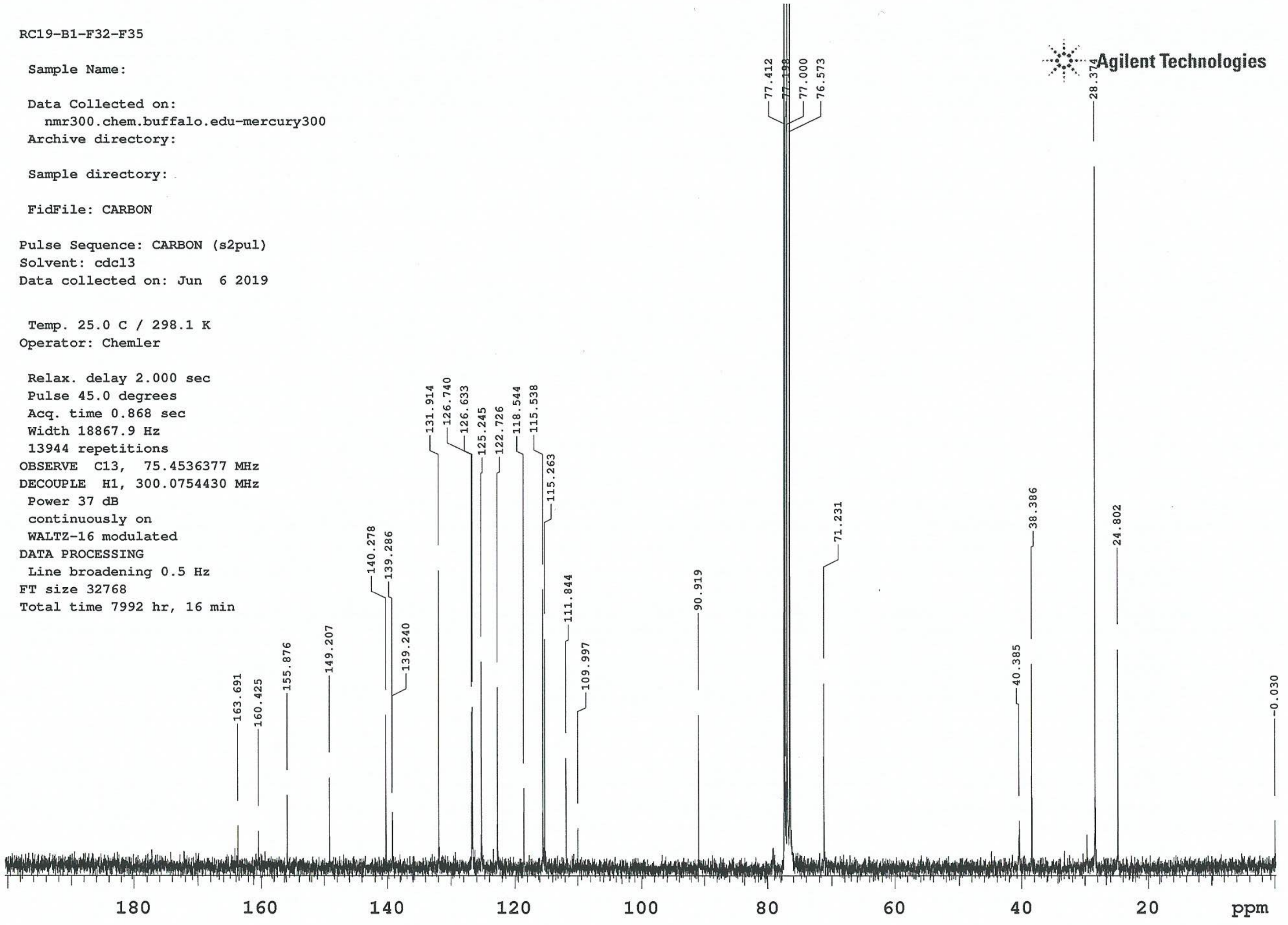

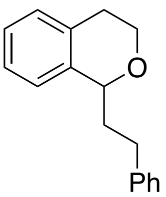

10a

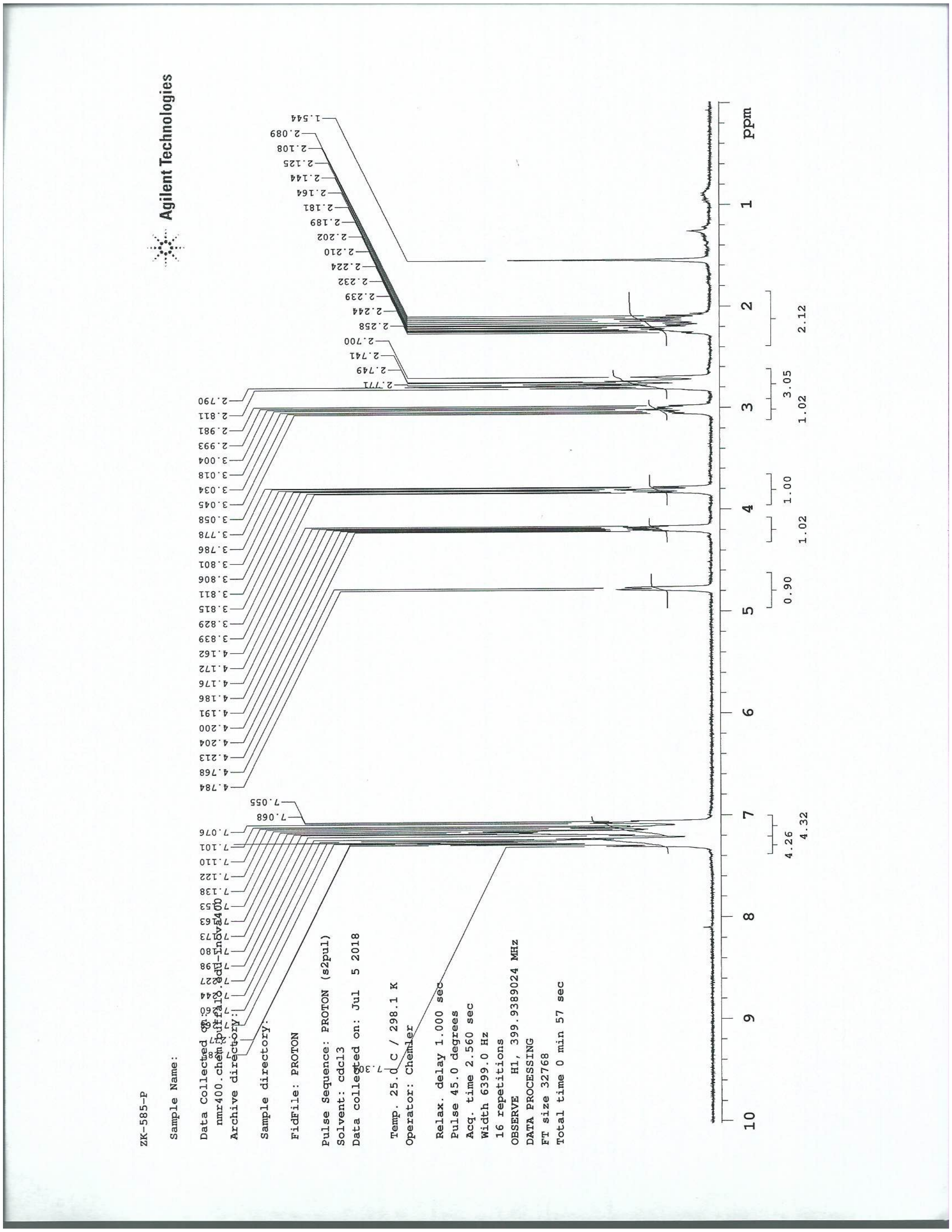
Agilent Technologies
ZK-585-P
Sample Name:
Data Collected on:
nmr400.chem.buffalo.edu-inova400
Archive directory:
Sample directory:
FidFile: PROTON
Pulse Sequence: PROTON (s2pul)
Solvent: cdcl3
Data collected on: Jul 5 2018
Temp. 25.0 C / 298.1 K
Operator: Chemler
Relax. delay 1.000 sec
Pulse 45.0 degrees
Acq. time 2.560 sec
Width 6399.0 Hz
16 repetitions
OBSERVE H1, 399.9389024 MHz
DATA PROCESSING
FT size 32768
Total time 0 min 57 sec
4.26
4.32
0.90
1.02
1.00
1.02
3.05
2.12
ppm

b6-R-____-5OTBDPS-step8-spot-below-aldehyde-from-PhIOAc2-step

Sample Name:

Data Collected on:
nmr400.chem.buffalo.edu-inova400
Archive directory:

Sample directory:

FidFile: CARBON

Pulse Sequence: CARBON (s2pul)
Solvent: cdcl3
Data collected on: Jul 9 2018

Temp. 25.0 C / 298.1 K
Operator: Chemler

Relax. delay 2.000 sec
Pulse 45.0 degrees
Acq. time 1.303 sec
Width 25141.4 Hz
2408 repetitions
OBSERVE C13, 100.5647177 MHz
DECOUPLE H1, 399.9409068 MHz
Power 33 dB
continuously on
WALTZ-16 modulated
DATA PROCESSING
Line broadening 0.5 Hz
FT size 65536
Total time 92 hr

Agilent Technologies

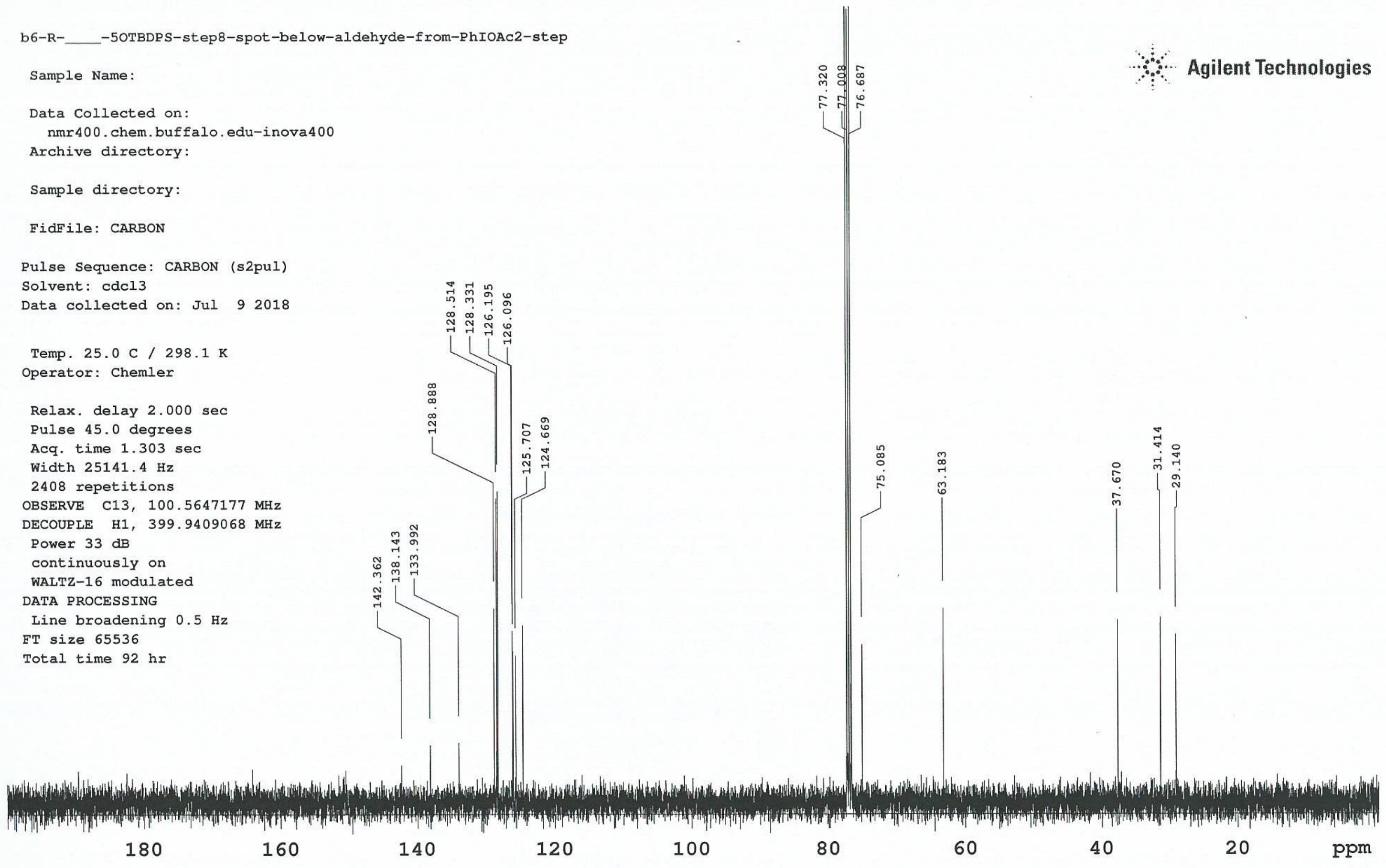

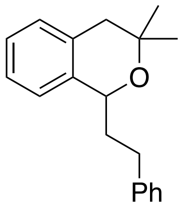

10b

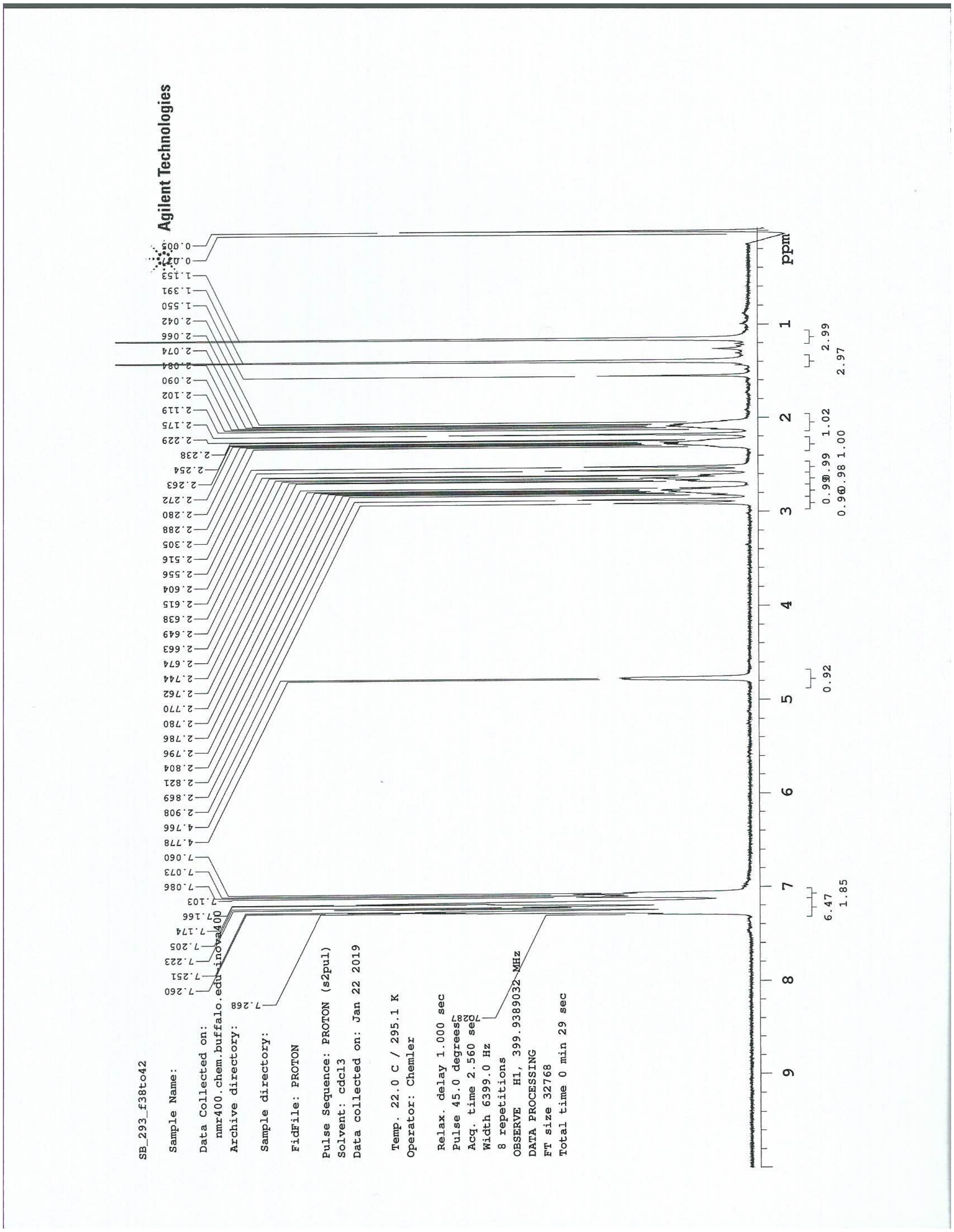
SB_293_f38to42
Sample Name:
Data Collected on:
nmr400.chem.buffalo.edu-inova400
Archive directory:
Sample directory:
FidFile: PROTON
Pulse Sequence: PROTON (s2pul)
Solvent: cdcl3
Data collected on: Jan 22 2019
Temp. 22.0 C / 295.1 K
Operator: Chemler
Relax. delay 1.000 sec
Pulse 45.0 degrees
Acq. time 2.560 sec
Width 6399.0 Hz
8 repetitions
OBSERVE H1, 399.9389032 MHz
DATA PROCESSING
FT size 32768
Total time 0 min 29 sec
Agilent Technologies
ppm

SB_293_f38to42

Sample Name:

Data Collected on:
nmr400.chem.buffalo.edu-inova400
Archive directory:

Sample directory:

FidFile: CARBON

Pulse Sequence: CARBON (s2pul)
Solvent: cdcl3
Data collected on: Jan 22 2019

Temp. 22.0 C / 295.1 K
Operator: Chemler

Relax. delay 2.000 sec
Pulse 45.0 degrees
Acq. time 1.303 sec
Width 25141.4 Hz
920 repetitions
OBSERVE C13, 100.5647177 MHz
DECOUPLE H1, 399.9409068 MHz
Power 33 dB
continuously on
WALTZ-16 modulated
DATA PROCESSING
Line broadening 0.5 Hz
FT size 65536
Total time 920 hr, 3 min

Agilent Technologies

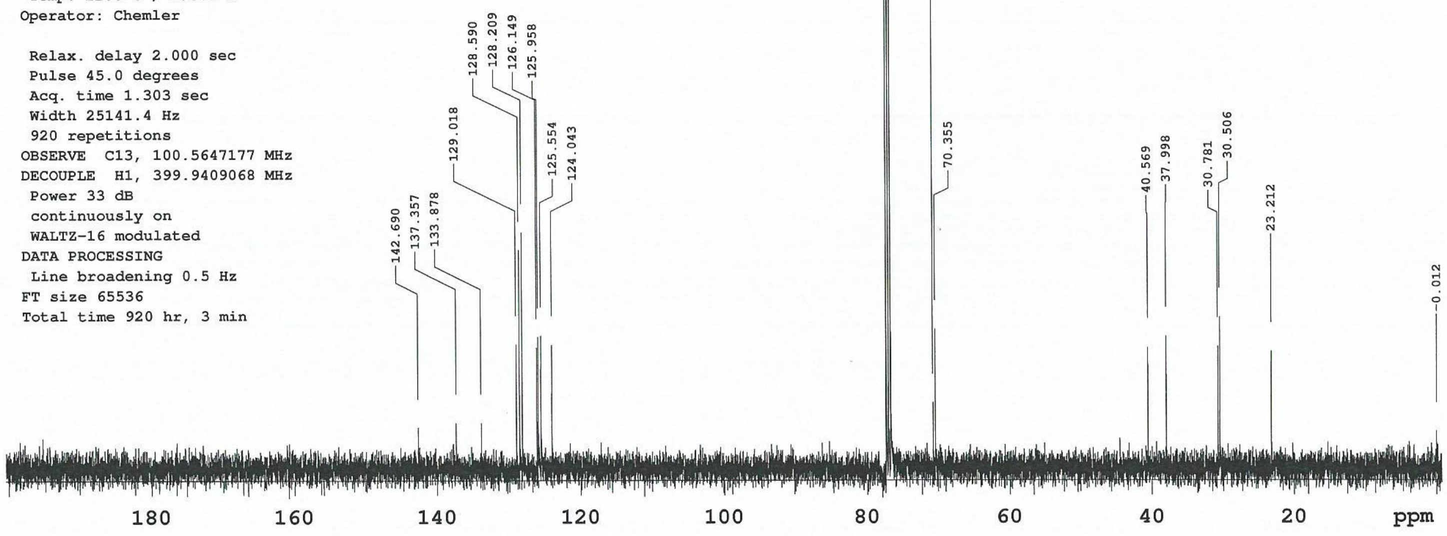

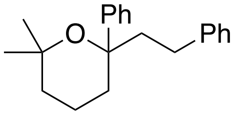
Ph
O
Ph

10c

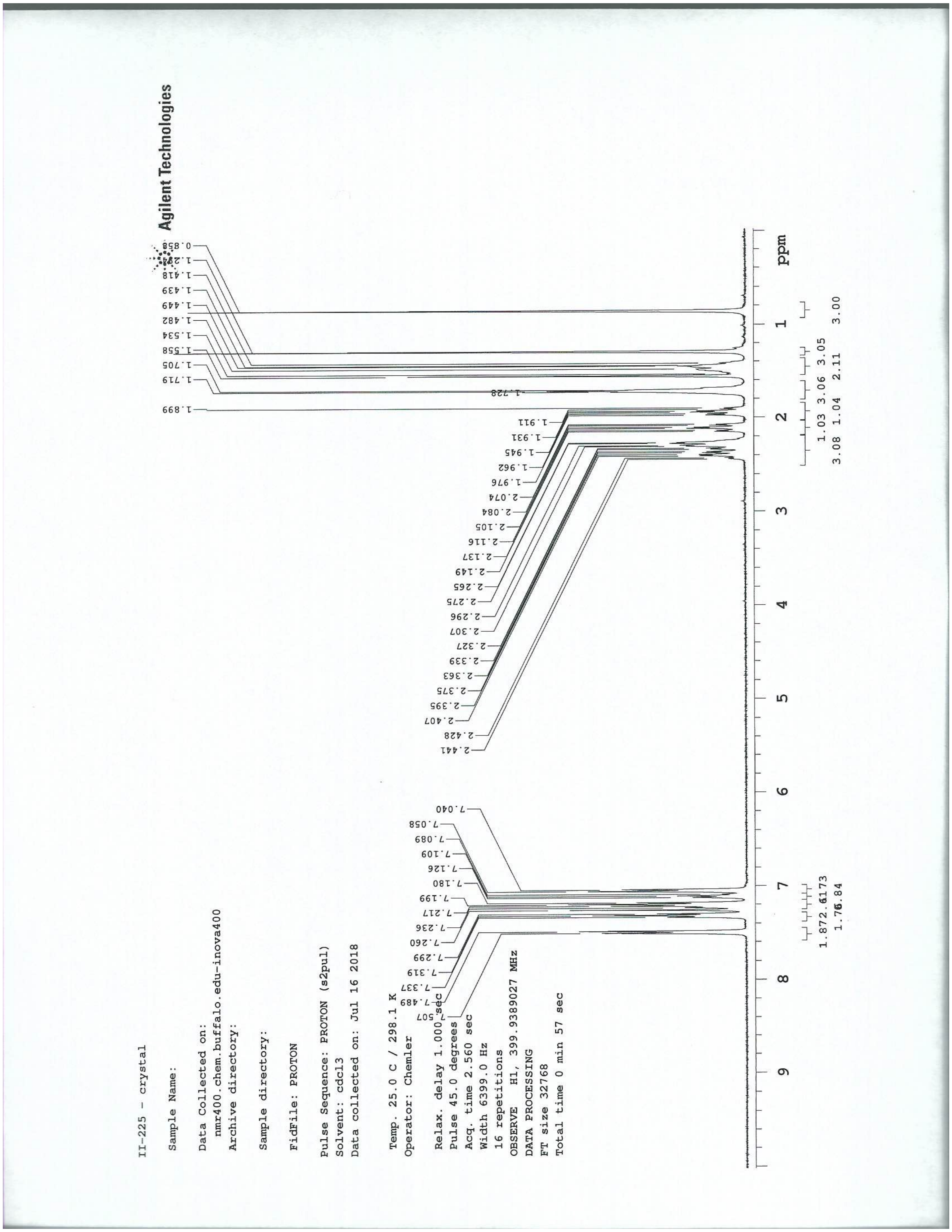
Agilent Technologies
II-225 - crystal
Sample Name:
Data Collected on:
nmr400.chem.buffalo.edu-inova400
Archive directory:
Sample directory:
FidFile: PROTON
Pulse Sequence: PROTON (s2pul)
Solvent: cdcl3
Data collected on: Jul 16 2018
Temp. 25.0 C / 298.1 K
Operator: Chemler
Relax. delay 1.000 sec
Pulse 45.0 degrees
Acq. time 2.560 sec
Width 6399.0 Hz
16 repetitions
OBSERVE H1, 399.9389027 MHz
DATA PROCESSING
FT size 32768
Total time 0 min 57 sec
ppm

II-225 - crystal

Sample Name:

Data Collected on:
nmr400.chem.buffalo.edu-inova400
Archive directory:

Sample directory:

FidFile: CARBON

Pulse Sequence: CARBON (s2pul)
Solvent: cdcl3
Data collected on: Jul 16 2018

Temp. 25.0 C / 298.1 K
Operator: Chemler

Relax. delay 2.000 sec
Pulse 45.0 degrees
Acq. time 1.303 sec
Width 25141.4 Hz
2072 repetitions
OBSERVE C13, 100.5647177 MHz
DECOUPLE H1, 399.9409068 MHz
Power 33 dB
continuously on
WALTZ-16 modulated
DATA PROCESSING
Line broadening 0.5 Hz
FT size 65536
Total time 920 hr, 3 min

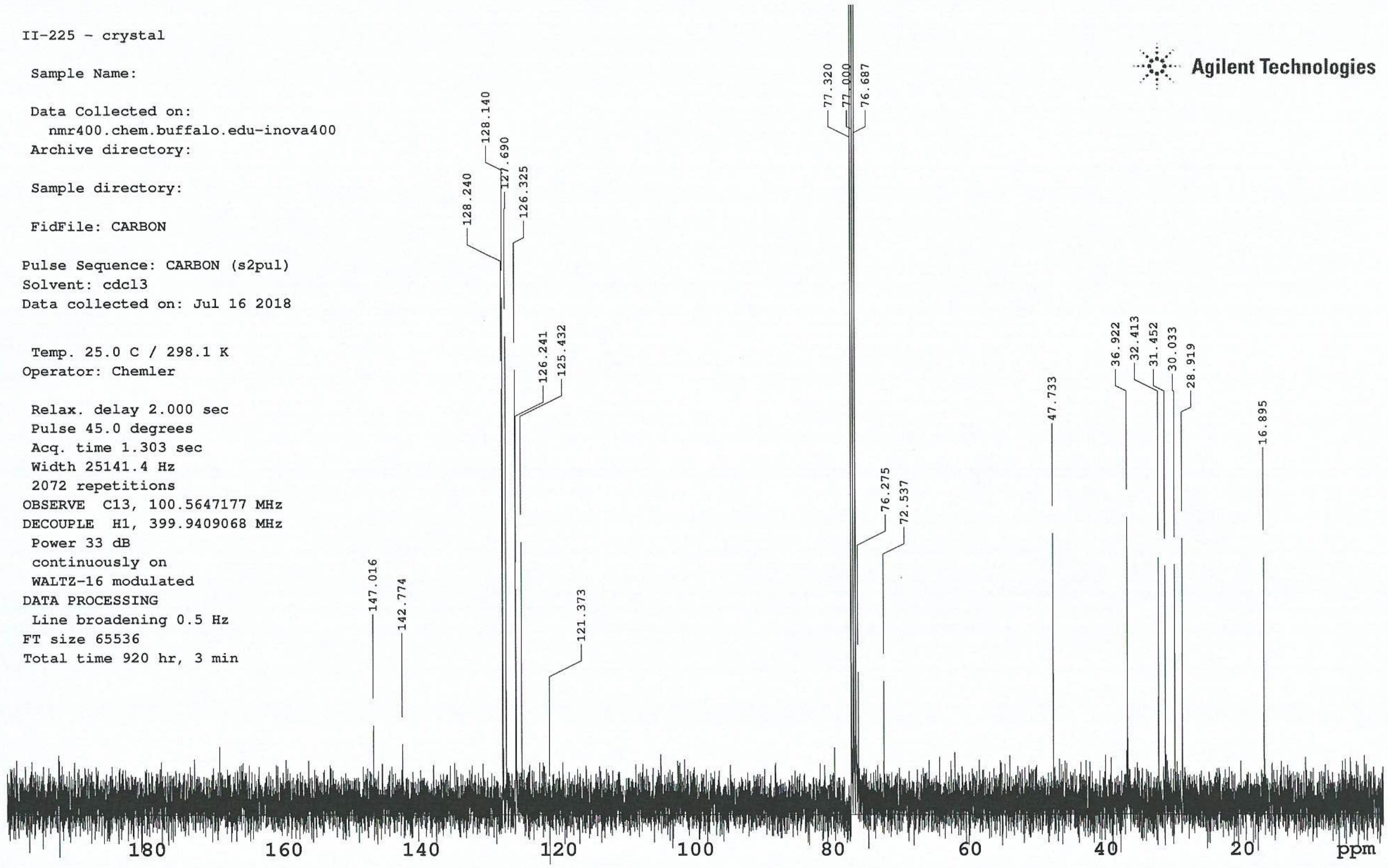

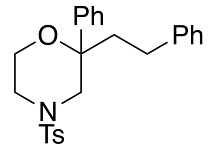

O
Ph
Ph
N
Ts

10d

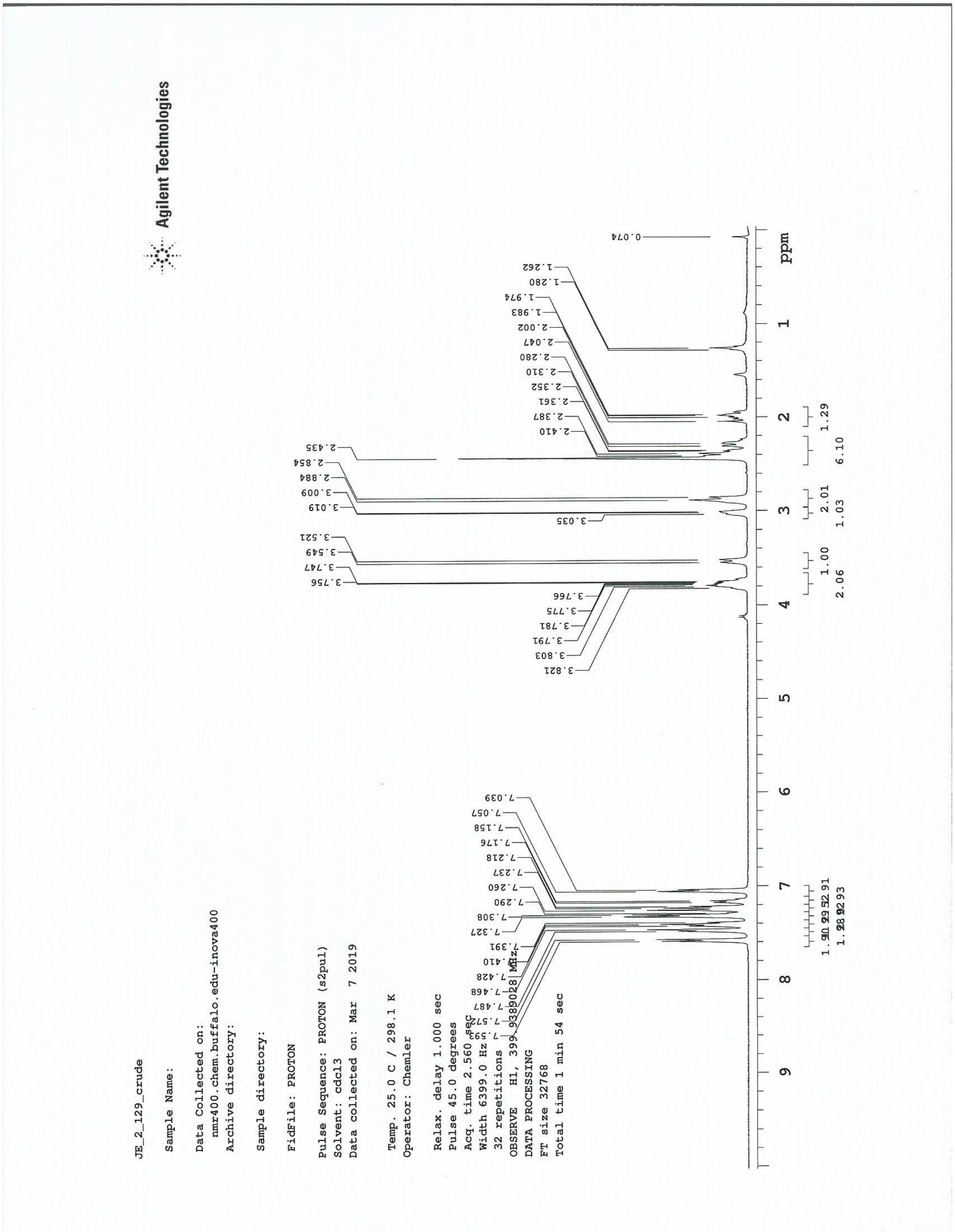

Agilent Technologies
JE_2_129_crude
Sample Name:
Data Collected on:
nmr400.chem.buffalo.edu-inova400
Archive directory:
Sample directory:
FidFile: PROTON
Pulse Sequence: PROTON (s2pul)
Solvent: cdcl3
Data collected on: Mar 7 2019
Temp. 25.0 C / 298.1 K
Operator: Chemler
Relax. delay 1.000 sec
Pulse 45.0 degrees
Acq. time 2.560 sec
Width 6399.0 Hz
32 repetitions
OBSERVE H1, 399.9389028 MHz
DATA PROCESSING
FT size 32768
Total time 1 min 54 sec
0.074
1.262
1.280
1.974
1.983
2.002
2.047
2.280
2.310
2.352
2.361
2.387
2.410
2.435
2.854
2.884
3.009
3.019
3.035
3.521
3.549
3.747
3.756
3.766
3.775
3.781
3.791
3.803
3.821
7.039
7.057
7.158
7.176
7.218
7.237
7.260
7.290
7.308
7.327
7.391
7.410
7.428
7.468
7.487
7.571
7.593
1.29
6.10
2.01
1.03
1.00
2.06
ppm
1
2
3
4
5
6
7
8
9

ASB-B___-allylalc-biphenyl-ketal-colpure

Sample Name:

Data Collected on:
nmr400.chem.buffalo.edu-inova400
Archive directory:

Sample directory:

FidFile: CARBON

Pulse Sequence: CARBON (s2pul)
Solvent: cdcl3
Data collected on: Mar 9 2019

Temp. 25.0 C / 298.1 K
Operator: Chemler

Relax. delay 2.000 sec
Pulse 45.0 degrees
Acq. time 1.303 sec
Width 25141.4 Hz
11824 repetitions
OBSERVE C13, 100.5647177 MHz
DECOUPLE H1, 399.9409068 MHz
Power 33 dB
continuously on
WALTZ-16 modulated
DATA PROCESSING
Line broadening 0.5 Hz
FT size 65536
Total time 92006 hr, 14 min

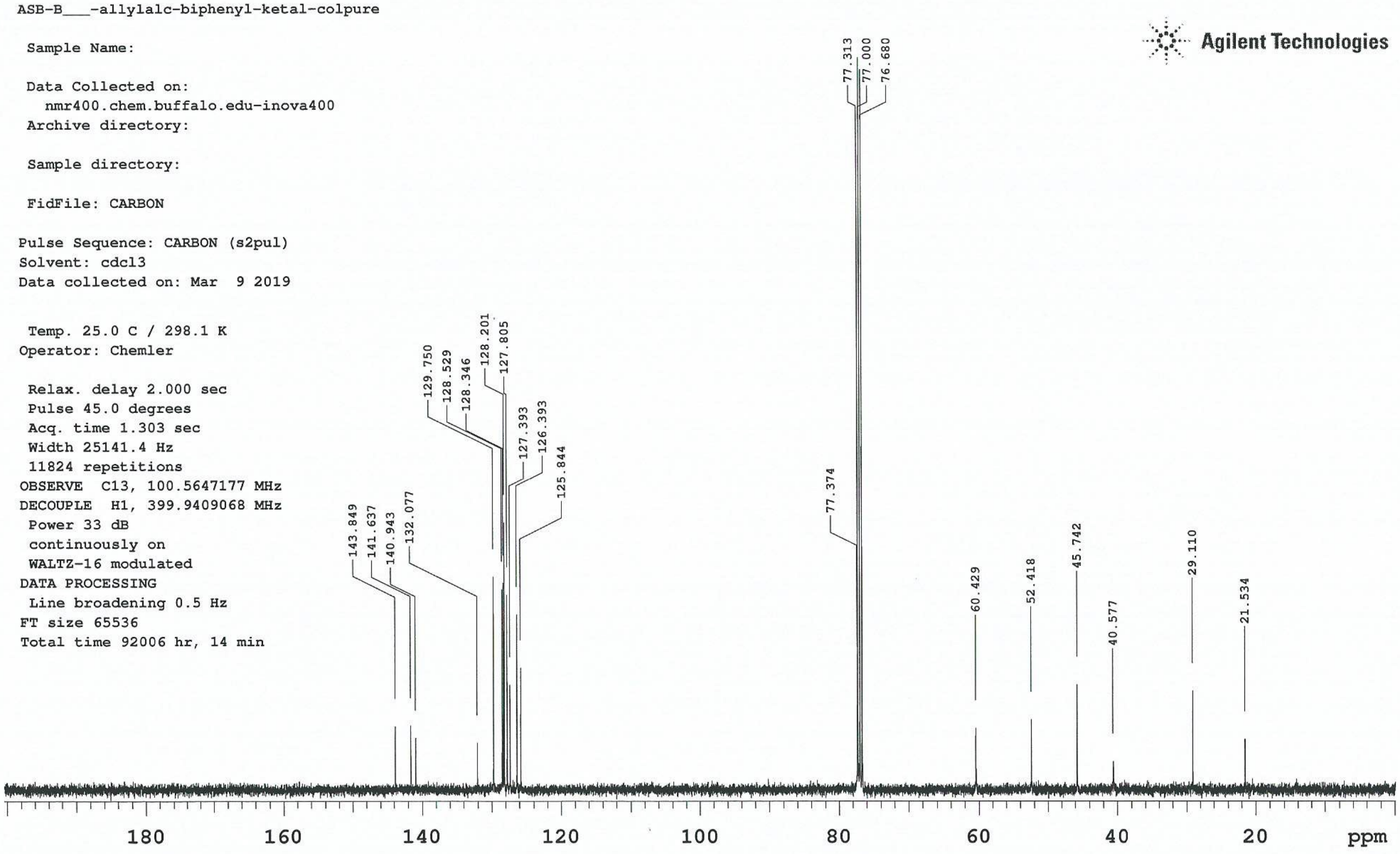

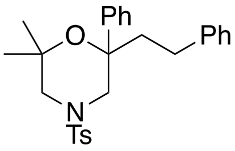
Ph
O
Ph
N
Ts

10e

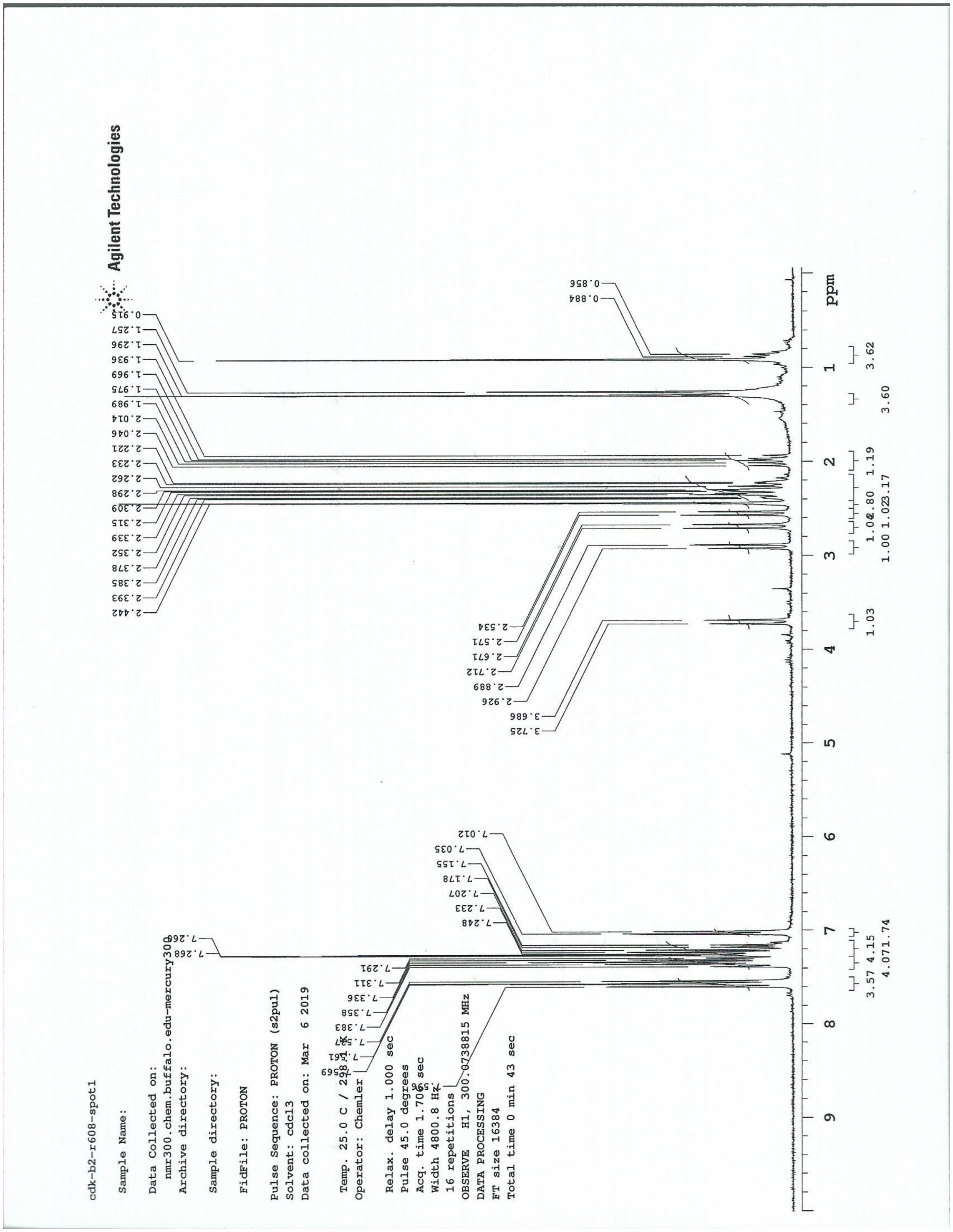
Agilent Technologies
cdk-b2-r608-spot1
Sample Name:
Data Collected on:
nmr300.chem.buffalo.edu-mercury300
Archive directory:
Sample directory:
FidFile: PROTON
Pulse Sequence: PROTON (s2pul)
Solvent: cdcl3
Data collected on: Mar 6 2019
Temp. 25.0 C / 298.1 K
Operator: Chemler
Relax. delay 1.000 sec
Pulse 45.0 degrees
Acq. time 1.706 sec
Width 4800.8 Hz
16 repetitions
OBSERVE H1, 300.0738815 MHz
DATA PROCESSING
FT size 16384
Total time 0 min 43 sec
7.569
7.561
7.537
7.383
7.358
7.336
7.311
7.291
7.268
7.266
7.248
7.233
7.207
7.178
7.155
7.035
7.012
3.725
3.686
2.926
2.889
2.712
2.671
2.571
2.534
2.442
2.393
2.385
2.378
2.352
2.339
2.315
2.309
2.268
2.262
2.233
2.221
2.046
2.014
1.989
1.975
1.969
1.936
1.296
1.257
0.915
0.884
0.856
3.57
4.15
4.07
1.74
1.03
1.00
1.02
1.02
0.80
3.17
1.19
3.60
3.62
ppm
9
8
7
6
5
4
3
2
1

SG_B2_P128_Crude

Sample Name:

Data Collected on:
nmr300.chem.buffalo.edu-mercury300
Archive directory:

Sample directory:

FidFile: CARBON

Pulse Sequence: CARBON (s2pul)
Solvent: cdcl3
Data collected on: Feb 28 2019

Temp. 25.0 C / 298.1 K
Operator: Chemler

Relax. delay 1.000 sec
Pulse 45.0 degrees
Acq. time 0.868 sec
Width 18867.9 Hz
904 repetitions
OBSERVE C13, 75.4536377 MHz
DECOUPLE H1, 300.0754430 MHz
Power 37 dB
continuously on
WALTZ-16 modulated
DATA PROCESSING
Line broadening 0.5 Hz
FT size 32768
Total time 5214 hr, 29 min

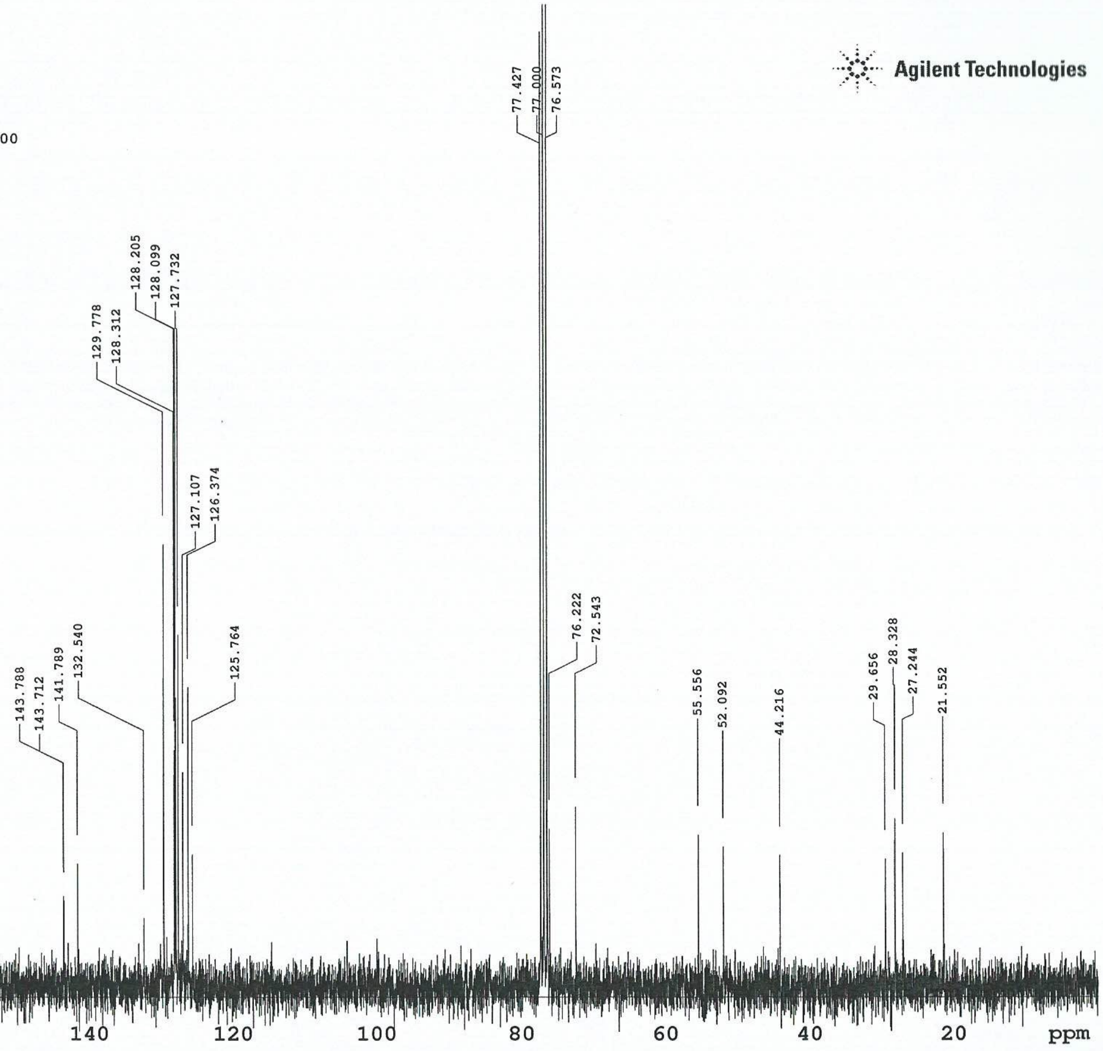

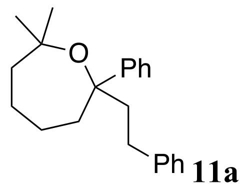
O
Ph
Ph

11a

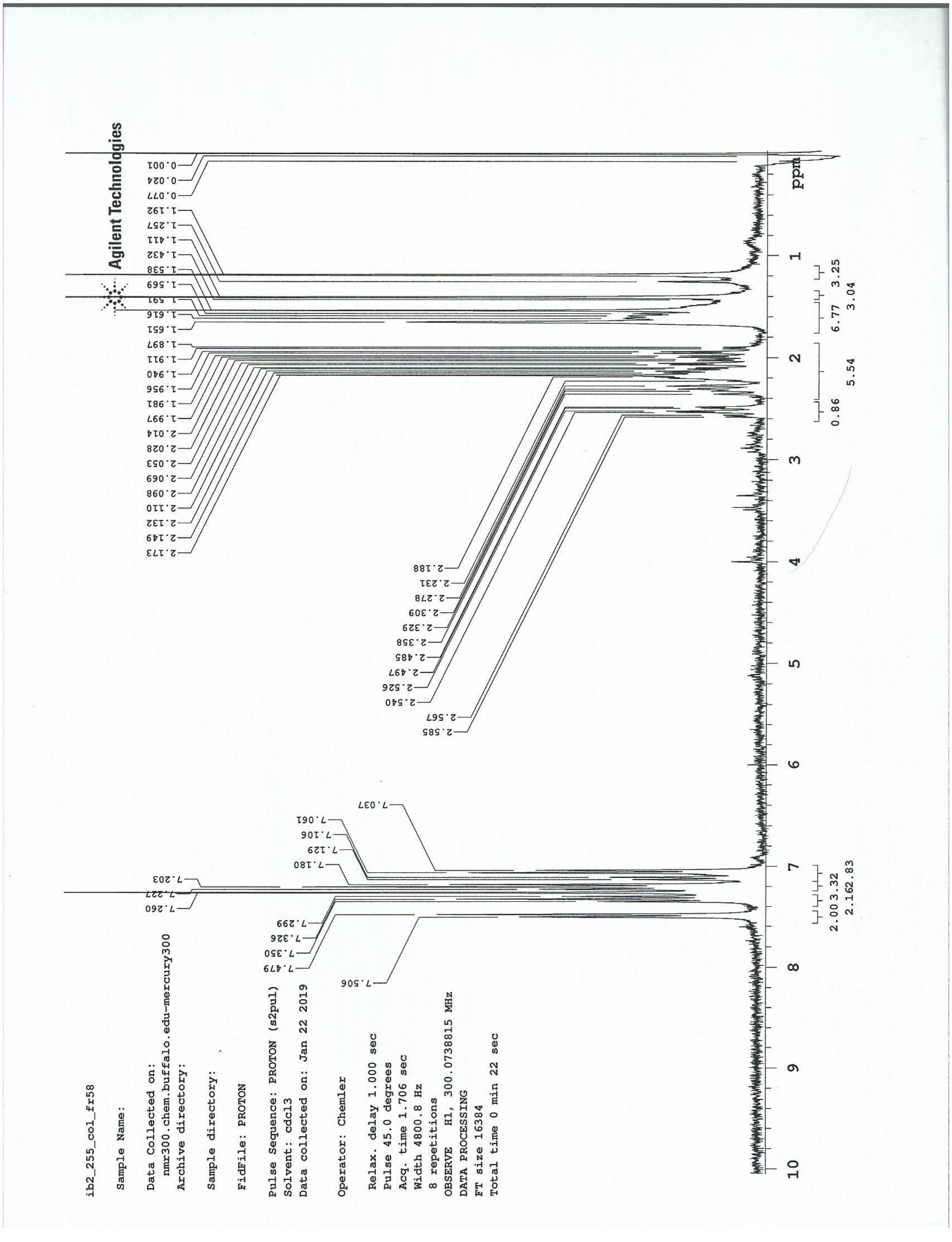
ib2_255_col_fr58
Sample Name:
Data Collected on:
nmr300.chem.buffalo.edu-mercury300
Archive directory:
Sample directory:
FidFile: PROTON
Pulse Sequence: PROTON (s2pul)
Solvent: cdcl3
Data collected on: Jan 22 2019
Operator: Chemler
Relax. delay 1.000 sec
Pulse 45.0 degrees
Acq. time 1.706 sec
Width 4800.8 Hz
8 repetitions
OBSERVE H1, 300.0738815 MHz
DATA PROCESSING
FT size 16384
Total time 0 min 22 sec
Agilent Technologies
7.506
7.479
7.350
7.326
7.299
7.260
7.227
7.203
7.180
7.129
7.106
7.061
7.037
2.585
2.567
2.540
2.526
2.497
2.485
2.358
2.329
2.309
2.278
2.231
2.188
2.173
2.149
2.132
2.110
2.098
2.069
2.053
2.028
2.014
1.997
1.981
1.956
1.940
1.911
1.897
1.651
1.616
1.591
1.569
1.538
1.432
1.411
1.257
1.192
0.077
0.024
0.001
10
9
8
7
6
5
4
3
2
1
ppm
2.00
3.32
2.16
2.83
0.86
5.54
6.77
3.04
3.25

ib2_255_col_fr58

Sample Name:

Data Collected on:
nmr300.chem.buffalo.edu-mercury300
Archive directory:

Sample directory:

FidFile: CARBON

Pulse Sequence: CARBON (s2pul)
Solvent: cdcl3
Data collected on: Jan 22 2019

Operator: Chemler

Relax. delay 2.000 sec
Pulse 45.0 degrees
Acq. time 0.868 sec
Width 18867.9 Hz
15168 repetitions
OBSERVE C13, 75.4536366 MHz
DECOUPLE H1, 300.0754430 MHz
Power 37 dB
continuously on
WALTZ-16 modulated
DATA PROCESSING
Line broadening 0.5 Hz
FT size 32768
Total time 79922 hr, 44 min

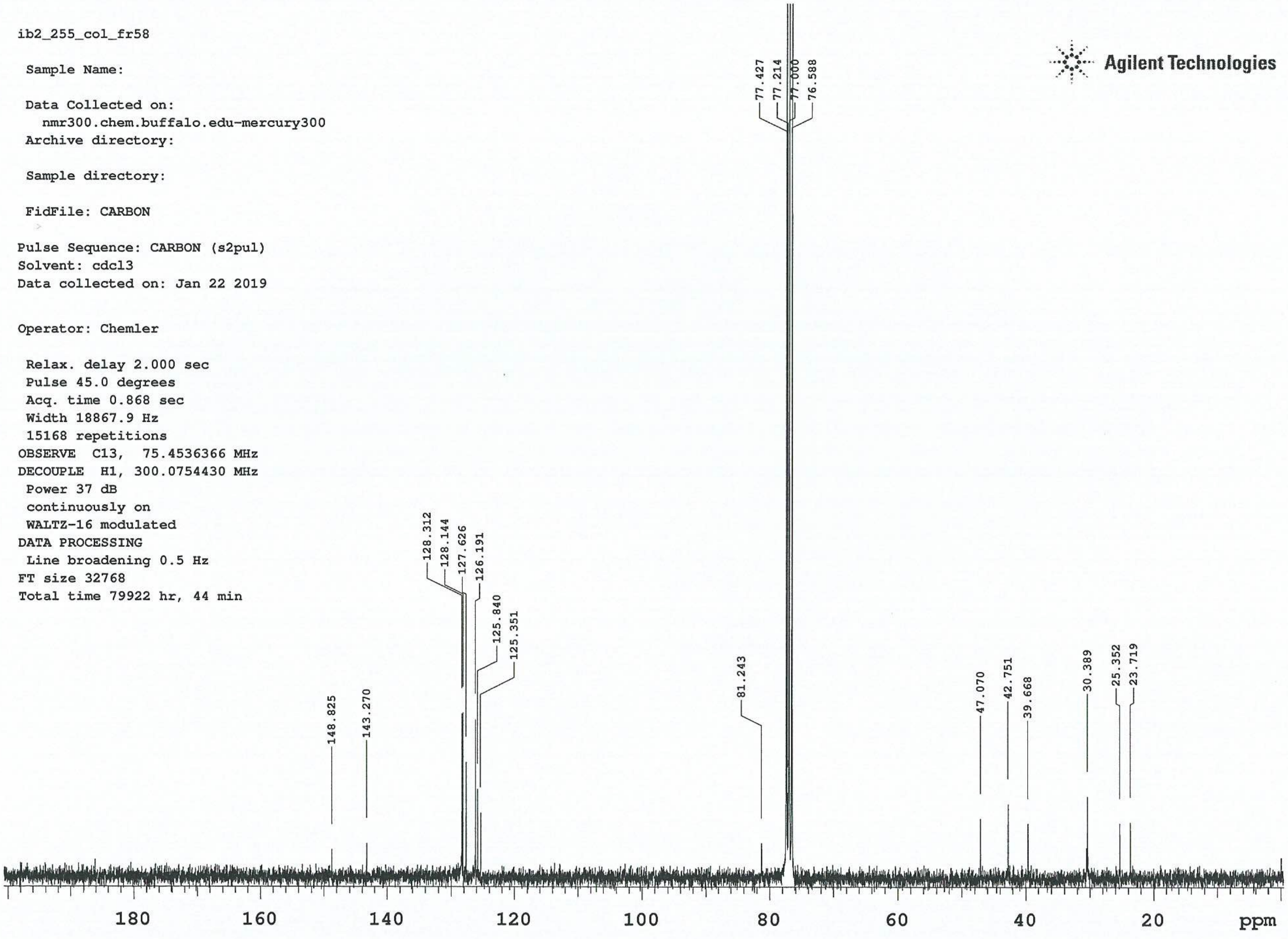

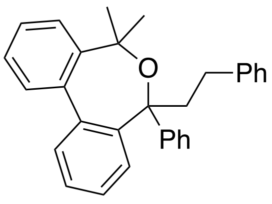

11b

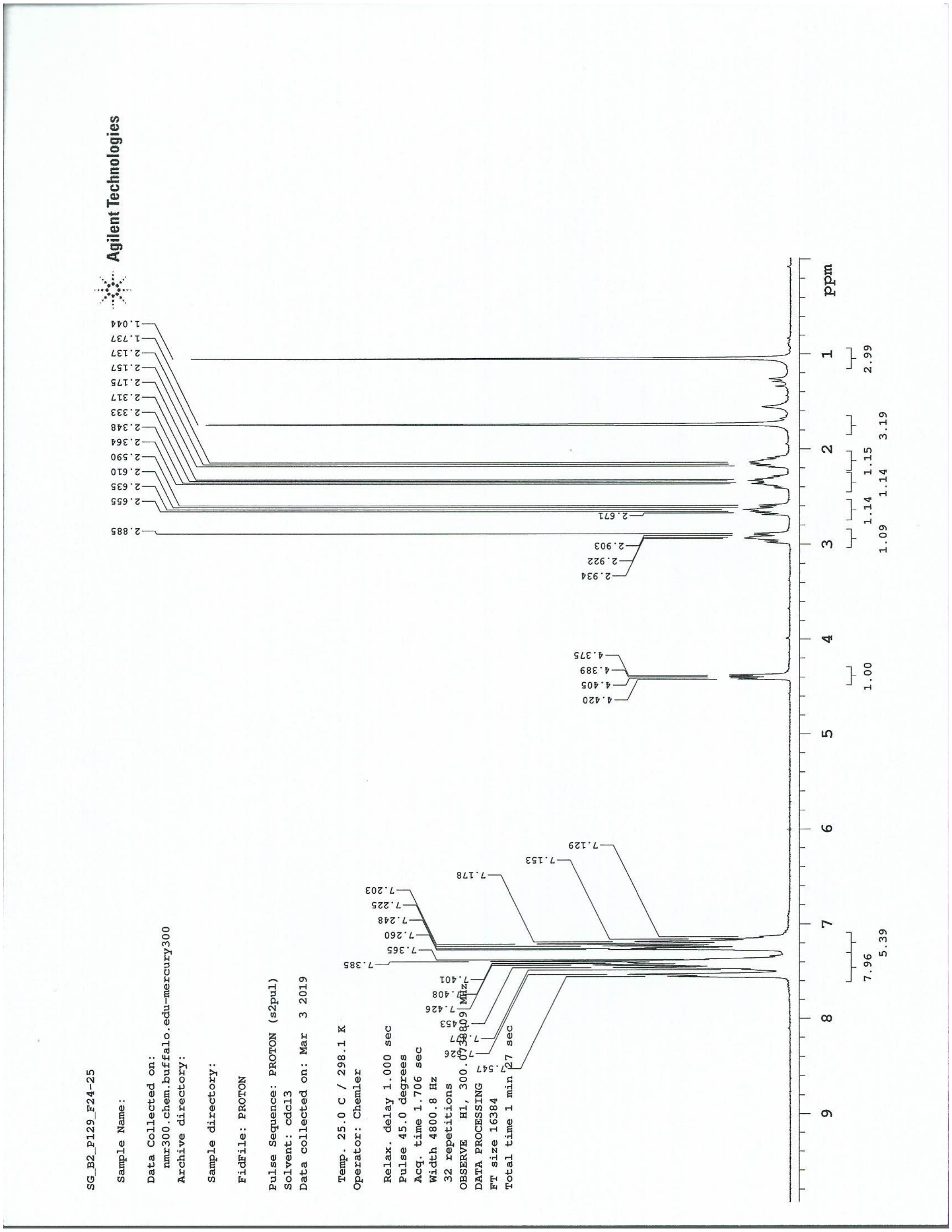
SG_B2_P129_F24-25
Sample Name:
Data Collected on:
nmr300.chem.buffalo.edu-mercury300
Archive directory:
Sample directory:
FidFile: PROTON
Pulse Sequence: PROTON (s2pul)
Solvent: cdcl3
Data collected on: Mar 3 2019
Temp. 25.0 C / 298.1 K
Operator: Chemler
Relax. delay 1.000 sec
Pulse 45.0 degrees
Acq. time 1.706 sec
Width 4800.8 Hz
32 repetitions
DATA PROCESSING
FT size 16384
Total time 1 min 27 sec
Agilent Technologies
ppm

SG_B2_P129_F24-25

Sample Name:

Data Collected on:
nmr300.chem.buffalo.edu-mercury300
Archive directory:

Sample directory:

FidFile: CARBON

Pulse Sequence: CARBON (s2pul)
Solvent: cdcl3
Data collected on: Mar 3 2019

Temp. 25.0 C / 298.1 K
Operator: Chemler

Relax. delay 2.000 sec
Pulse 45.0 degrees
Acq. time 0.868 sec
Width 18867.9 Hz
4760 repetitions
OBSERVE C13, 75.4536377 MHz
DECOUPLE H1, 300.0754430 MHz
Power 37 dB
continuously on
WALTZ-16 modulated
DATA PROCESSING
Line broadening 0.5 Hz
FT size 32768
Total time 7992 hr, 16 min

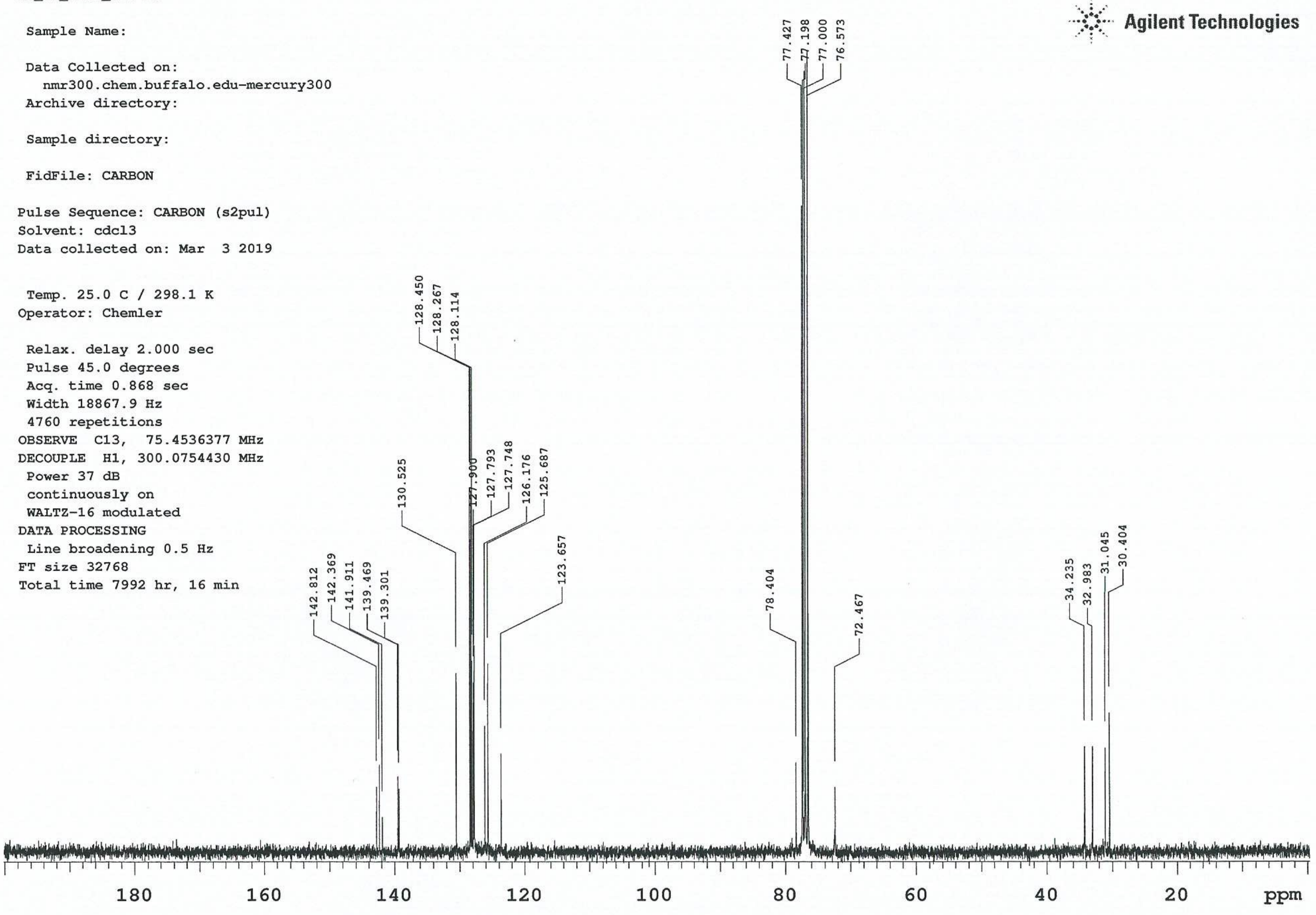

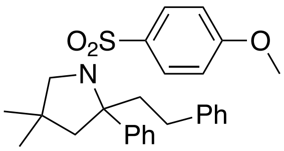
O2S
N
O
Ph
Ph

12a

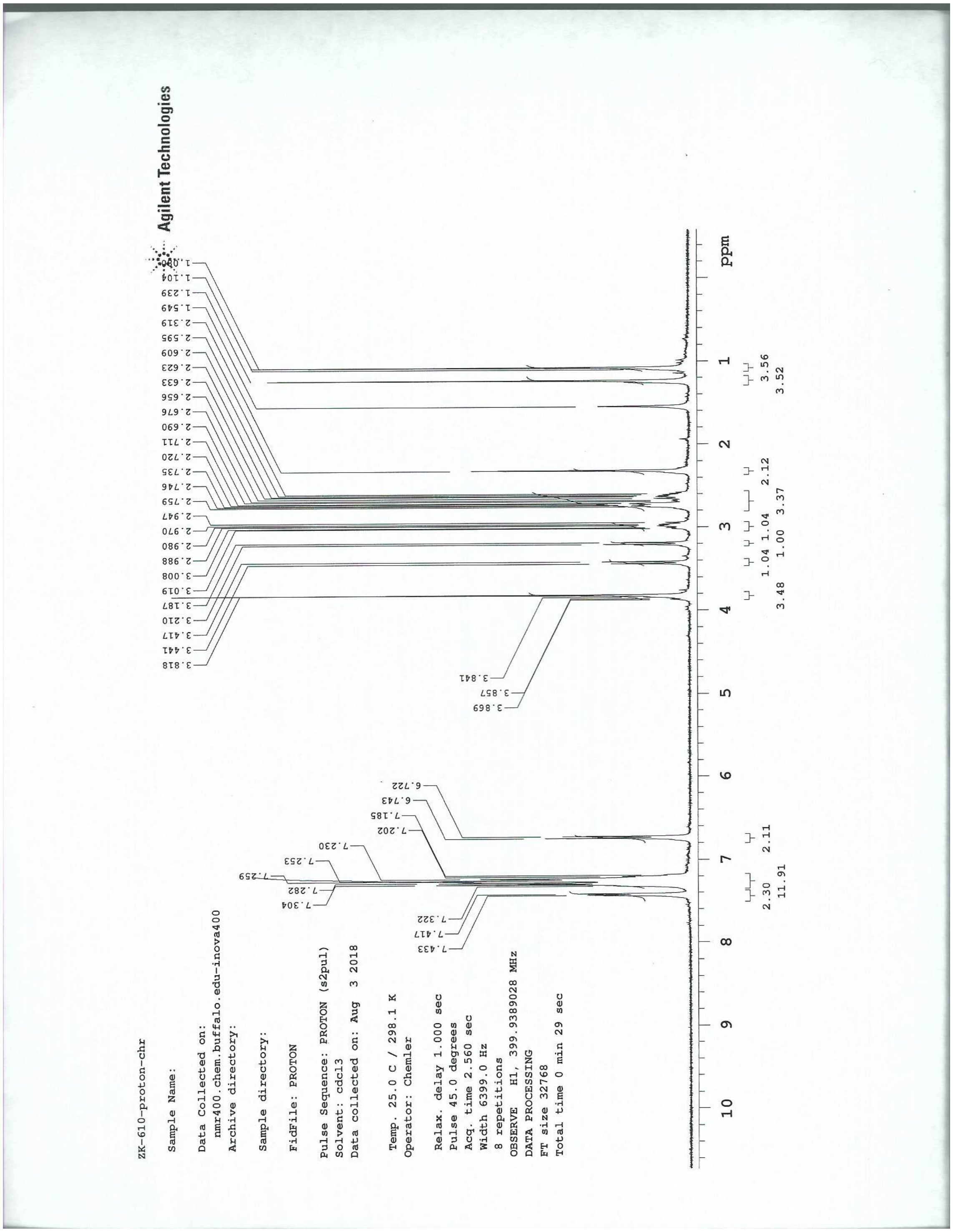
Agilent Technologies
ZK-610-proton-chr
Sample Name:
Data Collected on:
nmr400.chem.buffalo.edu-inova400
Archive directory:
Sample directory:
FidFile: PROTON
Pulse Sequence: PROTON (s2pul)
Solvent: cdcl3
Data collected on: Aug 3 2018
Temp. 25.0 C / 298.1 K
Operator: Chemler
Relax. delay 1.000 sec
Pulse 45.0 degrees
Acq. time 2.560 sec
Width 6399.0 Hz
8 repetitions
OBSERVE H1, 399.9389028 MHz
DATA PROCESSING
FT size 32768
Total time 0 min 29 sec
1.104
1.239
1.549
2.319
2.595
2.609
2.623
2.633
2.656
2.676
2.690
2.711
2.720
2.735
2.746
2.759
2.947
2.970
2.980
2.988
3.008
3.019
3.187
3.210
3.417
3.441
3.818
3.841
3.857
3.869
6.722
6.743
7.185
7.202
7.230
7.253
7.259
7.282
7.304
7.322
7.417
7.433
3.56
3.52
2.12
3.37
1.04
1.04
1.00
3.48
2.11
2.30
11.91
10
9
8
7
6
5
4
3
2
1
ppm

ZK-610-chr

Sample Name:

Data Collected on:
nmr300.chem.buffalo.edu-mercury300
Archive directory:

Sample directory:

FidFile: ZK-610-Carbon-chr

Pulse Sequence: CARBON (s2pul)
Solvent: cdcl3
Data collected on: Jul 31 2018

Operator: Chemler

Relax. delay 3.000 sec
Pulse 45.0 degrees
Acq. time 0.868 sec
Width 18867.9 Hz
3584 repetitions
OBSERVE C13, 75.4536377 MHz
DECOUPLE H1, 300.0754430 MHz
Power 37 dB
continuously on
WALTZ-16 modulated
DATA PROCESSING
Line broadening 0.5 Hz
FT size 32768
Total time 1329 hr, 38 min

Agilent Technologies

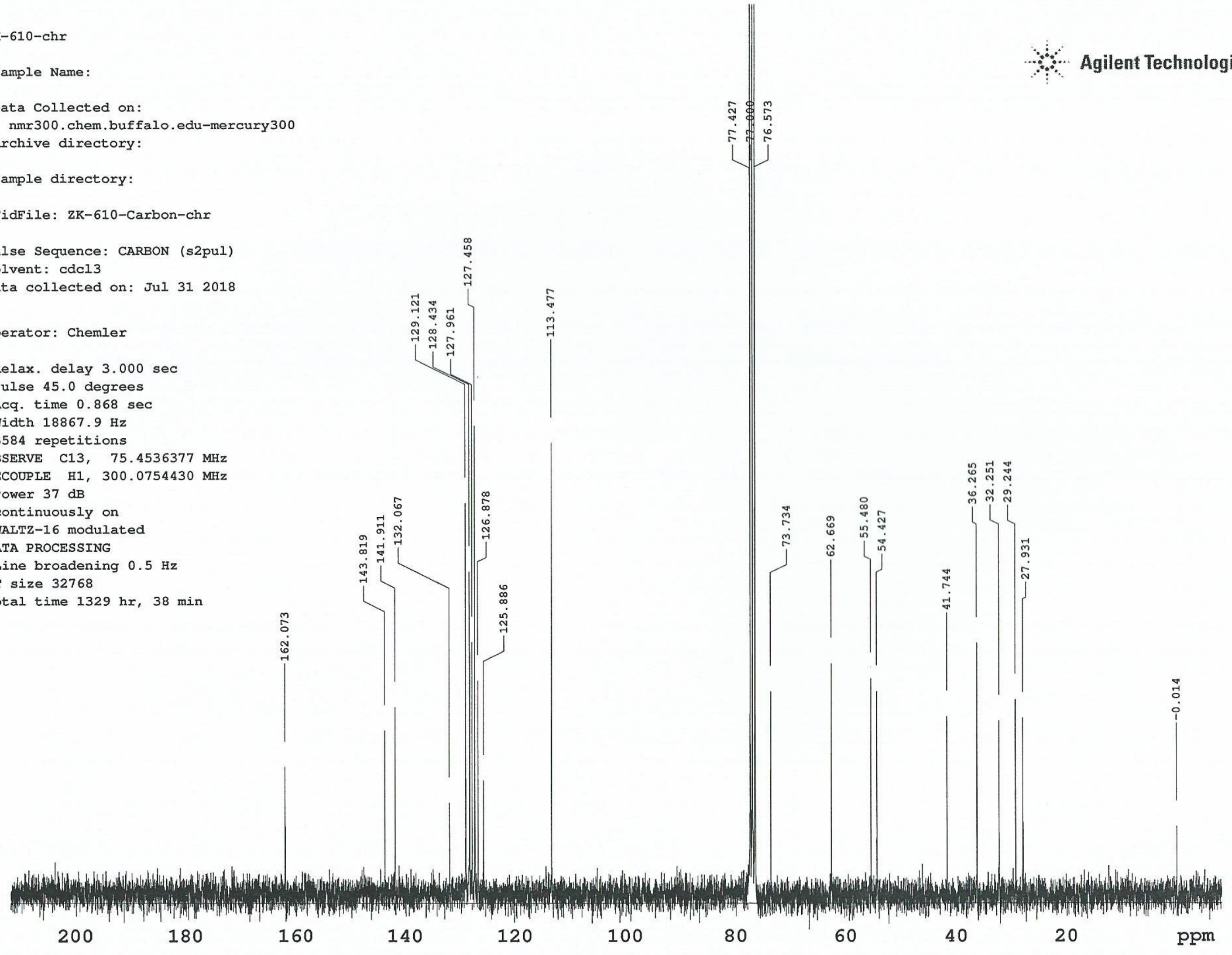

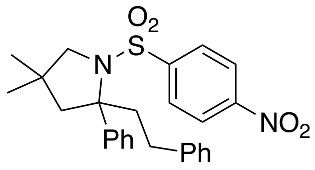

O2
S
N
NO2
Ph
Ph

12b

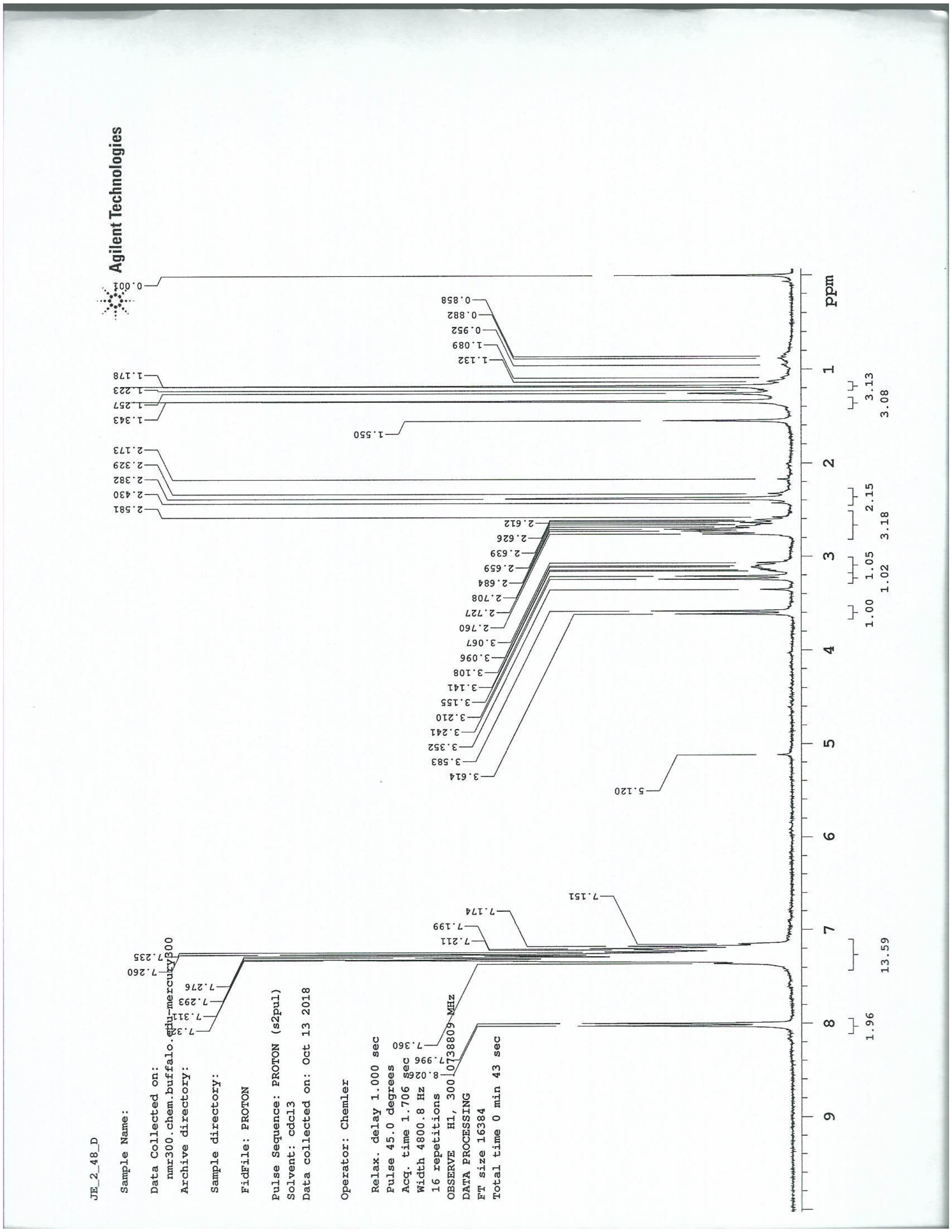

Agilent Technologies
JE_2_48_D
Sample Name:
Data Collected on:
nmr300.chem.buffalo.edu-mercury300
Archive directory:
Sample directory:
FidFile: PROTON
Pulse Sequence: PROTON (s2pul)
Solvent: cdcl3
Data collected on: Oct 13 2018
Operator: Chemler
Relax. delay 1.000 sec
Pulse 45.0 degrees
Acq. time 1.706 sec
Width 4800.8 Hz
16 repetitions
OBSERVE H1, 300.0738809 MHz
DATA PROCESSING
FT size 16384
Total time 0 min 43 sec
0.001
0.858
0.882
0.952
1.089
1.132
1.178
1.223
1.257
1.343
1.550
2.173
2.329
2.382
2.430
2.581
2.612
2.626
2.639
2.659
2.684
2.708
2.727
2.760
3.067
3.096
3.108
3.141
3.155
3.210
3.241
3.352
3.583
3.614
5.120
7.151
7.174
7.199
7.211
7.235
7.260
7.276
7.293
7.311
7.360
7.996
8.026
ppm
9
8
7
6
5
4
3
2
1
1.96
13.59
1.00
1.02
1.05
3.18
2.15
3.08
3.13

JE_2_48_D

Sample Name:

Data Collected on:
nmr300.chem.buffalo.edu-mercury300
Archive directory:

Sample directory:

FidFile: CARBON

Pulse Sequence: CARBON (s2pul)
Solvent: cdcl3
Data collected on: Oct 13 2018

Operator: Chemler

Relax. delay 2.000 sec
Pulse 45.0 degrees
Acq. time 0.868 sec
Width 18867.9 Hz
2992 repetitions
OBSERVE C13, 75.4536377 MHz
DECOUPLE H1, 300.0754430 MHz
Power 37 dB
continuously on
WALTZ-16 modulated
DATA PROCESSING
Line broadening 0.5 Hz
FT size 32768
Total time 79 hr, 55 min

Agilent Technologies

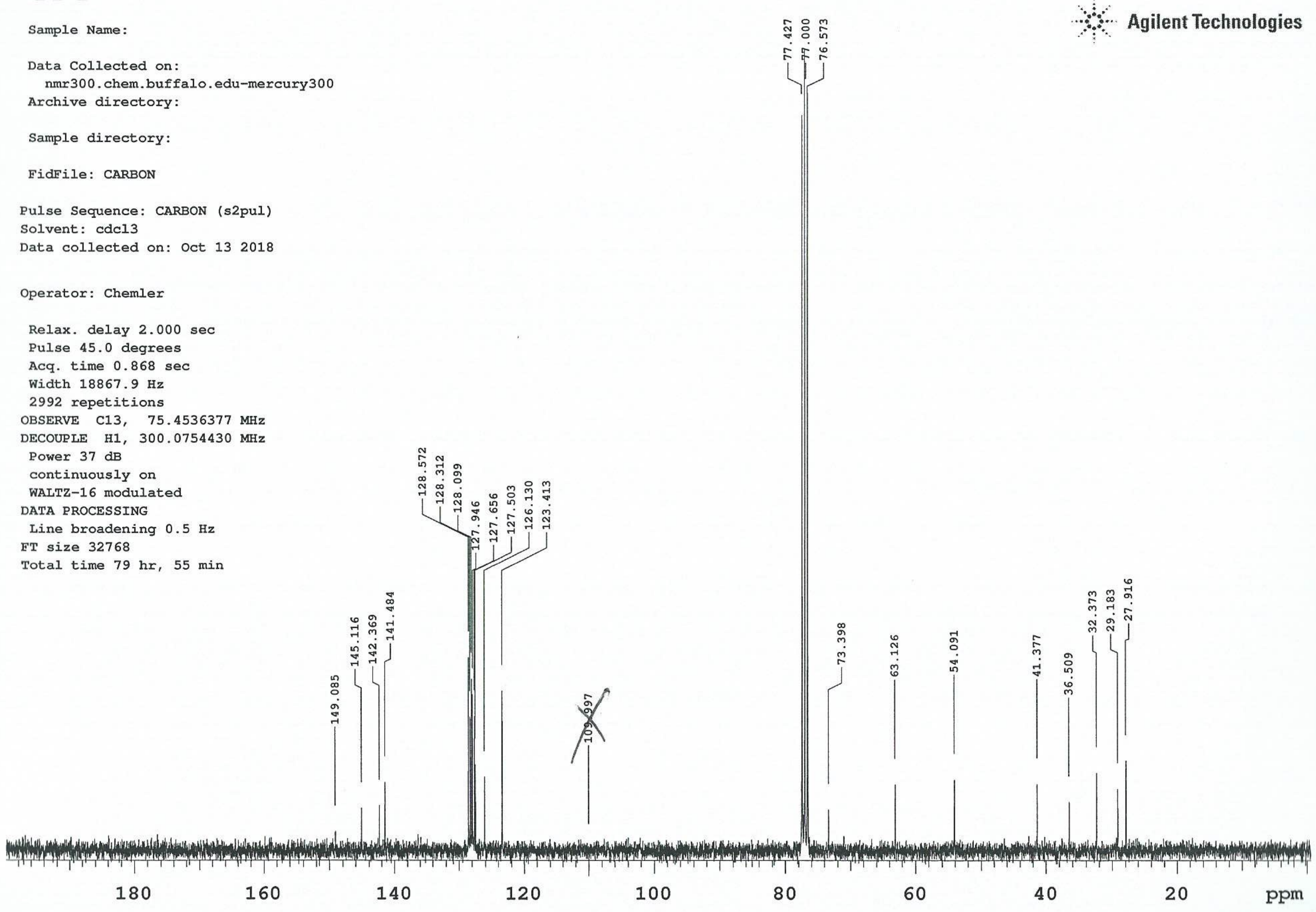

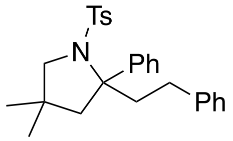
Ts
N
Ph
Ph

12c

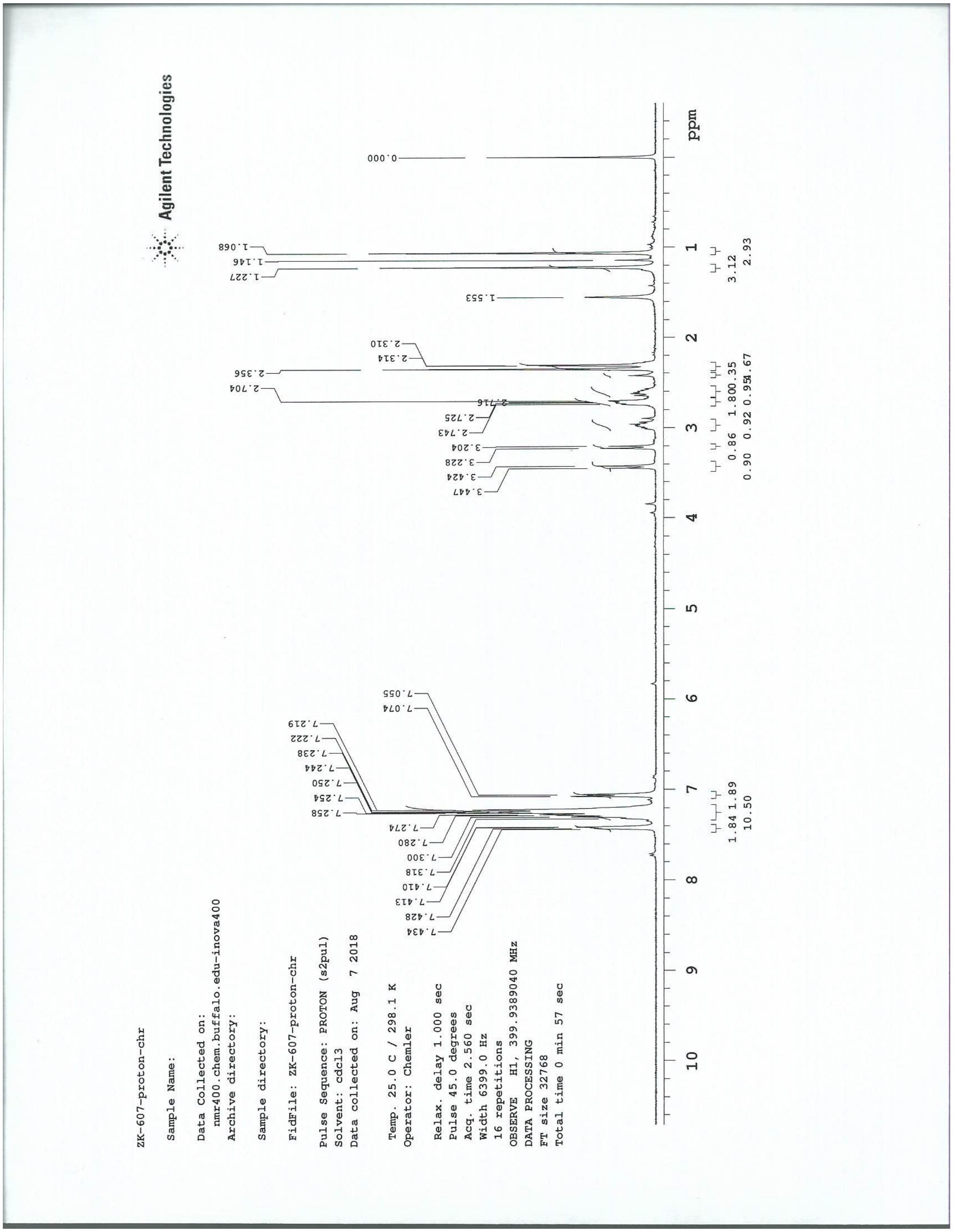
Agilent Technologies
ZK-607-proton-chr
Sample Name:
Data Collected on:
nmr400.chem.buffalo.edu-inova400
Archive directory:
Sample directory:
FidFile: ZK-607-proton-chr
Pulse Sequence: PROTON (s2pul)
Solvent: cdcl3
Data collected on: Aug 7 2018
Temp. 25.0 C / 298.1 K
Operator: Chemler
Relax. delay 1.000 sec
Pulse 45.0 degrees
Acq. time 2.560 sec
Width 6399.0 Hz
16 repetitions
OBSERVE H1, 399.9389040 MHz
DATA PROCESSING
FT size 32768
Total time 0 min 57 sec
ppm
0.000
1.068
1.146
1.227
1.553
2.310
2.314
2.356
2.704
2.716
2.725
2.743
3.204
3.228
3.424
3.447
7.055
7.074
7.219
7.222
7.238
7.244
7.250
7.254
7.258
7.274
7.280
7.300
7.318
7.410
7.413
7.428
7.434
3.12
2.93
0.90
0.86
0.92
1.80
0.95
0.35
1.67
1.84
1.89
10.50
10 9 8 7 6 5 4 3 2 1

cdk-b2-r512-spot1

Sample Name:

Data Collected on:
nmr300.chem.buffalo.edu-mercury300
Archive directory:

Sample directory:

FidFile: ZK-607-carbon-chr

Pulse Sequence: CARBON (s2pul)
Solvent: cdcl3
Data collected on: Aug 12 2018

Operator: Chemler

Relax. delay 1.000 sec
Pulse 45.0 degrees
Acq. time 0.868 sec
Width 18867.9 Hz
26360 repetitions
OBSERVE C13, 75.4536377 MHz
DECOUPLE H1, 300.0754430 MHz
Power 37 dB
continuously on
WALTZ-16 modulated
DATA PROCESSING
Line broadening 0.5 Hz
FT size 32768
Total time 643 hr, 45 min

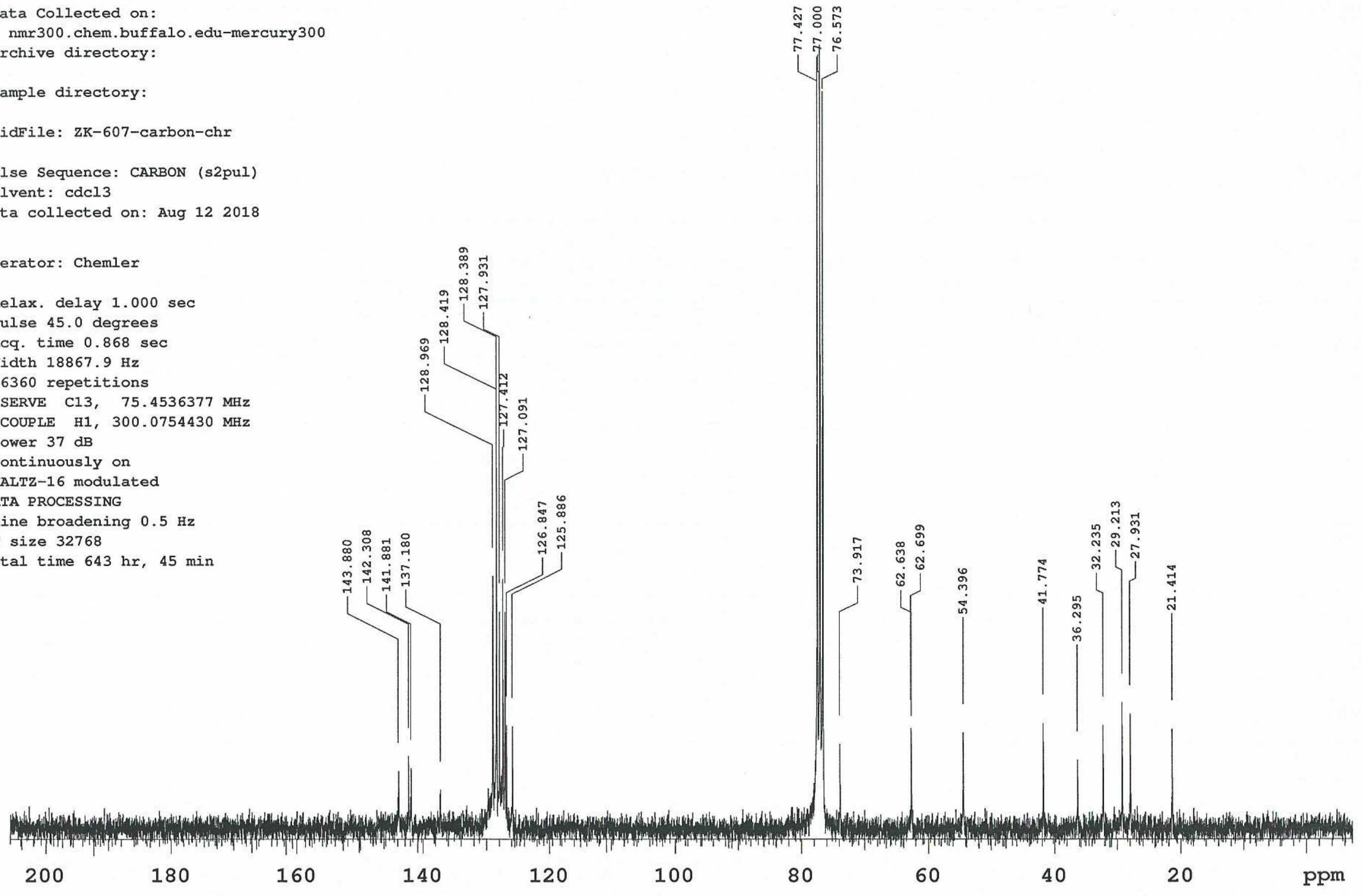

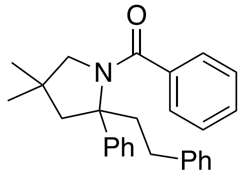
O
N
Ph
Ph

12d

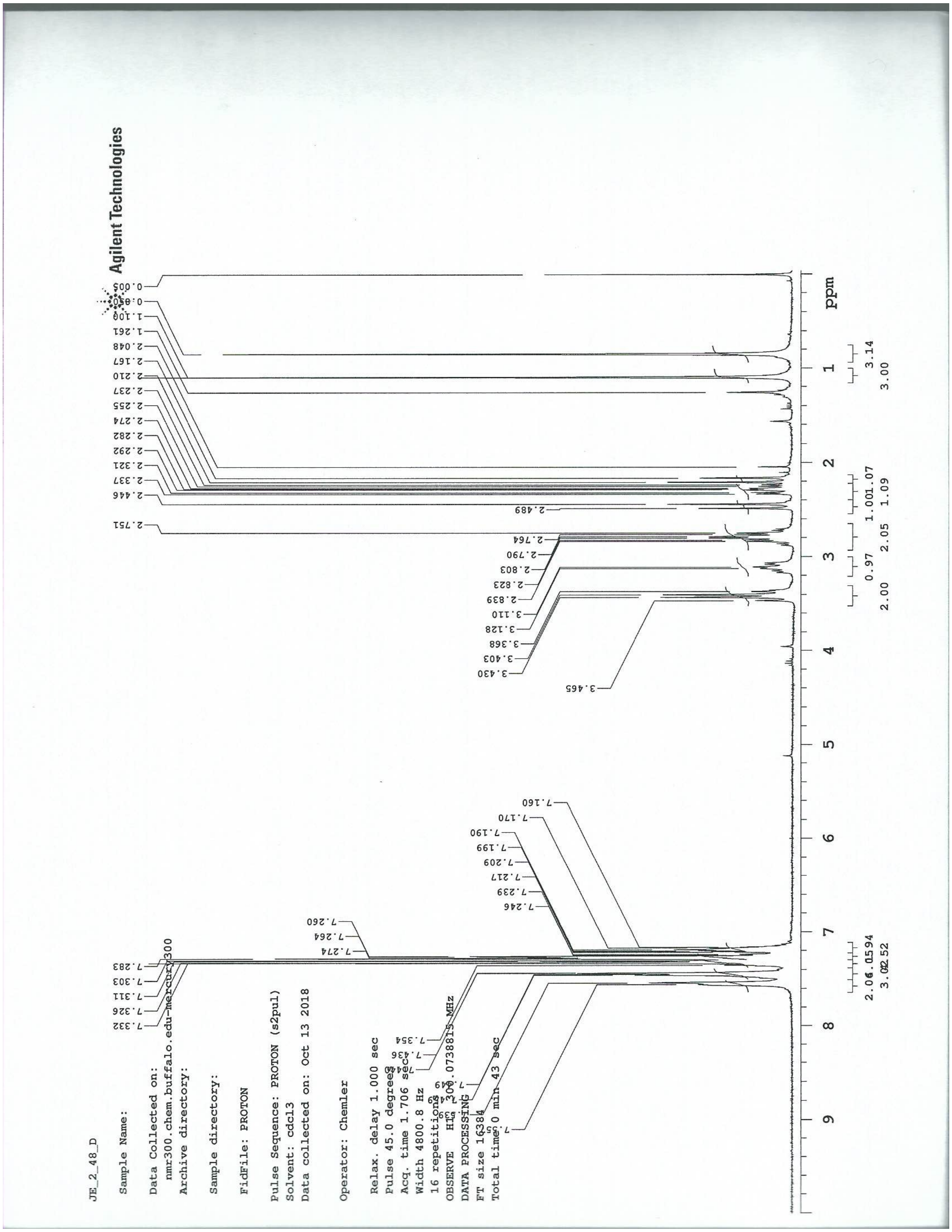
JE_2_48_D
Sample Name:
Data Collected on:
nmr300.chem.buffalo.edu-mercury300
Archive directory:
Sample directory:
FidFile: PROTON
Pulse Sequence: PROTON (s2pul)
Solvent: cdcl3
Data collected on: Oct 13 2018
Operator: Chemler
Relax. delay 1.000 sec
Pulse 45.0 degrees
Acq. time 1.706 sec
Width 4800.8 Hz
16 repetitions
OBSERVE H1, 300.0738815 MHz
DATA PROCESSING
FT size 16384
Total time 0 min 43 sec
Agilent Technologies
ppm

Agilent Technologies

JE_2_48_D

Sample Name:

Data Collected on:
nmr300.chem.buffalo.edu-mercury300
Archive directory:

Sample directory:

FidFile: CARBON

Pulse Sequence: CARBON (s2pul)
Solvent: cdcl3
Data collected on: Oct 14 2018

Operator: Chemler

Relax. delay 2.000 sec
Pulse 45.0 degrees
Acq. time 0.868 sec
Width 18867.9 Hz
2656 repetitions
OBSERVE C13, 75.4536377 MHz
DECOUPLE H1, 300.0754430 MHz
Power 37 dB
continuously on
WALTZ-16 modulated
DATA PROCESSING
Line broadening 0.5 Hz
FT size 32768
Total time 79 hr, 55 min

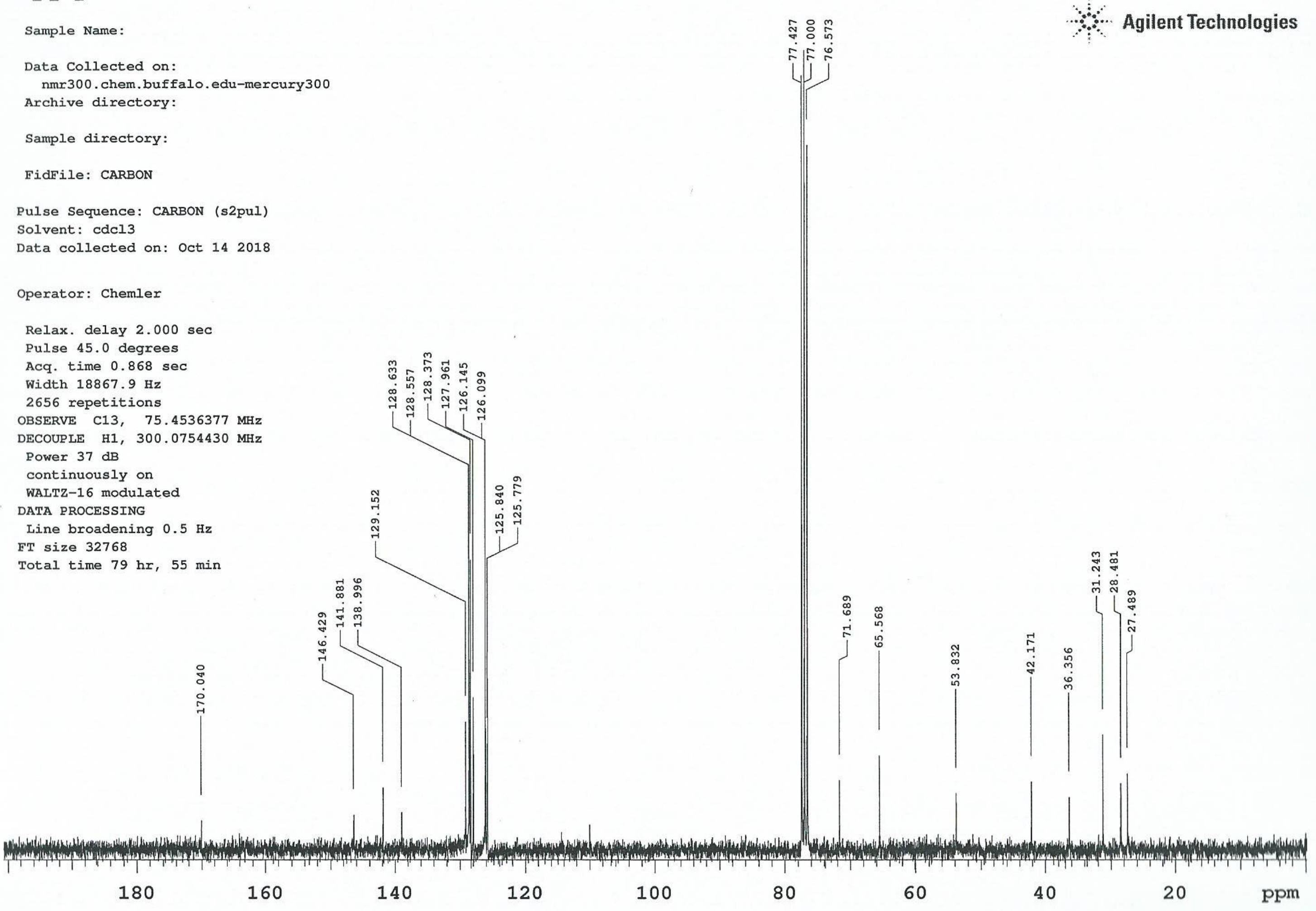

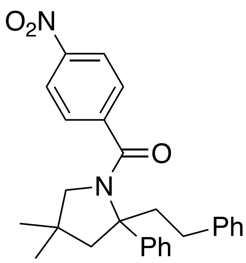

O2N
N
O
Ph
Ph

12e

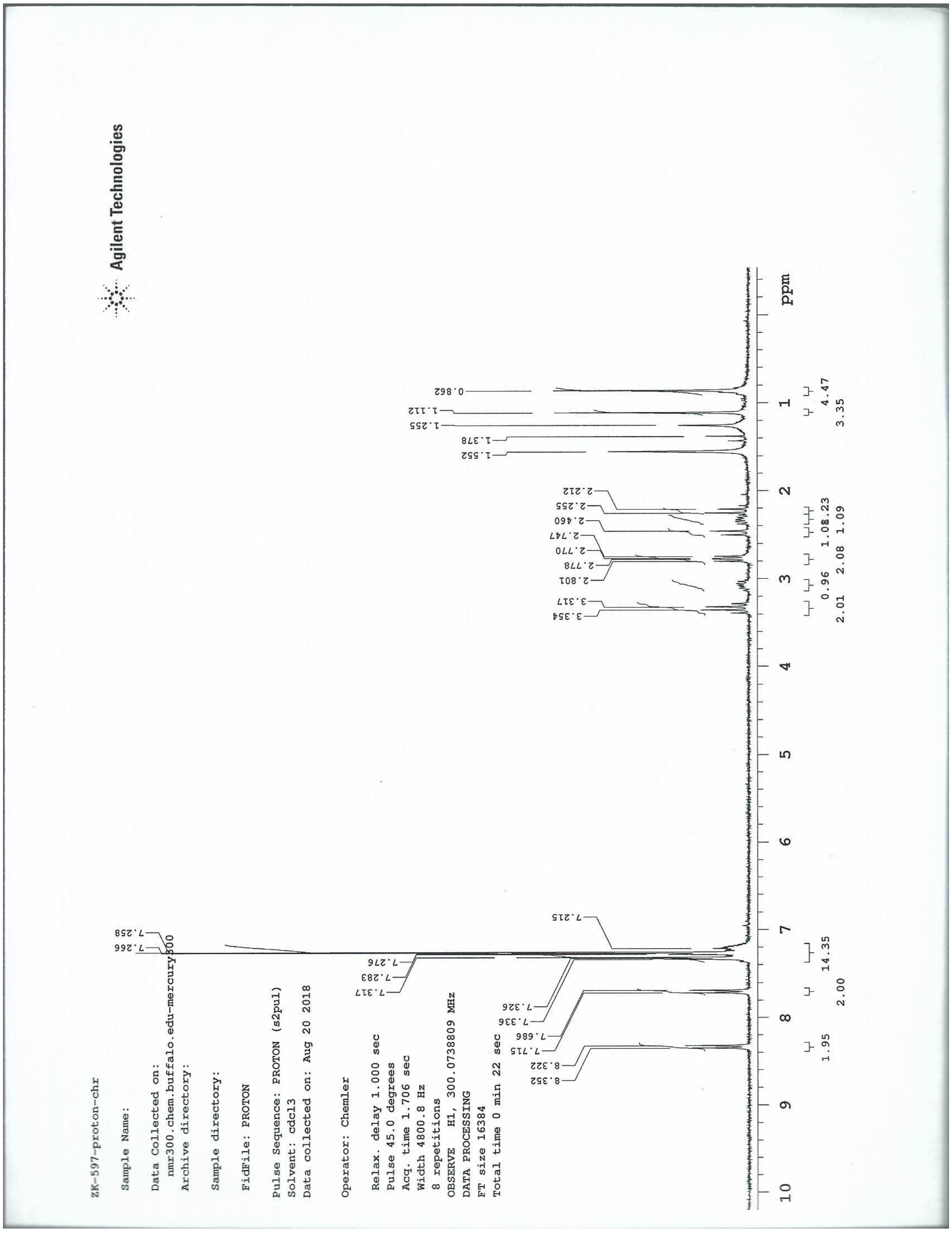

ZK-597-proton-chr
Sample Name:
Data Collected on:
nmr300.chem.buffalo.edu-mercury300
Archive directory:
Sample directory:
FidFile: PROTON
Pulse Sequence: PROTON (s2pul)
Solvent: cdcl3
Data collected on: Aug 20 2018
Operator: Chemler
Relax. delay 1.000 sec
Pulse 45.0 degrees
Acq. time 1.706 sec
Width 4800.8 Hz
8 repetitions
OBSERVE H1, 300.0738809 MHz
DATA PROCESSING
FT size 16384
Total time 0 min 22 sec
Agilent Technologies
ppm

ZK597V

Sample Name:

Data Collected on:
nmr300.chem.buffalo.edu-mercury300
Archive directory:

Sample directory:

FidFile: ZK597V-C13-1

Pulse Sequence: CARBON (s2pul)
Solvent: cdcl3
Data collected on: Aug 26 2018

Operator: Chemler

Relax. delay 1.000 sec
Pulse 45.0 degrees
Acq. time 0.868 sec
Width 18867.9 Hz
15372 repetitions
OBSERVE C13, 75.4536377 MHz
DECOUPLE H1, 300.0754430 MHz
Power 37 dB
continuously on
WALTZ-16 modulated
DATA PROCESSING
Line broadening 1.0 Hz
FT size 32768
Total time 6379 hr, 1 min

Agilent Technologies

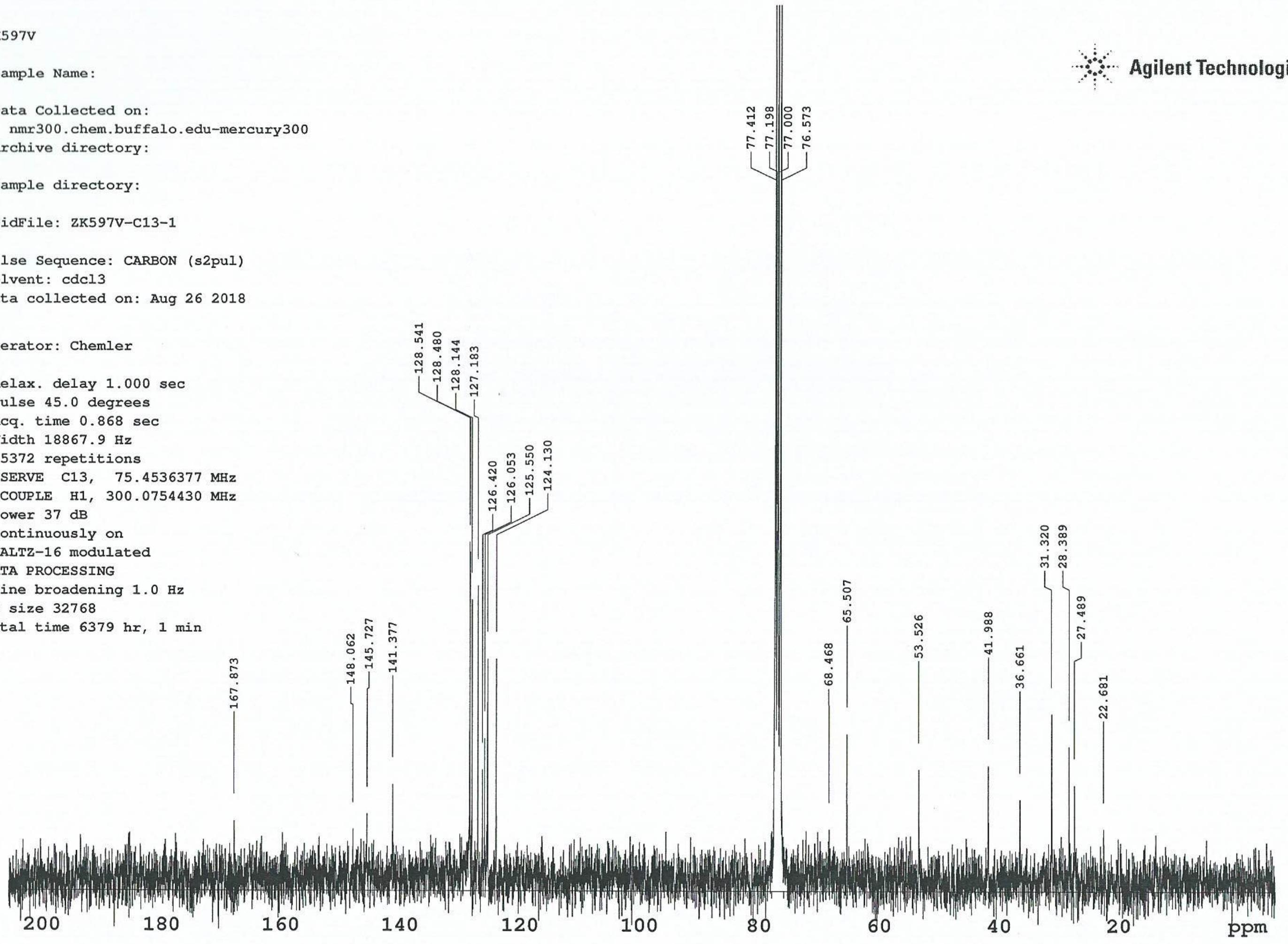

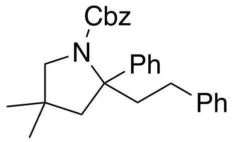

Cbz
N
Ph
Ph

12f

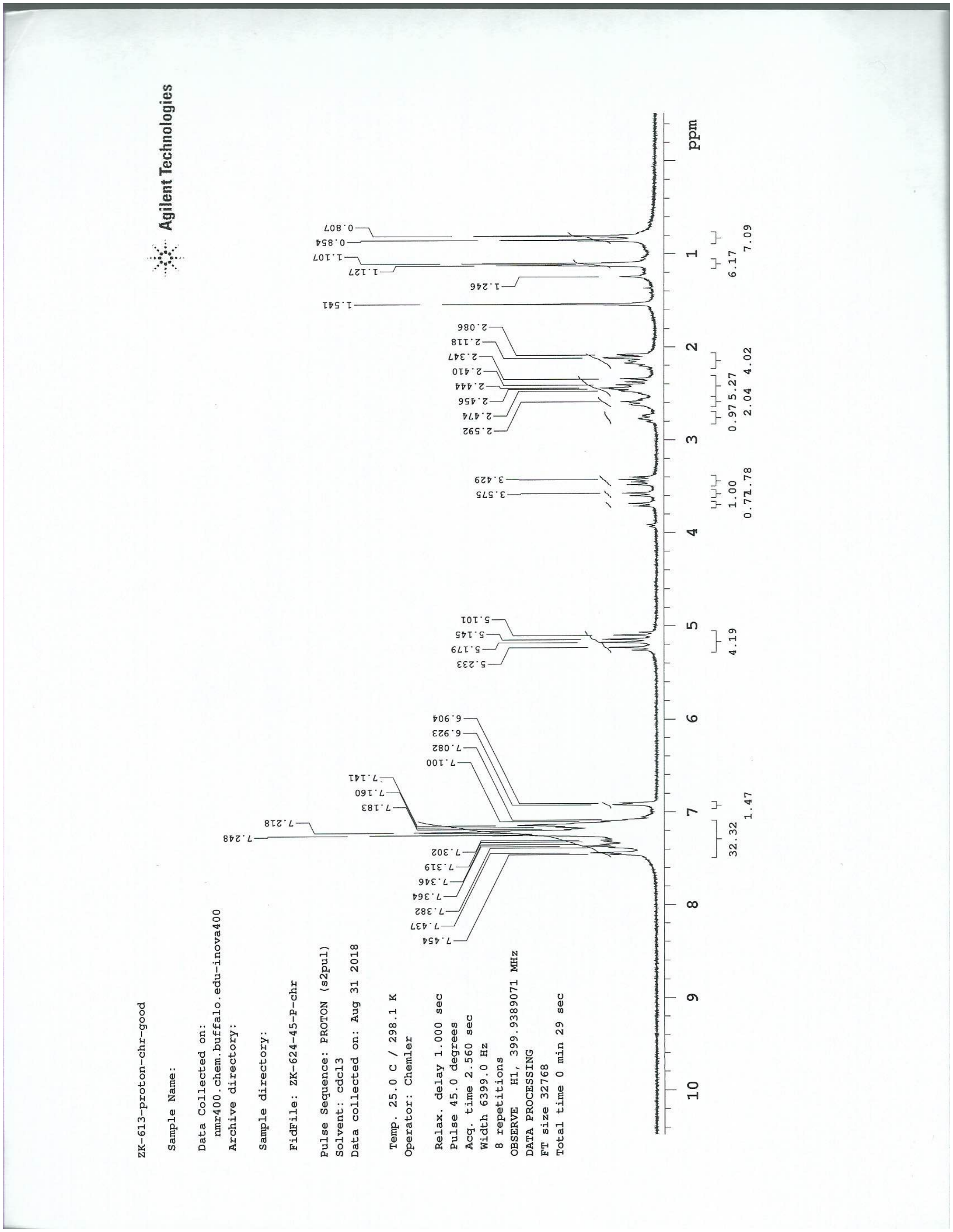

Agilent Technologies
ZK-613-proton-chr-good
Sample Name:
Data Collected on:
nmr400.chem.buffalo.edu-inova400
Archive directory:
Sample directory:
FidFile: ZK-624-45-P-chr
Pulse Sequence: PROTON (s2pul)
Solvent: cdcl3
Data collected on: Aug 31 2018
Temp. 25.0 C / 298.1 K
Operator: Chemler
Relax. delay 1.000 sec
Pulse 45.0 degrees
Acq. time 2.560 sec
Width 6399.0 Hz
8 repetitions
OBSERVE H1, 399.9389071 MHz
DATA PROCESSING
FT size 32768
Total time 0 min 29 sec
7.454
7.437
7.382
7.364
7.346
7.319
7.302
7.248
7.218
7.183
7.160
7.141
7.100
7.082
6.923
6.904
5.233
5.179
5.145
5.101
3.575
3.429
2.592
2.474
2.456
2.444
2.410
2.347
2.118
2.086
1.541
1.246
1.127
1.107
0.854
0.807
32.32
1.47
4.19
1.00
0.771.78
0.97
5.27
2.04
4.02
6.17
7.09
10
9
8
7
6
5
4
3
2
1
ppm

	Parameter	Value
1	Spectrometer	mercury
2	Solvent	cdcl3
3	Temperature	24.0
4	Pulse Sequence	s2pul
5	Experiment	1D
6	Probe	5mm_ASW_PFG
7	Receiver Gain	30
8	Relaxation Delay	3.0000
9	Pulse Width	6.5000
10	Presaturation Frequency	
11	Acquisition Time	0.8684
12	Acquisition Date	2018-09-01T20:48:14
13	Spectrometer Frequency	75.46
14	Spectral Width	18867.9
15	Lowest Frequency	-1133.7
16	Nucleus	13C
17	Acquired Size	16384
18	Spectral Size	32768

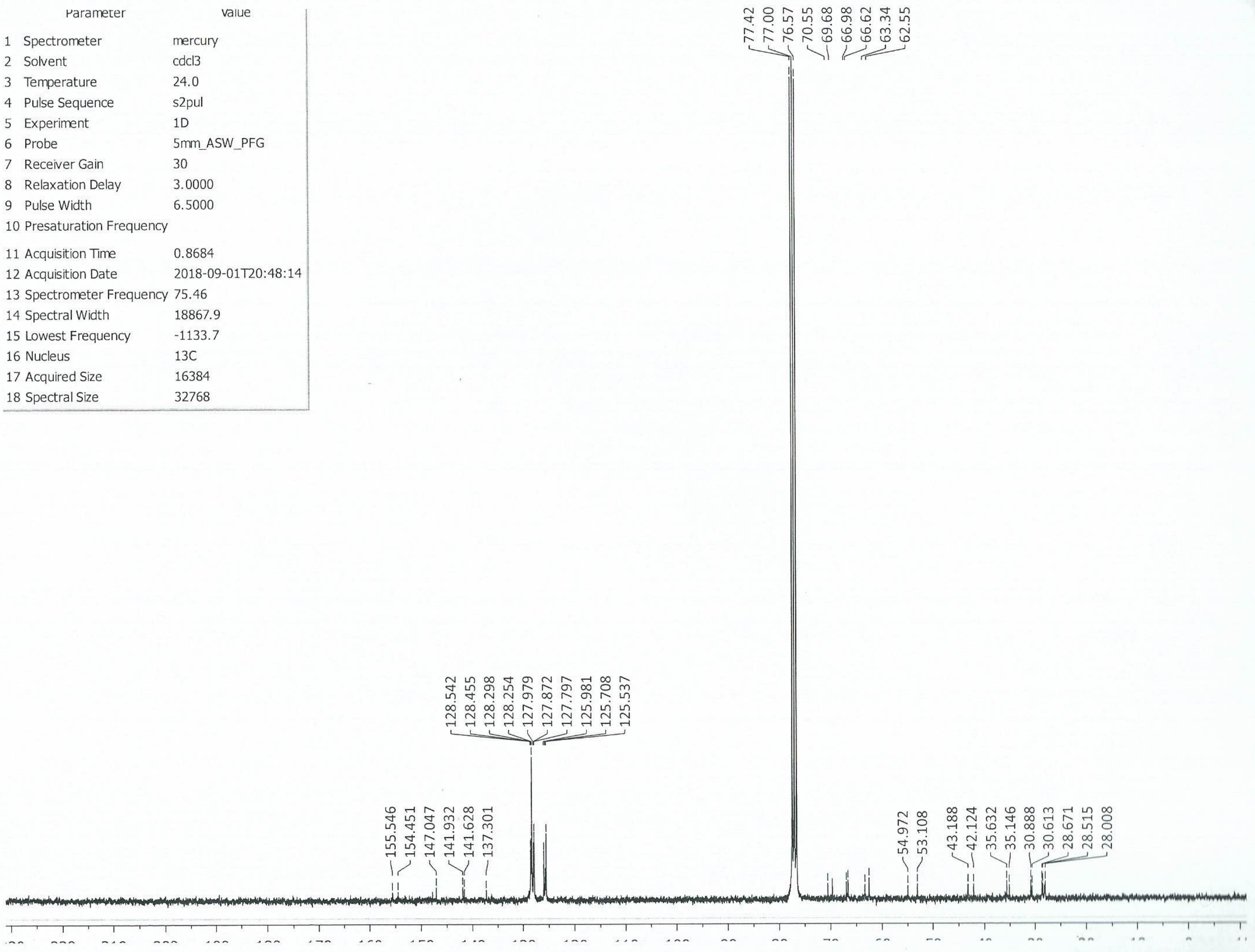

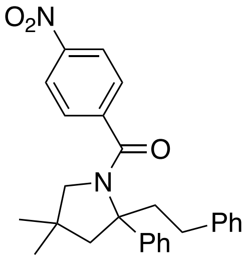

O_2N
O
N
Ph
Ph

12g

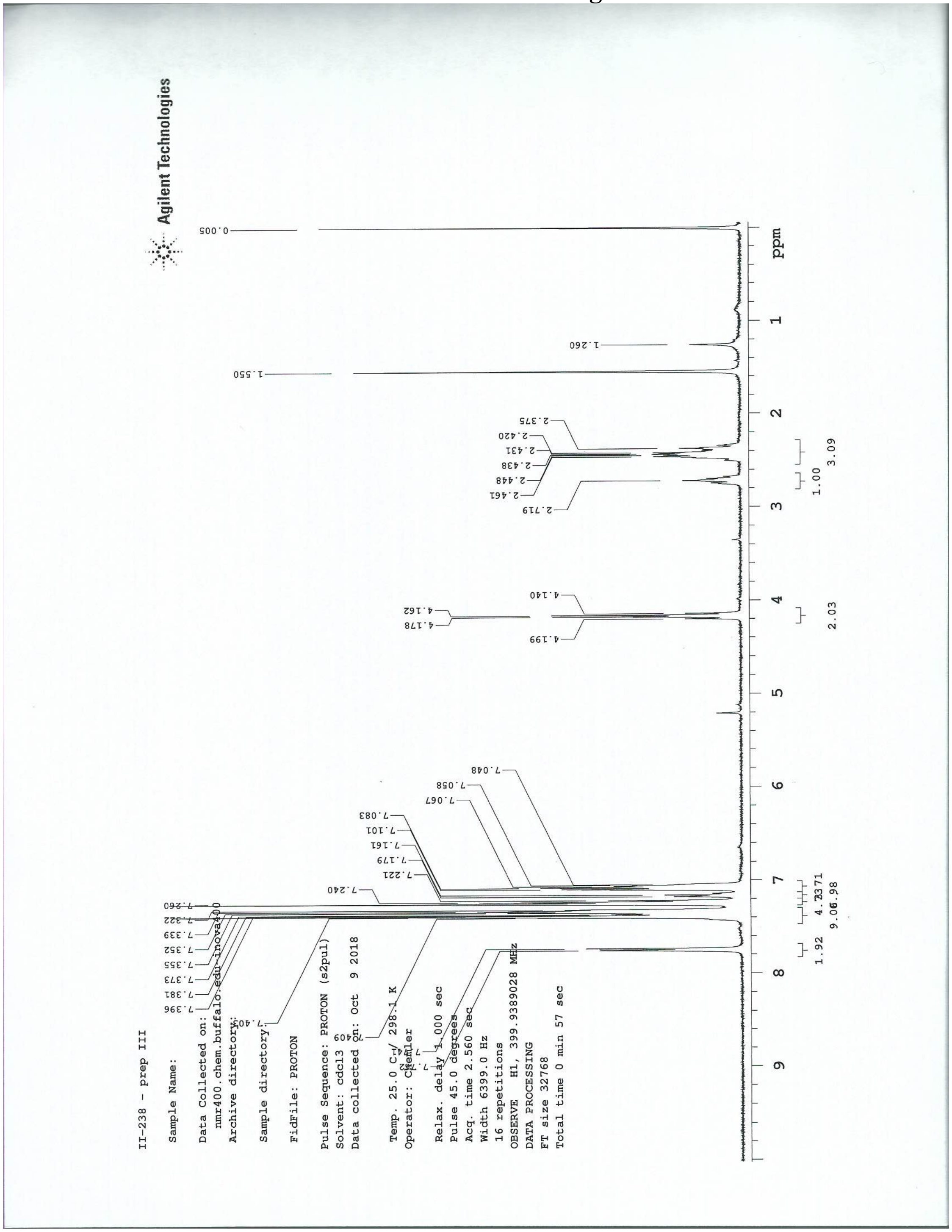

Agilent Technologies
II-238 - prep III
Sample Name:
Data Collected on: nmr400.chem.buffalo.edu-inova400
Archive directory:
Sample directory:
FidFile: PROTON
Pulse Sequence: PROTON (s2pul)
Solvent: cdcl3
Data collected on: Oct 9 2018
Temp. 25.0 C / 298.1 K
Operator: Chemler
Relax. delay 1.000 sec
Pulse 45.0 degrees
Acq. time 2.560 sec
Width 6399.0 Hz
16 repetitions
OBSERVE H1, 399.9389028 MHz
DATA PROCESSING
FT size 32768
Total time 0 min 57 sec
ppm
0.005
1.260
1.550
2.375
2.420
2.431
2.438
2.448
2.461
2.719
4.140
4.162
4.178
4.199
7.048
7.058
7.067
7.083
7.101
7.161
7.179
7.221
7.240
7.260
7.322
7.339
7.352
7.355
7.373
7.381
7.396
3.09
1.00
2.03
1.92
9.06

JE_2_48_crude

Sample Name:

Data Collected on:
nmr400.chem.buffalo.edu-inova400
Archive directory:

Sample directory:

FidFile: CARBON

Pulse Sequence: CARBON (s2pul)
Solvent: cdcl3
Data collected on: Oct 13 2018

Temp. 25.0 C / 298.1 K
Operator: Chemler

Relax. delay 2.000 sec
Pulse 45.0 degrees
Acq. time 1.303 sec
Width 25141.4 Hz
14928 repetitions
OBSERVE C13, 100.5647169 MHz
DECOUPLE H1, 399.9409068 MHz
Power 33 dB
continuously on
WALTZ-16 modulated
DATA PROCESSING
Line broadening 0.5 Hz
FT size 65536
Total time 920 hr, 3 min

Agilent Technologies

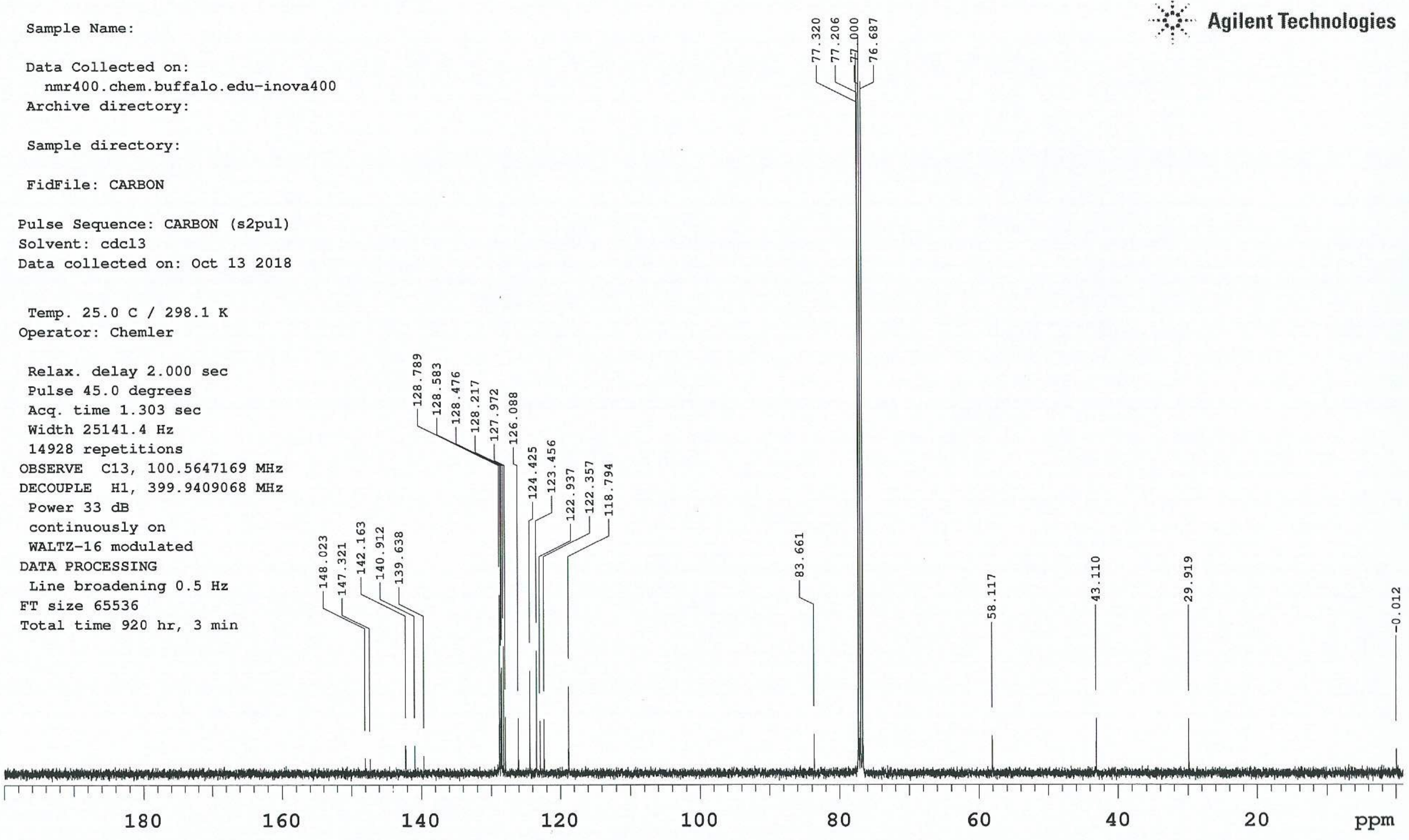

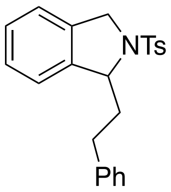
NTs
Ph

13

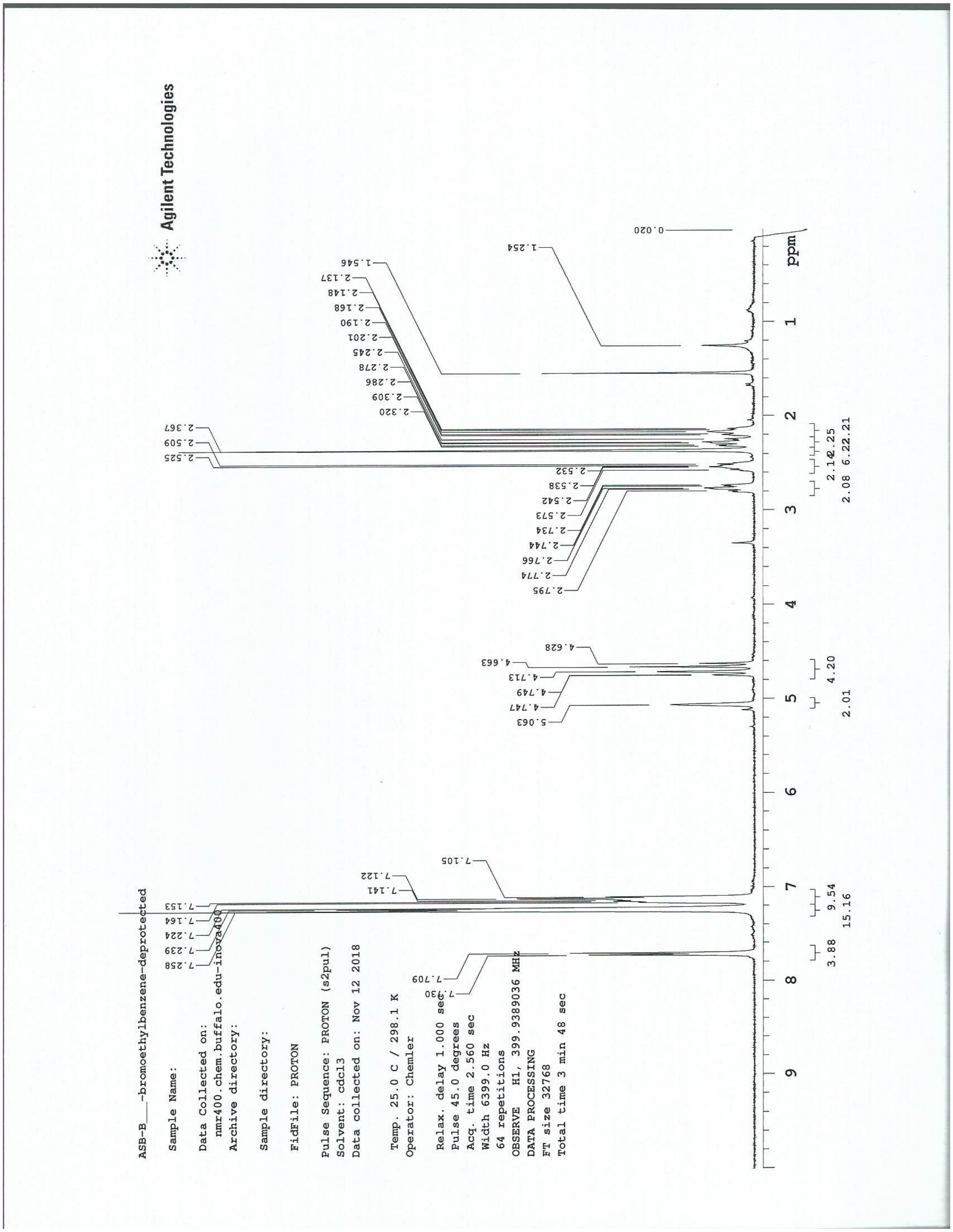
Agilent Technologies
ASB-B___-bromoethylbenzene-deprotected
Sample Name:
Data Collected on:
nmr400.chem.buffalo.edu-inova400
Archive directory:
Sample directory:
FidFile: PROTON
Pulse Sequence: PROTON (s2pul)
Solvent: cdcl3
Data collected on: Nov 12 2018
Temp. 25.0 C / 298.1 K
Operator: Chemler
Relax. delay 1.000 sec
Pulse 45.0 degrees
Acq. time 2.560 sec
Width 6399.0 Hz
64 repetitions
OBSERVE H1, 399.9389036 MHz
DATA PROCESSING
FT size 32768
Total time 3 min 48 sec
ppm

ASB-B___-bromoethylbenzene-deprotected

Sample Name:

Data Collected on:
nmr400.chem.buffalo.edu-inova400
Archive directory:

Sample directory:

FidFile: CARBON

Pulse Sequence: CARBON (s2pul)
Solvent: cdcl3
Data collected on: Nov 12 2018

Temp. 25.0 C / 298.1 K
Operator: Chemler

Relax. delay 2.000 sec
Pulse 45.0 degrees
Acq. time 1.303 sec
Width 25141.4 Hz
15592 repetitions
OBSERVE C13, 100.5647169 MHz
DECOUPLE H1, 399.9409068 MHz
Power 33 dB
continuously on
WALTZ-16 modulated
DATA PROCESSING
Line broadening 0.5 Hz
FT size 65536
Total time 920 hr, 3 min

Agilent Technologies

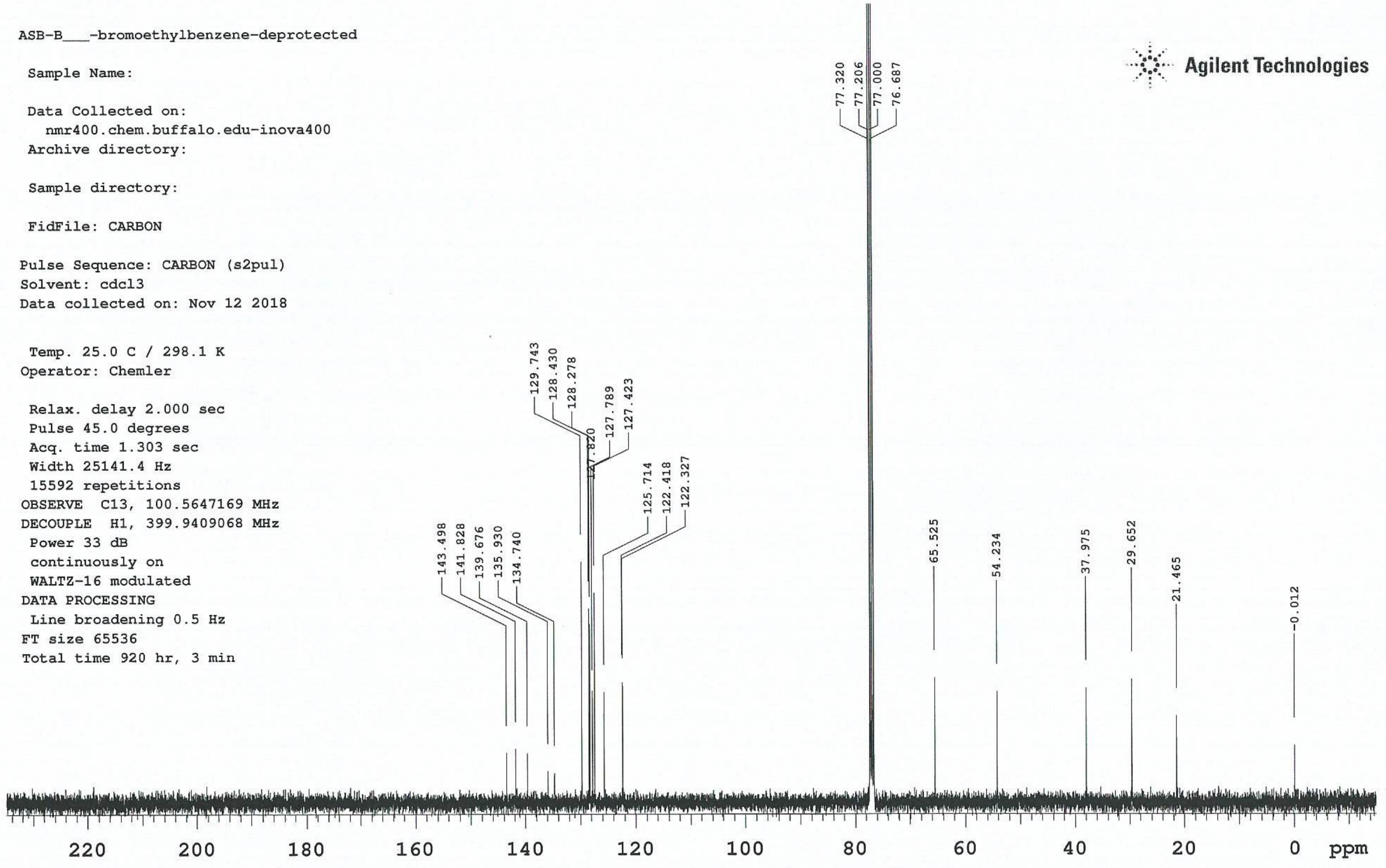

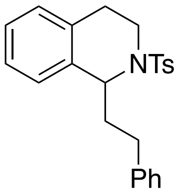
NTs
Ph

14

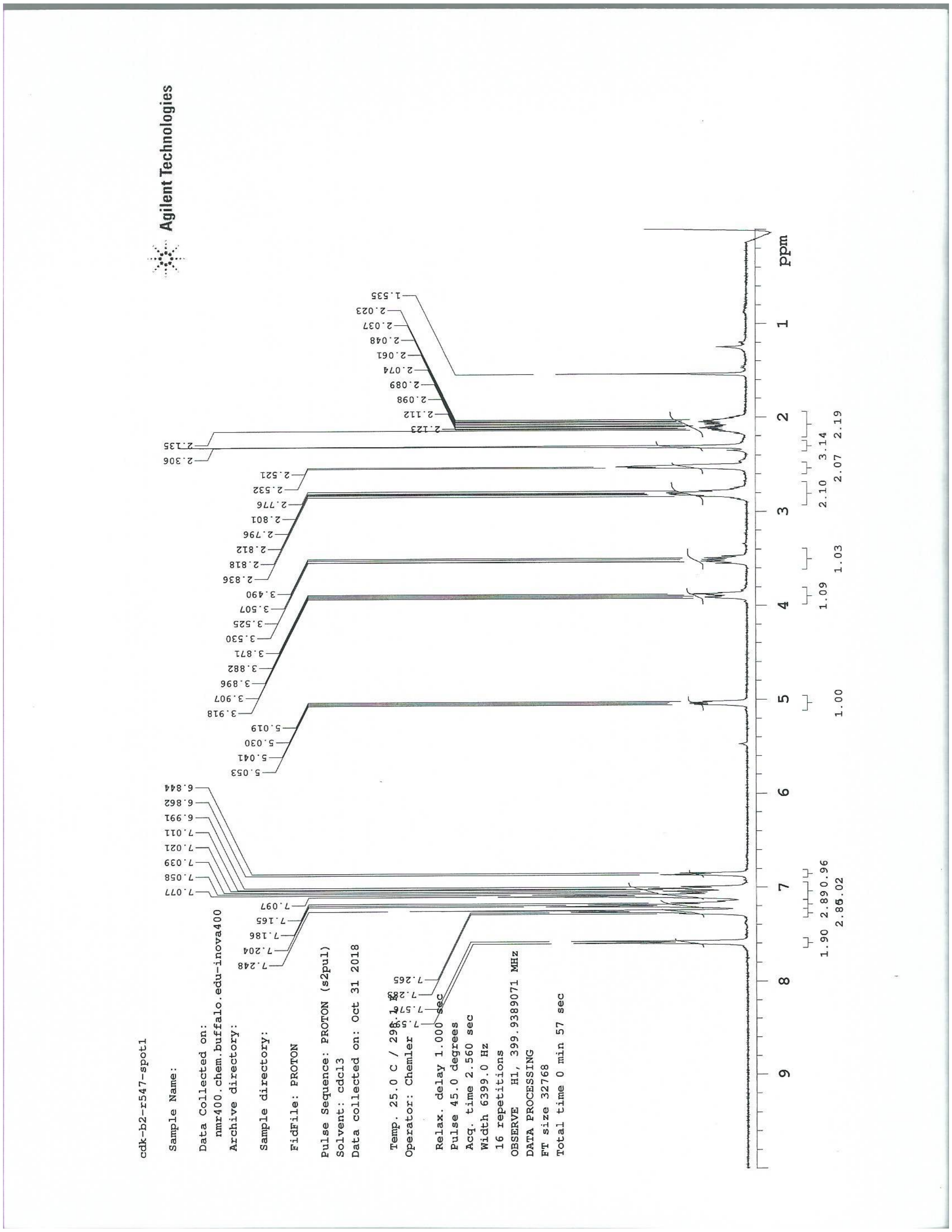
Agilent Technologies
cdk-b2-r547-spot1
Sample Name:
Data Collected on:
nmr400.chem.buffalo.edu-inova400
Archive directory:
Sample directory:
FidFile: PROTON
Pulse Sequence: PROTON (s2pul)
Solvent: cdcl3
Data collected on: Oct 31 2018
Temp. 25.0 C / 298.1 K
Operator: Chemler
Relax. delay 1.000 sec
Pulse 45.0 degrees
Acq. time 2.560 sec
Width 6399.0 Hz
16 repetitions
OBSERVE H1, 399.9389071 MHz
DATA PROCESSING
FT size 32768
Total time 0 min 57 sec
ppm

II-242B - crude

Sample Name:

Data Collected on:
nmr400.chem.buffalo.edu-inova400
Archive directory:

Sample directory:

FidFile: CARBON

Pulse Sequence: CARBON (s2pul)
Solvent: cdcl3
Data collected on: Nov 1 2018

Temp. 25.0 C / 298.1 K
Operator: Chemler

Relax. delay 2.000 sec
Pulse 45.0 degrees
Acq. time 1.303 sec
Width 25141.4 Hz
2088 repetitions
OBSERVE C13, 100.5647177 MHz
DECOUPLE H1, 399.9409068 MHz
Power 33 dB
continuously on
WALTZ-16 modulated
DATA PROCESSING
Line broadening 0.5 Hz
FT size 65536
Total time 920 hr, 3 min

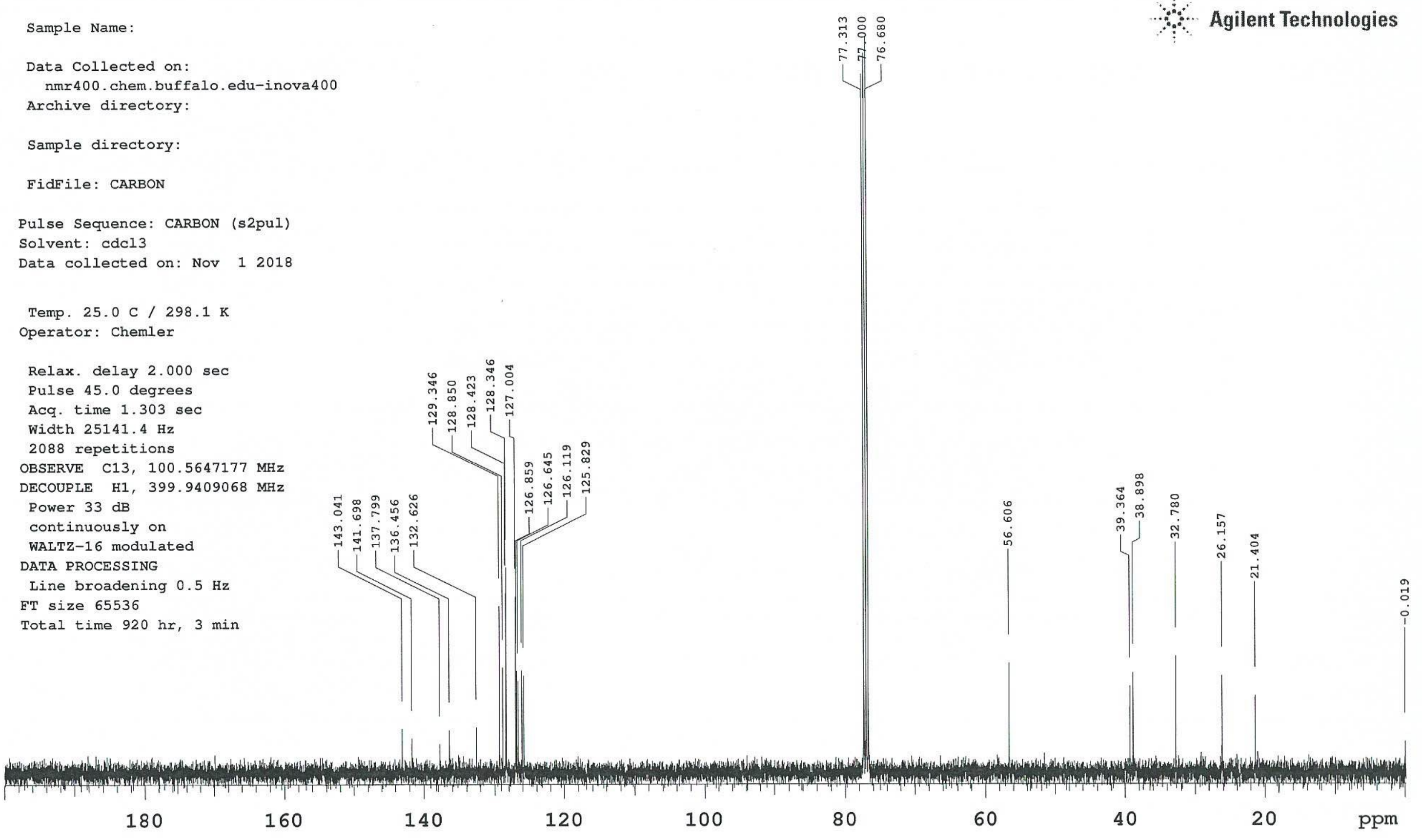

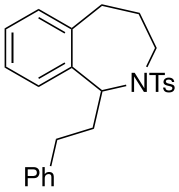

15

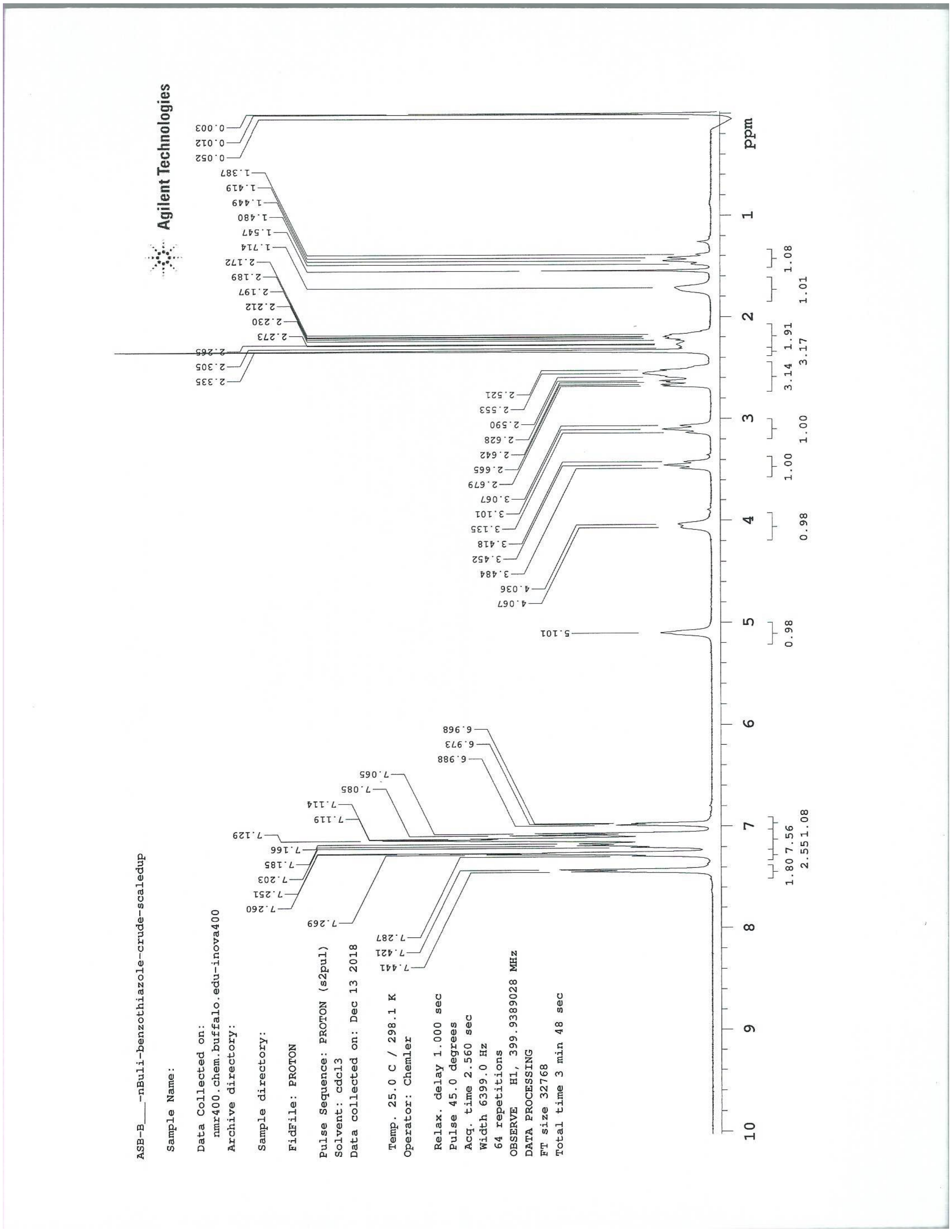
Agilent Technologies
ASB-B___-nBuli-benzothiazole-crude-scaledup
Sample Name:
Data Collected on:
nmr400.chem.buffalo.edu-inova400
Archive directory:
Sample directory:
FidFile: PROTON
Pulse Sequence: PROTON (s2pul)
Solvent: cdcl3
Data collected on: Dec 13 2018
Temp. 25.0 C / 298.1 K
Operator: Chemler
Relax. delay 1.000 sec
Pulse 45.0 degrees
Acq. time 2.560 sec
Width 6399.0 Hz
64 repetitions
OBSERVE H1, 399.9389028 MHz
DATA PROCESSING
FT size 32768
Total time 3 min 48 sec
0.003 0.012 0.052 1.387 1.419 1.449 1.480 1.547 1.714 2.172 2.189 2.197 2.212 2.230 2.273 2.265 2.305 2.335
2.521 2.553 2.590 2.628 2.642 2.665 2.679 3.067 3.101 3.135 3.418 3.452 3.484 4.036 4.067
5.101
6.968 6.973 6.988 7.065 7.085 7.114 7.119 7.129 7.166 7.185 7.203 7.251 7.260 7.269 7.287 7.421 7.441
1.08 1.01 1.91 3.14 3.17 1.00 1.00 0.98 0.98 1.80 7.56 2.55 1.08
10 9 8 7 6 5 4 3 2 1 ppm

JE_2_77_Crude

Sample Name:

Data Collected on:
nmr400.chem.buffalo.edu-inova400
Archive directory:

Sample directory:

FidFile: CARBON

Pulse Sequence: CARBON (s2pul)
Solvent: cdcl3
Data collected on: Dec 13 2018

Temp. 25.0 C / 298.1 K
Operator: Chemler

Relax. delay 2.000 sec
Pulse 45.0 degrees
Acq. time 1.303 sec
Width 25141.4 Hz
14832 repetitions
OBSERVE C13, 100.5647177 MHz
DECOUPLE H1, 399.9409068 MHz
Power 33 dB
continuously on
WALTZ-16 modulated
DATA PROCESSING
Line broadening 0.5 Hz
FT size 65536
Total time 92 hr

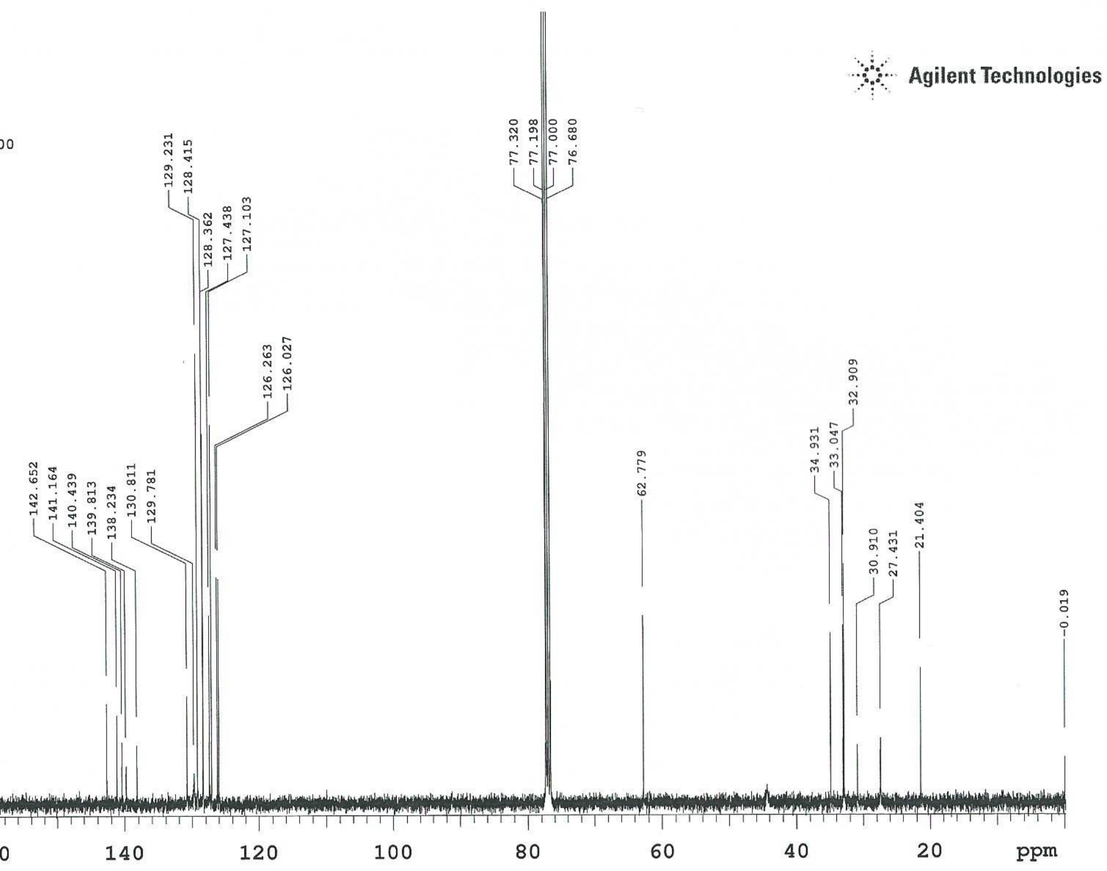

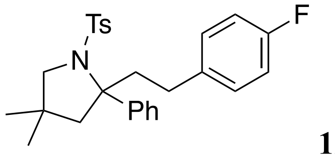
Ts
N
F
Ph

12h

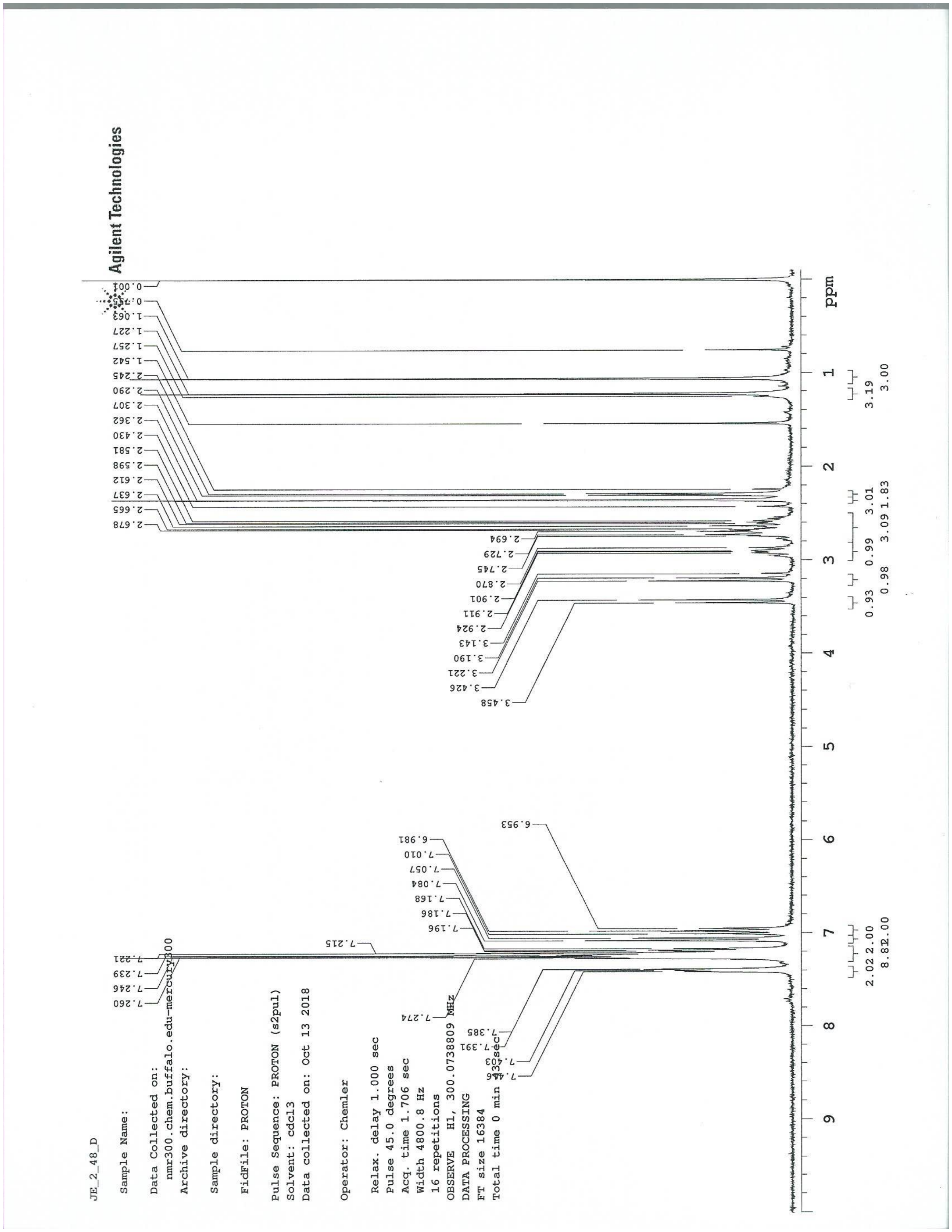
Agilent Technologies
JE_2_48_D
Sample Name:
Data Collected on:
nmr300.chem.buffalo.edu-mercury300
Archive directory:
Sample directory:
FidFile: PROTON
Pulse Sequence: PROTON (s2pul)
Solvent: cdcl3
Data collected on: Oct 13 2018
Operator: Chemler
Relax. delay 1.000 sec
Pulse 45.0 degrees
Acq. time 1.706 sec
Width 4800.8 Hz
16 repetitions
OBSERVE H1, 300.0738809 MHz
DATA PROCESSING
FT size 16384
Total time 0 min 43 sec
7.446
7.403
7.391
7.385
7.274
7.260
7.246
7.239
7.221
7.215
7.196
7.186
7.168
7.084
7.057
7.010
6.981
6.953
3.458
3.426
3.221
3.190
3.143
2.924
2.911
2.901
2.870
2.745
2.729
2.694
2.678
2.665
2.637
2.612
2.598
2.581
2.430
2.362
2.307
2.290
2.245
1.542
1.257
1.227
1.063
0.001
ppm
9
8
7
6
5
4
3
2
1
2.02
2.00
8.82
2.00
0.93
0.98
0.99
3.09
3.01
1.83
3.19
3.00

JE_2_48_D

Sample Name:

Data Collected on:
nmr300.chem.buffalo.edu-mercury300
Archive directory:

Sample directory:

FidFile: CARBON

Pulse Sequence: CARBON (s2pul)
Solvent: cdcl3
Data collected on: Oct 13 2018

Operator: Chemler

Relax. delay 2.000 sec
Pulse 45.0 degrees
Acq. time 0.868 sec
Width 18867.9 Hz
17232 repetitions
OBSERVE C13, 75.4536377 MHz
DECOUPLE H1, 300.0754430 MHz
Power 37 dB
continuously on
WALTZ-16 modulated
DATA PROCESSING
Line broadening 0.5 Hz
FT size 32768
Total time 79 hr, 55 min

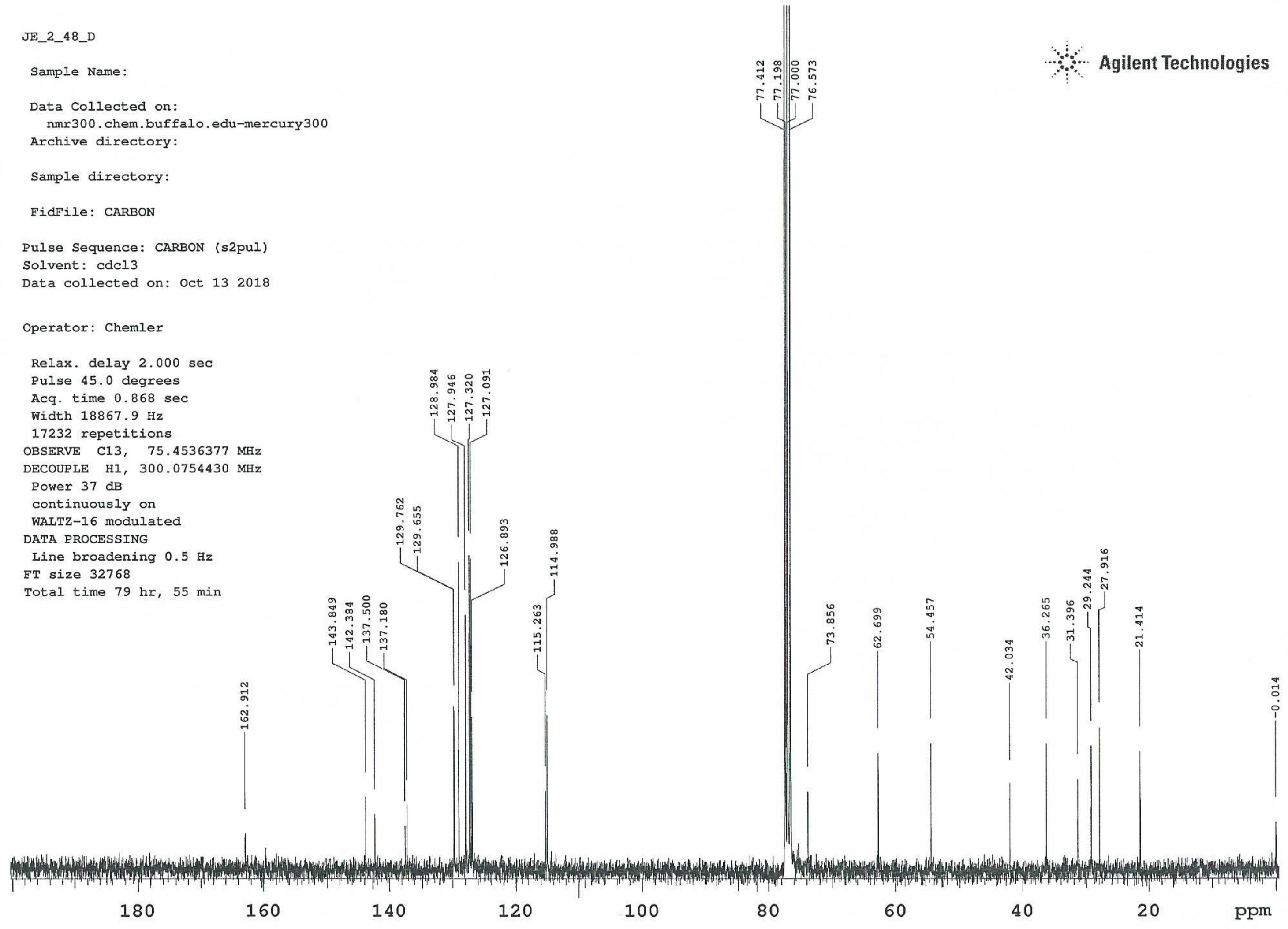

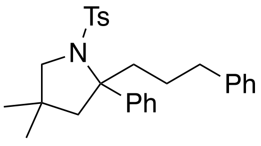

12i

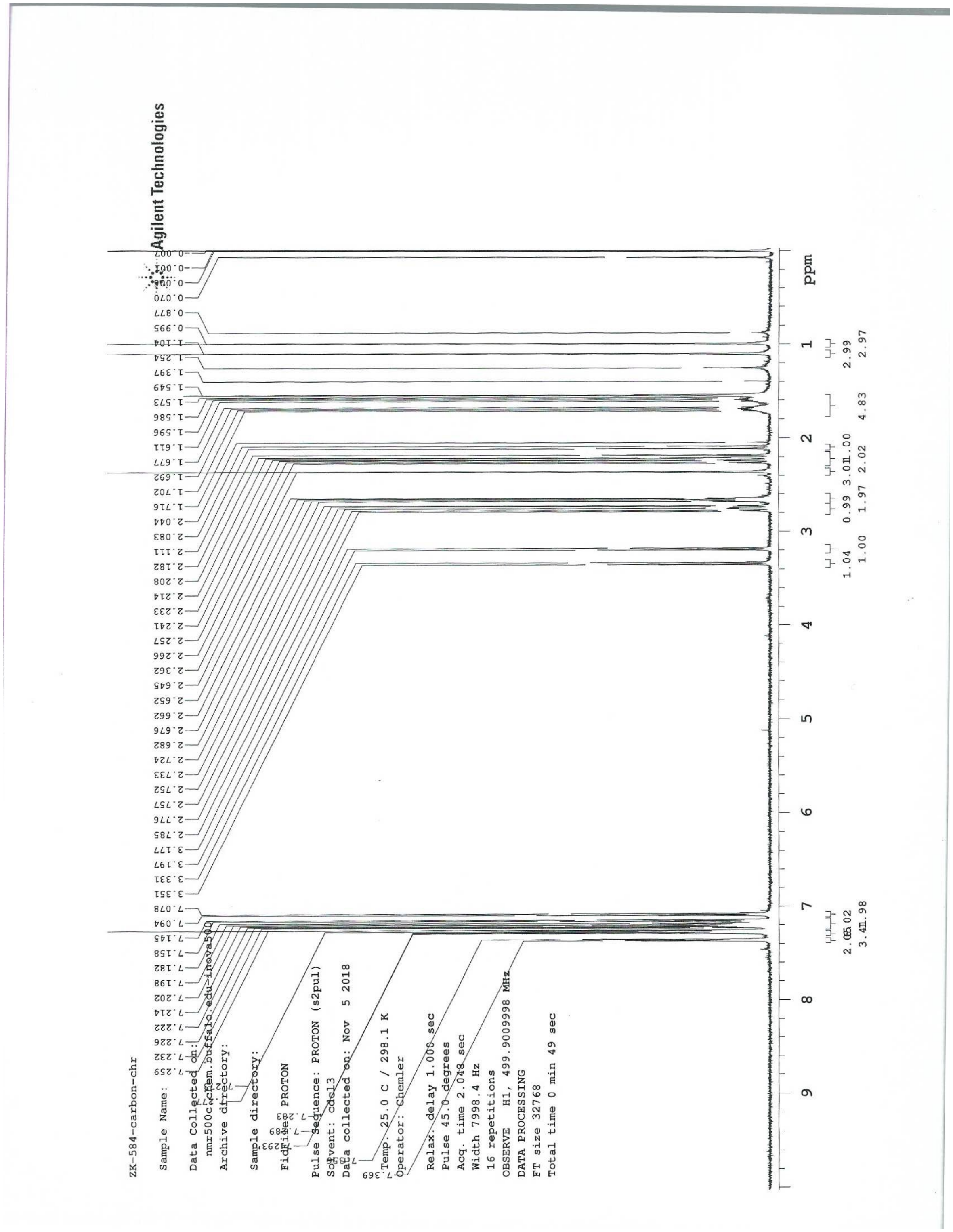

ZK-584-carbon-chr
Sample Name:
Data Collected on:
nmr500c.chem.buffalo.edu-inova500
Archive directory:
Sample directory:
FidFile: PROTON
Pulse Sequence: PROTON (s2pul)
Solvent: cdcl3
Data collected on: Nov 5 2018
Temp. 25.0 C / 298.1 K
Operator: Chemler
Relax. delay 1.000 sec
Pulse 45.0 degrees
Acq. time 2.048 sec
Width 7998.4 Hz
16 repetitions
OBSERVE H1, 499.9009998 MHz
DATA PROCESSING
FT size 32768
Total time 0 min 49 sec
Agilent Technologies
ppm

ZK-584-carbon-chr

Sample Name:

Data Collected on:
nmr500c.chem.buffalo.edu-inova500
Archive directory:

Sample directory:

FidFile: CARBON

Pulse Sequence: CARBON (s2pul)
Solvent: cdcl3
Data collected on: Nov 6 2018

Temp. 25.0 C / 298.1 K
Operator: Chemler

Relax. delay 2.000 sec
Pulse 45.0 degrees
Acq. time 1.043 sec
Width 31421.8 Hz
2880 repetitions
OBSERVE C13, 125.7002064 MHz
DECOUPLE H1, 499.9034960 MHz
Power 40 dB
continuously on
WALTZ-16 modulated
DATA PROCESSING
Line broadening 0.5 Hz
FT size 65536
Total time 8477 hr, 7 min

Agilent Technologies

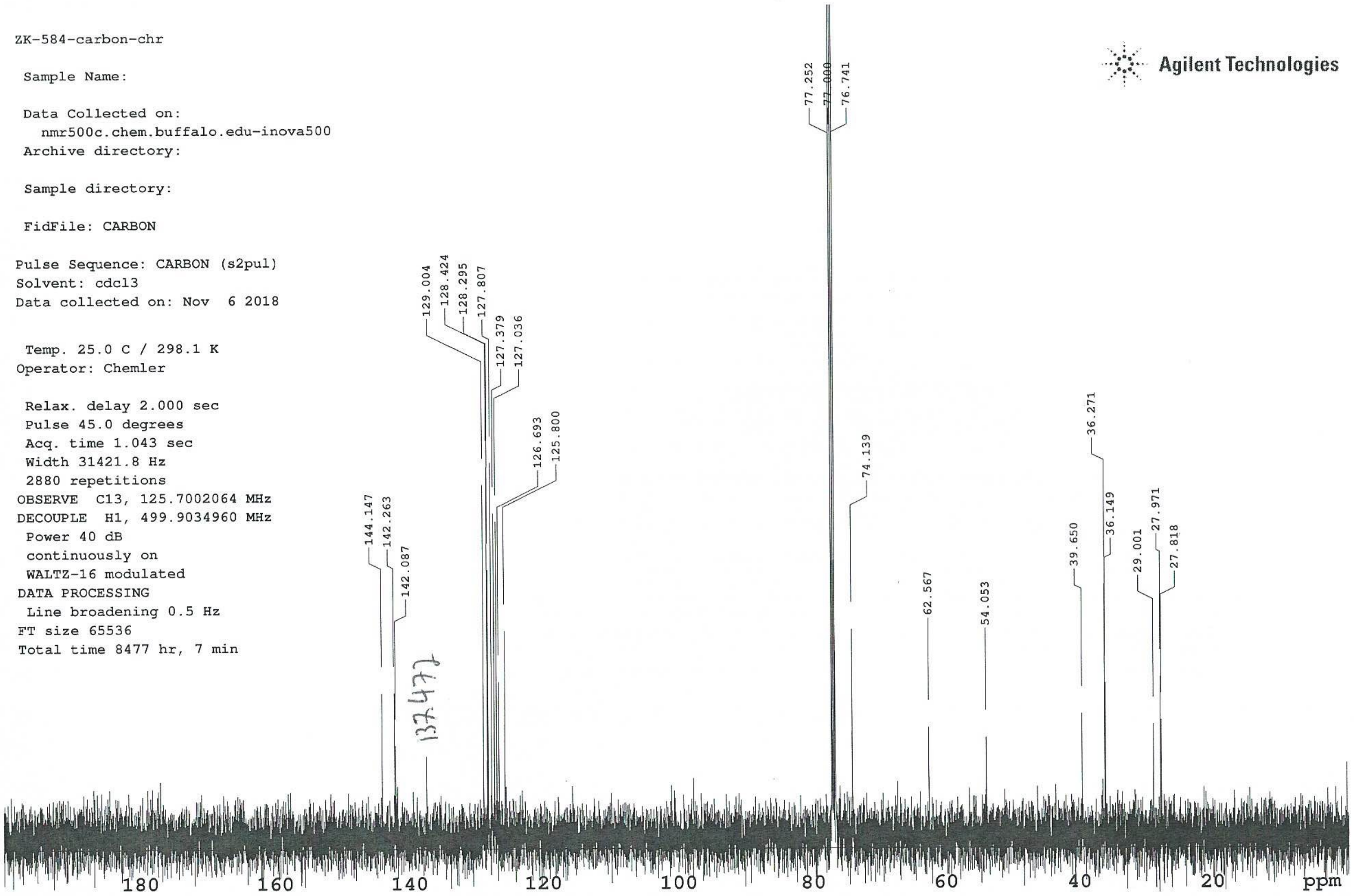

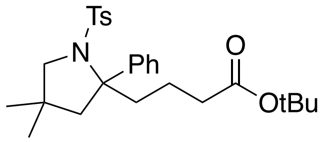
Ts
N
Ph
O
OtBu

12j

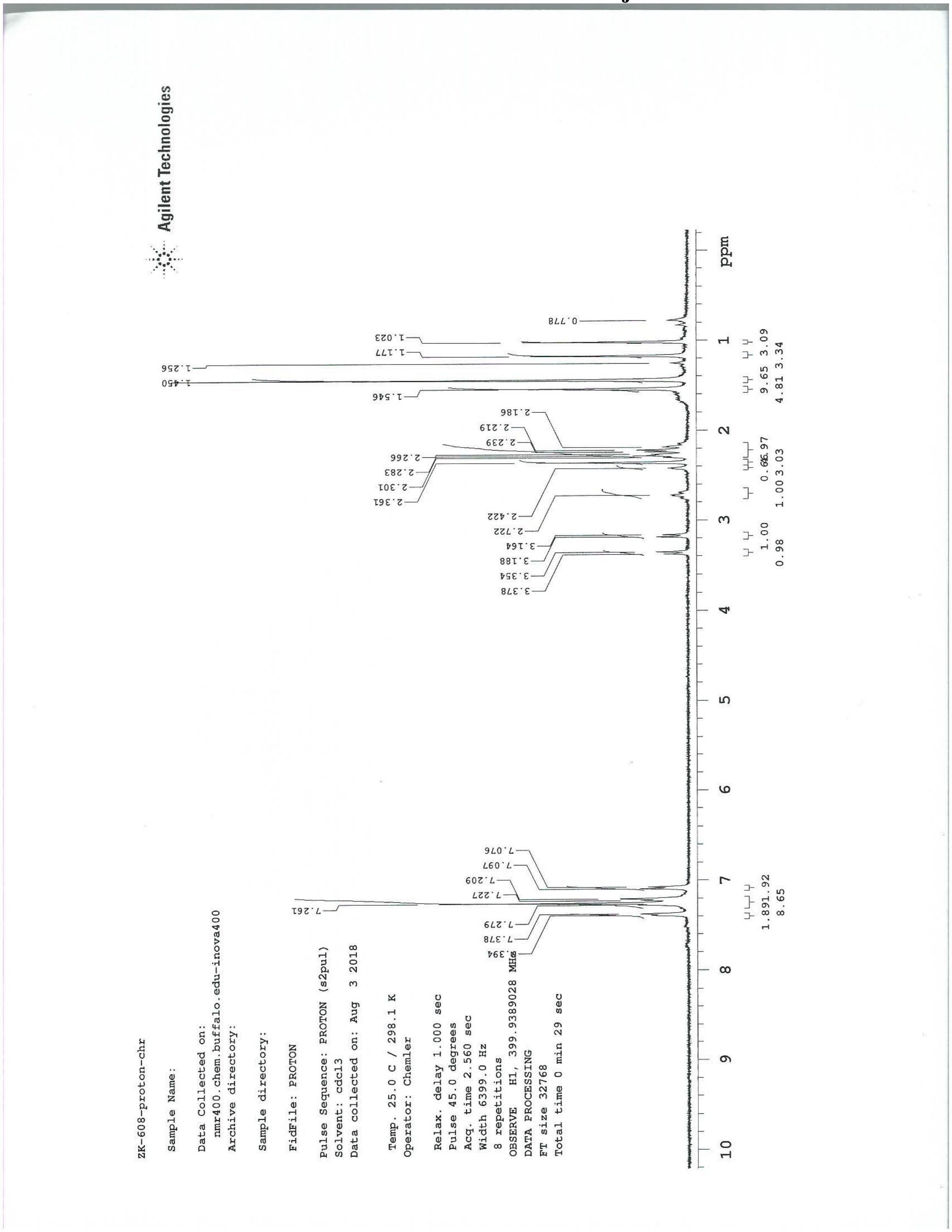
Agilent Technologies
ZK-608-proton-chr
Sample Name:
Data Collected on:
nmr400.chem.buffalo.edu-inova400
Archive directory:
Sample directory:
FidFile: PROTON
Pulse Sequence: PROTON (s2pul)
Solvent: cdcl3
Data collected on: Aug 3 2018
Temp. 25.0 C / 298.1 K
Operator: Chemler
Relax. delay 1.000 sec
Pulse 45.0 degrees
Acq. time 2.560 sec
Width 6399.0 Hz
8 repetitions
OBSERVE H1, 399.9389028 MHz
DATA PROCESSING
FT size 32768
Total time 0 min 29 sec
ppm

ZK-608+612-Proton-be4-Carbon

Sample Name:

Data Collected on:
nmr300.chem.buffalo.edu-mercury300
Archive directory:

Sample directory:

FidFile: ZK-608+612-Proton-be4-Carbon-2

Pulse Sequence: CARBON (s2pul)
Solvent: cdcl3
Data collected on: Aug 3 2018

Operator: Chemler

Relax. delay 3.000 sec
Pulse 45.0 degrees
Acq. time 0.868 sec
Width 18867.9 Hz
10944 repetitions
OBSERVE C13, 75.4536375 MHz
DECOUPLE H1, 300.0754430 MHz
Power 37 dB
continuously on
WALTZ-16 modulated
DATA PROCESSING
Line broadening 0.5 Hz
FT size 32768
Total time 1329 hr, 38 min

Agilent Technologies

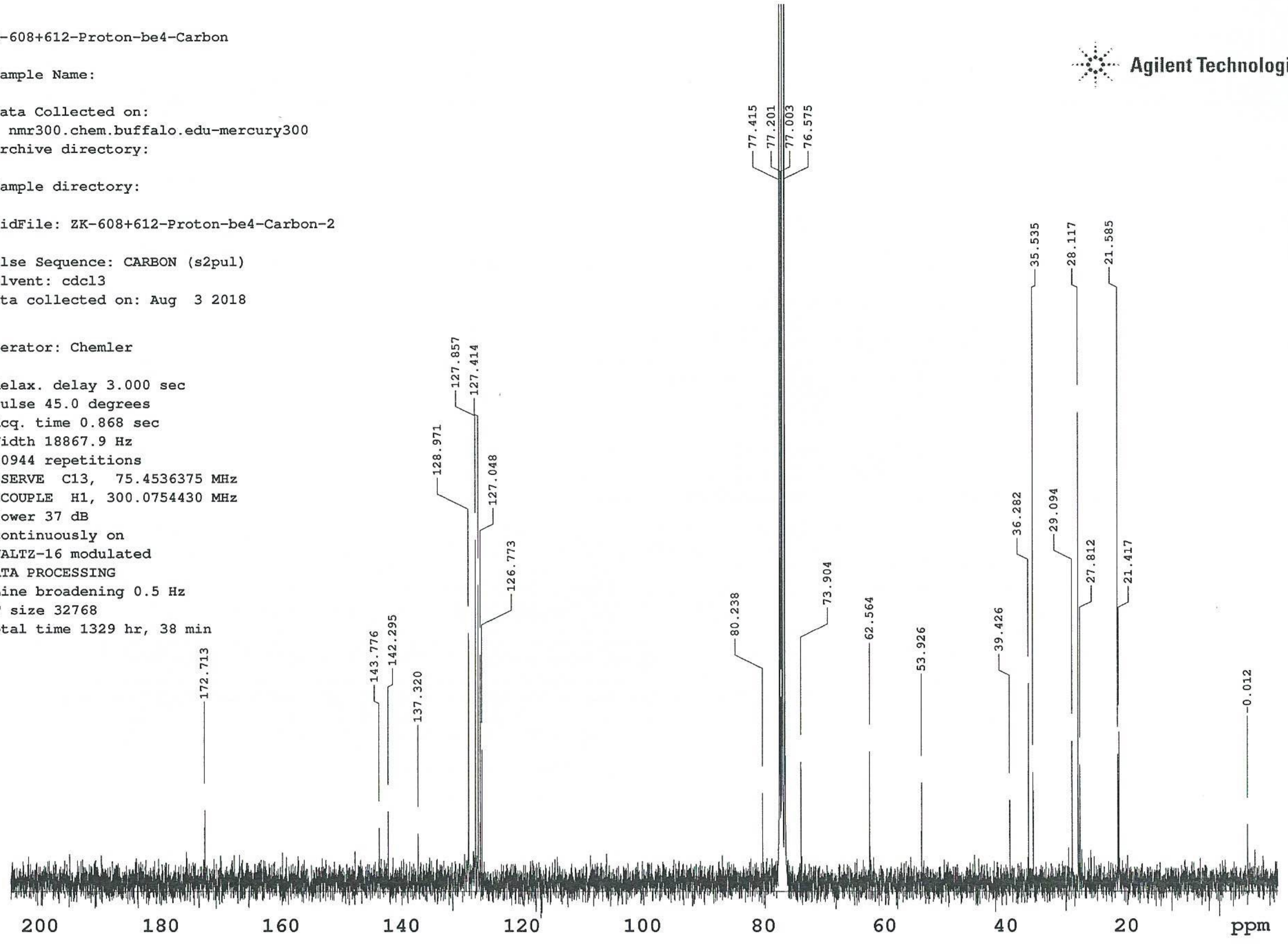

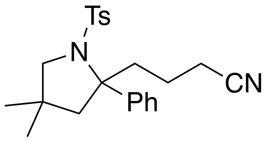
Ts
N
CN
Ph

12k

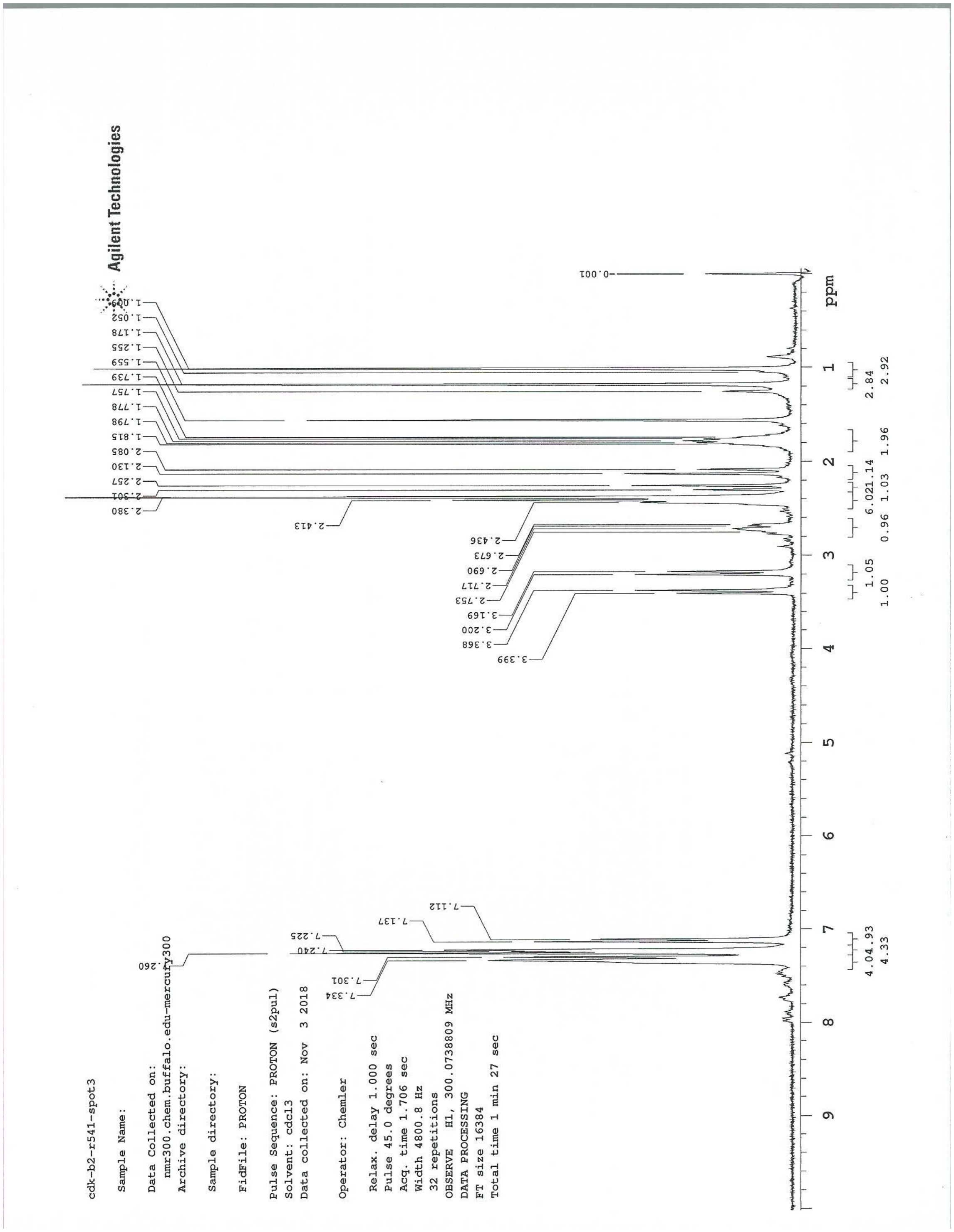
Agilent Technologies
cdk-b2-r541-spot3
Sample Name:
Data Collected on:
nmr300.chem.buffalo.edu-mercury300
Archive directory:
Sample directory:
FidFile: PROTON
Pulse Sequence: PROTON (s2pul)
Solvent: cdcl3
Data collected on: Nov 3 2018
Operator: Chemler
Relax. delay 1.000 sec
Pulse 45.0 degrees
Acq. time 1.706 sec
Width 4800.8 Hz
32 repetitions
OBSERVE H1, 300.0738809 MHz
DATA PROCESSING
FT size 16384
Total time 1 min 27 sec
ppm

cdk-b2-r541-spot3

Sample Name:

Data Collected on:
nmr300.chem.buffalo.edu-mercury300
Archive directory:

Sample directory:

FidFile: CARBON

Pulse Sequence: CARBON (s2pul)
Solvent: cdcl3
Data collected on: Nov 3 2018

Operator: Chemler

Relax. delay 2.000 sec
Pulse 45.0 degrees
Acq. time 0.868 sec
Width 18867.9 Hz
18808 repetitions
OBSERVE C13, 75.4536377 MHz
DECOUPLE H1, 300.0754430 MHz
Power 37 dB
continuously on
WALTZ-16 modulated
DATA PROCESSING
Line broadening 0.5 Hz
FT size 32768
Total time 79 hr, 55 min

Agilent Technologies

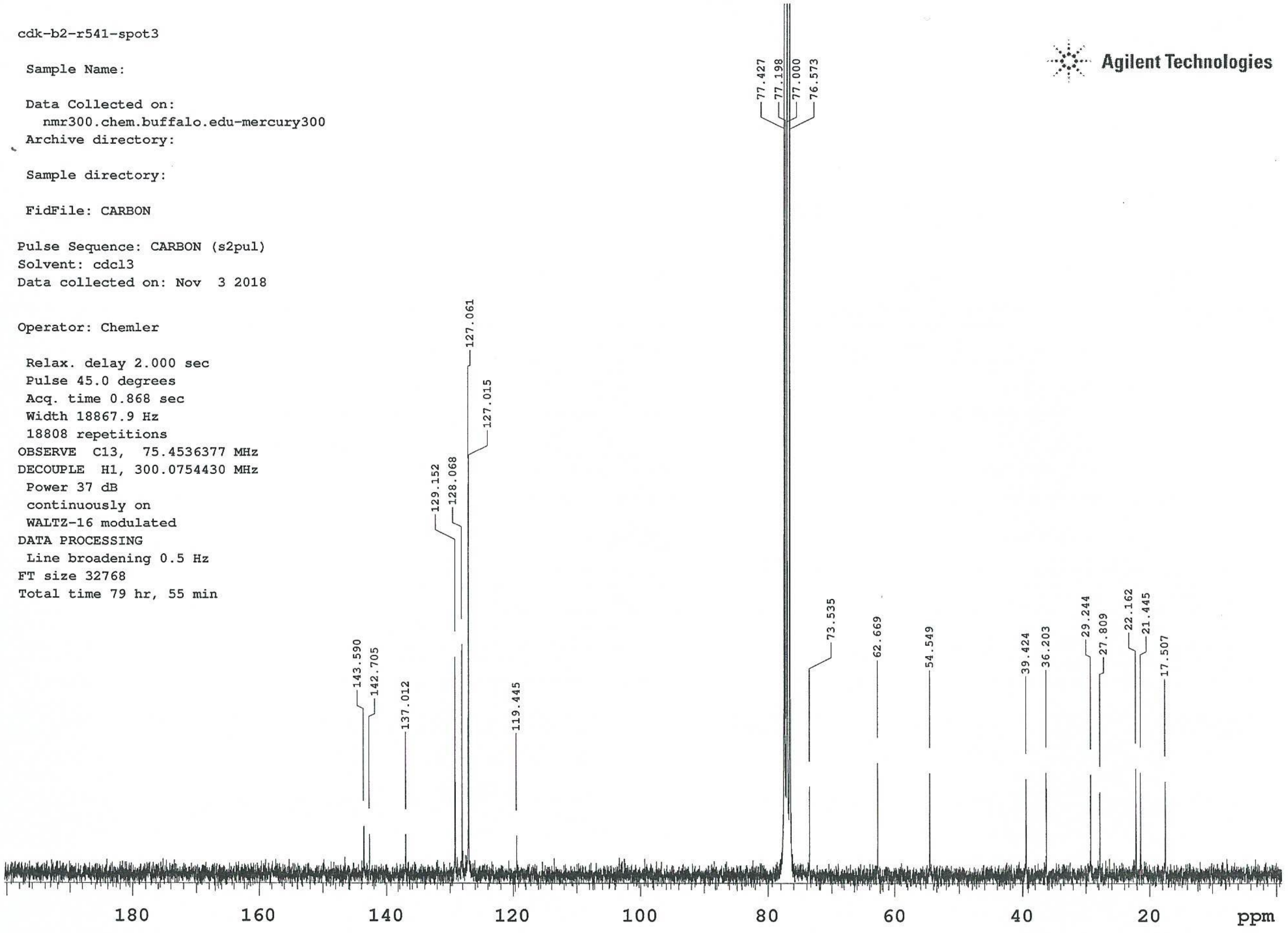

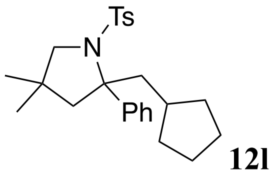

12l

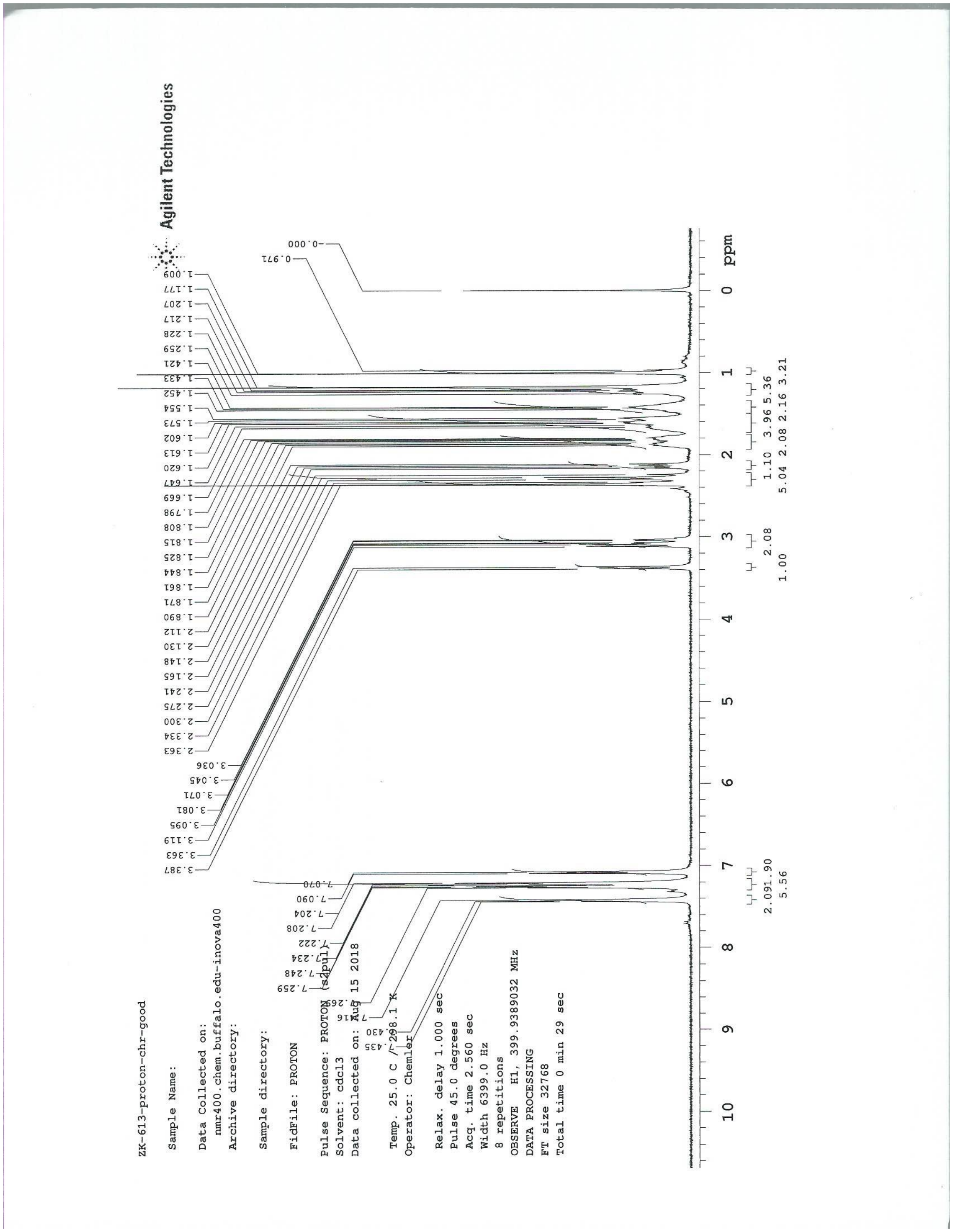
Agilent Technologies
ZK-613-proton-chr-good
Sample Name:
Data Collected on:
nmr400.chem.buffalo.edu-inova400
Archive directory:
Sample directory:
FidFile: PROTON
Pulse Sequence: PROTON (s2pul)
Solvent: cdcl3
Data collected on: Aug 15 2018
Temp. 25.0 C / 298.1 K
Operator: Chemler
Relax. delay 1.000 sec
Pulse 45.0 degrees
Acq. time 2.560 sec
Width 6399.0 Hz
8 repetitions
OBSERVE H1, 399.9389032 MHz
DATA PROCESSING
FT size 32768
Total time 0 min 29 sec
ppm

ZK-613-proton-chr-good

Sample Name:

Data Collected on:
nmr400.chem.buffalo.edu-inova400
Archive directory:

Sample directory:

FidFile: CARBON

Pulse Sequence: CARBON (s2pul)
Solvent: cdcl3
Data collected on: Aug 15 2018

Temp. 25.0 C / 298.1 K
Operator: Chemler

Relax. delay 1.000 sec
Pulse 45.0 degrees
Acq. time 1.303 sec
Width 25141.4 Hz
17024 repetitions
OBSERVE C13, 100.5647169 MHz
DECOUPLE H1, 399.9409068 MHz
Power 33 dB
continuously on
WALTZ-16 modulated
DATA PROCESSING
Line broadening 0.5 Hz
FT size 65536
Total time 792 hr, 56 min

Agilent Technologies

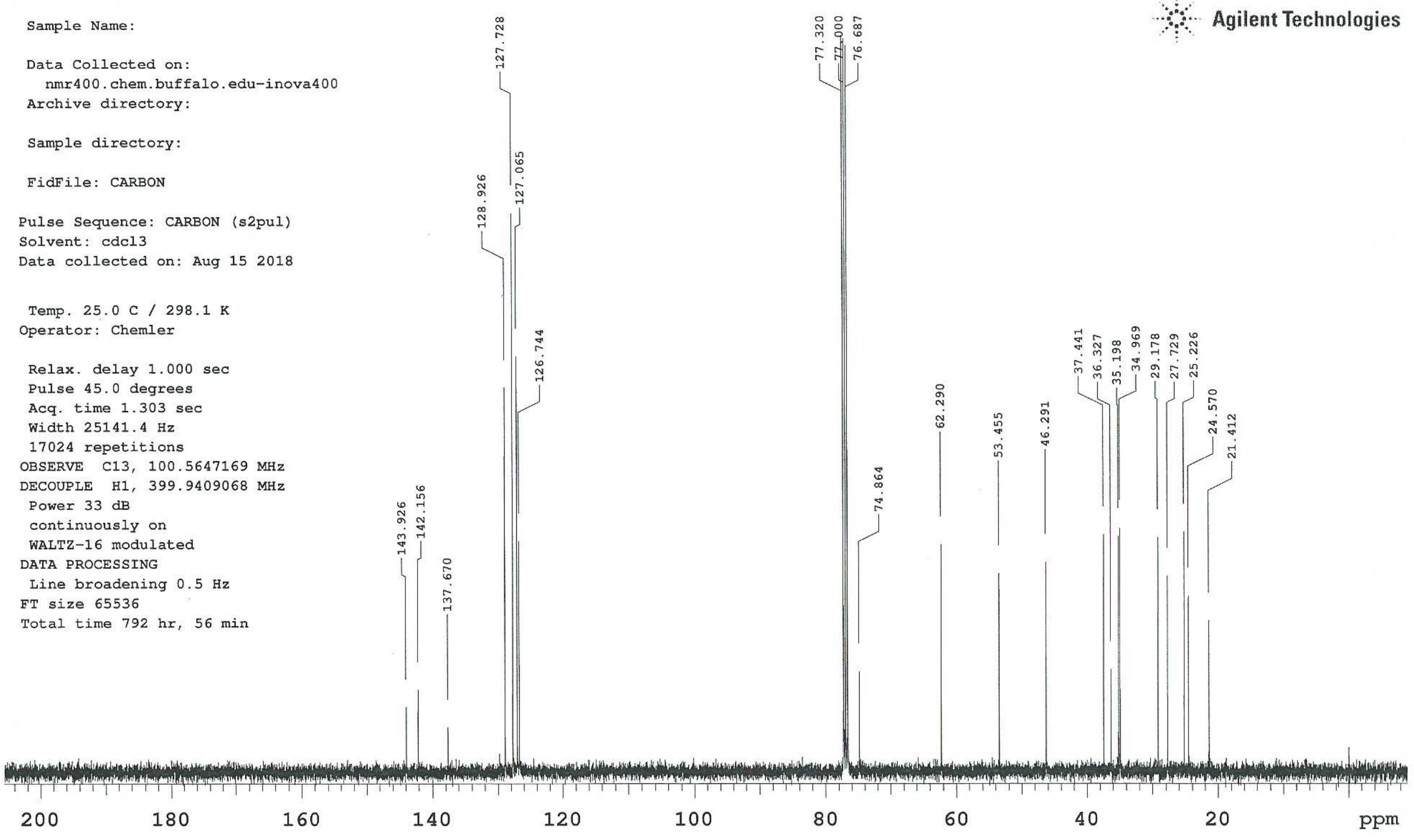

Supplement: Supplementary file 1 [file SC-010-C9SC02835H-s001.pdf]
