# Supplementary material for: Saturated oxygen and nitrogen heterocycles via oxidative coupling of alkyltrifluoroborates with alkenols, alkenoic acids and protected alkenylamines
Source: Chem Sci. 2019 Aug 19;10(40):9265–9. doi: 10.1039/c9sc02835h (PMC7003886; doi:10.1039/c9sc02835h)
Supplement: Supplementary file 2 [file SC-010-C9SC02835H-s002.pdf]

Electronic Supplementary Material (ESI) for Chemical Science.
This journal is © The Royal Society of Chemistry 2019

Saturated Oxygen and Nitrogen Heterocycles via Oxidative Coupling of Alkyltrifluoroborates with Alkenols, Alkenoic Acids, and Alkenylamines

Jonathan M. Shikora, Chanchamnan Um, Zainab M. Khoder, Sherry R. Chemler*

Department of Chemistry, Natural Sciences Complex,
State University of New York at Buffalo, Buffalo, NY 14260

schemler@buffalo.edu

Supporting Information: Experimental Procedures and Characterization of New Compounds

Table of Contents

General Experimental Information……………………………………………………………..S-2
Synthesis of Carboxylic Acids and Alcohols……………………………………………….S-3
Synthesis of Alkenyl Amines……………………………………………………………..…S-16
General Procedure for Synthesis of Lactone and Cyclic Ether Products **2 – 11**………………S-22
Characterization of Novel Lactone and Cyclic Ether Products **2 – 11**.................................S-22
General Procedure for Synthesis of Cyclic Amine Products **12 – 15**....................................S-43
Characterization of Novel Cyclic Amine Products **12 – 15**…………………………………...S-44
Procedure for the Enantioselective Carbolactonization……………………………………..S-51
GC Traces………………………………………………………………………………….....S-52
MnO_2 - Only Oxidative Cyclization Representative Procedure………………………………S-53
References…………………………………………………………………………………..S-55

General Experimental Information:

All reagents were used out of the bottle as purchased from the supplier without further purification unless otherwise noted. All reactions were carried out under an argon environment unless otherwise noted. 4,5-Dihydro-2-(2-(4,5-dihydrooxazol-2-yl)propan2-yl)oxazole (**4a**), (achiral bisoxazoline) was synthesized using our previously reported procedure.[1] 1,2-Dichloroethane was distilled over CaH_2 prior to use. α,α,α-Trifluorotoluene was distilled over P_2O_5 prior to use. ^{1}H NMR spectra were recorded in $CDCl_3$ (using 7.26 ppm for reference of $CHCl_3$) at 300, 400 or 500 MHz unless otherwise noted. ^{13}C NMR spectra were recorded in $CDCl_3$ (using 77.0 ppm as internal reference) at 75, 100, or 125 MHz unless otherwise noted. Coupling constants (*J*) are in Hertz. Abbreviations used are s = singlet, d = doublet, t = triplet, m = multiplet, q = quartet, ABq = AB quartet, quint = quintet, and bs = broad singlet. IR spectra were taken neat using a Nicolet-Impact 420 FTIR. Wave numbers in cm^{-1} are reported for characteristic peaks. High-resolution mass spectra were obtained at SUNY Buffalo's mass spectrometry facility on a ThermoFinnigan MAT XL spectrometer. Flash column chromatography was carried out using 230 x 400 mesh silica gel under increased pressure. Melting points were obtained on an electro thermal melting point apparatus and are reported uncorrected. Optical rotations were obtained using a Rudolph Autopol I Polarimeter fitted with a micro cell with a 1 dm path length. Enantiomeric excess was determined by gas chromatography (GC) using a CP-Chirasil-Dex CB column. Bis(trifluoromethylsulfonyloxy)copper and 1,10-phenanthroline, were purchased from Acros. Potassium alkyltrifluoroborates were purchased from Frontier Scientific. Manganese(IV) oxide (MnO_2, ~85%, <5 μm) was purchased from Aldrich and used without further purification.

Synthesis of Carboxylic Acids and Alcohols

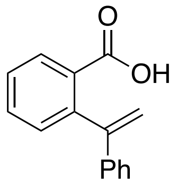

2-(1-Phenylvinyl)benzoic acid (1a)

Carboxylic acid **1a** was synthesized as previously reported.[2] Its ^{1}H NMR spectrum is in agreement with literature data.

^{1}H NMR (400 MHz, $CDCl_3$) δ 8.09 (d, *J* = 7.6 Hz, 1H), 7.92 (d, *J* = 8.0 Hz, 1H), 7.73 (d, *J* = 6.8 Hz, 2H), 7.67 (t, *J* = 7.6 Hz, 1H), 7.60 – 7.18 (m, 5H), 5.68 (s, 1H), 5.23 (s, 1H).

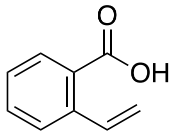

2-Vinylbenzoic acid (1c)

Carboxylic acid **1c** was synthesized as previously reported.[3] Its ^{1}H NMR spectrum is in agreement with literature data.

^{1}H NMR (400 MHz, $CDCl_3$) δ 8.04 (d, 8.0 Hz, 1H), 7.63 – 7.49 (m, 3H), 7.37 (t, *J* = 7.6 Hz, 1H), 5.68 (dd, *J* = 16.4, 0.8 Hz, 1H), 5.39 (dd, *J* = 15.2, 1.2 Hz, 1H).

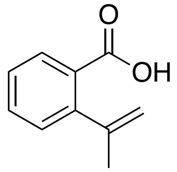

2-(Prop-1-en-2-yl)benzoic acid (1d)

Carboxylic acid **1d** was synthesized as previously reported.[2] Its ^{1}H NMR spectrum is in agreement with literature data.

^{1}H NMR (400 MHz, $CDCl_3$) δ 7.95 (dd, *J* = 7.6, 0.8 Hz, 1H), 7.50 (td, *J* = 7.6, 1.2 Hz, 1H), 7.35 (td, *J* = 7.6, 1.2 Hz, 1H), 7.26 (d, *J* = 7.6 Hz, 1H), 5.14 (s, 1H), 4.90 (s, 1H), 2.12 (s, 3H).

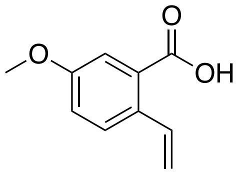

5-Methoxy-2-vinylbenzoic acid (1e)

Carboxylic acid **1e** was synthesized as previously reported.[4] Its ^{1}H NMR spectrum is in agreement with literature data.

^{1}H NMR (300 MHz, $CDCl_3$): δ 10.75 (bs, 1H), 7.52 (m, 3H), 7.10 (dd, *J* = 8.7, 2.5 Hz, 1H), 5.59 (d, *J* = 17.4 Hz, 1H), 5.29 (d, *J* = 11.0 Hz, 1H).

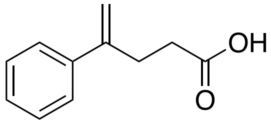

4-Phenylpent-4-enoic acid (1b)

Carboxylic acid **1b** was synthesized as previously reported.[5] Its ^{1}H NMR spectrum is in agreement with literature data.

^{1}H NMR (400 MHz, $CDCl_3$) δ 7.44 – 7.23 (m, 5H), 5.33 (s, 1H), 5.12 (s, 1H), 2.86 (t, *J* = 7.2 Hz, 2H), 2.54 (t, *J* = 7.2 Hz, 2H).

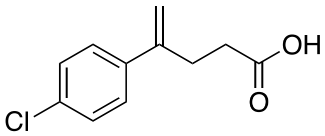

4-(4-Chlorophenyl)pent-4-enoic acid (1h)

Carboxylic acid **1h** was synthesized as previously reported.[5] Its ^{1}H NMR spectrum is in agreement with literature data.

^{1}H NMR (400 MHz, $CDCl_3$) δ 7.35 – 7.28 (m, 4H), 5.31 (s, 1H), 5.12 (s, 1H), 2.82 (t, *J* = 7.6 Hz, 2H), 2.52 (t, *J* = 7.6 Hz, 2H).

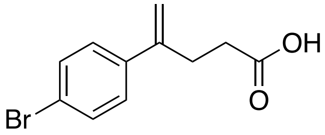

4-(4-Bromophenyl)pent-4-enoic acid (1i)

Carboxylic acid **1i** was synthesized as previously reported.[5] Its ^{1}H NMR spectrum is in agreement with literature data.

^{1}H NMR (400 MHz, $CDCl_3$) δ 7.45 (d, *J* = 8.8 Hz, 2H), 7.27 (d, *J* = 8.4 Hz, 2H), 5.32 (s, 1H), 5.13 (s, 1H), 2.82 (t, *J* = 7.6 Hz, 2H), 2.52 (t, *J* = 7.6 Hz, 2H).

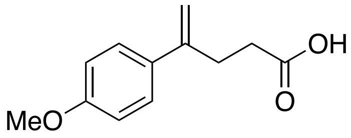

4-(4-Methoxyphenyl)pent-4-enoic acid (1g)

Carboxylic acid **1g** was synthesized as previously reported.[6] Its ^{1}H NMR spectrum is in agreement with literature data.

^{1}H NMR (400 MHz, $CDCl_3$) δ 7.35 (d, *J* = 8.8 Hz, 2H), 6.87 (d, *J* = 8.4 Hz, 2H), 5.26 (s, 1H), 5.03 (s, 1H), 3.82 (s, 3H), 2.83 (t, *J* = 7.6 Hz, 2H), 2.53 (t, *J* = 7.6 Hz, 2H).

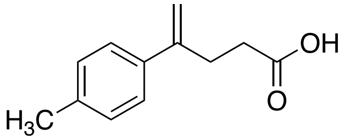

4-(*p*-Tolyl)pent-4-enoic acid (1f)

Carboxylic acid **1f** was synthesized as previously reported.[2] Its ^{1}H NMR spectrum is in agreement with literature data.

^{1}H NMR (400 MHz, $CDCl_3$) δ 7.30 (d, *J* = 8.4 Hz, 2H), 7.15 (d, *J* = 8.4 Hz, 2H), 5.30 (s, 1H), 5.07 (s, 1H), 2.84 (t, *J* = 7.6 Hz, 2H), 2.53 (t, *J* = 8.0 Hz, 2H), 2.35 (s, 3H).

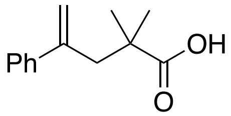

2,2-Dimethyl-4-phenylpent-4-enoic acid (1j)

Carboxylic acid **1j** was synthesized as previously reported.[5] Its ^{1}H NMR spectrum is in agreement with literature data.

^{1}H NMR (400 MHz, $CDCl_3$) δ 7.39 – 7.20 (m, 5H), 5.27 (s, 1H), 5.10 (s, H), 2.82 (s, 2H), 1.11 (s, 6H).

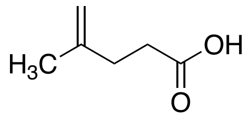

4-Methylpent-4-enoic acid (1k)

Carboxylic acid **1k** was synthesized as previously reported.[7] Its ^{1}H NMR spectrum is in agreement with literature data.

^{1}H NMR (400 MHz, $CDCl_3$) δ 4.77 (s, 1H), 4.71 (s, 1H), 2.52 (t, *J* = 8.0 Hz, 2H), 2.34 (t, *J* = 8.0 Hz, 2H), 1.75 (s, 3H).

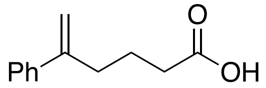

5-Phenylhex-5-enoic acid (1l)

Carboxylic acid **1l** was synthesized as previously reported.[5] Its ^{1}H NMR spectrum is in agreement with literature data.

^{1}H NMR (400 MHz, $CDCl_3$) δ 7.41 – 7.20 (m, 5H), 5.31 (s, 1H), 5.08 (s, 1H), 2.57 (t, *J* = 7.6 Hz, 2H), 2.38 (t, *J* = 7.6 Hz, 2H), 1.79 (quint, *J* = 7.6 Hz, 2H).

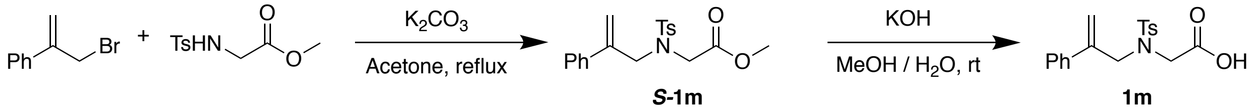

Methyl *N*-(2-phenylallyl)-*N*-tosylglycinate (*S*-1m)

To a suspension of K_2CO_3 (570 mg, 4.1 mmol, 2 equiv.) in acetone (20 mL) was added (3-bromoprop-1-en-2-yl)benzene (410 mg, 2.1 mmol, 1 equiv.) and methyl tosylglycinate (550 mg, 2.3 mmol, 1.1 equiv.). The reaction was refluxed overnight with stirring. The reaction was cooled to room temperature and the solvent removed under vacuum. The remaining residue was diluted with ethyl acetate (30 mL) and washed with water (50 mL). The aqueous phase was extracted with ethyl acetate (2 x 25 mL) and the organic layers were combined, dried over Na_2SO_4, filtered, concentrated, and purified by flash chromatography on silica gel (20% EtOAc / hexanes) to afford methyl 1-(2-phenylallyl)-*N*-tosylglycinate (620 mg, 84% yield) as a yellow oil.

^{1}H NMR (400 MHz, $CDCl_3$) δ 7.65 (d, *J* = 8.3 Hz, 1H), 7.40 - 7.33 (m, 2H), 7.32 - 7.15 (m, 4H), 5.47 (s, 1H), 5.18 (d, *J* = 2.0 Hz, 1H), 4.39 (s, 2H), 3.91 (s, 2H), 3.50 (s, 3H), 2.40 (s, 3H); ^{13}C

NMR (100 MHz, $CDCl_3$) δ 169.2, 143.5, 141.8, 137.7, 129.5, 128.5, 128.2, 127.5, 126.2, 117.2, 51.9, 51.1, 46.4, 21.5; IR (neat): 2953, 1742, 1631, 1598, 1496, 1437, 911, 660 cm^{-1}; HRMS (ESI) for $C_{19}H_{21}NO_4S$: calculated $[M + Na]^+$ *m/z* 382.1083, found 382.1082.

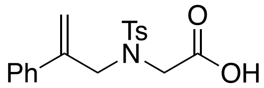

***N*-(2-Phenylallyl)-*N*-tosylglycine (1m)**

To a solution of ***S*-1m** (60 mg, 0.17 mmol) in methanol (1 mL) was added 6 M KOH (1 mL) dropwise. The reaction was allowed to stir at room temperature overnight. A solution of saturated NH_4Cl (50 mL) and the aqueous phase was extracted with CH_2Cl_2 (3 x 25 mL). The organic layers were combined, dried over Na_2SO_4, filtered and concentrated *in vacuo* to give *N*-(2-phenylallyl)-*N*-tosylglycine (57 mg, 97% yield) as a white crystal.

m.p. 91 - 92 ˚C; ^{1}H NMR (400 MHz, $CDCl_3$) δ 7.66 (d, *J* = 8.0 Hz, 2H), 7.39 - 7.22 (m, 7H), 5.50 (s, 1H), 5.21 (s, 1H), 4.40 (s, 2H), 3.94 (s, 2H), 2.42 (s, 3H); ^{13}C NMR (100 MHz, $CDCl_3$) δ 174.1,143.7, 141.8, 137.6, 136.1, 129.5, 128.5, 128.2, 127.5, 126.2, 117.4, 51.2, 46.4, 21.5; IR (neat): 3189 (broad), 2925, 1726, 1631, 1598, 1496, 1445, 1404, 1335, 1306, 1244, 1155, 1092, 1029, 1018, 949, 913, 808, 777, 730, 707, 661, 571, 547 cm^{-1}; HRMS (ESI) for $C_{18}H_{19}NO_4S$: calculated $[M + Na]^+$ *m/z* 346.1108, found 346.1107.

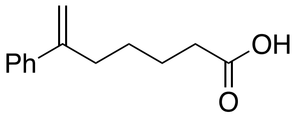

6-Phenylhept-6-enoic acid (1n)

Carboxylic acid **1n** was synthesized as previously reported.[5] Its ^{1}H NMR spectum is in agreement with literature data.

^{1}H NMR (400 MHz, $CDCl_3$) δ 7.42 – 7.21 (m, 5H), 5.27 (s, 1H), 5.06 (s, 1H), 2.53 (t, *J* = 7.6 Hz, 2H), 2.35 (t, *J* = 7.6 Hz, 2H), 1.68 (quint, *J* = 8.0 Hz, 2H), 1.51 (quint, *J* = 7.6 Hz, 2H).

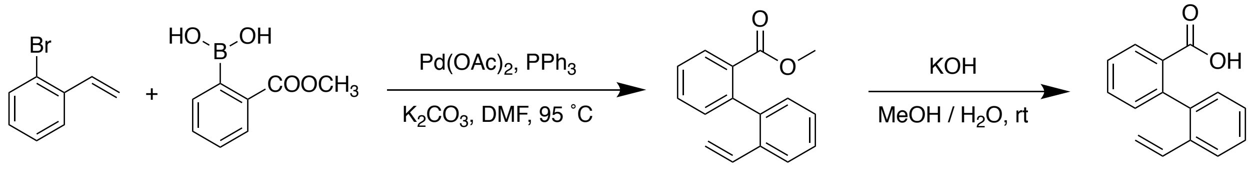

Methyl 2'-vinyl-[1,1'-biphenyl]-2-carboxylate (*S*-1o)

Following a procedure adapted from Wang,[8] 2-bromostyrene (250 mg, 170 µL, 1.4 mmol, 1 equiv.) in a round bottomed flask charged with a magnetic stir bar was treated with (2-(methoxycarbonyl)phenyl)boronic acid (540 mg, 3.0 mmol, 2.2 equiv.), K_2CO_3 (450 mg, 3.3 mmol, 2.4 equiv.), $Pd(PPh_3)_4$ (210 mg, 0.18 mmol, 0.13 equiv.) and DMF (20 mL). The reaction was heated to 95 ˚C overnight with stirring. After cooling to room temperature, the reaction was diluted with diethyl ether (75 mL) and washed with water (50 mL) and brine (50 mL). The organic layer was concentrated *in vacuo* and purified *via* flash chromatography on silica gel (5% EtOAc / hexanes) to afford 200 mg methyl 2'-vinyl-[1,1'-biphenyl]-2-carboxylate (61% yield) as a yellow oil.

^{1}H NMR (400 MHz, $CDCl_3$) δ 7.95 (dd, *J* = 7.2, 1.2 Hz, 1H), 7.62 (d, *J* = 6.8 Hz, 1H), 7.54 (td, *J* = 7.6, 1.2 Hz, 1H), 7.44 (td, *J* = 7.6, 1.2 Hz, 1H), 7.39 - 7.25 (m, 3H), 7.15 (dd, *J* = 7.2, 1.2 Hz, 1H), 6.43 (dd, *J* = 17.6, 10.8 Hz, 1H), 5.63 (dd, *J* = 17.6, 1.2 Hz, 1H), 5.10 (dd, *J* = 10.8, 1.2 Hz, 1H), 3.58 (s, 3H); ^{13}C NMR (100 MHz, $CDCl_3$) δ 167.8, 141.8, 140.4, 135.7, 135.1, 131.5, 131.4, 131.4, 131.0, 129.9, 129.1, 127.5, 127.3, 127.2, 124.7, 114.8, 51.9; IR (neat): 3473, 1717, 1628, 1431, 1289, 1250, 1190, 1126, 1083, 1045, 966, 911, 750, 712 cm^{-1}; HRMS (ESI) for $C_{16}H_{14}O_2$: calculated $[M + Na]^+$ *m/z* 261.0886, found 261.0884.

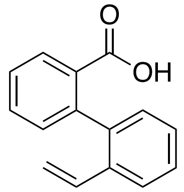

2'-Vinyl-[1,1'-biphenyl]-2-carboxylic acid (1o)

To a solution of ***S*-1o** (75 mg, 0.32 mmol) in methanol (1 mL) was added 6 M KOH (1 mL) dropwise. The reaction was allowed to stir at room temperature overnight. A solution of saturated NH_4Cl (50 mL) and the aqueous phase was extracted with CH_2Cl_2 (3 x 25 mL). The organic layers

were combined, dried over Na_2SO_4, filtered and concentrated *in vacuo* to give 2'-vinyl-[1,1'-biphenyl]-2-carboxylic acid (66 mg, 93% yield) as a white crystal.

m.p. 98 - 99 °C; ^{1}H NMR (400 MHz, $CDCl_3$) δ 8.05 - 7.98 (m, 1H), 7.62 - 7.53 (m, 2H), 7.44 (td, *J* = 7.6, 1.2 Hz, 1H), 7.37 - 7.23 (m, 3H), 7.13 (dd, *J* = 7.6, 1.2 Hz, 1H), 6.41 (dd, *J* = 17.6, 11.2 Hz, 1H), 5.59 (dd, *J* = 17.6, 1.2 Hz, 1H), 5.09 (dd, *J* = 7.6, 1.2 Hz, 1H); ^{13}C NMR (100 MHz, $CDCl_3$) δ 172.0, 142.4, 140.0, 135.7, 135.0, 132.2, 131.8, 129.7, 129.1, 127.5, 127.4, 127.2, 124.9, 115.1; IR (neat): 3016 (broad), 1694, 1597, 1474, 1443, 1406, 1292, 1272, 1139, 1087, 1004, 989, 910, 765, 749, 709, 655, 575 cm^{-1}; HRMS (ESI) for $C_{15}H_{12}O_2$: calculated $[M + Na]^+$ *m/z* 247.0730, found 247.0727.

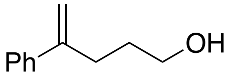

4-Phenylpent-4-en-1-ol (*S*-8a)

Alkenol ***S*-8a** was synthesized as previously reported.[9] Its ^{1}H NMR spectrum is in agreement with literature data.

^{1}H NMR (500 MHz, $CDCl_3$) δ 7.43 (d, *J* = 7.0 Hz, 2H), 7.34 (t, *J* = 6.5 Hz, 2H), 7.28 (t, *J* = 7.0 Hz, 1H), 5.32 (s, 1H), 5.11 (s, 1H), 3.66 (t, *J* = 6.5 Hz, 2H), 2.62 (t, *J* = 7.5 Hz, 2H), 1.73 (q, *J* = 7.0 Hz, 2H), 1.67 (bs, 1H).

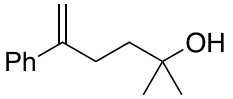

2-Methyl-5-phenylhex-5-en-2-ol (*S*-8b)

Alkenol ***S*-8a** was synthesized as previously reported.[10] Its ^{1}H NMR spectrum is in agreement with literature data.

^{1}H NMR (400 MHz, $CDCl_3$) δ 7.43 – 7.26 (m, 5H), 5.28 (s, 1H), 5.10 (d, *J* = 1.6 Hz, 1H), 2.63 – 2.58 (m, 2H), 1.66 – 1.59 (m, 2H), 1.26 (s, 6H).

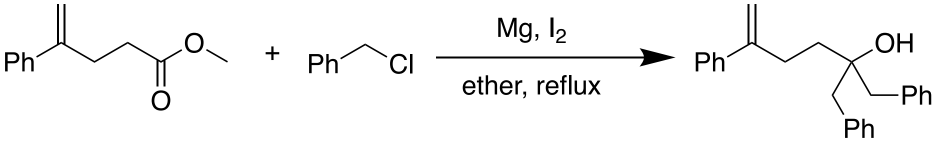

2-Benzyl-1,5-diphenylhex-5-en-2-ol (*S*-8c)

A solution of benzyl magnesium chloride, prepared from benzyl chloride (1.5 mL, 13 mmol, 4 equiv.), magnesium turnings (470 mg, 19 mmol, 6 equiv.), and iodine (1 crystal) in dry THF (10 mL) was added dropwise to a solution of methyl 4-phenylpent-4-enoate (610 mg, 3.2 mmol, 1 equiv.) in dry THF (10 mL) at room temperature. The reaction mixture was heated to reflux overnight and then quenched with a saturated solution of NH_4Cl (50 mL). The aqueous layer was extracted with ether (2 x 25 mL), and the organic layers were combined, dried over Na_2SO_4, filtered, and concentrated *in vacuo*. The crude reaction mixture was subjected to flash chromatography on silica gel (10 % EtOAc / hexanes) to afford 2-benzyl-1,5-diphenylhex-5-en-2-ol (960 mg, 86% yield) as clear oil.

^{1}H NMR (300 MHz, $CDCl_3$) δ 7.40 - 7.26 (m, 10H), 4.73 (s, 1H), 4.70 (s, 1H), 2.87 (s, 4H), 2.30 - 2.20 (m, 2H), 1.73 (s, 3H), 1.58 - 1.50 (m, 2H), 1.46 (s, 1H); ^{13}C NMR (100 MHz, $CDCl_3$) δ 145.7, 137.1, 130.6, 128.1, 126.4, 109.8, 74.1, 45.4, 36.2, 32.0, 22.6; IR (neat): 3568, 3063, 3028, 2922, 1648, 1601, 1494, 1453, 1374, 1274, 1181, 1087, 1031, 953, 885, 792, 748, 728, 700, 634, 542, 524 cm^{-1}; HRMS (ESI) for $C_{20}H_{24}O$: calculated $[M + Na]^+$ *m/z* 303.1719, found 303.1723.

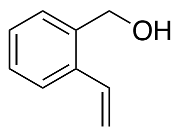

(2-Vinylphenyl)methanol (*S*-9a)

Alkenol ***S*-9a** was synthesized as previously reported.[11] Its ^{1}H NMR spectrum is in agreement with literature data.

^{1}H NMR (500 MHz, $CDCl_3$) δ 7.54 (d, *J* = 7.5 Hz, 1H), 7.39 – 7.25 (m, 3H), 7.04 (dd, *J* = 13.5, 11.0 Hz, 1H), 5.70 (d, *J* = 13.5 Hz, 1H), 5.36 (d, *J* = 10.5 Hz, 1H), 4.72 (s, 2H), 2.17 (bs, 1H).

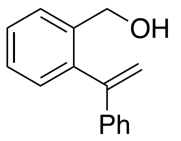

(2-(1-vinyl)phenyl)methanol (*S*-9b)

Alkenol ***S*-9b** was synthesized as previously reported.[11] Its ^{1}H NMR spectrum is in agreement with literature data.

^{1}H NMR (400 MHz, $CDCl_3$) δ 7.52 (d, *J* = 7.6 Hz, 1H), 7.41 – 7.26 (m, 3H), 5.83 (s, 1H), 5.73 (s, 1H), 4.45 (d, 2H), 1.88 (bs, 1H).

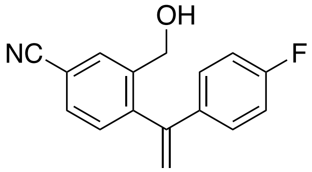

4-(1-(4-Fluorophenyl)vinyl)-3-(hydroxymethyl)benzonitrile (*S*-9c)

Alkenol ***S*-9c** was synthesized as previously reported.[12] Its ^{1}H NMR spectrum is in agreement with literature data.

^{1}H NMR (300 MHz, $CDCl_3$) δ 7.88 (s, 1H), 7.61 (dd, J = 7.8, 1.5 Hz, 1H), 7.33 (1H, d, J = 7.8 Hz, 1H), 7.20 – 7.14 (m, 2H), 6.99 – 6.92 (2H, m), 5.78 (s, 1H), 5.20 (s, 1H), 4.40 (d, J = 5.7 Hz, 2H), 2.85 (bs, 1H).

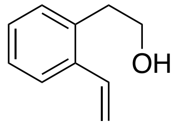

2-(2-Vinylphenyl)ethan-1-ol (*S*-10a)

Alkenol ***S*-10a** was synthesized as previously reported.[13] Its ^{1}H NMR spectrum is in agreement with literature data.

^{1}H NMR (400 MHz, $CDCl_3$) δ 7.53 – 7.47 (m, 1H), 7.25 – 7.09 (m, 3H), 7.02 (dd, *J* = 17.2, 11.2 Hz, 1H), 5.66 (dd, *J* = 16.8, 0.8 Hz, 1H), 5.32 (dd, *J* = 10.8, 0.8 Hz, 1H), 3.83 (q, *J* = 6.4 Hz, 2H), 2.98 (t, *J* = 6.8 Hz, 2H), 1.56 (bs, 1H).

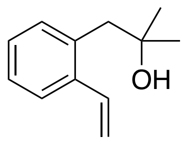

2-Methyl-1-(2-vinylphenyl)propan-2-ol (*S*-10b)

To a solution of methyl 2-(2-vinylphenyl)acetate (110 mg, 0.62 mmol) in dry THF (3 mL) was added a solution of methyl magnesium chloride (3 M in THF, 0.83 mL, 2.5 mmol, 4 equiv.) dropwise at 0 ˚C. The reaction was allowed to warm to room temperature and stir overnight. A saturated solution of NH_4Cl (25 mL) was added to quench the reaction and the aqueous phase was

extracted with diethyl ether (2 x 30 mL). The combined organic layers were dried over Na_2SO_4, filtered, and concentrated *in vacuo*. The crude reaction mixture was subjected to flash chromatography on silica gel (20% EtOAc / hexanes) to afford 2-methyl-1-(2-vinylphenyl)propan-2-ol (82 mg, 75% yield) as a clear oil.

^{1}H NMR (400 MHz, $CDCl_3$) δ 7.56 (m, 1H), 7.29 - 7.19 (m, 3H), 7.12 (dd, *J* = 17.2, 10.8 Hz, 1H), 5.65 (dd, *J* = 17.2, 1.2 Hz, 1H), 5.30 (dd, *J* = 10.8, 1.2 Hz, 1H), 2.90 (s, 2H), 1.46 (s, 1H), 1.24 (s, 6H); ^{13}C NMR (100 MHz, $CDCl_3$) δ 137.9, 135.7, 135.3, 131.8, 127.4, 126.9, 126.0, 115.5, 71.8, 45.3, 29.5; IR (neat): 3394, 3061, 3024, 2971, 2929, 1625, 1483, 1465, 1449, 1412, 1374, 1299, 1206, 1135, 1022, 989, 971, 906, 769, 721, 617, 575, 516 cm^{-1}; HRMS (ESI) for $C_{12}H_{16}O$: calculated $[M + Na]^+$ *m/z* 199.1093, found 199.1089.

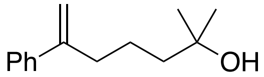

2-Methyl-6-phenylhept-6-en-2-ol (*S*-10c)

To a solution of methyl 5-phenylhex-5-enoate (150 mg, 0.72 mmol) in dry THF (4 mL) was added a solution of methyl magnesium chloride (3 M in THF, 0.96 mL, 2.9 mmol, 4 equiv.) dropwise at 0 °C. The reaction was allowed to warm to room temperature and stir overnight. A saturated solution of NH_4Cl (25 mL) was added to quench the reaction and the aqueous phase was extracted with diethyl ether (2 x 30 mL). The combined organic layers were dried over Na_2SO_4, filtered, and concentrated *in vacuo*. The crude reaction mixture was subjected to flash chromatography on silica gel (20% EtOAc / hexanes) to afford 2-methyl-1-(2-vinylphenyl)propan-2-ol (130 mg, 86% yield) as a clear oil.

^{1}H NMR (300 MHz, $CDCl_3$) δ 7.42 – 7.21 (m, 5H), 5.28 (d, *J* = 1.2 Hz, 1H), 5.07 (d, *J* = 1.8 Hz, 1H), 2.54 – 2.48 (m, 2H), 1.54 – 1.42 (m, 4H), 1.30 (bs, 1H), 1.17 (s, 6H);); ^{13}C NMR (75 MHz, $CDCl_3$) δ 148.4, 141.3, 128.3, 127.3, 126.1, 112.4, 70.9, 43.4, 35.7, 29.2, 22.9; IR (neat): 3367, 3081, 2968, 2941, 1626, 1600, 1574, 1494, 1465, 1443, 1376, 1301, 1199, 1133, 1074, 1027, 940, 894, 777, 703 cm^{-1}; HRMS (ESI) $C_{14}H_{20}O$: calculated $[M + Na]^+$ *m/*z 227.1406, found 227.1405.

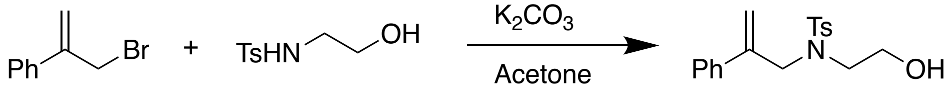

***N*-(2-Hydroxyethyl)-4-methyl-*N*-(2-phenylallyl)benzenesulfonamide (*S*-10d)**

To a stirring solution of *N*-tosyl-ethanolamine (210 mg, 0.97 mmol, 1 equiv.) in dry acetone (4 mL) was added potassium carbonate (400 mg, 2.9 mmol, 3 equiv.) followed by a solution of (3-bromoprop-1-en-2-yl)benzene (200 mg, 1.0 mmol, 1.1 equiv.) in dry acetone (1 mL) under nitrogen atmosphere. The stirring was continued for 8 hours. After completion of the reaction, the mixture was filtered and the residue was washed several times with acetone. The filtrate was concentrated in vacuum and extracted with dichloromethane (40 mL) and water (40 mL). The organic extract was washed with brine solution and concentrated under reduced pressure. The crude product was subjected to column chromatography (40% EtOAc / hexanes) to obtain *N*-(2-hydroxyethyl)-4-methyl-*N*-(2-phenylallyl)benzenesulfonamide (250 mg, 79% yield) as a viscous light yellow oil.

^{1}H NMR (300 MHz, $CDCl_3$) δ 7.68 (d, J = 8.4 Hz, 2H), 7.50 - 7.42 (m, 2H), 7.39 - 7.27 (m, 5H), 5.50 (s, 1H), 5.23 (s, 1H), 4.24 (s, 2H), 3.60 - 3.51 (m, 2H), 3.16 (t, J = 6.0 Hz, 2H), 2.44 (s, 3H), 2.00 (bs, 1H); ^{13}C NMR (75 MHz, $CDCl_3$) δ 143.7, 143.0, 137.8, 135.2, 129.8, 128.6, 128.3, 127.5, 126.5, 116.8, 61.0, 53.7, 50.2, 21.5; IR (neat): 3525, 2923, 1631, 1598, 1575, 1495, 1332, 1306, 1155, 1088, 1020, 914, 848, 814, 780, 759, 737, 709, 659, 614, 571, 549 cm^{-1}; HRMS (ESI) for $C_{18}H_{21}NO_3S$: calculated $[M + Na]^+$ *m/z* 354.1134, found 354.1134.

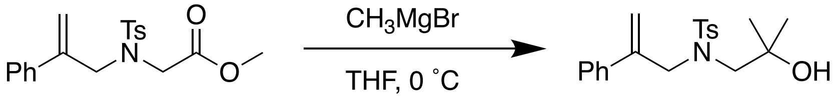

***S*-1m** **S-10e**

N-(2-Hydroxy-2-methylpropyl)-4-methyl-N-(2-phenylallyl)benzenesulfonamide (*S*-10e)

To a solution of ***S*-1m** (400 mg, 1.1 mmol) in dry THF (8 mL) was added a solution of methyl magnesium bromide (3 M in THF, 4.5 mmol, 4 equiv.) dropwise at 0 °C. The reaction was allowed to warm to room temperature and was stirred overnight. A saturated solution of NH_4Cl (25 mL) was added to quench the reaction and the aqueous phase was extracted with diethyl ether (2 x 30 mL). The combined organic layers were dried over Na_2SO_4, filtered, and concentrated *in vacuo*. The crude reaction mixture was subjected to flash chromatography on silica gel (20% EtOAc / hexanes) to afford *N*-(2-hydroxy-2-methylpropyl)-4-methyl-*N*-(2-phenylallyl)benzenesulfonamide (360 mg, 92% yield) as a viscous light yellow oil.

^{1}H NMR (400 MHz, $CDCl_3$) δ 7.68 (d, J = 8.4 Hz, 2H), 7.40 - 7.35 (m, 2H), 7.35 - 7.27 (m, 5H), 5.46 (s, 1H), 5.20 (s, 1H), 4.27 (s, 2H), 3.12 (s, 2H), 3.03 (s, 1H), 2.44 (s, 3H), 1.15 (s, 6H); ^{13}C

NMR (100 MHz, $CDCl_3$) δ 143.7, 143.3, 138.5, 135.1, 129.7, 128.4, 128.1, 127.7, 126.4, 116.5, 71.0, 59.4, 55.1, 27.6, 21.5; IR (neat): 3515, 2973, 2926, 1630, 1598, 1575, 1495, 1444, 1366, 1329, 1306, 1288, 1185, 1152, 1090, 1054, 1018, 973, 912, 872, 814, 804, 776, 753, 708, 658, 583, 550 cm^{-1}; HRMS (ESI) for $C_{20}H_{25}NO_3S$: calculated $[M + H]^+$ *m/z* 360.1628, found 360.1630.

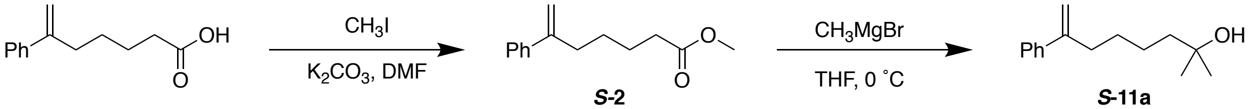

Methyl 6-phenylhept-6-enoate (*S*-2)

6-Phenylhept-6-enoic acid (230 mg, 1.2 mmol, 1 equiv.) was added to a suspension of K_2CO_3 (480 mg, 3.4 mmol, 3 equiv.) in DMF (5 mL) at room temperature. Methyl iodide (110 µL, 1.7 mmol, 1.5 equiv.) was added dropwise, and the solution was allowed to stir at room temperature overnight. The reaction was then diluted with ether (30 mL) and washed with a saturated solution of NH_4Cl (30 mL). The aqueous phase was extracted with ether (2 x 25 mL) and the organic layers were combined, dried over Na_2SO_4, filtered, and concentrated *in vacuo*. The crude reaction mixture was subjected to flash chromatography on silica gel (5 % EtOAc / hexanes) to afford methyl 6-phenylhept-6-enoate (230 mg, 91% yield) as a clear oil.

^{1}H NMR (400 MHz, $CDCl_3$) δ 7.42 - 7.22 (m, 5H), 5.27 (s, 1H), 5.06 (d, *J* = 1.2 Hz, 1H), 3.64 (s, 3H), 2.52 (t, *J* = 7.2 Hz, 2H), 2.30 (t, *J* = 7.6 Hz, 2H), 1.65 (m, 2H), 1.48 (m, 2H); ^{13}C NMR (100 MHz, $CDCl_3$) δ 174.1, 148.1, 141.1, 128.3, 127.3, 126.1, 112.5, 51.4, 34.9, 33.9, 27.6, 24.5; IR (neat): 2945, 2863, 1736, 1626, 1574, 1494, 1435, 1362, 1195, 1172, 1146, 1108, 1060, 1027, 895, 778, 704 cm^{-1}; HRMS (ESI) for $C_{14}H_{18}O_2$: calculated $[M + Na]^+$ *m/z* 241.1199, found 241.1204.

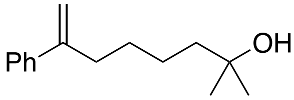

2-Methyl-7-phenyloct-7-en-2-ol (*S*-11a)

To a solution of ***S*-2** (230 mg, 1.1 mmol, 1 equiv.) in dry THF (5 mL) was added a solution of methyl magnesium bromide (3 M in THF, 1.4 mL, 4.2 mmol, 4 equiv.) dropwise at 0 ˚C. The reaction was allowed to warm to room temperature and stir overnight. A saturated solution of NH_4Cl (25 mL) was added to quench the reaction and the aqueous phase was extracted with diethyl ether (2 x 30 mL). The combined organic layers were dried over Na_2SO_4, filtered, and concentrated

in vacuo. The crude reaction mixture was subjected to flash chromatography on silica gel (5% EtOAc / hexanes) to afford 2-methyl-7-phenyloct-7-en-2-ol (190 mg, 83% yield) as a clear oil.

^{1}H NMR (400 MHz, $CDCl_3$) δ 7.42 - 7.38 (m, 2H), 7.36 - 7.22 (m, 3H), 5.25 (d, *J* =1.2 Hz, 1H), 5.05 (d, *J* = 1.6 Hz, 1H), 2.52 (t, *J* = 6.8 Hz, 2H), 1.49 - 1.33 (m, 6H), 1.19 (s, 6H); ^{13}C NMR (100 MHz, $CDCl_3$) δ 148.5, 141.3, 128.2, 127.3, 126.1, 112.2, 71.0, 43.7, 35.3, 29.2, 28.7, 24.0; IR (neat): 3358, 3081, 2969, 2861, 1739, 1626, 1574, 1494, 1466, 1443, 1365, 1192, 1148, 1028, 952, 895, 777, 703, 614 cm^{-1}; HRMS (ESI) for $C_{15}H_{22}O$: calculated $[M + Na]^+$ *m/z* 241.1563, found 241.1566.

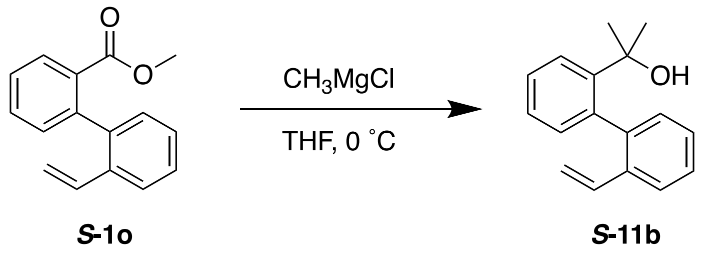

2-(2'-vinyl-[1,1'-biphenyl]-2-yl)propan-2-ol (*S*-11b)

To a solution of ***S*-1o** (170 mg, 0.73 mmol, 1 equiv.) in dry THF (6 mL) was added a solution of methyl magnesium bromide (3 M in THF, 1.0 mL, 2.9 mmol, 4 equiv.) dropwise at 0 ˚C. The reaction was allowed to warm to room temperature and was stirred overnight. A saturated solution of NH_4Cl (25 mL) was added to quench the reaction and the aqueous phase was extracted with diethyl ether (2 x 30 mL). The combined organic layers were dried over Na_2SO_4, filtered, and concentrated *in vacuo*. The crude reaction mixture was subjected to flash chromatography on silica gel (10% EtOAc / hexanes) to afford 2-(2'-vinyl-[1,1'-biphenyl]-2-yl)propan-2-ol (120 mg, 70% yield) as a white solid.

m.p. 56 - 58 ˚C; ^{1}H NMR (400 MHz, $CDCl_3$) δ 7.66 (d, *J* = 7.6 Hz, 2H), 7.39 - 7.32 (m, 2H), 7.30 - 7.19 (m, 3H), 6.99 (dd, *J* = 7.6, 1.2 Hz, 1H), 6.39 (dd, *J* = 17.6, 10.8 Hz, 1H), 5.65 (dd, *J* = 17.6, 1.2 Hz, 1H), 5.10 (dd, *J* = 10.8, 1.2 Hz, 1H), 1.70 (s, 1H), 1.46 (s, 3H), 1.39 (s, 3H); ^{13}C NMR (100 MHz, $CDCl_3$) δ 146.5, 142.1, 137.9, 136.1, 135.5, 132.1, 130.2, 127.6, 127.5, 126.9, 126.2, 126.2, 124.7, 114.5, 74.0, 32.3, 32.0; IR (neat): 3396, 3059, 2971, 2928, 1627, 1556, 1471, 1435, 1413, 1363, 1264, 1231, 1163, 1106, 1076, 1050, 1022, 995, 949, 911, 857, 782, 757, 623, 556 cm^{-1}; HRMS (ESI) for $C_{17}H_{18}O$: calculated $[M + Na]^+$ *m/z* 261.1250, found 261.1248.

Synthesis of Alkenyl Amines

General Procedure for Synthesis of the Substrates *S*-12a to *S*-12l

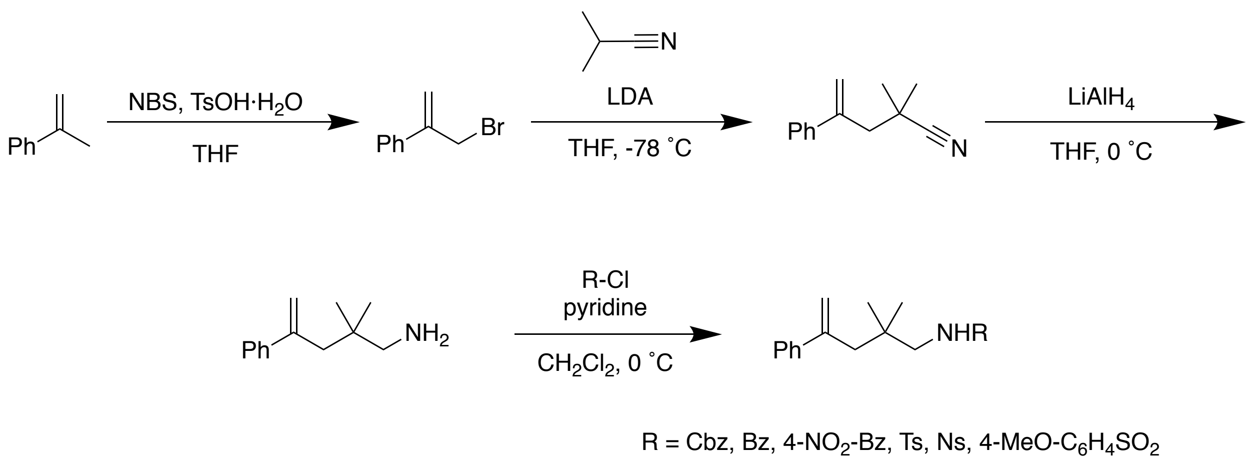

Compounds ***S*-12a to *S*-12l** were prepared from the 2,2-dimethyl-4-phenylpent-4-en-1-amine (previously reported)[14] as follows:

To a solution of 2,2-dimethyl-4-phenylpent-4-en-1-amine (1 equiv.) in anhydrous CH_2Cl_2 (0.1 M) at 0 ˚C, triethylamine (1.1 equiv.) was added followed by the required R-Cl reagent (1.1 equiv.). The solution was allowed to warm to room temperature and stirred for 16 h, quenched with water and extracted with CH_2Cl_2 (3 x 10 mL). The combined organic layers were dried over Na_2SO_4 then concentrated *in vacuo*. The crude products were isolated by flash column chromatography on silica gel using EtOAc / hexanes.

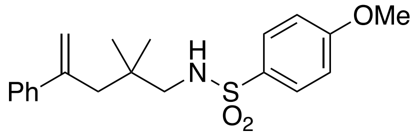

***N*-(2,2-Dimethyl-4-phenylpent-4-en-1-yl)-4-methoxybenzenesulfonamide (*S*-12a)**

2,2-Dimethyl-4-phenylpent-4-en-1-amine (76 mg, 0.40 mmol) was converted to ***S*-12a** as above (67 mg, 47% yield, clear oil).

^{1}H NMR (400 MHz, $CDCl_3$) δ 7.58 (d, *J* = 8.9 Hz, 2H), 7.41 - 7.18 (m, 5H), 6.91 (d, *J* = 8.9 Hz, 2H), 5.21 (s, 1H), 5.02 (s, 1H), 4.28 (t, *J* = 7.0 Hz, 1H), 3.86 (s, 3H), 2.53 - 2.38 (m, 4H), 0.79 (s, 6H); ^{13}C NMR (75 MHz, $CDCl_3$) δ 162.8, 146.0, 143.2, 131.4, 129.0, 128.4, 127.3, 126.3, 117.5, 55.5, 52.8, 44.8, 36.1, 25.5; IR (neat): 3281, 2951, 1578, 1596, 1302, 1258, 1153, 832, 560 cm^{-1}; HRMS (ESI) for $C_{20}H_{25}NO_3S$: calculated $[M + Na]^+$ *m/z* 382.1447, found 382.1456.

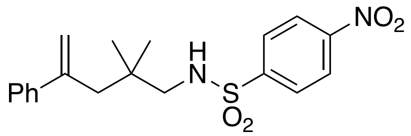

***N*-(2,2-Dimethyl-4-phenylpent-4-en-1-yl)-4-nitrobenzenesulfonamide (*S*-12b)**

2,2-Dimethyl-4-phenylpent-4-en-1-amine (300 mg, 1.6 mmol) was converted to ***S*-12b** as above (422 mg, 71% yield, yellow crystal).

m.p. 82 - 84 ˚C; ^{1}H NMR (400 MHz, $CDCl_3$) δ 8.28 (d, *J* = 8.8 Hz, 2H), 7.72 (d, *J* = 9.2 Hz, 2H), 7.35 - 7.28 (m, 5H), 5.24 (d, *J* = 1.2 Hz, 1H), 5.05 (s, 1H), 4.20 - 4.15 (m, 1H), 2.50 (d, *J* = 6.8 Hz, 2H), 2.46 (s, 2H), 0.86 (s, 6H); ^{13}C NMR (100 MHz, $CDCl_3$) δ 149.8, 145.6, 145.6, 143.2, 128.7, 128.1, 127.6, 126.3, 124.2, 118.0, 112.2, 52.7, 45.0, 35.3, 25.6; IR (neat): 3302, 1529, 1348, 1311, 1165, 1092, 1065, 907, 853, 781, 735, 685, 609, 558 cm^{-1}; HRMS (ESI) for $C_{19}H_{22}N_2O_4S$: calculated $[M + Na]^+$ *m/z* 397.1192, found 397.1192.

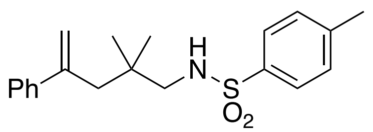

***N*-(2,2-Dimethyl-4-phenylpent-4-en-1-yl)-4-methylbenzenesulfonamide (*S*-12c)**

2,2-Dimethyl-4-phenylpent-4-en-1-amine (200 mg) was converted to **(*S*-12c)** as above (180 mg, 50% yield, white crystals).

m.p. 59 - 61 ˚C; ^{1}H NMR (400 MHz, $CDCl_3$) δ 8.28 (d, *J* = 8.8 Hz, 2H), 7.72 (d, *J* = 9.2 Hz, 2H), 7.35 - 7.28 (m, 5H), 5.24 (d, *J* = 1.2 Hz, 1H), 5.05 (s, 1H), 4.20 - 4.15 (m, 1H), 2.50 (d, *J* = 6.8 Hz, 2H), 2.46 (s, 2H), 0.86 (s, 6H); ^{13}C NMR (100 MHz, $CDCl_3$) δ 149.8, 145.6, 145.6, 143.2, 128.7, 128.1, 127.6, 126.3, 124.2, 118.0, 112.2, 52.7, 45.0, 35.3, 25.6; IR (neat): 3302, 1529, 1348, 1311, 1165, 1092, 1065, 907, 853, 781, 735, 685, 609, 558 cm^{-1}; HRMS (ESI) for $C_{19}H_{22}N_2O_4S$: calculated $[M + Na]^+$ *m/z* 397.1192, found 397.1192.

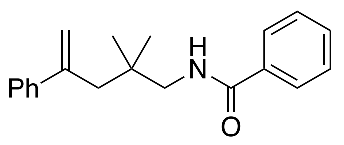

N-(2,2-Dimethyl-4-phenylpent-4-en-1-yl)benzamide (*S*-12d)

Amide ***S*-12d** was synthesized as previously reported.[14] Its ^{1}H NMR spectrum is in agreement with literature data.

^{1}H NMR (400 MHz, $CDCl_3$) δ 7.61 (d, J = 7.2 Hz, 2H), 7.55 - 7.23 (m, 8H), 5.90 (bs, 1H), 5.97 (s, 1H), 5.12 (s, 1H), 3.19 (d, J = 6.4 Hz, 2H), 2.55 (s, 2H), 0.90 (s, 6H).

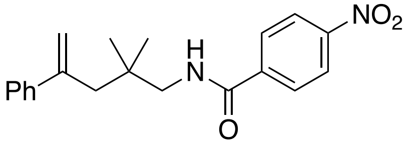

N-(2,2-Dimethyl-4-phenylpent-4-en-1-yl)-4-nitrobenzamide (*S*-12e)

2,2-Dimethyl-4-phenylpent-4-en-1-amine (200 mg) was converted to ***S*-12e** (283 mg, 80% yield, yellow solid) as above.

m.p. 82 – 84 ˚C. ^{1}H NMR (300 MHz, $CDCl_3$) δ 8.31 - 8.16 (d, J = 8.1 Hz, 2H), 7.74 - 7.61 (d, J = 7.7 Hz, 2H), 7.45 - 7.27 (m, 5H), 5.81 (bs, 1H), 5.31 (s, 1H), 5.12 (s, 1H), 3.18 (d, J = 6.6 Hz, 2H), 2.54 (s, 2H), 0.93 (s, 6H); ^{13}C NMR (75 MHz, $CDCl_3$) δ 165.3, 149.4, 146.0, 143.5, 140.3, 128.7, 127.9, 127.6, 126.4, 123.6, 117.7, 109.9, 48.8, 45.3, 36.3, 26.0; IR (neat): 2971, 1725, 1339, 1155, 661 cm^{-1}; HRMS (ESI) $C_{20}H_{22}N_2O_3$: calculated $[M + Na]^+$ m/z 361.1528, found 361.1523.

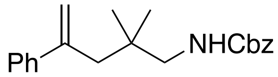

Benzyl (2,2-dimethyl-4-phenylpent-4-en-1-yl)carbamate (*S*-12f)

2,2-Dimethyl-4-phenylpent-4-en-1-amine (194 mg, 1.06 mmol) was converted to ***S*-12f** as above (211 mg, 62% yield, clear oil).

^{1}H NMR (400 MHz, $CDCl_3$) δ 7.44 - 7.16 (m, 10H), 5.25 (s, 1H), 5.06 (d, J = 10.5 Hz, 3H), 4.66 (bs, 1H), 2.94 (d, J = 6.4 Hz, 2H), 2.48 (s, 2H), 0.77 (s, 6H); ^{13}C NMR (100 MHz, $CDCl_3$) δ 156.6, 146.4, 143.2, 136.6, 128.4, 128.3, 128.0, 127.2, 126.3, 117.1, 66.5, 51.0, 44.9, 35.8, 25.4; IR (neat t): 3341, 2959, 1702, 1454, 1132, 778, 696; cm^{-1}; HRMS (ESI) for $C_{21}H_{26}NO_2$: calculated $[M + H]^+$ m/z 324.1964, found 324.1957.

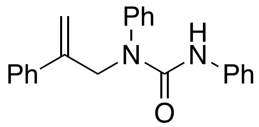

1,3-Diphenyl-1-(2-phenylallyl)urea (*S*-12g)

To a suspension of KOH (79 mg, 1.4 mmol, 1.2 equiv.) in THF (5 mL) was added 1,3-diphenylurea (250 mg, 1.2 mmol, 1 equiv.). The mixture was allowed to stir until the urea dissolved completely, then (3-bromoprop-1-en-2-yl)benzene (230 mg, 1.2 mmol, 1 equiv.) was added and the solution was allowed to stir at room temperature overnight. The mixture was then diluted with EtOAc (25 mL) and saturated NH_4Cl was added. The organic layer was separated and the aqueous layer was extracted with EtOAc (2 x 25 mL). The organic layers were combined and dried with Na_2SO_4, filtered, and concentrated *in vacuo*. The crude product was purified by flash chromatography on silica gel (15% EtOAc / hexanes) to afford 1,3-diphenyl-1-(2-phenylallyl)urea (280 mg, 71% yield) as a light yellow oil.

^{1}H NMR (400 MHz, $CDCl_3$) δ 7.46 (d, *J* = 6.8 Hz, 2H), 7.40 - 7.16 (m, 10H), 7.09 (d, *J* = 7.2 Hz, 2H), 6.98 (t, *J* = 6.8 Hz, 1H), 6.15 (s, 1H), 5.36 (s, 1H), 5.10 (s, 1H), 4.90 (s, 2H); ^{13}C NMR (100 MHz, $CDCl_3$) δ 159.0, 144.4, 140.5, 138.7, 138.6, 129.9, 128.7, 128.5, 128.3, 128.1, 127.8, 126.4, 122.9, 119.3, 115.3, 52.4; IR (neat): 3424, 3331, 3058, 2922, 1670, 1594, 1520, 1493, 1439, 1357, 1309, 1265, 1239, 1220, 1202, 1157, 1111, 1074, 1042, 1021, 943, 906, 854, 809, 777, 751, 720, 691, 647, 618, 593, 550, 528, 504 cm^{-1}; HRMS (ESI) for $C_{22}H_2N_2O$: calculated $[M + Na]^+$ *m/z* 351.1468, found 351.1475.

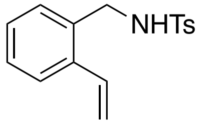

4-Methyl-*N*-(2-vinylbenzyl)benzenesulfonamide (*S*-13)

Sulfonamide ***S*-13** was synthesized as previously reported.[15] Its ^{1}H NMR spectrum is in agreement with literature data.

^{1}H NMR (400 MHz, $CDCl_3$) δ 7.76 (d, *J* = 8.0 Hz, 2H), 7.45 (d, *J* = 7.6 Hz, 1H), 7.35 - 7.10 (m, 4H), 6.77 (dd, *J* = 17.2, 11.2 Hz, 1H), 5.62 (d, *J* = 17.2 Hz, 1H), 5.29 (d, *J* = 10.8 Hz, 1H), 4.40 (bs, 1H), 4.17 (d, *J* = 6.0 Hz, 2H), 2.45 (s, 3H).

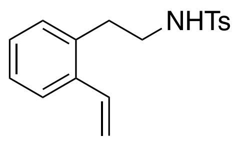

4-Methyl-*N*-(2-vinylphenethyl)benzenesulfonamide (*S*-14)

Sulfonamide ***S*-14** was synthesized as previously reported.[16] Its ^{1}H NMR spectrum is in agreement with literature data.

^{1}H NMR (400 MHz, $CDCl_3$) δ 7.68 (d, *J* = 8.4 Hz, 2H), 7.44 (d, *J* = 6.8 Hz, 1H), 7.29 - 7.16 (m, 4H), 7.04 (d, *J* = 7.2 Hz, 1H), 6.83 (dd, *J* = 17.2, 10.8 Hz, 1H), 5.59 (dd, *J* = 17.2, 1.2 Hz, 1H), 5.27 (dd, *J* = 10.8, 1.2 Hz, 1H), 4.33 (bs, 1H), 3.16 (q, *J* = 6.4 Hz, 2H), 2.86 (t, *J* = 7.2 Hz, 2H), 2.42 (s, 3H).

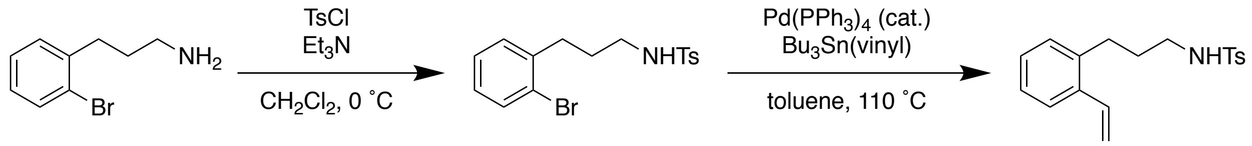

***N*-(3-(2-Bromophenyl)propyl)-4-methylbenzenesulfonamide (*S*-3)**

To a solution of 3-(2-bromophenyl)propan-1-amine (500 mg, 2.3 mmol, 1 equiv.), and trimethylamine (650 μL, 470 mg, 4.7 mmol, 2 equiv.) in dry CH_2Cl_2 (10 mL) was added tosyl chloride (490 mg, 2.6 mmol, 1.1 equiv.) at 0 °C. The reaction was allowed to warm to room temperature and stir overnight. The reaction was diluted with CH_2Cl_2 (30 mL) and 1 M HCl was added (50 mL). The organic layer was separated and the aqueous layer was extracted with CH_2Cl_2 (2 x 25 mL), the organic layers combined, dried over Na_2SO_4, filtered, and concentrated *in vacuo*. The crude product was purified by flash chromatography on silica gel (20% EtOAc / hexanes) to afford (780 mg, 91% yield) *N*-(3-(2-bromophenyl)propyl)-4-methylbenzenesulfonamide.

^{1}H NMR (400 MHz, $CDCl_3$) δ 7.77 (d, *J* = 8.0 Hz, 2H), 7.47 (d, *J* = 8.0 Hz, 1H), 7.29 (d, *J* = 8.4 Hz, 2H), 7.20 - 7.00 (m, 3H), 5.14 (t, *J* = 6.0 Hz, 1H), 2.99 (q, *J* = 6.4 Hz, 2H), 2.70 (t, *J* = 7.6 Hz, 2H), 2.41 (s, 3H), 1.81 - 1.73 (m, 2H); ^{13}C NMR (100 MHz, $CDCl_3$) δ 143.3, 140.2, 136.8, 132.7, 130.3, 129.6, 127.7, 127.4, 127.0, 124.2, 42.5, 32.9, 29.4, 21.4; IR (neat): 3276, 2926, 2867, 1598, 1567, 1495, 1471, 1439, 1322, 1305, 1156, 1121, 1093, 1020, 958, 908, 839, 751, 732, 706, 660, 570, 550 cm^{-1}; HRMS (ESI) for $C_{16}H_{18}BrNO_2S$: calculated $[M + H]^+$ *m/z* 368.0314, found 368.0318.

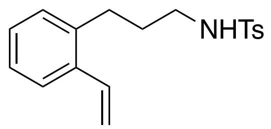

4-Methyl-*N*-(3-(2-vinylphenyl)propyl)benzenesulfonamide (*S*-15)

Following a reported procedure,[16] ***S*-3** (600 mg, 1.6 mmol, 1 equiv.) was treated with $Pd(PPh_3)_4$ (94 mg, 0.082 mmol, 5 mol%) and Bu_3Sn(vinyl) (580 mg, 1.8 mmol, 1.2 equiv.) in toluene (6 mL) under argon in a round bottom flask equipped with a stir bar. The reaction was heated to 120 ˚C for 24 h. Upon cooling to room temperature, the reaction mixture was diluted with Et_2O (40 mL) and washed with water (3 x 30 mL). The organic layer was concentrated *in vacuo* and purified via flash chromatography on silica gel (20% EtOAc / hexanes) to yield 4-methyl-*N*-(3-(2-vinylphenyl)propyl)benzenesulfonamide (400 mg, 77% yield) as a light yellow oil.

^{1}H NMR (400 MHz, $CDCl_3$) δ 7.77 (d, *J* = 8.4 Hz, 2H), 7.46 (d, *J* = 8.4 Hz, 1H), 7.30 (d, *J* = 8.4 Hz, 2H), 7.22 - 7.12 (m, 2H), 7.03 (d, *J* = 8.4 Hz, 1H), 6.88 (dd, *J* = 17.6, 7.2 Hz, 1H), 5.51 (dd, *J* = 17.2, 1.2 Hz, 1H), 5.26 (dd, *J* = 11.2, 0.8 Hz, 1H), 5.01 (t, *J* = 6.4 Hz, 1H), 2.97 (q, *J* = 6.4 Hz, 2H), 2.66 (t, *J* = 8.4 Hz, 2H), 2.42 (s, 3H), 1.80 - 1.68 (m, 2H); ^{13}C NMR (100 MHz, $CDCl_3$) δ 143.3, 138.2, 136.9, 136.4, 129.7, 129.4, 127.8, 127.0, 126.5, 125.9, 115.8, 42.8, 30.6, 30.1, 21.5; IR (neat): 3277, 2925, 2870, 1625, 1598, 1484, 1449, 1418, 1323, 1305, 1184, 1156, 1093, 1019, 990, 959, 910, 814, 773, 733, 707, 662, 550 cm^{-1}; HRMS (ESI) for $C_{18}H_{21}NO_2S$: calculated $[M + Na]^+$ *m/z* 338.1185, found 338.1189.

General Procedure for Synthesis of Lactone and Cyclic Ether Products 2 - 11

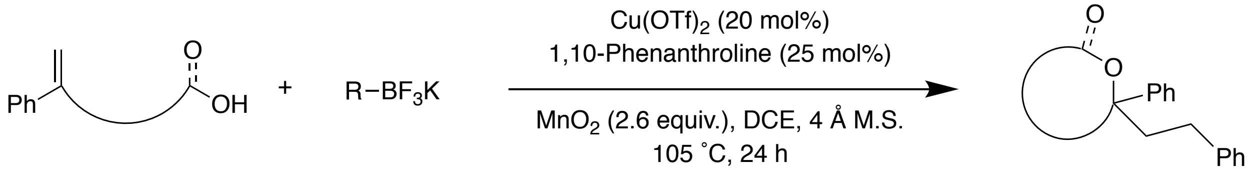

To an oven-dried pressure tube, $Cu(OTf)_2$ (9 mg, 0.025 mmol, 20 mol%) was flame-dried under vacuum and flushed with argon then, 1, 10-phenanthroline (5.6 mg, 0.031 mmol, 25 mol%) was added. 1, 2-Dichloroethane was then added (1 mL). The mixture was heated at 60 ˚C for 2 h then cooled to room temperature. Flame dried 4 Å molecular sieves (20 mg) were added and the reaction was stirred at room temperature for 10 min. Alkenoic acid or alkenyl alcohol (0.19 mmol, 1.5 equiv.), potassium alkyltrifluoroborate (0.13 mmol, 1 equiv.), and MnO_2 (33 mg, 0.32 mmol, 2.6 equiv.) were added. The tube was sealed and heated to 105 ˚C for 24 h. The reaction mixture was

allowed to cool to room temperature and diluted with EtOAc (5 mL) and filtered through a pad of silica gel (~5 cm) with EtOAc (3 x 50 mL). The combined filtrate was concentrated *in vacuo* and the crude product was purified by flash chromatography on silica gel using EtOAc / hexanes.

Characterization of Novel Lactone and Cyclic Ether Products 2 - 11

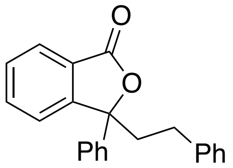

3-Phenethyl-3-phenylisobenzofuran-1(3*H*)-one (2a)

Alkenoic acid **1a** (42 mg, 0.19 mmol) and potassium benzyltrifluoroborate (25 mg, 0.13 mmol) were converted to 33 mg **2a** (84% yield, clear oil) as above.

^{1}H NMR (300 MHz, $CDCl_3$) δ 7.92 (dd, J = 7.5, 1.0 Hz, 1H), 7.70 - 7.64 (m, 1H), 7.60 - 7.48 (m, 4H), 7.45 - 7.25 (m, 3H), 7.25 - 7.03 (m, 5H), 2.86 - 2.73 (m, 1H), 2.68 - 2.56 (m, 1H), 2.54 - 2.37 (m, 2H); ^{13}C NMR (100 MHz, $CDCl_3$) δ 170, 152.8, 140.9, 140.2, 134.4, 129.2, 128.9, 128.5, 128.2, 126.1, 126.0, 125.4, 124.9, 122.0, 89.7, 42.3, 30.1; IR (neat): 3027, 2923, 1760, 1599, 750 cm^{-1}; HRMS (ESI) for $C_{22}H_{18}O_2$: calculated $[M + Na]^+$ *m/z* 337.1199, found 337.1197.

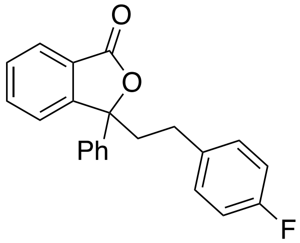

3-(4-Fluorophenethyl)-3-phenylisobenzofuran-1(3*H*)-one (2b)

Alkenoic acid **1a** (42 mg, 0.19 mmol) and potassium 4-fluorobenzyltrifluoroborate (27 mg, 0.13 mmol) were converted to 34 mg **2a** (81% yield, clear oil) as above.

^{1}H NMR (400 MHz, $CDCl_3$) δ 7.91 (d, J = 7.2 Hz, 1H), 7.67 (t, J = 7.6 Hz, 1H), 7.59 - 7.49 (m, 4H), 7.42 - 7.20 (m, 3H), 7.07 - 7.01 (m, 2H), 6.91 (t, J = 8.8 Hz, 2H), 2.82 - 2.71 (m, 1H), 2.64 - 2.52 (m, 1H), 2.49 - 2.37 (m, 2H); ^{13}C NMR (100 MHz, $CDCl_3$) δ 170.0, 161.3 (d, ^{1}J(C-F) = 243 Hz), 152.7, 140.1, 136.4, 134.4, 129.6, 129.6, 129.3, 128.9, 128.3, 126.0, 125.3, 125.8, 122.0, 115.3, 115.1, 89.6, 42.4, 29.4; ^{19}F NMR (282 MHz, $CDCl_3$) δ -118.1 (s); IR (neat): 2933, 1763,

1599, 1509, 1466, 1448, 1287, 1221, 1158, 1090, 1017, 949, 834, 766, 701, 610, 525, 504 cm^{-1}; HRMS (ESI) for $C_{22}H_{17}O_2$: calculated $[M + Na]^+$ *m/z* 355.1105, found 355.1103.

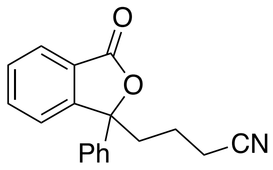

4-(3-Oxo-1-phenyl-1,3-dihydroisobenzofuran-1-yl)butanenitrile (2c)

Alkenoic acid **1a** (42 mg, 0.19 mmol) and potassium 2-cyanoethyltrifluoroborate (20 mg, 0.13 mmol) were converted to 23 mg **2c** (66% yield, clear oil) as above.

^{1}H NMR (400 MHz, $CDCl_3$) δ 7.90 (d, *J* = 7.6 Hz, 1H), 7.69 (t, *J* = 7.2 Hz, 1H), 7.60 - 7.50 (m, 4H), 7.42 - 7.27 (m, 3H), 2.72 - 2.62 (m, 1H), 2.39 - 2.24 (m, 3), 1.74 - 1.54 (m, 2H); ^{13}C NMR (100 MHz, $CDCl_3$) δ 169.6, 152.5, 139.5, 134.7, 129.5, 129.0, 128.4, 126.1, 125.0, 124.7, 122.0, 119.0, 89.0, 39.1, 20.1, 17.0; IR (neat): 2943, 2245, 1762, 1598, 1496, 1465, 1448, 1333, 1287, 1251, 1099, 1045, 1017, 954, 770, 753, 721, 701, 606 cm^{-1}; HRMS (ESI) for $C_{18}H_{15}NO_2$: calculated $[M + Na]^+$ *m/z* 300.0995, found 300.0990.

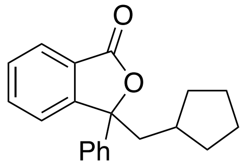

3-(Cyclopentylmethyl)-3-phenylisobenzofuran-1(3*H*)-one (2d)

Alkenoic acid **1a** (42 mg, 0.19 mmol) and potassium cyclopentyltrifluoroborate (22 mg, 0.13 mmol) were converted to 15 mg **2d** (42% yield, clear oil) as above.

^{1}H NMR (300 MHz, $CDCl_3$) δ 7.88 (dd, *J* = 7.7, 1.0 Hz, 1H), 7.65 (t, *J* = 7.5 Hz, 1H), 7.59 - 7.46 (m, 4H), 7.41 - 7.15 (m, 2H), 2.61 (dd, *J* = 14.6, 5.4 Hz, 1H), 2.27 (dd, *J* = 14.6, 6.2 Hz, 1H), 1.63 (q, *J* = 7.5, 7.0 Hz, 1H), 1.55 - 1.28 (m, 2H), 1.21 - 0.80 (m, 1H); ^{13}C NMR (75 MHz, $CDCl_3$) δ 170.1, 153.3, 141.2, 134.1, 129.0, 128.7, 128.0, 125.8, 125.5, 124.8, 122.4, 90.3, 46.4, 35.6, 33.8, 33.7, 24.9, 24.8; IR (neat): 2948, 2867, 1759, 1598, 956, 722 cm^{-1}; HRMS (ESI) for $C_{20}H_{20}O_2$: calculated $[M + Na]^+$ *m/z* 315.1356, found 315.1354.

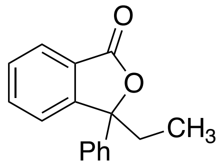

3-Ethyl-3-phenylisobenzofuran-1(3*H*)-one (2e)

Alkenoic acid **1a** (42 mg, 0.19 mmol) and potassium methyltrifluoroborate (15 mg, 0.13 mmol) were converted to 18 mg **2e** (60% yield, clear oil) as above, and in agreement with literature data.[17]

^{1}H NMR (300 MHz, $CDCl_3$) δ 7.88 (d, *J* = 8.1 Hz, 1H), 7.66 (t, *J* = 6.9 Hz, 1H), 7.54 - 7.37 (m, 4H), 7.40 - 7.21 (m, 3H), 2.59 - 2.41 (m, 1H), 2.37 - 2.17 (m, 1H), 0.81 (t, *J* = 7.5 Hz, 3H); ^{13}C NMR (75 MHz, $CDCl_3$) δ 152.7, 140.4, 134.2, 129.1, 128.7, 128.1, 125.8, 125.7, 125.0, 122.1, 90.4, 33.3, 8.1.

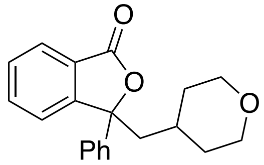

3-Phenyl-3-((tetrahydro-2*H*-pyran-4-yl)methyl)isobenzofuran-1(3*H*)-one (2f)

Alkenoic acid **1a** (42 mg, 0.19 mmol) and potassium tetrahydro-2*H*-pyran-4-trifluoroborate (24 mg, 0.13 mmol) were converted to 29 mg **2f** (75% yield, clear oil) as above using 10 mol% $Cu(OTf)_2$ and 12 mol% 1,10-phenanthroline.

^{1}H NMR (500 MHz, $CDCl_3$) δ 7.88 (d, *J* = 7.5 Hz, 1H), 7.65 (t, *J* = 7.3 Hz, 1H), 7.58 - 7.47 (m, 4H), 7.40 - 7.33 (m, 2H), 7.30 (td, *J* = 7.2, 6.3, 3.2 Hz, 1H), 3.90 - 3.66 (m, 2H), 3.20 (td, *J* = 28.0, 11.8, 2.3 Hz, 2H), 2.51 (dd, *J* = 14.8, 4.9 Hz, 1H), 2.06 (dd, *J* = 14.8, 6.7 Hz, 1H), 1.50 - 1.42 (m, 2H), 1.42 - 1.25 (m, 2H); ^{13}C NMR (125 MHz, $CDCl_3$) δ 170.0, 153.6, 140.7, 134.4, 129.2, 128.8, 128.1, 126.0, 124.9, 124.6, 122.1, 89.9, 67.7,67.7, 47.3, 34.1, 33.9, 31.2; IR (neat): 2915, 2842, 1761, 1598, 1098, 703 cm^{-1}; HRMS (ESI) for $C_{20}H_{20}O_3$: calculated $[M + H]^+$ *m/z* 309.1481, found 309.1481.

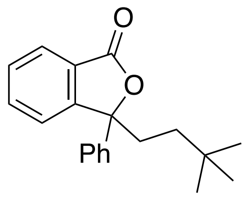

3-(3,3-Dimethylbutyl)-3-phenylisobenzofuran-1(3*H*)-one (2g)

Alkenoic acid **1a** (42 mg, 0.19 mmol) and potassium 2,2-dimethylpropyltrifluoroborate (22 mg, 0.13 mmol) were converted to 15 mg **2g** (41% yield, clear oil) as above.

^{1}H NMR (400 MHz, $CDCl_3$) δ 7.99 (d, J = 8.0 Hz, 1H), 7.66 (t, J = 7.2 Hz, 1H), 7.56 - 7.47 (m, 4H), 7.40 - 7.26 (m, 3H), 2.44 (td, J = 14.0, 4.4 Hz, 1H), 2.16 (td, J = 14.0, 4.0 Hz, 1H), 1.18 (td, J = 12.8, 4.0 Hz, 1H), 0.96 (td, J = 12.8, 4.0 Hz, 1H), 0.82 (s, 9H); ^{13}C NMR (100 MHz, $CDCl_3$) δ 170.0, 153.1, 140.7, 134.2, 129.0, 128.7, 128.0, 125.9, 125.5, 124.9, 122.0, 90.2, 37.0, 35.5, 29.9, 29.1; IR (neat): 2954, 2865, 1760, 1611, 1599, 1496, 1466, 1447, 1394, 1366, 1286, 1242, 1196, 1097, 1051, 1016, 983, 957, 933, 917, 771, 752, 720, 701, 660, 628, 609, 578, 535 cm^{-1}; HRMS (ESI) for $C_{20}H_{22}O_2$: calculated $[M + Na]^+$ m/z 317.1512, found 317.1510.

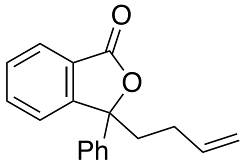

3-(But-3-en-1-yl)-3-phenylisobenzofuran-1(3*H*)-one (2h)

Alkenoic acid **1a** (42 mg, 0.19 mmol) and potassium allyltrifluoroborate (19 mg, 0.13 mmol) were converted to 9 mg **2h** (26% yield, clear oil) as above.

^{1}H NMR (400 MHz, $CDCl_3$) δ 7.89 (d, J = 8.0 Hz, 1H), 7.67 (t, J = 7.6 Hz, 1H), 7.57 - 7.47 (m, 4H), 7.39 - 7.25 (m, 3H), 5.78 - 5.66 (m, 1H), 4.96 - 4.87 (m, 2H), 2.62 - 2.52 (m, 1H), 2.33 - 2.23 (m, 1H), 2.09 - 1.97 (m, 1H), 1.95 - 1.82 (m, 1H); ^{13}C NMR (100 MHz, $CDCl_3$) δ 170.0, 152.7, 140.1, 137.0, 134.3, 129.4, 129.2, 128.8, 128.2, 125.9, 124.9, 122.1, 115.2, 89.7, 39.4, 28.0; IR (neat): 2923, 1762, 1641, 1598, 1496, 1465, 1447, 1333, 1286, 1254, 1089, 1001, 957, 915, 752, 722, 701, 644, 535 cm^{-1}; HRMS (ESI) for $C_{18}H_{16}O_2$: calculated $[M + Na]^+$ m/z 287.1043, found 287.1040.

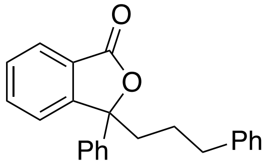

3-Phenyl-3-(3-phenylpropyl)isobenzofuran-1(3*H*)-one (2i)

Alkenoic acid **1a** (42 mg, 0.19 mmol) and potassium phenethyltrifluoroborate (27 mg, 0.13 mmol) were converted to 25 mg **2i** (60% yield, clear oil) as above.

^{1}H NMR (400 MHz, $CDCl_3$) δ 7.88 (d, *J* = 7.6 Hz, 1H), 7.64 (t, *J* = 7.2 Hz, 1H), 7.53 - 7.45 (m, 4H), 7.39 - 7.12 (m, 6H), 7.07 (d, *J* = 6.8 Hz, 2H), 2.59 (t, *J* = 7.6 Hz, 2H), 2.56 - 2.44 (m, 1H), 2.24 - 2.15 (m 1H), 1.71 - 1.57 (m, 1H), 1.53 - 1.38 (m, 1H); ^{13}C NMR (100 MHz, $CDCl_3$) δ 170.0, 152.9, 141.4, 134.2, 129.1, 128.7, 128.3, 128.1, 125.9, 125.9, 125.4, 124.9, 122.1, 90.0, 39.7, 35.4, 25.2; IR (neat): 3026, 2946, 1760, 1599, 1495, 1465, 1448, 1334, 1286, 1246, 1099, 1080, 1030, 952, 910, 768, 751, 699, 614, 563, 534, 512 cm^{-1}; HRMS (ESI) for $C_{23}H_{20}O_2$: calculated $[M + Na]^+$ *m/z* 351.1356, found 351.1355.

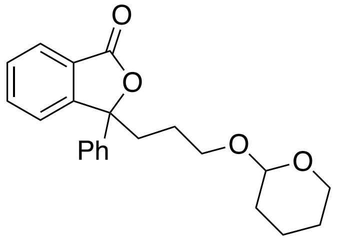

3-Phenyl-3-(3-((tetrahydro-2*H*-pyran-2-yl)oxy)propyl)isobenzofuran-1(3*H*)-one (2j)

Alkenoic acid **1a** (42 mg, 0.19 mmol) and potassium 2-(tetrahydro-2*H*-pyran-2-yloxy)ethyltrifluoroborate (30 mg, 0.13 mmol) were converted to 12 mg **2j** (28% yield, clear oil) as above.

^{1}H NMR (400 MHz, $CDCl_3$) δ 7.88 (d, *J* = 8.0 Hz, 1H), 7.66 (t, *J* = 7.2 Hz, 1H), 7.56 - 7.47 (m, 4H), 7.39 - 7.26 (m, 3H), 4.49 (s, 1H), 3.57 - 3.55 (m, 1H), 3.65 - 3.53 (m, 1H), 3.49 - 3.41 (m, 1H), 3.39 - 3.30 (m, 1H), 2.63 - 2.50 (m, 1H), 2.36 - 2.24 (m, 1H), 1.85 - 1.34 (m, 8H); ^{13}C NMR (100 MHz, $CDCl_3$) δ 170.0, 152.9, 140.3, 140.3, 134.3, 129.1, 128.7, 128.1, 125.9, 125.4, 124.9, 122.2, 122.2, 98.8, 89.9, 66.8, 62.4, 37.0, 30.9, 30.7, 25.4, 24.2, 19.6; IR (neat): 2941, 1764, 1598, 1465, 1447, 1351, 1286, 1200, 1120, 1075, 1033, 964, 907, 868, 813, 752, 702 cm^{-1}; HRMS (ESI) for $C_{22}H_{24}O_4$: calculated $[M + Na]^+$ *m/z* 375.1567, found 375.1567.

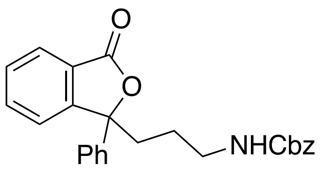

Benzyl (3-(3-oxo-1-phenyl-1,3-dihydroisobenzofuran-1-yl)propyl)carbamate (2k)

Alkenoic acid **1a** (42 mg, 0.19 mmol) and potassium benzyl *N*-[2-(trifluoroboranuidyl)ethyl]carbamate (36 mg, 0.13 mmol) were converted to 15 mg **2k** (30% yield, clear oil) as above.

^{1}H NMR (300 MHz, $CDCl_3$) δ 7.87 (d, *J* = 7.8 Hz, 1H), 7.65 (t, *J* = 7.8 Hz, 1H), 7.57 - 7.46 (m, 4H), 7.39 - 7.27 (m, 7H), 5.06 (s, 2H), 4.68 (bs, 1H), 3.18 (q, *J* = 6.3 Hz, 2H), 2.59 - 2.43 (m, 1H), 2.27 - 2.12 (m, 1H), 1.60 - 1.30 (m, 2H); ^{13}C NMR (75 MHz, $CDCl_3$) δ 169.9, 156.3, 152.8, 140.1, 136.5, 134.4, 129.2, 128.8, 128.5, 128.2, 128.1, 125.9, 125.2, 124.8, 122.1, 89.6, 66.7, 40.6, 37.4, 24.6; IR (neat): 3343, 3033, 2936, 1758, 1703, 1611, 1598, 1522, 1497, 1465, 1448, 1372, 1334, 1286, 1240, 1128, 1085, 1067, 1026, 961, 911, 771, 751, 734, 697, 646, 609, 577, 535 cm^{-1}; HRMS (ESI) for $C_{25}H_{23}NO_4$: calculated $[M + Na]^+$ *m/z* 424.1519, found 424.1518.

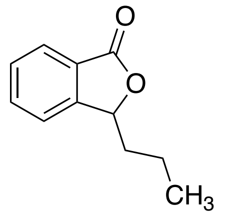

3-Propylisobenzofuran-1(3*H*)-one (2l)

Alkenoic acid **1c** (28 mg, 0.19 mmol) and potassium ethyltrifluoroborate (17 mg, 0.13 mmol) were converted to 7 mg **2l** (30% yield, clear oil) as above, and in agreement with literature data.[18]

^{1}H NMR (300 MHz, $CDCl_3$) δ 7.89 (d, *J* = 7.5 Hz, 1H), 7.67 (t, *J* = 7.5 Hz, 1H), 7.52 (t, *J* = 7.2 Hz, 1H), 7.43 (d, *J* = 7.8 Hz, 1H), 5.48 (dd, *J* = 7.8, 3.6 Hz, 1H), 2.04 - 1.96 (m, 1H), 1.81 - 1.64 (m, 1H), 1.62 - 1.40 (m, 2H), 0.98 (t, *J* = 6.9 Hz, 3H); ^{13}C NMR (75 MHz, $CDCl_3$) δ 170.7, 150.2, 133.9, 129.0, 126.1, 125.7, 121.7, 81.2, 36.8, 18.2, 13.8.

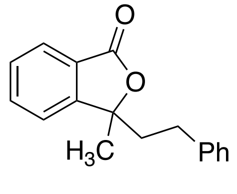

3-Methyl-3-phenethylisobenzofuran-1(3*H*)-one (2m)

Alkenoic acid **1d** (30 mg, 0.19 mmol) and potassium benzyltrifluoroborate (25 mg, 0.13 mmol) were converted to 20 mg **2m** (64% yield, clear oil) as above.

^{1}H NMR (400 MHz, $CDCl_3$) δ 7.91 (d, J = 7.2 Hz, 1H), 7.67 (t, J = 7.6 Hz, 1H), 7.53 (t, J = 7.2 Hz, 1H), 7.38 (d, J = 7.6 Hz, 1H), 7.23 (t, J = 7.6 Hz, 2H), 7.15 (t, J = 7.2 Hz, 1H), 7.07 (d, J = 6.8 Hz, 2H), 2.63 (td, J = 12.0, 4.0 Hz, 1H), 2.39 (td, J = 13.6, 5.2 Hz, 1H), 2.29 - 2.12 (m, 2H), 1.70 (s, 3H); ^{13}C NMR (100 MHz, $CDCl_3$) δ 170.0, 153.5, 140.9, 135.0, 129.1, 128.4, 128.2, 126.0, 125.9, 120.8, 87.3, 41.8, 29.9, 26.2; IR (neat): 3027, 2977, 2931, 1755, 1614, 1600, 1497, 1466, 1455, 1378, 1338, 1311, 1286, 1258, 1218, 1166, 1120, 1080, 1034, 946, 914, 758, 721, 696, 618, 596, 558 cm^{-1}; HRMS (ESI) for $C_{17}H_{16}O_2$: calculated $[M + Na]^+$ m/z 275.1043, found 275.1042.

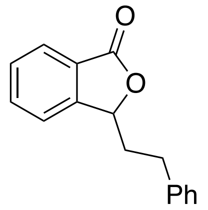

3-Phenethylisobenzofuran-1(3*H*)-one (2n)

Alkenoic acid **1d** (28 mg, 0.19 mmol) and potassium benzyltrifluoroborate (25 mg, 0.13 mmol) were converted to 12 mg **2n** (39% yield, clear oil) as above.

^{1}H NMR (400 MHz, $CDCl_3$) δ 7.91 (d, J = 7.6 Hz, 1H), 7.66 (td, J = 7.5, 1.2 Hz, 1H), 7.53 (t, J = 7.5 Hz, 1H), 7.44 - 7.37 (m, 1H), 7.30 (dd, J = 8.1, 6.7 Hz, 1H), 7.22 (dd, J = 7.7, 2.1 Hz, 2H), 5.47 (dd, J = 8.8, 3.5 Hz, 1H), 2.94 - 2.76 (m, 2H), 2.46 - 2.26 (m, 1H), 2.13 - 1.95 (m, 1H); ^{13}C NMR (100 MHz, $CDCl_3$) δ 170.6, 149.8, 140.5, 134.0, 129.1, 128.6, 128.5, 126.3, 125.8, 121.6, 110.0, 80.3, 36.7, 31.3; IR (neat): 3027, 2924, 1755, 1601, 750, 695 cm^{-1}; HRMS (ESI) for $C_{16}H_{14}O_2$: calculated $[M + Na]^+$ m/z 261.0886, found 261.0887.

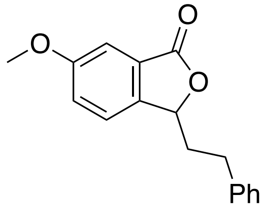

6-methoxy-3-phenethylisobenzofuran-1(3*H*)-one (2o)

Alkenoic acid **1e** (33 mg, 0.19 mmol) and potassium benzyltrifluoroborate (25 mg, 0.13 mmol) were converted to 12 mg **2o** (36% yield, clear oil) as above.

^{1}H NMR (300 MHz, $CDCl_3$) δ 7.36 - 7.16 (m, 8H), 5.41 (dd, *J* = 6.6, 2.1 Hz, 1H), 3.87 (s, 3H), 2.88 - 2.78 (m, 2H), 2.36 - 2.26 (m, 1H), 2.06 - 1.95 (m, 1H); ^{13}C NMR (75 MHz, $CDCl_3$) δ 170.6, 160.7, 128.6, 128.5, 127.5, 126.2, 123.0, 122.5, 107.5, 80.3, 55.8, 36.8, 31.2; IR (neat): 3026, 2931, 2838, 1754, 1623, 1603, 1494, 1454, 1434, 1321, 1279, 1244, 1197, 1167, 1114, 1086, 1053, 1019, 934, 851, 829, 774, 752, 730, 700, 591, 562 cm^{-1}; HRMS (ESI) $C_{17}H_{16}O_3$: calculated $[M + Na]^+$ *m/z* 291.0992, found 291.0998.

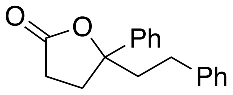

5-Phenethyl-5-phenyldihydrofuran-2(3*H*)-one (5a)

Alkenoic acid **1b** (33 mg, 0.19 mmol) and potassium benzyltrifluoroborate (25 mg, 0.13 mmol) were converted to 21 mg **5a** (62% yield, clear oil) as above.

^{1}H NMR (400 MHz, $CDCl_3$) δ 7.47 - 7.29 (m, 5H), 7.26 - 7.02 (m, 5H), 2.81 - 2.17 (m, 8H); ^{13}C NMR (100 MHz, $CDCl_3$) δ 176.6, 142.5, 141.2, 128.7, 128.4, 128.2, 127.7, 126.0, 124.6, 89.1, 44.4, 35.5, 30.3, 28.6; IR (neat): 3026, 2933, 1773, 1602, 1192, 766, 700 cm^{-1}; HRMS (ESI) for $C_{18}H_{18}O_2$: calculated $[M + Na]^+$ *m/z* 289.1199, found 289.1197.

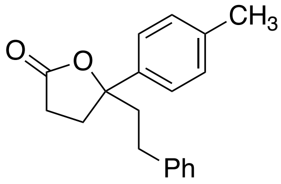

5-Phenethyl-5-(*p*-tolyl)dihydrofuran-2(3*H*)-one (5b)

Alkenoic acid **1f** (36 mg, 0.19 mmol) and potassium benzyltrifluoroborate (25 mg, 0.13 mmol) were converted to 25 mg **5b** (70% yield, clear oil) as above.

^{1}H NMR (400 MHz, $CDCl_3$) δ 7.30 - 7.13 (m, 7H), 7.08 (d, *J* = 7.2 Hz, 2H), 2.75 - 2.20 (m, 8H), 2.37 (s, 3H); ^{13}C NMR (100 MHz, $CDCl_3$) δ 176.6, 141.3, 139.4, 137.4, 129.3, 128.4, 128.2, 125.9, 124.5, 89.1, 44.4, 35.5, 30.3, 28.6, 21.0; IR (neat): 3026, 2922, 1773, 1603, 1514, 1497, 1454, 1418, 1297, 1279, 1225, 1194, 1180, 1117, 1082, 1068, 1042, 1020, 936, 884, 818, 776, 752, 726, 700, 654, 636, 608, 588, 572, 539, 508 cm^{-1}; HRMS (ESI) for $C_{19}H_{20}O_2$: calculated $[M + Na]^+$ *m/z* 303.1356, found 303.1359.

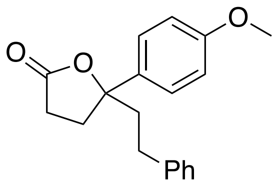

5-(4-Methoxyphenyl)-5-phenethyldihydrofuran-2(3*H*)-one (5c)

Alkenoic acid **1g** (39 mg, 0.19 mmol) and potassium benzyltrifluoroborate (25 mg, 0.13 mmol) were converted to 27 mg **5c** (72% yield, clear oil) as above.

^{1}H NMR (400 MHz, $CDCl_3$) δ 7.32 - 7.21 (m, 4H), 7.16 (t, *J* = 7.6 Hz, 1H), 7.08 (d, *J* = 6.8 Hz, 2H), 6.93 (d, *J* = 8.8 Hz, 2H), 3.83 (s, 3H), 2.72 - 2.21 (m, 8H); ^{13}C NMR (100 MHz, $CDCl_3$) δ 176.6, 159.0, 141.3, 134.4, 128.4, 128.2, 126.0, 125.9, 114.0, 89.0, 55.3, 44.5, 35.4, 30.3, 28.6; IR (neat): 2936, 2837, 1771, 1612, 1583, 1513, 1497, 1455, 1417, 1304, 1249, 1175, 1116, 1068, 1030, 935, 885, 834, 807, 777, 701, 661, 630, 684, 554 cm^{-1}; HRMS (ESI) for $C_{19}H_{20}O_3$: calculated $[M + Na]^+$ *m/z* 319.1305, found 319.1309.

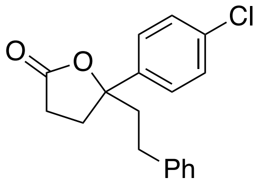

5-(4-Chlorophenyl)-5-phenethyldihydrofuran-2(3*H*)-one (5d)

Alkenoic acid **1h** (40 mg, 0.19 mmol) and potassium benzyltrifluoroborate (25 mg, 0.13 mmol) were converted to 28 mg **5d** (74% yield, off-white solid) as above.

m.p. 90 - 92 ˚C; ^{1}H NMR (300 MHz, $CDCl_3$) δ 7.42 - 7.30 (m, 4H), 7.25 (t, *J* = 7.5 Hz, 3H), 7.07 (d, *J* = 6.9 Hz, 2H), 2.75 - 2.41 (m, 3H), 2.37 - 2.21 (m, 3H); ^{13}C NMR (75 MHz, $CDCl_3$) δ 176.1, 141.1, 140.9, 133.7, 128.9, 128.5, 128.2, 126.1, 88.5, 44.3, 35.4, 30.2, 28.5; IR (neat): 3027, 2945, 1774, 1602, 1490, 1455, 1419, 1401, 1294, 1220, 1191, 1164, 1093, 1067, 1042, 1012, 962, 936, 910, 883, 833, 777, 728, 700, 670, 622, 552, 509 cm^{-1}; HRMS (ESI) for $C_{18}H_{17}ClO_2$: calculated $[M + Na]^+$ *m/z* 323.0809, found 323.0806.

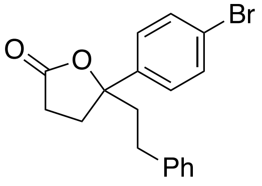

5-(4-Bromophenyl)-5-phenethyldihydrofuran-2(3*H*)-one (5e)

Alkenoic acid **1i** (48 mg, 0.19 mmol) and potassium benzyltrifluoroborate (25 mg, 0.13 mmol) were converted to 30 mg **5e** (70% yield, off-white solid) as above.

m.p. 93 - 94 ˚C; ^{1}H NMR (300 MHz, $CDCl_3$) δ 7.53 (d, *J* = 8.7 Hz, 2H), 7.31 - 7.13 (m, 5H), 7.07 (d, *J* = 6.9 Hz, 2H), 2.75 - 2.20 (m, 8H); ^{13}C NMR (75 MHz, $CDCl_3$) δ 176.1, 141.7, 140.9, 131.8, 128.5, 128.2, 126.4, 126.1, 121.8, 88.5, 44.3, 35.3, 30.2, 28.4; IR (neat): 3026, 2934, 1775, 1593, 1486, 1455, 1418, 1396, 1292, 1223, 1191, 1164, 1110, 1065, 1042, 1008, 962, 936, 883, 823, 777, 749, 723, 700, 667, 615, 550, 505 cm^{-1}; HRMS (ESI) for $C_{18}H_{17}BrO_2$: calculated $[M + Na]^+$ *m/z* 367.0304, found 367.0303.

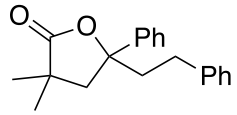

3,3-Dimethyl-5-phenethyl-5-phenyldihydrofuran-2(3*H*)-one (5f)

Alkenoic acid **1j** (38 mg, 0.19 mmol) and potassium benzyltrifluoroborate (25 mg, 0.13 mmol) were converted to 21 mg **5f** (57% yield, clear oil) as above.

^{1}H NMR (400 MHz, $CDCl_3$) δ 7.50 - 7.20 (m, 7H), 7.18 - 7.01 (m, 3H), 2.71 (td, *J* = 11.2, 3.9 Hz, 1H), 2.56, 2.39 (ABq, *J* = 13.2 Hz, 2H), 2.32 - 2.11 (m, 3H), 1.33 (s, 3H), 0.96 (s, 3H); ^{13}C NMR (100 MHz, $CDCl_3$) δ 176.6, 142.5, 141.2, 128.7, 128.4, 128.2, 127.7, 126.0, 124.6, 89.1, 44.4, 35.5, 30.3, 28.6; IR (neat): 3027, 2971, 1764, 1602, 926, 723 cm^{-1}; HRMS (ESI) for $C_{20}H_{22}O_2$: calculated $[M + Na]^+$ *m/z* 317.1512, found 317.1510.

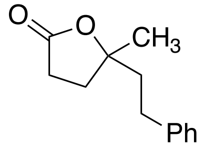

5-Methyl-5-phenethyldihydrofuran-2(3*H*)-one (5g)

Alkenoic acid **1k** (38 mg, 0.19 mmol) and potassium benzyltrifluoroborate (25 mg, 0.13 mmol) were converted to 21 mg **5g** (57% yield, clear oil) as above, and in agreement with literature data.[19]

^{1}H NMR (400 MHz, $CDCl_3$) δ 7.30 (t, *J* = 7.2 Hz, 2H), 7.20 (t, *J* = 8.0 Hz, 3H), 2.78 - 2.56 (m, 4H), 2.19 - 2.10 (m, 1H), 2.09 - 1.95 (m, 3H), 1.47 (s, 3H); ^{13}C NMR (100 MHz, $CDCl_3$) δ 176.6, 141.2, 128.5, 128.2, 126.1, 86.3, 42.9, 33.1, 30.2, 29.1, 25.6.

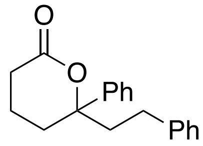

6-Phenethyl-6-phenyltetrahydro-2*H*-pyran-2-one (6a)

Alkenoic acid **1l** (36 mg, 0.19 mmol) and potassium benzyltrifluoroborate (25 mg, 0.13 mmol) were converted to 20 mg **6a** (54% yield, clear oil) as above.

^{1}H NMR (400 MHz, $CDCl_3$) δ 7.44 - 7.28 (m, 5H), 7.23 (t, *J* = 7.6 Hz, 2H), 7.15 (t, *J* = 7.2 Hz, 1H), 7.08 (d, *J* = 7.2 Hz, 2H), 2.80 (td, *J* = 12.4, 5.2 Hz, 1H), 2.53 - 2.44 (m, 2H), 2.38 - 2.14 (m, 4H), 2.06 - 2.00 (m, 1H), 1.83 - 1.72 (m, 1H), 1.64 - 1.52 (m, 1H); ^{13}C NMR (100 MHz, $CDCl_3$) δ 171.2, 142.7, 141.6, 128.8, 128.4, 128.3, 127.4, 125.8, 125.0, 87.4, 45.8, 33.3, 29.4, 29.3, 16.3; IR (neat): 3060, 3026, 2953, 1730, 1602, 1496, 1447, 1328, 1237, 1182, 1093, 1046, 1000, 950, 933, 804, 767, 753, 700, 657, 611, 538 cm^{-1}; HRMS (ESI) for $C_{19}H_{20}O_2$: calculated [M + Na]$^+$ *m/z* 303.1356, found 303.1359.

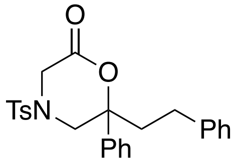

6-Phenethyl-6-phenyl-4-tosylmorpholin-2-one (6b)

Alkenoic acid **1m** (65 mg, 0.19 mmol) and potassium benzyltrifluoroborate (25 mg, 0.13 mmol) were converted to 29 mg **6b** (54% yield, light yellow oil) as above.

^{1}H NMR (400 MHz, $CDCl_3$) δ 7.54 (d, *J* = 8.0 Hz, 2H), 7.46 - 7.32 (m, 5H), 7.31 (d, *J* = 8.0 Hz, 2H), 7.26 - 7.14 (m, 3H), 7.06 (d, *J* = 7.6 Hz, 2H), 3.96, 3.67 (ABq, *J* = 18.0 Hz, 2H), 3.57, 3.47 (ABq, *J* = 12.8 Hz, 2H), 2.72 - 2.62 (m, 1H), 2.47 - 2.21 (m, 3H), 2.43 (s, 3H); ^{13}C NMR (100 MHz, $CDCl_3$) δ 164.6, 144.8, 140.7, 139.3, 131.8, 130.1, 129.0, 128.5, 128.3, 128.2, 127.7, 126.1, 124.9, 86.4, 51.0, 46.4, 41.8, 29.2, 21.6; IR (neat): 3029, 2925, 1748, 1598, 1497, 1450, 1355, 1295, 1261, 1167, 1104, 1091, 1024, 971, 912, 851, 816, 760, 734, 701, 668, 647, 566, 551 cm^{-1}; HRMS (ESI) for $C_{25}H_{25}NO_4S$: calculated [M + Na]$^+$ *m/z* 458.1397, found 458.1404.

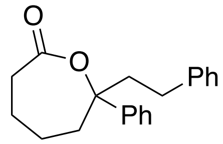

7-Phenethyl-7-phenyloxepan-2-one (7a)

Alkenoic acid **1n** (38 mg, 0.19 mmol) and potassium benzyltrifluoroborate (25 mg, 0.13 mmol) were converted to 8 mg **7a** (23% yield, clear oil) as above.

^{1}H NMR (400 MHz, $CDCl_3$) δ 7.42 (t, *J* = 6.8 Hz, 2H), 7.31 (t, *J* = 7.2 Hz, 3H), 7.21 (t, *J* = 7.2 Hz, 2H), 7.12 (t, *J* = 7.2 Hz, 1H), 7.06 (d, *J* = 7.2 Hz, 2H), 2.78 (td, *J* = 12.8, 4.4 Hz, 1H), 2.67 (d, *J* = 15.2 Hz, 1H), 2.58 (dd, *J* = 12.8, 5.6 Hz, 1H), 2.40 (td, *J* = 13.2, 3.6 Hz, 1H), 2.40 - 2.05 (m, 3H), 1.99 (td, *J* = 13.6, 4.4 Hz, 1H), 1.90 - 1.63 (m 3H), 1.62 - 1.50 (m, 1H); ^{13}C NMR (100 MHz, $CDCl_3$) δ 175.8, 141.6, 141.6, 128.9, 128.3, 128.3, 127.3, 125.8, 125.7, 86.2, 49.7, 37.7, 37.0, 29.8, 24.1, 23.2; IR (neat): 3026, 2937, 2864, 1720, 1602, 1496, 1447, 1366, 1350, 1332, 1284, 1242, 1218, 1168, 1140, 1117, 1088, 1058, 1009, 956, 939, 911, 878, 851, 799, 771, 749, 701, 672, 642, 595, 585, 558, 539, 507 cm^{-1}; HRMS (ESI) for $C_{20}H_{22}O_2$: calculated $[M + Na]^+$ *m/z* 317.1512, found 317.1514

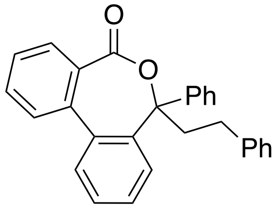

7-Phenethyl-7-phenyldibenzo[*c*,*e*]oxepin-5(7*H*)-one (7b)

Alkenoic acid **1o** (42 mg, 0.19 mmol) and potassium benzyltrifluoroborate (25 mg, 0.13 mmol) were converted to 26 mg **7b** (54% yield, light yellow oil) as above.

^{1}H NMR (500 MHz, $CDCl_3$) δ 8.00 (d, *J* = 8.0 Hz, 1H), 7.66 (t, *J* = 7.5 Hz, 1H), 7.64 - 7.55 (m, 2H), 7.54 - 7.48 (m, 3H), 7.45 (t, *J* = 8.0 Hz, 1H), 7.29 - 7.14 (m, 5H), 5.02 (dd, *J* = 10.0, 3.0 Hz, 1H), 3.24 - 3.05 (m, 1H), 2.85 - 2.66 (m, 2H), 2.42 - 2.32 (m, 1H); ^{13}C NMR (125 MHz, $CDCl_3$) δ 170.1, 140.9, 138.9, 137.3, 136.8, 132.5, 131.2, 130.8, 129.5, 129.1, 128.7, 128.5, 128.5, 128.4, 126.2, 124.2, 76.1, 76.0, 32.6, 32.2; IR (neat): 3064, 3027, 2926, 1710, 1601, 1564, 1497, 1482, 1450, 1334, 1277, 1239, 1166, 1120, 1094, 1045, 1024, 948, 910, 796, 772, 759, 740, 701, 644, 618, 582, 563 cm^{-1}; HRMS (ESI) for $C_{28}H_{22}O$: calculated $[M + Na]^+$ *m/z* 337.1199, found 337.1203.

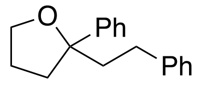

2-Phenethyl-2-phenyltetrahydrofuran (8a)

Alkenyl alcohol ***S*-8a** (30 mg, 0.19 mmol) and potassium benzyltrifluoroborate (25 mg, 0.13 mmol) were converted to 21 mg **8a** (65% yield, clear oil) as above.

^{1}H NMR (400 MHz, $CDCl_3$) δ 7.44 - 7.04 (m, 10H), 4.08 - 3.92 (m, 2H), 2.72 - 2.60 (m, 1H), 2.38 - 1.78 (m, 7H); ^{13}C NMR (125 MHz, $CDCl_3$) δ 146.5, 142.7, 128.3, 128.2, 128.1, 126.4, 125.5, 125.2, 86.5, 67.6, 44.4, 38.7, 30.8, 25.6; IR (neat): 3060, 3025, 2925, 2861, 1721, 1602, 1494, 1446, 12273, 1055, 1029, 911, 761, 700, 549 cm^{-1}; HRMS (ESI) for $C_{18}H_{20}O$: calculated [M + Na]$^+$ *m/z* 275.1406, found 275.1422.

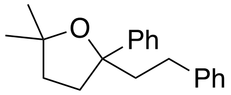

2,2-Dimethyl-5-phenethyl-5-phenyltetrahydrofuran (8b)

Alkenyl alcohol ***S*-8b** (36 mg, 0.19 mmol) and potassium benzyltrifluoroborate (25 mg, 0.13 mmol) were converted to 29 mg **8b** (84% yield, clear oil) as above.

^{1}H NMR (400 MHz, $CDCl_3$) δ 7.50 - 7.42 (m, 2H), 7.33 (t, *J* = 7.7 Hz, 2H), 7.25 - 7.18 (m, 3H), 7.17 - 7.05 (m, 3H), 2.67 (ddd, *J* = 13.7, 11.8, 5.4 Hz, 1H), 2.38 - 2.22 (m, 3H), 2.19 - 1.95 (m, 2H), 1.92 - 1.80 (m, 1H), 1.77 - 1.67 (m, 1H), 1.40 (s, 3H), 1.30 (s, 3H); ^{13}C NMR (100 MHz, $CDCl_3$) δ 148.1, 142.3, 128.3, 128.2, 127.8, 126.1, 125.5, 125.3, 86.9, 81.8, 46.0, 38.8, 38.3, 30.8, 29.6, 28.9; IR (neat): 3025, 2968, 1602, 695, 699 cm^{-1}; HRMS (ESI) for $C_{20}H_{24}O$ calculated [M + Na]$^+$ *m/z* 303.1719, found 303.1723.

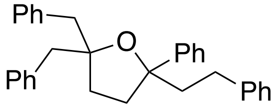

2,2-Dibenzyl-5-phenethyl-5-phenyltetrahydrofuran (8c)

Alkenyl alcohol ***S*-8c** (64 mg, 0.19 mmol) and potassium benzyltrifluoroborate (25 mg, 0.13 mmol) were converted to 23 mg **8c** (42% yield, white solid) as above.

m.p. 125 - 128 °C; ^{1}H NMR (300 MHz, $CDCl_3$) δ 7.47 (d, J = 7.8 Hz, 2H), 7.37 - 7.08 (m, 16H), 6.98 (d, J = 7.5 Hz, 2H), 3.01 - 2.73 (m, 4H), 2.68 - 2.56 (m, 1H), 2.19 - 2.04 (m, 2H), 1.96 - 1.76 (m, 5H); ^{13}C NMR (75 MHz, $CDCl_3$) δ 147.4, 142.7, 138.5, 138.3, 131.0, 130.4, 128.2, 128.1, 127.9, 127.8, 127.7, 126.3, 126.1, 126.1, 125.7, 125.4, 87.4, 87.1, 46.9, 45.7, 45.3, 37.9, 33.4, 30.7; IR (neat): 3026, 2945, 1602, 1494, 1453, 1180, 1081, 1030, 931, 752, 699, 550, 517 cm^{-1}; HRMS (ESI) for $C_{32}H_{32}O$: calculated $[M + Na]^+$ *m/z* 455.2345, found 455.2344.

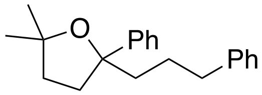

2,2-Dimethyl-5-phenyl-5-(3-phenylpropyl)tetrahydrofuran (8d)

Alkenyl alcohol ***S*-8b** (36 mg, 0.19 mmol) and potassium phenethyltrifluoroborate (27 mg, 0.13 mmol) were converted to 17 mg **8d** (46% yield, clear oil) as above.

^{1}H NMR (300 MHz, $CDCl_3$) δ 7.38 (d, J = 7.8 Hz, 2H), 7.32 - 7.07 (m, 6H), 2.52 (t, J = 7.5 Hz, 2H), 2.26 - 2.18 (m, 2H), 1.82 - 1.37 (m, 6H), 1.31 (s, 3H), 1.23 (s, 3H); ^{13}C NMR (75 MHz, $CDCl_3$) δ 148.5, 142.5, 128.4, 128.1, 127.7, 126.0, 125.5, 125.3, 87.0, 81.5, 43.7, 38.3, 36.1, 29.5, 28.9, 26.0; IR (neat): 3061, 3025, 2968, 1602, 1495, 1446, 1379, 1365, 1308, 1257, 1165, 1136, 1092, 1056, 1028, 985, 913, 890, 750, 699, 602, 559 cm^{-1}; HRMS (ESI) $C_{21}H_{26}O$: calculated $[M + Na]^+$ *m/z* 317.1876, found 317.1889.

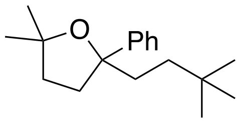

2-(3,3-Dimethylbutyl)-5,5-dimethyl-2-phenyltetrahydrofuran (8e)

Alkenyl alcohol ***S*-8b** (36 mg, 0.19 mmol) and potassium 2,2-dimethylpropyltrifluoroborate (22 mg, 0.13 mmol) were converted to 15 mg **8e** (46% yield, clear oil) as above.

^{1}H NMR (300 MHz, $CDCl_3$) δ 7.39 (d, J = 7.8 Hz, 2H), 7.30 (t, J = 7.8 Hz, 2H), 7.19 (t, J = 6.9 Hz, 1H), 2.30 - 2.17 (m, 2H), 1.88 - 1.62 (m, 4H), 1.34 (s, 3H), 1.24 (s, 3H), 1.24 - 1.13 (m, 1H), 0.99 - 0.85 (m, 1H), 0.79 (s, 9H); ^{13}C NMR (75 MHz, $CDCl_3$) δ 148.6, 127.7, 125.9, 125.3, 87.2, 81.5, 38.7, 38.6, 38.4, 37.9, 29.8, 29.6, 29.3, 28.9; IR (neat): 2954, 2867, 1602, 1491, 1446, 1379, 1364, 1305, 1248, 1166, 1137, 1087, 1063, 1025, 1007, 990, 968, 913, 888, 856, 758, 731, 701,

649, 599, 580, 519 cm^{-1}; HRMS (ESI) $C_{18}H_{28}O$: calculated $[M + Na]^+$ *m/z* 283.2032, found 283.2039.

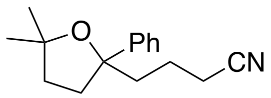

4-(5,5-Dimethyl-2-phenyltetrahydrofuran-2-yl)butanenitrile (8f)

Alkenyl alcohol ***S*-8b** (36 mg, 0.19 mmol) and potassium 2-cyanoethyltrifluoroborate (20 mg, 0.13 mmol) were converted to 22 mg **8f** (73% yield, clear oil) as above.

^{1}H NMR (400 MHz, $CDCl_3$) δ 7.38 (d, *J* = 7.2 Hz, 2H), 7.31 (t, *J* = 6.8 Hz, 2H), 7.21 (t, *J* = 7.2 Hz, 1H), 2.36 - 2.18 (m, 4H), 1.97 - 1.77 (m, 3H), 1.74 - 1.58 (m, 2H), 1.44 - 1.36 (m, 1H), 1.35 (s, 3H), 1.26 (s, 3H); ^{13}C NMR (100 MHz, $CDCl_3$) δ 147.4 128.0, 126.3, 125.2, 119.9, 86.5, 82.1, 42.6, 39.4, 38.0, 29.5, 28.8, 20.7, 17.3; IR (neat): 2968, 2245, 1601, 1492, 1446, 1380, 1366, 1309, 1259, 1168, 1134, 1093, 1058, 985, 917, 890, 759, 703, 551 cm^{-1}; HRMS (ESI) $C_{16}H_{21}ON$: calculated $[M + Na]^+$ *m/z* 266.1515, found 266.1521.

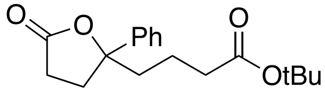

***tert*-Butyl 4-(5-oxo-2-phenyltetrahydrofuran-2-yl)butanoate (8g)**

Alkenyl alcohol ***S*-8b** (36 mg, 0.19 mmol) and potassium 3-trifluoroboratopropanoate *tert*-butyl ester (30 mg, 0.13 mmol) were converted to 27 mg **8g** (68% yield, white solid) as above, using $PhCF_3$ as a solvent and heating to 120 ˚C.

m.p. 88 - 89 ˚C; ^{1}H NMR (300 MHz, $CDCl_3$) δ 7.44 - 7.27 (m, 5H), 2.76 - 2.27 (m, 4H), 2.23 - 1.72 (m, 6H), 1.40 (s, 9H); ^{13}C NMR (75 MHz, $CDCl_3$) δ 176.5, 172.5, 142.5, 128.6, 127.6, 124.6, 89.2, 80.3, 41.5, 35.1, 28.6, 28.1, 19.5; IR (neat): 2976, 1775, 1723, 1448, 934, 702 cm^{-1}; HRMS (ESI) for $C_{18}H_{24}O_4$: calculated $[M + Na]^+$ *m/z* 327.1567, found 327.1583.

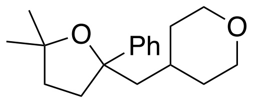

4-((5,5-Dimethyl-2-phenyltetrahydrofuran-2-yl)methyl)tetrahydro-2*H*-pyran (8h)

Alkenyl alcohol ***S*-8b** (36 mg, 0.19 mmol) and potassium tetrahydro-2*H*-pyran-4-trifluoroborate (24 mg, 0.13 mmol) were converted to 25 mg **8h** (73% yield, clear oil) as above.

^{1}H NMR (400 MHz, $CDCl_3$) δ 7.40 (d, *J* = 6.8 Hz, 2H), 7.29 (t, *J* = 7.6 Hz, 2H), 7.19 (t, *J* = 7.2 Hz, 1H), 3.82 (d, *J* = 9.6 Hz, 1H), 3.72 (d, *J* = 11.6 Hz, 1H), 3.29 - 3.11 (m, 2H), 2.26 - 2.12 (m, 2H), 1.83 - 1.60 (5H), 1.43 - 1.22 (m, 2H), 1.33 (s, 3H), 1.25 (s, 3H), 1.12 - 1.05 (m, 2H); ^{13}C NMR (100 MHz, $CDCl_3$) δ 148.4, 127.7, 126.0, 125.3, 87.1, 81.8, 68.1, 67.9, 51.1, 40.5, 40.1, 38.0, 34.6, 34.4, 31.3, 29.6, 29.0; IR (neat): 2966, 2915, 2837, 1601, 1491, 1445, 1379, 1365, 1304, 1259, 1240, 1163, 1131, 1098, 1048, 1027, 1013, 982, 965, 927, 889, 864, 760, 738, 703, 591, 563 cm^{-1}; HRMS (ESI) $C_{18}H_{26}O_2$: calculated $[M + Na]^+$ *m/z* 297.1825, found 297.1841.

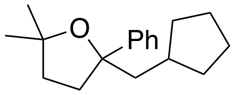

2-(Cyclopentylmethyl)-5,5-dimethyl-2-phenyltetrahydrofuran (8i)

Alkenyl alcohol ***S*-8b** (36 mg, 0.19 mmol) and potassium cyclopentyltrifluoroborate (22 mg, 0.13 mmol) were converted to 14 mg **8i** (42% yield, clear oil) as above, using 10 mol% $Cu(OTf)_2$ and 12 mol% 1, 10-phenanthroline.

^{1}H NMR (500 MHz, $CDCl_3$) δ 7.45 - 7.36 (m, 2H), 7.31 - 7.26 (m, 2H), 7.18 (t, *J* = 7.5 Hz, 1H), 2.34 - 2.06 (m, 2H), 1.96 - 1.55 (m, 6H), 1.51 - 1.28 (m, 8H), 1.23 (s, 2H), 1.14 - 1.00 (m, 1H), 0.91 - 0.76 (m, 1H); ^{13}C NMR (125 MHz, $CDCl_3$) δ 148.8, 127.6, 125.9, 125.5, 87.4, 81.4, 50.4, 39.2, 38.2, 36.4, 34.3, 33.9, 29.6, 29.0, 24.9, 24.9; IR (neat): 2966, 2868, 1447, 1379, 1137, 702 cm^{-1}; HRMS (ESI) for $C_{18}H_{26}O$: calculated $[M + Na]^+$ *m/z* 281.1876, found 281.1880.

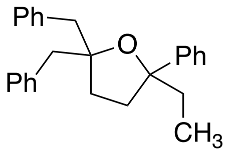

2,2-Dibenzyl-5-ethyl-5-phenyltetrahydrofuran (8j)

Alkenyl alcohol ***S*-8c** (64 mg, 0.19 mmol) and potassium methyltrifluoroborate (15 mg, 0.13 mmol) were converted to 27 mg **8j** (60% yield, clear oil) as above, using $PhCF_3$ as a solvent at 120 ˚C for 48 h.

^{1}H NMR (500 MHz, $CDCl_3$) δ 7.39 - 7.34 (m, 2H), 7.27 (d, *J* = 7.9 Hz, 2H), 7.25 - 7.22 (m, 3H), 7.21 - 7.10 (m, 7H), 2.90, 2.68 (ABq, *J* = 14.0 Hz, 2H), 2.82, 2.79 (ABq, *J* = 14.0 Hz, 2H), 2.11 - 1.99 (m, 1H), 1.83 (m, 3H), 1.73 (td, *J* = 13.0, 6.7 Hz, 1H), 1.49 (q, *J* = 7.5 Hz, 2H), 0.57 (t, *J* = 7.4 Hz, 3H); ^{13}C NMR (125 MHz, $CDCl_3$) δ 147.3, 138.6, 138.3, 131.1, 130.5, 127.9, 127.9, 127.7, 126.1, 126.0, 125.9, 110.0, 88.0, 86.7, 46.9, 45.2, 36.9, 36.3, 33.3, 8.9; IR (neat): 3027, 2968, 1602, 1493, 756, 699 cm^{-1}; HRMS (ESI) for $C_{26}H_{28}O$: calculated $[M + Na]^+$ *m/z* 379.2032, found 379.2039.

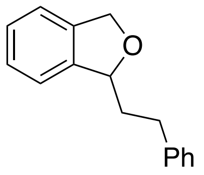

1-Phenethyl-1,3-dihydroisobenzofuran (9a)

Alkenyl alcohol ***S*-9a** (25 mg, 0.19 mmol) and potassium benzyltrifluoroborate (25 mg, 0.13 mmol) were converted to 8 mg **9a** (29% yield, clear oil) as above.

^{1}H NMR (300 MHz, $CDCl_3$) δ 7.35 - 7.08 (m, 9H), 5.27 (m, 1H), 5.18, 5.10 (ABq, *J* = 12.3 Hz, 2H), 2.81 - 2.74 (m, 2H), 2.23 - 1.96 (m, 2H); ^{13}C NMR (75 MHz, $CDCl_3$) δ 142.1, 141.9, 139.5, 128.5, 128.4, 127.4, 127.3, 125.8, 121.0, 121.0, 83.2, 72.6, 38.1, 31.4; IR (neat): 3027, 2926, 2854, 1691, 1602, 1496, 1454, 1367, 1038, 748, 700 cm^{-1}; HRMS (ESI) $C_{16}H_{16}O$: calculated $[M + Na]^+$ *m/z* 247.1093, found 247.1092.

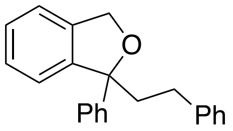

1-Phenethyl-1-phenyl-1,3-dihydroisobenzofuran (9b)

Alkenyl alcohol ***S*-9b** (39 mg, 0.19 mmol) and potassium benzyltrifluoroborate (25 mg, 0.13 mmol) were converted to 27 mg **9b** (71% yield, clear oil) as above.

^{1}H NMR (400 MHz, $CDCl_3$) δ 7.55 (d, *J* = 7.6 Hz, 2H), 7.39 - 7.10 (m, 12H), 5.24 (AB_q, 2H), $\Delta\delta_{AB}$ = 6.7 Hz, J_{AB} = 13.2 Hz), 2.70 - 2.40 (m, 4H); ^{13}C NMR (100 MHz, $CDCl_3$) δ 145.2, 144.1, 142.3, 139.2, 128.3, 128.3, 127.6, 127.5, 126.9, 125.6, 125.0, 121.9, 121.1, 91.1, 72.1, 43.7, 30.6;

IR (neat): 3025, 2915, 2848, 1601, 1495, 1456, 1446, 1354, 1269, 1185, 1156, 1123, 1019, 949, 910, 890, 840, 772, 748, 723, 698, 645, 612, 557 cm^{-1}; HRMS (ESI) $C_{22}H_{20}O$: calculated $[M + Na]^+$ *m/z* 323.1406, found 323.1408.

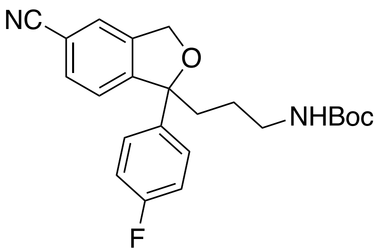

***tert*-Butyl (3-(5-cyano-1-(4-fluorophenyl)-1,3-dihydroisobenzofuran-1-yl)propyl)carbamate (9c)**

Alkenyl alcohol ***S*-9c** (48 mg, 0.19 mmol) and potassium *tert*-butyl N-[2-(trifluoroboranuidyl)ethyl]carbamate (31 mg, 0.13 mmol) were converted to 16 mg **9c** (32% yield, clear oil) as above.

^1H NMR (300 MHz, $CDCl_3$) δ 7.60 (d, J = 7.8 Hz, 1H), 7.50 (s, 2H), 7.44 – 7.35 (m, 3H), 7.05 – 6.97 (m, 2H), 5.13 (ABq, 2H, $\Delta\delta_{AB}$ = 13.4, J_{AB} = 12.9 Hz), 4.49 (bs, 1H), 3.10 (q, J = 6.3 Hz, 2H), 2.23 – 2.04 (m, 2H), 1.53 – 1.20 (m, 2H), 1.41 (s, 9H); ^{13}C NMR (75 MHz, $CDCl_3$) δ 162.1 (d, ^1J (C-F) = 245 Hz), 155.9, 149.2, 140.3, 139.3 (d, ^4J (C-F) = 3 Hz), 131.9, 126.7 (d, 3J (C-F) = 8 Hz), 125.3, 122.7, 118.5, 115.4 (d, ^2J (C-F) = 21 Hz), 111.8, 110.0, 90.9, 71.2, 40.4, 38.4, 28.4, 24.8; ^{19}F NMR (282 MHz, $CDCl_3$) δ -116.1 (s); IR (neat): 3363 (broad), 2976, 2230, 1698, 1601, 1507, 1452, 1392, 1366, 1271, 1126, 1161, 1074, 1034, 1013, 911, 833, 781, 732, 648, 598, 541 cm^{-1}; HRMS (ESI) $C_{23}H_{25}FN_2O_3$: calculated $[M + Na]^+$ *m/*z 419.1741, found 419.1752.

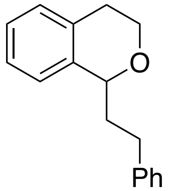

1-Phenethylisochromane (10a)

Alkenyl alcohol ***S*-10a** (28 mg, 0.19 mmol) and potassium benzyltrifluoroborate (25 mg, 0.13 mmol) were converted to 22 mg **10a** (74% yield, clear oil) as above.

^1H NMR (400 MHz, $CDCl_3$) δ 7.44 - 6.85 (m, 9H), 4.78 (dd, J = 8.4, 3.0 Hz, 1H), 4.29 - 3.99 (m, 1H), 3.93 - 3.66 (m, 1H), 3.13 - 2.91 (m, 1H), 2.85 - 2.64 (m, 3H), 2.32 - 1.99 (m, 2H); ^{13}C NMR (100 MHz, $CDCl_3$) δ 142.3, 138.1, 134.0, 128.9, 128.5, 128.3, 126.2, 126.1, 125.7, 124.7, 75.1, 63.2, 37.7, 31.5, 29.2; IR (neat): 3025, 2925, 2854, 1603, 1108, 748, 699 cm^{-1}; HRMS (ESI) for $C_{17}H_{18}O$: calculated $[M + Na]^+$ *m/z* 261.1250, found 261.1254.

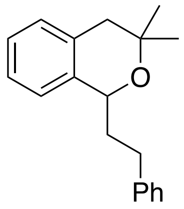

3,3-Dimethyl-1-phenethylisochromane (10b)

Alkenyl alcohol ***S*-10b** (33 mg, 0.19 mmol) and potassium benzyltrifluoroborate (25 mg, 0.13 mmol) were converted to 20 mg **10b** (61% yield, clear oil) as above.

^1H NMR (400 MHz, $CDCl_3$) δ 7.30 - 7.02 (m, 9H), 4.77 (d, J = 4.8 Hz, 1H), 2.89, 2.53 (ABq, J = 16.0 Hz, 2H), 2.83 - 2.72 (m, 1H), 2.69 - 2.59 (m, 1H), 2.32 - 2.21 (m, 1H), 2.13 - 2.01 (m, 1H), 1.39 (s, 3H), 1.15 (s, 3H); ^{13}C NMR (100 MHz, $CDCl_3$) δ 142.7, 137.4, 133.9, 129.0, 128.6, 128.2, 126.1, 126.0, 125.6, 124.0, 70.7, 70.4, 40.6, 38.0, 30.8, 30.5, 23.2; IR (neat): 3062, 3025, 2971, 2923, 2831, 1603, 1494, 1453, 1379, 1367, 1338, 1281, 1253, 1211, 1180, 1130, 1114, 1085, 1054, 1038, 975, 907, 798, 745, 699, 657, 533, 510 cm^{-1}; HRMS (ESI) for $C_{19}H_{22}O$: calculated $[M + Na]^+$ *m/z* 289.1563, found 289.1563.

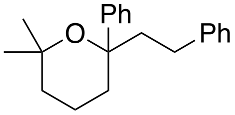

2,2-Dimethyl-6-phenethyl-6-phenyltetrahydro-2*H*-pyran (10c)

Alkenyl alcohol ***S*-10c** (38 mg, 0.19 mmol) and potassium benzyltrifluoroborate (25 mg, 0.13 mmol) were converted to 25 mg **10c** (68% yield, clear oil) as above.

^1H NMR (400 MHz, $CDCl_3$) δ 7.53 - 7.47 (m, 2H), 7.32 (t, J = 7.6 Hz, 2H), 7.28 - 7.16 (m, 3H), 7.15 - 6.99 (m, 3H), 2.50 - 2.19 (m, 3H), 2.11 (td, J = 12.8, 4.5 Hz, 1H), 1.94 (td, J = 12.8, 5.2 Hz, 1H), 1.78 - 1.62 (m, 3H), 1.59 - 1.35 (m, 2H), 1.29 (s, 3H), 0.86 (s, 3H); ^{13}C NMR (100 MHz, $CDCl_3$) δ 147.0, 142.8, 128.2, 128.1, 127.7, 126.3, 126.2, 125.4, 121.4, 76.3, 72.5, 47.7, 36.9, 32.4,

31.5, 30.0, 28.9, 16.9; IR (neat): 3026, 2937, 1603, 1108, 748, 699 cm^{-1}; HRMS (ESI) for $C_{21}H_{26}O$: calculated $[M + Na]^+$ *m/z* 317.1876, found 317.1881.

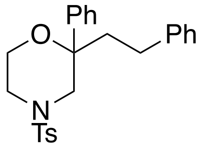

2-Phenethyl-2-phenyl-4-tosylmorpholine (10d)

Alkenyl alcohol ***S*-10d** (62 mg, 0.19 mmol) and potassium benzyltrifluoroborate (25 mg, 0.13 mmol) were converted to 32 mg **10d** (61% yield, light yellow oil) as above.

^1H NMR (400 MHz, $CDCl_3$) δ 7.58 (d, *J* = 8.4 Hz, 2H), 7.47 (d, *J* = 7.6 Hz, 2H), 7.45 - 7.38 (m, 2H), 7.35 - 7.28 (m, 3H), 7.26 - 7.15 (m, 3H), 7.05 (d, *J* = 7.2 Hz, 2H), 3.57 - 3.54 (m, 2H), 3.53 (d, *J* = 11.2 Hz, 1H), 3.06 - 2.98 (m, 1H), 2.94 - 2.63 (m, 2H), 2.44 (s, 3H), 2.44 - 2.22 (m, 3H), 2.05 - 1.94 (1H); ^{13}C NMR (100 MHz, $CDCl_3$) δ 143.8, 141.6, 140.9, 132.1, 129.8, 128.5, 128.3, 128.2, 127.8, 127.4, 126.4, 125.8, 77.4, 60.4, 52.4, 45.7, 40.6, 29.1, 21.5; IR (neat): 3027, 2924, 2852, 1599, 1495, 1453, 1350, 1339, 1305, 1289, 1244, 1220, 1165, 1134, 1120, 1090, 1027, 1001, 972, 948, 910, 847, 816, 802, 770, 754, 730, 700, 663, 647, 615, 600, 573, 548, 533 cm^{-1}; HRMS (ESI) $C_{25}H_{27}NO_3S$: calculated $[M + Na]^+$ *m/z* 444.1604, found 444.1602.

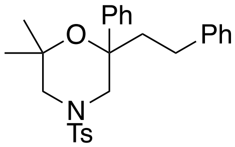

2,2-Dimethyl-6-phenethyl-6-phenyl-4-tosylmorpholine (10e)

Alkenyl alcohol ***S*-10e** (67 mg, 0.19 mmol) and potassium benzyltrifluoroborate (25 mg, 0.13 mmol) were converted to 22 mg **10e** (39% yield, light yellow oil) as above.

^1H NMR (300 MHz, $CDCl_3$) δ 7.60 - 7.56 (m, 4H), 7.39 - 7.28 (m, 4H), 7.28 - 7.15 (m, 4H), 7.02 (d, *J* = 6.9 Hz, 2H), 3.70, 2.69 (ABq, *J* = 11.7 Hz, 2H), 2.91, 2.55 (ABq, *J* = 11.1 Hz, 2H), 2.44 (s, 3H), 2.41 - 2.32 (m, 3H), 2.05 - 1.93 (m, 1H), 1.30 (s, 3H), 0.92 (s, 3H); ^{13}C NMR (75 MHz, $CDCl_3$) δ 143.8, 143.7, 141.8, 132.5, 129.8, 128.3, 128.2, 128.1, 127.7, 127.1, 126.4, 125.8, 76.2, 72.5, 55.6, 52.1, 44.2, 29.7, 28.3, 27.2, 21.6; IR (neat): 3027, 2975, 2928, 1599, 1495, 1455, 1386, 1352, 1340, 1305, 1290, 1264, 1240, 1205, 1185, 1164, 1093, 1030, 999, 977, 910, 834, 814, 767,

734, 700, 664, 598, 572, 561, 550 cm^{-1}; HRMS (ESI) $C_{27}H_{31}NO_3S$: calculated $[M + Na]^+$ *m/z* 472.1917, found 472.1918.

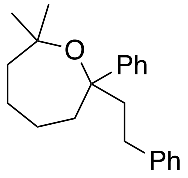

2,2-Dimethyl-7-phenethyl-7-phenyloxepane (11a)

Alkenyl alcohol ***S*-11a** (41 mg, 0.19 mmol) and potassium benzyltrifluoroborate (25 mg, 0.13 mmol) were converted to 5 mg **11a** (13% yield, clear oil) as above.

^{1}H NMR (300 MHz, $CDCl_3$) δ 7.49 (d, *J* = 8.1 Hz, 2H), 7.33 (t, *J* = 7.5 Hz, 2H), 7.26 - 7.02 (m, 6H), 2.53 (td, *J* = 13.5, 2.4 Hz, 1H), 2.38 - 1.89 (m, 6H), 1.70 - 1.50 (m, 6H), 1.41 (s, 3H), 1.19 (s, 3H); ^{13}C NMR (75 MHz, $CDCl_3$) δ 148.8, 143.3, 128.3, 128.1, 127.6, 125.8, 125.4, 81.2, 77.2, 47.1, 42.8, 39.7, 30.4, 25.4, 23.7; IR (neat): 3025, 2925, 2858, 1602, 1494, 1445, 1382, 1153, 1086, 1067, 1030, 761, 700, 636, 555 cm^{-1}; HRMS (ESI) for $C_{22}H_{28}O$: calculated $[M + Na]^+$ *m/z* 331.2032, found 331.2032.

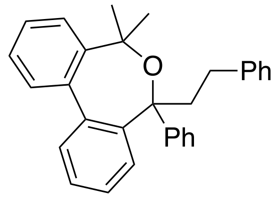

5,5-Dimethyl-7-phenethyl-7-phenyl-5,7-dihydrodibenzo[*c*,*e*]oxepine **(11b)**

Alkenyl alcohol ***S*-11b** (45 mg, 0.19 mmol) and potassium benzyltrifluoroborate (25 mg, 0.13 mmol) were converted to 28 mg **11b** (54% yield, light yellow oil) as above.

^{1}H NMR (500 MHz, $CDCl_3$) δ 8.00 (d, *J* = 8.0 Hz, 1H), 7.66 (t, *J* = 7.5 Hz, 1H), 7.64 - 7.55 (m, 2H), 7.54 - 7.48 (m, 3H), 7.45 (t, *J* = 8.0 Hz, 1H), 7.29 - 7.14 (m, 5H), 5.02 (dd, *J* = 10.0, 3.0 Hz, 1H), 3.24 - 3.05 (m, 1H), 2.85 - 2.66 (m, 2H), 2.42 - 2.32 (m, 1H); ^{13}C NMR (125 MHz, $CDCl_3$) δ 170.1, 140.9, 138.9, 137.3, 136.8, 132.5, 131.2, 130.8, 129.5, 129.1, 128.7, 128.5, 128.5, 128.4, 126.2, 124.2, 76.1, 76.0, 32.6, 32.2; IR (neat): 3064, 3027, 2926, 1710, 1601, 1564, 1497, 1482, 1450, 1334, 1277, 1239, 1166, 1120, 1094, 1045, 1024, 948, 910, 796, 772, 759, 740, 701, 644,

618, 582, 563 cm^{-1}; HRMS (ESI) for $C_{28}H_{22}O$: calculated $[M + Na]^+$ *m/z* 337.1199, found 337.1203.

General Procedure for Synthesis of Cyclic Amine Products 12 - 15

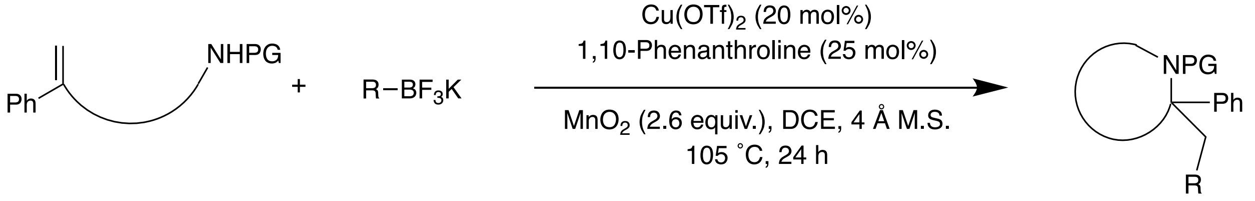

To an oven-dried pressure tube, $Cu(OTf)_2$ (4.5 mg, 0.013 mmol, 20 mol%) was flame-dried under vacuum and flushed with argon then, 1, 10-phenanthroline (2.8 mg, 0.016 mmol, 25 mol%) was added. 1, 2-Dichloroethane was then added (0.5 mL). The mixture was heated at 60 ˚C for 2 h then cooled to room temperature. Flame dried 4 Å molecular sieves (20 mg) were added and the reaction was stirred at room temperature for 10 min. Alkenyl amine (0.18 mmol, 3 equiv.), potassium alkyltrifluoroborate (0.060 mmol, 1 equiv.), and MnO_2 (16 mg, 0.16 mmol, 2.6 equiv.) were added. The tube was sealed and heated to 105 ˚C for 24 h. The reaction mixture was allowed to cool to room temperature and diluted with EtOAc (5 mL) and filtered through a pad of silica gel (~5 cm) with EtOAc (3 x 50 mL). The combined filtrate was concentrated *in vacuo* and the crude product was purified by flash chromatography on silica gel using EtOAc / hexanes.

Characterization of Novel Cyclic Amine Products 12 – 15

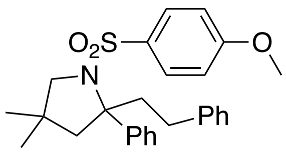

1-((4-Methoxyphenyl)sulfonyl)-4,4-dimethyl-2-phenethyl-2-phenylpyrrolidine (12a)

Alkenylamine **S-12a** (65 mg, 0.18 mmol) and potassium benzyltrifluoroborate (12 mg, 0.060 mmol) were converted to 17 mg **12a** (62% yield, clear oil) as above using $PhCF_3$ as a solvent at 120 ˚C.

^1H NMR (400 MHz, $CDCl_3$) δ 7.43 (d, J = 6.6 Hz, 2H), 7.36 - 7.18 (m, 10H), 6.73 (d, J = 8.9 Hz, 2H), 3.81 (s, 3H), 3.43 (d, J = 9.4 Hz, 1H), 3.20 (d, J = 9.4 Hz, 1H), 3.06 - 2.92 (m, 1H), 2.81 -

2.69 (m, 1H), 2.69 - 2.56 (m, 1H), 2.30 (s, 2H), 1.24 (s, 3H), 1.05 (s, 3H); ^{13}C NMR (75 MHz, $CDCl_3$) δ 162.0, 143.8, 141.9, 132.0, 129.1, 128.4, 127.9, 127.4, 126.8, 125.8, 73.7, 62.6, 55.4, 54.4, 41.7, 36.2, 32.2, 29.2, 27.9; IR (neat): 2957, 1596, 1496, 1334, 1149, 698 cm^{-1}; HRMS (ESI) for $C_{27}H_{31}NO_3S$: calculated $[M + H]^+$ *m/z* 450.2097, found 450.2125.

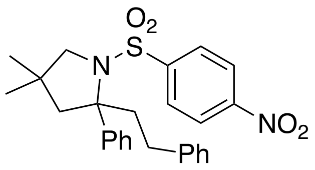

4,4-Dimethyl-1-((4-nitrophenyl)sulfonyl)-2-phenethyl-2-phenylpyrrolidine (12b)

Alkenylamine ***S*-12b** (67 mg, 0.18 mmol) and potassium benzyltrifluoroborate (12 mg, 0.060 mmol) were converted to 7 mg **12b** (24% yield, white solid) as above.

m.p. 164 – 166 ˚C; ^{1}H NMR (300 MHz, $CDCl_3$) δ 8.01 (d, *J* = 9.0 Hz, 2H), 7.39 - 7.14 (m, 12H), 3.59, 3.23 (ABq, *J* = 9.3 Hz, 2H), 3.17 - 3.04 (m, 1H), 2.79 - 2.57 (m, 3H), 2.43 - 2.33 (m, 2H), 1.34 (s, 3H), 1.18 (s, 3H); ^{13}C NMR (75 MHz, $CDCl_3$) δ 149.1, 145.1, 142.4, 141.5, 128.6, 128.3, 128.1, 127.9, 127.7, 127.5, 126.1, 123.4, 73.4, 63.1, 54.1, 41.4, 36.5, 32.4, 29.2, 27.9; IR (neat): 3059, 3026, 2958, 2868, 1738, 1638, 1602, 1578, 1496, 1468, 1445, 1395, 1292, 1230, 1141, 1127, 1075, 1029, 911, 785, 733, 698, 665, 633, 620, 589, 549 cm^{-1}; HRMS (ESI) for $C_{26}H_{28}N_2O_4S$: calculated $[M + Na]^+$ *m/z* 487.1662, found 487.1666.

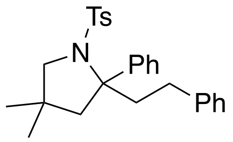

4,4-Dimethyl-2-phenethyl-2-phenyl-1-tosylpyrrolidine (12c)

Alkenylamine ***S*-12c** (62 mg, 0.18 mmol) and potassium benzyltrifluoroborate (12 mg, 0.060 mmol) were converted to 16 mg **12c** (61% yield, clear oil) as above using $PhCF_3$ as the solvent and heating to 120 ˚C.

^{1}H NMR (400 MHz, $CDCl_3$) δ 7.48 - 7.36 (m, 2H), 7.36 - 7.15 (m, 10H), 7.08 (d, *J* = 7.6 Hz, 2 H), 3.44 (d, *J* = 9.4 Hz, 1H), 3.22 (d, *J* = 9.5 Hz, 1H), 2.97 (ddd, *J* = 29.4, 18.7, 10.6 Hz, 1H), 2.72

(dd, J = 9.5, 6.0 Hz, 2H), 2.61 (dq, J = 10.7, 6.5 Hz, 1H), 2.36 (s, 3H), 2.31 (d, J = 1.8 Hz, 2H), 1.23 (s, 3H), 1.07 (s, 3H); ^{13}C NMR (75 MHz, $CDCl_3$) δ 143.8, 142.3, 141.8, 137.1, 128.9, 128.4, 128.3, 127.9, 127.4, 127.0, 126.8, 125.8, 73.9, 62.6, 54.3, 41.7, 36.2, 32.2, 29.2, 27.9; IR (neat): 3026, 2957, 2869, 1599, 1335, 1090, 660, 698 cm^{-1}; HRMS (ESI) for $C_{27}H_{32}NO_2S$: calculated [M + H]$^+$ *m/z* 434.2154, found 434.2151.

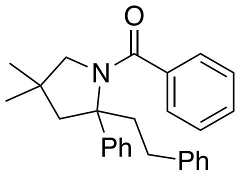

(4,4-Dimethyl-2-phenethyl-2-phenylpyrrolidin-1-yl)(phenyl)methanone (12d)

Alkenylamine ***S*-12d** (53 mg, 0.18 mmol) and potassium benzyltrifluoroborate (12 mg, 0.060 mmol) were converted to 11 mg **12d** (48% yield, white solid) as above.

m.p. 176 - 177 °C; ^{1}H NMR (300 MHz, $CDCl_3$) δ 7.58 - 7.52 (m, 2H), 7.48 - 7.41 (m, 3H), 7.36 - 7.14 (m, 9H), 3.42 (dd, J = 10.5, 8.1 Hz, 2H), 3.12 (td, J = 14.4, 4.8 Hz, 1H), 2.89 - 2.70 (m, 2H), 2.47, 2.20 (ABq, J = 12.9 Hz, 2H), 2.34 - 2.23 (m, 1H), 1.10 (s, 3H), 0.85 (s, 3H); ^{13}C NMR (75 MHz, $CDCl_3$) δ 170.0, 146.4, 141.9, 139.0, 129.2, 128.6, 128.6, 128.4, 128.0, 126.1, 126.1, 125.8, 125.8, 71.7, 65.6, 53.8, 42.2, 36.4, 31.2, 28.5, 27.5; IR (neat): 3027.5, 2960, 2870, 1738, 1605, 1528, 1497, 1454, 1348, 1314, 1205, 1161, 1090, 1046, 1030, 1012, 969, 912, 854, 763, 737, 699, 618, 609, 568, 530 cm^{-1}; HRMS (ESI) for $C_{27}H_{29}NO$: calculated [M + H]$^+$ *m/z* 384.2322, found 384.2327.

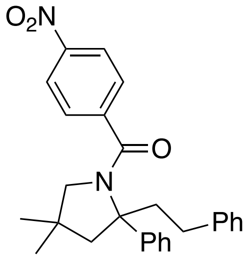

(4,4-Dimethyl-2-phenethyl-2-phenylpyrrolidin-1-yl)(4-nitrophenyl)methanone (12e)

Alkenylamine **S-12e** (61 mg, 0.18 mmol) and potassium benzyltrifluoroborate were (12 mg, 0.060 mmol) converted to 10 mg **12e** (38% yield, clear oil) as above.

^{1}H NMR (300 MHz, $CDCl_3$) δ 8.34 (d, J = 8.7 Hz, 2H), 7.70 (d, J = 8.7 Hz, 2H), 7.37 - 7.15 (m, 10H), 3.34 (q, J = 10.5 Hz, 2H), 2.99-3.14 (m, 1H), 2.78 (dd, J = 9.6, 7.1 Hz, 2H), 2.48 (d, J_{AB} = 13.2 Hz, 1H), 2.39 - 2.27 (m, 1H), 2.23 (d, J_{AB} = 12.9 Hz, 1H), 1.11 (s, 3H), 0.86 (s, 3H).; ^{13}C NMR (75 MHz, $CDCl_3$) δ 167.7, 148.0, 128.5, 128.4, 128.1, 127.1, 126.4, 126.0, 125.5, 124.1, 76.5, 68.4, 65.5, 53.5, 41.9, 36.6, 31.3, 28.3, 27.4; IR (neat): 2925, 2866, 1640, 1407, 1344, 698 cm^{-1}; HRMS (ESI) for $C_{27}H_{28}N_2NaO_3$: calculated $[M + Na]^+$ *m/z* 451.1998, found 451.1995.

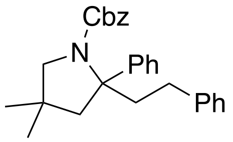

Benzyl 4,4-dimethyl-2-phenethyl-2-phenylpyrrolidine-1-carboxylate (12f)

Alkenylamine **S-12f** (58 mg, 0.18 mmol) and potassium benzyltrifluoroborate were (12 mg, 0.060 mmol) converted to 12 mg **12f** (49% yield, clear oil) as above.

^{1}H NMR (400 MHz, $CDCl_3$) reported for the rotomers δ 7.46 - 7.14 (m, 28H), 6.91 (d, J = 6.7 Hz, 2H), 5.16 (td, J = 35.7, 12.7 Hz, 4H), 3.71 (d, J = 11.6 Hz, 1H), 3.60 (d, J = 10.7 Hz, 1H), 3.45 (dd, J = 26.8, 10.9 Hz, 1H), 2.78 (t, J = 8.6, 1H), 2.62 (d, J = 12.2 Hz, 2H), 2.44 (dd, J = 32.7, 13.0 Hz, 4H), 2.37 (d, J = 12.8 Hz, 2H), 2.19 - 2.08 (m, 4H), 1.13 (d, J = 10.3 Hz, 6H), 0.83 (d, J = 18.8 Hz, 6H); ^{13}C NMR (75 MHz, $CDCl_3$) δ 155.9, 155.5, 154.4, 147.8, 147.0, 141.9, 141.6, 137.3, 128.5, 128.4, 128.2, 127.8, 125.9, 125.7, 124.5, 70.5, 67.6, 66.9, 66.6, 64.8, 63.3, 62.5, 61.6, 54.9, 53.4, 53.1, 49.5, 43.1, 42.5, 36.6, 35.6, 35.1, 30.8, 30.7, 28.6, 28.0; IR (neat): 3027, 2956, 1703, 1397, 1340, 697 cm^{-1}; HRMS (ESI) for $C_{28}H_{32}NO_2$: calculated $[M + H]^+$ *m/z* 414.2433, found 414.2429.

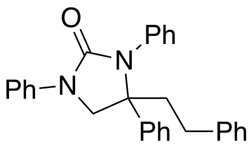

4-Phenethyl-1,3,4-triphenylimidazolidin-2-one (12g)

Alkenylamine ***S*-12g** (59 mg, 0.18 mmol) and potassium benzyltrifluoroborate (12 mg, 0.060 mmol) were converted to 14 mg **12g** (56% yield, white solid) as above.

m.p. 87 - 89 °C; ^{1}H NMR (400 MHz, $CDCl_3$) δ 7.75 (d, *J* = 8.4 Hz, 2H), 7.44 - 7.32 (m, 9H), 7.28 - 7.21 (m, 4H), 7.19 - 7.13 (m, 1H), 7.12 - 7.03 (m, 4H), 4.17 (dd, *J* = 8.4 , 6.4 Hz, 2H), 2.77 - 2.66 (m, 1H), 2.42 - 2.32 (m, 3H); ^{13}C NMR (100 MHz, $CDCl_3$) δ 159.4, 148.0, 147.3, 142.2, 140.9, 139.6, 128.8, 128.6, 128.5, 128.2, 128.0, 126.1, 124.4, 123.5, 122.9, 122.4, 118.8, 83.7, 58.1, 43.1, 29.9; IR (neat): 3029, 1675, 1590, 1500, 1449, 1403, 1318, 1217, 1134, 1092, 1028, 980, 900, 752, 694, 510 cm^{-1}; HRMS (ESI) for $C_{29}H_{26}N_2O$: calculated $[M + H]^+$ *m/z* 419.2118, found 419.2116.

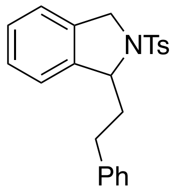

1-Phenethyl-2-tosylisoindoline (13)

Alkenylamine ***S*-13** (52 mg, 0.18 mmol) and potassium benzyltrifluoroborate (12 mg, 0.060 mmol) were converted to 8 mg **13** (37% yield, light yellow oil) as above.

^{1}H NMR (400 MHz, $CDCl_3$) δ 7.72 (d, *J* = 8.4 Hz, 2H), 7.29 - 7.08 (m, 11H), 5.06 (s, 1H), 4.73, 4.64 (ABq, *J* = 14.0 Hz, 2H), 2.77 (td, *J* = 12.8, 4.0 Hz, 1H), 2.59 - 2.48 (m, 1H), 2.37 (s, 3H), 2.33 - 2.11 (m, 2H); ^{13}C NMR (100 MHz, $CDCl_3$) δ 143.5, 141.8, 139.7, 135.9, 134.7, 129.7, 128.4, 127.8, 127.8, 127.4, 125.7, 122.4, 122.3, 65.5, 54.2, 38.0, 29.7, 21.5; IR (neat): 3027, 2971, 1739, 1598, 1455, 1366, 1349, 1229, 1217, 1163, 1094, 1057, 815, 751, 700, 667, 617, 561, 528 cm^{-1}; HRMS (ESI) for $C_{23}H_{23}NO_2S$: calculated $[M + H]^+$ *m/z* 378.1522, found 378.1528.

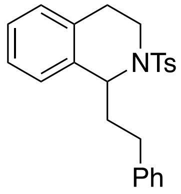

1-Phenethyl-2-tosyl-1,2,3,4-tetrahydroisoquinoline (14)

Alkenylamine ***S*-14** (54 mg, 0.18 mmol) and potassium benzyltrifluoroborate (12 mg, 0.060 mmol) were converted to 13 mg **14** (56% yield, clear oil) as above.

^{1}H NMR (400 MHz, $CDCl_3$) δ 7.59 (d, *J* = 8.4 Hz, 2H), 7.30 -7.23 (m, 2H), 7.23 - 7.14 (m, 3H), 7.14 - 6.98 (m, 5H), 6.85 (d, *J* = 7.2 Hz, 1H), 5.04 (dd, *J* = 8.8, 4.4 Hz, 1H), 3.89 (td, *J* = 14.4, 4.4 Hz, 1H), 3.56 - 3.47 (m, 1H), 2.88 - 2.74 (m, 2H), 2.55 - 2.49 (m, 2H), 2.31 (s, 3H), 2.18 - 1.97

(m, 2H); ^{13}C NMR (100 MHz, $CDCl_3$) δ 143.0, 141.7, 137.8, 136.5, 132.6, 129.3, 128.9, 128.3, 127.0, 126.9, 126.6, 126.1, 125.8, 56.6, 39.4, 38.9, 32.8, 26.2, 21.4; IR (neat): 3025, 2925, 1599, 1494, 1453, 1378, 1333, 1305, 1272, 1208, 1184, 1155, 1120, 1091, 1066, 1011, 942, 909, 814, 763, 730, 710, 699, 661, 635, 596, 562, 549 cm^{-1}; HRMS (ESI) for $C_{24}H_{25}NO_2S$: calculated $[M + H]^+$ *m/z* 392.1679, found 392.1682.

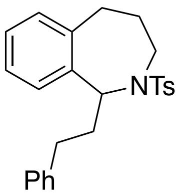

1-Phenethyl-2-tosyl-2,3,4,5-tetrahydro-1*H*-benzo[*c*]azepine (15)

Alkenylamine ***S*-15** (114 mg, 0.36 mmol) and potassium benzyltrifluoroborate (24 mg, 0.12 mmol) were converted to 16 mg **15** (31% yield, clear oil) as above.

^1H NMR (400 MHz, $CDCl_3$) δ 7.43 (d, *J* = 8.0 Hz, 2H), 7.29 - 7.23 (m, 3H), 7.22 - 7.04 (m, 7H), 7.00 - 6.95 (m, 1H), 5.10 (s, 1H), 4.05 (d, *J* = 12.4 Hz, 1H), 3.45 (t, *J* = 12.8 Hz, 1H), 3.10 (t, *J* = 13.6 Hz, 1H), 2.70 - 2.46 (m, 3H), 2.34 (s, 3H), 2.34 - 2.12 (m, 2H), 1.78 - 1.68 (m, 1H), 1.49 - 1.36 (m, 1H); ^{13}C NMR (100 MHz, $CDCl_3$) δ 142.6, 141.2, 140.4, 139.8, 138.2, 130.8, 129.8, 129.2, 128.4, 128.4, 127.4, 127.1, 126.3, 126.0, 62.8, 34.9, 33.0, 32.9, 30.9, 27.4, 21.4; IR (neat): 3026, 2938, 1599, 1494, 1453, 1330, 1305, 1199, 1155, 1110, 1093, 981, 911, 862, 814, 753, 729, 700, 660, 604, 582, 560 cm^{-1}; HRMS (ESI) for $C_{25}H_{27}NO_2S$: calculated $[M + H]^+$ *m/z* 406.1835, 406.1850.

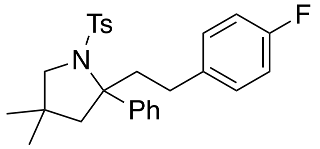

2-(4-Fluorophenethyl)-4,4-dimethyl-2-phenyl-1-tosylpyrrolidine (12h)

Alkenylamine ***S*-12c** (62 mg, 0.18 mmol) and potassium 4-fluorobenzylborate (13 mg, 0.060 mmol) were converted to 14 mg **12h** (50% yield, clear oil) as above.

^{1}H NMR (300 MHz, $CDCl_3$) δ 7.42 - 7.37 (m, 2H), 7.28 - 7.16 (m, 8H), 7.07 (d, J = 7.5 Hz, 2H), 6.98 (t, J = 8.4 Hz, 2H), 3.44, 3.20 (ABq, J = 9.3 Hz, 2H), 2.97 - 2.55 (m, 4H), 2.36 (s, 3H), 2.30 (dd, J = 13.5, 5.1 Hz, 2H), 1.23 (s, 3H), 1.06 (s, 3H); ^{13}C NMR (75 MHz, $CDCl_3$) δ 162.9, 143.9, 142.4, 137.5, 137.2, 129.8, 129.7, 129.0, 127.9, 127.3, 129.1, 126.9, 115.3, 115.0, 73.9, 62.7, 54.5, 42.0, 36.3, 31.4, 29.2, 27.9, 21.4; ^{19}F NMR (282 MHz, $CDCl_3$) δ -118.6 (s); IR (neat): 2959, 2871, 1600, 1509, 1446, 1369, 1336, 1304, 1219, 1155, 1091, 1048, 1028, 1015, 969, 914, 815, 760, 732, 701, 661, 614, 584, 575, 546, 521, 508 cm^{-1}; HRMS (ESI) for $C_{27}H_{30}FNO_2S$: calculated $[M + H]^+$ *m/z* 452.2054, found 452.2056.

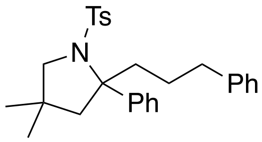

4,4-Dimethyl-2-phenyl-2-(3-phenylpropyl)-1-tosylpyrrolidine (12i)

Alkenylamine ***S*-12c** (62 mg, 0.18 mmol) and potassium phenethyltrifluoroborate (13 mg, 0.060 mmol) were converted to 13 mg **12i** (48% yield, light yellow oil) as above.

^{1}H NMR (500 MHz, $CDCl_3$) δ 7.38 - 7.34 (m, 2H), 7.30 - 7.26 (m, 4H), 7.23 - 7.14 (m, 6H), 7.09 (d, J = 8.0 Hz, 2H), 3.34, 3.19 (ABq, J = 10.0 Hz, 2H), 2.79 - 2.72 (m, 1H), 2.69 - 2.64 (m, 1H), 2.36 (s, 3H), 2.27 - 2.18 (m, 1H), 2.19, 2.10 (ABq, J = 14..0 Hz, 2H), 1.74 - 1.55 (m, 2H), 1.10 (s, 3H), 1.00 (s, 3H); ^{13}C NMR (125 MHz, $CDCl_3$) δ 144.1, 142.3,142.1, 137.5, 129.0, 128.4, 127.8, 127.4, 127.0, 126.7, 125.8, 74.1, 62.6, 54.1, 39.7, 36.3, 36.1, 29.0, 28.0, 27.8; IR (neat): 3026, 2957, 2869, 2254, 1599, 1495, 1453, 1369, 1336, 1304, 1288, 1207, 1154, 1091, 1031, 1016, 970, 910, 814, 761, 731, 699, 661, 614, 583, 547 cm^{-1}; HRMS (ESI) for $C_{28}H_{33}NO_2S$: calculated $[M + H]^+$ *m/z* 448.2305, found 448.2300.

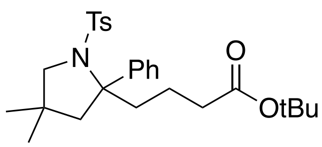

***tert*-Butyl 4-(4,4-dimethyl-2-phenyl-1-tosylpyrrolidin-2-yl)butanoate (12j)**

Alkenylamine ***S*-12c** (62 mg, 0.18 mmol) and potassium 3-trifluoroboratopropanoate *tert*-butyl ester (14 mg, 0.060 mmol) were converted to **12j** (13 mg, 45% yield, clear oil) as above.

^{1}H NMR (400 MHz, $CDCl_3$) δ 7.38 (d, *J* = 6.2 Hz, 2H), 7.24 (dd, *J* = 20.7, 7.1 Hz, 5H), 7.09 (d, *J* = 7.9 Hz, 2H), 3.36 (d, *J* = 9.4 Hz, 1H), 3.17 (d, *J* = 9.3 Hz, 1H), 2.72 (t, *J* = 10.0, 1H), 2.36 (s, 3H), 2.32 - 2.12 (m, 5H), 1.54 (s, 2H), 1.45 (s, 9H), 1.18 (s, 3H), 1.02 (s, 3H); ^{13}C NMR (75 MHz, $CDCl_3$) δ 172.7, 143.7, 142.3, 137.3, 128.9, 127.8, 127.4, 127.0, 126.7, 80.2, 73.9, 62.5, 53.9, 39.4, 36.2, 35.5, 29.1, 28.1, 27.8, 21.5, 21.4; IR (neat): 2971, 1725, 1339, 1155, 661 cm^{-1}; HRMS (ESI) for $C_{27}H_{37}NO_4S$: calculated $[M + Na]^+$ *m/z* 494.2341, found 494.2347.

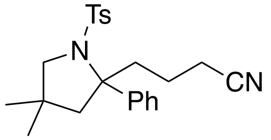

4-(4,4-Dimethyl-2-phenyl-1-tosylpyrrolidin-2-yl)butanenitrile (12k)

Alkenylamine ***S*-12c** (62 mg, 0.18 mmol) and potassium 2-cyanoethyltrifluoroborate (10 mg, 0.060 mmol) were converted to 10 mg **12k** (43% yield, clear oil) as above.

^{1}H NMR (300 MHz, $CDCl_3$) δ 7.39 - 7.28 (m, 4H), 7.26 - 7.20 (m, 3H), 7.13 (d, *J* = 7.5 Hz, 2H), 3.38, 3.17 (ABq, *J* = 9.3 Hz, 2H), 2.79 - 2.66 (m, 1H), 2.48 - 2.36 (m, 3H), 2.38 (s, 3H), 2.26, 2.11 (ABq, *J* = 22.5 Hz, 2H), 1.86 - 1.71 (m, 2H), 1.18 (s, 3H), 1.01 (s, 3H); ^{13}C NMR (75 MHz, $CDCl_3$) δ 143.6, 142.7, 137.0, 129.2, 128.1, 127.1, 127.0, 119.4, 77.2, 73.5, 63.0, 54.5, 39.4, 36.2, 29.2, 27.8, 22.2, 21.4, 17.5; IR (neat): 2959, 2871, 2245, 1599, 1495, 1446, 1369, 1335, 1305, 1289, 1209, 1154, 1091, 1055, 1035, 1016, 971, 914, 815, 762, 732, 703, 661, 584, 547 cm^{-1}; HRMS (ESI) for $C_{22}H_{28}N_2O_2S$: calculated $[M + H]^+$ *m/z* 397.1944, found 397.1948.

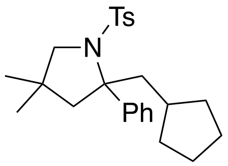

2-(Cyclopentylmethyl)-4,4-dimethyl-2-phenyl-1-tosylpyrrolidine (12l)

Alkenylamine ***S*-12c** (62 mg, 0.18 mmol) and potassium cyclopentyltrifluoroborate (11 mg, 0.060 mmol) were converted to 21 mg **12l** (84% yield, clear oil) as above using $PhCF_3$ as a solvent at 120 ˚C. ^{1}H NMR (400 MHz, $CDCl_3$) δ 7.41 (d, *J* = 12.4 Hz, 2H), 7.31 - 7.15 (m, 5H), 7.08 (d, *J* = 8.0 Hz, 2H), 3.37 (d, *J* = 9.5 Hz, 1H), 3.11 (d, *J* = 9.6, 1H), 3.06 (dd, *J* = 14.3, 3.8 Hz 1H), 2.36 (s, 3H), 2.29 (d, *J* = 9.6 Hz, 1H), 2.14 (dd, *J* = 14.3, 6.8 Hz, 1H), 1.92 - 1.75 (m, 2H), 1.73 - 1.56 (m, 2H), 1.54 (s, 2H), 1.49 - 1.36 (m, 2H), 1.30 - 1.13 (m, 5H), 1.01 (s, 3H); ^{13}C NMR (75 MHz, $CDCl_3$) δ 143.9, 142.1, 137.6, 128.9, 127.7, 127.0, 126.7, 74.8, 62.2, 53.4, 46.2, 37.4, 36.3, 35.1, 34.9, 29.1, 27.7, 25.2, 24.5, 21.4; IR (neat): 2952, 2867, 1334, 1153, 6699 cm^{-1}; HRMS (ESI) for $C_{25}H_{34}NO_2S$: calculated $[M + H]^+$ *m/z* 412.2310, found 412.2306.

Procedure for the Enantioselective Carbolactonization

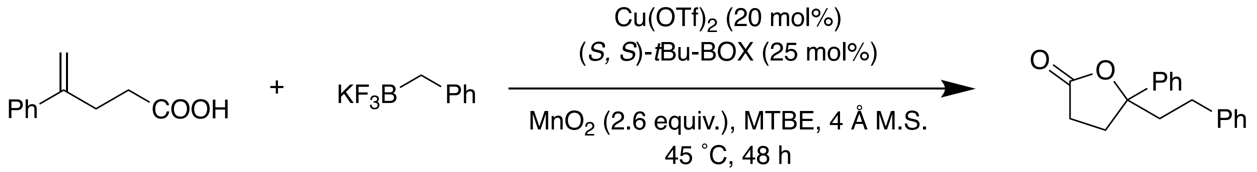

To an oven-dried pressure tube, $Cu(OTf)_2$ (9 mg, 0.025 mmol, 20 mol%) was flame-dried under vacuum and flushed with argon then, (*S, S*)-*tert*-Bu-bisoxazoline (9.4 mg, 0.031 mmol, 25 mol%) was added. Methyl *tert*-butyl ether was then added (1 mL). The mixture was stirred at room temperature for 2 h. Flame dried 4 Å molecular sieves (20 mg) were added and the reaction was stirred at room temperature for 10 min. **1b** (33 mg, 0.19 mmol, 1.5 equiv.), potassium benzyltrifluoroborate (25 mg, 0.13 mmol, 1 equiv.), and MnO_2 (33 mg, 0.32 mmol, 2.6 equiv.) were added. The tube was sealed and heated to 45 ˚C for 48 h. The reaction mixture was allowed to cool to room temperature and diluted with EtOAc (5 mL) and filtered through a pad of silica gel (~5 cm) with EtOAc (3 x 50 mL). The combined filtrate was concentrated *in vacuo* and the crude product was purified by flash chromatography on silica gel (20% EtOAc / hexanes) to afford 14 mg **5a** (42% yield).

$[\alpha]_D^{26}$ = -23.6 (c = 0.41 in $CHCl_3$); *ee* = 44%, determined by GC analysis (CP-Chirasil-Dex CB column), T_{inj} = 200 ˚C, T_{det} = 220 ˚C, flow = 2 mL/min, t_i = 160 ˚C, t_f = 195 ˚C, rate = 1.0 ˚C/min for 20 mins, then hold at 180 ˚C for 5 mins, then 0.4 ˚C/min for 37.5 mins, retention times using He as carrier gas: t_{minor} = 58.64 mins, t_{major} = 59.76 mins.

GC Traces:

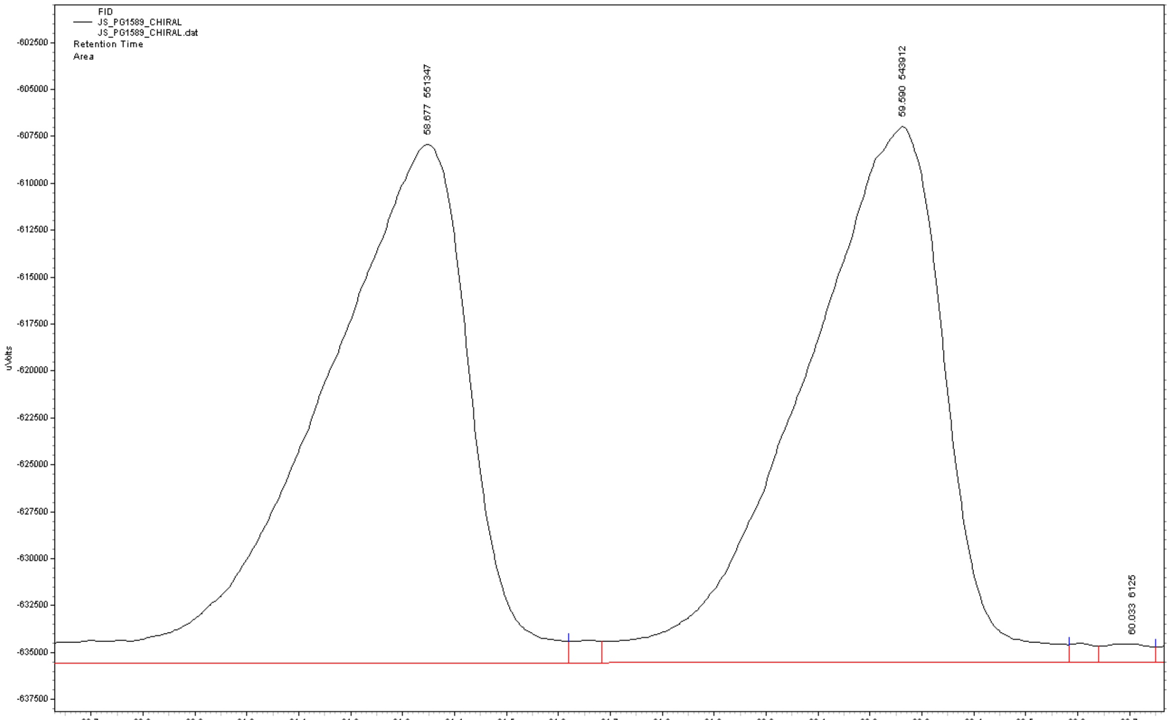

#	Time (min)	Area (%)
1	58.68	50.3
2	59.59	49.7

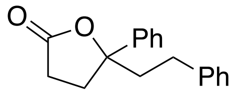

5a

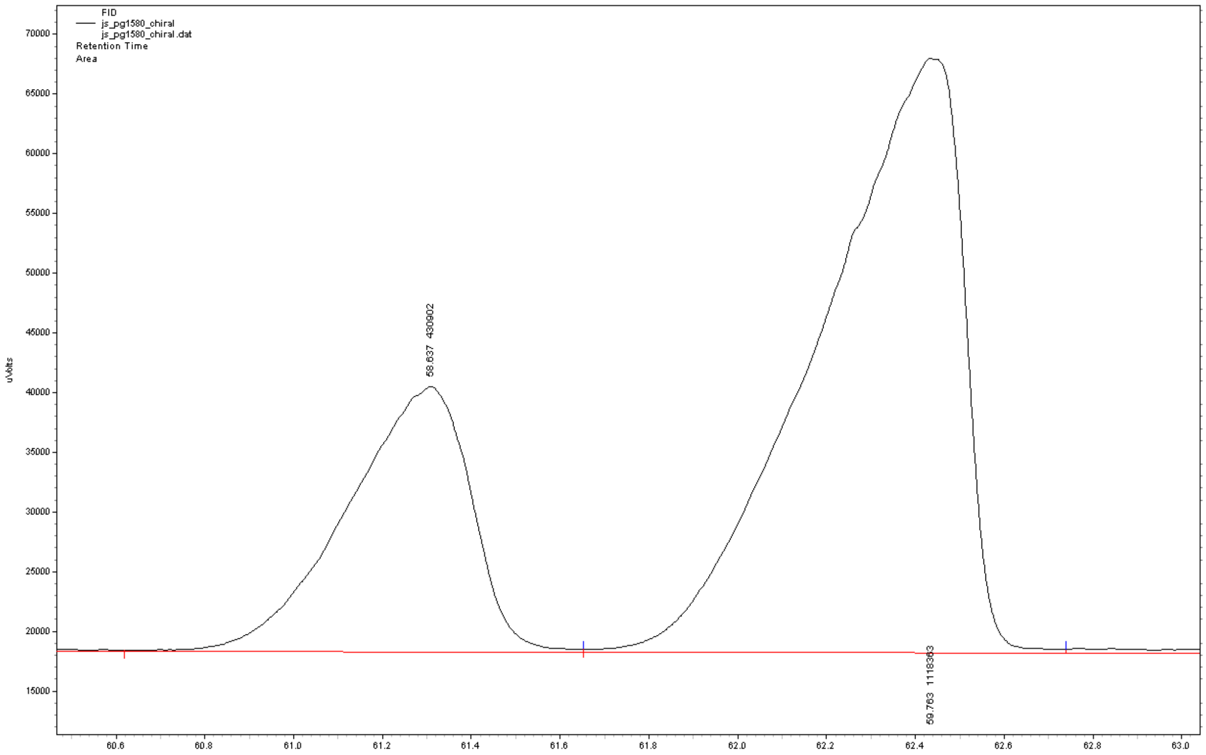

#	Time (min)	Area (%)
1	58.64	27.8
2	59.76	72.2

MnO_2 - Only Oxidative Cyclization Representative Procedure

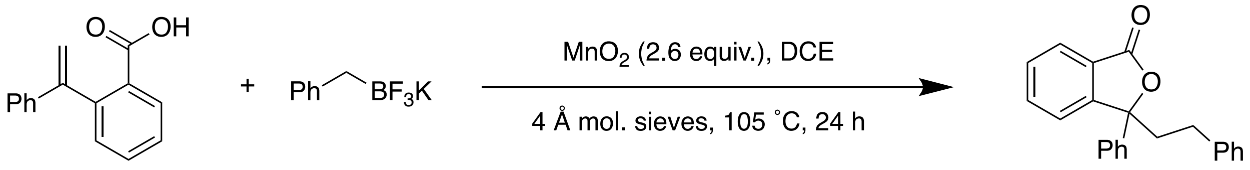

To an oven-dried pressure tube 1, 2-dichloroethane was added (1 mL). Then, flame dried 4 Å molecular sieves (20 mg) were added and the mixture was stirred at room temperature for 10 min. 2-(1-Phenylvinyl)benzoic acid (33 mg, 0.19 mmol, 1.5 equiv.), potassium benzyltrifluoroborate (25 mg, 0.13 mmol, 1 equiv.), and MnO_2 (33 mg, 0.32 mmol, 2.6 equiv.) were added. The tube was sealed and heated to 105 ˚C for 24 h. The reaction mixture was allowed to cool to room temperature and diluted with EtOAc (5 mL) and filtered through a pad of silica gel (~5 cm) with

EtOAc (3 x 50 mL). The combined filtrate was concentrated *in vacuo* and the crude product was purified by flash chromatography on silica gel using EtOAc / hexanes (20%) to afford 20 mg **2a** in 51% yield.

References

1. Miao, L.; Haque, I.; Manzoni, M. R.; Tham, W. S.; Chemler, S. R.; *Org. Lett.* **2010,** *12* (21), 4739-4741.
2. Sha, W.; Zhang, W.; Ni, S.; Mei, H.; Han, J.; Pan, Y.; *J. Org. Chem.* **2017,** *82* (18), 9824-9831.
3. Zhu, R.; Buchwald, S. L.; *J. Am. Chem. Soc.* **2012,** *134* (30), 12462-12465.
4. Dydio, P.; Reek, J. N. H.; *Angew. Chem. Inter. Ed.* **2013,** *52* (14), 3878-3882.
5. Zhu, R.; Buchwald, S. L., *Angew. Chem. Inter. Ed.* **2013,** *52* (48), 12655-12658.
6. Zhu, R.; Buchwald, S. L.; *J. Am. Chem. Soc.* **2015,** *137* (25), 8069-8077.
7. Du, K.; Kier, M. J.; Rheingold, A. L.; Micalizio, G. C.; *Org. Lett.* **2018,** *20* (20), 6457-6461.
8. Cui, J.; Jin, J.; Hsieh, Y.-H.; Yang, H.; Ke, B.; Damera, K.; Tai, P. C.; Wang, B.; *Chem. Med. Chem.* **2013,** *8* (8), 1384-1393.
9. Kawauchi, D.; Ueda, H.; Tokuyama, H.; *Eur. J. Org. Chem.* **2019,** *2019* (10), 2056-2060.
10. Bose, D.; Denmark, S. E.; *Synlett* **2018,** *29* (04), 433-439.
11. Hemric, B. N.; Chen, A. W.; Wang, Q.; *J. Org. Chem.* **2019,** *84* (3), 1468-1488.
12. Hewitt, J. F. M.; Williams, L.; Aggarwal, P.; Smith, C. D.; France, D. J.; *Chem. Sci.* **2013,** *4* (9), 3538-3543.
13. Marsault, E.; Hoveyda, H. R.; Peterson, M. L.; Saint-Louis, C.; Landry, A.; Vezina, M.; Ouellet, L.; Wang, Z.; Ramaseshan, M.; Beaubien, S.; Benakli, K.; Beauchemin, S.; Deziel, R.; Peeters, T.; Fraser, G. L., *J. Med. Chem.* **2006,** *49* (24), 7190-7197.
14. Lin, J.-S.; Yu, P.; Huang, L.; Zhang, P.; Tan, B.; Liu, X.-Y.; *Angew. Chem. Inter. Ed.* **2015,** *54* (27), 7847-7851.
15. Yu, W. Z.; Cheng, Y. A.; Wong, M. W.; Yeung, Y.-Y.; *Adv. Synth. Catal.* **2017,** *359* (2), 234-239.
16. Sherman, E. S.; Fuller, P. H.; Kasi, D.; Chemler, S. R.; *J. Org. Chem.* **2007,** *72* (10), 3896-3905.
17. Zhang, S.; Li, L.; Wang, H.; Li, Q.; Liu, W.; Xu, K.; Zeng, C.; *Org. Lett.* **2018,** *20* (1), 252-255.
18. Fukuyama, T.; Bando, T.; Ryu, I.; *Synthesis* **2018,** *50* (15), 3015-3021.
19. Jeffrey, J. L.; Terrett, J. A.; MacMillan, D. W. C.; *Science* **2015,** *349* (6255), 1532-1536.
